# Mendelian Randomization and Double Machine Learning Modeling Reveal Brain Imaging‐Derived Phenotypes as Functional Contributors to 18 Autoimmune Inflammatory Diseases

**DOI:** 10.1002/advs.202515675

**Published:** 2025-12-25

**Authors:** Jinbin Chen, Xin Wang, Haifeng Ding, Bosheng Zheng, Keni Zeng, Chuying Hu, Jiayi Liu, Xiao Zhu, Haibing Yu

**Affiliations:** ^1^ Dongguan Key Laboratory of Chronic Disease Prevention and Control The First Dongguan Affiliated Hospital School of Public Health Guangdong Medical University Dongguan Guangdong China; ^2^ Affiliated Hospital Group of Guangdong Medical University, Shenzhen Baoan Central Hospital (Baoan Central Hospital of Shenzhen) Shenzhen Guangdong China; ^3^ The First Dongguan Affiliated Hospital of Guangdong Medical University Dongguan Guangdong China; ^4^ The Second Affiliated Hospital of Guangdong Medical University School of Ocean and Tropical Medicine Guangdong Medical University Zhanjiang Guangdong China

**Keywords:** autoimmune inflammatory diseases, brain functional networks, double machine learning, imaging‐derived phenotype, Mendelian randomization

## Abstract

Autoimmune inflammatory diseases (AIDs) are genetically linked disorders with unclear causal links to brain functional networks. Using bidirectional two‐sample Mendelian randomization (MR) on GWAS data from 18 AIDs and 1,366 brain imaging‐derived phenotypes (*n* = 8,428), we identified significant associations, including reduced left striatal activity increasing multiple sclerosis risk (OR = 0.59), left uncinate fasciculus activity elevating systemic lupus erythematosus risk (OR = 3.72), and asymmetric cerebellar peduncle effects in cutaneous vasculitis (left: OR = 0.11; right: OR = 8.57) [exploratory finding with 24.8%–37.8% power]. Fibromyalgia suppressed cerebellar area VIIIa (β = −0.023). Sensitivity analyses, double machine learning, and >99% statistical power supported robustness. These findings suggest alterations in default mode, salience, and central executive networks contribute to AIDs pathogenesis, highlighting brain regions such as the striatum and cerebellar peduncles as potential therapeutic targets.

## Graphical abstract

This schematic integrates the eight statistically significant causal relationships identified between 1,366 brain imaging‐derived phenotypes (IDPs) and 18 autoimmune inflammatory diseases (AIDs). Arrows indicate the direction of causality inferred from bidirectional two‐sample MR analyses. Statistical tests: Causal estimates are derived using inverse variance weighted (IVW) MR as the primary method, with sensitivity analyses including MR‐Egger, weighted median, MR‐PRESSO, and leave‐one‐out tests. DML validation employed orthogonalized cross‐fitting with Lasso and Random Forest regressors. Data presentation: Results are shown as ORs or β values with 95% confidence intervals. Significance thresholds are Bonferroni‐corrected (forward MR: P < 5.75 × 10^−4^; reverse MR: P < 1.67 × 10^−2^). No significance symbols are used in this figure.

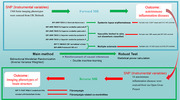

## 1 Introduction

AIDs, or autoimmune disorders, are diseases caused by immune reactions attacking normal cells of the body, which can extensively invade the joints, muscles, bones, connective tissues, and peripheral soft tissues, and accumulate in the organs [1].

It is noteworthy that the nervous system is a common site of involvement in AIDs [2, 3]. One of its defining features is brain structure. Clinical and neuroimaging evidence, such as brain lesions observed in systemic lupus erythematosus (SLE) and Behçet's disease, strongly suggests an association between AIDs and alterations in brain structure and function [4].

A functional brain network is formed by connecting different regions of the brain structure, which is the basis of human advanced cognition, and the connection between functional brain networks is indispensable for the human brain to perform complex functions [5, 6]. However, an increasing number of observational studies employing techniques such as magnetic resonance imaging (MRI) have further revealed associations between AIDs and specific functional brain networks, such as multiple sclerosis (MS), which often triggers abnormal cortical activity, further affecting cortico‐cortical and cortico‐subcortical circuit connections [7]; The strength of functional connectivity in the inferior frontal‐parietal cortex is positively correlated with disease activity in patients with SLE [8]. However, a critical question remains unresolved: Are these brain features causal factors in the pathogenesis of AIDs, or merely consequences of chronic inflammation, drug effects, or other confounding factors? Traditional observational studies face inherent limitations in addressing this causality question due to potential reverse causality and unmeasured confounders.

MR has emerged as a powerful methodological framework to address this limitation. By employing genetic variation—typically single nucleotide polymorphisms (SNPs)—as instrumental variables, MR leverages the random allocation of alleles at conception to minimize confounding factors and help establish causal directionality, thereby providing more robust evidence for causality [9]. Although pioneering studies have begun applying MR to explore relationships among brain features, a comprehensive assessment of causal relationships between multiple brain features and various AIDs remains lacking. This indicates significant knowledge gaps in understanding the pathogenic pathways of AIDs from a neuroimmunological perspective.

Furthermore, conventional MR methods rely on stringent assumptions, and violations such as horizontal pleiotropy can bias results. To bolster the robustness of our causal inferences, we integrated Double Machine Learning (DML), a cutting‐edge approach from the causal inference literature [10]. DML combines flexible machine learning algorithms with semiparametric theory to control for high‐dimensional confounding nonparametrically, providing an independent validation that is more robust to model misspecification. The integration of bidirectional two‐sample MR with DML represents a methodological advance, allowing us to dissect the causal interplay between the brain and AIDs with greater confidence and rigor than previously possible.

Therefore, this study employed bidirectional two‐sample MR analysis using large‐scale genome‐wide association study (GWAS) data from 1,366 brain structural genetics cases and 18 AIDs cases. Our objectives were: (1) to determine the causal effects of brain structure and function on AID risk; (2) to investigate the reverse causality of AIDs on brain alterations; and (3) to validate these findings using DML. Our work aims to elucidate the genetic mechanisms of AIDs from a brain‐organism genetics perspective and identify potential neuromodulatory therapeutic targets.

## 2 Methods

### 2.1 GWAS for Imaging‐Derived Phenotypes

Changes in image‐derived phenotypes (IDP) of brain structures have been widely reported in AIDs. To explore the potential causal relationship between brain structure and AIDs, we used the IDP dataset from reference 11 in our study [11]. Specifically, reference 11 analyzed a sample of genetic and brain imaging data (*n* = 8428) from the UK Biobank (UKB) covering 3,144 IDPs of brain structure for a whole GWAS and detected 11 734 353 SNPs in the samples. This study delved into various dimensions of brain structure, revealed significant genetic effects on brain structure and function, and successfully identified 148 associated SNP clusters. They identified 1,578 out of 3,144 IDPs that showed significant SNP genetic effects and 1,262 SNPs significantly associated with IDPs of the brain. They validated the major genetic associations in the discovery phase using two independent data sets obtained from UKB. In addition, the study performed heritability analyses of GWAS and SNPs for multiple traits, IDP genetic correlation analyses for brain‐related traits, and enrichment analyses of different functional genomic regions, which provided a solid theoretical foundation for this study. We screened the GWAS summary statistics of 1366 IDPs and 18 AIDs from reference 11 for bidirectional two‐sample MR analysis. Among these, 1,366 IDPs were selected based on the principle of “prioritizing phenotypes associated with well‐defined, classical brain structures or functions.” We systematically extracted phenotype data directly linked to well‐defined brain regions (e.g., hippocampal volume, prefrontal cortex thickness), specific white matter tracts (e.g., corpus callosum fractional anisotropy), and classical functional networks. Concurrently, we proactively excluded phenotypes with ambiguous identifiers that could not be mapped to specific neuroanatomical structures or cognitive functions. Such phenotypes, due to their unclear biological significance, may introduce noise and complicate result interpretation and were therefore excluded. We have classified the features of 1,366 IDPs and presented them in Table 1. Detailed information on the 18 AIDs is shown in Table S1.

**TABLE 1:** Categorization and Characterization of Brain Imaging‐Derived Phenotypes (IDPs).

Primary imaging modality	IDP feature name	Classification category	Specific metric/Brief description of biological significance	Quantity
T1‐weighted MRI	a2009s	Structural Morphology	Surface area of cortical regions in the left hemisphere based on a specific atlas (e.g., 2009 atlas), reflecting regional cortical size.	296
	DKTatlas		Cortical thickness or volume of regions defined by the Desikan‐Killiany‐Tourville (DKT) atlas, a standard measure of cortical architecture.	128
	IDP T1 FIRST		Precise volumetric measures of **subcortical nuclei** (e.g., hippocampus, amygdala), structures linked to memory and emotional processing.	22
	volume		Volume of a specific brain region‐of‐interest (ROI), a fundamental metric of brain structure.	58
	IDP T1 SIENAX	Global Brain Measures	Estimated **total brain volume, grey matter volume, and white matter volume** (normalized for skull size), indicative of global atrophy or development.	10
	IDP T1fast	Tissue Composition	Voxel‐based estimates of **grey matter, white matter, and CSF probability/density**, used for voxel‐based morphometry (VBM) analysis.	139
Diffusion MRI (dMRI)	IDP dMRI TBSS	White Matter Microstructure	Skeleton‐based metrics (e.g., **Fractional Anisotropy (FA), Mean Diffusivity (MD)**) reflecting the integrity and organization of white matter tracts.	432
	IDP dMRI ProbtrackX	White Matter Macrostructure	Quantification of **structural connectivity strength** (e.g., streamline count) between brain regions using probabilistic tractography.	243
Susceptibility‐weighted MRI (SWI)	IDP SWI T2star	Tissue Composition / Metallomics	Sensitivity to **iron deposition and venous vasculature**, useful for assessing mineral metabolism or vascular abnormalities.	21
T2 FLAIR MRI	IDP T2 FLAIR	Lesion Load	Automated quantification of **white matter hyperintensity (WMH) volume**, a key marker of cerebral small vessel disease.	1
Task‐functional MRI (fMRI)	IDP tfMRI	Task‐Evoked Brain Activity	Activation magnitude (e.g., Beta values) in specific brain regions during a cognitive task (e.g., face recognition).	16

**FIGURE 1:**
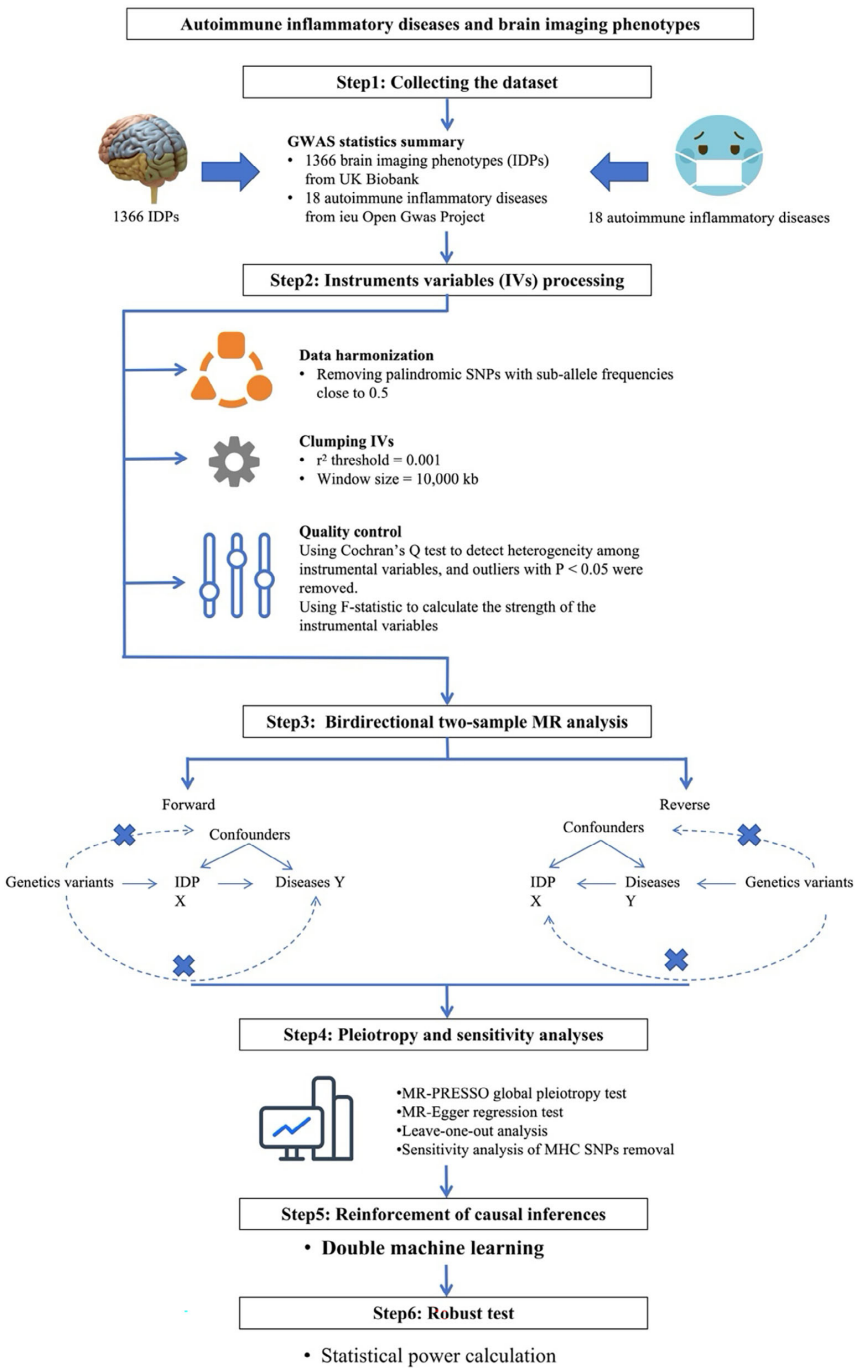
Workflow for bidirectional Mendelian randomization analysis between brain imaging‐derived phenotype and autoimmune inflammatory diseases. Workflow for bidirectional MR analysis between brain IDP and AIDs. Workflow for bidirectional two‐sample Mendelian randomization (MR) and double machine learning (DML) analyses between 1,366 brain imaging‐derived phenotypes (IDPs) and 18 autoimmune inflammatory diseases (AIDs). Single‐nucleotide polymorphisms (SNPs) significantly and independently associated with exposures (P < 5 × 10^−8^) were selected as instrumental variables (IVs), and those related to potential confounders were excluded. After quality control, MR analyses were conducted to infer causal relationships between IDPs and AIDs, followed by sensitivity analyses and statistical power estimation. DML was subsequently applied to revalidate the causal inferences. All analyses were based on summary‐level GWAS data (*n* = 8,428 UK Biobank participants for brain IDPs; AID sample sizes detailed in Table S1). This figure illustrates the analytical workflow only; no statistical tests were performed.

The study design is summarized in Figure 1. To fulfill the underlying assumptions of MR, we strictly controlled the quality of the instrumental variables (IV). We selected only SNPs significantly associated with exposure (*P* < 5 × 10^−8^) as IVs. To test the robustness of forward and reverse MR inference, we performed pleiotropy and sensitivity analyses and statistical power computations. These pieces of evidence suggest that our choice of IVs is appropriate and the causality of the forward and reverse MR estimates is reliable.

### 2.2 GWAS for AIDs

To gain insight into the causal relationship between brain structure and the pathogenesis of AIDs, this study used publicly available GWAS datasets of AIDs for MR analysis. To ensure the robustness of causal inferences, we did not include autoimmune inflammatory diseases with small sample sizes and no reported genome‐wide significant loci. Additionally, to minimize possible MR association bias due to differences in genetic background and other factors [12], we almost exclusively used the GWAS dataset for AIDs, which contains only samples of European ancestry. Based on these selection criteria, we performed MR analyses on GWAS datasets for 18 AIDs, including diseases such as SLE [13], dermatopolymyositis [13], Behcet's disease [13], Sjogren's syndrome [14], connective tissue disorder (CTD) [15], and MS [16]. Most GWAS summary statistics are downloaded directly from public databases, with preference given to datasets with more significant numbers of SNPs and sample sizes.

Table S1 details the 18 autoimmune inflammatory diseases used in the study and their ancestry information.

### 2.3 IVs Selection

Before conducting the MR analyses, we systematically screened the instrumental variables. First, we retained only SNPs that were significantly associated with the exposure (P < 5 × 10^−8^). Second, to ensure instrument independence, we applied linkage disequilibrium (LD) pruning using the clump function in the **TwoSampleMR** R package (v0.6.6) with r^2^ = 0.001 and a clumping window of 10 000 kb. These procedures were implemented to help satisfy the core assumptions of MR: (1) the selected IVs must be strongly correlated with the exposure, and (2) the IVs should influence the outcome solely through the exposure rather than through alternative pathways or horizontal pleiotropy [17].

### 2.4 Data Harmonization

In preparation for conducting the MR analysis, we carefully harmonized the exposure and outcome using the TwoSampleMR (v.0.6.6) R package. This process began with the standardization and matching of SNPs in the exposure and outcome data, with a focus on removing palindromic SNPs with sub‐allele frequencies close to 0.5 to avoid potential biases and errors [18]. The core of data harmonization was to ensure that instrumental variables were derived from the same direction of DNA stranding in both the exposure and outcome datasets to maintain consistency and accuracy in the analyses. In addition, this step ensures that each SNP has a consistent direction of effect in both datasets, which is key to safeguarding the validity of MR analyses. These comprehensive processing measures ultimately enhance the accuracy and confidence of MR analysis results.

### 2.5 Quality Control of Instrumental Variables

MR inferred causal relationships between exposures and outcomes by using genetic variants as IVs. To ensure the quality of IVs, we performed heterogeneity tests to deal with outliers. We performed Cochran's Q test on the ivw model using the R package RadialMR (v.1.0) to detect heterogeneity of IVs and remove outliers with *P* < 0.05 [19]. Moreover, we used the F‐statistic to assess the strength of the IVs; an F‐statistic > 10 indicates a low risk of weak instruments in the MR analysis, thus increasing the confidence and reliability of the results (Tables S2 and S3). The F‐statistic was calculated using the formula:

(1) $$F=\frac{R^{2}\left(N-k-1\right)}{k\left(1-R^{2}\right)}$$

where R^2^ represents the proportion of phenotypic variance explained by genetic variation, N represents the sample size, and k is the number of instruments. The calculation of R^2^ relies on β (the size of the genetic effect in the exposed GWAS data), standard error (s.e.), and sample size (N). The calculation formula is:

(2) $$R^{2}=\frac{\beta^{2}}{\beta^{2}+s.e.^{2}\times N}$$

### 2.6 Bidirectional Two‐Sample MR Analyses

We conducted a bidirectional two‐sample MR analysis to determine the causal link between AIDs and structural brain IDPs. We performed forward MR with IDPs as the exposure variable and AIDs as the outcome variable. Reverse MR analysis used AIDs as the exposure variable and set IDPs as the outcome variable. In this study, the inverse variance weighting (ivw) method was our primary causal inference method. Although the ivw method performs superiorly in terms of statistical validity, it may lead to biased results in the presence of pleiotropic effects. Therefore, we employed four additional MR methods to improve the reliability of our estimates. Of these, the MR‐Egger method assumes that the strength of the instrumental variable is independent of the direct effect and that the estimation of the causal impact relies on the slope coefficient of the Egger regression [20]. The Weighted median (W‐median) method bases its inference of causal effects on the median of the weighted empirical density function of the individual SNP effect estimates, allowing up to 50% of the genetic variance to exhibit horizontal pleiotropy [21].

To ensure the robustness of the MR results, we would expect the exposure to be associated with the outcome only if the estimates from the different methods in the forward and reverse MR analyses, respectively, satisfy the following requirements: at least 2 SNPs available for the IVs in bidirectional MR, *P* < 5.75 × 10^−4^ for the ivw method in forward MR (Bonferroni correction, 0.05/87, 87 is the number of IDPs for the positive outcome and the estimates are the same for all causal effects). *P* < 1.67 × 10^−2^ (Bonferroni‐corrected, 0.05/3, 3 being the number of AIDs with a positive outcome) for the ivw method in reverse MR. This conservative approach was chosen to prioritize the minimization of false positive results (Type I error) and ensure the robustness of causal claims, which we considered paramount for this study. Only then would we expect a causal relationship between the exposure and the outcome. Since IDPs are continuous variables and AIDs are binary variables, we use OR in positive MR to represent the effect size of the causal relationship between exposure and outcome, while we use β in reverse MR.

### 2.7 Sensitivity Analyses

We performed a series of sensitivity analyses to test the robustness of the MR estimates. First, we checked for the presence of horizontal pleiotropy using the MR‐PRESSO global test (*P* < 0.05), which excludes outliers where genetic variation may affect biological pathways other than exposure and outcome. We then ran the MR‐Egger regression test to assess directional pleiotropy, with *P* < 0.05 indicating the presence of directional pleiotropy, which helped us identify and correct for potential data bias [20]. Finally, we applied leave‐one‐out sensitivity analyses, in which we tested whether a specific SNP drove causality by removing one variant from the analysis and re‐estimating causality; this was done to prevent an SNP in particular from overly influencing the overall causal effect, further confirming the reliability of the study [22]. We used the data to create scatter plots, forest plots, funnel plots, and loo plots, which are shown in Figure S1, and through which the results of the MR analysis can be more fully assessed and understood. The results of the analysis are presented in Tables S4 and S5.

Additionally, to ensure our findings remain free from potential confounding biases, we screened IVs used in all significant results to identify variants located within the Major Histocompatibility Complex (MHC) region [23]. This region, characterized by intense linkage disequilibrium and extensive pleiotropy, represents a significant source of false positives. Following widely adopted standards in the field, we defined the MHC region as chr6: 28,477,797–33,448,354 (GRCh37/hg19 reference genome) [24]. For any significant results where the IVs included an SNP within this region, we conducted a dedicated sensitivity analysis: we removed that SNP, reran the MR analysis using the remaining IVs, and compared the effect estimates before and after removal to quantitatively assess the potential bias introduced by the MHC region.

### 2.8 Double Machine Learning

To complement our traditional MR analyses, we implemented a Double Machine Learning (DML) framework adapted for two‐sample summary‐level GWAS data. This approach uses the principles of orthogonality and cross‐fitting to provide a robust causal estimate [10]. We chose a selection of 16 AIDs for DML analysis of selected exposures in the traditional bidirectional two‐sample MR analysis, and the specific choices were the 5 exposures with the smallest p‐value for each outcome, which were positive under the ivw method in the results of the forward MR analysis (*p*<0.05). We use Python to perform DML on the data. The data for the 16 AIDs by DML as outcome were obtained from the ieu open GWAS project, GWAS Catalog, and FinnGen, and the specific data information is showed on Table 2.

**TABLE 2:** Source of data used for Double Machine learning analyses.

Autoimmune inflammatory diseases	Database	Sample sizes	Ancestry
Behcet's disease	GWAS Catalog	27 cases and 317,225 controls	EUR
Connective tissue disorder	GWAS Catalog	3,206 cases and 481,392 controls	NA
Dermatopolymyositis	FinnGen	174 cases and 484,260 controls	EUR
Diseases of the musculoskeletal system and connective tissue	FinnGen	5,732 cases and 494,616 controls	EUR
Drug‐induced systemic lupus erythematosus	FinnGen	234 cases and 500,114 controls	EUR
Fibromyalgia	FinnGen	3,632 cases and 357,549 controls	EUR
Fibromyalgia related co‐morbidities	ieu open GWAS project	2,305 cases and 358,889 controls	EUR
Giant cell arteritis	FinnGen	1,387 cases and 484,260 controls	EUR
Giant cell arteritis with polymyalgia rheumatica	FinnGen	1,225 cases and 484,260 controls	EUR
Multiple Sclerosis	ieu open GWAS project	47,429 cases and 68,374 controls	EUR
Polyarteritis nodosa	FinnGen	242 cases and 484,260 controls	EUR
Polymyositis	GWAS Catalog	44 cases and 350,228 controls	EUR
Sjogren's syndrome (Firth correction)	GWAS Catalog	407,746 individuals	EUR
Systemic connective tissue disorders	FinnGen	16,088 cases and 484,260 controls	EUR
Systemic lupus erythematosus	GWAS Catalog	647 cases and 482,264 controls	EUR
Systemic sclerosis	FinnGen	854 cases and 484,260 controls	EUR

Instead of individual‐level confounders, this summary‐data DML approach treats the genetic instruments (SNPs) as the high‐dimensional features. The goal is to estimate the causal effect θ in the partially linear model:

(3) $$\beta_{Y,i\;}=\theta \cdot \beta_{X,i}+h\left(Z_{i}\right)+\in_{i}$$

where β_*Y*,*i* _ and β_*X*,*i*_ are the effect sizes of SNP_i_ on the outcome (AID) and exposure (IDP), respectively. *Z_i_* represents the full set of SNPs (our high‐dimensional data), and *h(Z_i_)* is a nuisance function capturing confounding effects (i.e., horizontal pleiotropy) that *Z_i_* might have on the outcome that is *not* mediated by the exposure.

We implemented a K‐fold cross‐fitting procedure (where K = 5) to avoid overfitting:

1. The set of SNPs was randomly partitioned into K folds.
2. For each fold *k*, we used the other K‐1 folds to train two machine learning models (e.g., Lasso, Random Forest, or gradient boosting) to predict the nuisance components:
(4) $$\hat{m}\;\left(Z_{k}\right)=E\left[\beta_{X,i}|Z_{i}\in \mathrm{fold}\;k\right]$$
(5) $$\hat{r}\;\left(Z_{k}\right)=E\left[\beta_{Y,i}|Z_{i}\in \mathrm{fold}\;k\right]$$

Equation (4) is predicting exposure effects, and Equation (5) is predicting outcome effects

1. Using the ‘out‐of‐sample’ predictions for fold *k*, we constructed residuals:
(6) $${\tilde{\beta }}_{X}=\beta_{X,k}\;-\hat{m}\left(Z_{k}\right)$$
(7) $${\tilde{\beta }}_{Y}=\beta_{Y,k}\;-\hat{r}\left(Z_{k}\right)$$
2. The final causal estimateθis then computed as the coefficient from a simple linear regression on the residuals, aggregated across all *K* folds:
(8) $$\theta =\frac{E\left[{\tilde{\beta }}_{X}\cdot {\tilde{\beta }}_{Y}\right]}{E\left[{{\tilde{\beta }}^{2}}_{X}\right]}$$

This DML estimate is ‘Neyman‐orthogonal,’ meaning it is robust to small errors in the estimation of the nuisance models $\hat{m}$ and $\hat{r}$, providing a powerful validation of our primary ivw‐MR findings. The results are visualized in Figure 4 and Figure S3.

### 2.9 Statistical Power

MR analysis relies on genetic variation as an instrumental variable to estimate causal effects, and processing the results of MR by calculating statistical power methods can increase the credibility of the study. At the same time, higher statistical power can increase the credibility of the study and its value. We defined the level of significance (α = 0.05) to calculate the final efficacy value between 0 and 1. Statistical power was calculated by the formula:

(9) $$\mathrm{Power}=\Phi \left(\mathrm{OR}\times \sqrt{n\times VE}-z_{\alpha /2}-z_{\beta }\right)$$

where Φ is the standard normal cumulative distribution function, $\mathrm{OR}\times \sqrt{n\times VE}$ denotes the strength of the effect size, and *z*_α/2_ − *z*_β_ combines the significance level and the standard normal distribution threshold to be overcome for desired efficacy.

### 2.10 Reliability of Data

The GWAS data used in our research are publicly available and are all accessible on the ieu Open Gwas Project (https://gwas.mrcieu.ac.uk/). Some of the GWAS data are obtained from the GWAS Catalog (https://www.ebi.ac.uk/gwas/) and FinnGen (https://finngen.gitbook.io/documentation/) and used in DML. The brain region model maps we produced in our results were adapted from BodyParts3D (CC BY 4.0), with data from BodyParts3D, The Database Center for Life Science, licensed under CC Attribution 4.0 International (https://lifesciencedb.jp/bp3d/). All MR Analyses were performed in R, version 4.3.2 (https://www.r‐project.org), with the use of packages “MendelianRandomization”, “TwoSampleMR”, “RadialMR”, “data.table ”, “dplyr”, “tidyr”, “ggplot2”, “forestplot”, and “MR‐PRESSO”. The Double Machine Learning analyses were conducted using Python (v3.11.6) with the following key packages: doubleml.

### 2.11 Statistical Analysis

All statistical analyses were performed using R (version 4.3.2) and Python (version 3.11.6). Mendelian randomization (MR) analyses were implemented with the R packages MendelianRandomization (v0.7.0) and data.table (v1.15.4), dplyr(v1.1.4), tidyr (v1.3.1), ggplot2 (v3.5.1), forestplot (v3.1.0), TwoSampleMR (v0.6.6), RadialMR (v1.0), and MR‐PRESSO(v1.0), while double machine learning (DML) modeling was conducted in Python using doubleml (v0.10.1).

All MR and DML analyses were performed with fixed random seed values (set to 1234) to ensure full reproducibility.

#### 2.11.1 Pre‐Processing of Data

GWAS summary statistics for imaging‐derived phenotypes (IDPs) and autoimmune inflammatory diseases (AIDs) were harmonized using TwoSampleMR. Palindromic SNPs with intermediate allele frequencies were excluded, and linkage disequilibrium (LD) pruning was applied (r^2^ < 0.001, window = 10,000 kb). Outlier SNPs were removed using RadialMR Cochran's Q test (*P* < 0.05) and the MR‐PRESSO global test. All alleles were aligned to the same strand. For DML, variables were standardized (z‐score normalization) and split into five folds for cross‐fitted estimation to prevent overfitting. Potential pleiotropic effects of instrumental SNPs were further examined using the PhenoScanner R package (v1.0) and the PhenoScanner database (v2) to identify associations with potential confounders or other traits.

#### 2.11.2 Data Presentation

Results are expressed as odds ratios (ORs) or beta coefficients (β) with 95% confidence intervals (95% CI) and two‐sided P values. Multiple testing correction was performed using the Bonferroni method, with significance thresholds set at *P* < 0.05 / 87 for forward MR analyses and *P* < 0.05 / 3 for reverse MR analyses.

#### 2.11.3 Sample Size (n)

Sample sizes for GWAS datasets used in MR and DML analyses are detailed in Table S1 and Table 2, ranging from 27 to 47 429 cases and up to 500 000 controls for AIDs, and 8,428 participants for IDP GWAS.

#### 2.11.4 Statistical Tests and Significance Thresholds

Primary causal estimates were obtained using the inverse variance weighted (IVW) method, with complementary estimates from MR‐Egger regression, weighted median, and Wald ratio. Sensitivity analyses included leave‐one‐out, MR‐Egger intercept, and MR‐PRESSO global tests to assess pleiotropy. Heterogeneity across IVs was evaluated using Cochran's Q test. Statistical power was computed under a two‐sided α = 0.05 using the normal cumulative distribution function. In DML, causal effects were estimated via orthogonalized cross‐fitting using Lasso and Random Forest regressors. Statistical significance was defined as *P* < 0.05 after multiple‐testing correction.

#### 2.11.5 Post‐Hoc Statistical Power Calculations

To address concerns regarding Winner's Curse and unstable estimates in small‐case cohorts, we performed comprehensive post‐hoc power calculations for all 18 AIDs using the formula:

$$\mathrm{Power}=\Phi \left(\mathrm{OR}\times \sqrt{n\times VE}-z_{\alpha /2}-z_{\beta }\right)$$

Where Φ is the standard normal cumulative distribution function, n is the case sample size, VE is the variance explained by instruments (R^2^), and α = 0.05 (two‐sided). We defined power ≥80% as adequate for hypothesis‐confirming inferences. For the 11 AIDs with <1,000 cases, we critically evaluated whether statistically significant findings (p<Bonferroni threshold) achieved adequate power; underpowered significant findings were explicitly reclassified as exploratory. Complete power calculations are provided in Table 3 (positive outcomes), Table S8 (forward MR), and Table S9 (reverse MR).

**TABLE 3:** Calculations of the statistical power of forward MR positive outcomes.

Outcome	Exposure	pval	Variance_explained	Power_80_5
multiple sclerosis	IDP T1 FAST ROIs R ventral striatum	0.001632419	0.05	1
	IDP SWI T2star left caudate	0.000835472	0.05	1
	IDP SWI T2star right caudate	0.043444139	0.05	1
	IDP SWI T2star left caudate plus IDP SWI T2star right caudate	0.024529457	0.05	1
	volume Left‐Lateral‐Ventricle	0.038823378	0.05	1
	IDP T1 SIENAX CSF normalised volume	0.000477618	0.05	1
	IDP T1 SIENAX CSF unnormalised volume	0.028910193	0.05	1
	IDP dMRI ProbtrackX OD str l	0.000052124	0.05	1
Systemic lupus erythematosus	IDP T1 FAST ROIs L cerebellum crus I	0.031595945	0.05	1
	IDP SWI T2star left putamen	0.00930319	0.05	1
	IDP dMRI TBSS FA Cingulum cingulate gyrus R	0.045840348	0.05	0.999999469
	IDP dMRI TBSS MD External capsule L	0.002998632	0.05	1
	IDP dMRI TBSS MD Cingulum cingulate gyrus R	0.030694254	0.05	1
	IDP dMRI TBSS MD Cingulum cingulate gyrus L	0.029827597	0.05	1
	IDP dMRI TBSS MD Uncinate fasciculus L	0.001188223	0.05	1
	IDP dMRI TBSS L3 Uncinate fasciculus L	0.000317073	0.05	1
	IDP dMRI TBSS ICVF Anterior limb of internal capsule L	0.003908531	0.05	1
	IDP dMRI TBSS ICVF Posterior limb of internal capsule L	0.022282651	0.05	1
	IDP dMRI TBSS ICVF Retrolenticular part of internal capsule L	0.014759735	0.05	1
	IDP dMRI TBSS ICVF Anterior corona radiata R	0.026853835	0.05	1
	IDP dMRI TBSS ICVF Anterior corona radiata L	0.016304699	0.05	1
	IDP dMRI TBSS ICVF Superior corona radiata R	0.037966187	0.05	1
	IDP dMRI TBSS ICVF Superior corona radiata L	0.030347093	0.05	1
	IDP dMRI TBSS ICVF Posterior corona radiata L	0.048515778	0.05	1
	IDP dMRI TBSS ICVF External capsule R	0.002047548	0.05	1
	IDP dMRI TBSS ICVF External capsule L	0.000968098	0.05	1
	IDP dMRI TBSS ICVF Cingulum hippocampus R	0.013616175	0.05	1
	IDP dMRI TBSS ICVF Cingulum hippocampus L	0.007496049	0.05	1
	IDP dMRI TBSS ICVF Superior longitudinal fasciculus L	0.044847344	0.05	1
	IDP dMRI TBSS ICVF Superior fronto‐occipital fasciculus R	0.044275018	0.05	0.999999944
	IDP dMRI ProbtrackX MD cgc l	0.017061374	0.05	1
	IDP dMRI ProbtrackX L1 unc l	0.001493147	0.05	1
	IDP dMRI ProbtrackX L3 fmi	0.044434044	0.05	1
	IDP dMRI ProbtrackX ICVF ar l	0.003170112	0.05	1
	IDP dMRI ProbtrackX ICVF ar r	0.003514043	0.05	1
	IDP dMRI ProbtrackX ICVF atr l	0.005983629	0.05	1
	IDP dMRI ProbtrackX ICVF atr r	0.007723708	0.05	1
	IDP dMRI ProbtrackX ICVF cgh l	0.005200365	0.05	1
	IDP dMRI ProbtrackX ICVF cst l	0.00874505	0.05	1
	IDP dMRI ProbtrackX ICVF cst r	0.008900028	0.05	1
	IDP dMRI ProbtrackX ICVF slf l	0.026464718	0.05	1
	IDP dMRI ProbtrackX ICVF str l	0.020564195	0.05	1
	IDP dMRI ProbtrackX ICVF str r	0.025786989	0.05	1
	IDP dMRI ProbtrackX ICVF unc l	0.001331102	0.05	1
Vasculitis limited to skin, not elsewhere classified	IDP dMRI TBSS FA Superior cerebellar peduncle R	0.000030501	0.05	0.377592826
	IDP dMRI TBSS FA Superior cerebellar peduncle L	0.000028277	0.05	0.247716395
	volume Left‐Lateral‐Ventricle	0.034141659	0.05	1
	volume CSF	0.030565779	0.05	0.991344179
	IDP T1 SIENAX CSF normalised volume	0.004387794	0.05	1
	IDP T1 SIENAX CSF unnormalised volume	0.023660087	0.05	1
	IDP dMRI TBSS L2 Superior cerebellar peduncle R	0.000029687	0.05	1
	IDP dMRI TBSS L3 Superior cerebellar peduncle R	0.000028773	0.05	1
	IDP dMRI TBSS ICVF Posterior thalamic radiation L	0.023477006	0.05	1
	IDP dMRI TBSS ICVF Sagittal stratum L	0.015425095	0.05	1
	IDP dMRI ProbtrackX OD str l	0.01343919	0.05	0.642598984

*Note*: Power calculations assume α = 0.05 (two‐sided), variance explained = 5% (R^2^ = 0.05). Associations with power ≥80% (MS and SLE findings) are considered hypothesis‐confirming; associations with power <80% (vasculitis limited to skin) are considered exploratory and hypothesis‐generating only, requiring independent replication.

## 3 Results

### 3.1 Forward MR Analysis Results

**FIGURE 2:**
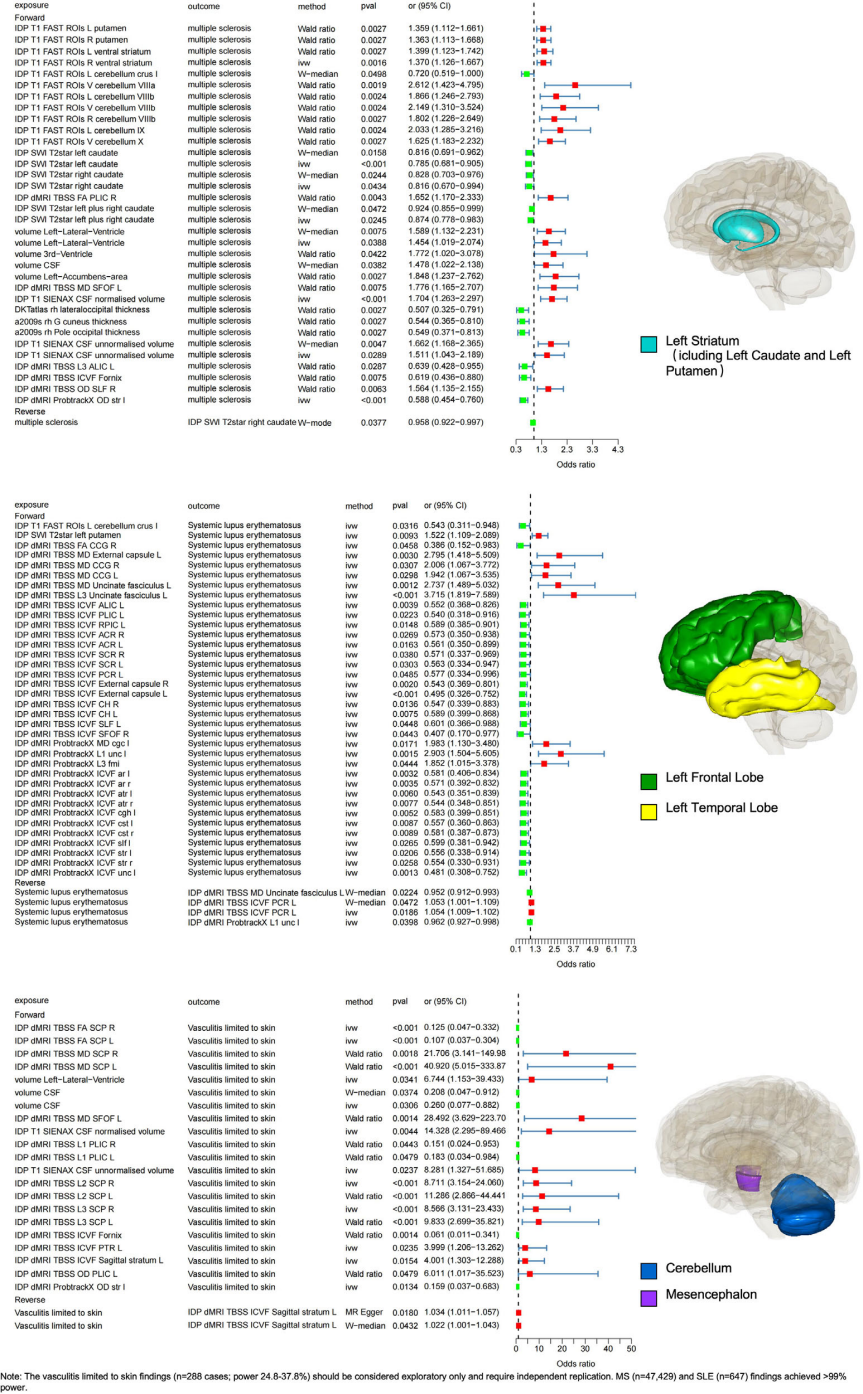
Visualized significant causal associations identified by forward Mendelian randomization. Forward MR is causal for positive outcomes. Visualized MR images show the significant causal effects identified by forward two‐sample MR analyses using four complementary methods (inverse‐variance weighted [IVW], weighted median, MR‐Egger, and Wald ratio). Data are presented as odds ratios (OR) with 95% confidence intervals (CI) for each IDP–AID pair. The colored 3D brain regions illustrate representative anatomical locations of the significant IDPs. Sample sizes: n = 1,366 derived from GWAS of 8,428 UK Biobank participants for brain IDPs (exposure GWAS) and disease‐specific sample sizes for AIDs (see Table S1). Statistical significance was evaluated by two‐sided tests with Bonferroni‐corrected thresholds (*P* < 5.747 × 10^−4^). No significance symbols are displayed in the figure; all P values are two‐tailed.

To explore the causal relationship between brain structure and its neural activity and functional connectivity to AIDs, we first performed a forward MR analysis. We identified seven IDPs that may have a causal effect on three AIDs (Bonferroni‐corrected *P*  <  5.75 × 10^−4^ (0.05/87, with 0.05 being the significance threshold for a single test, and 87 being the number of IDP phenotypes that had a forward MR and a positive result under the number of IDPs with a positive outcome under the ivw approach)), including SLE, vasculitis limited to the skin, and MS (Figure 2; Table S6). Finally, we screened the IVs (SNPs) used for these seven positive causal relationships and did not identify any SNPs within the MHC region. This indicates that MHC SNPs did not influence the positive results of the forward MR.

### 3.2 Influence of Brain Structure on MS

According to our study, left striatum and cerebrospinal fluid volume were associated with the risk of developing MS. A 1 standard deviation increase in amplitude characteristics of the left striatal region was associated with a 41% reduction in the risk of MS (ivw OR = 0.59, 95% CI: 0.45‐0.76, *P*  =  5.21 × 10^−5^), whereas a one standard deviation increase in amplitude characteristics of the cerebrospinal fluid volume was associated with a 70% increase in the risk of MS (ivw OR = 1.70, 95% CI: 1.26‐2.30, *P*  =  4.78 × 10^−4^).

### 3.3 Influence of Brain Structure on SLE

The Uncinate Fasciculus (UF) is a bundle of white matter fibers in the brain that primarily connects structures such as the amygdala and hippocampus in the limbic system to the orbitofrontal and anterior polar cortices in the frontal lobe. In contrast, the left UF plays an essential role in the modulation of affective responses, memory processing, and language (e.g., semantic processing) [25, 26]. As shown in Figure 2, a one standard deviation increase in the left UF amplitude profile was associated with a 271% increase in the risk of SLE (ivw OR = 3.72, 95% CI: 1.82‐7.59, *P*  =  3.17 × 10^−4^).

### 3.4 Influence of Brain Structure on Vasculitis Limited to the Skin

Vasculitis limited to the skin, confined to the skin, is an inflammatory disease of the small blood vessels that primarily affects the skin and usually manifests as skin lesions such as erythema, purpura, nodules, and ulcers [27]. We found that the left superior cerebral peduncle and the right superior cerebral peduncle were closely associated with vasculitis limited to the skin (Figure 2). An increase of 1 standard deviation in the amplitude characterization of the left superior cerebellar peduncle region was associated with an 89% reduction in the risk of vasculitis limited to skin (ivw OR = 0.11, 95% (CI): 0.04‐0.30, *P*  =  2.83 × 10^−5^). As shown in the results, the effect of the three positive right superior cerebellar peduncle characterizations on the risk of vasculitis limited to the skin was variable. We chose the result with the smallest p‐value to ensure accuracy. The increase of 1 standard deviation in the amplitude characterization of the right superior cerebellar peduncle region was associated with a 757% increase in the risk of vasculitis limited to skin (ivw OR = 8.57, 95% CI: 3.13‐23.4, *P*  =  2.88 × 10^−5^). The complete visualization of the results of the forward MR is shown in Figure S2.

### 3.5 Brain Structure does not Affect 15 Other AIDs

We similarly analyzed positive MR for 15 other AIDs, including Sjogren's syndrome, dermatopolymyositis, and Behcet's disease. Because the analysis involved multiple causality assessments, we applied a Bonferroni correction. After correction, the results were statistically significant only if the p‐value of the ivw method was *P*  <  5.75 × 10^−4^. However, the P‐value of the ivw method was greater than 5.75 × 10^−4^ for all 15 diseases analyzed. Hence, the results of the study were not statistically significant, indicating that IDPs did not have a significant effect on the other 15 AIDs.

### 3.6 Reverse MR Analysis Results

The risk of AIDs may also affect neural activity and functional network connectivity in relevant brain structure regions. To explore the causal effects of AIDs on brain structures, we performed a reverse MR analysis between AIDs and IDPs. In this study, we identified potential causal associations between 2 AIDs and 2 IDPs. The risk of AIDs mainly affects different brain regions, including the cerebellar area VIIIa and the left cerebellar cortex. (Bonferroni‐corrected *P*  <  1.67 × 10^−2^ (0.05/3, 0.05 is the significance threshold for a single test, and 3 is the number of disorders with reverse MR and a positive outcome under the ivw method)).

### 3.7 Effects of AIDs on Brain Structure

**FIGURE 3:**
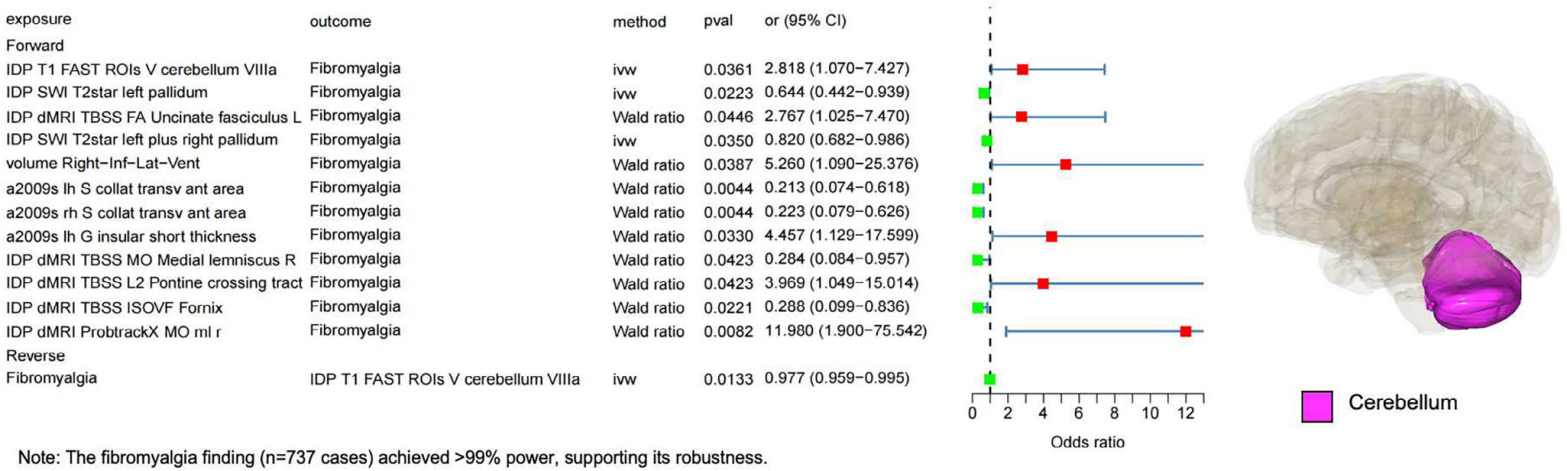
Visualized significant causal associations identified by reverse Mendelian randomization. Reverse MR is causal for positive outcomes. Visualized MR images depict the causal influence of AIDs on neural activity or structure in relevant brain regions. Each plot shows effect estimates (β with 95% CIs) derived from inverse‐variance weighted (IVW), weighted median, MR‐Egger, and Wald ratio analyses. Colored brain maps highlight the corresponding IDP locations. Sample sizes: n = 1,366 derived from GWAS of 8,428 UK Biobank participants for brain IDPs (outcome GWAS) and disease‐specific sample sizes for AIDs (Table S1). Statistical significance was assessed by two‐sided tests with Bonferroni correction (*P*  <  1.67  ×  10^−2^). No significance symbols are shown in the figure; all P values are two‐tailed.

Fibromyalgia and Fibromyalgia‐related co‐morbidities affect neural activity in areas associated with brain structures. Analysis of reverse MR showed that a higher risk of fibromyalgia was negatively correlated with neural activity in cerebellar area VIIIa (ivw β = −0.023, 95% CI: −0.042 to −0.0049, *P*  =  1.33  ×  10^−2^). Similarly, the risk of fibromyalgia−related comorbidities showed a strong negative correlation with neural activity in the left cerebellar cortex (ivw β = −6.50, 95% CI: −11.68 to −1.31, *P*  =  1.41  ×  10^−2^). Finally, we screened the IVs used for the causal relationship between these two positives and found that in the reverse MR analysis with “Fibromyalgia‐related comorbidities as exposure and volume Left‐Cerebellum‐Cortex as outcome,” one SNP—rs16895223 (chr6:29626949)—was located within our defined MHC region. After removing this SNP and rerunning the reverse MR analysis, our sensitivity analysis revealed that the IVW method's p‐value changed from 0.0141 to 0.0369, exceeding our required positive p‐value threshold. This indicates that the MHC SNP caused a false positive result. Consequently, we excluded this finding from our positive results (Figure 3; Table S7). The complete visualization of the results of the reverse MR is shown in Figure S2.

### 3.8 DML Results

**FIGURE 4:**
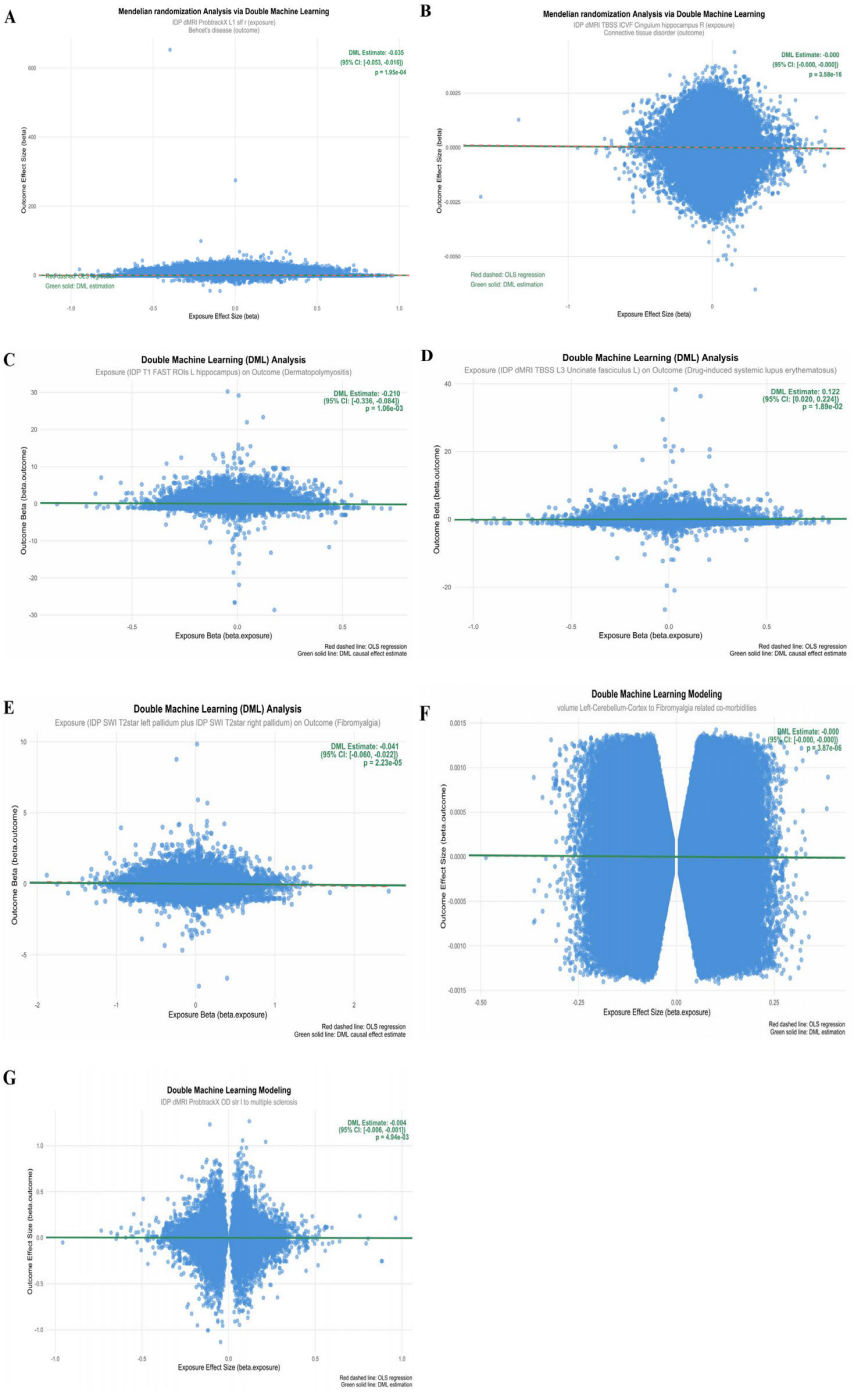
Double Machine Learning (DML) analysis used to validate Mendelian randomization findings. Results of Double machine learning. Scatter plots show the causal effect estimates derived from DML analyses for seven AID–IDP pairs (A–G), each consistent in direction with the corresponding bidirectional two‐sample MR results. Each point represents one SNP instrument; the green solid line shows the DML‐estimated causal slope (Estimate), and the red dashed line shows the ordinary least squares trend. Data are presented as estimated β values. DML analyses were performed using 5‐fold cross‐fitting with Lasso or Random Forest models to control for high‐dimensional confounders. Sample sizes for each AID dataset are detailed in Table 2. (A) IDP dMRI ProbtrackX L1 slf right → Behcet's disease. (B) IDP dMRI TBSS ICVF Cingulum hippocampus right → Connective tissue disorder. (C) IDP T1 FAST ROIs left hippocampus → Dermatopolymyositis. (D) IDP dMRI TBSS L3 Uncinate fasciculus left → Drug‐induced systemic lupus erythematosus. (E) IDP SWI T2star left + right pallidum → Fibromyalgia. (F) Volume Left‐Cerebellum‐Cortex → Fibromyalgia‐related comorbidities. (G) IDP dMRI ProbtrackX OD str left → Multiple sclerosis. All P values are two‐sided; statistical significance was defined as P < 0.05. No significance symbols are shown in the figure.

We modeled causal inference by DML for 16 AIDs as outcomes and their five exposures with the smallest p‐values under the ivw method in the forward MR analysis results, and the analysis showed that seven AIDs had a statistically significant effect with their exposures (*p* < 0.05), and with the direction of the effect in full agreement with the MR analysis results. Whereas some AIDs had a statistically significant effect with their exposure, but the direction of the effect was not consistent with the forward MR analysis, only a few AIDs had no statistically significant effect. All the positive and statistically strongest DML result plots (Estimate vs. bidirectional two‐sample MR analysis effect value β direction, *p*<0.05) among the 7 AIDs that were positive and consistent with the positive MR direction are shown in Figure 4. The remaining results from the DML analyses are shown in Figure S3. Figure 4A–G shows the results of these seven pairs of correlations of exposure‐outcome causality. Each scatter in the subgraph A‐G represents a genetic tool (SNP), the horizontal axis represents the exposure effect (beta.exposure), the vertical axis represents the outcome effect (beta.outcome), the green solid line intercept is fixed at 0, the slope is the estimated effect value from the DML analysis (i.e., Estimate), and the red dashed line is the trend of all scatters under traditional least squares between the exposure and the the trend of the fit between endings. From this we see that the green solid line and red dashed line trends in subgraph A–G are almost identical or even the red line is covered, indicating that the traditional linear regression estimates are highly consistent with more advanced DML methods for the given data, for example, Figure 4G demonstrates that the amplitude characteristics of the left striatal region is significantly and negatively associated with the risk of MS (Estimate values are in the negative direction with a statistically significant effect of p‐value much less than 0.05). The causal effect estimated by the DML in the figure is in the same direction as the traditional MR analysis (Table S6), indicating that the effect of exposure on outcome changed very little after controlling for high‐dimensional confounders, and that the analysis of the DML and the traditional MR methods yielded highly concordant results, supporting the reliability of the causal effect and mutual validation.

### 3.9 Calculations of Statistical Power

As shown in Table 3, the explained variance of the positive outcomes (i.e., Bonferroni‐corrected) associated with our results was both 0.05, meaning that the exposure explained 5% of the variance of the target trait, i.e., the SNP or genetic variant predicted change in the trait or disease at 5%. And at 5% error rate, the efficacy is overwhelmingly close to 100%. These results illustrate the robustness and reliability of the MR results and increase the credibility and scientific value of the study. We calculated the statistical power of MR for all diseases, and complete tables are provided in Tables S8 and S9.

## 4 Discussion

In this study, we performed a bidirectional two‐sample MR analysis to investigate the causal relationship between 1366 brain structure IDPs and 18 AIDs. Our results explain the causal relationship between brain structures and AIDs. Our study not only reveals the causal relationship between brain structures and their neural activity and functional connectivity to AIDs but also suggests that the risk of AIDs may affect brain structure and function. Possibly for the first time, we demonstrated a causal relationship between the neural activity of brain structures and AIDs and analyzed the genetic mechanisms of different AIDs, a finding that has allowed us to propose new non‐invasive therapeutic strategies for autoimmune inflammatory disorders manifesting in neurological symptoms. That is, the development of targeted neuroprotective drugs (e.g., antioxidants or nerve growth factors) as well as neurorestorative therapies (e.g., stem cell therapy) for the destruction of brain tissues by AIDs could help to repair damaged neural tissues and may have therapeutic potential for the neurological symptoms of AIDs.

Our MR identified robust causal relationships between IDPs and AID. To explore potential mechanistic pathways, we can situate our findings within the context of large‐scale brain networks (e.g., DMN, SN, CEN). However, it must be emphasized that these associations are hypothetical and inferred from existing literature on the general functions of these neuroanatomical structures. Our study design, based on pooled GWAS data for individual IDPs, cannot provide direct evidence of functional connectivity at the network level. Therefore, the following discussion regarding network involvement should be understood as a speculative framework aimed at constructing plausible biological narratives and future research hypotheses, rather than established conclusions.

Brain structures correspond to three large networks (DMN, SN, and CEN), and the current study reveals that the DMN, the default mode network in SLE patients, may be affected. In our forward MR analysis, we found that neural activity in the left uncinate fasciculus was associated with an increased risk of developing SLE. The left uncinate fasciculus can influence DMN function by connecting DMN‐related brain regions (medial prefrontal cortex and medial temporal lobe) to brain regions associated with autobiographical memory and emotional processing, which have been implicated in DMN activity patterns [28, 29]. SLE is one of the multi‐system AIDs known to affect the central nervous system and may trigger neuropsychiatric symptoms, and patients with SLE often present with cognitive dysfunction and mood disorders [30]. Based on our genetic findings, we hypothesize that cognitive deficits, mood disorders, and other neuropsychiatric symptoms in SLE patients may be associated with abnormal functioning of the DMN and alterations in white matter fiber bundles (e.g., uncinate fasciculus). We further speculate that alterations in the left uncinate fasciculus may affect prefrontal‐temporal connectivity, which may interfere with the normal functioning of the DMN, triggering a range of symptoms. Taken together, this speculative framework posits that an affected DMN, potentially influenced by genetic liability underlying white matter integrity, may play an important role in SLE pathogenesis.

In our analysis of vasculitis limited to skin, we observed a strong, asymmetric, and opposing causal effect from the superior cerebellar peduncles (Left: OR = 0.11; Right: OR = 8.57). This finding is highly novel and lacks a clear biological rationale, as this AID rarely presents with neurologic involvement [31]. Given that these estimates were derived from a small number of IVs (*n* = 2) and a small case sample (*n* = 288), this result must be interpreted with extreme caution as it may represent a false positive. Given that these estimates were derived from a small number of IVs (*n* = 4), a small case sample (*n* = 288 cases), and post‐hoc power calculations showing only 24.8%–37.8% power for these associations—well below the conventional 80% threshold—this result must be interpreted with extreme caution as it may represent a false positive driven by Winner's Curse. We report it here as an exploratory, hypothesis‐generating finding that warrants independent replication before any biological conclusions can be drawn.

The results of forward MR analysis of MS showed that reduced neural activity in the left striatum and altered cerebrospinal fluid volume can contribute to the development of MS. The striatum is also capable of affecting CEN connectivity. The striatum (especially the left caudate nucleus) is functionally connected to key areas of the CEN, including the dorsolateral prefrontal cortex and the posterior parietal cortex. This connectivity supports the integration and modulation of cognitive control functions, whereas changes in the striatal connections to the CEN can affect these cognitive processes [32]. It has also been shown that MS affects both white and gray matter and that microstructural changes in the striatum can lead to motor and cognitive dysfunction in patients with MS [33]. Collectively, these observations lead us to propose a hypothesis: damage to the left striatal region, as indicated by our MR results, might contribute to the cognitive deficits common in people with MS through a potential disruption of CEN connectivity. Our review of relevant data revealed studies showing that biomarkers in cerebrospinal fluid, such as oligoclonal bands and free light chains, are essential in the diagnosis and monitoring of the course of MS, and that these markers may reflect inflammatory activity within the CNS and correlate with the pathogenesis of MS [34].

We did not find positive results of forward MR for AIDs with corresponding positive effects of reverse MR. Interestingly; we found in reverse MR that fibromyalgia inhibits cerebellar area VIIIa. Similarly, the cerebellum is strongly associated with both CEN and motor control networks. To interpret this reverse MR finding (where the disease affects the brain), we speculate on a potential mechanism. It is plausible that the cerebellum's involvement in CEN areas through cortico‐cerebellar‐cortical loops with prefrontal cortex connections is critical for cognitive control and decision‐making processes. In contrast, connections between the left cerebellar cortex and motor areas of the brain (e.g., the primary motor cortex) are critical for fine motor control and coordination. Area VIIIa of the cerebellum is also involved in motor control, coordinating movement through connections with the cerebral motor cortex [35, 36]. Clear studies show that people with fibromyalgia have morphological and connectivity changes in brain structure that involve multiple regions related to pain, emotional processing, and movement, including the cerebellum [37].

Our MR analyses provide genetic evidence for a bidirectional causal relationship between the brain and AIDs. From a mechanistic perspective, these associations can be further elucidated through the brain‐immune axis [38, 39]. Our forward MR findings could be interpreted as identifying the genetic determinants of this axis at the level of brain structure/function. For instance, our key finding—that reduced neural activity in the left striatum significantly increases the risk of MS—can be mechanistically unpacked. The striatum is a key hub in the cortico‐striato‐thalamo‐cortical circuits, which are critically involved in regulating the autonomic nervous system [40]. A genetically predisposed reduction in striatal activity could lead to a dysregulated sympathetic output. This, in turn, could disrupt the neural innervation of lymphoid organs (e.g., the spleen), altering the release of neurotransmitters like norepinephrine and shifting the immune milieu toward a pro‐inflammatory state [41, 42]. This creates a permissive environment for the breakdown of self‐tolerance, a hallmark of MS pathogenesis. Similarly, our finding—that altered neural activity in the left uncinate fasciculus exerts a causal effect on SLE risk—can be mechanistically interpreted. The uncinate fasciculus, a major limbic–frontal white matter tract, connects the amygdala and hippocampus with the orbitofrontal cortex, forming a key structural substrate for emotional regulation and stress processing [25, 26]. This pathway is intimately involved in modulating the hypothalamic–pituitary–adrenal (HPA) axis and affective responses [43]. Disruption of uncinate fasciculus integrity could therefore impair top‐down control of stress and glucocorticoid signaling, leading to maladaptive activation of the HPA axis. Dysregulated cortisol and corticotropin‐releasing hormone dynamics, in turn, influence immune cell differentiation and cytokine production, promoting a pro‐inflammatory milieu characteristic of SLE. Thus, compromised uncinate fasciculus function may serve as a neuroanatomical link between stress‐related neuroendocrine dysregulation and systemic autoimmunity.

In stark contrast, we interpreted the reverse causal inferences as the top‐down consequences of the chronic disease state. Our finding that fibromyalgia suppresses neural activity in cerebellar area VIIIa. The cerebellum is increasingly recognized for its role in pain processing and modulation [44]. The persistent, widespread pain that defines fibromyalgia is not a passive symptom but a driver of central sensitization and neuroplastic changes [45]. Chronic nociceptive input can lead to functional alterations in the cerebellar circuits involved in sensory integration and motor control, resulting in the observed reduction in activity. This is further exacerbated by the chronic stress, sleep disturbances, and potential neuroinflammatory components of the disease, which collectively contribute to remodeling brain function and structure over time. Therefore, this framework clarifies the distinct nature of our bidirectional results: the forward inferences point to the etiological roles of specific neural phenotypes in setting the stage for immune dysregulation, while the reverse inferences likely reflect the enduring impact of a dysregulated immune system and its symptomatology on the central nervous system.

After performing bidirectional two‐sample MR analysis, we conducted sensitivity analysis to screen and exclude MHC SNPs associated with positive MR results, ensuring that the analysis was unaffected by MHC SNPs. We introduced DML analysis. Its advantages include the ability to enhance the handling of high‐dimensional confounding variables by traditional MR methods, to be more robust to model‐setting errors, and also to accommodate a variety of machine‐learning methods and nonlinear relationships. Thus, a more comprehensive assessment of causality can be achieved by combining DML with Bidirectional two‐sample MR analyses. In particular, DML's causal inference modeling of 16 AIDs as outcomes and their five exposures with the smallest p‐values under the ivw approach in the forward MR analysis results had statistically significant effects with their exposures in seven AIDs and was consistent with the causal effects of the traditional MR analyses, and the results were not statistically significant for only a few AIDs, which led to the mutual validation of the DML and the traditional MR results, further reinforcing our traditional MR analyses. Further reinforcing that our traditional MR analysis results are highly reliable. However, DML analysis combines machine learning methods and semiparametric statistical theory, which makes the data processing of DML analysis quite time‐consuming and labor‐intensive, which suggests that we can conduct further research by optimizing the DML parameters or expanding the sample size.

Finally, the introduction of statistical power calculations provided a quantitative reliability assessment for our research. By setting a significance level (α = 0.05) and calculating the efficacy value, we gained a clear understanding of the ability to test hypotheses under the given sample conditions. The final demonstration of extremely high efficacy values suggests that our study design has sufficient statistical validity to detect potential causal relationships. This statistical rigor provides additional confidence in MR, making our inferences more scientifically valuable. The robustness of the results reflects the soundness of the study design and further supports our findings regarding the relationship between IDPs and AIDs.

This study still has several limitations. First, the IDP data source is restricted. We used the earlier UKB imaging GWAS (n≈8,428), which has known sampling limitations. A much larger dataset, BIG40 (∼40 000 participants), is now available, but because it fully contains the original sample, it cannot serve as an independent replication cohort. Nevertheless, future studies should re‐run the analyses using BIG40 to improve power and robustness. Second, all GWAS datasets were from individuals of European ancestry. Although this reduces population stratification, the findings may not generalize to other ancestries. Third, MRI‐derived IDPs inevitably suffer from measurement variability due to scanner differences, acquisition parameters, and processing pipelines, which may introduce noise into causal estimates. Fourth, statistical power was limited for many AIDs. Eleven of the eighteen AIDs had fewer than 1,000 cases, increasing the risk of Type II errors and Winner's Curse (inflated effect estimates when findings do reach significance). For analyses with adequate instruments, F‐statistics remained well above 10, reducing concerns regarding weak instruments. We conducted a comprehensive post‐hoc power calculation (Table 3, Tables S8 and S9), which shows that the three AIDs with statistically significant results after Bonferroni correction (MS *n* = 47,429; SLE *n* = 647; Fibromyalgia *n* = 3,632) all demonstrated >99% power, strongly supporting the robustness and reliability of these hypothesis‐confirming findings. In stark contrast, vasculitis limited to skin (*n* = 288) showed only 24.8%–37.8% power for the reported superior cerebellar peduncle associations, despite passing our stringent Bonferroni threshold (*p* < 5.75×10^−4^). This finding is particularly concerning given the small number of instruments (*n* = 14 IVs) and the lack of biological plausibility for neurologic involvement in this cutaneous disease. The 15 AIDs showing no significant associations after Bonferroni correction (including 8 small‐case cohorts) represent true negative findings where the power concern is less critical, as the absence of statistical significance already indicates insufficient evidence for causation. Given these power calculations, we explicitly reclassify the vasculitis limited to skin findings as exploratory and hypothesis‐generating only. Fifth, the conservative Bonferroni correction [46] minimized false positives but increased the likelihood of false negatives. Alternative approaches, such as FDR, may be more suitable for hypothesis‐generating studies [47]. Sixth, we applied univariable MR across 1,366 correlated IDPs, which may violate the exclusivity assumption. Although MR‐PRESSO, MR‐Egger, and DML provide safeguards, they do not replace multivariable MR. Future work should use MVMR to better separate the effects of correlated brain traits. Despite these limitations, this study is, to our knowledge, the first to systematically examine causal relationships between IDPs and AIDs using combined MR and DML approaches, offering new neuro‐immunological insights into AID mechanisms.

Overall, we identified eight causal relationships between 1366 IDPs and 18 AIDs (Figure S4). Our study reveals the genetic mechanisms of different AIDs from the perspective of brain tissue genetics. In addition, our study points out brain regions that are important for the development of new therapies.

## Author Contributions

J.C. performed the analyses and wrote the first draft of the manuscript. X.W., H.D., and H.Y. performed the literature search, discussed the results, and edited the manuscript. B.Z., K.Z., C.H., and J.L. assisted with the statistical analysis. X.Z. checked statistical accuracy as an expert in statistics and supervised the study with H.Y. All authors read and approved the final manuscript.

## Funding

This work was supported by the State Key Laboratory of Pathogenesis, Prevention, Treatment of Central Asian High Incidence Diseases Fund (SKL‐HIDCA‐2024‐GD7B); the 2024 Guangdong Basic and Applied Basic Research Foundation City‐University Joint Fund Project (2024A1515140126); Clinical & Basic Science Technology Innovation Project of Guangdong Medical University (GDMULCJC2024103, GDMULCJC2025167); Guangdong Medical University Undergraduate Innovation and Entrepreneurship Education Base Project (JDXM2024069F); and the 2022 Guangdong Basic and Applied Basic Research Foundation Natural Science Fund Project (2022A1515012407). Dongguan Science and Technology of Social Development Program (20221800905642); Guangdong Medical University University‐level Undergraduate Training Program for Innovation and Entrepreneurship Project (202510571030).

[Correction added on 6 May 2026 after first online publication: Funding section has been updated in this version.]

## Ethics Approval and Consent to Participate

The study aligns with the principles of medical research ethics as outlined in the Helsinki Declaration. No ethics approval or written consent was needed for the secondary analysis of public data. Given that patient data was extracted from public databases, informed consent forms were not deemed necessary for this study.

## Conflicts of Interest

The authors declare no conflicts of interest.

## Supporting information




**Supporting file**: advs73573‐sup‐0001‐SuppMat.pdf

## Data Availability

GWAS summary data came from the IEU Open GWAS Project (https://gwas.mrcieu.ac.uk/), as detailed in Table S1. Some of the GWAS data are obtained from the GWAS Catalog (https://www.ebi.ac.uk/gwas/) and FinnGen (https://finngen.gitbook.io/documentation/) and used in DML. Brain region model map data sourced from BodyParts3D (https://lifesciencedb.jp/bp3d/). All data generated or analyzed during this study are included in this published article. Data sharing is not applicable to this article as no new data were created or analyzed in this study. The source code and packages used for Mendelian randomization and double machine learning are publicly available: TwoSampleMR (https://github.com/MRCIEU/TwoSampleMR) and MendelianRandomization (https://github.com/cran/MendelianRandomization) for MR analyses; DoubleML for Python (https://github.com/DoubleML/doubleml‐for‐py) for DML implementation.
