# Supplementary material for: Mendelian Randomization and Double Machine Learning Modeling Reveal Brain Imaging‐Derived Phenotypes as Functional Contributors to 18 Autoimmune Inflammatory Diseases
Source: Adv Sci (Weinh). 2025 Dec 25;13(14):e15675. doi: 10.1002/advs.202515675 (PMC12970252; doi:10.1002/advs.202515675)
Supplement: Supplementary file 1 — Supporting file: advs73573‐sup‐0001‐SuppMat.pdf [file ADVS-13-e15675-s001.pdf]

Mendelian randomization and double machine learning modeling reveal brain imaging-derived phenotypes as functional contributors to 18 autoimmune inflammatory diseases

Abbreviations 2

Figure S1 7

Figure S2 15

Figure S3 24

Figure S4 28

Table S1 30

Table S2 31

Table S3 32

Table S4 34

Table S5 35

Table S6 36

Table S7 37

Table S8 38

Table S9 610

Table S10 632

Abbreviations

AID: Autoimmune inflammatory disease; CI: confidence interval; CTD: Connective tissue disorder; DML: Double Machine Learning; Drug-induced SLE: Drug-induced systemic lupus erythematosus; FDR: False Discovery Rate; GCA: Giant cell arteritis; GCA-PMR: Giant cell arteritis with polymyalgia rheumatica; GWAS: Genome-wide association Studies; LOO: leave-one-out; IDP dMRI TBSS FA CCG R: IDP dMRI TBSS FA Cingulum cingulate gyrus R; IDP dMRI TBSS FA PCR L: IDP dMRI TBSS FA Posterior corona radiata L; IDP dMRI TBSS FA PLIC R: IDP dMRI TBSS FA Posterior limb of internal capsule R; IDP dMRI TBSS FA PLIC R: IDP dMRI TBSS FA Posterior limb of internal capsule R; IDP dMRI TBSS FA RPIC L: IDP dMRI TBSS FA Retrolenticular part of internal capsule L; IDP dMRI TBSS FA SCC: IDP dMRI TBSS FA Splenium of corpus callosum; IDP dMRI TBSS FA SCC: IDP dMRI TBSS FA Splenium of corpus callosum; IDP dMRI TBSS FA SCP L: IDP dMRI TBSS FA Superior cerebellar peduncle L; IDP dMRI TBSS FA SCP L: IDP dMRI TBSS FA Superior cerebellar peduncle L; IDP dMRI TBSS FA SCP R: IDP dMRI TBSS FA Superior cerebellar peduncle R; IDP dMRI TBSS FA SCP R: IDP dMRI TBSS FA Superior cerebellar peduncle R; IDP dMRI TBSS FA SLF L: IDP dMRI TBSS FA Superior longitudinal fasciculus L; IDP dMRI TBSS ICVF ACR L: IDP dMRI TBSS ICVF Anterior corona radiata L; IDP dMRI TBSS ICVF ACR R: IDP dMRI TBSS ICVF Anterior corona radiata R; IDP dMRI TBSS ICVF ALIC L: IDP dMRI TBSS ICVF Anterior limb of internal capsule L; IDP dMRI TBSS ICVF BCC: IDP dMRI TBSS ICVF Body of corpus callosum; IDP dMRI TBSS ICVF BCC: IDP dMRI TBSS

ICVF Body of corpus callosum; IDP dMRI TBSS ICVF CCG L: IDP dMRI TBSS ICVF Cingulum cingulate gyrus L; IDP dMRI TBSS ICVF CH L: IDP dMRI TBSS ICVF Cingulum hippocampus L; IDP dMRI TBSS ICVF CH R: IDP dMRI TBSS ICVF Cingulum hippocampus R; IDP dMRI TBSS ICVF FC+ST L: IDP dMRI TBSS ICVF Fornix cres+Stria terminalis L; IDP dMRI TBSS ICVF PCR L: IDP dMRI TBSS ICVF Posterior corona radiata L; IDP dMRI TBSS ICVF PLIC L: IDP dMRI TBSS ICVF Posterior limb of internal capsule L; IDP dMRI TBSS ICVF PLIC R: IDP dMRI TBSS ICVF Posterior limb of internal capsule R; IDP dMRI TBSS ICVF PTR L: IDP dMRI TBSS ICVF Posterior thalamic radiation L; IDP dMRI TBSS ICVF RLIC L: IDP dMRI TBSS ICVF Retrolenticular part of internal capsule L; IDP dMRI TBSS ICVF RLIC R: IDP dMRI TBSS ICVF Retrolenticular part of internal capsule R; IDP dMRI TBSS ICVF RPIC L: IDP dMRI TBSS ICVF Retrolenticular part of internal capsule L; IDP dMRI TBSS ICVF SCC: IDP dMRI TBSS ICVF Splenium of corpus callosum; IDP dMRI TBSS ICVF SCC: IDP dMRI TBSS ICVF Splenium of corpus callosum; IDP dMRI TBSS ICVF SCR L: IDP dMRI TBSS ICVF Superior corona radiata L; IDP dMRI TBSS ICVF SCR R: IDP dMRI TBSS ICVF Superior corona radiata R; IDP dMRI TBSS ICVF SFOF L: IDP dMRI TBSS ICVF Superior fronto-occipital fasciculus L; IDP dMRI TBSS ICVF SFOF R: IDP dMRI TBSS ICVF Superior fronto-occipital fasciculus R; IDP dMRI TBSS ICVF SLF L: IDP dMRI TBSS ICVF Superior longitudinal fasciculus L; IDP dMRI TBSS ICVF SLF L: IDP dMRI TBSS ICVF Superior longitudinal fasciculus L; IDP dMRI TBSS L1 ALIC L: IDP dMRI TBSS L1 Anterior limb of internal capsule L; IDP dMRI TBSS L1 GCC: IDP dMRI TBSS L1

Genu of corpus callosum; IDP dMRI TBSS L1 PLIC L: IDP dMRI TBSS L1 Posterior limb of internal capsule L; IDP dMRI TBSS L1 PLIC L: IDP dMRI TBSS L1 Posterior limb of internal capsule L; IDP dMRI TBSS L1 PLIC R: IDP dMRI TBSS L1 Posterior limb of internal capsule R; IDP dMRI TBSS L1 PLIC R: IDP dMRI TBSS L1 Posterior limb of internal capsule R; IDP dMRI TBSS L1 SLF R: IDP dMRI TBSS L1 Superior longitudinal fasciculus R; IDP dMRI TBSS L2 CCG L: IDP dMRI TBSS L2 Cingulum cingulate gyrus L; IDP dMRI TBSS L2 PLIC L: IDP dMRI TBSS L2 Posterior limb of internal capsule L; IDP dMRI TBSS L2 RPIC L: IDP dMRI TBSS L2 Retrolenticular part of internal capsule L; IDP dMRI TBSS L2 SCC: IDP dMRI TBSS L2 Splenium of corpus callosum; IDP dMRI TBSS L2 SCC: IDP dMRI TBSS L2 Splenium of corpus callosum; IDP dMRI TBSS L2 SCC: IDP dMRI TBSS L2 Splenium of corpus callosum; IDP dMRI TBSS L2 SCP L: IDP dMRI TBSS L2 Superior cerebellar peduncle L; IDP dMRI TBSS L2 SCP R: IDP dMRI TBSS L2 Superior cerebellar peduncle R; IDP dMRI TBSS L3 ALIC L: IDP dMRI TBSS L3 Anterior limb of internal capsule L; IDP dMRI TBSS L3 ALIC L: IDP dMRI TBSS L3 Anterior limb of internal capsule L; IDP dMRI TBSS L3 ALIC R: IDP dMRI TBSS L3 Anterior limb of internal capsule R; IDP dMRI TBSS L3 MCP: IDP dMRI TBSS L3 Middle cerebellar peduncle; IDP dMRI TBSS L3 PCR L: IDP dMRI TBSS L3 Posterior corona radiata L; IDP dMRI TBSS L3 RPIC L: IDP dMRI TBSS L3 Retrolenticular part of internal capsule L; IDP dMRI TBSS L3 SCC: IDP dMRI TBSS L3 Splenium of corpus callosum; IDP dMRI TBSS L3 SCP L: IDP dMRI TBSS L3 Superior cerebellar peduncle L; IDP dMRI TBSS L3 SCP R: IDP dMRI TBSS L3 Superior cerebellar peduncle R; IDP dMRI TBSS L3 SCR L: IDP

dMRI TBSS L3 Superior corona radiata L; IDP dMRI TBSS L3 SCR R: IDP dMRI TBSS L3 Superior corona radiata R; IDP dMRI TBSS MD ALIC L: IDP dMRI TBSS MD Anterior limb of internal capsule L; IDP dMRI TBSS MD CCG L: IDP dMRI TBSS MD Cingulum cingulate gyrus L; IDP dMRI TBSS MD CCG R: IDP dMRI TBSS MD Cingulum cingulate gyrus R; IDP dMRI TBSS MD ICP L: IDP dMRI TBSS MD Inferior cerebellar peduncle L; IDP dMRI TBSS MD SCC: IDP dMRI TBSS MD Splenium of corpus callosum; IDP dMRI TBSS MD SCP L: IDP dMRI TBSS MD Superior cerebellar peduncle L; IDP dMRI TBSS MD SCP R: IDP dMRI TBSS MD Superior cerebellar peduncle R; IDP dMRI TBSS MD SFOF L: IDP dMRI TBSS MD Superior fronto-occipital fasciculus L; IDP dMRI TBSS OD PCR R: IDP dMRI TBSS OD Posterior corona radiata R; IDP dMRI TBSS OD PLIC L: IDP dMRI TBSS OD Posterior limb of internal capsule L; IDP dMRI TBSS OD PLIC L: IDP dMRI TBSS OD Posterior limb of internal capsule L; IDP dMRI TBSS OD SLF R: IDP dMRI TBSS OD Superior longitudinal fasciculus R; IDP dMRI TBSS OD SLF R: IDP dMRI TBSS OD Superior longitudinal fasciculus R; IDP SWI T2star left plus right caudate: IDP SWI T2star left caudate plus IDP SWI T2star right caudate; IDP SWI T2star left plus right caudate: IDP SWI T2star left caudate plus IDP SWI T2star right caudate; IDP SWI T2star left plus right pallidum: IDP SWI T2star left pallidum plus IDP SWI T2star right pallidum; IDP SWI T2star left plus right putamen: IDP SWI T2star left putamen plus IDP SWI T2star right putamen; IDP SWI T2star left plus right putamen: IDP SWI T2star left putamen plus IDP SWI T2star right putamen; IDP SWI T2star left plus right thalamus: IDP SWI T2star left thalamus plus IDP SWI T2star right thalamus; IDP SWI

T2star left plus right thalamus: IDP SWI T2star left thalamus plus IDP SWI T2star right thalamus; IDP T1 FIRST left plus right pallidum volume: IDP T1 FIRST left pallidum volume plus IDP T1 FIRST right pallidum volume; IDP T1 FIRST left plus right putamen volume: IDP T1 FIRST left putamen volume plus IDP T1 FIRST right putamen volume; IDP: image-derived phenotype; IV: instrumental variable; ivw: Inverse variance weighted; MHC: Major Histocompatibility Complex; MR: Mendelian Randomization; MRI: Magnetic resonance imaging; MS: multiple sclerosis; MSKCTD: Diseases of the musculoskeletal system and connective tissue; OR: Odds ratio; SCTD: Systemic connective tissue disorders; Sjogren's syndrome: Sjogren's syndrome (Firth correction); SLE: Systemic lupus erythematosus; SNP: single nucleotide polymorphism; TMD-MP-FM: TMD muscular pain lnked with fibromyalgia; UKB: UK Biobank; Vasculitis limited to skin: Vasculitis limited to skin, not elsewhere classified; W-median: Weighted median; W-mode: Weighted mode

A

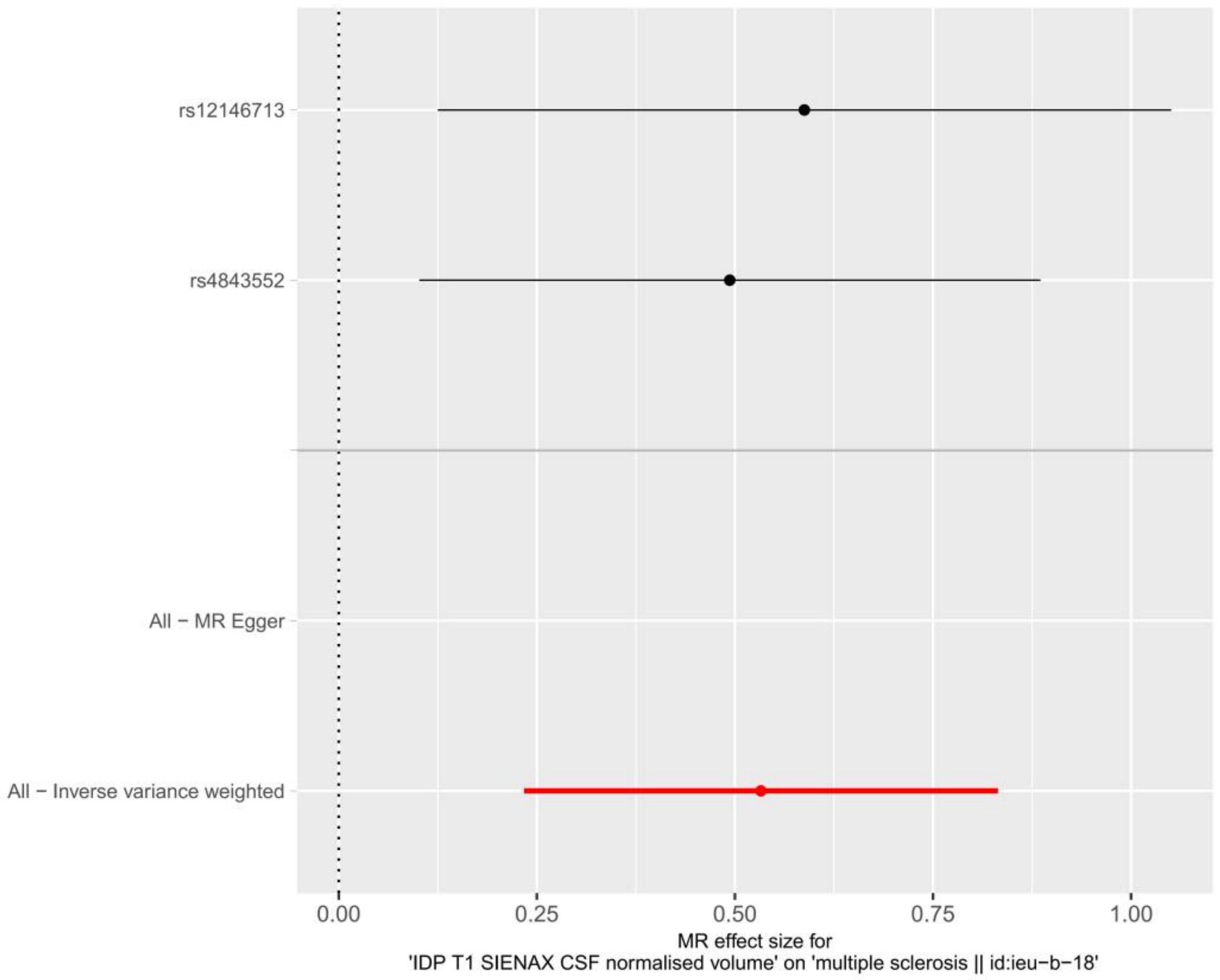
rs12146713
rs4843552
All − MR Egger
All − Inverse variance weighted
0.00
0.25
0.50
0.75
1.00
MR effect size for
'IDP T1 SIENAX CSF normalised volume' on 'multiple sclerosis || id:ieu−b−18'

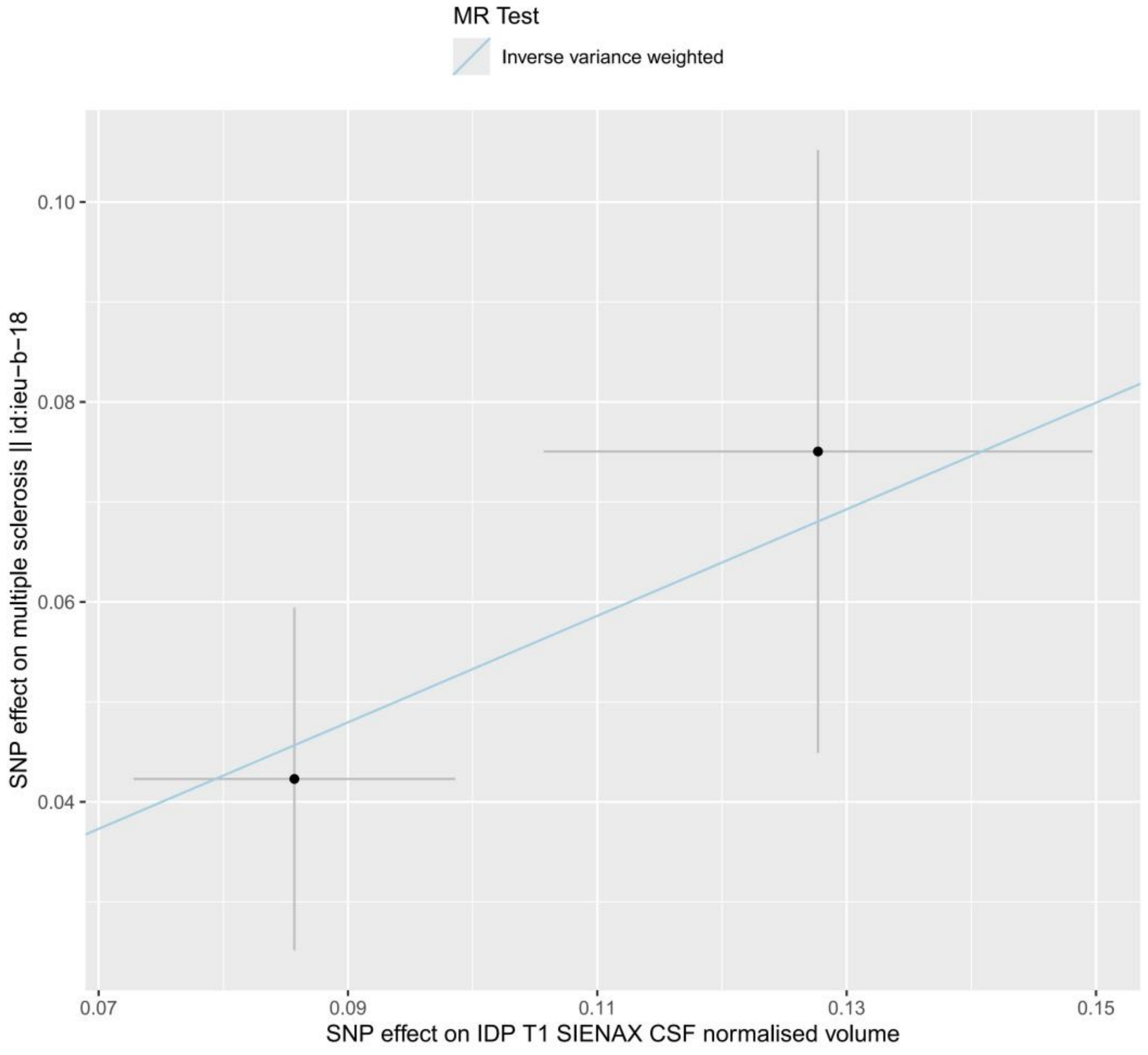
MR Test
Inverse variance weighted
0.10
0.08
0.06
0.04
SNP effect on multiple sclerosis || id:ieu−b−18
0.07
0.09
0.11
0.13
0.15
SNP effect on IDP T1 SIENAX CSF normalised volume

B

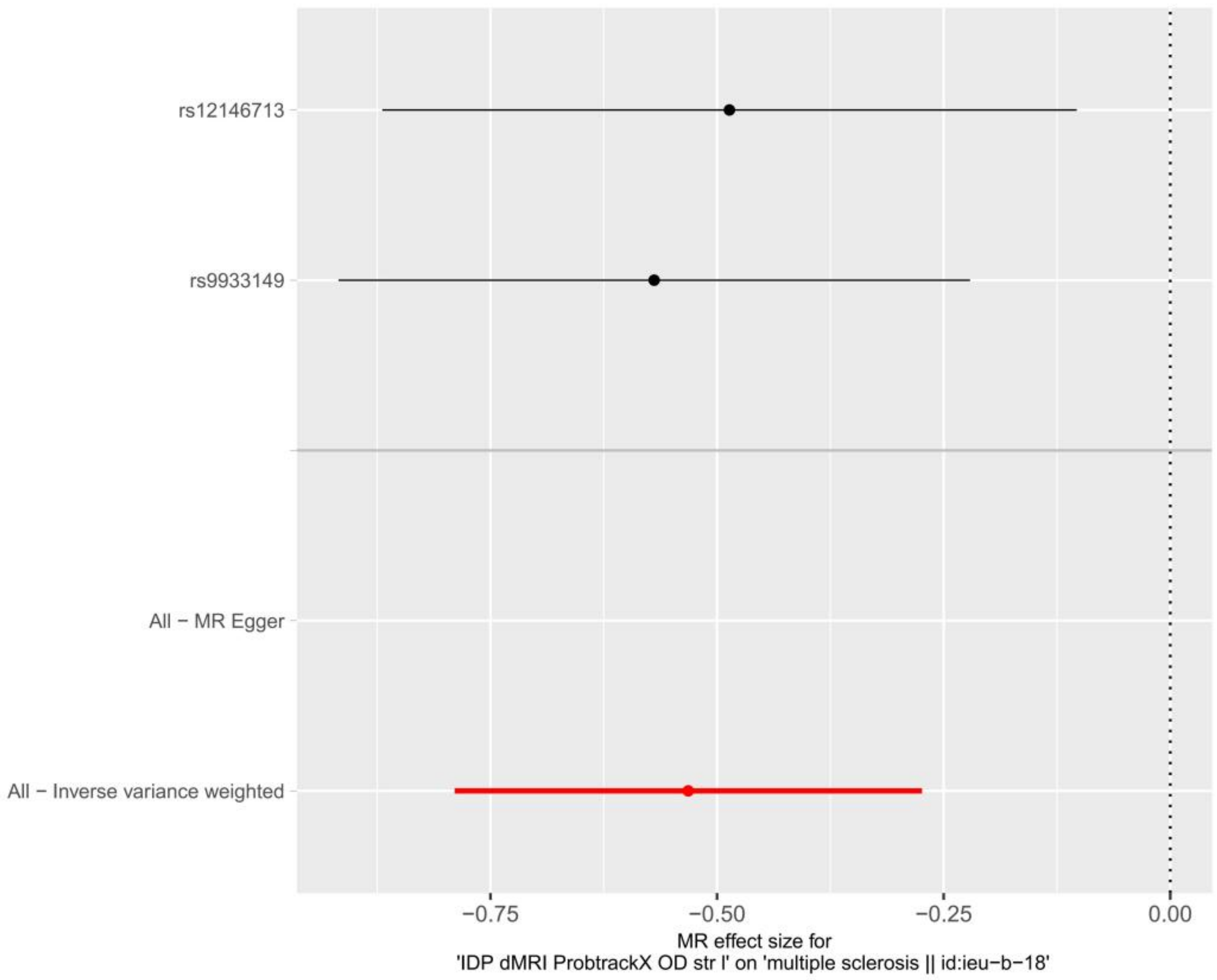

rs12146713
rs9933149
All − MR Egger
All − Inverse variance weighted
−0.75
−0.50
−0.25
0.00
MR effect size for
'IDP dMRI ProbtrackX OD str l' on 'multiple sclerosis || id:ieu−b−18'

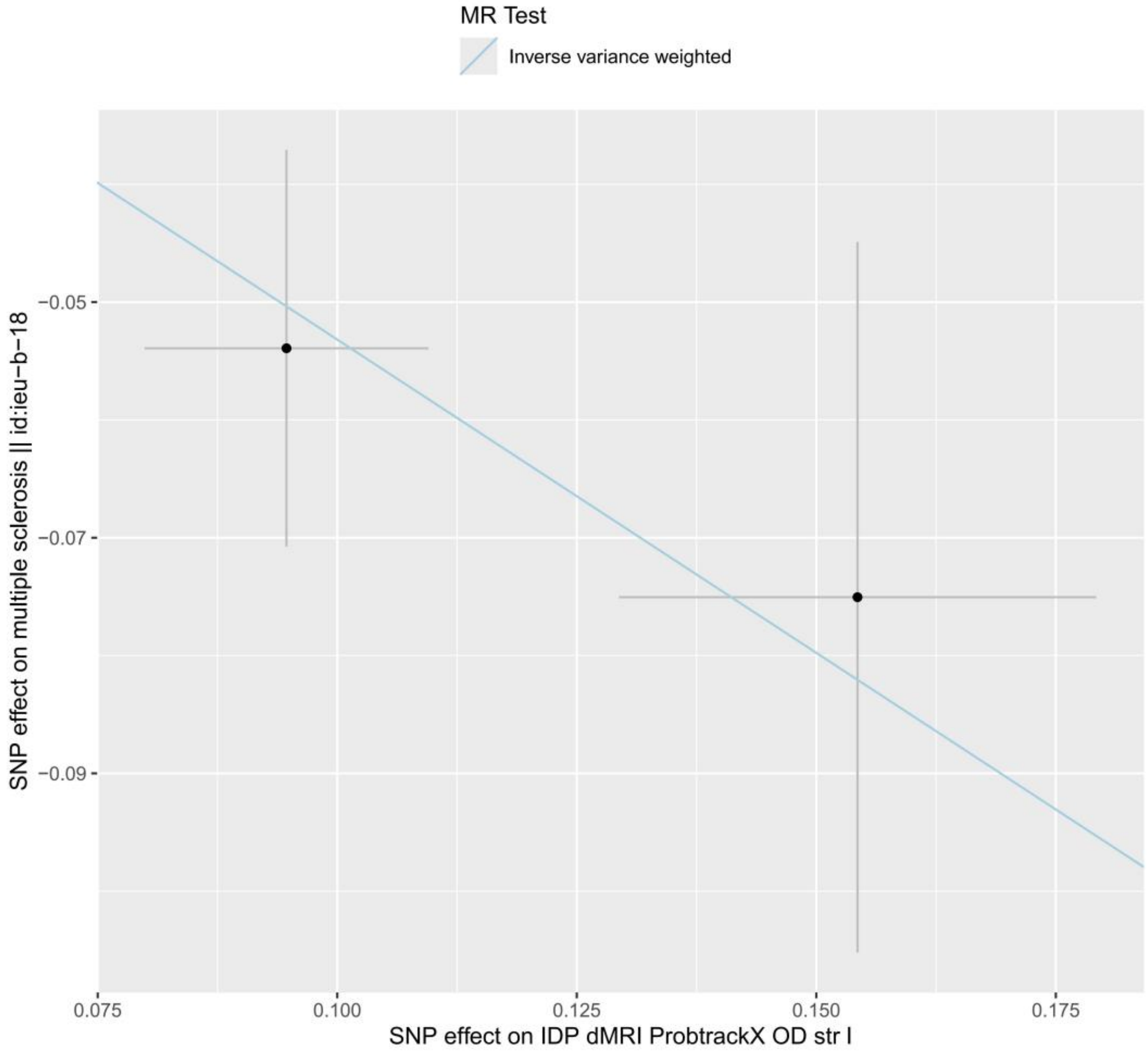

MR Test
Inverse variance weighted
−0.05
−0.07
−0.09
SNP effect on multiple sclerosis || id:ieu−b−18
0.075
0.100
0.125
0.150
0.175
SNP effect on IDP dMRI ProbtrackX OD str l

C

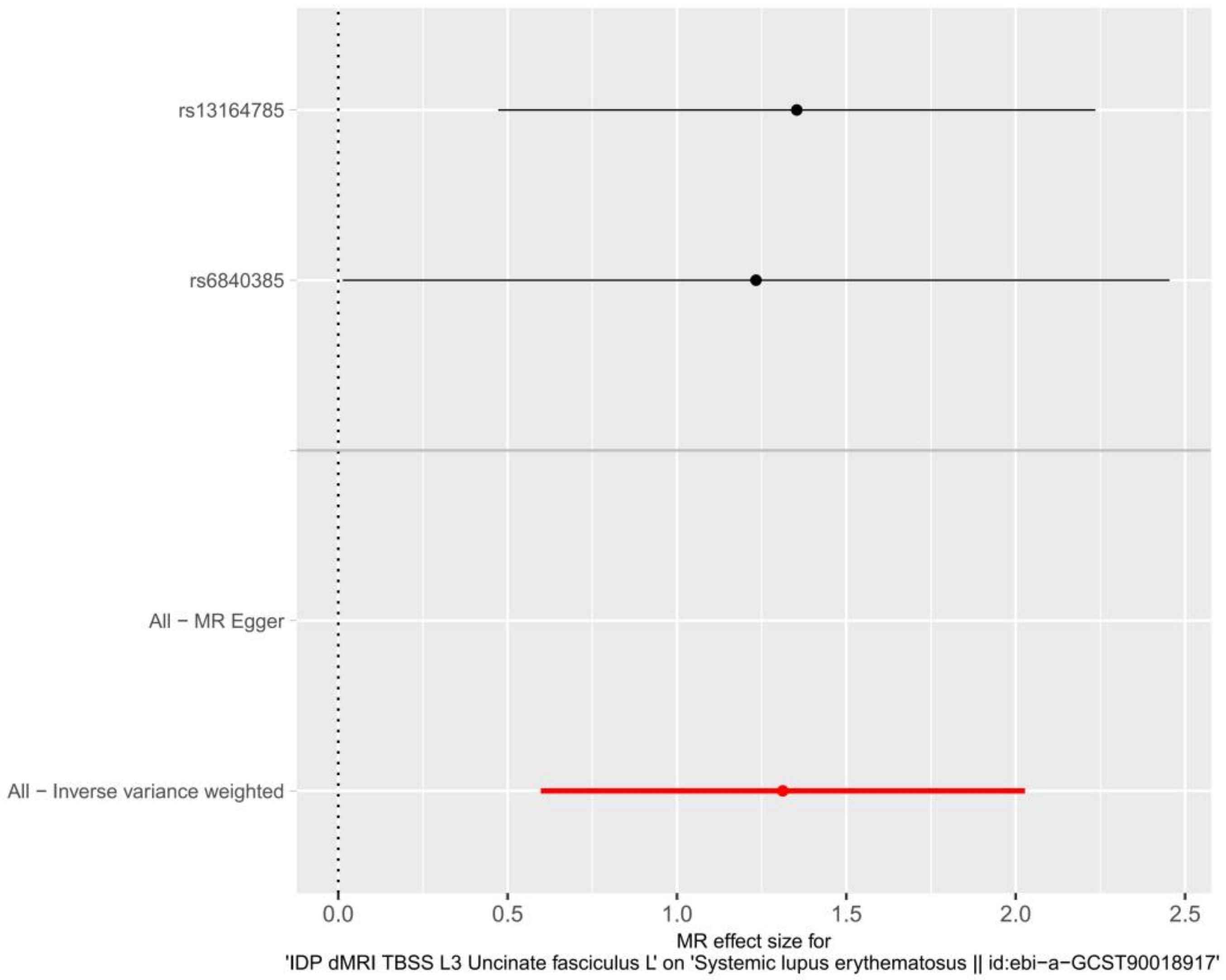
rs13164785
rs6840385
All − MR Egger
All − Inverse variance weighted
0.0
0.5
1.0
1.5
2.0
2.5
MR effect size for
'IDP dMRI TBSS L3 Uncinate fasciculus L' on 'Systemic lupus erythematosus || id:ebi−a−GCST90018917'

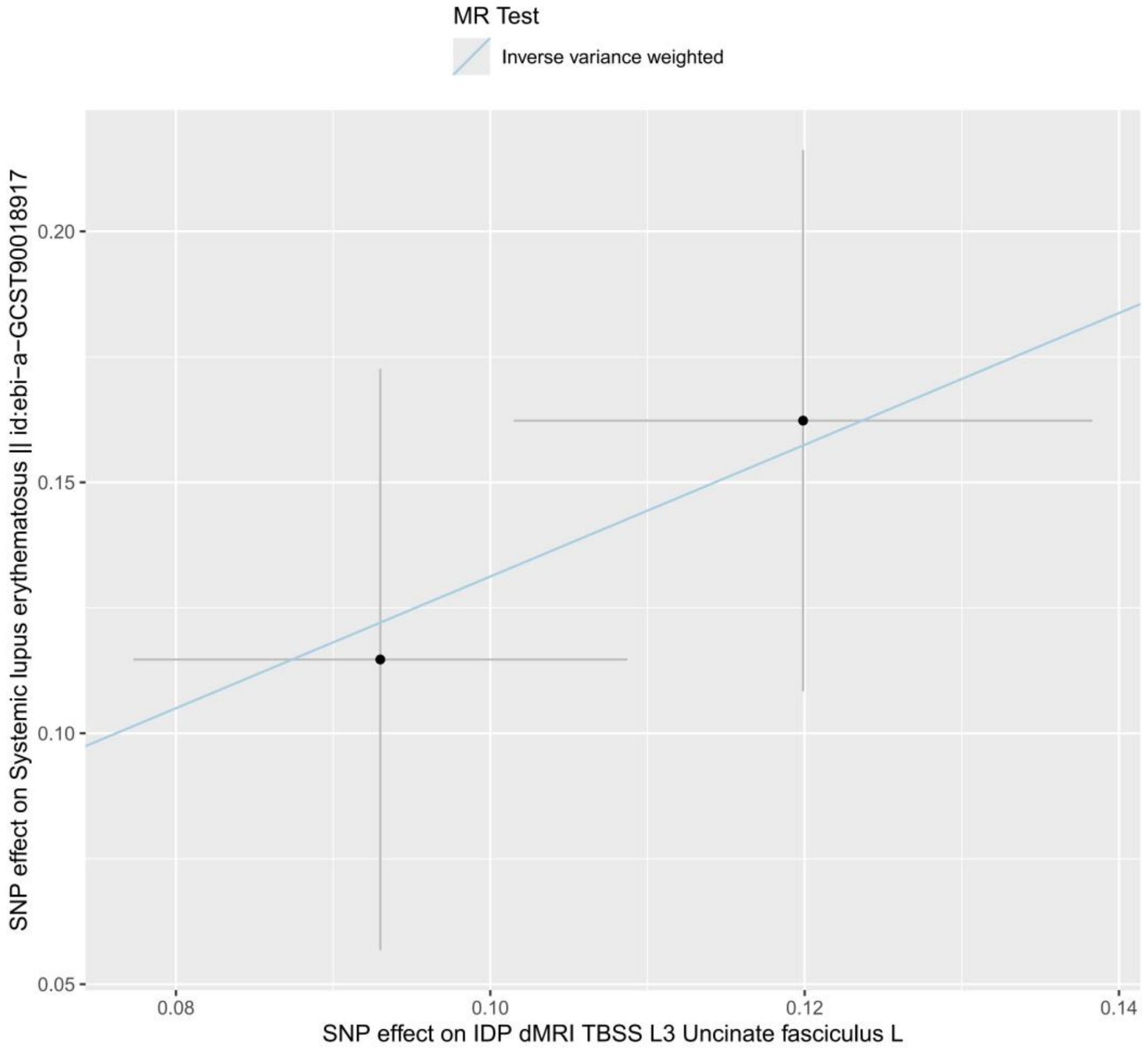
MR Test
Inverse variance weighted
SNP effect on Systemic lupus erythematosus || id:ebi−a−GCST90018917
0.20
0.15
0.10
0.05
0.08
0.10
0.12
0.14
SNP effect on IDP dMRI TBSS L3 Uncinate fasciculus L

D

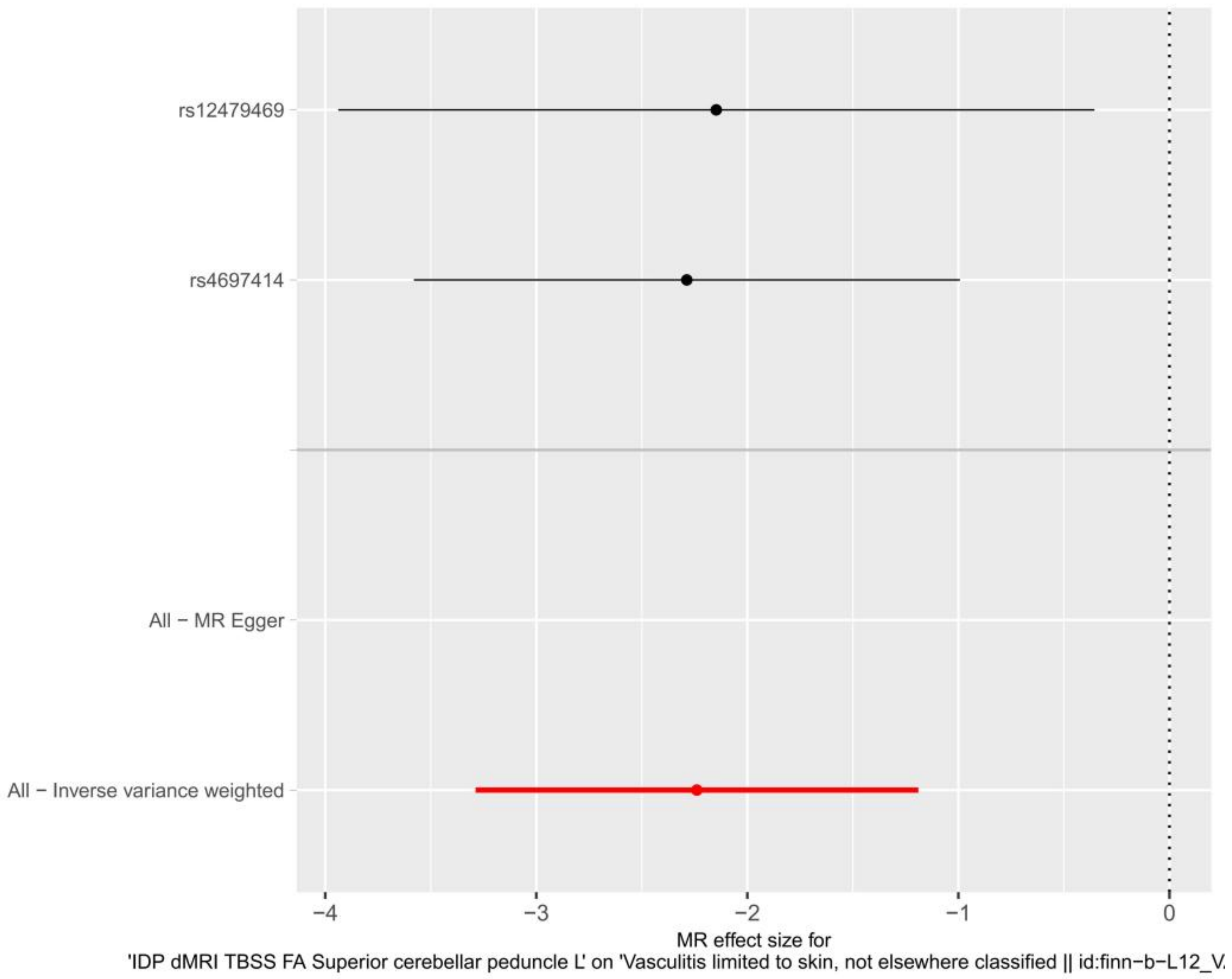
rs12479469
rs4697414
All − MR Egger
All − Inverse variance weighted
−4
−3
−2
−1
0
MR effect size for
'IDP dMRI TBSS FA Superior cerebellar peduncle L' on 'Vasculitis limited to skin, not elsewhere classified || id:finn−b−L12_V

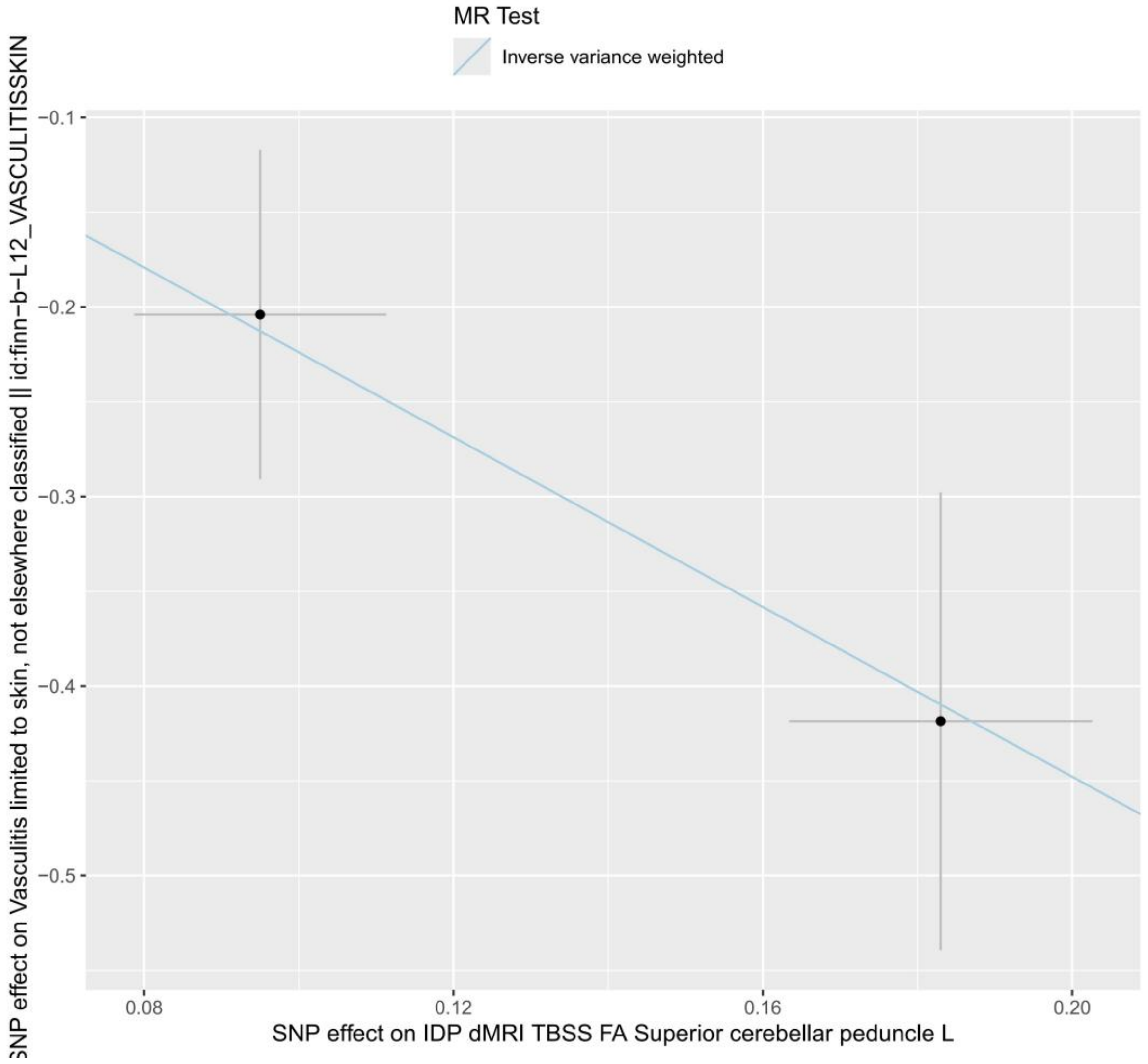
MR Test
Inverse variance weighted
−0.1
−0.2
−0.3
−0.4
−0.5
0.08
0.12
0.16
0.20
SNP effect on IDP dMRI TBSS FA Superior cerebellar peduncle L
SNP effect on Vasculitis limited to skin, not elsewhere classified || id:finn−b−L12_VASCULITISSKIN

E

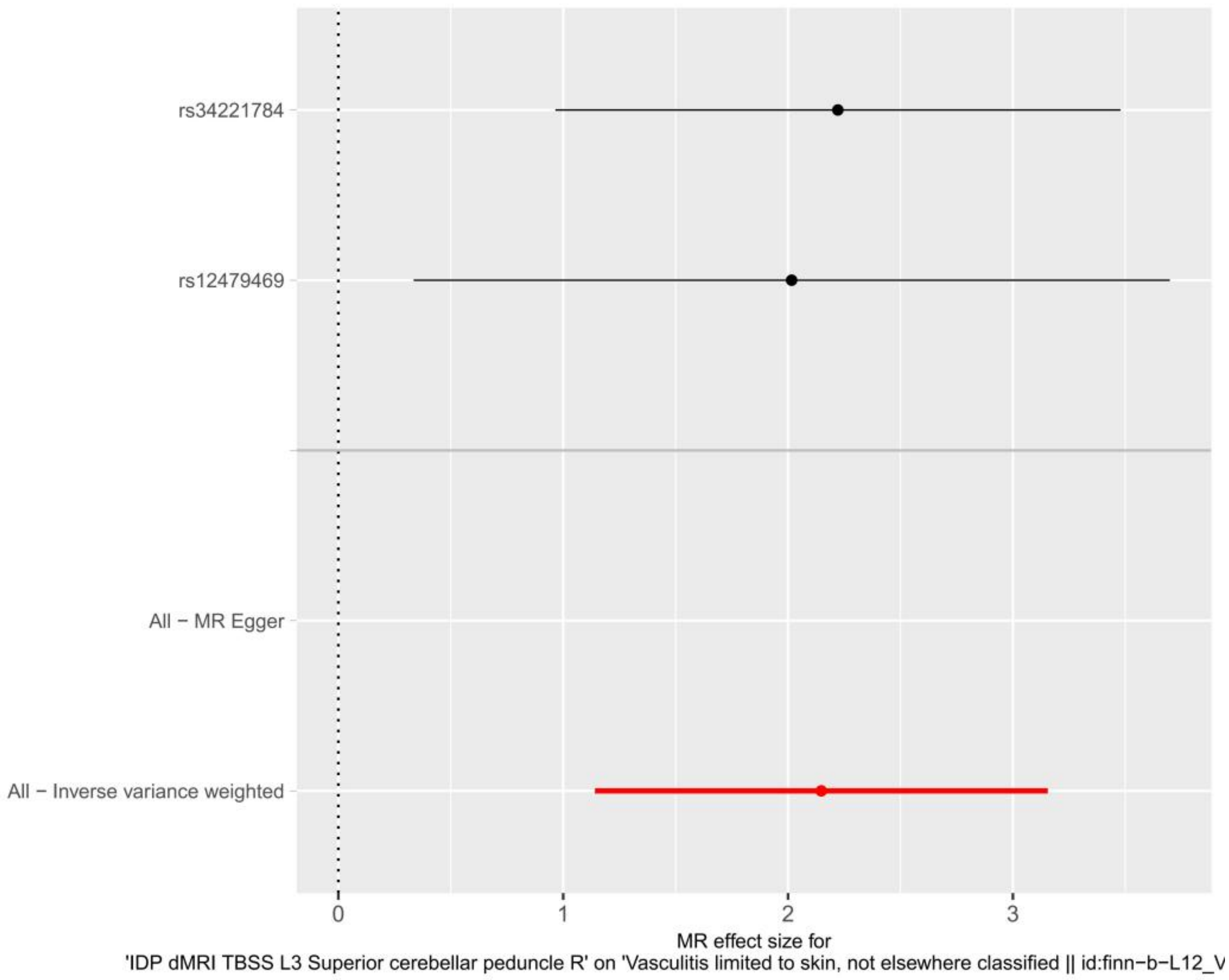
rs34221784
rs12479469
All − MR Egger
All − Inverse variance weighted
0
1
2
3
MR effect size for
'IDP dMRI TBSS L3 Superior cerebellar peduncle R' on 'Vasculitis limited to skin, not elsewhere classified || id:finn−b−L12_V

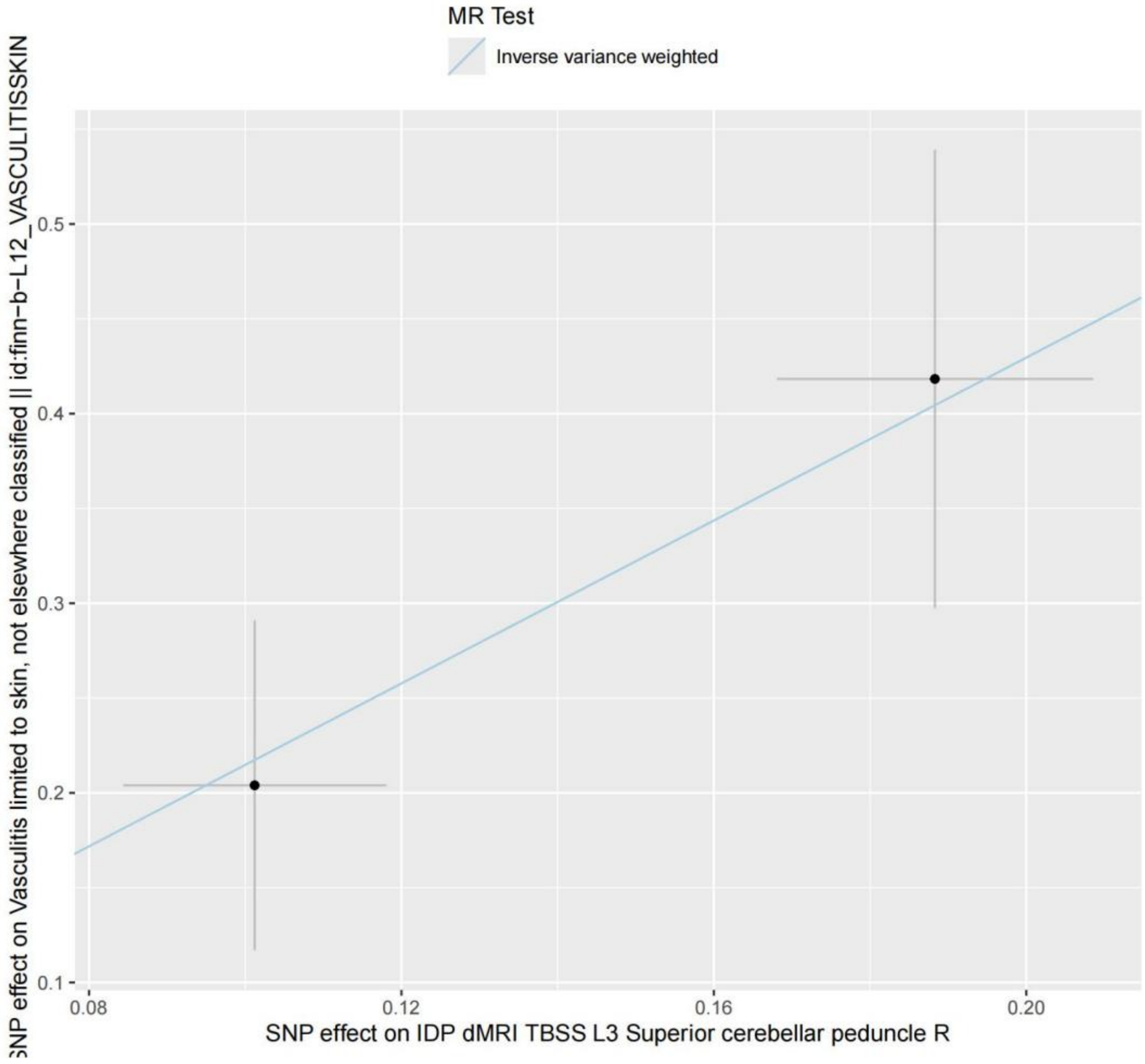
MR Test
Inverse variance weighted
SNP effect on Vasculitis limited to skin, not elsewhere classified || id:finn−b−L12_VASCULITISSKIN
0.5
0.4
0.3
0.2
0.1
0.08
0.12
0.16
0.20
SNP effect on IDP dMRI TBSS L3 Superior cerebellar peduncle R

F

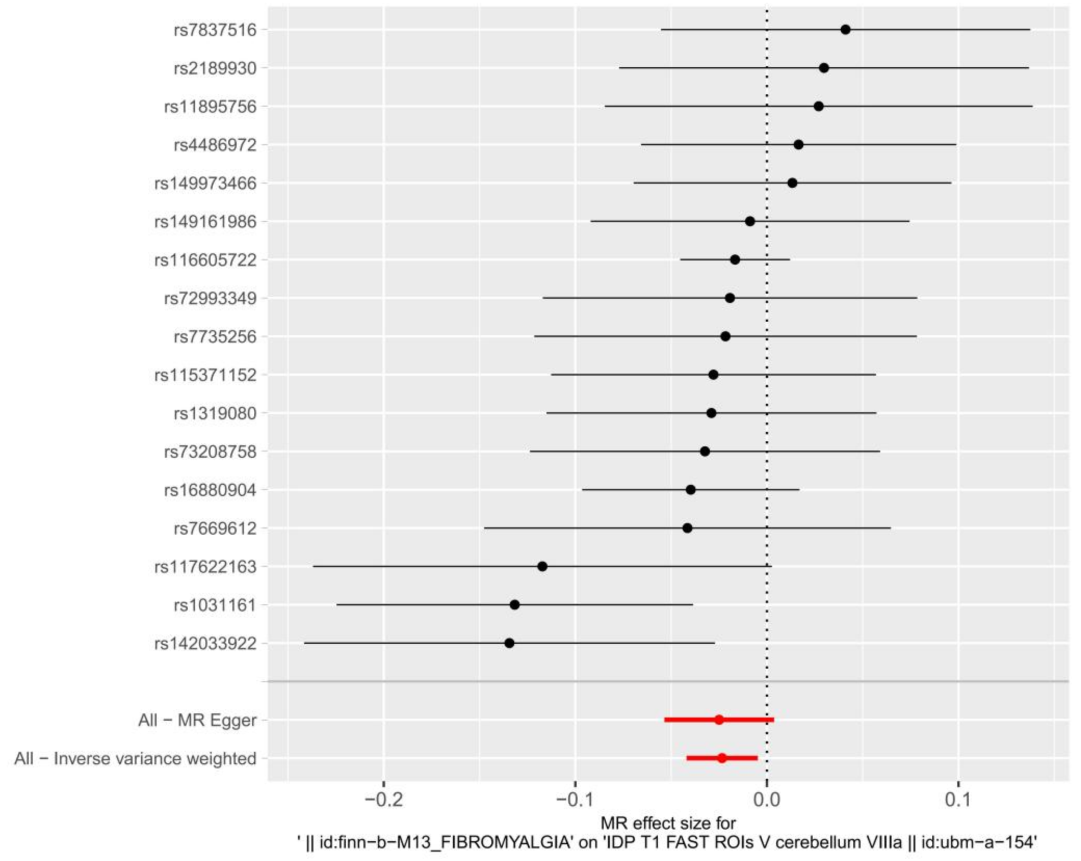
rs7837516
rs2189930
rs11895756
rs4486972
rs149973466
rs149161986
rs116605722
rs72993349
rs7735256
rs115371152
rs1319080
rs73208758
rs16880904
rs7669612
rs117622163
rs1031161
rs142033922
All − MR Egger
All − Inverse variance weighted
MR effect size for
' || id:finn-b-M13_FIBROMYALGIA' on 'IDP T1 FAST ROIs V cerebellum VIIIa || id:ubm-a-154'

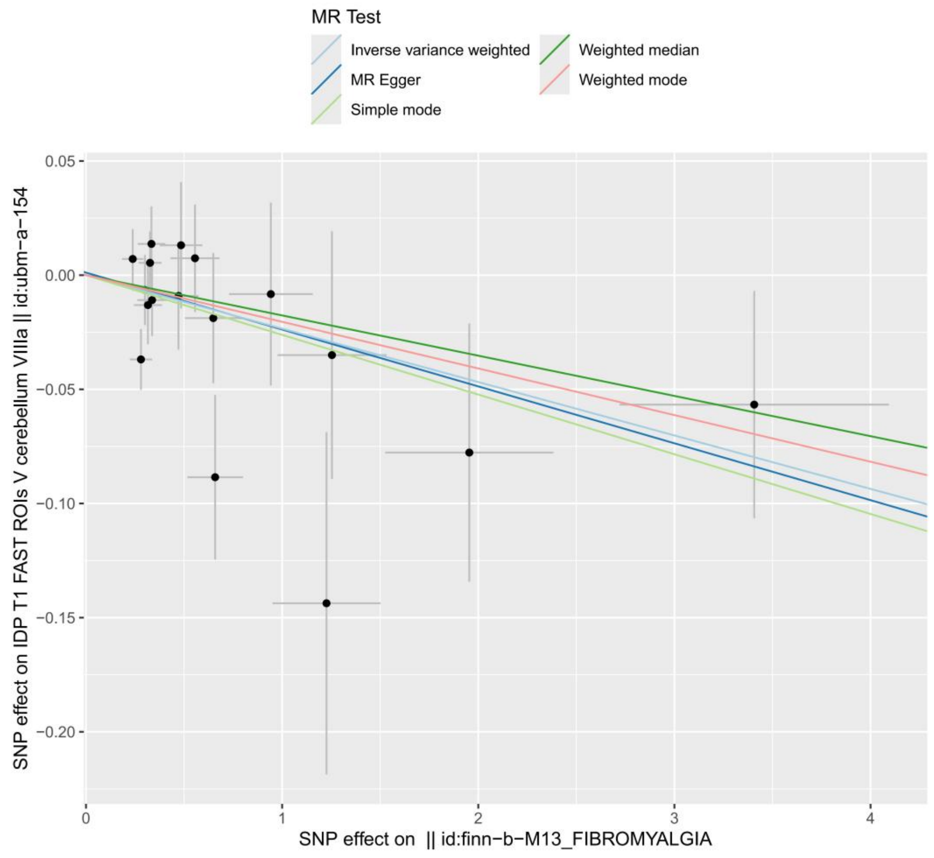
MR Test
Inverse variance weighted
MR Egger
Simple mode
Weighted median
Weighted mode
SNP effect on IDP T1 FAST ROIs V cerebellum VIIIa || id:ubm-a-154
SNP effect on || id:finn-b-M13_FIBROMYALGIA

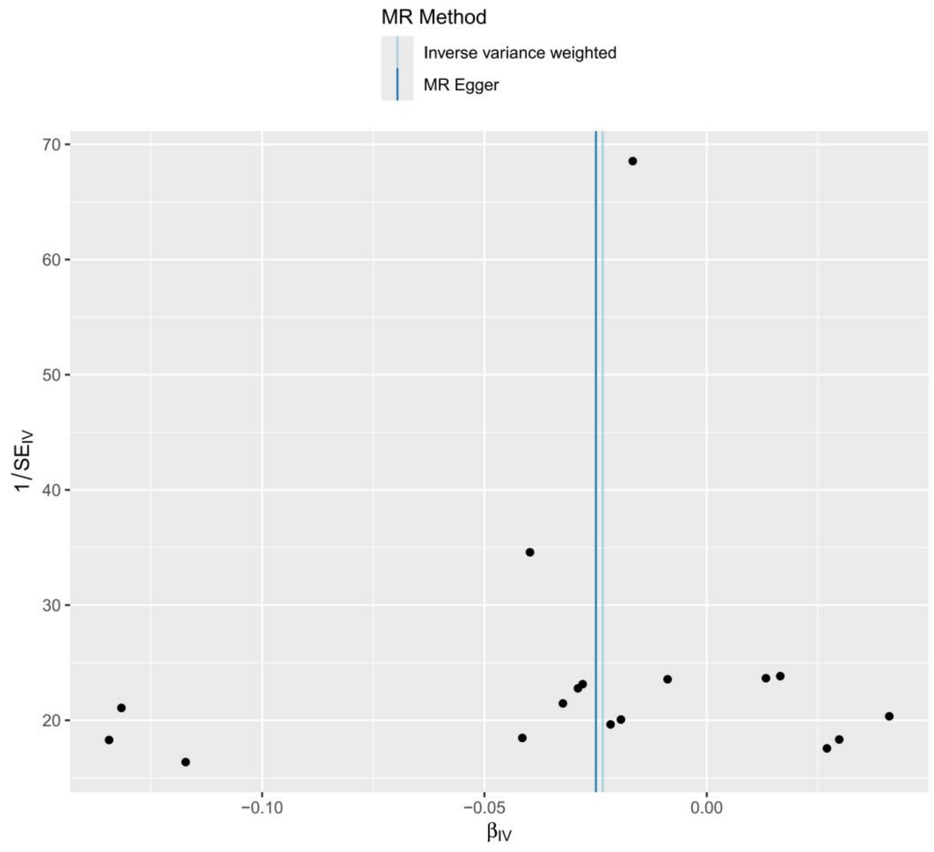
MR Method
Inverse variance weighted
MR Egger
1/SE_IV
β_IV

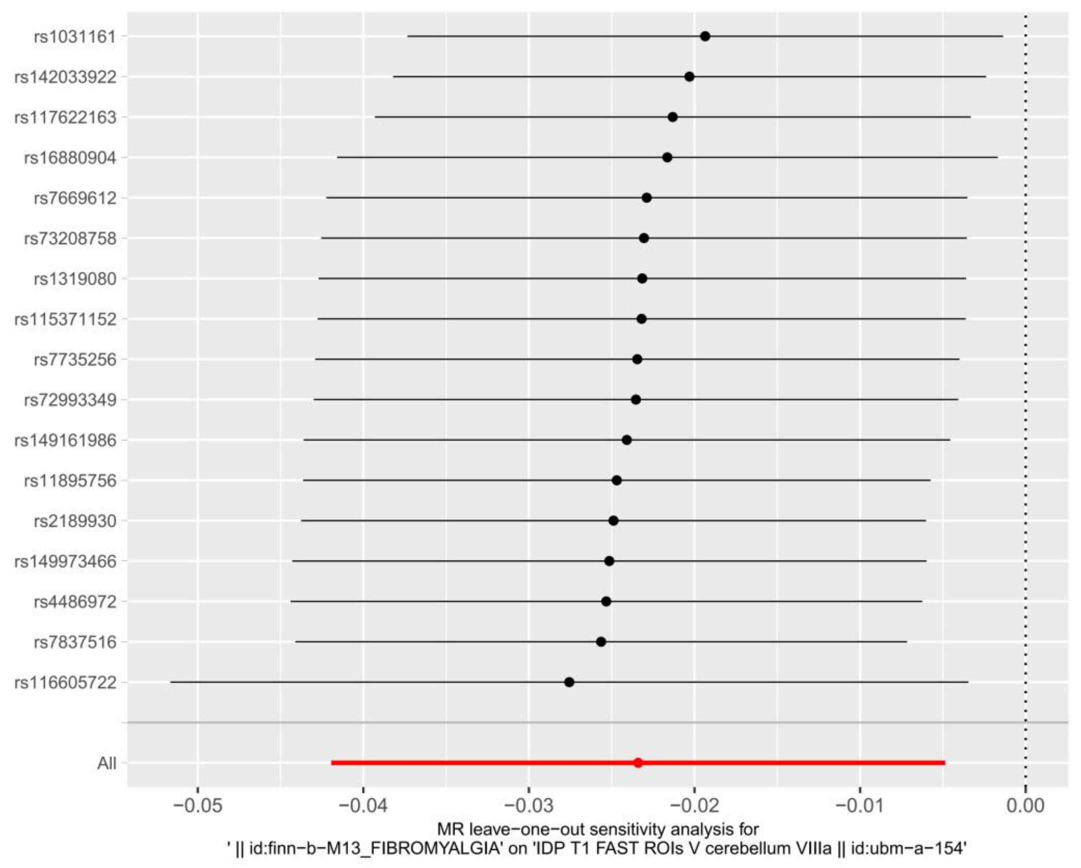
rs1031161
rs142033922
rs117622163
rs16880904
rs7669612
rs73208758
rs1319080
rs115371152
rs7735256
rs72993349
rs149161986
rs11895756
rs2189930
rs149973466
rs4486972
rs7837516
rs116605722
All
MR leave−one−out sensitivity analysis for
' || id:finn-b-M13_FIBROMYALGIA' on 'IDP T1 FAST ROIs V cerebellum VIIIa || id:ubm-a-154'

G

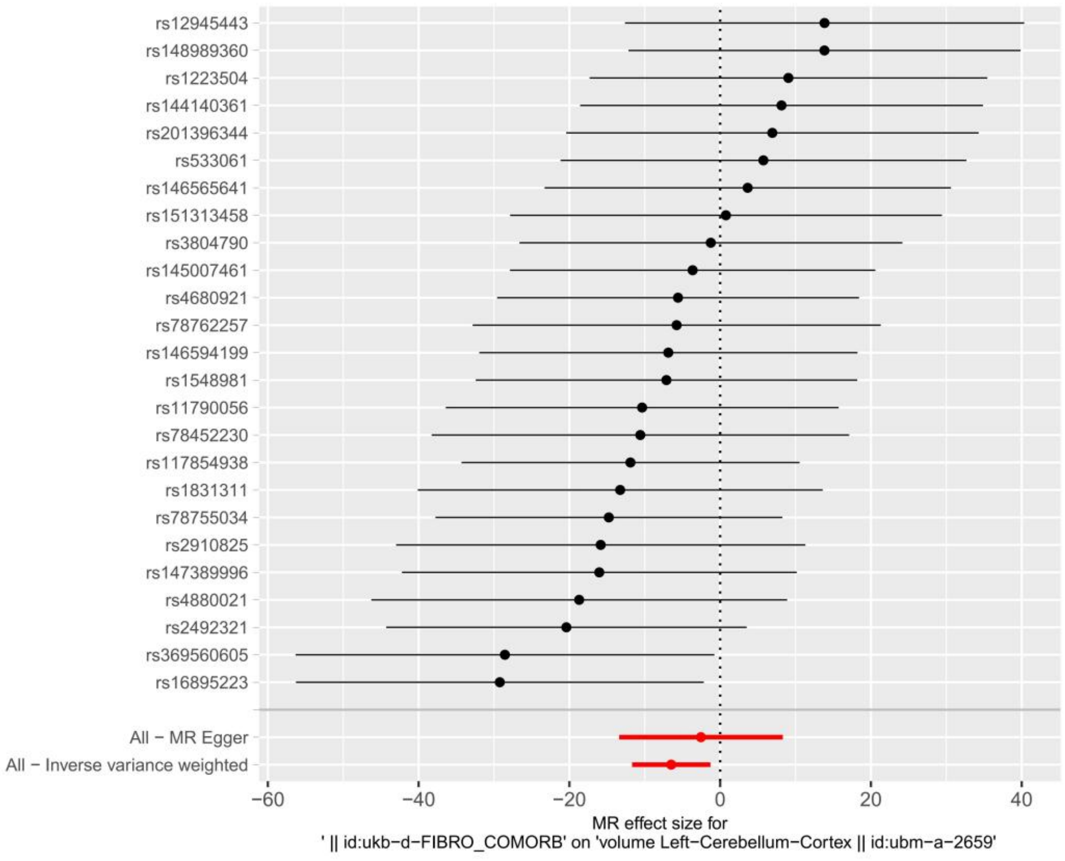
rs12945443
rs148989360
rs1223504
rs144140361
rs201396344
rs533061
rs146565641
rs151313458
rs3804790
rs145007461
rs4680921
rs78762257
rs146594199
rs1548981
rs11790056
rs78452230
rs117854938
rs1831311
rs78755034
rs2910825
rs147389996
rs4880021
rs2492321
rs369560605
rs16895223
All − MR Egger
All − Inverse variance weighted
−60
−40
−20
0
20
40
MR effect size for
' || id:ukb−d−FIBRO_COMORB' on 'volume Left−Cerebellum−Cortex || id:ubm−a−2659'

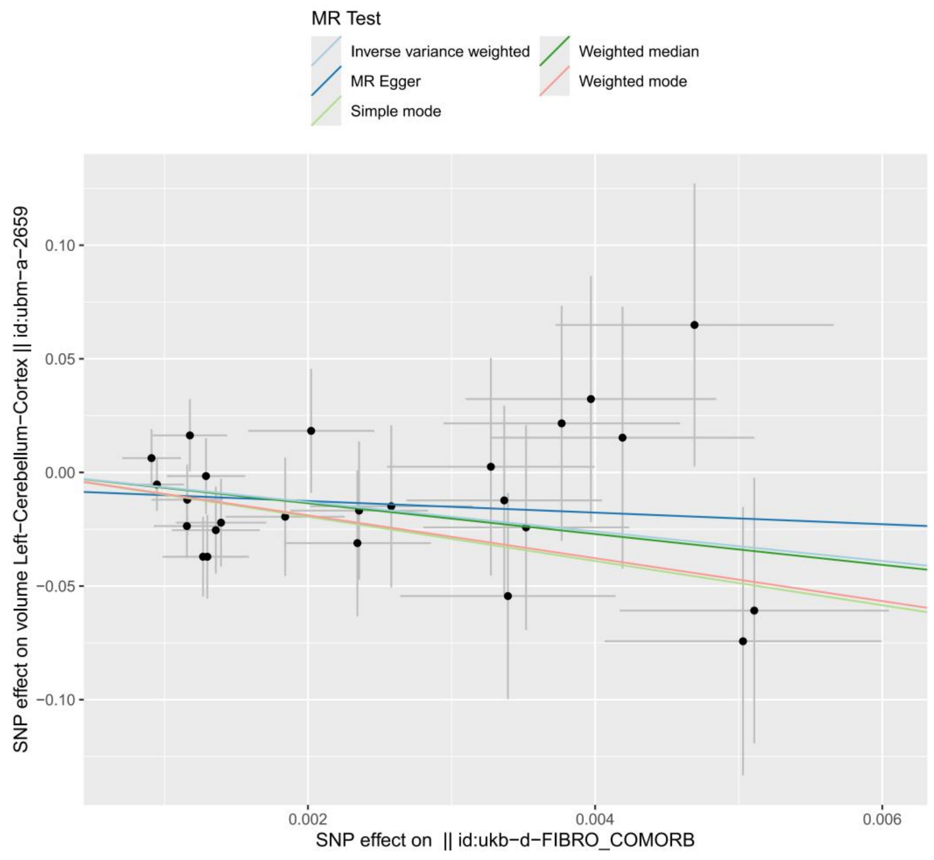
MR Test
Inverse variance weighted
MR Egger
Simple mode
Weighted median
Weighted mode
SNP effect on volume Left−Cerebellum−Cortex || id:ubm−a−2659
0.10
0.05
0.00
−0.05
−0.10
0.002
0.004
0.006
SNP effect on || id:ukb−d−FIBRO_COMORB

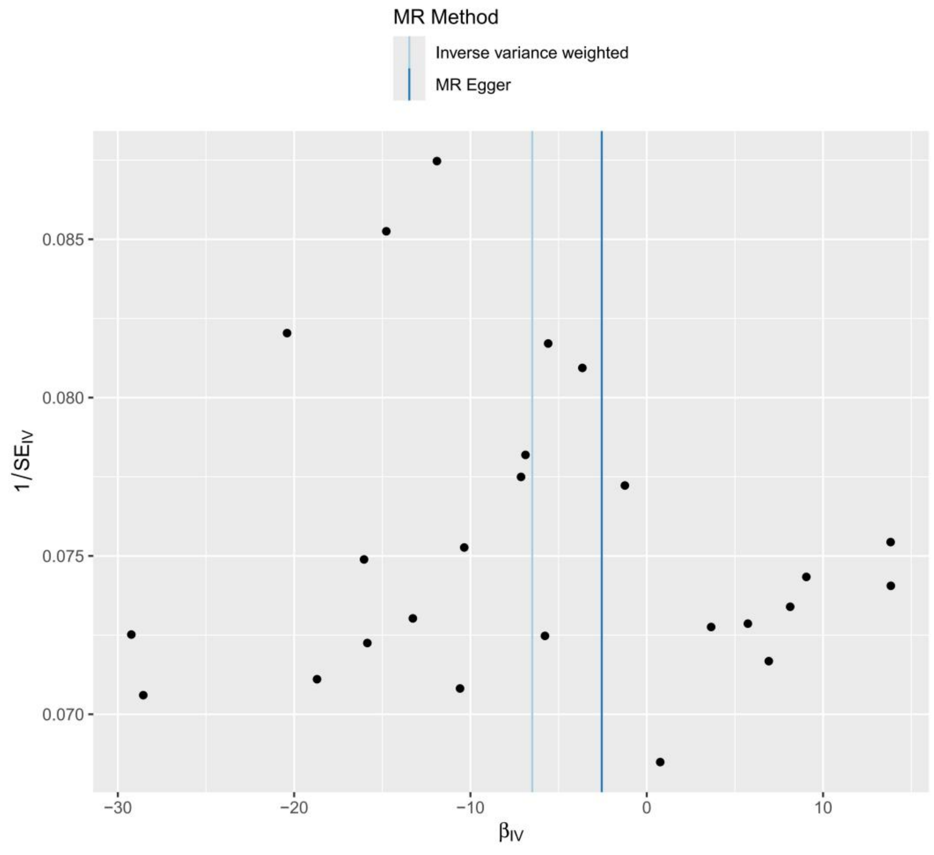
MR Method
Inverse variance weighted
MR Egger
1/SE$_{IV}$
0.085
0.080
0.075
0.070
−30
−20
−10
0
10
β$_{IV}$

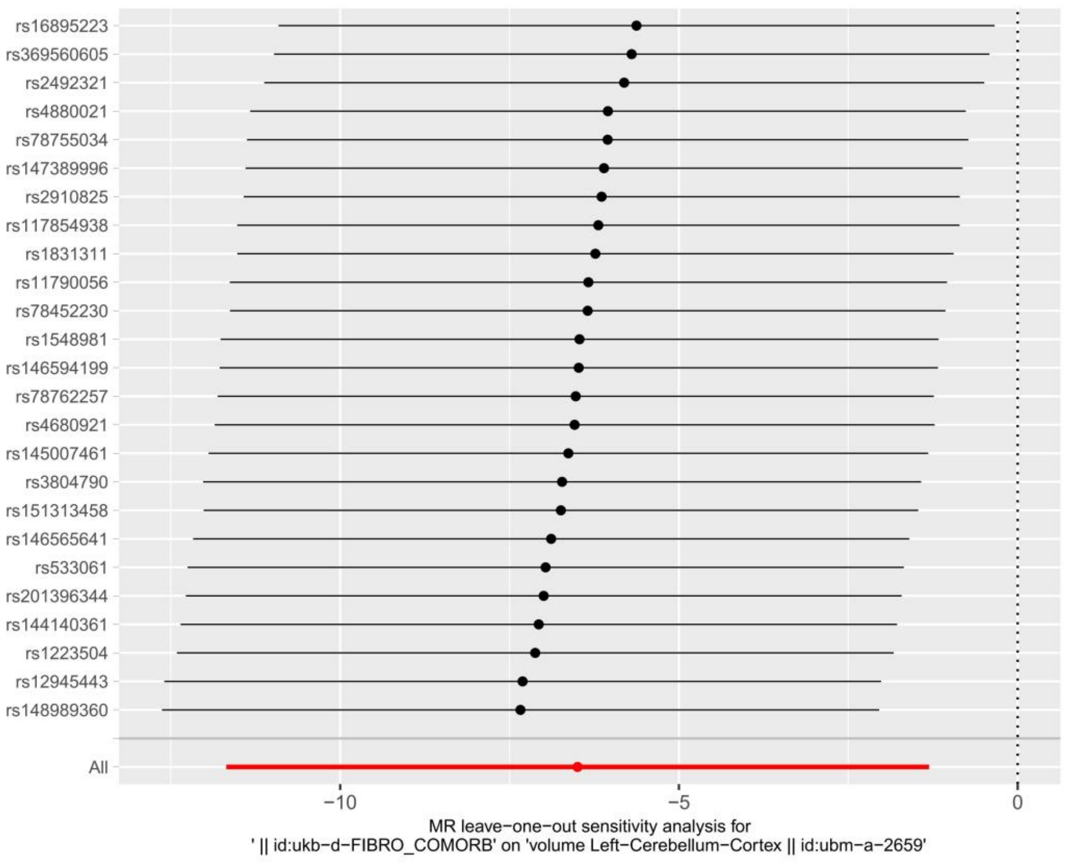
rs16895223
rs369560605
rs2492321
rs4880021
rs78755034
rs147389996
rs2910825
rs117854938
rs1831311
rs11790056
rs78452230
rs1548981
rs146594199
rs78762257
rs4680921
rs145007461
rs3804790
rs151313458
rs146565641
rs533061
rs201396344
rs144140361
rs1223504
rs12945443
rs148989360
All
−10
−5
0
MR leave−one−out sensitivity analysis for
' || id:ukb−d−FIBRO_COMORB' on 'volume Left−Cerebellum−Cortex || id:ubm−a−2659'

Figure S1. Visualization results of heterogeneity and pleiotropy analyses

(A – E) Forward MR analyses: forest and scatter plots showing causal effects of selected imaging-derived phenotypes (IDPs) on autoimmune inflammatory diseases (AIDs). Specifically:
(A) IDP T1 SIENAX CSF normalized volume → multiple sclerosis;
(B) IDP dMRI ProbtrackX OD str l → multiple sclerosis;
(C) IDP dMRI TBSS L3 Uncinate fasciculus L → systemic lupus erythematosus;
(D) IDP dMRI TBSS FA Superior cerebellar peduncle L → vasculitis limited to skin;
(E) IDP dMRI TBSS L3 Superior cerebellar peduncle R → vasculitis limited to skin.
(F – G) Reverse MR analyses: forest, scatter, funnel, and leave-one-out (LOO) plots for (F) IDP T1 FAST ROIs V Cerebellum VIIIa → Fibromyalgia, and (G) volume Left-Cerebellum-Cortex → Fibromyalgia-related co-morbidities.
In forward MR analyses (A – E), only two SNPs were available as instrumental variables (n = 2), and funnel or LOO analyses were not applicable. In reverse MR (F – G), sensitivity analyses included funnel plots, MR-Egger intercept, and LOO tests; no substantial heterogeneity or horizontal pleiotropy was detected ($P > 0.05$ for MR-Egger intercept).
Statistical significance for MR estimates was evaluated using two-sided tests with Bonferroni correction ($P < 0.05 / 87$ for forward MR, $P < 0.05 / 3$ for reverse MR).

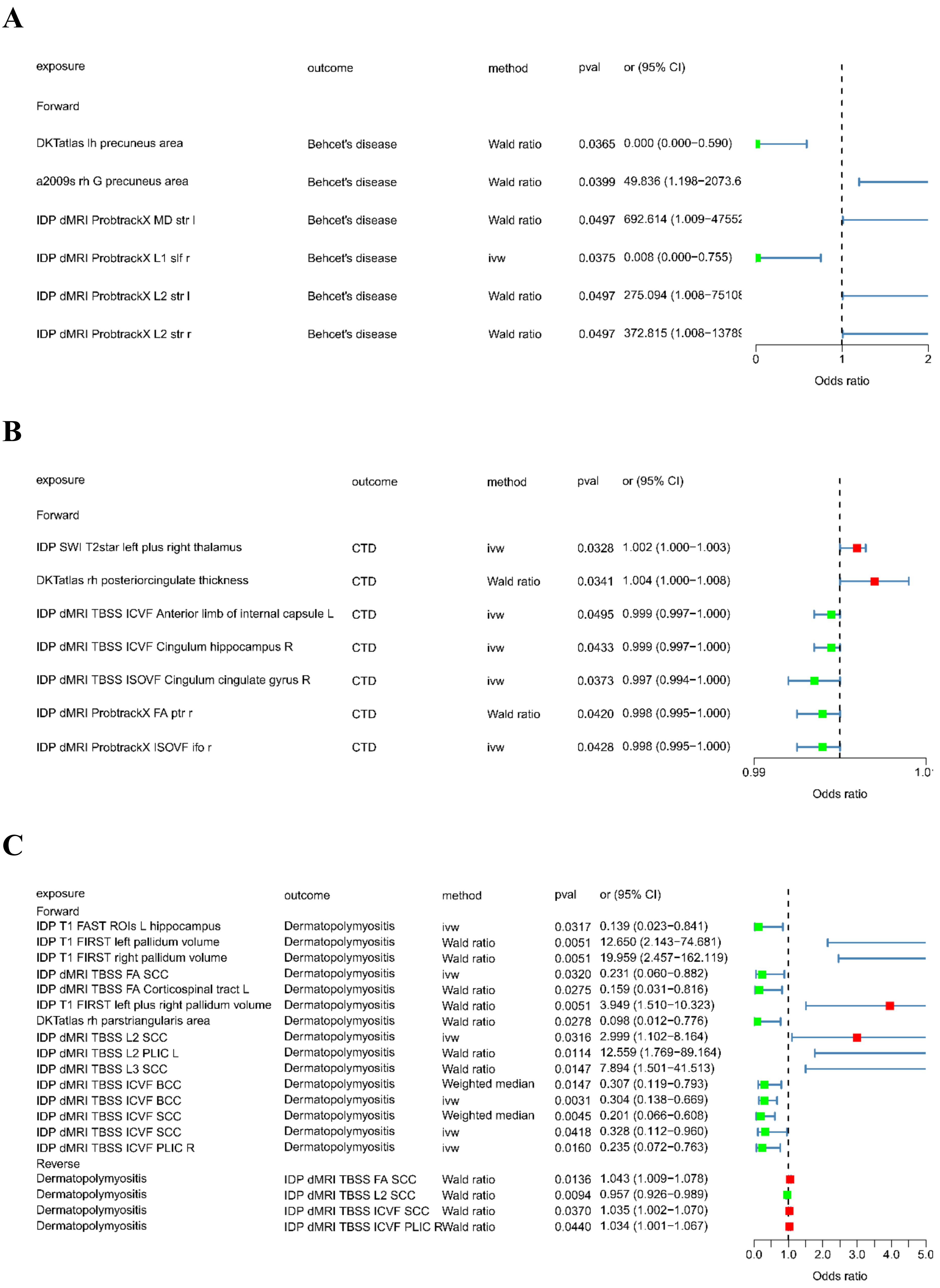
A
exposure
outcome
method
pval
or (95% CI)
Forward
DKTatlas lh precuneus area
Behcet's disease
Wald ratio
0.0365
0.000 (0.000−0.590)
a2009s rh G precuneus area
Behcet's disease
Wald ratio
0.0399
49.836 (1.198−2073.6
IDP dMRI ProbtrackX MD str l
Behcet's disease
Wald ratio
0.0497
692.614 (1.009−47552
IDP dMRI ProbtrackX L1 slf r
Behcet's disease
ivw
0.0375
0.008 (0.000−0.755)
IDP dMRI ProbtrackX L2 str l
Behcet's disease
Wald ratio
0.0497
275.094 (1.008−75108
IDP dMRI ProbtrackX L2 str r
Behcet's disease
Wald ratio
0.0497
372.815 (1.008−13789
0
1
2
Odds ratio
B
exposure
outcome
method
pval
or (95% CI)
Forward
IDP SWI T2star left plus right thalamus
CTD
ivw
0.0328
1.002 (1.000−1.003)
DKTatlas rh posteriorcingulate thickness
CTD
Wald ratio
0.0341
1.004 (1.000−1.008)
IDP dMRI TBSS ICVF Anterior limb of internal capsule L
CTD
ivw
0.0495
0.999 (0.997−1.000)
IDP dMRI TBSS ICVF Cingulum hippocampus R
CTD
ivw
0.0433
0.999 (0.997−1.000)
IDP dMRI TBSS ISOVF Cingulum cingulate gyrus R
CTD
ivw
0.0373
0.997 (0.994−1.000)
IDP dMRI ProbtrackX FA ptr r
CTD
Wald ratio
0.0420
0.998 (0.995−1.000)
IDP dMRI ProbtrackX ISOVF ifo r
CTD
ivw
0.0428
0.998 (0.995−1.000)
0.99
1.01
Odds ratio
C
exposure
outcome
method
pval
or (95% CI)
Forward
IDP T1 FAST ROIs L hippocampus
Dermatopolymyositis
ivw
0.0317
0.139 (0.023−0.841)
IDP T1 FIRST left pallidum volume
Dermatopolymyositis
Wald ratio
0.0051
12.650 (2.143−74.681)
IDP T1 FIRST right pallidum volume
Dermatopolymyositis
Wald ratio
0.0051
19.959 (2.457−162.119)
IDP dMRI TBSS FA SCC
Dermatopolymyositis
ivw
0.0320
0.231 (0.060−0.882)
IDP dMRI TBSS FA Corticospinal tract L
Dermatopolymyositis
Wald ratio
0.0275
0.159 (0.031−0.816)
IDP T1 FIRST left plus right pallidum volume
Dermatopolymyositis
Wald ratio
0.0051
3.949 (1.510−10.323)
DKTatlas rh parstriangularis area
Dermatopolymyositis
Wald ratio
0.0278
0.098 (0.012−0.776)
IDP dMRI TBSS L2 SCC
Dermatopolymyositis
ivw
0.0316
2.999 (1.102−8.164)
IDP dMRI TBSS L2 PLIC L
Dermatopolymyositis
Wald ratio
0.0114
12.559 (1.769−89.164)
IDP dMRI TBSS L3 SCC
Dermatopolymyositis
Wald ratio
0.0147
7.894 (1.501−41.513)
IDP dMRI TBSS ICVF BCC
Dermatopolymyositis
Weighted median
0.0147
0.307 (0.119−0.793)
IDP dMRI TBSS ICVF BCC
Dermatopolymyositis
ivw
0.0031
0.304 (0.138−0.669)
IDP dMRI TBSS ICVF SCC
Dermatopolymyositis
Weighted median
0.0045
0.201 (0.066−0.608)
IDP dMRI TBSS ICVF SCC
Dermatopolymyositis
ivw
0.0418
0.328 (0.112−0.960)
IDP dMRI TBSS ICVF PLIC R
Dermatopolymyositis
ivw
0.0160
0.235 (0.072−0.763)
Reverse
Dermatopolymyositis
IDP dMRI TBSS FA SCC
Wald ratio
0.0136
1.043 (1.009−1.078)
Dermatopolymyositis
IDP dMRI TBSS L2 SCC
Wald ratio
0.0094
0.957 (0.926−0.989)
Dermatopolymyositis
IDP dMRI TBSS ICVF SCC
Wald ratio
0.0370
1.035 (1.002−1.070)
Dermatopolymyositis
IDP dMRI TBSS ICVF PLIC RWald ratio
0.0440
1.034 (1.001−1.067)
0.0
1.0
2.0
3.0
4.0
5.0
Odds ratio

D

exposure	outcome	method	pval	or (95% CI)
Forward				
IDP T1 FAST ROIs L putamen	MSKCTD	Wald ratio	0.0076	1.240 (1.059−1.452)
IDP T1 FAST ROIs R putamen	MSKCTD	Wald ratio	0.0076	1.242 (1.059−1.457)
IDP T1 FAST ROIs L ventral striatum	MSKCTD	Wald ratio	0.0076	1.265 (1.065−1.504)
IDP T1 FAST ROIs L cerebellum VIIIb	MSKCTD	Wald ratio	0.0127	1.445 (1.082−1.931)
IDP T1 FAST ROIs R cerebellum VIIIb	MSKCTD	Wald ratio	0.0076	1.511 (1.116−2.047)
IDP T1 FAST ROIs V cerebellum X	MSKCTD	Wald ratio	0.0076	1.406 (1.095−1.804)
IDP SWI T2star left pallidum	MSKCTD	Weighted median	0.0460	0.933 (0.872−0.999)
IDP SWI T2star left pallidum	MSKCTD	ivw	0.0318	0.935 (0.879−0.994)
volume Left−Lateral−Ventricle	MSKCTD	Weighted median	0.0204	0.856 (0.751−0.976)
volume Left−Lateral−Ventricle	MSKCTD	ivw	0.0060	0.855 (0.765−0.956)
volume Brain−Stem	MSKCTD	Wald ratio	0.0192	1.252 (1.037−1.510)
volume Left−Accumbens−area	MSKCTD	Wald ratio	0.0076	1.538 (1.122−2.110)
volume Right−Lateral−Ventricle	MSKCTD	ivw	0.0139	0.840 (0.731−0.965)
volume Right−Cerebellum−White−Matter	MSKCTD	Wald ratio	0.0238	1.189 (1.023−1.381)
DKTatlas lh pericalcarine area	MSKCTD	ivw	0.0322	0.898 (0.813−0.991)
DKTatlas rh parstriangularis area	MSKCTD	Wald ratio	0.0366	1.172 (1.010−1.361)
IDP T1 SIENAX CSF normalised volume	MSKCTD	ivw	0.0475	0.869 (0.756−0.998)
DKTatlas rh lateraloccipital thickness	MSKCTD	Wald ratio	0.0076	0.621 (0.438−0.881)
a2009s rh G cuneus thickness	MSKCTD	Wald ratio	0.0076	0.652 (0.477−0.893)
a2009s rh Pole occipital thickness	MSKCTD	Wald ratio	0.0076	0.657 (0.483−0.894)
IDP dMRI TBSS MO Anterior corona radiata R	MSKCTD	Wald ratio	0.0397	1.172 (1.007−1.362)
IDP dMRI TBSS MO Cingulum cingulate gyrus L	MSKCTD	ivw	0.0174	0.883 (0.797−0.978)
IDP T1 SIENAX CSF unnormalised volume	MSKCTD	Weighted median	0.0185	0.844 (0.732−0.972)
IDP T1 SIENAX CSF unnormalised volume	MSKCTD	ivw	0.0062	0.844 (0.748−0.953)
IDP dMRI TBSS L3 Anterior limb of internal capsule L	MSKCTD	Wald ratio	0.0227	1.331 (1.041−1.701)
IDP dMRI TBSS ICVF Cerebral peduncle R	MSKCTD	Weighted median	0.0222	1.113 (1.015−1.221)
IDP dMRI TBSS ICVF Cerebral peduncle R	MSKCTD	ivw	0.0025	1.128 (1.043−1.219)
IDP dMRI TBSS ICVF Cerebral peduncle L	MSKCTD	Wald ratio	0.0127	1.345 (1.065−1.698)
IDP dMRI TBSS OD Cerebral peduncle R	MSKCTD	Wald ratio	0.0275	1.322 (1.031−1.694)
IDP dMRI TBSS OD Cerebral peduncle L	MSKCTD	Wald ratio	0.0275	1.327 (1.032−1.705)
IDP dMRI TBSS OD Superior longitudinal fasciculus R	MSKCTD	Wald ratio	0.0128	0.769 (0.625−0.946)
IDP dMRI ProbtrackX FA str l	MSKCTD	Wald ratio	<0.001	1.274 (1.112−1.459)
IDP dMRI ProbtrackX MO atr l	MSKCTD	Wald ratio	0.0265	0.834 (0.711−0.979)
IDP dMRI ProbtrackX L1 slf r	MSKCTD	Wald ratio	0.0043	1.440 (1.121−1.851)

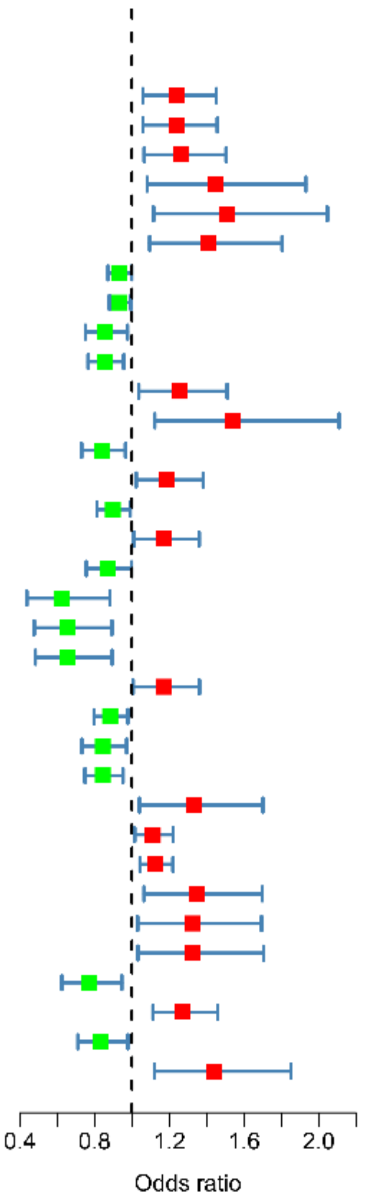

E

exposure	outcome	method	pval	or (95% CI)
Forward				
IDP dMRI TBSS FA SLF L	Drug−induced SLE	ivw	0.0270	0.099 (0.013−0.769)
volume Brain−Stem	Drug−induced SLE	Wald ratio	0.0144	109.171 (2.547−4679.124)
volume Right−Cerebellum−White−Matter	Drug−induced SLE	Wald ratio	0.0263	29.808 (1.492−595.351)
volume Right−Amygdala	Drug−induced SLE	Wald ratio	0.0060	0.001 (0.000−0.149)
DKTatlas lh lingual area	Drug−induced SLE	Wald ratio	0.0494	21.433 (1.008−455.816)
DKTatlas lh pericalcarine area	Drug−induced SLE	ivw	0.0419	7.878 (1.079−57.528)
a2009s lh S calcarine area	Drug−induced SLE	Wald ratio	0.0494	11.517 (1.006−131.820)
a2009s lh S subparietal area	Drug−induced SLE	Wald ratio	0.0494	0.064 (0.004−0.993)
DKTatlas rh cuneus area	Drug−induced SLE	Wald ratio	0.0494	29.221 (1.009−846.608)
DKTatlas rh lingual area	Drug−induced SLE	Wald ratio	0.0492	16.803 (1.009−279.722)
a2009s rh G cuneus area	Drug−induced SLE	Wald ratio	0.0494	19.642 (1.008−382.928)
a2009s rh G oc−temp med−Lingual area	Drug−induced SLE	Wald ratio	0.0492	21.380 (1.010−452.498)
a2009s rh Pole occipital area	Drug−induced SLE	Wald ratio	0.0494	20.433 (1.008−414.327)
a2009s rh S calcarine area	Drug−induced SLE	Wald ratio	0.0492	13.487 (1.009−180.349)
DKTatlas rh posteriorcingulate thickness	Drug−induced SLE	ivw	0.0097	32.654 (2.326−458.436)
IDP dMRI TBSS L3 Posterior corona radiata R	Drug−induced SLE	ivw	0.0053	18.111 (2.367−138.588)
IDP dMRI TBSS L3 Uncinate fasciculus L	Drug−induced SLE	ivw	0.0126	13.288 (1.742−101.353)
IDP dMRI TBSS ICVF RLIC R	Drug−induced SLE	Weighted median	0.0268	0.257 (0.077−0.855)
IDP dMRI TBSS ICVF RLIC R	Drug−induced SLE	ivw	0.0325	0.329 (0.119−0.912)
IDP dMRI TBSS ICVF RLIC L	Drug−induced SLE	Weighted median	0.0379	0.259 (0.073−0.928)
IDP dMRI TBSS ICVF Anterior corona radiata R	Drug−induced SLE	Weighted median	0.0472	0.241 (0.059−0.983)
IDP dMRI TBSS ICVF Anterior corona radiata L	Drug−induced SLE	Weighted median	0.0400	0.237 (0.060−0.936)
IDP dMRI TBSS ICVF Superior corona radiata R	Drug−induced SLE	Weighted median	0.0479	0.230 (0.054−0.987)
IDP dMRI TBSS ICVF Superior corona radiata L	Drug−induced SLE	Weighted median	0.0483	0.227 (0.052−0.989)
IDP dMRI TBSS ICVF SLF L	Drug−induced SLE	Weighted median	0.0339	0.246 (0.067−0.899)
IDP T1 FAST ROIs L intracalc cortex	Drug−induced SLE	Wald ratio	0.0494	11.570 (1.006−133.047)
IDP T1 FAST ROIs R intracalc cortex	Drug−induced SLE	Wald ratio	0.0494	10.057 (1.006−100.558)
IDP dMRI ProbtrackX ICVF ar l	Drug−induced SLE	Weighted median	0.0331	0.287 (0.091−0.905)
IDP dMRI ProbtrackX ICVF ar l	Drug−induced SLE	ivw	0.0384	0.319 (0.108−0.941)
IDP dMRI ProbtrackX ICVF ar r	Drug−induced SLE	Weighted median	0.0339	0.268 (0.079−0.905)
IDP dMRI ProbtrackX ICVF ar r	Drug−induced SLE	ivw	0.0395	0.305 (0.098−0.945)
IDP dMRI ProbtrackX ICVF atr l	Drug−induced SLE	Weighted median	0.0393	0.231 (0.057−0.931)
IDP dMRI ProbtrackX ICVF atr r	Drug−induced SLE	Weighted median	0.0382	0.233 (0.059−0.924)
IDP dMRI ProbtrackX ICVF cgh l	Drug−induced SLE	Weighted median	0.0472	0.276 (0.078−0.984)
IDP dMRI ProbtrackX ICVF cst l	Drug−induced SLE	Weighted median	0.0495	0.251 (0.063−0.997)
IDP dMRI ProbtrackX ICVF cst l	Drug−induced SLE	ivw	0.0426	0.284 (0.084−0.959)
IDP dMRI ProbtrackX ICVF fmi	Drug−induced SLE	Weighted median	0.0426	0.289 (0.087−0.959)
IDP dMRI ProbtrackX ICVF slf l	Drug−induced SLE	Weighted median	0.0355	0.263 (0.076−0.914)
IDP dMRI ProbtrackX ICVF str l	Drug−induced SLE	Weighted median	0.0412	0.237 (0.060−0.944)
IDP dMRI ProbtrackX ICVF str r	Drug−induced SLE	Weighted median	0.0456	0.225 (0.052−0.971)
IDP dMRI ProbtrackX ICVF unc l	Drug−induced SLE	ivw	0.0370	0.240 (0.063−0.918)

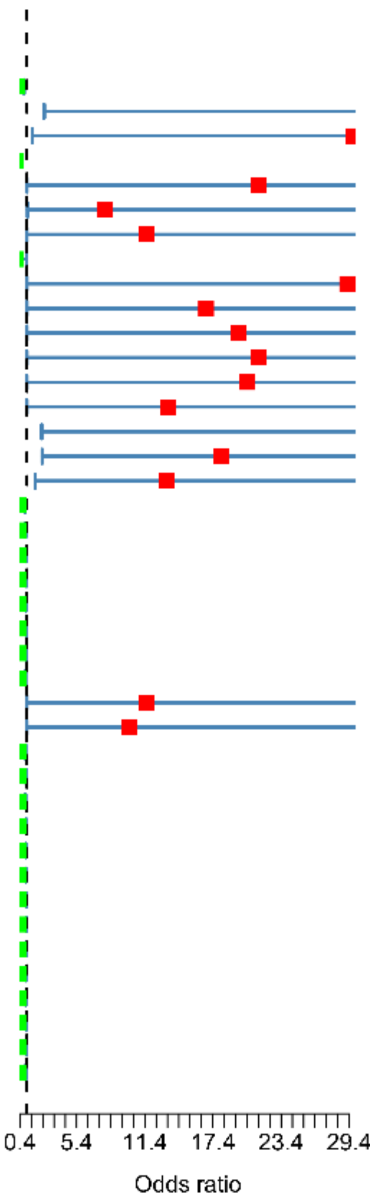

F

exposure	outcome	method	pval	or (95% CI)
Forward				
IDP T1 FAST ROIs V cerebellum VIIIa	Fibromyalgia	ivw	0.0361	2.818 (1.070−7.427)
IDP SWI T2star left pallidum	Fibromyalgia	ivw	0.0223	0.644 (0.442−0.939)
IDP dMRI TBSS FA Uncinate fasciculus L	Fibromyalgia	Wald ratio	0.0446	2.767 (1.025−7.470)
IDP SWI T2star left plus right pallidum	Fibromyalgia	ivw	0.0350	0.820 (0.682−0.986)
volume Right−Inf−Lat−Vent	Fibromyalgia	Wald ratio	0.0387	5.260 (1.090−25.376)
a2009s lh S collat transv ant area	Fibromyalgia	Wald ratio	0.0044	0.213 (0.074−0.618)
a2009s rh S collat transv ant area	Fibromyalgia	Wald ratio	0.0044	0.223 (0.079−0.626)
a2009s lh G insular short thickness	Fibromyalgia	Wald ratio	0.0330	4.457 (1.129−17.599)
IDP dMRI TBSS MO Medial lemniscus R	Fibromyalgia	Wald ratio	0.0423	0.284 (0.084−0.957)
IDP dMRI TBSS L2 Pontine crossing tract	Fibromyalgia	Wald ratio	0.0423	3.969 (1.049−15.014)
IDP dMRI TBSS ISOVF Fornix	Fibromyalgia	Wald ratio	0.0221	0.288 (0.099−0.836)
IDP dMRI ProbtrackX MO ml r	Fibromyalgia	Wald ratio	0.0082	11.980 (1.900−75.542)
Reverse				
Fibromyalgia	IDP T1 FAST ROIs V cerebellum VIIIa	ivw	0.0133	0.977 (0.959−0.995)

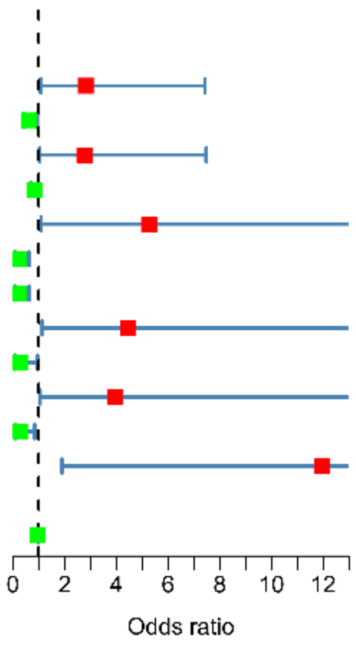

G

exposure	outcome	method	pval	or (95% CI)
Forward				
IDP T1 FAST ROIs R temp occ fusif cortex	Fibromyalgia related co−morbidities	Wald ratio	0.0429	1.005 (1.000−1.010)
IDP T1 FAST ROIs L cerebellum crus I	Fibromyalgia related co−morbidities	W−median	0.0266	0.997 (0.995−1.000)
IDP T1 FAST ROIs L cerebellum crus I	Fibromyalgia related co−morbidities	ivw	0.0119	0.997 (0.995−0.999)
IDP dMRI TBSS FA SCP R	Fibromyalgia related co−morbidities	ivw	0.0438	0.998 (0.996−1.000)
IDP dMRI TBSS FA SCP L	Fibromyalgia related co−morbidities	ivw	0.0433	0.998 (0.995−1.000)
IDP dMRI TBSS MD SCC	Fibromyalgia related co−morbidities	ivw	0.0226	0.997 (0.994−1.000)
IDP T1 FIRST left plus right putamen volume	Fibromyalgia related co−morbidities	Wald ratio	0.0408	1.003 (1.000−1.006)
volume Left−Cerebellum−Cortex	Fibromyalgia related co−morbidities	ivw	0.0439	0.997 (0.994−1.000)
a2009s lh G front inf−Opercular area	Fibromyalgia related co−morbidities	Wald ratio	0.0342	1.005 (1.000−1.010)
DKTatlas rh parstriangularis area	Fibromyalgia related co−morbidities	Wald ratio	0.0347	0.995 (0.991−1.000)
a2009s rh S circular insula ant thickness	Fibromyalgia related co−morbidities	Wald ratio	0.0316	1.005 (1.000−1.009)
Reverse				
Fibromyalgia related co−morbidities	IDP dMRI TBSS FA SCP R	MR Egger	0.0463	3896196.596 (2.877−5280
Fibromyalgia related co−morbidities	volume Left−Cerebellum−Cortex	ivw	0.0141	0.002 (0.000−0.270)

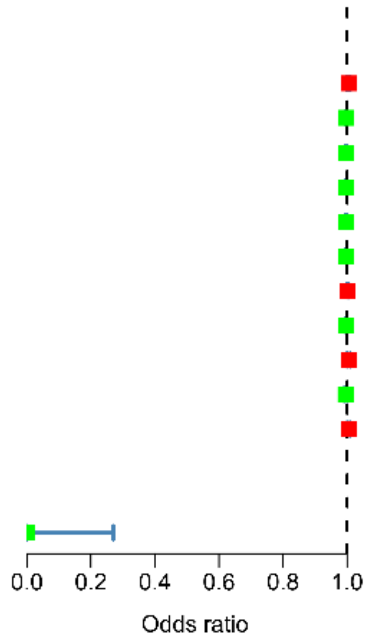

H

exposure	outcome	method	pval	or (95% CI)
Forward				
a2009s lh S collat transv ant area	GCA	Wald ratio	0.0436	0.251 (0.066−0.961)
a2009s rh S collat transv ant area	GCA	Wald ratio	0.0436	0.261 (0.071−0.962)
DKTatlas rh posteriorcingulate thickness	GCA	ivw	0.0079	0.224 (0.074−0.675)
IDP dMRI TBSS L1 PLIC L	GCA	Wald ratio	0.0264	0.218 (0.057−0.837)
IDP dMRI TBSS OD PLIC L	GCA	Wald ratio	0.0264	4.985 (1.207−20.590)

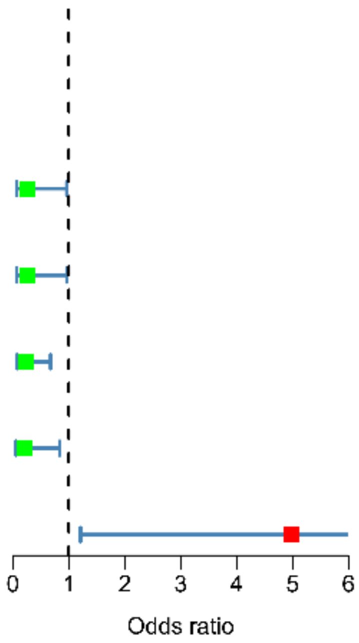

I

exposure	outcome	method	pval	or (95% CI)
Forward				
volume 4th−Ventricle	GCA−PMR	ivw	0.0216	3.141 (1.183−8.337)
volume CC Posterior	GCA−PMR	Wald ratio	0.0277	6.302 (1.224−32.444)
a2009s lh S collat transv ant area	GCA−PMR	Wald ratio	0.0161	0.179 (0.044−0.727)
a2009s rh S collat transv ant area	GCA−PMR	Wald ratio	0.0161	0.188 (0.048−0.733)
DKTatlas rh posteriorcingulate thickness	GCA−PMR	ivw	0.0235	0.264 (0.083−0.835)
IDP dMRI TBSS L1 PLIC R	GCA−PMR	Wald ratio	0.0444	0.207 (0.045−0.961)
IDP dMRI TBSS L1 PLIC L	GCA−PMR	Wald ratio	0.0383	0.227 (0.056−0.923)
IDP dMRI TBSS OD Pontine crossing tract	GCA−PMR	ivw	0.0325	3.275 (1.104−9.716)
IDP dMRI TBSS OD PLIC L	GCA−PMR	Wald ratio	0.0383	4.775 (1.088−20.966)

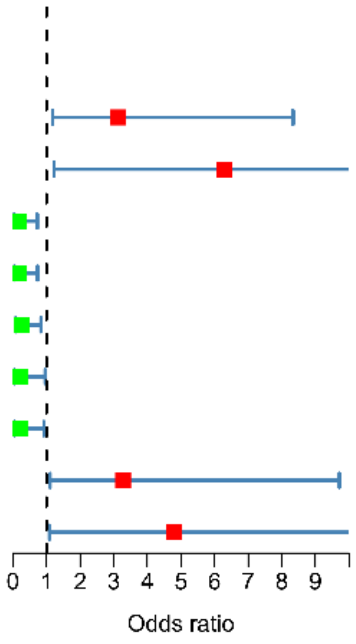

J

exposure	outcome	method	pval	or (95% CI)
Forward				
IDP T1 FAST ROIs L putamen	multiple sclerosis	Wald ratio	0.0027	1.359 (1.112−1.661)
IDP T1 FAST ROIs R putamen	multiple sclerosis	Wald ratio	0.0027	1.363 (1.113−1.668)
IDP T1 FAST ROIs L ventral striatum	multiple sclerosis	Wald ratio	0.0027	1.399 (1.123−1.742)
IDP T1 FAST ROIs R ventral striatum	multiple sclerosis	ivw	0.0016	1.370 (1.126−1.667)
IDP T1 FAST ROIs L cerebellum crus I	multiple sclerosis	W−median	0.0498	0.720 (0.519−1.000)
IDP T1 FAST ROIs V cerebellum VIIIa	multiple sclerosis	Wald ratio	0.0019	2.612 (1.423−4.795)
IDP T1 FAST ROIs L cerebellum VIIIb	multiple sclerosis	Wald ratio	0.0024	1.866 (1.246−2.793)
IDP T1 FAST ROIs V cerebellum VIIIb	multiple sclerosis	Wald ratio	0.0024	2.149 (1.310−3.524)
IDP T1 FAST ROIs R cerebellum VIIIb	multiple sclerosis	Wald ratio	0.0027	1.802 (1.226−2.649)
IDP T1 FAST ROIs L cerebellum IX	multiple sclerosis	Wald ratio	0.0024	2.033 (1.285−3.216)
IDP T1 FAST ROIs V cerebellum X	multiple sclerosis	Wald ratio	0.0027	1.625 (1.183−2.232)
IDP SWI T2star left caudate	multiple sclerosis	W−median	0.0158	0.816 (0.691−0.962)
IDP SWI T2star left caudate	multiple sclerosis	ivw	<0.001	0.785 (0.681−0.905)
IDP SWI T2star right caudate	multiple sclerosis	W−median	0.0244	0.828 (0.703−0.976)
IDP SWI T2star right caudate	multiple sclerosis	ivw	0.0434	0.816 (0.670−0.994)
IDP dMRI TBSS FA PLIC R	multiple sclerosis	Wald ratio	0.0043	1.652 (1.170−2.333)
IDP SWI T2star left plus right caudate	multiple sclerosis	W−median	0.0472	0.924 (0.855−0.999)
IDP SWI T2star left plus right caudate	multiple sclerosis	ivw	0.0245	0.874 (0.778−0.983)
volume Left−Lateral−Ventricle	multiple sclerosis	W−median	0.0075	1.589 (1.132−2.231)
volume Left−Lateral−Ventricle	multiple sclerosis	ivw	0.0388	1.454 (1.019−2.074)
volume 3rd−Ventricle	multiple sclerosis	Wald ratio	0.0422	1.772 (1.020−3.078)
volume CSF	multiple sclerosis	W−median	0.0382	1.478 (1.022−2.138)
volume Left−Accumbens−area	multiple sclerosis	Wald ratio	0.0027	1.848 (1.237−2.762)
IDP dMRI TBSS MD SFOF L	multiple sclerosis	Wald ratio	0.0075	1.776 (1.165−2.707)
IDP T1 SIENAX CSF normalised volume	multiple sclerosis	ivw	<0.001	1.704 (1.263−2.297)
DKTatlas rh lateraloccipital thickness	multiple sclerosis	Wald ratio	0.0027	0.507 (0.325−0.791)
a2009s rh G cuneus thickness	multiple sclerosis	Wald ratio	0.0027	0.544 (0.365−0.810)
a2009s rh Pole occipital thickness	multiple sclerosis	Wald ratio	0.0027	0.549 (0.371−0.813)
IDP T1 SIENAX CSF unnormalised volume	multiple sclerosis	W−median	0.0047	1.662 (1.168−2.365)
IDP T1 SIENAX CSF unnormalised volume	multiple sclerosis	ivw	0.0289	1.511 (1.043−2.189)
IDP dMRI TBSS L3 ALIC L	multiple sclerosis	Wald ratio	0.0287	0.639 (0.428−0.955)
IDP dMRI TBSS ICVF Fornix	multiple sclerosis	Wald ratio	0.0075	0.619 (0.436−0.880)
IDP dMRI TBSS OD SLF R	multiple sclerosis	Wald ratio	0.0063	1.564 (1.135−2.155)
IDP dMRI ProbtrackX OD str l	multiple sclerosis	ivw	<0.001	0.588 (0.454−0.760)
Reverse				
multiple sclerosis	IDP SWI T2star right caudate	W−mode	0.0377	0.958 (0.922−0.997)

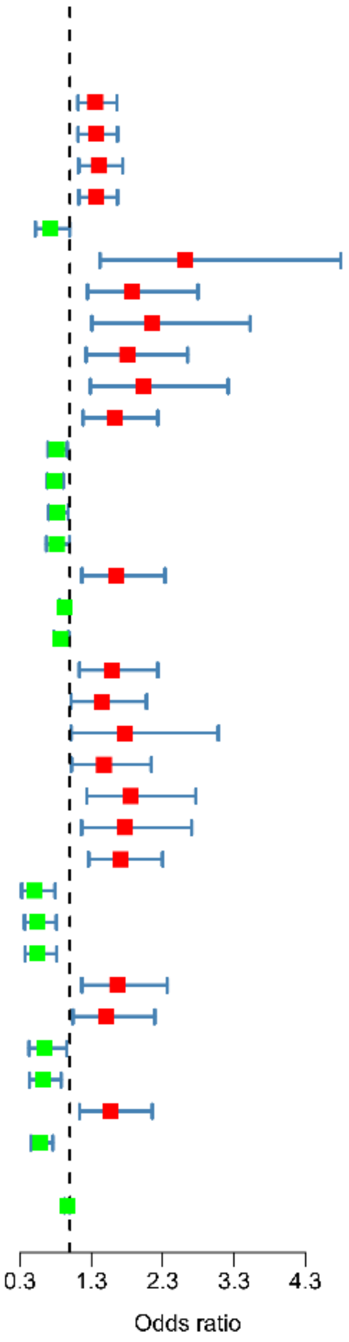

K

exposure	outcome	method	pval	or (95% CI)
Forward				
IDP T1 FAST ROIs L thalamus	Polyarteritis nodosa	ivw	0.0313	23.915 (1.330−429.902)
IDP T1 FAST ROIs R hippocampus	Polyarteritis nodosa	Wald ratio	0.0343	0.018 (0.000−0.744)
IDP T1 FIRST right putamen volume	Polyarteritis nodosa	Wald ratio	0.0432	0.020 (0.000−0.888)
IDP SWI T2star right caudate	Polyarteritis nodosa	MR Egger	0.0458	12468.318 (46.003−33793
IDP dMRI TBSS MD ALIC L	Polyarteritis nodosa	Wald ratio	0.0075	109.375 (3.500−3417.996)
IDP SWI T2star left plus right caudate	Polyarteritis nodosa	MR Egger	0.0282	84.863 (4.921−1463.359)
volume Right−Cerebellum−Cortex	Polyarteritis nodosa	W−median	0.0125	17.417 (1.849−164.051)
volume Right−Cerebellum−Cortex	Polyarteritis nodosa	ivw	0.0029	17.728 (2.678−117.368)
volume Right−Putamen	Polyarteritis nodosa	Wald ratio	0.0432	0.031 (0.001−0.899)
volume Right−Hippocampus	Polyarteritis nodosa	Wald ratio	0.0343	0.011 (0.000−0.718)
DKTatlas lh pericalcarine area	Polyarteritis nodosa	ivw	0.0107	17.682 (1.948−160.467)
a2009s lh S collat transv ant area	Polyarteritis nodosa	Wald ratio	0.0151	49.464 (2.125−1151.424)
IDP dMRI TBSS MD Uncinate fasciculus R	Polyarteritis nodosa	Wald ratio	0.0075	133.338 (3.688−4820.994)
a2009s rh S collat transv ant area	Polyarteritis nodosa	Wald ratio	0.0151	44.401 (2.081−947.316)
a2009s rh S orbital med−olfact area	Polyarteritis nodosa	Wald ratio	0.0431	0.015 (0.000−0.878)
IDP dMRI TBSS L1 ALIC L	Polyarteritis nodosa	Wald ratio	0.0075	178.349 (3.988−7976.465)

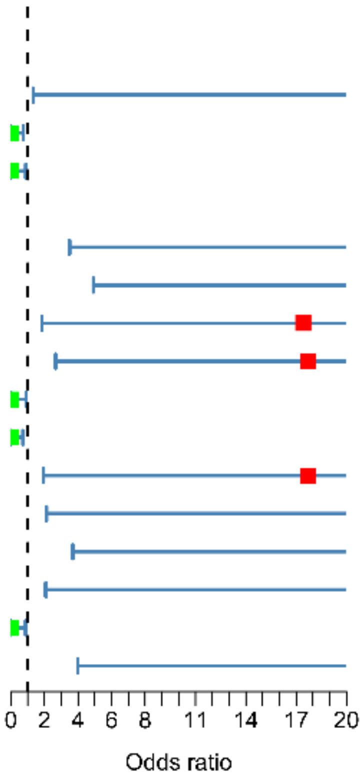

L

exposure	outcome	method	pval	or (95% CI)
Forward				
IDP T1 FAST ROIs R occ pole	Polymyositis	ivw	0.0388	24.583 (1.179−512.552)
IDP T1 FAST ROIs L cerebellum VI	Polymyositis	Wald ratio	0.0230	0.013 (0.000−0.548)
IDP dMRI TBSS FA PLIC R	Polymyositis	Wald ratio	0.0019	334.104 (8.591−12993.203)
IDP dMRI TBSS FA RPIC L	Polymyositis	Wald ratio	0.0241	90.658 (1.804−4555.849)
IDP dMRI TBSS FA PCR L	Polymyositis	Wald ratio	0.0241	183.382 (1.978−16998.801)
IDP dMRI TBSS FA Sagittal stratum L	Polymyositis	Wald ratio	0.0266	89.375 (1.684−4743.529)
IDP dMRI TBSS MD ICP L	Polymyositis	Wald ratio	0.0241	0.008 (0.000−0.530)
volume Left−Inf−Lat−Vent	Polymyositis	Wald ratio	0.0083	113.524 (3.389−3802.728)
volume Right−VentralDC	Polymyositis	Wald ratio	0.0317	827.507 (1.800−380412.190)
a2009s rh S orbital med−olfact area	Polymyositis	Wald ratio	0.0324	0.003 (0.000−0.608)
a2009s rh S oc sup&transversal thickness	Polymyositis	Wald ratio	0.0401	0.016 (0.000−0.829)
IDP dMRI TBSS L1 GCC	Polymyositis	Wald ratio	0.0266	0.005 (0.000−0.546)
IDP dMRI TBSS L1 PLIC R	Polymyositis	Wald ratio	0.0496	0.011 (0.000−0.993)
IDP dMRI TBSS L1 Cingulum hippocampus L	Polymyositis	Wald ratio	0.0266	0.007 (0.000−0.560)
IDP dMRI TBSS L1 Uncinate fasciculus R	Polymyositis	Wald ratio	0.0241	0.003 (0.000−0.475)
IDP dMRI TBSS L2 RPIC L	Polymyositis	Wald ratio	0.0241	0.015 (0.000−0.579)
IDP dMRI TBSS L2 Sagittal stratum L	Polymyositis	Wald ratio	0.0266	0.018 (0.001−0.628)
IDP dMRI TBSS L2 CCG L	Polymyositis	Wald ratio	0.0266	0.010 (0.000−0.585)
IDP dMRI TBSS L3 MCP	Polymyositis	Wald ratio	0.0241	0.005 (0.000−0.500)
IDP dMRI TBSS L3 RPIC L	Polymyositis	Wald ratio	0.0241	0.017 (0.001−0.589)
IDP dMRI TBSS L3 SCR R	Polymyositis	Wald ratio	0.0266	0.010 (0.000−0.586)
IDP dMRI TBSS L3 SCR L	Polymyositis	Wald ratio	0.0266	0.009 (0.000−0.577)
IDP dMRI TBSS L3 PCR L	Polymyositis	Wald ratio	0.0241	0.011 (0.000−0.555)
IDP dMRI TBSS ICVF Medial lemniscus R	Polymyositis	ivw	0.0454	10.857 (1.050−112.285)
IDP dMRI TBSS ICVF CCG L	Polymyositis	ivw	0.0135	10.252 (1.618−64.957)
IDP dMRI TBSS ICVF FC+ST L	Polymyositis	Wald ratio	0.0266	16.195 (1.381−189.872)
IDP dMRI TBSS ICVF SFOF L	Polymyositis	Wald ratio	0.0266	70.494 (1.638−3033.416)
IDP dMRI TBSS ICVF Tapetum R	Polymyositis	Wald ratio	0.0241	73.136 (1.754−3049.495)
IDP dMRI TBSS ICVF Tapetum L	Polymyositis	Wald ratio	0.0241	86.527 (1.793−4175.541)
IDP dMRI TBSS ISOVF Fornix	Polymyositis	Wald ratio	0.0498	30.823 (1.004−946.731)
IDP dMRI ProbtrackX FA atr r	Polymyositis	Wald ratio	0.0241	70.105 (1.744−2817.580)
IDP dMRI ProbtrackX FA fma	Polymyositis	Wald ratio	0.0241	104.529 (1.838−5944.739)
IDP dMRI ProbtrackX FA ptr l	Polymyositis	Wald ratio	0.0266	37.252 (1.521−912.147)
IDP dMRI ProbtrackX FA slf r	Polymyositis	Wald ratio	0.0241	84.563 (1.788−4000.154)
IDP dMRI ProbtrackX L1 fmi	Polymyositis	Wald ratio	0.0266	0.006 (0.000−0.550)
IDP dMRI ProbtrackX L1 ifo l	Polymyositis	Wald ratio	0.0266	0.008 (0.000−0.575)
IDP dMRI ProbtrackX L1 ifo r	Polymyositis	Wald ratio	0.0266	0.007 (0.000−0.564)
IDP dMRI ProbtrackX L1 ilf r	Polymyositis	Wald ratio	0.0266	0.008 (0.000−0.573)
IDP dMRI ProbtrackX L1 str r	Polymyositis	Wald ratio	0.0349	80.821 (1.366−4782.691)
IDP dMRI ProbtrackX L2 cgc l	Polymyositis	Wald ratio	0.0266	0.012 (0.000−0.596)
IDP dMRI ProbtrackX L2 cgc r	Polymyositis	Wald ratio	0.0266	0.021 (0.001−0.639)
IDP dMRI ProbtrackX L3 cgc l	Polymyositis	Wald ratio	0.0241	0.015 (0.000−0.579)
IDP dMRI ProbtrackX L3 cgc r	Polymyositis	Wald ratio	0.0241	0.018 (0.001−0.591)
IDP dMRI ProbtrackX L3 str r	Polymyositis	Wald ratio	0.0266	0.004 (0.000−0.531)
IDP dMRI ProbtrackX ICVF cgc l	Polymyositis	ivw	0.0141	13.024 (1.679−101.008)
IDP dMRI ProbtrackX ICVF cgc r	Polymyositis	Wald ratio	0.0266	14.667 (1.365−157.537)

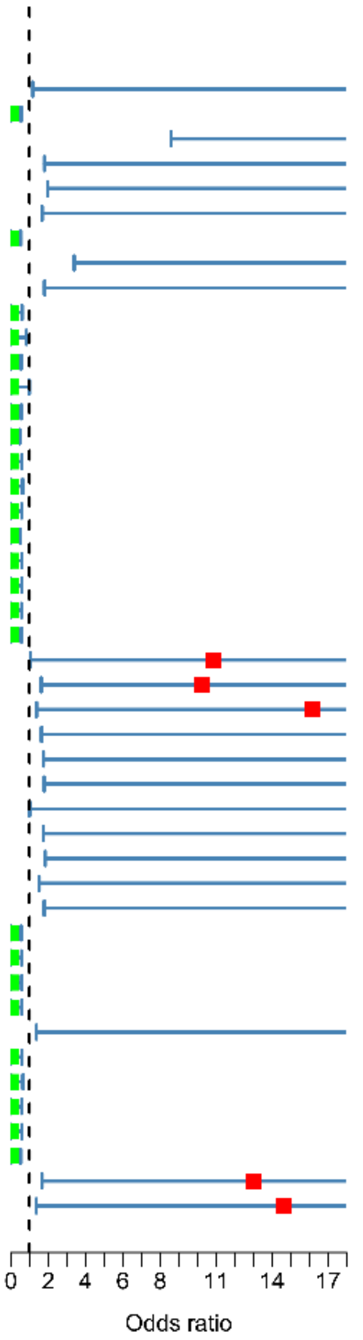

M

exposure	outcome	method	pval	or (95% CI)
Forward				
IDP T1 FAST ROIs R occ pole	Sjogren's syndrome	Wald ratio	0.0059	15.918 (2.219−114.187)
volume Right−Inf−Lat−Vent	Sjogren's syndrome	Wald ratio	0.0185	6.143 (1.356−27.831)
DKTatlas lh supramarginal area	Sjogren's syndrome	Wald ratio	0.0401	0.138 (0.021−0.914)
a2009s lh G pariet inf−Supramar area	Sjogren's syndrome	Wald ratio	0.0401	0.165 (0.029−0.921)
a2009s lh S collat transv ant area	Sjogren's syndrome	Wald ratio	0.0345	5.448 (1.131−26.231)
DKTatlas rh lateraloccipital area	Sjogren's syndrome	ivw	0.0086	6.421 (1.604−25.702)
a2009s rh S collat transv ant area	Sjogren's syndrome	Wald ratio	0.0345	5.198 (1.127−23.963)
IDP dMRI TBSS L1 SLF R	Sjogren's syndrome	ivw	0.0322	0.169 (0.033−0.859)
IDP dMRI TBSS L3 ALIC R	Sjogren's syndrome	Wald ratio	0.0057	0.084 (0.014−0.486)
IDP dMRI TBSS L3 ALIC L	Sjogren's syndrome	Wald ratio	0.0048	0.071 (0.011−0.447)
IDP dMRI TBSS OD PCR R	Sjogren's syndrome	Wald ratio	0.0031	14.689 (2.476−87.154)
IDP dMRI TBSS OD SLF R	Sjogren's syndrome	Wald ratio	<0.001	19.471 (3.492−108.555)
IDP dMRI ProbtrackX MO ml r	Sjogren's syndrome	Wald ratio	0.0374	3.858 (1.082−13.753)
Reverse				
Sjogren's syndrome	DKTatlas rh lateraloccipital area	Wald ratio	0.0017	0.924 (0.880−0.971)

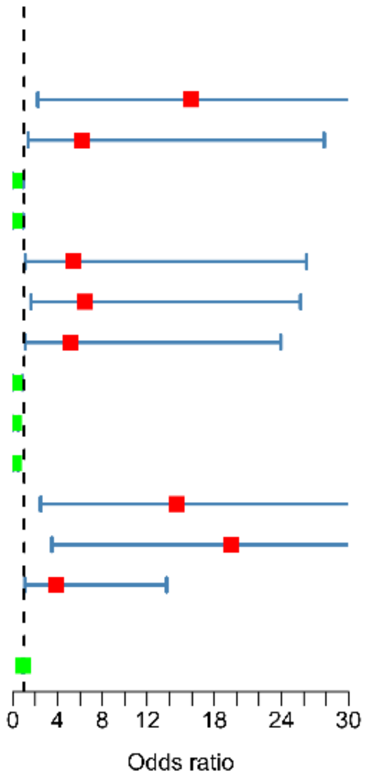

N

exposure	outcome	method	pval	or (95% CI)
Forward				
IDP SWI T2star left putamen	SCTD	W−median	0.0128	1.247 (1.048−1.484)
IDP SWI T2star left putamen	SCTD	ivw	0.0037	1.231 (1.070−1.415)
IDP SWI T2star left plus right thalamus	SCTD	W−median	0.0497	1.207 (1.000−1.457)
IDP SWI T2star left plus right thalamus	SCTD	ivw	0.0205	1.202 (1.029−1.405)
IDP SWI T2star left plus right caudate	SCTD	ivw	0.0223	1.089 (1.012−1.172)
IDP SWI T2star left plus right putamen	SCTD	W−median	0.0114	1.110 (1.024−1.204)
IDP SWI T2star left plus right putamen	SCTD	ivw	0.0013	1.109 (1.041−1.181)
volume Right−Cerebellum−Cortex	SCTD	W−median	0.0330	1.353 (1.025−1.788)
IDP dMRI ProbtrackX FA ml l	SCTD	Wald ratio	0.0444	1.632 (1.012−2.631)

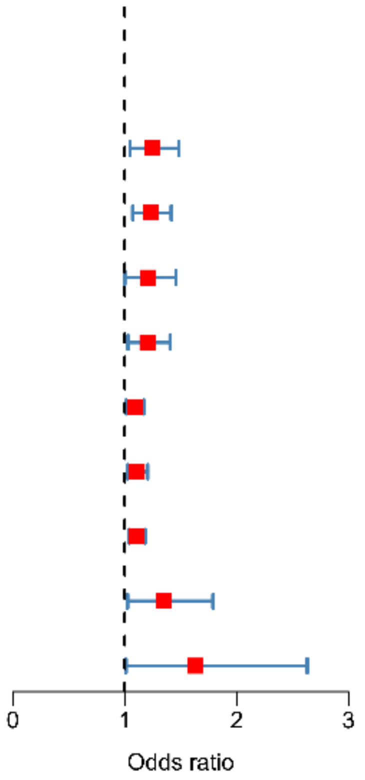

O

exposure	outcome	method	pval	or (95% CI)
Forward				
IDP T1 FAST ROIs L cerebellum crus I	Systemic lupus erythematosus	ivw	0.0316	0.543 (0.311−0.948)
IDP SWI T2star left putamen	Systemic lupus erythematosus	ivw	0.0093	1.522 (1.109−2.089)
IDP dMRI TBSS FA CCG R	Systemic lupus erythematosus	ivw	0.0458	0.386 (0.152−0.983)
IDP dMRI TBSS MD External capsule L	Systemic lupus erythematosus	ivw	0.0030	2.795 (1.418−5.509)
IDP dMRI TBSS MD CCG R	Systemic lupus erythematosus	ivw	0.0307	2.006 (1.067−3.772)
IDP dMRI TBSS MD CCG L	Systemic lupus erythematosus	ivw	0.0298	1.942 (1.067−3.535)
IDP dMRI TBSS MD Uncinate fasciculus L	Systemic lupus erythematosus	ivw	0.0012	2.737 (1.489−5.032)
IDP dMRI TBSS L3 Uncinate fasciculus L	Systemic lupus erythematosus	ivw	<0.001	3.715 (1.819−7.589)
IDP dMRI TBSS ICVF ALIC L	Systemic lupus erythematosus	ivw	0.0039	0.552 (0.368−0.826)
IDP dMRI TBSS ICVF PLIC L	Systemic lupus erythematosus	ivw	0.0223	0.540 (0.318−0.916)
IDP dMRI TBSS ICVF RPIC L	Systemic lupus erythematosus	ivw	0.0148	0.589 (0.385−0.901)
IDP dMRI TBSS ICVF ACR R	Systemic lupus erythematosus	ivw	0.0269	0.573 (0.350−0.938)
IDP dMRI TBSS ICVF ACR L	Systemic lupus erythematosus	ivw	0.0163	0.561 (0.350−0.899)
IDP dMRI TBSS ICVF SCR R	Systemic lupus erythematosus	ivw	0.0380	0.571 (0.337−0.969)
IDP dMRI TBSS ICVF SCR L	Systemic lupus erythematosus	ivw	0.0303	0.563 (0.334−0.947)
IDP dMRI TBSS ICVF PCR L	Systemic lupus erythematosus	ivw	0.0485	0.577 (0.334−0.996)
IDP dMRI TBSS ICVF External capsule R	Systemic lupus erythematosus	ivw	0.0020	0.543 (0.369−0.801)
IDP dMRI TBSS ICVF External capsule L	Systemic lupus erythematosus	ivw	<0.001	0.495 (0.326−0.752)
IDP dMRI TBSS ICVF CH R	Systemic lupus erythematosus	ivw	0.0136	0.547 (0.339−0.883)
IDP dMRI TBSS ICVF CH L	Systemic lupus erythematosus	ivw	0.0075	0.589 (0.399−0.868)
IDP dMRI TBSS ICVF SLF L	Systemic lupus erythematosus	ivw	0.0448	0.601 (0.366−0.988)
IDP dMRI TBSS ICVF SFOF R	Systemic lupus erythematosus	ivw	0.0443	0.407 (0.170−0.977)
IDP dMRI ProbtrackX MD cgc l	Systemic lupus erythematosus	ivw	0.0171	1.983 (1.130−3.480)
IDP dMRI ProbtrackX L1 unc l	Systemic lupus erythematosus	ivw	0.0015	2.903 (1.504−5.605)
IDP dMRI ProbtrackX L3 fmi	Systemic lupus erythematosus	ivw	0.0444	1.852 (1.015−3.378)
IDP dMRI ProbtrackX ICVF ar l	Systemic lupus erythematosus	ivw	0.0032	0.581 (0.406−0.834)
IDP dMRI ProbtrackX ICVF ar r	Systemic lupus erythematosus	ivw	0.0035	0.571 (0.392−0.832)
IDP dMRI ProbtrackX ICVF atr l	Systemic lupus erythematosus	ivw	0.0060	0.543 (0.351−0.839)
IDP dMRI ProbtrackX ICVF atr r	Systemic lupus erythematosus	ivw	0.0077	0.544 (0.348−0.851)
IDP dMRI ProbtrackX ICVF cgh l	Systemic lupus erythematosus	ivw	0.0052	0.583 (0.399−0.851)
IDP dMRI ProbtrackX ICVF cst l	Systemic lupus erythematosus	ivw	0.0087	0.557 (0.360−0.863)
IDP dMRI ProbtrackX ICVF cst r	Systemic lupus erythematosus	ivw	0.0089	0.581 (0.387−0.873)
IDP dMRI ProbtrackX ICVF slf l	Systemic lupus erythematosus	ivw	0.0265	0.599 (0.381−0.942)
IDP dMRI ProbtrackX ICVF str l	Systemic lupus erythematosus	ivw	0.0206	0.556 (0.338−0.914)
IDP dMRI ProbtrackX ICVF str r	Systemic lupus erythematosus	ivw	0.0258	0.554 (0.330−0.931)
IDP dMRI ProbtrackX ICVF unc l	Systemic lupus erythematosus	ivw	0.0013	0.481 (0.308−0.752)
Reverse				
Systemic lupus erythematosus	IDP dMRI TBSS MD Uncinate fasciculus L	W−median	0.0224	0.952 (0.912−0.993)
Systemic lupus erythematosus	IDP dMRI TBSS ICVF PCR L	W−median	0.0472	1.053 (1.001−1.109)
Systemic lupus erythematosus	IDP dMRI TBSS ICVF PCR L	ivw	0.0186	1.054 (1.009−1.102)
Systemic lupus erythematosus	IDP dMRI ProbtrackX L1 unc l	ivw	0.0398	0.962 (0.927−0.998)

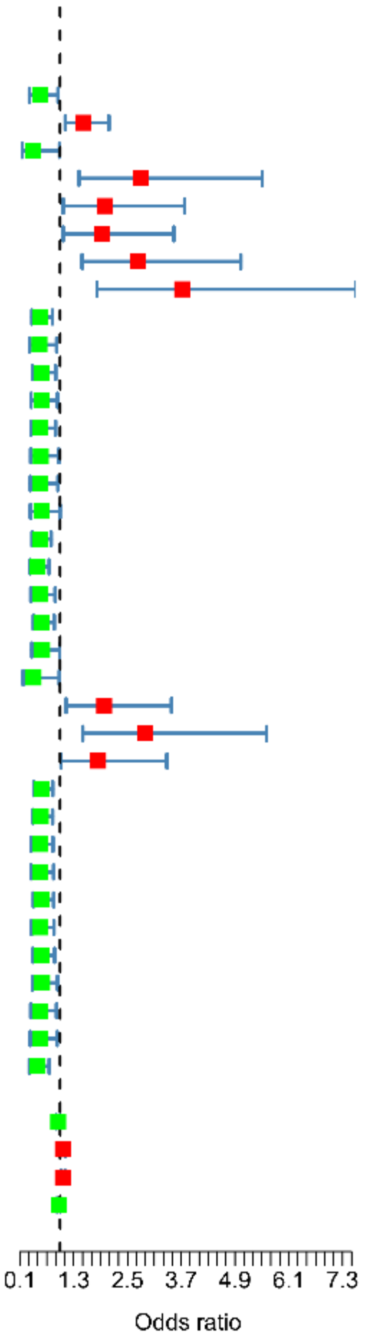

P

exposure	outcome	method	pval	or (95% CI)
Forward				
IDP SWI T2star left caudate	Systemic sclerosis	W−median	0.0260	2.544 (1.118−5.786)
IDP SWI T2star left caudate	Systemic sclerosis	ivw	0.0248	2.537 (1.125−5.718)
IDP SWI T2star right caudate	Systemic sclerosis	ivw	0.0230	2.200 (1.115−4.340)
IDP SWI T2star left putamen	Systemic sclerosis	W−median	0.0435	2.358 (1.025−5.422)
IDP SWI T2star left putamen	Systemic sclerosis	ivw	0.0066	2.309 (1.263−4.222)
IDP SWI T2star right putamen	Systemic sclerosis	W−median	0.0387	2.167 (1.041−4.512)
IDP SWI T2star right putamen	Systemic sclerosis	ivw	0.0077	2.078 (1.214−3.559)
IDP dMRI TBSS FA PLIC R	Systemic sclerosis	Wald ratio	0.0355	0.181 (0.037−0.891)
IDP dMRI TBSS MD Corticospinal tract R	Systemic sclerosis	Wald ratio	0.0316	0.147 (0.026−0.844)
IDP SWI T2star left plus right caudate	Systemic sclerosis	ivw	0.0300	1.483 (1.039−2.116)
IDP SWI T2star left plus right putamen	Systemic sclerosis	ivw	0.0208	1.388 (1.051−1.832)
DKTatlas rh parstriangularis thickness	Systemic sclerosis	Wald ratio	<0.001	0.015 (0.002−0.135)
IDP dMRI TBSS L2 Corticospinal tract R	Systemic sclerosis	Wald ratio	0.0316	0.144 (0.025−0.843)

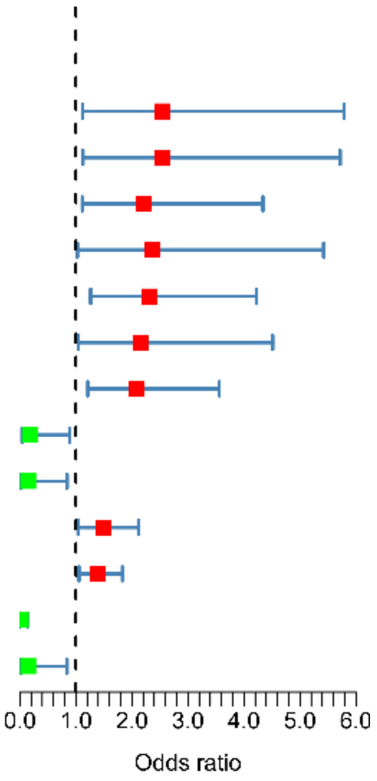

Q

exposure	outcome	method	pval	or (95% CI)
Forward				
DKTatlas lh superiorparietal area	TMD−MP−FM	Wald ratio	0.0247	2.101 (1.099−4.017)
a2009s lh G parietal sup area	TMD−MP−FM	Wald ratio	0.0243	2.138 (1.104−4.140)
a2009s lh G postcentral area	TMD−MP−FM	Wald ratio	0.0242	0.447 (0.222−0.900)
a2009s lh S intrapariet&P trans area	TMD−MP−FM	Wald ratio	0.0243	2.141 (1.104−4.152)
IDP dMRI TBSS L2 SCC	TMD−MP−FM	ivw	0.0457	0.741 (0.552−0.994)
IDP dMRI TBSS ICVF BCC	TMD−MP−FM	ivw	0.0248	1.305 (1.034−1.645)
IDP dMRI TBSS ICVF SCC	TMD−MP−FM	ivw	0.0476	1.302 (1.003−1.691)
IDP dMRI TBSS OD Pontine crossing tract	TMD−MP−FM	W−median	0.0494	1.663 (1.001−2.761)
IDP dMRI TBSS OD Pontine crossing tract	TMD−MP−FM	ivw	0.0185	1.641 (1.087−2.479)
IDP dMRI TBSS ISOVF External capsule R	TMD−MP−FM	Wald ratio	0.0353	0.504 (0.267−0.954)
IDP dMRI ProbtrackX FA fmi	TMD−MP−FM	ivw	0.0450	1.299 (1.006−1.677)
Reverse				
TMD−MP−FM	IDP dMRI TBSS ICVF SCC	W−median	0.0431	1.058 (1.002−1.118)

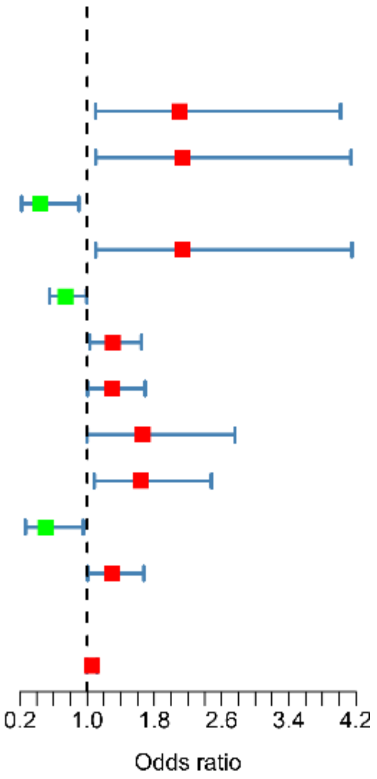

R

exposure	outcome	method	pval	or (95% CI)
Forward				
IDP dMRI TBSS FA SCP R	Vasculitis limited to skin	ivw	<0.001	0.125 (0.047−0.332)
IDP dMRI TBSS FA SCP L	Vasculitis limited to skin	ivw	<0.001	0.107 (0.037−0.304)
IDP dMRI TBSS MD SCP R	Vasculitis limited to skin	Wald ratio	0.0018	21.706 (3.141−149.98
IDP dMRI TBSS MD SCP L	Vasculitis limited to skin	Wald ratio	<0.001	40.920 (5.015−333.87
volume Left−Lateral−Ventricle	Vasculitis limited to skin	ivw	0.0341	6.744 (1.153−39.433)
volume CSF	Vasculitis limited to skin	W−median	0.0374	0.208 (0.047−0.912)
volume CSF	Vasculitis limited to skin	ivw	0.0306	0.260 (0.077−0.882)
IDP dMRI TBSS MD SFOF L	Vasculitis limited to skin	Wald ratio	0.0014	28.492 (3.629−223.70
IDP T1 SIENAX CSF normalised volume	Vasculitis limited to skin	ivw	0.0044	14.328 (2.295−89.466
IDP dMRI TBSS L1 PLIC R	Vasculitis limited to skin	Wald ratio	0.0443	0.151 (0.024−0.953)
IDP dMRI TBSS L1 PLIC L	Vasculitis limited to skin	Wald ratio	0.0479	0.183 (0.034−0.984)
IDP T1 SIENAX CSF unnormalised volume	Vasculitis limited to skin	ivw	0.0237	8.281 (1.327−51.685)
IDP dMRI TBSS L2 SCP R	Vasculitis limited to skin	ivw	<0.001	8.711 (3.154−24.060)
IDP dMRI TBSS L2 SCP L	Vasculitis limited to skin	Wald ratio	<0.001	11.286 (2.866−44.441
IDP dMRI TBSS L3 SCP R	Vasculitis limited to skin	ivw	<0.001	8.566 (3.131−23.433)
IDP dMRI TBSS L3 SCP L	Vasculitis limited to skin	Wald ratio	<0.001	9.833 (2.699−35.821)
IDP dMRI TBSS ICVF Fornix	Vasculitis limited to skin	Wald ratio	0.0014	0.061 (0.011−0.341)
IDP dMRI TBSS ICVF PTR L	Vasculitis limited to skin	ivw	0.0235	3.999 (1.206−13.262)
IDP dMRI TBSS ICVF Sagittal stratum L	Vasculitis limited to skin	ivw	0.0154	4.001 (1.303−12.288)
IDP dMRI TBSS OD PLIC L	Vasculitis limited to skin	Wald ratio	0.0479	6.011 (1.017−35.523)
IDP dMRI ProbtrackX OD str l	Vasculitis limited to skin	ivw	0.0134	0.159 (0.037−0.683)
Reverse				
Vasculitis limited to skin	IDP dMRI TBSS ICVF Sagittal stratum L	MR Egger	0.0180	1.034 (1.011−1.057)
Vasculitis limited to skin	IDP dMRI TBSS ICVF Sagittal stratum L	W−median	0.0432	1.022 (1.001−1.043)

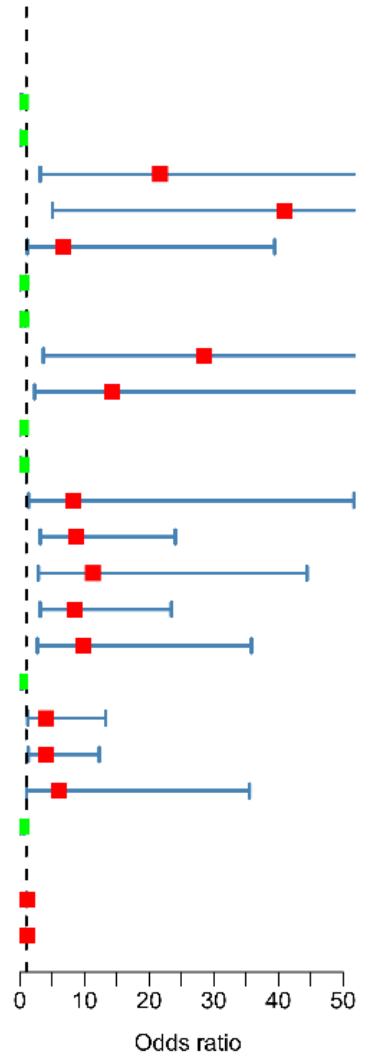

Figure S2. Causality of MR in 18 AIDs

(A－R) Behcet’s disease (A), connective tissue disorder (B), dermatopolymyositis (C), diseases of the musculoskeletal system and connective tissue (D), drug-induced systemic lupus erythematosus (E), fibromyalgia (F), fibromyalgia-related co-morbidities (G), giant cell arteritis (H), giant cell arteritis with polymyalgia rheumatica (I), multiple sclerosis (J), polyarteritis nodosa (K), polymyositis (L), Sjögren’s syndrome (M), systemic connective tissue disorders (N), systemic lupus erythematosus (O), systemic sclerosis (P), temporomandibular disorder (TMD) muscular pain linked with fibromyalgia (Q), and vasculitis limited to skin (R).

Each panel shows significant causal associations identified by forward and reverse MR analyses using four complementary methods: inverse-variance weighted (IVW), weighted median, MR-Egger, and Wald ratio.

In forward MR, the IVW estimates represent the odds ratio (OR) for the effect of a one-standard-deviation (SD) increase in mean IDP value on the risk of AIDs; in reverse MR, the OR represents the effect of a one-SD increase in AID liability on the mean IDP level.

Error bars denote 95 % confidence intervals (CIs). All tests were two-sided and adjusted for multiple comparisons using the Bonferroni correction ($P < 0.05 / 87$ for forward MR; $P < 0.05 / 3$ for reverse MR).

A

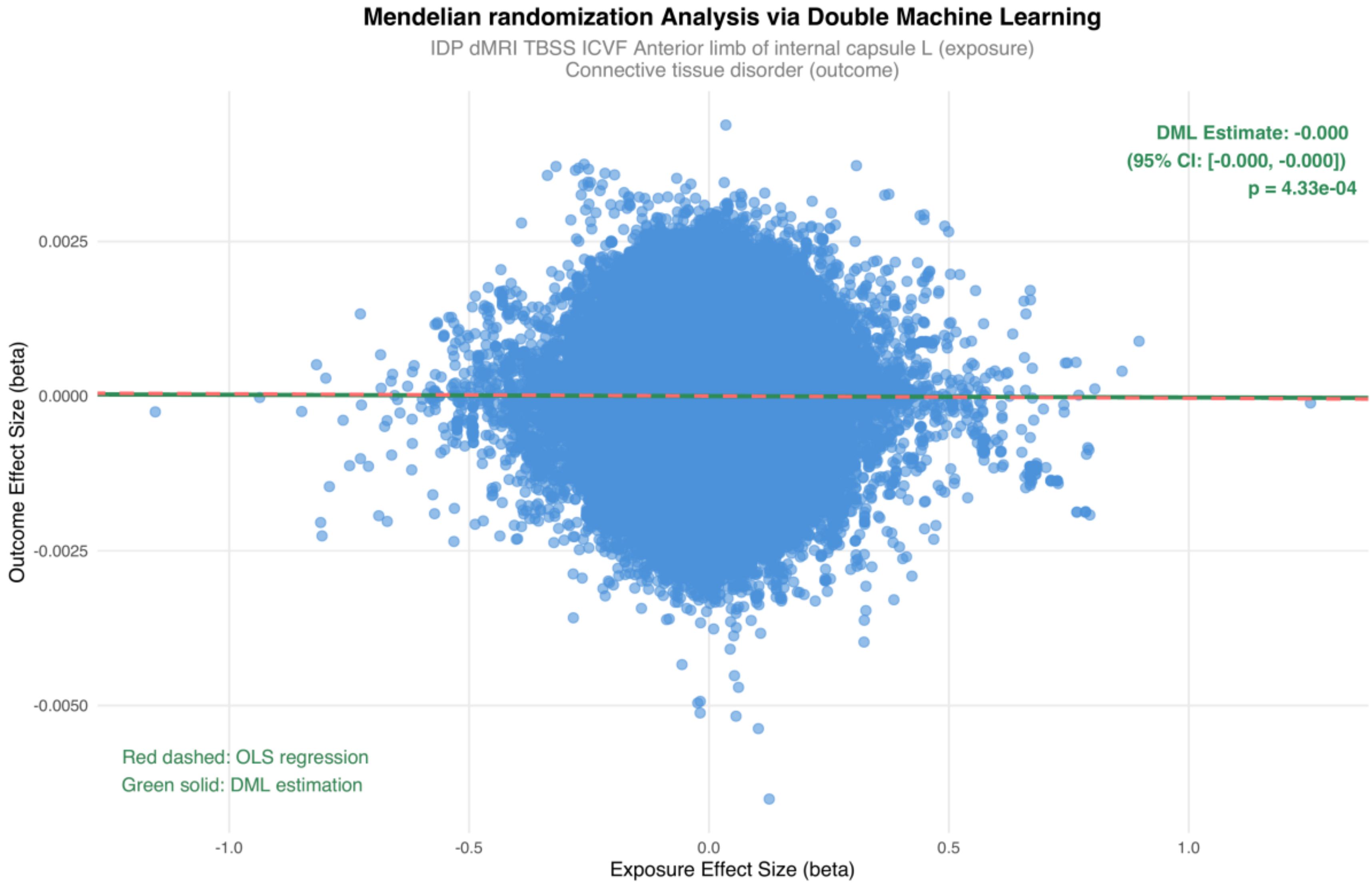
Mendelian randomization Analysis via Double Machine Learning
IDP dMRI TBSS ICVF Anterior limb of internal capsule L (exposure)
Connective tissue disorder (outcome)
DML Estimate: -0.000
(95% CI: [-0.000, -0.000])
p = 4.33e-04
0.0025
0.0000
-0.0025
-0.0050
Outcome Effect Size (beta)
Red dashed: OLS regression
Green solid: DML estimation
-1.0
-0.5
0.0
0.5
1.0
Exposure Effect Size (beta)

B

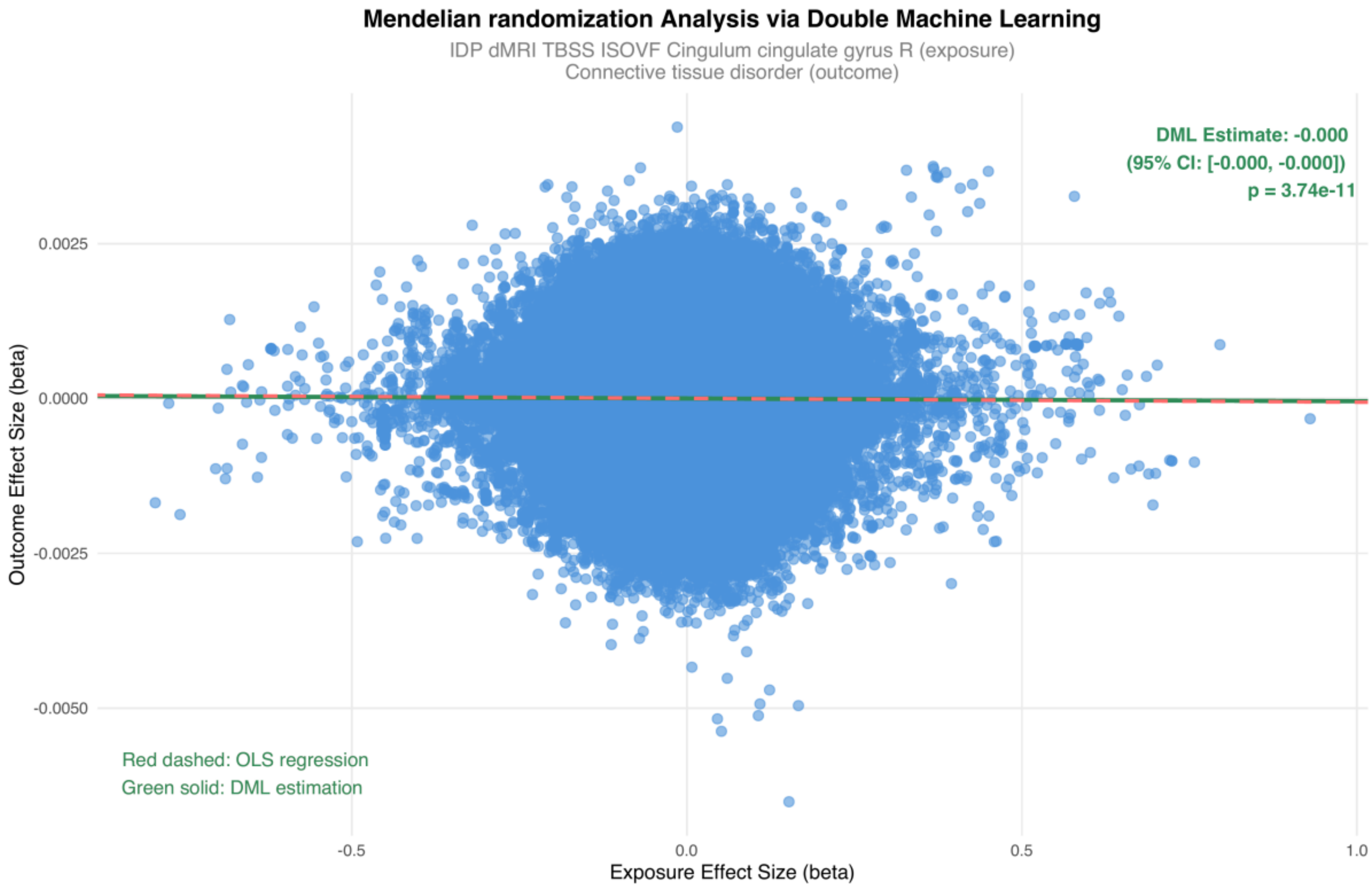
Mendelian randomization Analysis via Double Machine Learning
IDP dMRI TBSS ISOVF Cingulum cingulate gyrus R (exposure)
Connective tissue disorder (outcome)
DML Estimate: -0.000
(95% CI: [-0.000, -0.000])
p = 3.74e-11
0.0025
0.0000
-0.0025
-0.0050
Outcome Effect Size (beta)
Red dashed: OLS regression
Green solid: DML estimation
-0.5
0.0
0.5
1.0
Exposure Effect Size (beta)

C

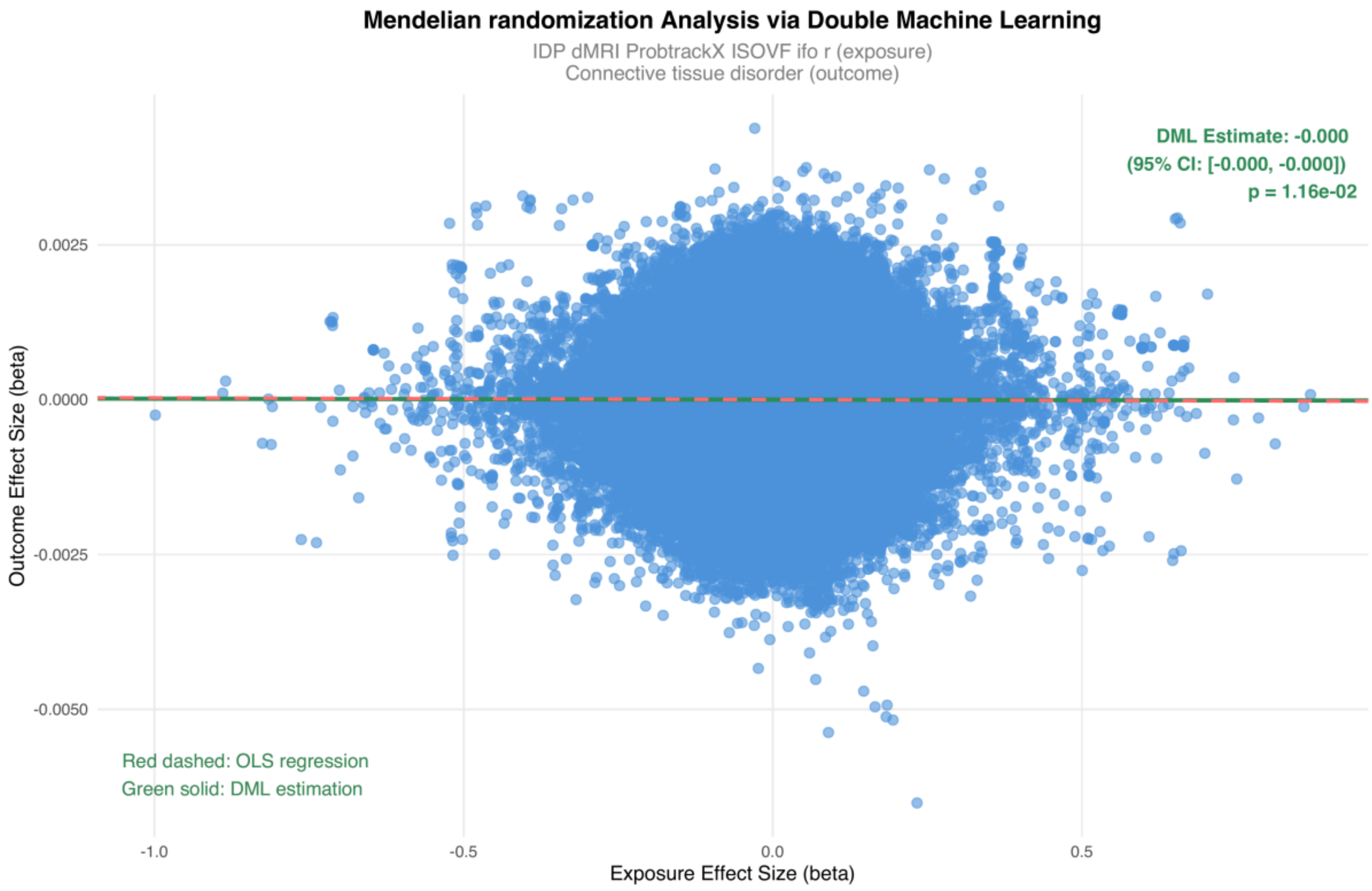

Mendelian randomization Analysis via Double Machine Learning
IDP dMRI ProbtrackX ISOVF ifo r (exposure)
Connective tissue disorder (outcome)
DML Estimate: -0.000
(95% CI: [-0.000, -0.000])
p = 1.16e-02
Red dashed: OLS regression
Green solid: DML estimation
Outcome Effect Size (beta)
0.0025
0.0000
-0.0025
-0.0050
-1.0
-0.5
0.0
0.5
Exposure Effect Size (beta)

D

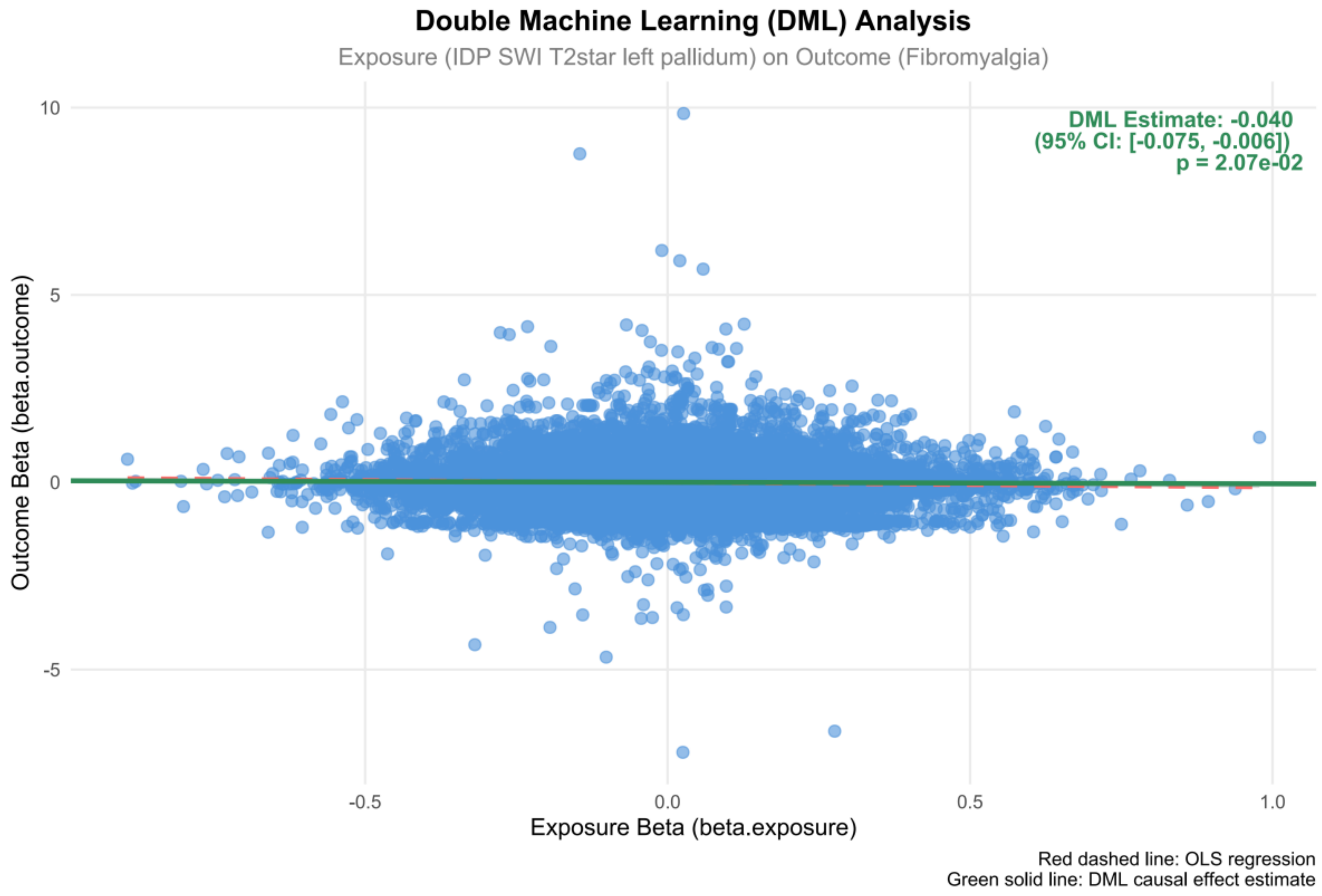

Double Machine Learning (DML) Analysis
Exposure (IDP SWI T2star left pallidum) on Outcome (Fibromyalgia)
DML Estimate: -0.040
(95% CI: [-0.075, -0.006])
p = 2.07e-02
Outcome Beta (beta.outcome)
10
5
0
-5
-0.5
0.0
0.5
1.0
Exposure Beta (beta.exposure)
Red dashed line: OLS regression
Green solid line: DML causal effect estimate

E

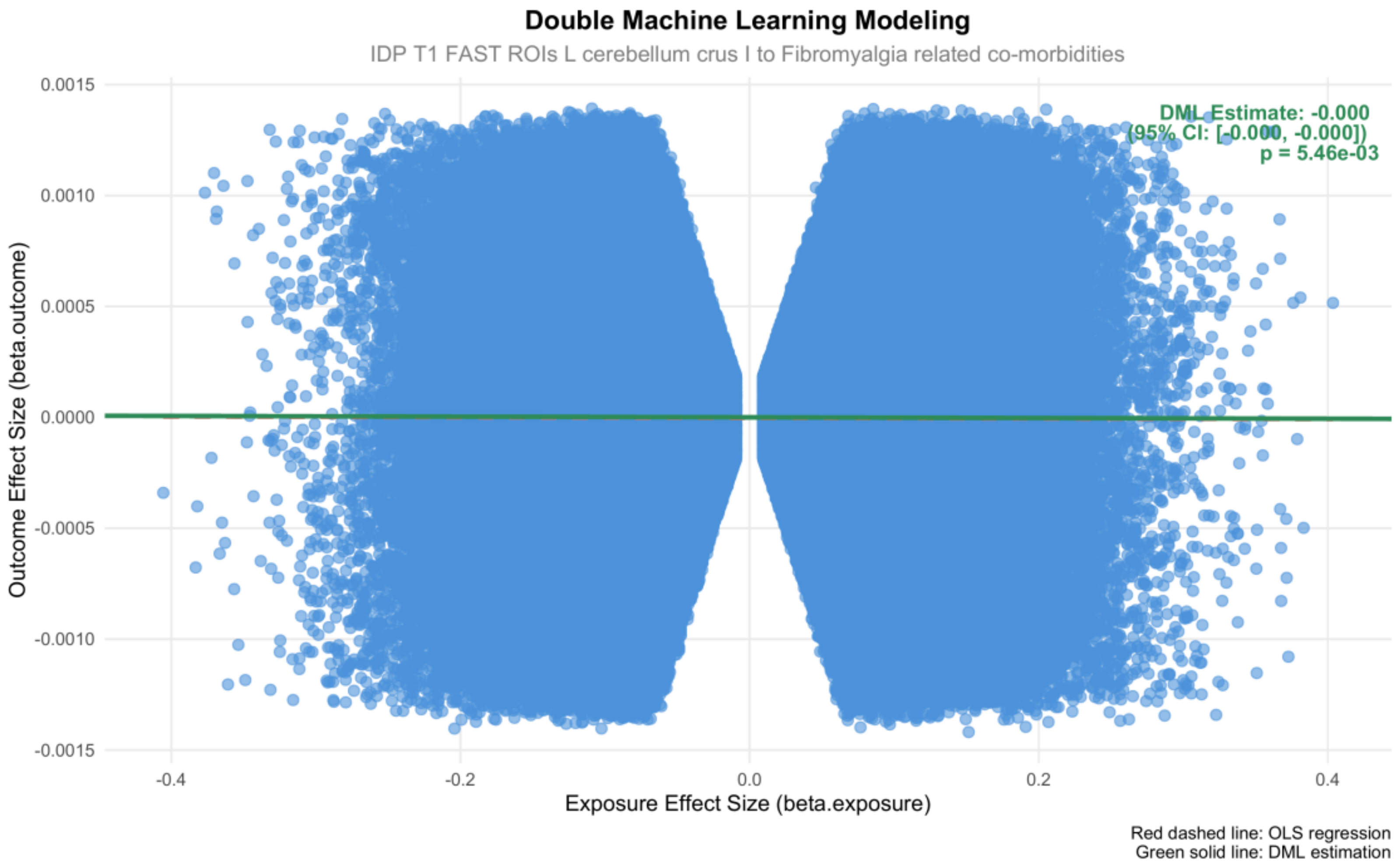

Double Machine Learning Modeling
IDP T1 FAST ROIs L cerebellum crus I to Fibromyalgia related co-morbidities
DML Estimate: -0.000
(95% CI: [-0.000, -0.000])
p = 5.46e-03
Outcome Effect Size (beta.outcome)
Exposure Effect Size (beta.exposure)
Red dashed line: OLS regression
Green solid line: DML estimation

F

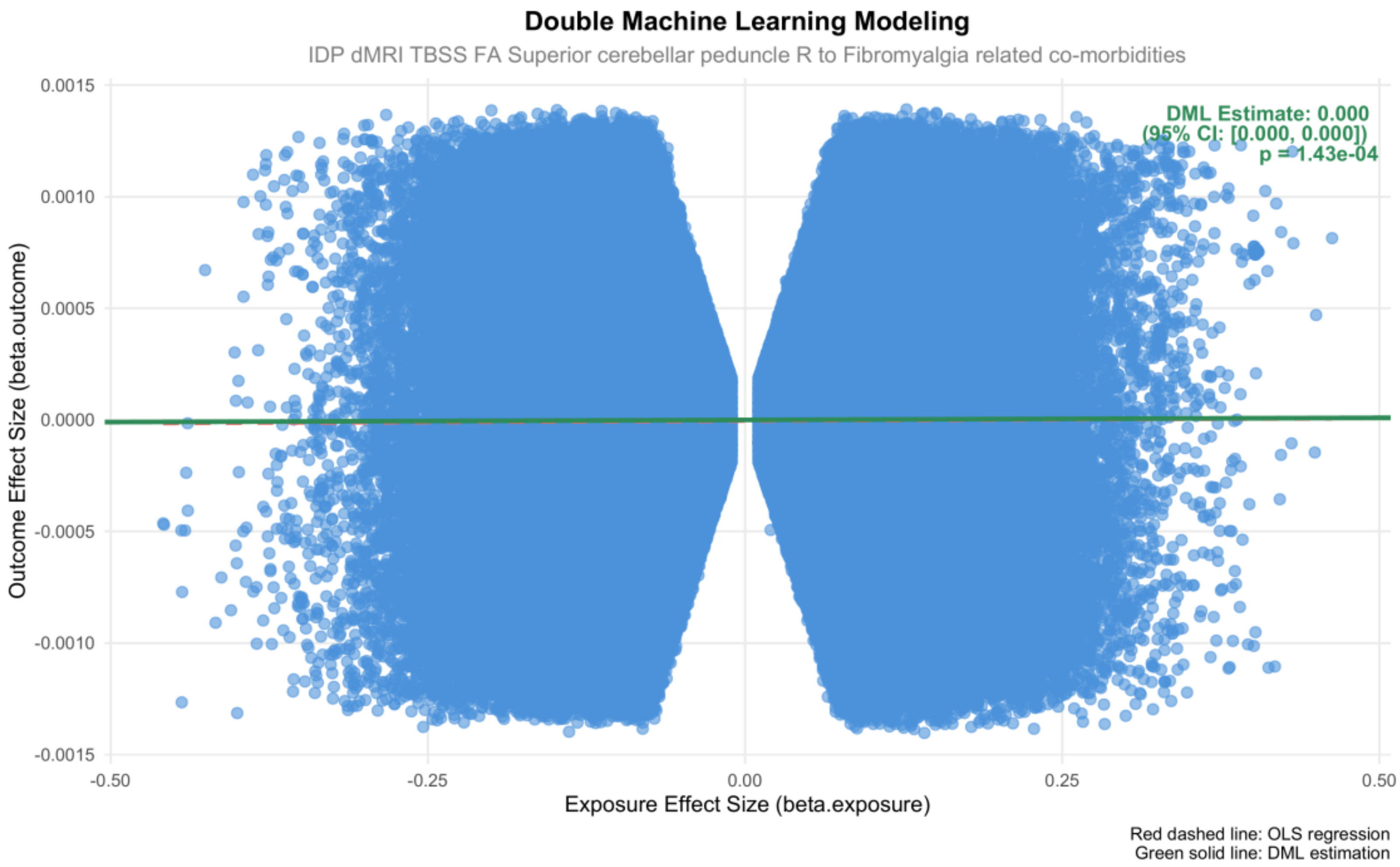

Double Machine Learning Modeling
IDP dMRI TBSS FA Superior cerebellar peduncle R to Fibromyalgia related co-morbidities
DML Estimate: 0.000
(95% CI: [0.000, 0.000])
p = 1.43e-04
Outcome Effect Size (beta.outcome)
Exposure Effect Size (beta.exposure)
Red dashed line: OLS regression
Green solid line: DML estimation

G

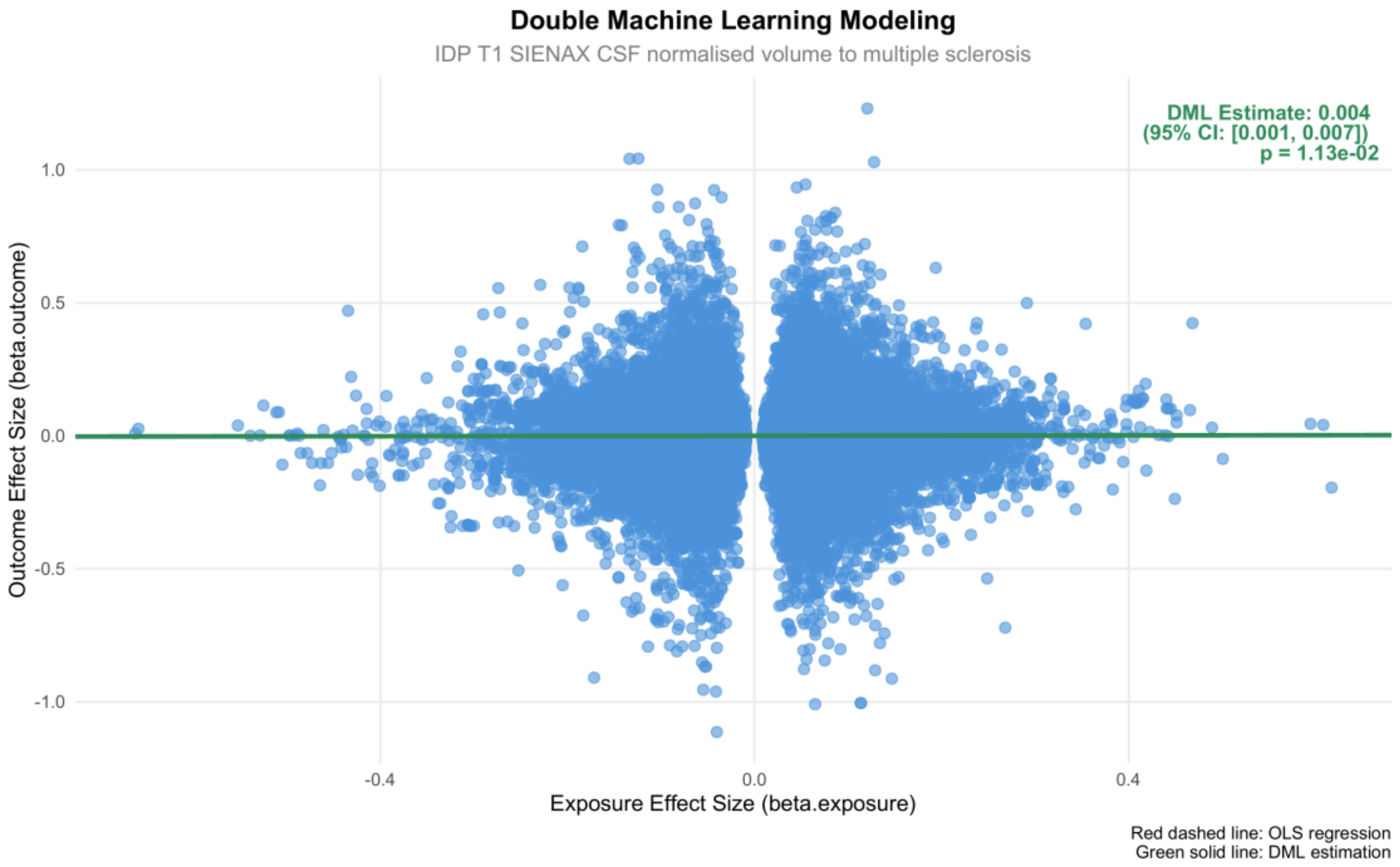

Figure S3. Double Machine Learning modeling results

(A – G) Visualization of DML-estimated causal effects:

(A) IDP dMRI TBSS ICVF Anterior limb of internal capsule L to Connective tissue disorder; (B) IDP dMRI TBSS ISOVF Cingulum cingulate gyrus R to Connective tissue disorder; (C) IDP dMRI ProbtrackX ISOVF ifo r to Connective tissue disorder; (D) IDP SWI T2star left pallidum to Fibromyalgia; (E) IDP T1 FAST ROIs L cerebellum crus I to Fibromyalgia related co-morbidities; (F) IDP dMRI TBSS FA Superior cerebellar peduncle R to Fibromyalgia related co-morbidities; (G) IDP T1 SIENAX CSF normalised volume to Multiple Sclerosis

Each scatterplot represents single-nucleotide polymorphisms (SNPs) used as instrumental variables; the x-axis indicates the exposure effect (beta.exposure), and the y-axis indicates the outcome effect (beta.outcome). The solid green line shows the DML-estimated causal slope (intercept = 0), and the dashed red line indicates the conventional ordinary least-squares trend. For all models (A – G), DML estimates were statistically significant ($P < 0.05$) and directionally consistent with two-sample MR results, confirming robust causal inference.

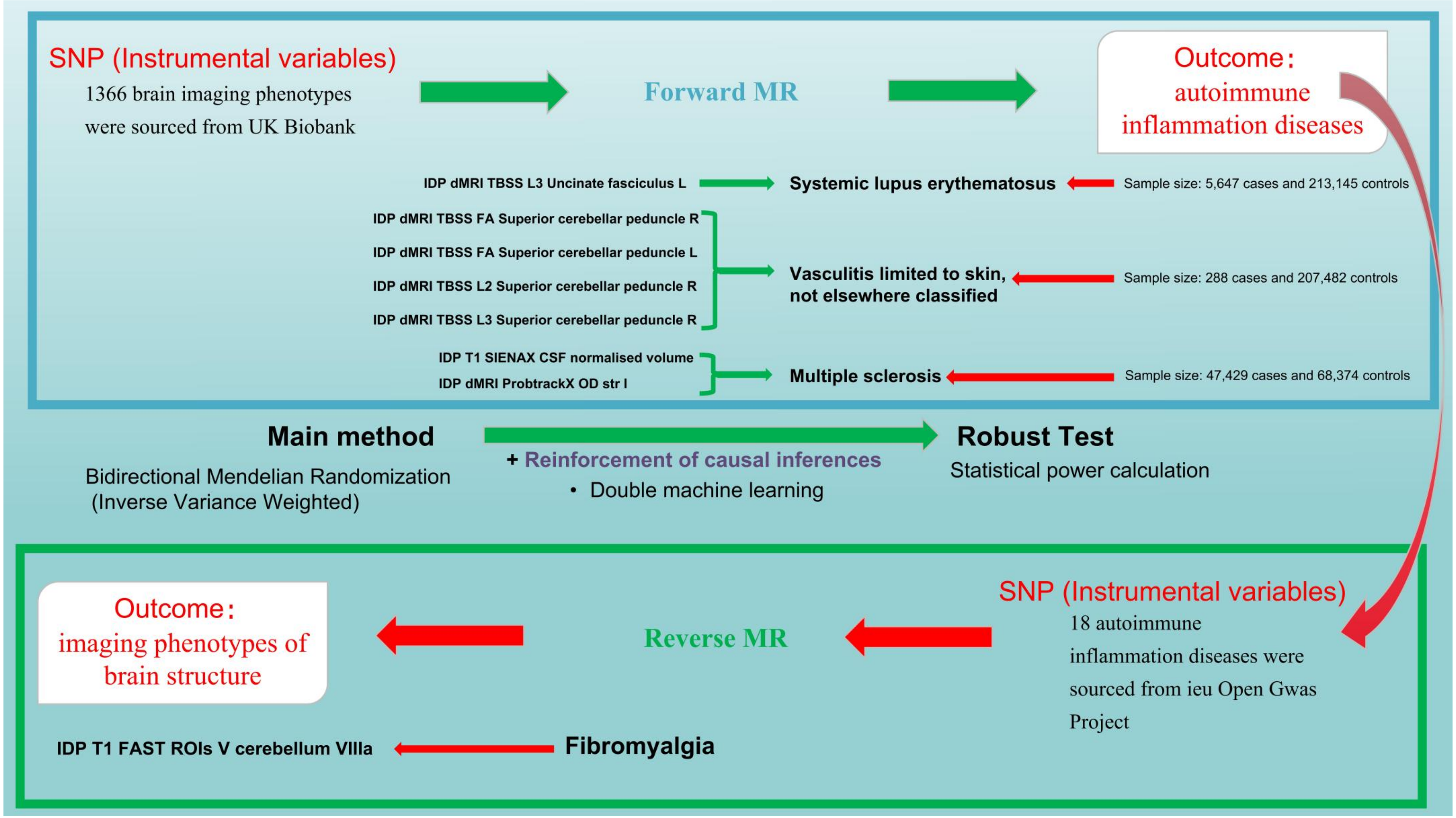

Figure S4. Graphical Abstract

This schematic integrates the eight statistically significant causal relationships identified between 1,366 brain imaging-derived phenotypes (IDPs) and 18 autoimmune inflammatory diseases (AIDs). Arrows indicate the direction of causality inferred from bidirectional two-sample MR analyses.

Statistical tests: Causal estimates were derived using inverse variance weighted (IVW) MR as the primary method, with sensitivity analyses including MR-Egger, weighted median, MR-PRESSO, and leave-one-out tests. DML validation employed orthogonalized cross-fitting with Lasso and Random Forest regressors.

Data presentation: Results are shown as ORs or β values with 95% confidence intervals. Significance thresholds were Bonferroni-corrected (forward MR: $P < 5.75 \times 10^{-4}$; reverse MR: $P < 1.67 \times 10^{-2}$).
No significance symbols are used in this figure.

Table S1. Autoimmune Inflammatory Diseases GWAS summary statistics, main source and description

Supplementary Table 1: Autoimmune Inflammatory Diseases GWAS summary statistics, main source and description					
**Autoimmune Inflammatory Diseases**	**Open GWAS Id**	**PubMed ID**	**Consortium**	**Sample Sizes**	**Ancestry**
Behcet's disease	ebi-a-GCST90018798	34594039	NA	27 cases and 317,225 controls	EUR
Connective tissue disorder	ebi-a-GCST90038660	33959723	NA	3,206 cases and 481,392 controls	NA
Dermatopolymyositis	finn-b-M13_DERMATOPOLY	NA	NA	208 cases and 213,145 controls	EUR
Diseases of the musculoskeletal system and connective tissue	finn-b-M13_MUSCULOSKELETAL	NA	NA	115,768 cases and 103,024 controls	EUR
Drug-induced systemic lupus erythematosus	finn-b-DRUGADVERS_SYSTEMIC_LUPUS_ERYTHEMAT	NA	NA	101 cases and 218,691 controls	EUR
Fibromyalgia	finn-b-M13_FIBROMYALGIA	NA	NA	737 cases and 167,641 controls	EUR
Fibromyalgia related co-morbidities	ukb-d-FIBRO_COMORB	NA	NA	2,305 cases and 358,889 controls	EUR
Giant cell arteritis	finn-b-M13_GIANTCELL	NA	NA	459 cases and 213,145 controls	EUR
Giant cell arteritis with polymyalgia rheumatica	finn-b-GIANT_CELL_TEMP_ARTERITIS	NA	NA	421 cases and 217,965 controls	EUR
Multiple Sclerosis	ieu-b-18	31604244	IMSGC	47,429 cases and 68,374 controls	EUR
Polyarteritis nodosa	finn-b-M13_POLNODOSA	NA	NA	82 cases and 213,145 controls	EUR
Polymyositis	ebi-a-GCST90018891	34594039	NA	44 cases and 350,228 controls	EUR
Sjogren's syndrome (Firth correction)	ebi-a-GCST90013879	34017140	NA	407,746 individuals	EUR
Systemic connective tissue disorders	finn-b-M13_SYSTCONNECT	NA	NA	5,647 cases and 213,145 controls	EUR
Systemic lupus erythematosus	ebi-a-GCST90018917	34594039	NA	647 cases and 482,264 controls	EUR
Systemic sclerosis	finn-b-M13_SYSTSLCE	NA	NA	302 cases and 213,145 controls	EUR
TMD muscular pain lnked with fibromyalgia	finn-b-DENTAL_TMD_FIBRO	NA	NA	2,530 cases and 216,262 controls	EUR
Vasculitis limited to skin, not elsewhere classified	finn-b-L12_VASCULITISSKIN	NA	NA	288 cases and 207,482 controls	EUR
**IMSGC, International Multiple Sclerosis Genetics Consortium; EUR, European**					

Table S2. Information of IVs for significant exposure-outcome pairs in forward MR analyses

Supplementary Table 2: Information of IVs for significant exposure-outcome pairs in forward MR analyses

Exposure	Outcome	SNP	Effect_Allele	Other_Allele	Beta.Exposure	Beta.Outcome	SE.Outcome	Pval.Outcome	SE.Exposure	Pval.exposure	R^2	*F*-statistic
IDP dMRI TBSS L3 Uncinate fasciculus L	Systemic lupus erythematosus	rs6840385	T	G	0.093	0.1147	0.0579	0.0476102	0.0157	2.95E-09	0.003703521	29.41861892
		rs13164785	G	T	0.1199	0.1623	0.0539	0.002618	0.0184	8.71E-11	0.004399744	34.97344584
IDP dMRI TBSS FA Superior cerebellar peduncle R	Vasculitis limited to skin, not elsewhere classified	rs4697414	T	C	-0.1866	0.4185	0.1207	0.000524397	0.0197	3.63E-21	0.010131798	81.0037669
		rs12479469	A	G	-0.111	0.204	0.0868	0.0188001	0.0164	1.38E-11	0.005533366	44.03471485
IDP dMRI TBSS FA Superior cerebellar peduncle L		rs4697414	T	C	-0.183	0.4185	0.1207	0.000524397	0.0196	1.17E-20	0.009744632	77.87790926
		rs12479469	A	G	-0.095	0.204	0.0868	0.0188001	0.0163	5.89E-09	0.004053131	32.20701586
IDP dMRI TBSS L2 Superior cerebellar peduncle R		rs34221784	C	A	0.1825	0.4183	0.1207	0.000529103	0.02	1.12E-19	0.00966559	77.24005015
		rs12479469	A	G	0.104	0.204	0.0868	0.0188001	0.0167	4.37E-10	0.004857469	38.62965579
IDP dMRI TBSS L3 Superior cerebellar peduncle R		rs34221784	C	A	0.1883	0.4183	0.1207	0.000529103	0.0202	1.20E-20	0.010289713	82.27942244
		rs12479469	A	G	0.1012	0.204	0.0868	0.0188001	0.0168	1.58E-09	0.004599434	36.56811627
IDP T1 SIENAX CSF normalised volume	multiple sclerosis	rs12146713	C	T	0.1277	0.0750469	0.0301446	0.01279	0.022	6.76E-09	0.00274855	21.81197647
		rs4843552	A	G	0.0857	0.0422929	0.0171297	0.01355	0.0129	3.39E-11	0.003581266	28.44400476
IDP dMRI ProbtrackX OD str l		rs12146713	C	T	-0.1543	0.0750469	0.0301446	0.01279	0.0249	5.75E-10	0.004012857	31.88570489
		rs9933149	C	T	-0.0947	0.0539198	0.0168252	0.00135201	0.0148	1.86E-10	0.004232563	33.63888315

Table S3. Information of IVs for significant exposure-outcome pairs in reverse MR analyses

Supplementary Table 3: Information of IVs for significant exposure-outcome pairs in reverse MR analyses												
Exposure	Outcome	SNP	Effect_Allele	Other_Allele	Beta.Exposure	Beta.Outcome	SE.Outcome	Pval.Outcome	SE.Exposure	Pval.exposure	R^2	F-statistic
Fibromyalgia	IDP T1 FAST ROIs V cerebellum VIIIa	rs116605722	A	G	3.4068	-0.0567	0.0497	0.251189	0.6857	6.75E-07	0.061142499	10965.38015
		rs1319080	T	G	0.6491	-0.0188	0.0285	0.512861	0.1447	7.26E-06	0.030742043	5340.396988
		rs149973466	G	C	0.5558	0.0074	0.0235	0.758578	0.124	7.38E-06	0.030644557	5322.926673
		rs11895756	T	G	0.4847	0.0131	0.0276	0.630957	0.1076	6.65E-06	0.029936621	5196.164109
		rs7669612	C	T	0.3158	-0.0131	0.0171	0.446684	0.0709	8.56E-06	0.02856292	4950.717146
		rs7735256	T	C	0.3005	-0.0065	0.0153	0.676083	0.0627	1.64E-06	0.033175183	5777.576797
		rs115371152	G	A	1.254	-0.035	0.0542	0.512861	0.2777	6.32E-06	0.035045051	6115.047665
		rs16880904	T	C	1.9542	-0.0777	0.0565	0.169824	0.4281	4.99E-06	0.042736038	7516.968506
		rs2189930	T	C	0.2383	0.0071	0.013	0.57544	0.0538	9.26E-06	0.027473497	4756.556847
		rs7837516	C	T	-0.3337	-0.0137	0.0164	0.398107	0.0697	1.69E-06	0.033213002	5784.389279
		rs73208758	C	T	-0.337	0.0109	0.0157	0.489779	0.075	7.00E-06	0.035779502	6247.958324
		rs142033922	G	C	0.6584	-0.0885	0.036	0.0138038	0.1412	3.11E-06	0.034732932	6058.625941
		rs1031161	G	A	0.2803	-0.0369	0.0133	0.00562341	0.0565	6.92E-07	0.034678472	6048.784944
		rs149161986	T	C	0.9424	-0.0083	0.04	0.831764	0.2127	9.38E-06	0.033636792	5860.765873
		rs117622163	G	A	1.2262	-0.1437	0.0749	0.0549541	0.2751	8.29E-06	0.035652567	6224.972961

		rs4486972	G	A	-0.3265	-0.0054	0.0137	0.691831	0.0579	1.69E-08	0.046285426	8171.579994
		rs72993349	A	C	0.4715	-0.0091	0.0235	0.691831	0.1018	3.63E-06	0.032001882	5566.486902

Table S4. Heterogeneity and Pleiotropy analyses for significant exposure-outcome pairs in forward MR

Supplementary Table 4: Heterogeneity and Pleiotropy analyses for significant exposure-outcome pairs in forward MR								
Exposure	Outcome	Heterogeneity				Pleiotropy		
		method	Q	Q_df	Q_pval	egger_intercept	se	pval
IDP dMRI TBSS L3 Uncinate fasciculus L	Systemic lupus erythematosus	Inverse variance weighted	0.024539527	1	0.875519929	NA	NA	NA
IDP dMRI TBSS FA Superior cerebellar peduncle R	Vasculitis limited to skin, not elsewhere classified	Inverse variance weighted	0.159206677	1	0.689887962	NA	NA	NA
IDP dMRI TBSS FA Superior cerebellar peduncle L	Vasculitis limited to skin, not elsewhere classified	Inverse variance weighted	0.015328635	1	0.901466612	NA	NA	NA
IDP dMRI TBSS L2 Superior cerebellar peduncle R	Vasculitis limited to skin, not elsewhere classified	Inverse variance weighted	0.096333092	1	0.75627525	NA	NA	NA
IDP dMRI TBSS L3 Superior cerebellar peduncle R	Vasculitis limited to skin, not elsewhere classified	Inverse variance weighted	0.036884681	1	0.847700025	NA	NA	NA
IDP T1 SIENAX CSF normalised volume	multiple sclerosis	Inverse variance weighted	0.092711752	1	0.760757622	NA	NA	NA
IDP dMRI ProbtrackX OD str l	multiple sclerosis	Inverse variance weighted	0.098802557	1	0.753271372	NA	NA	NA

Table S5. Heterogeneity and Pleiotropy analyses for significant exposure-outcome pairs in reverse MR

Supplementary Table 5: Heterogeneity and Pleiotropy analyses for significant exposure-outcome pairs in reverse MR								
Exposure	Outcome	Heterogeneity				Pleiotropy		
		method	Q	Q_df	Q_pval	egger_intercept	se	pval
Fibromyalgia	IDP T1 FAST ROIs V cerebellum VIIIa	MR Egger	17.61259122	15	0.283579978	0.001117008	0.007978995	0.890527516
		Inverse variance weighted	17.63560291	16	0.345662601			

Table S6. Significant results of forward MR

Supplementary Table 6: Significant results of forward MR											
Exposure	Outcome	method	N-SNP	beta	se (standard error)	*P*-value	low_CI 95% of beta	up_CI 95% of beta	OR (Odd Ratio)	low_CI 95% of OR	up_CI 95% of OR
IDP dMRI TBSS L3 Uncinate fasciculus L	Systemic lupus erythematosus	Inverse variance weighted	2	1.312403191	0.364461647	0.000317073	0.598058364	2.026748019	3.715091068	1.818584341	7.589365713
IDP dMRI TBSS FA Superior cerebellar peduncle R	Vasculitis limited to skin, not elsewhere classified	Inverse variance weighted	2	-2.0782617	0.498420405	3.05E-05	-3.055165693	-1.101357706	0.125147567	0.047114914	0.332419449
IDP dMRI TBSS FA Superior cerebellar peduncle L	Vasculitis limited to skin, not elsewhere classified	Inverse variance weighted	2	-2.239089498	0.534782836	2.83E-05	-3.287263857	-1.190915138	0.106555479	0.037355921	0.303942987
IDP dMRI TBSS L2 Superior cerebellar peduncle R	Vasculitis limited to skin, not elsewhere classified	Inverse variance weighted	2	2.164566187	0.518352995	2.97E-05	1.148594315	3.180538058	8.710822225	3.153756605	24.05969557
IDP dMRI TBSS L3 Superior cerebellar peduncle R	Vasculitis limited to skin, not elsewhere classified	Inverse variance weighted	2	2.147759434	0.513453766	2.88E-05	1.141390053	3.154128815	8.565644986	3.131117761	23.43261404
IDP T1 SIENAX CSF normalised volume	multiple sclerosis	Inverse variance weighted	2	0.532827733	0.152541376	0.000477618	0.233846637	0.83180883	1.703743235	1.263450711	2.297470718
IDP dMRI ProbtrackX OD str l	multiple sclerosis	Inverse variance weighted	2	-0.53180104	0.131442161	5.21E-05	-0.789427675	-0.274174405	0.587545822	0.454104616	0.760199481

Table S7. Significant results of reverse MR

Supplementary Table 7: Significant results of reverse MR											
**Exposure**	**Outcome**	**method**	**N-SNP**	**beta**	**se (standard error)**	***P*-value**	**low_CI 95% of beta**	**up_CI 95% of beta**	**OR (Odd Ratio)**	**low_CI 95% of OR**	**up_CI 95% of OR**
Fibromyalgia	IDP T1 FAST ROIs V cerebellum VIIIa	Inverse variance weighted	17	-0.023399581	0.009456096	0.013340416	-0.041933529	-0.004865634	0.976872066	0.95893352	0.995146184

Table S8. Statistical power calculation of forward MR

Supplementary Table 8: Statistical power calculation of forward MR					
Outcome	Exposure	Method	Pval	Variance_explained	Power_80_5
Behcet's disease	IDP T1 SIENAX peripheral grey normalised volume	Wald ratio	0.826771984	0.05	1
	IDP T1 FAST ROIs R temp occ fusif cortex	Wald ratio	0.408308365	0.05	0.037411651
	IDP T1 FAST ROIs R occ pole	Inverse variance weighted	0.826034208	0.05	1
	IDP T1 FAST ROIs L thalamus	Inverse variance weighted	0.492164567	0.05	0.999884927
	IDP T1 FAST ROIs R thalamus	Wald ratio	0.755556791	0.05	0.999864416
	IDP T1 FAST ROIs L putamen	Wald ratio	0.965125851	0.05	1
	IDP T1 FAST ROIs R putamen	Wald ratio	0.965125851	0.05	1
	IDP T1 FAST ROIs L pallidum	Wald ratio	0.405382793	0.05	1
	IDP T1 FIRST left caudate volume	Wald ratio	0.353209471	0.05	1
	IDP T1 FAST ROIs L hippocampus	Inverse variance weighted	0.82360636	0.05	1
	IDP T1 FAST ROIs R hippocampus	Wald ratio	0.716878816	0.05	1
	IDP T1 FAST ROIs L ventral striatum	Wald ratio	0.965125851	0.05	1
	IDP T1 FAST ROIs R ventral striatum	Inverse variance weighted	0.490182562	0.05	1
	IDP T1 FAST ROIs L cerebellum VI	Wald ratio	0.127539339	0.05	0.047129341
	IDP T1 FAST ROIs R cerebellum crus I	Inverse variance weighted	0.421661787	0.05	0.310996119
	IDP T1 FAST ROIs V cerebellum crus II	Inverse variance weighted	0.19811829	0.05	0.104779727
	IDP T1 FAST ROIs R cerebellum crus II	Inverse variance weighted	0.327391857	0.05	1
	IDP T1 FIRST left putamen volume	Wald ratio	0.903642939	0.05	1

	IDP T1 FAST ROIs R cerebellum VIIb	Wald ratio	0.838974553	0.05	1
	IDP T1 FAST ROIs L cerebellum VIIIb	Wald ratio	0.842174848	0.05	1
	IDP T1 FAST ROIs V cerebellum VIIIb	Inverse variance weighted	0.853685608	0.05	1
	IDP T1 FAST ROIs R cerebellum VIIIb	Wald ratio	0.965125851	0.05	1
	IDP T1 FAST ROIs L cerebellum IX	Inverse variance weighted	0.160346	0.05	1
	IDP T1 FAST ROIs V cerebellum IX	Inverse variance weighted	0.468084246	0.05	1
	IDP T1 FAST ROIs R cerebellum IX	Inverse variance weighted	0.20269617	0.05	1
	IDP T1 FAST ROIs V cerebellum X	Wald ratio	0.965125851	0.05	1
	IDP T2 FLAIR BIANCA WMH volume	Inverse variance weighted	0.886709803	0.05	1
	IDP T1 FIRST left pallidum volume	Wald ratio	0.53066964	0.05	1
	IDP T1 FIRST right pallidum volume	Wald ratio	0.53066964	0.05	1
	IDP dMRI TBSS FA Genu of corpus callosum	Wald ratio	0.347236808	0.05	1
	IDP dMRI TBSS FA Corticospinal tract L	Wald ratio	0.882980249	0.05	1
	IDP dMRI TBSS FA Superior cerebellar peduncle R	Inverse variance weighted	0.604191371	0.05	1
	IDP dMRI TBSS FA Superior cerebellar peduncle L	Inverse variance weighted	0.608018646	0.05	1
	IDP dMRI TBSS FA Posterior limb of internal capsule R	Wald ratio	0.982729495	0.05	1
	IDP dMRI TBSS FA Retrolenticular part of internal capsule R	Inverse variance weighted	0.837905307	0.05	1
	IDP dMRI TBSS FA Retrolenticular part of internal capsule L	Wald ratio	0.387635175	0.05	1
	IDP dMRI TBSS FA Anterior corona radiata R	Wald ratio	0.347236808	0.05	1
	IDP dMRI TBSS FA Anterior corona radiata L	Wald ratio	0.347236808	0.05	1

	IDP dMRI TBSS FA Posterior corona radiata L	Wald ratio	0.387635175	0.05	1
	IDP dMRI TBSS FA Posterior thalamic radiation R	Inverse variance weighted	0.589219155	0.05	0.999999174
	IDP dMRI TBSS FA Posterior thalamic radiation L	Inverse variance weighted	0.571836903	0.05	0.999994991
	IDP dMRI TBSS FA Sagittal stratum R	Inverse variance weighted	0.359754945	0.05	1
	IDP dMRI TBSS FA Sagittal stratum L	Wald ratio	0.413598574	0.05	1
	IDP dMRI TBSS FA Cingulum cingulate gyrus R	Inverse variance weighted	0.191257457	0.05	1
	IDP dMRI TBSS FA Superior longitudinal fasciculus L	Inverse variance weighted	0.491075221	0.05	1
	IDP dMRI TBSS FA Uncinate fasciculus L	Wald ratio	0.260719845	0.05	0.145366449
	IDP dMRI TBSS MD Genu of corpus callosum	Wald ratio	0.347236808	0.05	0.999982458
	IDP dMRI TBSS MD Body of corpus callosum	Wald ratio	0.817729035	0.05	1
	IDP dMRI TBSS MD Splenium of corpus callosum	Inverse variance weighted	0.905606201	0.05	1
	IDP dMRI TBSS MD Corticospinal tract R	Wald ratio	0.925002953	0.05	1
	IDP dMRI TBSS MD Inferior cerebellar peduncle R	Inverse variance weighted	0.052531666	0.05	0.012804093
	IDP dMRI TBSS MD Inferior cerebellar peduncle L	Wald ratio	0.387635175	0.05	0.165534309
	IDP dMRI TBSS MD Superior cerebellar peduncle R	Wald ratio	0.756080983	0.05	1
	IDP dMRI TBSS MD Superior cerebellar peduncle L	Wald ratio	0.75053355	0.05	1
	IDP dMRI TBSS MD Anterior limb of internal capsule L	Wald ratio	0.6945318	0.05	1
	IDP dMRI TBSS MD Retrolenticular part of internal capsule R	Inverse variance weighted	0.525464845	0.05	1
	IDP T1 FIRST left caudate volume plus IDP T1 FIRST right caudate volume	Wald ratio	0.176323566	0.05	1

	IDP T1 FIRST left putamen volume plus IDP T1 FIRST right putamen volume	Wald ratio	0.556725481	0.05	1
	IDP T1 FIRST left pallidum volume plus IDP T1 FIRST right pallidum volume	Wald ratio	0.53066964	0.05	1
	IDP dMRI TBSS MD Retrolenticular part of internal capsule L	Inverse variance weighted	0.561323243	0.05	1
	volume Left-Inf-Lat-Vent	Wald ratio	0.61142786	0.05	0.999999932
	volume Left-Cerebellum-White-Matter	Inverse variance weighted	0.235239029	0.05	1
	volume Left-Cerebellum-Cortex	Wald ratio	0.942960085	0.05	1
	IDP dMRI TBSS MD Anterior corona radiata R	Inverse variance weighted	0.902593193	0.05	1
	volume Left-Putamen	Wald ratio	0.866988451	0.05	1
	volume 3rd-Ventricle	Wald ratio	0.228283692	0.05	0.038481916
	volume 4th-Ventricle	Inverse variance weighted	0.600813451	0.05	1
	volume Brain-Stem	Inverse variance weighted	0.947503612	0.05	1
	volume Left-Hippocampus	Inverse variance weighted	0.800122102	0.05	1
	IDP dMRI TBSS MD Anterior corona radiata L	Inverse variance weighted	0.938862607	0.05	1
	volume Left-Accumbens-area	Wald ratio	0.965125851	0.05	1
	volume Right-Lateral-Ventricle	Inverse variance weighted	0.639993177	0.05	1
	volume Right-Inf-Lat-Vent	Wald ratio	0.754601741	0.05	0.999936057
	volume Right-Cerebellum-White-Matter	Wald ratio	0.277162399	0.05	1
	IDP dMRI TBSS MD Superior corona radiata R	Inverse variance weighted	0.903311626	0.05	1
	volume Right-Hippocampus	Wald ratio	0.716878816	0.05	1
	volume Right-Amygdala	Wald ratio	0.116232065	0.05	0.002834539

	volume Right-VentralDC	Wald ratio	0.361144771	0.05	0.016430318
	volume CC Posterior	Wald ratio	0.825063103	0.05	1
	IDP dMRI TBSS MD Superior corona radiata L	Inverse variance weighted	0.907169719	0.05	1
	IDP dMRI TBSS MD Posterior corona radiata R	Inverse variance weighted	0.557653214	0.05	1
	volume BrainSegVol-to-eTIV	Wald ratio	0.444340649	0.05	1
	IDP dMRI TBSS MD Posterior corona radiata L	Inverse variance weighted	0.589153507	0.05	1
	volume rhSurfaceHoles	Wald ratio	0.69869753	0.05	0.999999987
	DKTatlas lh cuneus area	Wald ratio	0.736121683	0.05	1
	IDP dMRI TBSS MD Posterior thalamic radiation R	Inverse variance weighted	0.544732957	0.05	1
	DKTatlas lh lateraloccipital area	Wald ratio	0.736121683	0.05	1
	DKTatlas lh lingual area	Wald ratio	0.754173402	0.05	1
	IDP dMRI TBSS MD Posterior thalamic radiation L	Inverse variance weighted	0.535895484	0.05	1
	DKTatlas lh parstriangularis area	Wald ratio	0.597887527	0.05	1
	DKTatlas lh pericalcarine area	Inverse variance weighted	0.919914825	0.05	1
	DKTatlas lh postcentral area	Inverse variance weighted	0.604103976	0.05	0.855963762
	DKTatlas lh posteriorcingulate area	Wald ratio	0.864012525	0.05	1
	DKTatlas lh precentral area	Wald ratio	0.424233059	0.05	0.979066056
	DKTatlas lh precuneus area	Wald ratio	0.036527454	0.05	0.002577332
	IDP dMRI TBSS MD Sagittal stratum R	Inverse variance weighted	0.517919791	0.05	1
	DKTatlas lh superiorparietal area	Wald ratio	0.827809977	0.05	1
	DKTatlas lh superiortemporal area	Wald ratio	0.575513693	0.05	1

	DKTatlas lh supramarginal area	Wald ratio	0.41652405	0.05	1
	DKTatlas lh WhiteSurfArea area	Wald ratio	0.470912914	0.05	0.403922936
	IDP dMRI TBSS MD Sagittal stratum L	Inverse variance weighted	0.614858273	0.05	1
	a2009s lh G&S subcentral area	Wald ratio	0.424233059	0.05	0.172861163
	a2009s lh G cuneus area	Wald ratio	0.777509097	0.05	1
	a2009s lh G front inf-Opercular area	Wald ratio	0.137608863	0.05	1
	IDP dMRI TBSS MD External capsule R	Wald ratio	0.347236808	0.05	0.998037431
	IDP dMRI TBSS MD External capsule L	Inverse variance weighted	0.364040485	0.05	0.999999999
	a2009s lh G pariet inf-Supramar area	Wald ratio	0.41652405	0.05	1
	a2009s lh G parietal sup area	Wald ratio	0.118946122	0.05	1
	a2009s lh G postcentral area	Wald ratio	0.07081741	0.05	0.007803082
	a2009s lh G precentral area	Wald ratio	0.424233059	0.05	0.970631642
	a2009s lh G precuneus area	Wald ratio	0.777509097	0.05	0.999999999
	a2009s lh S calcarine area	Wald ratio	0.754173402	0.05	1
	a2009s lh S central area	Wald ratio	0.438518687	0.05	0.999970891
	a2009s lh S collat transv ant area	Wald ratio	0.876938984	0.05	1
	a2009s lh S front middle area	Wald ratio	0.494890148	0.05	0.175057075
	IDP dMRI TBSS MD Cingulum hippocampus R	Inverse variance weighted	0.608958967	0.05	1
	a2009s lh S intrapariet&P trans area	Wald ratio	0.118946122	0.05	1
	IDP dMRI TBSS MD Cingulum hippocampus L	Inverse variance weighted	0.625254958	0.05	1
	a2009s lh S subparietal area	Wald ratio	0.754173402	0.05	0.999999904
	DKTatlas rh cuneus area	Wald ratio	0.754173402	0.05	1

	DKTatlas rh lateraloccipital area	Inverse variance weighted	0.7133366	0.05	1
	DKTatlas rh lingual area	Wald ratio	0.740965919	0.05	1
	DKTatlas rh parstriangularis area	Wald ratio	0.870755897	0.05	1
	DKTatlas rh pericalcarine area	Inverse variance weighted	0.838169175	0.05	1
	IDP dMRI TBSS MD Superior longitudinal fasciculus R	Inverse variance weighted	0.519439131	0.05	1
	DKTatlas rh postcentral area	Wald ratio	0.438518687	0.05	0.026674548
	DKTatlas rh precentral area	Wald ratio	0.424233059	0.05	0.956900005
	IDP dMRI TBSS MD Superior longitudinal fasciculus L	Inverse variance weighted	0.501310535	0.05	1
	a2009s rh G&S subcentral area	Wald ratio	0.438518687	0.05	0.367907439
	a2009s rh G&S cingul-Mid-Post area	Wald ratio	0.680293026	0.05	0.904556066
	a2009s rh G cuneus area	Wald ratio	0.754173402	0.05	1
	IDP dMRI TBSS MD Superior fronto-occipital fasciculus L	Wald ratio	0.816989369	0.05	1
	a2009s rh G oc-temp med-Lingual area	Wald ratio	0.740965919	0.05	1
	a2009s rh G parietal sup area	Wald ratio	0.418023144	0.05	0.756932174
	IDP dMRI TBSS MD Uncinate fasciculus R	Inverse variance weighted	0.468111126	0.05	1
	a2009s rh G precentral area	Wald ratio	0.424233059	0.05	0.808504964
	a2009s rh G precuneus area	Wald ratio	0.039895227	0.05	1
	IDP dMRI TBSS MD Uncinate fasciculus L	Inverse variance weighted	0.340939346	0.05	0.999999998
	a2009s rh Pole occipital area	Wald ratio	0.754173402	0.05	1
	a2009s rh S calcarine area	Wald ratio	0.740965919	0.05	1
	a2009s rh S central area	Wald ratio	0.424233059	0.05	0.999824376
	a2009s rh S collat transv ant area	Wald ratio	0.876938984	0.05	1

	a2009s rh S orbital med-olfact area	Wald ratio	0.365173579	0.05	0.021950959
	DKTatlas lh postcentral thickness	Wald ratio	0.438518687	0.05	0.680274583
	a2009s lh G insular short thickness	Wald ratio	0.454472154	0.05	0.192614484
	a2009s lh G postcentral thickness	Wald ratio	0.438518687	0.05	0.999490814
	IDP T1 SIENAX CSF normalised volume	Inverse variance weighted	0.580041029	0.05	0.999999873
	IDP dMRI TBSS MO Medial lemniscus R	Wald ratio	0.44245418	0.05	0.999999977
	a2009s lh S postcentral thickness	Wald ratio	0.628932913	0.05	0.999958237
	DKTatlas rh lateraloccipital thickness	Wald ratio	0.965125851	0.05	1
	DKTatlas rh parstriangularis thickness	Wald ratio	0.341618152	0.05	0.019217618
	DKTatlas rh postcentral thickness	Wald ratio	0.438518687	0.05	0.576607865
	DKTatlas rh posteriorcingulate thickness	Inverse variance weighted	0.894064281	0.05	1
	a2009s rh G&S cingul-Mid-Ant thickness	Wald ratio	0.697803017	0.05	0.996944069
	a2009s rh G cuneus thickness	Wald ratio	0.965125851	0.05	1
	a2009s rh G postcentral thickness	Wald ratio	0.405144641	0.05	0.987820482
	a2009s rh Pole occipital thickness	Wald ratio	0.965125851	0.05	1
	a2009s rh S circular insula ant thickness	Wald ratio	0.281015217	0.05	1
	a2009s rh S oc sup&transversal thickness	Wald ratio	0.170280699	0.05	1
	a2009s rh S parieto occipital thickness	Wald ratio	0.475912901	0.05	0.12012349
	a2009s rh S temporal transverse thickness	Wald ratio	0.541214753	0.05	0.77418447
	IDP dMRI TBSS MO Anterior corona radiata R	Wald ratio	0.564319444	0.05	1
	IDP dMRI TBSS MO Superior corona radiata R	Wald ratio	0.620770312	0.05	1
	IDP dMRI TBSS MO Cingulum cingulate gyrus L	Inverse variance weighted	0.585135141	0.05	0.999999999

	IDP dMRI TBSS L1 Genu of corpus callosum	Wald ratio	0.413598574	0.05	0.130118689
	IDP dMRI TBSS L1 Anterior limb of internal capsule L	Wald ratio	0.6945318	0.05	1
	IDP dMRI TBSS L1 Posterior limb of internal capsule R	Wald ratio	0.952903333	0.05	1
	IDP dMRI TBSS L1 Posterior limb of internal capsule L	Wald ratio	0.733569964	0.05	1
	IDP dMRI TBSS L1 Anterior corona radiata R	Wald ratio	0.347236808	0.05	0.998937307
	IDP dMRI TBSS L1 Anterior corona radiata L	Wald ratio	0.347236808	0.05	0.999723421
	IDP dMRI TBSS L1 Posterior corona radiata R	Wald ratio	0.595167944	0.05	0.99999034
	IDP dMRI TBSS L1 Posterior corona radiata L	Wald ratio	0.347236808	0.05	0.994551593
	IDP dMRI TBSS L1 Sagittal stratum L	Wald ratio	0.347236808	0.05	0.999723421
	IDP dMRI TBSS L1 External capsule R	Wald ratio	0.347236808	0.05	0.980409174
	IDP dMRI TBSS L1 Cingulum hippocampus L	Wald ratio	0.413598574	0.05	0.171026558
	IDP dMRI TBSS L1 Fornix cres+Stria terminalis L	Wald ratio	0.837662866	0.05	1
	IDP T1 FAST ROIs L precentral gyrus	Wald ratio	0.438518687	0.05	0.796888751
	IDP dMRI TBSS L1 Superior longitudinal fasciculus R	Inverse variance weighted	0.398730307	0.05	1
	IDP dMRI TBSS L1 Uncinate fasciculus R	Wald ratio	0.387635175	0.05	0.056050473
	IDP dMRI TBSS L1 Uncinate fasciculus L	Wald ratio	0.493627458	0.05	0.999998319
	IDP dMRI TBSS L2 Pontine crossing tract	Wald ratio	0.44245418	0.05	1
	IDP dMRI TBSS L2 Body of corpus callosum	Wald ratio	0.347236808	0.05	0.998734588
	IDP dMRI TBSS L2 Corticospinal tract R	Wald ratio	0.925002953	0.05	1
	IDP T1 FAST ROIs L temporal pole	Wald ratio	0.814572818	0.05	1

	IDP dMRI TBSS L2 Superior cerebellar peduncle R	Inverse variance weighted	0.605011313	0.05	1
	IDP dMRI TBSS L2 Superior cerebellar peduncle L	Wald ratio	0.75053355	0.05	1
	IDP dMRI TBSS L2 Posterior limb of internal capsule R	Inverse variance weighted	0.716087066	0.05	1
	IDP dMRI TBSS L2 Posterior limb of internal capsule L	Wald ratio	0.635233532	0.05	1
	IDP dMRI TBSS L2 Retrolenticular part of internal capsule R	Inverse variance weighted	0.815403416	0.05	1
	IDP dMRI TBSS L2 Retrolenticular part of internal capsule L	Wald ratio	0.387635175	0.05	0.399013338
	IDP dMRI TBSS L2 Anterior corona radiata R	Inverse variance weighted	0.9082455	0.05	1
	IDP dMRI TBSS L2 Anterior corona radiata L	Wald ratio	0.347236808	0.05	0.999991059
	IDP dMRI TBSS L2 Superior corona radiata L	Inverse variance weighted	0.482059416	0.05	1
	IDP dMRI TBSS L2 Posterior corona radiata R	Inverse variance weighted	0.50739823	0.05	1
	IDP dMRI TBSS L2 Posterior corona radiata L	Inverse variance weighted	0.522797973	0.05	1
	IDP dMRI TBSS L2 Posterior thalamic radiation R	Inverse variance weighted	0.543469055	0.05	1
	IDP dMRI TBSS L2 Posterior thalamic radiation L	Inverse variance weighted	0.543564945	0.05	1
	IDP dMRI TBSS L2 Sagittal stratum R	Inverse variance weighted	0.529678714	0.05	1
	IDP dMRI TBSS L2 Sagittal stratum L	Wald ratio	0.413598574	0.05	0.569947332
	IDP dMRI TBSS L2 Cingulum cingulate gyrus R	Wald ratio	0.347236808	0.05	0.999998945
	IDP dMRI TBSS L2 Cingulum cingulate gyrus L	Wald ratio	0.413598574	0.05	0.280946411
	IDP dMRI TBSS L2 Cingulum hippocampus R	Inverse variance weighted	0.571226956	0.05	1

	IDP dMRI TBSS L2 Cingulum hippocampus L	Wald ratio	0.347236808	0.05	0.999999016
	IDP dMRI TBSS L2 Uncinate fasciculus L	Wald ratio	0.347236808	0.05	0.986847654
	IDP dMRI TBSS L3 Middle cerebellar peduncle	Wald ratio	0.387635175	0.05	0.091821718
	IDP dMRI TBSS L3 Genu of corpus callosum	Wald ratio	0.347236808	0.05	0.999734623
	IDP dMRI TBSS L3 Body of corpus callosum	Wald ratio	0.347236808	0.05	0.992802612
	IDP dMRI TBSS L3 Splenium of corpus callosum	Inverse variance weighted	0.399657876	0.05	0.999990364
	IDP dMRI TBSS L3 Inferior cerebellar peduncle R	Wald ratio	0.347236808	0.05	0.999988014
	IDP dMRI TBSS L3 Inferior cerebellar peduncle L	Wald ratio	0.347236808	0.05	0.999935801
	IDP dMRI TBSS L3 Superior cerebellar peduncle R	Inverse variance weighted	0.606674674	0.05	1
	IDP dMRI TBSS L3 Superior cerebellar peduncle L	Wald ratio	0.75053355	0.05	1
	IDP dMRI TBSS L3 Cerebral peduncle R	Wald ratio	0.293231374	0.05	0.013778064
	IDP dMRI TBSS L3 Anterior limb of internal capsule R	Wald ratio	0.327762933	0.05	0.025714092
	IDP dMRI TBSS L3 Anterior limb of internal capsule L	Wald ratio	0.588412932	0.05	0.70989917
	IDP dMRI TBSS L3 Retrolenticular part of internal capsule R	Inverse variance weighted	0.540595191	0.05	1
	IDP dMRI TBSS L3 Retrolenticular part of internal capsule L	Wald ratio	0.387635175	0.05	0.464465485
	IDP dMRI TBSS L3 Anterior corona radiata R	Inverse variance weighted	0.906473194	0.05	1
	IDP dMRI TBSS L3 Anterior corona radiata L	Inverse variance weighted	0.947287007	0.05	1
	IDP dMRI TBSS L3 Superior corona radiata R	Wald ratio	0.413598574	0.05	0.284778592
	IDP dMRI TBSS L3 Superior corona radiata L	Wald ratio	0.413598574	0.05	0.241799562

	IDP dMRI TBSS L3 Posterior corona radiata R	Inverse variance weighted	0.981861477	0.05	1
	IDP dMRI TBSS L3 Posterior corona radiata L	Wald ratio	0.387635175	0.05	0.266332731
	IDP dMRI TBSS L3 Posterior thalamic radiation R	Inverse variance weighted	0.596600557	0.05	1
	IDP dMRI TBSS L3 Posterior thalamic radiation L	Inverse variance weighted	0.560065105	0.05	1
	IDP dMRI TBSS L3 Sagittal stratum R	Inverse variance weighted	0.556867866	0.05	1
	IDP dMRI TBSS L3 Sagittal stratum L	Inverse variance weighted	0.638139015	0.05	1
	IDP dMRI TBSS L3 External capsule R	Wald ratio	0.347236808	0.05	0.999350923
	IDP dMRI TBSS L3 External capsule L	Wald ratio	0.347236808	0.05	0.99976075
	IDP dMRI TBSS L3 Cingulum cingulate gyrus R	Inverse variance weighted	0.9342839	0.05	1
	IDP dMRI TBSS L3 Cingulum cingulate gyrus L	Wald ratio	0.347236808	0.05	0.999999742
	IDP dMRI TBSS L3 Cingulum hippocampus R	Wald ratio	0.347236808	0.05	0.999999843
	IDP dMRI TBSS L3 Cingulum hippocampus L	Wald ratio	0.347236808	0.05	0.9999999
	IDP dMRI TBSS L3 Fornix cres+Stria terminalis R	Wald ratio	0.91490942	0.05	1
	IDP dMRI TBSS L3 Superior longitudinal fasciculus R	Inverse variance weighted	0.530878366	0.05	1
	IDP dMRI TBSS L3 Superior longitudinal fasciculus L	Inverse variance weighted	0.514949957	0.05	1
	IDP dMRI TBSS L3 Uncinate fasciculus R	Wald ratio	0.347236808	0.05	0.997044369
	IDP dMRI TBSS L3 Uncinate fasciculus L	Inverse variance weighted	0.818288195	0.05	1
	IDP dMRI TBSS ICVF Middle cerebellar peduncle	Inverse variance weighted	0.634215626	0.05	0.999999998
	IDP dMRI TBSS ICVF Fornix	Wald ratio	0.816989369	0.05	1

	IDP dMRI TBSS ICVF Medial lemniscus R	Inverse variance weighted	0.196012444	0.05	0.924422334
	IDP dMRI TBSS ICVF Medial lemniscus L	Wald ratio	0.506732281	0.05	0.999983713
	IDP dMRI TBSS ICVF Superior cerebellar peduncle L	Inverse variance weighted	0.380611076	0.05	1
	IDP dMRI TBSS ICVF Cerebral peduncle L	Inverse variance weighted	0.496820436	0.05	1
	IDP dMRI TBSS ICVF Anterior limb of internal capsule R	Wald ratio	0.347236808	0.05	1
	IDP dMRI TBSS ICVF Posterior thalamic radiation R	Inverse variance weighted	0.63510673	0.05	1
	IDP dMRI TBSS ICVF External capsule L	Inverse variance weighted	0.319275409	0.05	1
	IDP dMRI TBSS ICVF Cingulum cingulate gyrus R	Inverse variance weighted	0.960713862	0.05	1
	IDP dMRI TBSS ICVF Cingulum cingulate gyrus L	Inverse variance weighted	0.6991812	0.05	1
	IDP dMRI TBSS ICVF Fornix cres+Stria terminalis R	Inverse variance weighted	0.800982543	0.05	1
	IDP dMRI TBSS ICVF Fornix cres+Stria terminalis L	Wald ratio	0.413598574	0.05	1
	IDP dMRI TBSS ICVF Superior fronto-occipital fasciculus R	Inverse variance weighted	0.481248726	0.05	1
	IDP dMRI TBSS ICVF Superior fronto-occipital fasciculus L	Wald ratio	0.413598574	0.05	1
	IDP dMRI TBSS ICVF Uncinate fasciculus R	Wald ratio	0.347236808	0.05	1
	IDP dMRI TBSS ICVF Uncinate fasciculus L	Wald ratio	0.347236808	0.05	1
	IDP dMRI TBSS ICVF Tapetum R	Wald ratio	0.387635175	0.05	1
	IDP dMRI TBSS ICVF Tapetum L	Wald ratio	0.387635175	0.05	1
	IDP dMRI TBSS OD Genu of corpus callosum	Wald ratio	0.996871049	0.05	1
	IDP dMRI TBSS OD Cerebral peduncle R	Wald ratio	0.861064129	0.05	1
	IDP dMRI TBSS OD Cerebral peduncle L	Wald ratio	0.861064129	0.05	1

	IDP dMRI TBSS OD Posterior limb of internal capsule L	Wald ratio	0.733569964	0.05	1
	IDP dMRI TBSS OD Posterior corona radiata R	Wald ratio	0.438430905	0.05	1
	IDP dMRI TBSS OD External capsule R	Inverse variance weighted	0.900193494	0.05	1
	IDP dMRI TBSS OD External capsule L	Wald ratio	0.544651185	0.05	0.993527998
	IDP dMRI TBSS OD Superior longitudinal fasciculus R	Wald ratio	0.47155178	0.05	1
	IDP dMRI TBSS ISOVF Fornix	Wald ratio	0.473191009	0.05	1
	IDP dMRI TBSS ISOVF External capsule R	Wald ratio	0.136282383	0.05	0.010497193
	IDP dMRI TBSS ISOVF Cingulum cingulate gyrus R	Inverse variance weighted	0.876198521	0.05	1
	IDP dMRI ProbtrackX FA ar l	Wald ratio	0.347236808	0.05	1
	IDP dMRI ProbtrackX FA atr l	Wald ratio	0.347236808	0.05	1
	IDP dMRI ProbtrackX FA atr r	Wald ratio	0.387635175	0.05	1
	IDP dMRI ProbtrackX FA fma	Wald ratio	0.387635175	0.05	1
	IDP dMRI ProbtrackX FA ifo l	Inverse variance weighted	0.641665303	0.05	1
	IDP dMRI ProbtrackX FA ifo r	Inverse variance weighted	0.729303847	0.05	1
	IDP dMRI ProbtrackX FA ilf l	Inverse variance weighted	0.825215754	0.05	1
	IDP dMRI ProbtrackX FA ilf r	Inverse variance weighted	0.819177297	0.05	1
	IDP dMRI ProbtrackX FA ml l	Wald ratio	0.778167347	0.05	1
	IDP dMRI ProbtrackX FA ptr l	Wald ratio	0.413598574	0.05	1
	IDP dMRI ProbtrackX FA ptr r	Wald ratio	0.597567008	0.05	1
	IDP dMRI ProbtrackX FA slf l	Inverse variance weighted	0.711198362	0.05	1
	IDP dMRI ProbtrackX FA slf r	Wald ratio	0.387635175	0.05	1

	IDP dMRI ProbtrackX FA str l	Wald ratio	0.17236353	0.05	0.648457896
	IDP dMRI ProbtrackX FA unc l	Wald ratio	0.347236808	0.05	1
	IDP dMRI ProbtrackX FA unc r	Wald ratio	0.347236808	0.05	1
	IDP dMRI ProbtrackX MD ar l	Wald ratio	0.347236808	0.05	0.998000116
	IDP dMRI ProbtrackX MD ar r	Wald ratio	0.347236808	0.05	0.997203154
	IDP dMRI ProbtrackX MD atr l	Wald ratio	0.347236808	0.05	0.999073242
	IDP dMRI ProbtrackX MD atr r	Wald ratio	0.347236808	0.05	0.995826431
	IDP dMRI ProbtrackX MD fmi	Inverse variance weighted	0.935837876	0.05	1
	IDP dMRI ProbtrackX MD ifo l	Inverse variance weighted	0.568783145	0.05	1
	IDP dMRI ProbtrackX MD ifo r	Inverse variance weighted	0.552176018	0.05	1
	IDP dMRI ProbtrackX MD ilf l	Inverse variance weighted	0.564887922	0.05	1
	IDP dMRI ProbtrackX MD ilf r	Inverse variance weighted	0.605446033	0.05	1
	IDP dMRI ProbtrackX MD ptr l	Wald ratio	0.347236808	0.05	0.999412673
	IDP dMRI ProbtrackX MD ptr r	Wald ratio	0.347236808	0.05	0.999932963
	IDP dMRI ProbtrackX MD slf l	Inverse variance weighted	0.913033646	0.05	1
	IDP dMRI ProbtrackX MD slf r	Inverse variance weighted	0.528448165	0.05	1
	IDP dMRI ProbtrackX MD str l	Wald ratio	0.04968909	0.05	1
	IDP dMRI ProbtrackX MD str r	Wald ratio	0.347236808	0.05	0.958205906
	IDP dMRI ProbtrackX MD unc l	Wald ratio	0.347236808	0.05	0.999954662
	IDP dMRI ProbtrackX MD unc r	Inverse variance weighted	0.157824342	0.05	0.99928874
	IDP dMRI ProbtrackX MO atr l	Wald ratio	0.946037781	0.05	1

	IDP dMRI ProbtrackX MO ml r	Wald ratio	0.728006078	0.05	1
	IDP T1 FAST ROIs L intracalc cortex	Wald ratio	0.754173402	0.05	1
	IDP dMRI ProbtrackX L1 fmi	Wald ratio	0.413598574	0.05	0.141209221
	IDP dMRI ProbtrackX L1 ifo l	Wald ratio	0.413598574	0.05	0.232530782
	IDP dMRI ProbtrackX L1 ifo r	Wald ratio	0.413598574	0.05	0.185494859
	IDP dMRI ProbtrackX L1 ilf l	Wald ratio	0.347236808	0.05	0.9965788
	IDP dMRI ProbtrackX L1 ilf r	Wald ratio	0.413598574	0.05	0.224597695
	IDP T1 FAST ROIs R intracalc cortex	Wald ratio	0.754173402	0.05	1
	IDP dMRI ProbtrackX L1 slf l	Wald ratio	0.347236808	0.05	0.994922797
	IDP dMRI ProbtrackX L1 slf r	Inverse variance weighted	0.037538209	0.05	0.004005107
	IDP dMRI ProbtrackX L1 str r	Wald ratio	0.645930949	0.05	0.999999274
	IDP dMRI ProbtrackX L1 unc l	Inverse variance weighted	0.356481105	0.05	0.99999999
	IDP dMRI ProbtrackX L1 unc r	Inverse variance weighted	0.139286337	0.05	0.113208676
	IDP dMRI ProbtrackX L2 ar l	Wald ratio	0.347236808	0.05	0.996116228
	IDP dMRI ProbtrackX L2 ar r	Wald ratio	0.347236808	0.05	0.995099501
	IDP dMRI ProbtrackX L2 atr l	Wald ratio	0.347236808	0.05	0.999835776
	IDP dMRI ProbtrackX L2 atr r	Wald ratio	0.347236808	0.05	0.999468784
	IDP dMRI ProbtrackX L2 cgc l	Wald ratio	0.413598574	0.05	0.342424548
	IDP dMRI ProbtrackX L2 cgc r	Wald ratio	0.413598574	0.05	0.64976138
	IDP dMRI ProbtrackX L2 cgh l	Wald ratio	0.182126704	0.05	1
	IDP dMRI ProbtrackX L2 fma	Wald ratio	0.788040153	0.05	1
	IDP dMRI ProbtrackX L2 fmi	Inverse variance weighted	0.933807765	0.05	1

	IDP dMRI ProbtrackX L2 ifo l	Inverse variance weighted	0.554546142	0.05	1
	IDP dMRI ProbtrackX L2 ifo r	Inverse variance weighted	0.529222058	0.05	1
	IDP dMRI ProbtrackX L2 ilf l	Inverse variance weighted	0.568680422	0.05	1
	IDP dMRI ProbtrackX L2 ilf r	Inverse variance weighted	0.593779392	0.05	1
	IDP dMRI ProbtrackX L2 ptr l	Wald ratio	0.347236808	0.05	0.999934397
	IDP dMRI ProbtrackX L2 ptr r	Wald ratio	0.338409172	0.05	0.999995892
	IDP dMRI ProbtrackX L2 slf l	Inverse variance weighted	0.907196378	0.05	1
	IDP dMRI ProbtrackX L2 slf r	Inverse variance weighted	0.503437008	0.05	1
	IDP dMRI ProbtrackX L2 str l	Wald ratio	0.04968909	0.05	1
	IDP dMRI ProbtrackX L2 str r	Wald ratio	0.04968909	0.05	1
	IDP dMRI ProbtrackX L2 unc l	Wald ratio	0.347236808	0.05	0.999947189
	IDP dMRI ProbtrackX L2 unc r	Inverse variance weighted	0.161714032	0.05	0.998824335
	IDP dMRI ProbtrackX L3 ar l	Wald ratio	0.347236808	0.05	0.998895116
	IDP dMRI ProbtrackX L3 ar r	Wald ratio	0.347236808	0.05	0.997044369
	IDP dMRI ProbtrackX L3 atr l	Wald ratio	0.347236808	0.05	0.999948326
	IDP dMRI ProbtrackX L3 atr r	Wald ratio	0.347236808	0.05	0.999809788
	IDP dMRI ProbtrackX L3 cgc l	Wald ratio	0.387635175	0.05	0.39761552
	IDP dMRI ProbtrackX L3 cgc r	Wald ratio	0.387635175	0.05	0.481777507
	IDP dMRI ProbtrackX L3 ifo l	Inverse variance weighted	0.971564359	0.05	1
	IDP dMRI ProbtrackX L3 ifo r	Inverse variance weighted	0.580930243	0.05	1
	IDP dMRI ProbtrackX L3 ilf l	Inverse variance weighted	0.598150629	0.05	1

	IDP dMRI ProbtrackX L3 ilf r	Inverse variance weighted	0.630970358	0.05	1
	IDP dMRI ProbtrackX L3 ptr l	Wald ratio	0.347236808	0.05	0.999977592
	IDP dMRI ProbtrackX L3 ptr r	Wald ratio	0.338409172	0.05	0.999994617
	IDP dMRI ProbtrackX L3 slf l	Inverse variance weighted	0.924086853	0.05	1
	IDP dMRI ProbtrackX L3 slf r	Inverse variance weighted	0.546269442	0.05	1
	IDP dMRI ProbtrackX L3 str l	Wald ratio	0.338409172	0.05	0.952778474
	IDP dMRI ProbtrackX L3 str r	Wald ratio	0.413598574	0.05	0.096524046
	IDP dMRI ProbtrackX L3 unc l	Wald ratio	0.347236808	0.05	0.999896981
	IDP dMRI ProbtrackX L3 unc r	Inverse variance weighted	0.151660455	0.05	0.99876035
	IDP dMRI ProbtrackX ICVF cgc l	Inverse variance weighted	0.716378822	0.05	1
	IDP dMRI ProbtrackX ICVF cgc r	Wald ratio	0.413598574	0.05	1
	IDP dMRI ProbtrackX ICVF cgh r	Wald ratio	0.347236808	0.05	1
	IDP dMRI ProbtrackX ICVF ifo l	Inverse variance weighted	0.981060301	0.05	1
	IDP dMRI ProbtrackX ICVF ifo r	Inverse variance weighted	0.985412921	0.05	1
	IDP dMRI ProbtrackX ICVF ilf l	Inverse variance weighted	0.9988923	0.05	1
	IDP dMRI ProbtrackX ICVF ilf r	Inverse variance weighted	0.652934447	0.05	1
	IDP dMRI ProbtrackX ICVF mcp	Inverse variance weighted	0.08411044	0.05	1
	IDP dMRI ProbtrackX ICVF ml l	Wald ratio	0.493627458	0.05	1
	IDP dMRI ProbtrackX ICVF ml r	Wald ratio	0.347236808	0.05	1
	IDP dMRI ProbtrackX ICVF ptr l	Inverse variance weighted	0.668167346	0.05	1

	IDP dMRI ProbtrackX ICVF unc l	Inverse variance weighted	0.983105357	0.05	1
	IDP dMRI ProbtrackX ICVF unc r	Inverse variance weighted	0.911102968	0.05	1
	IDP dMRI ProbtrackX OD atr r	Wald ratio	0.568673096	0.05	0.548360199
	IDP dMRI ProbtrackX OD ilf l	Wald ratio	0.326420846	0.05	1
	IDP dMRI ProbtrackX OD slf l	Wald ratio	0.269363463	0.05	0.526795261
	IDP dMRI ProbtrackX OD str l	Inverse variance weighted	0.618550471	0.05	1
	IDP dMRI ProbtrackX ISOVF atr r	Wald ratio	0.347236808	0.05	1
	IDP dMRI ProbtrackX ISOVF cgc l	Wald ratio	0.279387064	0.05	1
	IDP dMRI ProbtrackX ISOVF ifo r	Inverse variance weighted	0.895314438	0.05	1
	IDP dMRI ProbtrackX ISOVF ilf l	Wald ratio	0.228022645	0.05	0.008850027
	IDP dMRI ProbtrackX ISOVF slf l	Inverse variance weighted	0.664861794	0.05	1
	IDP dMRI ProbtrackX ISOVF unc l	Wald ratio	0.203377749	0.05	0.276713404
	IDP dMRI ProbtrackX ISOVF unc r	Wald ratio	0.203289894	0.05	0.598108784
	IDP T1 SIENAX brain-normalised volume	Wald ratio	0.135256052	0.05	1
	IDP dMRI ProbtrackX FA fmi	MR Egger	0.945914632	0.05	1
		Weighted median	0.228438305	0.05	0.564998896
		Inverse variance weighted	0.197217379	0.05	0.719655494
		Simple mode	0.309885005	0.05	0.03246531
		Weighted mode	0.243954915	0.05	0.08782424
	IDP dMRI ProbtrackX ICVF ar l	MR Egger	0.617040489	0.05	0.013953439
		Weighted median	0.064225949	0.05	1
		Inverse variance weighted	0.074477664	0.05	1

		Simple mode	0.260934744	0.05	1
		Weighted mode	0.228697081	0.05	1
	IDP dMRI ProbtrackX ICVF ar r	MR Egger	0.405121929	0.05	0.002542843
		Weighted median	0.909950312	0.05	1
		Inverse variance weighted	0.628236591	0.05	1
		Simple mode	0.879106292	0.05	1
		Weighted mode	0.903476779	0.05	1
	IDP dMRI ProbtrackX ICVF atr l	MR Egger	0.555654218	0.05	0.117003345
		Weighted median	0.388156365	0.05	1
		Inverse variance weighted	0.386419422	0.05	1
		Simple mode	0.610310225	0.05	1
		Weighted mode	0.472657913	0.05	1
	IDP dMRI ProbtrackX ICVF atr r	MR Egger	0.801598604	0.05	1
		Weighted median	0.655760699	0.05	1
		Inverse variance weighted	0.161623744	0.05	1
		Simple mode	0.804928648	0.05	1
		Weighted mode	0.857589701	0.05	1
	IDP dMRI ProbtrackX ICVF cgh l	MR Egger	0.192879784	0.05	0.052579769
		Weighted median	0.364134619	0.05	1
		Inverse variance weighted	0.333649616	0.05	1
		Simple mode	0.336974685	0.05	0.999999999
		Weighted mode	0.404267921	0.05	1

	IDP dMRI ProbtrackX ICVF cst l	MR Egger	0.411467199	0.05	0.124352145
		Weighted median	0.626184715	0.05	1
		Inverse variance weighted	0.344150685	0.05	1
		Simple mode	0.863121644	0.05	1
		Weighted mode	0.841337033	0.05	1
	IDP dMRI ProbtrackX ICVF cst r	MR Egger	0.229104281	0.05	0.021913184
		Weighted median	0.540119178	0.05	1
		Inverse variance weighted	0.67864239	0.05	1
		Simple mode	0.664937576	0.05	1
		Weighted mode	0.48774538	0.05	0.999956965
	IDP dMRI ProbtrackX ICVF fma	MR Egger	0.511614854	0.05	0.963551104
		Weighted median	0.647410855	0.05	1
		Inverse variance weighted	0.884008159	0.05	1
		Simple mode	0.994654007	0.05	1
		Weighted mode	0.958802001	0.05	1
	IDP dMRI ProbtrackX ICVF fmi	MR Egger	0.766625014	0.05	1
		Weighted median	0.440970967	0.05	1
		Inverse variance weighted	0.2988819	0.05	1
		Simple mode	0.580938229	0.05	1
		Weighted mode	0.602870527	0.05	1
	IDP dMRI ProbtrackX ICVF ptr r	MR Egger	0.590988534	0.05	0.074610364
		Weighted median	0.625380196	0.05	1

		Inverse variance weighted	0.747235263	0.05	1
		Simple mode	0.868725276	0.05	1
		Weighted mode	0.613521958	0.05	1
	IDP dMRI ProbtrackX ICVF slf l	MR Egger	0.717318888	0.05	1
		Weighted median	0.290892503	0.05	1
		Inverse variance weighted	0.135319092	0.05	1
		Simple mode	0.13260058	0.05	0.999977322
		Weighted mode	0.872265656	0.05	1
	IDP dMRI ProbtrackX ICVF slf r	MR Egger	0.109284352	0.05	0.877891683
		Weighted median	0.911404155	0.05	1
		Inverse variance weighted	0.771463503	0.05	1
		Simple mode	0.993642055	0.05	1
		Weighted mode	0.839146755	0.05	1
	IDP dMRI ProbtrackX ICVF str l	MR Egger	0.312650535	0.05	0.999994199
		Weighted median	0.639785816	0.05	1
		Inverse variance weighted	0.855895984	0.05	1
		Simple mode	0.749367293	0.05	1
		Weighted mode	0.745066686	0.05	1
	IDP dMRI ProbtrackX ICVF str r	MR Egger	0.976604653	0.05	1
		Weighted median	0.534779789	0.05	0.999999978
		Inverse variance weighted	0.487265514	0.05	0.999999979
		Simple mode	0.701473991	0.05	1

		Weighted mode	0.621632955	0.05	0.999999986
	IDP dMRI ProbtrackX ISOVF ilf r	MR Egger	0.847340903	0.05	1
		Weighted median	0.625990408	0.05	1
		Inverse variance weighted	0.56516562	0.05	1
		Simple mode	0.720818952	0.05	1
		Weighted mode	0.733616447	0.05	1
	IDP dMRI ProbtrackX ISOVF slf r	MR Egger	0.948762581	0.05	1
		Weighted median	0.88488534	0.05	1
		Inverse variance weighted	0.65009742	0.05	1
		Simple mode	0.938170239	0.05	1
		Weighted mode	0.94894741	0.05	1
	IDP dMRI ProbtrackX L3 fmi	MR Egger	0.355456428	0.05	0.002815613
		Weighted median	0.47607083	0.05	1
		Inverse variance weighted	0.931648667	0.05	1
		Simple mode	0.570310905	0.05	1
		Weighted mode	0.516724773	0.05	1
	IDP dMRI ProbtrackX MD cgc l	MR Egger	0.349825623	0.05	0.002884933
		Weighted median	0.46544214	0.05	1
		Inverse variance weighted	0.927307043	0.05	1
		Simple mode	0.568650756	0.05	1
		Weighted mode	0.51819927	0.05	1
	IDP dMRI ProbtrackX MD cgc r	MR Egger	0.360748907	0.05	1

		Weighted median	0.276380141	0.05	1
		Inverse variance weighted	0.46511834	0.05	1
		Simple mode	0.413691314	0.05	1
		Weighted mode	0.373329774	0.05	1
	IDP dMRI TBSS FA Splenium of corpus callosum	MR Egger	0.836411772	0.05	1
		Weighted median	0.574231573	0.05	1
		Inverse variance weighted	0.440428086	0.05	1
		Simple mode	0.691135212	0.05	1
		Weighted mode	0.672316001	0.05	1
	IDP dMRI TBSS ICVF Anterior corona radiata L	MR Egger	0.541035726	0.05	0.002620293
		Weighted median	0.353295455	0.05	0.999999727
		Inverse variance weighted	0.866892487	0.05	1
		Simple mode	0.572287482	0.05	0.999998761
		Weighted mode	0.42837951	0.05	0.999990766
	IDP dMRI TBSS ICVF Anterior corona radiata R	MR Egger	0.828232926	0.05	0.420795968
		Weighted median	0.477108473	0.05	1
		Inverse variance weighted	0.965811914	0.05	1
		Simple mode	0.571574149	0.05	0.999999981
		Weighted mode	0.592653509	0.05	1
	IDP dMRI TBSS ICVF Anterior limb of internal capsule L	MR Egger	0.965610673	0.05	1
		Weighted median	0.527887636	0.05	0.999998378
		Inverse variance weighted	0.482233137	0.05	0.999999109

		Simple mode	0.662514997	0.05	0.999999196
		Weighted mode	0.646132621	0.05	0.999996985
	IDP dMRI TBSS ICVF Body of corpus callosum	MR Egger	0.889407881	0.05	0.673435846
		Weighted median	0.210367083	0.05	1
		Inverse variance weighted	0.26957535	0.05	1
		Simple mode	0.437180326	0.05	1
		Weighted mode	0.296770633	0.05	1
	IDP dMRI TBSS ICVF Cerebral peduncle R	MR Egger	0.460356123	0.05	1
		Weighted median	0.358322196	0.05	1
		Inverse variance weighted	0.880833342	0.05	1
		Simple mode	0.402098952	0.05	1
		Weighted mode	0.369130341	0.05	1
	IDP dMRI TBSS ICVF Cingulum hippocampus L	MR Egger	0.372826616	0.05	1
		Weighted median	0.23474972	0.05	0.999990473
		Inverse variance weighted	0.24113482	0.05	0.999993377
		Simple mode	0.163669543	0.05	0.081801448
		Weighted mode	0.319279166	0.05	0.98359185
	IDP dMRI TBSS ICVF Cingulum hippocampus R	MR Egger	0.610169512	0.05	1
		Weighted median	0.934832301	0.05	1
		Inverse variance weighted	0.498919648	0.05	1
		Simple mode	0.801046801	0.05	1
		Weighted mode	0.790061942	0.05	1

	IDP dMRI TBSS ICVF External capsule R	MR Egger	0.304953783	0.05	1
		Weighted median	0.938389588	0.05	1
		Inverse variance weighted	0.936930971	0.05	1
		Simple mode	0.419971788	0.05	1
		Weighted mode	0.500793003	0.05	0.999830037
	IDP dMRI TBSS ICVF Genu of corpus callosum	MR Egger	0.300243708	0.05	1
		Weighted median	0.891999062	0.05	1
		Inverse variance weighted	0.904788738	0.05	1
		Simple mode	0.388109891	0.05	1
		Weighted mode	0.419216622	0.05	0.964854402
	IDP dMRI TBSS ICVF Inferior cerebellar peduncle L	MR Egger	0.69756455	0.05	1
		Weighted median	0.426555638	0.05	1
		Inverse variance weighted	0.433112251	0.05	1
		Simple mode	0.679355424	0.05	1
		Weighted mode	0.496018716	0.05	1
	IDP dMRI TBSS ICVF Inferior cerebellar peduncle R	MR Egger	0.834185209	0.05	0.800898337
		Weighted median	0.324982315	0.05	1
		Inverse variance weighted	0.662848246	0.05	1
		Simple mode	0.441925046	0.05	1
		Weighted mode	0.370340019	0.05	1
	IDP dMRI TBSS ICVF Posterior corona radiata L	MR Egger	0.887811496	0.05	1
		Weighted median	0.282453911	0.05	1

		Inverse variance weighted	0.125085005	0.05	1
		Simple mode	0.522896589	0.05	1
		Weighted mode	0.379742352	0.05	1
	IDP dMRI TBSS ICVF Posterior corona radiata R	MR Egger	0.399901645	0.05	1
		Weighted median	0.184340655	0.05	0.996132095
		Inverse variance weighted	0.305089752	0.05	0.999853117
		Simple mode	0.721286171	0.05	1
		Weighted mode	0.233955254	0.05	0.204625176
	IDP dMRI TBSS ICVF Posterior limb of internal capsule L	MR Egger	0.26857089	0.05	1
		Weighted median	0.441781669	0.05	1
		Inverse variance weighted	0.599214654	0.05	1
		Simple mode	0.263267034	0.05	0.110890832
		Weighted mode	0.565785049	0.05	1
	IDP dMRI TBSS ICVF Posterior limb of internal capsule R	MR Egger	0.466204616	0.05	1
		Weighted median	0.434236507	0.05	1
		Inverse variance weighted	0.746372399	0.05	1
		Simple mode	0.586911815	0.05	1
		Weighted mode	0.396836636	0.05	1
	IDP dMRI TBSS ICVF Posterior thalamic radiation L	MR Egger	0.26513984	0.05	1
		Weighted median	0.647428676	0.05	1
		Inverse variance weighted	0.68992799	0.05	1
		Simple mode	0.315004856	0.05	0.301037606

		Weighted mode	0.657695571	0.05	1
	IDP dMRI TBSS ICVF Retrolenticular part of internal capsule L	MR Egger	0.264896342	0.05	1
		Weighted median	0.536025198	0.05	1
		Inverse variance weighted	0.626168359	0.05	1
		Simple mode	0.2933315	0.05	0.262740955
		Weighted mode	0.704653997	0.05	1
	IDP dMRI TBSS ICVF Retrolenticular part of internal capsule R	MR Egger	0.266002843	0.05	1
		Weighted median	0.571293903	0.05	1
		Inverse variance weighted	0.657268278	0.05	1
		Simple mode	0.29946352	0.05	0.189156466
		Weighted mode	0.606127713	0.05	1
	IDP dMRI TBSS ICVF Sagittal stratum L	MR Egger	0.265303303	0.05	1
		Weighted median	0.523045234	0.05	1
		Inverse variance weighted	0.607485644	0.05	1
		Simple mode	0.292884227	0.05	0.28695589
		Weighted mode	0.78729285	0.05	1
	IDP dMRI TBSS ICVF Sagittal stratum R	MR Egger	0.264832521	0.05	1
		Weighted median	0.536280616	0.05	1
		Inverse variance weighted	0.621163607	0.05	1
		Simple mode	0.292059626	0.05	0.230898552
		Weighted mode	0.732488708	0.05	1
		MR Egger	0.383839223	0.05	1

	IDP dMRI TBSS ICVF Splenium of corpus callosum	Weighted median	0.891157242	0.05	1
		Inverse variance weighted	0.820224455	0.05	1
		Simple mode	0.525584654	0.05	0.999997358
		Weighted mode	0.590743241	0.05	1
	IDP dMRI TBSS ICVF Superior cerebellar peduncle R	MR Egger	0.400069931	0.05	1
		Weighted median	0.899224595	0.05	1
		Inverse variance weighted	0.837121409	0.05	1
		Simple mode	0.514743778	0.05	0.999985971
		Weighted mode	0.54533379	0.05	1
	IDP dMRI TBSS ICVF Superior corona radiata L	MR Egger	0.803370812	0.05	1
		Weighted median	0.427997288	0.05	1
		Inverse variance weighted	0.49452992	0.05	1
		Simple mode	0.724401561	0.05	1
		Weighted mode	0.389462621	0.05	0.999891978
	IDP dMRI TBSS ICVF Superior corona radiata R	MR Egger	0.698963716	0.05	1
		Weighted median	0.763015037	0.05	1
		Inverse variance weighted	0.486395207	0.05	1
		Simple mode	0.87054442	0.05	1
		Weighted mode	0.319795103	0.05	0.995981145
	IDP dMRI TBSS ICVF Superior longitudinal fasciculus L	MR Egger	0.780094402	0.05	1
		Weighted median	0.593510254	0.05	1
		Inverse variance weighted	0.573357698	0.05	1

		Simple mode	0.9241918	0.05	1
		Weighted mode	0.485988928	0.05	0.99980015
	IDP dMRI TBSS ICVF Superior longitudinal fasciculus R	MR Egger	0.556771243	0.05	1
		Weighted median	0.34811266	0.05	1
		Inverse variance weighted	0.393244314	0.05	1
		Simple mode	0.562461536	0.05	1
		Weighted mode	0.467630829	0.05	1
	IDP dMRI TBSS L1 External capsule L	MR Egger	0.506381602	0.05	1
		Weighted median	0.358984801	0.05	1
		Inverse variance weighted	0.864530974	0.05	1
		Simple mode	0.695848337	0.05	1
		Weighted mode	0.436588984	0.05	1
	IDP dMRI TBSS L2 Genu of corpus callosum	MR Egger	0.266071128	0.05	1
		Weighted median	0.728010404	0.05	1
		Inverse variance weighted	0.733088433	0.05	1
		Simple mode	0.301240221	0.05	0.224341769
		Weighted mode	0.583339094	0.05	1
	IDP dMRI TBSS L2 Splenium of corpus callosum	MR Egger	0.371180127	0.05	1
		Weighted median	0.075470337	0.05	0.898075968
		Inverse variance weighted	0.200804332	0.05	0.983580541
		Simple mode	0.266977008	0.05	0.448640032
		Weighted mode	0.239341976	0.05	0.779835626

	IDP dMRI TBSS L2 Superior corona radiata R	MR Egger	0.267999301	0.05	1
		Weighted median	0.545833757	0.05	1
		Inverse variance weighted	0.621990643	0.05	1
		Simple mode	0.337284184	0.05	0.644288518
		Weighted mode	0.859447928	0.05	1
	IDP dMRI TBSS MD Cingulum cingulate gyrus L	MR Egger	0.687117285	0.05	0.010602547
		Weighted median	0.620464718	0.05	1
		Inverse variance weighted	0.561717453	0.05	1
		Simple mode	0.860537266	0.05	1
		Weighted mode	0.689539066	0.05	1
	IDP dMRI TBSS MD Cingulum cingulate gyrus R	MR Egger	0.31669197	0.05	1
		Weighted median	0.363231487	0.05	1
		Inverse variance weighted	0.572421131	0.05	1
		Simple mode	0.775070229	0.05	1
		Weighted mode	0.523933045	0.05	1
	IDP dMRI TBSS MO Pontine crossing tract	MR Egger	0.331450981	0.05	0.003268516
		Weighted median	0.450847178	0.05	1
		Inverse variance weighted	0.865537549	0.05	1
		Simple mode	0.548287139	0.05	1
		Weighted mode	0.508357965	0.05	1
	IDP dMRI TBSS OD Pontine crossing tract	MR Egger	0.364233468	0.05	0.002556669
		Weighted median	0.882347424	0.05	1

		Inverse variance weighted	0.786254777	0.05	1
		Simple mode	0.599407786	0.05	0.999999981
		Weighted mode	0.833341844	0.05	1
	IDP SWI T2star left caudate	MR Egger	0.26483864	0.05	0.002790813
		Weighted median	0.421992675	0.05	1
		Inverse variance weighted	0.562312834	0.05	1
		Simple mode	0.274209353	0.05	1
		Weighted mode	0.7735703	0.05	1
	IDP SWI T2star left caudate plus IDP SWI T2star right caudate	MR Egger	0.42319266	0.05	1
		Weighted median	0.62526986	0.05	1
		Inverse variance weighted	0.938419279	0.05	1
		Simple mode	0.950744027	0.05	1
		Weighted mode	0.535400673	0.05	1
	IDP SWI T2star left pallidum	MR Egger	0.394616228	0.05	1
		Weighted median	0.619324243	0.05	1
		Inverse variance weighted	0.935281362	0.05	1
		Simple mode	0.934140888	0.05	1
		Weighted mode	0.525465157	0.05	1
	IDP SWI T2star left pallidum plus IDP SWI T2star right pallidum	MR Egger	0.269148628	0.05	1
		Weighted median	0.641258032	0.05	1
		Inverse variance weighted	0.708466545	0.05	1
		Simple mode	0.308898137	0.05	0.087020502

		Weighted mode	0.588536955	0.05	1
	IDP SWI T2star left putamen	MR Egger	0.269970442	0.05	1
		Weighted median	0.576677549	0.05	1
		Inverse variance weighted	0.682663145	0.05	1
		Simple mode	0.26497852	0.05	0.081958291
		Weighted mode	0.524377281	0.05	1
	IDP SWI T2star left putamen plus IDP SWI T2star right putamen	MR Egger	0.270155053	0.05	1
		Weighted median	0.602178528	0.05	1
		Inverse variance weighted	0.709582566	0.05	1
		Simple mode	0.280199767	0.05	0.170137153
		Weighted mode	0.518222244	0.05	1
	IDP SWI T2star left thalamus plus IDP SWI T2star right thalamus	MR Egger	0.399657781	0.05	1
		Weighted median	0.717156548	0.05	1
		Inverse variance weighted	0.858922165	0.05	1
		Simple mode	0.917653812	0.05	1
		Weighted mode	0.530183694	0.05	1
	IDP SWI T2star right caudate	MR Egger	0.30745945	0.05	1
		Weighted median	0.988931779	0.05	1
		Inverse variance weighted	0.543110471	0.05	1
		Simple mode	0.789885206	0.05	1
		Weighted mode	0.649526774	0.05	1
	IDP SWI T2star right pallidum	MR Egger	0.489402632	0.05	1

		Weighted median	0.797097066	0.05	1
		Inverse variance weighted	0.848262055	0.05	1
		Simple mode	0.575603276	0.05	1
		Weighted mode	0.59984435	0.05	1
	IDP SWI T2star right putamen	MR Egger	0.24291158	0.05	1
		Weighted median	0.945129183	0.05	1
		Inverse variance weighted	0.696504018	0.05	1
		Simple mode	0.767421327	0.05	1
		Weighted mode	0.465206848	0.05	1
	IDP T1 FAST ROIs L cerebellum crus I	MR Egger	0.416676808	0.05	1
		Weighted median	0.791282454	0.05	1
		Inverse variance weighted	0.746854178	0.05	1
		Simple mode	0.487734606	0.05	1
		Weighted mode	0.256547111	0.05	0.699847037
	IDP T1 FAST ROIs L cerebellum crus II	MR Egger	0.264848085	0.05	1
		Weighted median	0.563779059	0.05	1
		Inverse variance weighted	0.644339677	0.05	1
		Simple mode	0.297667561	0.05	0.457406872
		Weighted mode	0.719412313	0.05	1
	IDP T1 FAST ROIs V cerebellum VIIIa	MR Egger	0.355830509	0.05	1
		Weighted median	0.126122425	0.05	0.891350406
		Inverse variance weighted	0.240133486	0.05	0.981765175

		Simple mode	0.278920523	0.05	0.291855021
		Weighted mode	0.287005263	0.05	0.664643743
	IDP T1 SIENAX CSF unnormalised volume	MR Egger	0.26730792	0.05	1
		Weighted median	0.494240449	0.05	1
		Inverse variance weighted	0.616995753	0.05	1
		Simple mode	0.293890265	0.05	0.172336536
		Weighted mode	0.604973968	0.05	1
	volume CSF	MR Egger	0.265076312	0.05	1
		Weighted median	0.530882241	0.05	1
		Inverse variance weighted	0.623451308	0.05	1
		Simple mode	0.291081413	0.05	0.176280416
		Weighted mode	0.698729374	0.05	1
	volume Left-Lateral-Ventricle	MR Egger	0.987817427	0.05	1
		Weighted median	0.902639817	0.05	1
		Inverse variance weighted	0.932768696	0.05	1
		Simple mode	0.967260233	0.05	1
		Weighted mode	0.855332457	0.05	1
	volume Right-Cerebellum-Cortex	MR Egger	0.889748887	0.05	1
		Weighted median	0.694751744	0.05	1
		Inverse variance weighted	0.437551084	0.05	0.999999999
		Simple mode	0.87685201	0.05	1
		Weighted mode	0.857596685	0.05	1

Connective tissue disorder	IDP T1 SIENAX peripheral grey normalised volume	Wald ratio	0.811065505	0.05	1
	IDP T1 FAST ROIs R temp occ fusif cortex	Wald ratio	0.921712525	0.05	1
	IDP T1 FAST ROIs R occ pole	Inverse variance weighted	0.562332582	0.05	1
	IDP T1 FAST ROIs L thalamus	Inverse variance weighted	0.971716755	0.05	1
	IDP T1 FAST ROIs R thalamus	Wald ratio	0.211686724	0.05	1
	IDP T1 FAST ROIs L caudate	Wald ratio	0.653194421	0.05	1
	IDP T1 FAST ROIs R caudate	Wald ratio	0.653194421	0.05	1
	IDP T1 FAST ROIs L putamen	Wald ratio	0.813349769	0.05	1
	IDP T1 FAST ROIs R putamen	Wald ratio	0.813349769	0.05	1
	IDP T1 FAST ROIs L pallidum	Wald ratio	0.196863045	0.05	1
	IDP T1 FIRST left caudate volume	Wald ratio	0.687003229	0.05	1
	IDP T1 FAST ROIs L hippocampus	Inverse variance weighted	0.374468944	0.05	1
	IDP T1 FAST ROIs R hippocampus	Wald ratio	0.519747415	0.05	1
	IDP T1 FAST ROIs L ventral striatum	Wald ratio	0.813349769	0.05	1
	IDP T1 FAST ROIs R ventral striatum	Inverse variance weighted	0.944181877	0.05	1
	IDP T1 FAST ROIs L cerebellum VI	Wald ratio	0.511659327	0.05	1
	IDP T1 FAST ROIs L cerebellum crus I	MR Egger	0.546903611	0.05	1
		Weighted median	0.690104413	0.05	1
		Inverse variance weighted	0.778481319	0.05	1
		Simple mode	0.709380378	0.05	1
		Weighted mode	0.649659796	0.05	1
	IDP T1 FAST ROIs R cerebellum crus I	Inverse variance weighted	0.714210273	0.05	1

	IDP T1 FAST ROIs L cerebellum crus II	MR Egger	0.542853913	0.05	1
		Weighted median	0.840085807	0.05	1
		Inverse variance weighted	0.90640699	0.05	1
		Simple mode	0.725532536	0.05	1
		Weighted mode	0.745430663	0.05	1
	IDP T1 FAST ROIs V cerebellum crus II	Inverse variance weighted	0.238829803	0.05	1
	IDP T1 FAST ROIs R cerebellum crus II	Inverse variance weighted	0.832343843	0.05	1
	IDP T1 FIRST left putamen volume	Wald ratio	0.216625059	0.05	1
	IDP T1 FAST ROIs R cerebellum VIIb	Wald ratio	0.833776502	0.05	1
	IDP T1 FAST ROIs V cerebellum VIIIa	MR Egger	0.55797987	0.05	1
		Weighted median	0.67090494	0.05	1
		Inverse variance weighted	0.422348457	0.05	1
		Simple mode	0.796449518	0.05	1
		Weighted mode	0.808258819	0.05	1
	IDP T1 FAST ROIs L cerebellum VIIIb	Wald ratio	0.8868007	0.05	1
	IDP T1 FAST ROIs V cerebellum VIIIb	Inverse variance weighted	0.828626422	0.05	1
	IDP T1 FAST ROIs R cerebellum VIIIb	Wald ratio	0.813349769	0.05	1
	IDP T1 FAST ROIs L cerebellum IX	Inverse variance weighted	0.369322293	0.05	1
	IDP T1 FAST ROIs V cerebellum IX	Inverse variance weighted	0.671353584	0.05	1
	IDP T1 FAST ROIs R cerebellum IX	Inverse variance weighted	0.452318374	0.05	1
	IDP T1 FAST ROIs V cerebellum X	Wald ratio	0.813349769	0.05	1

	IDP T2 FLAIR BIANCA WMH volume	MR Egger	0.768904775	0.05	1
		Weighted median	0.601196594	0.05	1
		Inverse variance weighted	0.590535805	0.05	1
		Simple mode	0.592513463	0.05	1
		Weighted mode	0.921367623	0.05	1
	IDP SWI T2star left caudate	MR Egger	0.768417359	0.05	1
		Weighted median	0.99892722	0.05	1
		Inverse variance weighted	0.994882684	0.05	1
		Simple mode	0.969288554	0.05	1
		Weighted mode	0.992286439	0.05	1
	IDP SWI T2star right caudate	MR Egger	0.414841536	0.05	1
		Weighted median	0.429216325	0.05	1
		Inverse variance weighted	0.49153152	0.05	1
		Simple mode	0.666517179	0.05	1
		Weighted mode	0.258331658	0.05	1
	IDP T1 FIRST left pallidum volume	Wald ratio	0.650613107	0.05	1
	IDP SWI T2star left putamen	MR Egger	0.855105726	0.05	1
		Weighted median	0.558265797	0.05	1
		Inverse variance weighted	0.975484372	0.05	1
		Simple mode	0.639281122	0.05	1
		Weighted mode	0.759489682	0.05	1
	IDP SWI T2star right putamen	MR Egger	0.440453003	0.05	1

		Weighted median	0.675320355	0.05	1
		Inverse variance weighted	0.291565227	0.05	1
		Simple mode	0.998923102	0.05	1
		Weighted mode	0.875112411	0.05	1
	IDP SWI T2star left pallidum	MR Egger	0.700611865	0.05	1
		Weighted median	0.509516809	0.05	1
		Inverse variance weighted	0.542453602	0.05	1
		Simple mode	0.543378823	0.05	1
		Weighted mode	0.616433994	0.05	1
	IDP SWI T2star right pallidum	MR Egger	0.709598837	0.05	1
		Weighted median	0.928151188	0.05	1
		Inverse variance weighted	0.695024038	0.05	1
		Simple mode	0.57238543	0.05	1
		Weighted mode	0.650101229	0.05	1
	IDP T1 FIRST right pallidum volume	Wald ratio	0.650613107	0.05	1
	IDP dMRI TBSS FA Genu of corpus callosum	Wald ratio	0.0868087	0.05	1
	IDP dMRI TBSS FA Splenium of corpus callosum	MR Egger	0.234057434	0.05	1
		Weighted median	0.700091768	0.05	1
		Inverse variance weighted	0.906563346	0.05	1
		Simple mode	0.863140179	0.05	1
		Weighted mode	0.635550612	0.05	1
	IDP dMRI TBSS FA Corticospinal tract L	Wald ratio	0.105158955	0.05	1

	IDP dMRI TBSS FA Superior cerebellar peduncle R	Inverse variance weighted	0.762215198	0.05	1
	IDP dMRI TBSS FA Superior cerebellar peduncle L	Inverse variance weighted	0.772997265	0.05	1
	IDP dMRI TBSS FA Posterior limb of internal capsule R	Wald ratio	0.484528998	0.05	1
	IDP dMRI TBSS FA Retrolenticular part of internal capsule R	Inverse variance weighted	0.301702105	0.05	1
	IDP dMRI TBSS FA Retrolenticular part of internal capsule L	Wald ratio	0.060854008	0.05	1
	IDP dMRI TBSS FA Anterior corona radiata R	Wald ratio	0.0868087	0.05	1
	IDP dMRI TBSS FA Anterior corona radiata L	Wald ratio	0.0868087	0.05	1
	IDP dMRI TBSS FA Posterior corona radiata L	Wald ratio	0.060854008	0.05	1
	IDP dMRI TBSS FA Posterior thalamic radiation R	Inverse variance weighted	0.189544433	0.05	1
	IDP dMRI TBSS FA Posterior thalamic radiation L	Inverse variance weighted	0.152486928	0.05	1
	IDP dMRI TBSS FA Sagittal stratum R	Inverse variance weighted	0.429818006	0.05	1
	IDP dMRI TBSS FA Sagittal stratum L	Wald ratio	0.079481048	0.05	1
	IDP dMRI TBSS FA Cingulum cingulate gyrus R	Inverse variance weighted	0.175468734	0.05	1
	IDP dMRI TBSS FA Superior longitudinal fasciculus L	Inverse variance weighted	0.837558526	0.05	1
	IDP dMRI TBSS FA Uncinate fasciculus L	Wald ratio	0.460323682	0.05	1
	IDP dMRI TBSS MD Genu of corpus callosum	Wald ratio	0.0868087	0.05	1
	IDP dMRI TBSS MD Body of corpus callosum	Wald ratio	0.302568256	0.05	1
	IDP dMRI TBSS MD Splenium of corpus callosum	Inverse variance weighted	0.30083417	0.05	1
	IDP dMRI TBSS MD Corticospinal tract R	Wald ratio	0.701871649	0.05	1

	IDP dMRI TBSS MD Inferior cerebellar peduncle R	Inverse variance weighted	0.717564179	0.05	1
	IDP dMRI TBSS MD Inferior cerebellar peduncle L	Wald ratio	0.060854008	0.05	1
	IDP dMRI TBSS MD Superior cerebellar peduncle R	Wald ratio	0.956682418	0.05	1
	IDP dMRI TBSS MD Superior cerebellar peduncle L	Wald ratio	0.83188146	0.05	1
	IDP dMRI TBSS MD Anterior limb of internal capsule L	Wald ratio	0.559220736	0.05	1
	IDP dMRI TBSS MD Retrolenticular part of internal capsule R	Inverse variance weighted	0.263604648	0.05	1
	IDP T1 FIRST left caudate volume plus IDP T1 FIRST right caudate volume	Wald ratio	0.891150088	0.05	1
	IDP T1 FIRST left putamen volume plus IDP T1 FIRST right putamen volume	Wald ratio	0.143405184	0.05	1
	IDP T1 FIRST left pallidum volume plus IDP T1 FIRST right pallidum volume	Wald ratio	0.650613107	0.05	1
	IDP SWI T2star left thalamus plus IDP SWI T2star right thalamus	MR Egger	0.775339405	0.05	1
		Weighted median	0.179002685	0.05	1
		Inverse variance weighted	0.032847132	0.05	1
		Simple mode	0.456809868	0.05	1
		Weighted mode	0.426832966	0.05	1
	IDP dMRI TBSS MD Retrolenticular part of internal capsule L	Inverse variance weighted	0.21944033	0.05	1
	IDP SWI T2star left caudate plus IDP SWI T2star right caudate	MR Egger	0.347982152	0.05	1
		Weighted median	0.953674082	0.05	1
		Inverse variance weighted	0.613480758	0.05	1
		Simple mode	0.576795786	0.05	1
		Weighted mode	0.560014289	0.05	1

	IDP SWI T2star left putamen plus IDP SWI T2star right putamen	MR Egger	0.529468476	0.05	1
		Weighted median	0.458651324	0.05	1
		Inverse variance weighted	0.830488824	0.05	1
		Simple mode	0.70362244	0.05	1
		Weighted mode	0.715672973	0.05	1
	IDP SWI T2star left pallidum plus IDP SWI T2star right pallidum	MR Egger	0.539504763	0.05	1
		Weighted median	0.611599474	0.05	1
		Inverse variance weighted	0.806874689	0.05	1
		Simple mode	0.503441822	0.05	1
		Weighted mode	0.61465831	0.05	1
	volume Left-Lateral-Ventricle	MR Egger	0.725290956	0.05	1
		Weighted median	0.756233355	0.05	1
		Inverse variance weighted	0.857631791	0.05	1
		Simple mode	0.76216977	0.05	1
		Weighted mode	0.735311084	0.05	1
	volume Left-Inf-Lat-Vent	Wald ratio	0.719352649	0.05	1
	volume Left-Cerebellum-White-Matter	Inverse variance weighted	0.849509761	0.05	1
	volume Left-Cerebellum-Cortex	Inverse variance weighted	0.963307303	0.05	1
	IDP dMRI TBSS MD Anterior corona radiata R	Inverse variance weighted	0.22467392	0.05	1
	volume Left-Putamen	Wald ratio	0.393296755	0.05	1
	volume 3rd-Ventricle	Wald ratio	0.567255315	0.05	1
	volume 4th-Ventricle	Inverse variance weighted	0.73828744	0.05	1

	volume Brain-Stem	Inverse variance weighted	0.356515403	0.05	1
	volume Left-Hippocampus	Inverse variance weighted	0.915974913	0.05	1
	volume CSF	MR Egger	0.544785117	0.05	1
		Weighted median	0.632961156	0.05	1
		Inverse variance weighted	0.580120696	0.05	1
		Simple mode	0.81801566	0.05	1
		Weighted mode	0.87550649	0.05	1
	IDP dMRI TBSS MD Anterior corona radiata L	Inverse variance weighted	0.182691933	0.05	1
	volume Left-Accumbens-area	Wald ratio	0.813349769	0.05	1
	volume Right-Lateral-Ventricle	Inverse variance weighted	0.964151397	0.05	1
	volume Right-Inf-Lat-Vent	Wald ratio	0.545615261	0.05	1
	volume Right-Cerebellum-White-Matter	Wald ratio	0.489435012	0.05	1
	volume Right-Cerebellum-Cortex	MR Egger	0.709671382	0.05	1
		Weighted median	0.815414071	0.05	1
		Inverse variance weighted	0.828619587	0.05	1
		Simple mode	0.909097749	0.05	1
		Weighted mode	0.766644891	0.05	1
	IDP dMRI TBSS MD Superior corona radiata R	Inverse variance weighted	0.22383081	0.05	1
	volume Right-Hippocampus	Wald ratio	0.519747415	0.05	1
	volume Right-Amygdala	Wald ratio	0.10231908	0.05	1
	volume Right-VentralDC	Wald ratio	0.289188986	0.05	1
	volume CC Posterior	Wald ratio	0.985226292	0.05	1

	IDP dMRI TBSS MD Superior corona radiata L	Inverse variance weighted	0.219309458	0.05	1
	IDP dMRI TBSS MD Posterior corona radiata R	Inverse variance weighted	0.202320319	0.05	1
	volume BrainSegVol-to-eTIV	Wald ratio	0.425372897	0.05	1
	IDP dMRI TBSS MD Posterior corona radiata L	Inverse variance weighted	0.134094618	0.05	1
	volume rhSurfaceHoles	Wald ratio	0.830395563	0.05	1
	DKTatlas lh cuneus area	Wald ratio	0.896198827	0.05	1
	IDP dMRI TBSS MD Posterior thalamic radiation R	Inverse variance weighted	0.183440773	0.05	1
	DKTatlas lh lateraloccipital area	Wald ratio	0.896198827	0.05	1
	DKTatlas lh lingual area	Wald ratio	0.930138155	0.05	1
	IDP dMRI TBSS MD Posterior thalamic radiation L	Inverse variance weighted	0.227601791	0.05	1
	DKTatlas lh parstriangularis area	Wald ratio	0.829199549	0.05	1
	DKTatlas lh pericalcarine area	Inverse variance weighted	0.819432325	0.05	1
	DKTatlas lh postcentral area	Inverse variance weighted	0.177338091	0.05	1
	DKTatlas lh posteriorcingulate area	Wald ratio	0.539840696	0.05	1
	DKTatlas lh precentral area	Wald ratio	0.338535661	0.05	1
	DKTatlas lh precuneus area	Wald ratio	0.909886531	0.05	1
	IDP dMRI TBSS MD Sagittal stratum R	Inverse variance weighted	0.249387357	0.05	1
	DKTatlas lh superiorparietal area	Wald ratio	0.572549941	0.05	1
	DKTatlas lh superiortemporal area	Wald ratio	0.202769266	0.05	1
	DKTatlas lh supramarginal area	Wald ratio	0.095863817	0.05	1
	DKTatlas lh WhiteSurfArea area	Wald ratio	0.77212765	0.05	1

	IDP dMRI TBSS MD Sagittal stratum L	Inverse variance weighted	0.147241	0.05	1
	a2009s lh G&S subcentral area	Wald ratio	0.338535661	0.05	1
	a2009s lh G cuneus area	Wald ratio	0.79076033	0.05	1
	a2009s lh G front inf-Opercular area	Wald ratio	0.168880744	0.05	1
	IDP dMRI TBSS MD External capsule R	Wald ratio	0.0868087	0.05	1
	IDP dMRI TBSS MD External capsule L	Inverse variance weighted	0.08544634	0.05	1
	a2009s lh G pariet inf-Supramar area	Wald ratio	0.095863817	0.05	1
	a2009s lh G parietal sup area	Wald ratio	0.587808337	0.05	1
	a2009s lh G postcentral area	Wald ratio	0.597287732	0.05	1
	a2009s lh G precentral area	Wald ratio	0.338535661	0.05	1
	a2009s lh G precuneus area	Wald ratio	0.79076033	0.05	1
	IDP dMRI TBSS MD Cingulum cingulate gyrus R	MR Egger	0.423448841	0.05	1
		Weighted median	0.135305399	0.05	1
		Inverse variance weighted	0.127134653	0.05	1
		Simple mode	0.430929304	0.05	1
		Weighted mode	0.274280477	0.05	1
	IDP dMRI TBSS MD Cingulum cingulate gyrus L	MR Egger	0.422410501	0.05	1
		Weighted median	0.134766836	0.05	1
		Inverse variance weighted	0.126624458	0.05	1
		Simple mode	0.406557356	0.05	1
		Weighted mode	0.267215259	0.05	1
	a2009s lh S calcarine area	Wald ratio	0.930138155	0.05	1

	a2009s lh S central area	Wald ratio	0.333917589	0.05	1
	a2009s lh S collat transv ant area	Wald ratio	0.413838739	0.05	1
	IDP dMRI TBSS MD Cingulum hippocampus R	Inverse variance weighted	0.149098267	0.05	1
	a2009s lh S intrapariet&P trans area	Wald ratio	0.587808337	0.05	1
	IDP dMRI TBSS MD Cingulum hippocampus L	Inverse variance weighted	0.144088739	0.05	1
	a2009s lh S subparietal area	Wald ratio	0.930138155	0.05	1
	DKTatlas rh cuneus area	Wald ratio	0.930138155	0.05	1
	DKTatlas rh lateraloccipital area	Inverse variance weighted	0.221253667	0.05	1
	DKTatlas rh lingual area	Wald ratio	0.963994404	0.05	1
	DKTatlas rh parstriangularis area	Wald ratio	0.948295912	0.05	1
	DKTatlas rh pericalcarine area	Inverse variance weighted	0.82107012	0.05	1
	IDP dMRI TBSS MD Superior longitudinal fasciculus R	Inverse variance weighted	0.271330304	0.05	1
	DKTatlas rh postcentral area	Wald ratio	0.333917589	0.05	1
	DKTatlas rh precentral area	Wald ratio	0.338535661	0.05	1
	IDP dMRI TBSS MD Superior longitudinal fasciculus L	Inverse variance weighted	0.27019476	0.05	1
	a2009s rh G&S subcentral area	Wald ratio	0.333917589	0.05	1
	a2009s rh G&S cingul-Mid-Post area	Wald ratio	0.836782846	0.05	1
	a2009s rh G cuneus area	Wald ratio	0.930138155	0.05	1
	IDP dMRI TBSS MD Superior fronto-occipital fasciculus L	Wald ratio	0.800966226	0.05	1
	a2009s rh G oc-temp med-Lingual area	Wald ratio	0.963994404	0.05	1
	a2009s rh G parietal sup area	Wald ratio	0.539535807	0.05	1

	IDP dMRI TBSS MD Uncinate fasciculus R	Inverse variance weighted	0.056671006	0.05	1
	a2009s rh G precentral area	Wald ratio	0.338535661	0.05	1
	a2009s rh G precuneus area	Wald ratio	0.373558339	0.05	1
	IDP dMRI TBSS MD Uncinate fasciculus L	Inverse variance weighted	0.075136216	0.05	1
	a2009s rh Pole occipital area	Wald ratio	0.930138155	0.05	1
	a2009s rh S calcarine area	Wald ratio	0.963994404	0.05	1
	a2009s rh S central area	Wald ratio	0.338535661	0.05	1
	a2009s rh S collat transv ant area	Wald ratio	0.413838739	0.05	1
	a2009s rh S orbital med-olfact area	Wald ratio	0.177210795	0.05	1
	IDP dMRI TBSS MO Pontine crossing tract	MR Egger	0.400969885	0.05	1
		Weighted median	0.902933728	0.05	1
		Inverse variance weighted	0.569093107	0.05	1
		Simple mode	0.920270469	0.05	1
		Weighted mode	0.873410335	0.05	1
	DKTatlas lh postcentral thickness	Wald ratio	0.333917589	0.05	1
	a2009s lh G insular short thickness	Wald ratio	0.553583489	0.05	1
	a2009s lh G postcentral thickness	Wald ratio	0.333917589	0.05	1
	IDP T1 SIENAX CSF normalised volume	Inverse variance weighted	0.926399197	0.05	1
	IDP dMRI TBSS MO Medial lemniscus R	Wald ratio	0.140823345	0.05	1
	a2009s lh S postcentral thickness	Wald ratio	0.479138329	0.05	1
	DKTatlas rh lateraloccipital thickness	Wald ratio	0.813349769	0.05	1
	DKTatlas rh parstriangularis thickness	Wald ratio	0.831277991	0.05	1

	DKTatlas rh postcentral thickness	Wald ratio	0.333917589	0.05	1
	DKTatlas rh posteriorcingulate thickness	Wald ratio	0.034086201	0.05	1
	a2009s rh G&S cingul-Mid-Ant thickness	Wald ratio	0.981668159	0.05	1
	a2009s rh G cuneus thickness	Wald ratio	0.813349769	0.05	1
	a2009s rh G postcentral thickness	Wald ratio	0.521196454	0.05	1
	a2009s rh Pole occipital thickness	Wald ratio	0.813349769	0.05	1
	a2009s rh S circular insula ant thickness	Wald ratio	0.876795808	0.05	1
	a2009s rh S oc sup&transversal thickness	Wald ratio	0.252811684	0.05	1
	IDP dMRI TBSS MO Anterior corona radiata R	Wald ratio	0.97601718	0.05	1
	IDP dMRI TBSS MO Superior corona radiata R	Wald ratio	0.313299659	0.05	1
	IDP dMRI TBSS MO Cingulum cingulate gyrus L	Inverse variance weighted	0.658745641	0.05	1
	IDP dMRI TBSS L1 Genu of corpus callosum	Wald ratio	0.079481048	0.05	1
	IDP dMRI TBSS L1 Anterior limb of internal capsule L	Wald ratio	0.559220736	0.05	1
	IDP dMRI TBSS L1 Posterior limb of internal capsule R	Wald ratio	0.309672333	0.05	1
	IDP dMRI TBSS L1 Posterior limb of internal capsule L	Wald ratio	0.69316456	0.05	1
	IDP dMRI TBSS L1 Anterior corona radiata R	Wald ratio	0.0868087	0.05	1
	IDP dMRI TBSS L1 Anterior corona radiata L	Wald ratio	0.0868087	0.05	1
	IDP dMRI TBSS L1 Posterior corona radiata R	Wald ratio	0.276112618	0.05	1
	IDP dMRI TBSS L1 Posterior corona radiata L	Wald ratio	0.0868087	0.05	1
	IDP dMRI TBSS L1 Sagittal stratum L	Wald ratio	0.0868087	0.05	1

	IDP dMRI TBSS L1 External capsule R	Wald ratio	0.0868087	0.05	1
	IDP dMRI TBSS L1 External capsule L	MR Egger	0.744865196	0.05	1
		Weighted median	0.432500467	0.05	1
		Inverse variance weighted	0.697737812	0.05	1
		Simple mode	0.51952488	0.05	1
		Weighted mode	0.452657249	0.05	1
	IDP dMRI TBSS L1 Cingulum hippocampus L	Wald ratio	0.079481048	0.05	1
	IDP dMRI TBSS L1 Fornix cres+Stria terminalis L	Wald ratio	0.479030014	0.05	1
	IDP T1 FAST ROIs L precentral gyrus	Wald ratio	0.333917589	0.05	1
	IDP dMRI TBSS L1 Superior longitudinal fasciculus R	Inverse variance weighted	0.797166516	0.05	1
	IDP dMRI TBSS L1 Uncinate fasciculus R	Wald ratio	0.060854008	0.05	1
	IDP dMRI TBSS L1 Uncinate fasciculus L	Wald ratio	0.050011707	0.05	1
	IDP dMRI TBSS L2 Pontine crossing tract	Wald ratio	0.140823345	0.05	1
	IDP dMRI TBSS L2 Genu of corpus callosum	MR Egger	0.360797176	0.05	1
		Weighted median	0.800725949	0.05	1
		Inverse variance weighted	0.343957278	0.05	1
		Simple mode	0.880651749	0.05	1
		Weighted mode	0.890941044	0.05	1
	IDP dMRI TBSS L2 Body of corpus callosum	Wald ratio	0.0868087	0.05	1
	IDP dMRI TBSS L2 Splenium of corpus callosum	MR Egger	0.123065776	0.05	1
		Weighted median	0.958903419	0.05	1
		Inverse variance weighted	0.938059413	0.05	1

		Simple mode	0.978989023	0.05	1
		Weighted mode	0.943938664	0.05	1
	IDP dMRI TBSS L2 Corticospinal tract R	Wald ratio	0.701871649	0.05	1
	IDP T1 SIENAX CSF unnormalised volume	MR Egger	0.700498684	0.05	1
		Weighted median	0.584516263	0.05	1
		Inverse variance weighted	0.776526453	0.05	1
		Simple mode	0.677463706	0.05	1
		Weighted mode	0.652145721	0.05	1
	IDP T1 FAST ROIs L temporal pole	Wald ratio	0.815981982	0.05	1
	IDP dMRI TBSS L2 Superior cerebellar peduncle R	Inverse variance weighted	0.707407917	0.05	1
	IDP dMRI TBSS L2 Superior cerebellar peduncle L	Wald ratio	0.83188146	0.05	1
	IDP dMRI TBSS L2 Posterior limb of internal capsule R	Inverse variance weighted	0.620659404	0.05	1
	IDP dMRI TBSS L2 Posterior limb of internal capsule L	Wald ratio	0.99606234	0.05	1
	IDP dMRI TBSS L2 Retrolenticular part of internal capsule R	Inverse variance weighted	0.328029582	0.05	1
	IDP dMRI TBSS L2 Retrolenticular part of internal capsule L	Wald ratio	0.060854008	0.05	1
	IDP dMRI TBSS L2 Anterior corona radiata R	Inverse variance weighted	0.218050708	0.05	1
	IDP dMRI TBSS L2 Anterior corona radiata L	Wald ratio	0.0868087	0.05	1
	IDP dMRI TBSS L2 Superior corona radiata R	MR Egger	0.48478332	0.05	1
		Weighted median	0.619379812	0.05	1
		Inverse variance weighted	0.21398771	0.05	1
		Simple mode	0.869216996	0.05	1

		Weighted mode	0.87913074	0.05	1
	IDP dMRI TBSS L2 Superior corona radiata L	Inverse variance weighted	0.295076923	0.05	1
	IDP dMRI TBSS L2 Posterior corona radiata R	Inverse variance weighted	0.230018822	0.05	1
	IDP dMRI TBSS L2 Posterior corona radiata L	Inverse variance weighted	0.210286599	0.05	1
	IDP dMRI TBSS L2 Posterior thalamic radiation R	Inverse variance weighted	0.243687351	0.05	1
	IDP dMRI TBSS L2 Posterior thalamic radiation L	Inverse variance weighted	0.184831177	0.05	1
	IDP dMRI TBSS L2 Sagittal stratum R	Inverse variance weighted	0.258252026	0.05	1
	IDP dMRI TBSS L2 Sagittal stratum L	Wald ratio	0.079481048	0.05	1
	IDP dMRI TBSS L2 Cingulum cingulate gyrus R	Wald ratio	0.0868087	0.05	1
	IDP dMRI TBSS L2 Cingulum cingulate gyrus L	Wald ratio	0.079481048	0.05	1
	IDP dMRI TBSS L2 Cingulum hippocampus R	Inverse variance weighted	0.18718782	0.05	1
	IDP dMRI TBSS L2 Cingulum hippocampus L	Wald ratio	0.0868087	0.05	1
	IDP dMRI TBSS L2 Uncinate fasciculus L	Wald ratio	0.0868087	0.05	1
	IDP dMRI TBSS L3 Middle cerebellar peduncle	Wald ratio	0.060854008	0.05	1
	IDP dMRI TBSS L3 Genu of corpus callosum	Wald ratio	0.0868087	0.05	1
	IDP dMRI TBSS L3 Body of corpus callosum	Wald ratio	0.0868087	0.05	1
	IDP dMRI TBSS L3 Splenium of corpus callosum	Inverse variance weighted	0.350518113	0.05	1
	IDP dMRI TBSS L3 Inferior cerebellar peduncle R	Wald ratio	0.0868087	0.05	1
	IDP dMRI TBSS L3 Inferior cerebellar peduncle L	Wald ratio	0.0868087	0.05	1

	IDP dMRI TBSS L3 Superior cerebellar peduncle R	Inverse variance weighted	0.710853643	0.05	1
	IDP dMRI TBSS L3 Superior cerebellar peduncle L	Wald ratio	0.83188146	0.05	1
	IDP dMRI TBSS L3 Cerebral peduncle R	Wald ratio	0.080678046	0.05	1
	IDP dMRI TBSS L3 Anterior limb of internal capsule R	Wald ratio	0.27543801	0.05	1
	IDP dMRI TBSS L3 Anterior limb of internal capsule L	Wald ratio	0.318020402	0.05	1
	IDP dMRI TBSS L3 Retrolenticular part of internal capsule R	Inverse variance weighted	0.244582028	0.05	1
	IDP dMRI TBSS L3 Retrolenticular part of internal capsule L	Wald ratio	0.060854008	0.05	1
	IDP dMRI TBSS L3 Anterior corona radiata R	Inverse variance weighted	0.220124918	0.05	1
	IDP dMRI TBSS L3 Anterior corona radiata L	Inverse variance weighted	0.173164022	0.05	1
	IDP dMRI TBSS L3 Superior corona radiata R	Wald ratio	0.079481048	0.05	1
	IDP dMRI TBSS L3 Superior corona radiata L	Wald ratio	0.079481048	0.05	1
	IDP dMRI TBSS L3 Posterior corona radiata R	Inverse variance weighted	0.8702133	0.05	1
	IDP dMRI TBSS L3 Posterior corona radiata L	Wald ratio	0.060854008	0.05	1
	IDP dMRI TBSS L3 Posterior thalamic radiation R	Inverse variance weighted	0.181355141	0.05	1
	IDP dMRI TBSS L3 Posterior thalamic radiation L	Inverse variance weighted	0.19959463	0.05	1
	IDP dMRI TBSS L3 Sagittal stratum R	Inverse variance weighted	0.203211229	0.05	1
	IDP dMRI TBSS L3 Sagittal stratum L	Inverse variance weighted	0.140385911	0.05	1
	IDP dMRI TBSS L3 External capsule R	Wald ratio	0.0868087	0.05	1
	IDP dMRI TBSS L3 External capsule L	Wald ratio	0.0868087	0.05	1

	IDP dMRI TBSS L3 Cingulum cingulate gyrus R	Inverse variance weighted	0.187912138	0.05	1
	IDP dMRI TBSS L3 Cingulum cingulate gyrus L	Wald ratio	0.0868087	0.05	1
	IDP dMRI TBSS L3 Cingulum hippocampus R	Wald ratio	0.0868087	0.05	1
	IDP dMRI TBSS L3 Cingulum hippocampus L	Wald ratio	0.0868087	0.05	1
	IDP dMRI TBSS L3 Fornix cres+Stria terminalis R	Wald ratio	0.637962629	0.05	1
	IDP dMRI TBSS L3 Superior longitudinal fasciculus R	Inverse variance weighted	0.256735818	0.05	1
	IDP dMRI TBSS L3 Superior longitudinal fasciculus L	Inverse variance weighted	0.253061197	0.05	1
	IDP dMRI TBSS L3 Uncinate fasciculus R	Wald ratio	0.0868087	0.05	1
	IDP dMRI TBSS L3 Uncinate fasciculus L	Inverse variance weighted	0.068814523	0.05	1
	IDP dMRI TBSS ICVF Middle cerebellar peduncle	Inverse variance weighted	0.140555159	0.05	1
	IDP dMRI TBSS ICVF Genu of corpus callosum	MR Egger	0.276003774	0.05	1
		Weighted median	0.918982757	0.05	1
		Inverse variance weighted	0.402281919	0.05	1
		Simple mode	0.957647486	0.05	1
		Weighted mode	0.950564521	0.05	1
	IDP dMRI TBSS ICVF Body of corpus callosum	MR Egger	0.163806403	0.05	1
		Weighted median	0.435955471	0.05	1
		Inverse variance weighted	0.161926868	0.05	1
		Simple mode	0.906247226	0.05	1
		Weighted mode	0.909490796	0.05	1

	IDP dMRI TBSS ICVF Splenium of corpus callosum	MR Egger	0.081179198	0.05	1
		Weighted median	0.489795097	0.05	1
		Inverse variance weighted	0.482049641	0.05	1
		Simple mode	0.669834328	0.05	1
		Weighted mode	0.405521398	0.05	1
	IDP dMRI TBSS ICVF Fornix	Wald ratio	0.800966226	0.05	1
	IDP dMRI TBSS ICVF Medial lemniscus R	Inverse variance weighted	0.558488387	0.05	1
	IDP dMRI TBSS ICVF Medial lemniscus L	Wald ratio	0.126157058	0.05	1
	IDP dMRI TBSS ICVF Inferior cerebellar peduncle R	MR Egger	0.493710941	0.05	1
		Weighted median	0.241840179	0.05	1
		Inverse variance weighted	0.120886212	0.05	1
		Simple mode	0.877539958	0.05	1
		Weighted mode	0.212977392	0.05	1
	IDP dMRI TBSS ICVF Inferior cerebellar peduncle L	MR Egger	0.526577865	0.05	1
		Weighted median	0.266280357	0.05	1
		Inverse variance weighted	0.131589271	0.05	1
		Simple mode	0.885592471	0.05	1
		Weighted mode	0.195018084	0.05	1
	IDP dMRI TBSS ICVF Superior cerebellar peduncle R	MR Egger	0.906313482	0.05	1
		Weighted median	0.935234095	0.05	1
		Inverse variance weighted	0.352904077	0.05	1
		Simple mode	0.996755562	0.05	1

		Weighted mode	0.967044008	0.05	1
	IDP dMRI TBSS ICVF Superior cerebellar peduncle L	Inverse variance weighted	0.56640869	0.05	1
	IDP dMRI TBSS ICVF Cerebral peduncle R	MR Egger	0.869807158	0.05	1
		Weighted median	0.71780545	0.05	1
		Inverse variance weighted	0.958382396	0.05	1
		Simple mode	0.474700985	0.05	1
		Weighted mode	0.642452727	0.05	1
	IDP dMRI TBSS ICVF Cerebral peduncle L	Inverse variance weighted	0.367460104	0.05	1
	IDP dMRI TBSS ICVF Anterior limb of internal capsule R	Wald ratio	0.0868087	0.05	1
	IDP dMRI TBSS ICVF Anterior limb of internal capsule L	MR Egger	0.666922688	0.05	1
		Weighted median	0.101631084	0.05	1
		Inverse variance weighted	0.049468363	0.05	1
		Simple mode	0.467723525	0.05	1
		Weighted mode	0.151803302	0.05	1
	IDP dMRI TBSS ICVF Posterior limb of internal capsule R	MR Egger	0.230685756	0.05	1
		Weighted median	0.593110455	0.05	1
		Inverse variance weighted	0.217401564	0.05	1
		Simple mode	0.988364187	0.05	1
		Weighted mode	0.152551834	0.05	1
	IDP dMRI TBSS ICVF Posterior limb of internal capsule L	MR Egger	0.364064432	0.05	1
		Weighted median	0.417538505	0.05	1
		Inverse variance weighted	0.248185372	0.05	1

		Simple mode	0.899628313	0.05	1
		Weighted mode	0.211704963	0.05	1
	IDP dMRI TBSS ICVF Retrolenticular part of internal capsule R	MR Egger	0.29859326	0.05	1
		Weighted median	0.664498293	0.05	1
		Inverse variance weighted	0.573056104	0.05	1
		Simple mode	0.95737213	0.05	1
		Weighted mode	0.373130996	0.05	1
	IDP dMRI TBSS ICVF Retrolenticular part of internal capsule L	MR Egger	0.365773186	0.05	1
		Weighted median	0.278126584	0.05	1
		Inverse variance weighted	0.209990165	0.05	1
		Simple mode	0.876249625	0.05	1
		Weighted mode	0.212993282	0.05	1
	IDP dMRI TBSS ICVF Anterior corona radiata R	MR Egger	0.366759878	0.05	1
		Weighted median	0.372118087	0.05	1
		Inverse variance weighted	0.23736857	0.05	1
		Simple mode	0.889688244	0.05	1
		Weighted mode	0.214689438	0.05	1
	IDP dMRI TBSS ICVF Anterior corona radiata L	MR Egger	0.364559891	0.05	1
		Weighted median	0.319826665	0.05	1
		Inverse variance weighted	0.222666371	0.05	1
		Simple mode	0.896291408	0.05	1
		Weighted mode	0.210040187	0.05	1

	IDP dMRI TBSS ICVF Superior corona radiata R	MR Egger	0.371473147	0.05	1
		Weighted median	0.379244005	0.05	1
		Inverse variance weighted	0.247604813	0.05	1
		Simple mode	0.885172374	0.05	1
		Weighted mode	0.201066201	0.05	1
	IDP dMRI TBSS ICVF Superior corona radiata L	MR Egger	0.368205811	0.05	1
		Weighted median	0.358967008	0.05	1
		Inverse variance weighted	0.240192169	0.05	1
		Simple mode	0.885150033	0.05	1
		Weighted mode	0.212993485	0.05	1
	IDP dMRI TBSS ICVF Posterior corona radiata R	MR Egger	0.42402522	0.05	1
		Weighted median	0.218108044	0.05	1
		Inverse variance weighted	0.153379998	0.05	1
		Simple mode	0.739961975	0.05	1
		Weighted mode	0.241062598	0.05	1
	IDP dMRI TBSS ICVF Posterior corona radiata L	MR Egger	0.434025255	0.05	1
		Weighted median	0.211407514	0.05	1
		Inverse variance weighted	0.14817193	0.05	1
		Simple mode	0.71717626	0.05	1
		Weighted mode	0.248606203	0.05	1
	IDP dMRI TBSS ICVF Posterior thalamic radiation R	Inverse variance weighted	0.139691583	0.05	1
		MR Egger	0.247305145	0.05	1

	IDP dMRI TBSS ICVF Posterior thalamic radiation L	Weighted median	0.346725204	0.05	1
		Inverse variance weighted	0.673546796	0.05	1
		Simple mode	0.704536836	0.05	1
		Weighted mode	0.277328397	0.05	1
	IDP dMRI TBSS ICVF Sagittal stratum R	MR Egger	0.149450964	0.05	1
		Weighted median	0.081446979	0.05	1
		Inverse variance weighted	0.681898591	0.05	1
		Simple mode	0.327590715	0.05	1
		Weighted mode	0.188958287	0.05	1
	IDP dMRI TBSS ICVF Sagittal stratum L	MR Egger	0.236521342	0.05	1
		Weighted median	0.268187472	0.05	1
		Inverse variance weighted	0.759094878	0.05	1
		Simple mode	0.685989771	0.05	1
		Weighted mode	0.285568689	0.05	1
	IDP dMRI TBSS ICVF External capsule R	MR Egger	0.357190587	0.05	1
		Weighted median	0.076980267	0.05	1
		Inverse variance weighted	0.134506705	0.05	1
		Simple mode	0.390665543	0.05	1
		Weighted mode	0.234244608	0.05	1
	IDP dMRI TBSS ICVF External capsule L	Inverse variance weighted	0.075096177	0.05	1
	IDP dMRI TBSS ICVF Cingulum cingulate gyrus R	Inverse variance weighted	0.136865962	0.05	1
	IDP dMRI TBSS ICVF Cingulum cingulate gyrus L	Inverse variance weighted	0.299470236	0.05	1

	IDP dMRI TBSS ICVF Cingulum hippocampus R	MR Egger	0.66663526	0.05	1
		Weighted median	0.093649516	0.05	1
		Inverse variance weighted	0.043289427	0.05	1
		Simple mode	0.311064227	0.05	1
		Weighted mode	0.253284899	0.05	1
	IDP dMRI TBSS ICVF Cingulum hippocampus L	MR Egger	0.364509856	0.05	1
		Weighted median	0.242295251	0.05	1
		Inverse variance weighted	0.193123009	0.05	1
		Simple mode	0.875045255	0.05	1
		Weighted mode	0.215245804	0.05	1
	IDP dMRI TBSS ICVF Fornix cres+Stria terminalis R	Inverse variance weighted	0.053549798	0.05	1
	IDP dMRI TBSS ICVF Fornix cres+Stria terminalis L	Wald ratio	0.079481048	0.05	1
	IDP dMRI TBSS ICVF Superior longitudinal fasciculus R	MR Egger	0.37513751	0.05	1
		Weighted median	0.413640462	0.05	1
		Inverse variance weighted	0.251679306	0.05	1
		Simple mode	0.892758056	0.05	1
		Weighted mode	0.175150239	0.05	1
	IDP dMRI TBSS ICVF Superior longitudinal fasciculus L	MR Egger	0.379652589	0.05	1
		Weighted median	0.341270341	0.05	1
		Inverse variance weighted	0.243361763	0.05	1
		Simple mode	0.882795819	0.05	1
		Weighted mode	0.215540286	0.05	1

	IDP dMRI TBSS ICVF Superior fronto-occipital fasciculus R	Inverse variance weighted	0.688129779	0.05	1
	IDP dMRI TBSS ICVF Superior fronto-occipital fasciculus L	Wald ratio	0.079481048	0.05	1
	IDP dMRI TBSS ICVF Uncinate fasciculus R	Wald ratio	0.0868087	0.05	1
	IDP dMRI TBSS ICVF Uncinate fasciculus L	Wald ratio	0.0868087	0.05	1
	IDP dMRI TBSS ICVF Tapetum R	Wald ratio	0.060854008	0.05	1
	IDP dMRI TBSS ICVF Tapetum L	Wald ratio	0.060854008	0.05	1
	IDP dMRI TBSS OD Pontine crossing tract	MR Egger	0.90788313	0.05	1
		Weighted median	0.899301934	0.05	1
		Inverse variance weighted	0.710578956	0.05	1
		Simple mode	0.902660601	0.05	1
		Weighted mode	0.898427316	0.05	1
	IDP dMRI TBSS OD Genu of corpus callosum	Wald ratio	0.686877836	0.05	1
	IDP dMRI TBSS OD Cerebral peduncle R	Wald ratio	0.780247235	0.05	1
	IDP dMRI TBSS OD Cerebral peduncle L	Wald ratio	0.780247235	0.05	1
	IDP dMRI TBSS OD Posterior limb of internal capsule L	Wald ratio	0.69316456	0.05	1
	IDP dMRI TBSS OD Posterior corona radiata R	Wald ratio	0.052942497	0.05	1
	IDP dMRI TBSS OD External capsule R	Inverse variance weighted	0.534821042	0.05	1
	IDP dMRI TBSS OD External capsule L	Wald ratio	0.459299674	0.05	1
	IDP dMRI TBSS OD Superior longitudinal fasciculus R	Wald ratio	0.166470831	0.05	1
	IDP dMRI TBSS ISOVF Fornix	Wald ratio	0.192917564	0.05	1
	IDP dMRI TBSS ISOVF External capsule R	Wald ratio	0.35794106	0.05	1

	IDP dMRI TBSS ISOVF Cingulum cingulate gyrus R	Inverse variance weighted	0.037314469	0.05	1
	IDP dMRI ProbtrackX FA ar l	Wald ratio	0.0868087	0.05	1
	IDP dMRI ProbtrackX FA atr l	Wald ratio	0.0868087	0.05	1
	IDP dMRI ProbtrackX FA atr r	Wald ratio	0.060854008	0.05	1
	IDP dMRI ProbtrackX FA fma	Wald ratio	0.060854008	0.05	1
	IDP dMRI ProbtrackX FA fmi	MR Egger	0.261061065	0.05	1
		Weighted median	0.839459522	0.05	1
		Inverse variance weighted	0.304302878	0.05	1
		Simple mode	0.986467349	0.05	1
		Weighted mode	0.982308155	0.05	1
	IDP dMRI ProbtrackX FA ifo l	Inverse variance weighted	0.13341587	0.05	1
	IDP dMRI ProbtrackX FA ifo r	Inverse variance weighted	0.146898626	0.05	1
	IDP dMRI ProbtrackX FA ilf l	Inverse variance weighted	0.106270837	0.05	1
	IDP dMRI ProbtrackX FA ilf r	Inverse variance weighted	0.107820495	0.05	1
	IDP dMRI ProbtrackX FA ml l	Wald ratio	0.208397443	0.05	1
	IDP dMRI ProbtrackX FA ptr l	Wald ratio	0.079481048	0.05	1
	IDP dMRI ProbtrackX FA ptr r	Wald ratio	0.042007364	0.05	1
	IDP dMRI ProbtrackX FA slf l	Inverse variance weighted	0.16002779	0.05	1
	IDP dMRI ProbtrackX FA slf r	Wald ratio	0.060854008	0.05	1
	IDP dMRI ProbtrackX FA str l	Wald ratio	0.187126199	0.05	1
	IDP dMRI ProbtrackX FA unc l	Wald ratio	0.0868087	0.05	1
	IDP dMRI ProbtrackX FA unc r	Wald ratio	0.0868087	0.05	1

	IDP dMRI ProbtrackX MD ar l	Wald ratio	0.0868087	0.05	1
	IDP dMRI ProbtrackX MD ar r	Wald ratio	0.0868087	0.05	1
	IDP dMRI ProbtrackX MD atr l	Wald ratio	0.0868087	0.05	1
	IDP dMRI ProbtrackX MD atr r	Wald ratio	0.0868087	0.05	1
	IDP dMRI ProbtrackX MD cgc l	MR Egger	0.424082481	0.05	1
		Weighted median	0.141004098	0.05	1
		Inverse variance weighted	0.125488691	0.05	1
		Simple mode	0.53953361	0.05	1
		Weighted mode	0.264383947	0.05	1
	IDP dMRI ProbtrackX MD cgc r	MR Egger	0.415817386	0.05	1
		Weighted median	0.124450111	0.05	1
		Inverse variance weighted	0.117974511	0.05	1
		Simple mode	0.415960515	0.05	1
		Weighted mode	0.245854414	0.05	1
	IDP dMRI ProbtrackX MD fmi	Inverse variance weighted	0.186137341	0.05	1
	IDP dMRI ProbtrackX MD ifo l	Inverse variance weighted	0.189874725	0.05	1
	IDP dMRI ProbtrackX MD ifo r	Inverse variance weighted	0.208568065	0.05	1
	IDP dMRI ProbtrackX MD ilf l	Inverse variance weighted	0.194191755	0.05	1
	IDP dMRI ProbtrackX MD ilf r	Inverse variance weighted	0.151380944	0.05	1
	IDP dMRI ProbtrackX MD ptr l	Wald ratio	0.0868087	0.05	1
	IDP dMRI ProbtrackX MD ptr r	Wald ratio	0.0868087	0.05	1

	IDP dMRI ProbtrackX MD slf l	Inverse variance weighted	0.212459419	0.05	1
	IDP dMRI ProbtrackX MD slf r	Inverse variance weighted	0.236532095	0.05	1
	IDP dMRI ProbtrackX MD str l	Wald ratio	0.917733206	0.05	1
	IDP dMRI ProbtrackX MD str r	Wald ratio	0.0868087	0.05	1
	IDP dMRI ProbtrackX MD unc l	Wald ratio	0.0868087	0.05	1
	IDP dMRI ProbtrackX MD unc r	Inverse variance weighted	0.879333435	0.05	1
	IDP dMRI ProbtrackX MO atr l	Wald ratio	0.151284429	0.05	1
	IDP dMRI ProbtrackX MO ml r	Wald ratio	0.824237276	0.05	1
	IDP T1 FAST ROIs L intracalc cortex	Wald ratio	0.930138155	0.05	1
	IDP dMRI ProbtrackX L1 fmi	Wald ratio	0.079481048	0.05	1
	IDP dMRI ProbtrackX L1 ifo l	Wald ratio	0.079481048	0.05	1
	IDP dMRI ProbtrackX L1 ifo r	Wald ratio	0.079481048	0.05	1
	IDP dMRI ProbtrackX L1 ilf l	Wald ratio	0.0868087	0.05	1
	IDP dMRI ProbtrackX L1 ilf r	Wald ratio	0.079481048	0.05	1
	IDP T1 FAST ROIs R intracalc cortex	Wald ratio	0.930138155	0.05	1
	IDP dMRI ProbtrackX L1 slf l	Wald ratio	0.0868087	0.05	1
	IDP dMRI ProbtrackX L1 slf r	Inverse variance weighted	0.947479793	0.05	1
	IDP dMRI ProbtrackX L1 str r	Wald ratio	0.675269776	0.05	1
	IDP dMRI ProbtrackX L1 unc l	Inverse variance weighted	0.081846887	0.05	1
	IDP dMRI ProbtrackX L1 unc r	Inverse variance weighted	0.857608785	0.05	1
	IDP dMRI ProbtrackX L2 ar l	Wald ratio	0.0868087	0.05	1
	IDP dMRI ProbtrackX L2 ar r	Wald ratio	0.0868087	0.05	1

	IDP dMRI ProbtrackX L2 atr l	Wald ratio	0.0868087	0.05	1
	IDP dMRI ProbtrackX L2 atr r	Wald ratio	0.0868087	0.05	1
	IDP dMRI ProbtrackX L2 cgc l	Wald ratio	0.079481048	0.05	1
	IDP dMRI ProbtrackX L2 cgc r	Wald ratio	0.079481048	0.05	1
	IDP dMRI ProbtrackX L2 cgh l	Wald ratio	0.443036137	0.05	1
	IDP dMRI ProbtrackX L2 fma	Wald ratio	0.962152328	0.05	1
	IDP dMRI ProbtrackX L2 fmi	Inverse variance weighted	0.188456552	0.05	1
	IDP dMRI ProbtrackX L2 ifo l	Inverse variance weighted	0.205854687	0.05	1
	IDP dMRI ProbtrackX L2 ifo r	Inverse variance weighted	0.235597729	0.05	1
	IDP dMRI ProbtrackX L2 ilf l	Inverse variance weighted	0.189988032	0.05	1
	IDP dMRI ProbtrackX L2 ilf r	Inverse variance weighted	0.163198923	0.05	1
	IDP dMRI ProbtrackX L2 ptr l	Wald ratio	0.0868087	0.05	1
	IDP dMRI ProbtrackX L2 ptr r	Wald ratio	0.067101748	0.05	1
	IDP dMRI ProbtrackX L2 slf l	Inverse variance weighted	0.219278254	0.05	1
	IDP dMRI ProbtrackX L2 slf r	Inverse variance weighted	0.267495808	0.05	1
	IDP dMRI ProbtrackX L2 str l	Wald ratio	0.917733206	0.05	1
	IDP dMRI ProbtrackX L2 str r	Wald ratio	0.917733206	0.05	1
	IDP dMRI ProbtrackX L2 unc l	Wald ratio	0.0868087	0.05	1
	IDP dMRI ProbtrackX L2 unc r	Inverse variance weighted	0.861937809	0.05	1
	IDP dMRI ProbtrackX L3 ar l	Wald ratio	0.0868087	0.05	1
	IDP dMRI ProbtrackX L3 ar r	Wald ratio	0.0868087	0.05	1

	IDP dMRI ProbtrackX L3 atr l	Wald ratio	0.0868087	0.05	1
	IDP dMRI ProbtrackX L3 atr r	Wald ratio	0.0868087	0.05	1
	IDP dMRI ProbtrackX L3 cgc l	Wald ratio	0.060854008	0.05	1
	IDP dMRI ProbtrackX L3 cgc r	Wald ratio	0.060854008	0.05	1
	IDP dMRI ProbtrackX L3 fmi	MR Egger	0.368350988	0.05	1
		Weighted median	0.468916513	0.05	1
		Inverse variance weighted	0.268932289	0.05	1
		Simple mode	0.894635298	0.05	1
		Weighted mode	0.207162514	0.05	1
	IDP dMRI ProbtrackX L3 ifo l	Inverse variance weighted	0.158384065	0.05	1
	IDP dMRI ProbtrackX L3 ifo r	Inverse variance weighted	0.176685903	0.05	1
	IDP dMRI ProbtrackX L3 ilf l	Inverse variance weighted	0.158722538	0.05	1
	IDP dMRI ProbtrackX L3 ilf r	Inverse variance weighted	0.142419109	0.05	1
	IDP dMRI ProbtrackX L3 ptr l	Wald ratio	0.0868087	0.05	1
	IDP dMRI ProbtrackX L3 ptr r	Wald ratio	0.067101748	0.05	1
	IDP dMRI ProbtrackX L3 slf l	Inverse variance weighted	0.199631162	0.05	1
	IDP dMRI ProbtrackX L3 slf r	Inverse variance weighted	0.215394434	0.05	1
	IDP dMRI ProbtrackX L3 str l	Wald ratio	0.067101748	0.05	1
	IDP dMRI ProbtrackX L3 str r	Wald ratio	0.079481048	0.05	1
	IDP dMRI ProbtrackX L3 unc l	Wald ratio	0.0868087	0.05	1
	IDP dMRI ProbtrackX L3 unc r	Inverse variance weighted	0.908356184	0.05	1

	IDP dMRI ProbtrackX ICVF ar l	MR Egger	0.334602556	0.05	1
		Weighted median	0.333229649	0.05	1
		Inverse variance weighted	0.265068529	0.05	1
		Simple mode	0.707619487	0.05	1
		Weighted mode	0.210981336	0.05	1
	IDP dMRI ProbtrackX ICVF ar r	MR Egger	0.336044945	0.05	1
		Weighted median	0.390102764	0.05	1
		Inverse variance weighted	0.278534453	0.05	1
		Simple mode	0.709473064	0.05	1
		Weighted mode	0.202758959	0.05	1
	IDP dMRI ProbtrackX ICVF atr l	MR Egger	0.364159912	0.05	1
		Weighted median	0.254638429	0.05	1
		Inverse variance weighted	0.201405636	0.05	1
		Simple mode	0.87854754	0.05	1
		Weighted mode	0.216399562	0.05	1
	IDP dMRI ProbtrackX ICVF atr r	MR Egger	0.364363849	0.05	1
		Weighted median	0.293018134	0.05	1
		Inverse variance weighted	0.211328464	0.05	1
		Simple mode	0.887695704	0.05	1
		Weighted mode	0.202321507	0.05	1
	IDP dMRI ProbtrackX ICVF cgc l	Inverse variance weighted	0.324681227	0.05	1
	IDP dMRI ProbtrackX ICVF cgc r	Wald ratio	0.079481048	0.05	1

	IDP dMRI ProbtrackX ICVF cgh l	MR Egger	0.364418887	0.05	1
		Weighted median	0.276428406	0.05	1
		Inverse variance weighted	0.200901496	0.05	1
		Simple mode	0.87949744	0.05	1
		Weighted mode	0.22001337	0.05	1
	IDP dMRI ProbtrackX ICVF cgh r	Wald ratio	0.0868087	0.05	1
	IDP dMRI ProbtrackX ICVF cst l	MR Egger	0.335740614	0.05	1
		Weighted median	0.536572251	0.05	1
		Inverse variance weighted	0.360517045	0.05	1
		Simple mode	0.726129979	0.05	1
		Weighted mode	0.212828016	0.05	1
	IDP dMRI ProbtrackX ICVF cst r	MR Egger	0.159571584	0.05	1
		Weighted median	0.700029289	0.05	1
		Inverse variance weighted	0.756150641	0.05	1
		Simple mode	0.994859165	0.05	1
		Weighted mode	0.290074257	0.05	1
	IDP dMRI ProbtrackX ICVF fma	MR Egger	0.35422821	0.05	1
		Weighted median	0.295438712	0.05	1
		Inverse variance weighted	0.158900386	0.05	1
		Simple mode	0.964062279	0.05	1
		Weighted mode	0.190570425	0.05	1
	IDP dMRI ProbtrackX ICVF fmi	MR Egger	0.248255395	0.05	1

		Weighted median	0.658492048	0.05	1
		Inverse variance weighted	0.285143901	0.05	1
		Simple mode	0.925176746	0.05	1
		Weighted mode	0.171643711	0.05	1
	IDP dMRI ProbtrackX ICVF ifo l	Inverse variance weighted	0.142776535	0.05	1
	IDP dMRI ProbtrackX ICVF ifo r	Inverse variance weighted	0.14410073	0.05	1
	IDP dMRI ProbtrackX ICVF ilf l	Inverse variance weighted	0.14833927	0.05	1
	IDP dMRI ProbtrackX ICVF ilf r	Inverse variance weighted	0.136394235	0.05	1
	IDP dMRI ProbtrackX ICVF mcp	Inverse variance weighted	0.810891814	0.05	1
	IDP dMRI ProbtrackX ICVF ml l	Wald ratio	0.050011707	0.05	1
	IDP dMRI ProbtrackX ICVF ml r	Wald ratio	0.0868087	0.05	1
	IDP dMRI ProbtrackX ICVF ptr l	Inverse variance weighted	0.132555032	0.05	1
	IDP dMRI ProbtrackX ICVF ptr r	MR Egger	0.626409984	0.05	1
		Weighted median	0.162433827	0.05	1
		Inverse variance weighted	0.866285592	0.05	1
		Simple mode	0.47947526	0.05	1
		Weighted mode	0.228965371	0.05	1
	IDP dMRI ProbtrackX ICVF slf l	MR Egger	0.368520734	0.05	1
		Weighted median	0.341848367	0.05	1
		Inverse variance weighted	0.229981415	0.05	1
		Simple mode	0.880697838	0.05	1

		Weighted mode	0.224300203	0.05	1
	IDP dMRI ProbtrackX ICVF slf r	MR Egger	0.364026463	0.05	1
		Weighted median	0.321062966	0.05	1
		Inverse variance weighted	0.218993064	0.05	1
		Simple mode	0.873899	0.05	1
		Weighted mode	0.198004469	0.05	1
	IDP dMRI ProbtrackX ICVF str l	MR Egger	0.364052476	0.05	1
		Weighted median	0.363997861	0.05	1
		Inverse variance weighted	0.240055164	0.05	1
		Simple mode	0.888991066	0.05	1
		Weighted mode	0.202836372	0.05	1
	IDP dMRI ProbtrackX ICVF str r	MR Egger	0.365955855	0.05	1
		Weighted median	0.364010844	0.05	1
		Inverse variance weighted	0.238268469	0.05	1
		Simple mode	0.884883144	0.05	1
		Weighted mode	0.216221317	0.05	1
	IDP dMRI ProbtrackX ICVF unc l	Inverse variance weighted	0.152789559	0.05	1
	IDP dMRI ProbtrackX ICVF unc r	Inverse variance weighted	0.124270617	0.05	1
	IDP dMRI ProbtrackX OD atr r	Wald ratio	0.661547112	0.05	1
	IDP dMRI ProbtrackX OD ilf l	Wald ratio	0.765566362	0.05	1
	IDP dMRI ProbtrackX OD slf l	Wald ratio	0.384364598	0.05	1
	IDP dMRI ProbtrackX OD str l	Inverse variance weighted	0.680621845	0.05	1

	IDP dMRI ProbtrackX ISOVF atr r	Wald ratio	0.0868087	0.05	1
	IDP dMRI ProbtrackX ISOVF cgc l	Wald ratio	0.904501476	0.05	1
	IDP dMRI ProbtrackX ISOVF ifo r	Inverse variance weighted	0.042823249	0.05	1
	IDP dMRI ProbtrackX ISOVF ilf l	Wald ratio	0.785663937	0.05	1
	IDP dMRI ProbtrackX ISOVF ilf r	MR Egger	0.559928924	0.05	1
		Weighted median	0.219861896	0.05	1
		Inverse variance weighted	0.693249374	0.05	1
		Simple mode	0.400144818	0.05	1
		Weighted mode	0.375243372	0.05	1
	IDP dMRI ProbtrackX ISOVF slf l	Inverse variance weighted	0.064656552	0.05	1
	IDP dMRI ProbtrackX ISOVF slf r	MR Egger	0.63254765	0.05	1
		Weighted median	0.084470294	0.05	1
		Inverse variance weighted	0.309684014	0.05	1
		Simple mode	0.26562965	0.05	1
		Weighted mode	0.264324064	0.05	1
	IDP dMRI ProbtrackX ISOVF unc l	Wald ratio	0.165497468	0.05	1
	IDP dMRI ProbtrackX ISOVF unc r	Wald ratio	0.083999655	0.05	1
	IDP T1 SIENAX brain-normalised volume	Wald ratio	0.139762546	0.05	1
Dermatopolymyositis	IDP T1 SIENAX peripheral grey normalised volume	Wald ratio	0.387942842	0.05	1
	IDP T1 FAST ROIs R temp occ fusif cortex	Wald ratio	0.802805197	0.05	1
	IDP T1 FAST ROIs R occ pole	Inverse variance weighted	0.430519642	0.05	1
	IDP T1 FAST ROIs L thalamus	Inverse variance weighted	0.811313771	0.05	1

	IDP T1 FAST ROIs R thalamus	Wald ratio	0.794396315	0.05	1
	IDP T1 FAST ROIs L putamen	Wald ratio	0.719300517	0.05	1
	IDP T1 FAST ROIs R putamen	Wald ratio	0.719300517	0.05	1
	IDP T1 FAST ROIs L pallidum	Wald ratio	0.434915381	0.05	0.999999944
	IDP T1 FIRST left caudate volume	Wald ratio	0.579438789	0.05	1
	IDP T1 FAST ROIs L hippocampus	Inverse variance weighted	0.031729088	0.05	0.483996825
	IDP T1 FAST ROIs R hippocampus	Wald ratio	0.080739476	0.05	0.398735581
	IDP T1 FAST ROIs L ventral striatum	Wald ratio	0.719300517	0.05	1
	IDP T1 FAST ROIs R ventral striatum	Inverse variance weighted	0.827035747	0.05	1
	IDP T1 FAST ROIs L cerebellum VI	Wald ratio	0.250006737	0.05	0.869593624
	IDP T1 FAST ROIs L cerebellum crus I	MR Egger	0.975176401	0.05	1
		Weighted median	0.933566875	0.05	1
		Inverse variance weighted	0.882324315	0.05	1
		Simple mode	0.988375154	0.05	1
		Weighted mode	0.920643158	0.05	1
	IDP T1 FAST ROIs R cerebellum crus I	Inverse variance weighted	0.838865324	0.05	1
	IDP T1 FAST ROIs L cerebellum crus II	MR Egger	0.512753761	0.05	0.794403264
		Weighted median	0.760606107	0.05	1
		Inverse variance weighted	0.99046792	0.05	1
		Simple mode	0.822864017	0.05	1
		Weighted mode	0.67965894	0.05	1
	IDP T1 FAST ROIs V cerebellum crus II	Inverse variance weighted	0.953943268	0.05	1

	IDP T1 FAST ROIs R cerebellum crus II	Inverse variance weighted	0.960251599	0.05	1
	IDP T1 FIRST left putamen volume	Inverse variance weighted	0.310382273	0.05	1
	IDP T1 FAST ROIs R cerebellum VIIb	Wald ratio	0.810723327	0.05	1
	IDP T1 FAST ROIs V cerebellum VIIIa	MR Egger	0.41330706	0.05	1
		Weighted median	0.271674406	0.05	0.999993028
		Inverse variance weighted	0.442592501	0.05	1
		Simple mode	0.599215332	0.05	0.999999945
		Weighted mode	0.343606052	0.05	0.999886056
	IDP T1 FAST ROIs L cerebellum VIIIb	Wald ratio	0.392397715	0.05	0.729171519
	IDP T1 FAST ROIs V cerebellum VIIIb	Inverse variance weighted	0.135884001	0.05	0.954852324
	IDP T1 FAST ROIs R cerebellum VIIIb	Wald ratio	0.719300517	0.05	1
	IDP T1 FAST ROIs L cerebellum IX	Inverse variance weighted	0.522745008	0.05	1
	IDP T1 FIRST right putamen volume	Wald ratio	0.283821115	0.05	1
	IDP T1 FAST ROIs V cerebellum IX	Inverse variance weighted	0.384746987	0.05	0.999999999
	IDP T1 FAST ROIs R cerebellum IX	Inverse variance weighted	0.515690737	0.05	1
	IDP T1 FAST ROIs V cerebellum X	Wald ratio	0.719300517	0.05	1
	IDP T2 FLAIR BIANCA WMH volume	Inverse variance weighted	0.492186053	0.05	1
	IDP SWI T2star left caudate	MR Egger	0.581043161	0.05	0.862696542
		Weighted median	0.750530471	0.05	1
		Inverse variance weighted	0.865820343	0.05	1
		Simple mode	0.397778947	0.05	1

		Weighted mode	0.662106287	0.05	1
	IDP SWI T2star right caudate	MR Egger	0.710152795	0.05	1
		Weighted median	0.96344912	0.05	1
		Inverse variance weighted	0.97309345	0.05	1
		Simple mode	0.886569084	0.05	1
		Weighted mode	0.953372777	0.05	1
	IDP T1 FIRST left pallidum volume	Wald ratio	0.005091501	0.05	1
	IDP SWI T2star left putamen	MR Egger	0.207009568	0.05	0.511846229
		Weighted median	0.807904215	0.05	1
		Inverse variance weighted	0.448596891	0.05	1
		Simple mode	0.855370181	0.05	1
		Weighted mode	0.867122121	0.05	1
	IDP SWI T2star right putamen	MR Egger	0.475271615	0.05	0.963410624
		Weighted median	0.828474479	0.05	1
		Inverse variance weighted	0.798603034	0.05	1
		Simple mode	0.710060483	0.05	1
		Weighted mode	0.685646426	0.05	1
	IDP SWI T2star left pallidum	MR Egger	0.507290414	0.05	1
		Weighted median	0.645933878	0.05	1
		Inverse variance weighted	0.802188049	0.05	1
		Simple mode	0.823188939	0.05	1
		Weighted mode	0.783669198	0.05	1

	IDP SWI T2star right pallidum	MR Egger	0.965717105	0.05	1
		Weighted median	0.930862803	0.05	1
		Inverse variance weighted	0.798819748	0.05	1
		Simple mode	0.902463676	0.05	1
		Weighted mode	0.920031672	0.05	1
	IDP T1 FIRST right pallidum volume	Wald ratio	0.005091501	0.05	1
	IDP dMRI TBSS FA Genu of corpus callosum	Wald ratio	0.78336801	0.05	1
	IDP dMRI TBSS FA Splenium of corpus callosum	Inverse variance weighted	0.032007116	0.05	0.962876131
	IDP dMRI TBSS FA Corticospinal tract L	Wald ratio	0.027521165	0.05	0.643524763
	IDP dMRI TBSS FA Superior cerebellar peduncle R	Inverse variance weighted	0.165828093	0.05	0.999999999
	IDP dMRI TBSS FA Superior cerebellar peduncle L	Inverse variance weighted	0.144964971	0.05	0.999999916
	IDP dMRI TBSS FA Posterior limb of internal capsule R	Wald ratio	0.95408656	0.05	1
	IDP dMRI TBSS FA Retrolenticular part of internal capsule R	Inverse variance weighted	0.990150324	0.05	1
	IDP dMRI TBSS FA Anterior corona radiata R	Wald ratio	0.78336801	0.05	1
	IDP dMRI TBSS FA Anterior corona radiata L	Wald ratio	0.78336801	0.05	1
	IDP dMRI TBSS FA Posterior thalamic radiation R	Wald ratio	0.569795936	0.05	1
	IDP dMRI TBSS FA Posterior thalamic radiation L	Wald ratio	0.569160445	0.05	1
	IDP dMRI TBSS FA Sagittal stratum R	Wald ratio	0.856906388	0.05	1
	IDP dMRI TBSS FA Cingulum cingulate gyrus R	Inverse variance weighted	0.739941569	0.05	1
	IDP dMRI TBSS FA Superior longitudinal fasciculus L	Inverse variance weighted	0.717969313	0.05	1

	IDP dMRI TBSS FA Uncinate fasciculus L	Wald ratio	0.800938494	0.05	1
	IDP dMRI TBSS MD Genu of corpus callosum	Wald ratio	0.78336801	0.05	1
	IDP dMRI TBSS MD Body of corpus callosum	Wald ratio	0.202315022	0.05	1
	IDP dMRI TBSS MD Splenium of corpus callosum	Inverse variance weighted	0.276906965	0.05	1
	IDP dMRI TBSS MD Corticospinal tract R	Wald ratio	0.709735791	0.05	1
	IDP dMRI TBSS MD Superior cerebellar peduncle R	Wald ratio	0.069684887	0.05	1
	IDP dMRI TBSS MD Superior cerebellar peduncle L	Wald ratio	0.0866451	0.05	1
	IDP dMRI TBSS MD Anterior limb of internal capsule L	Wald ratio	0.664991075	0.05	1
	IDP dMRI TBSS MD Retrolenticular part of internal capsule R	Wald ratio	0.569160445	0.05	1
	IDP T1 FIRST left caudate volume plus IDP T1 FIRST right caudate volume	Wald ratio	0.55936579	0.05	1
	IDP T1 FIRST left putamen volume plus IDP T1 FIRST right putamen volume	Inverse variance weighted	0.323682299	0.05	1
	IDP T1 FIRST left pallidum volume plus IDP T1 FIRST right pallidum volume	Wald ratio	0.005091501	0.05	1
	IDP SWI T2star left thalamus plus IDP SWI T2star right thalamus	MR Egger	0.85018605	0.05	1
		Weighted median	0.665402386	0.05	1
		Inverse variance weighted	0.399886632	0.05	1
		Simple mode	0.928514152	0.05	1
		Weighted mode	0.941423213	0.05	1
	IDP dMRI TBSS MD Retrolenticular part of internal capsule L	Wald ratio	0.569160445	0.05	1
	IDP SWI T2star left caudate plus IDP SWI T2star right caudate	MR Egger	0.633164669	0.05	1
		Weighted median	0.949544097	0.05	1

		Inverse variance weighted	0.842314787	0.05	1
		Simple mode	0.783636499	0.05	1
		Weighted mode	0.932674266	0.05	1
	IDP SWI T2star left putamen plus IDP SWI T2star right putamen	MR Egger	0.197285477	0.05	0.999999963
		Weighted median	0.385850472	0.05	1
		Inverse variance weighted	0.401851334	0.05	1
		Simple mode	0.633132122	0.05	1
		Weighted mode	0.632754578	0.05	1
	IDP SWI T2star left pallidum plus IDP SWI T2star right pallidum	MR Egger	0.502096828	0.05	1
		Weighted median	0.915303186	0.05	1
		Inverse variance weighted	0.591036742	0.05	1
		Simple mode	0.845406443	0.05	1
		Weighted mode	0.875054999	0.05	1
	volume Left-Lateral-Ventricle	MR Egger	0.852342712	0.05	1
		Weighted median	0.247528985	0.05	0.999992
		Inverse variance weighted	0.141031797	0.05	0.999643203
		Simple mode	0.637471015	0.05	1
		Weighted mode	0.583828577	0.05	1
	volume Left-Cerebellum-White-Matter	Inverse variance weighted	0.644833899	0.05	1
	volume Left-Cerebellum-Cortex	Inverse variance weighted	0.924040223	0.05	1
	IDP dMRI TBSS MD Anterior corona radiata R	Inverse variance weighted	0.900351981	0.05	1

	volume Left-Putamen	Inverse variance weighted	0.97937139	0.05	1
	volume 4th-Ventricle	Inverse variance weighted	0.958791928	0.05	1
	volume Brain-Stem	Wald ratio	0.118083559	0.05	1
	volume Left-Hippocampus	Inverse variance weighted	0.123120057	0.05	0.013990914
	volume CSF	MR Egger	0.861704312	0.05	0.002658206
		Weighted median	0.817576098	0.05	1
		Inverse variance weighted	0.362729896	0.05	1
		Simple mode	0.913240864	0.05	1
		Weighted mode	0.90773459	0.05	1
	IDP dMRI TBSS MD Anterior corona radiata L	Inverse variance weighted	0.922208209	0.05	1
	volume Left-Accumbens-area	Wald ratio	0.719300517	0.05	0.999999999
	volume Right-Lateral-Ventricle	Inverse variance weighted	0.148702187	0.05	0.974835212
	volume Right-Inf-Lat-Vent	Wald ratio	0.672099758	0.05	1
	volume Right-Cerebellum-White-Matter	Wald ratio	0.152965764	0.05	1
	volume Right-Cerebellum-Cortex	MR Egger	0.863645636	0.05	1
		Weighted median	0.962411339	0.05	1
		Inverse variance weighted	0.99988099	0.05	1
		Simple mode	0.996634385	0.05	1
		Weighted mode	0.929354549	0.05	1
	IDP dMRI TBSS MD Superior corona radiata R	Inverse variance weighted	0.900782856	0.05	1
	volume Right-Putamen	Wald ratio	0.283821115	0.05	1

	volume Right-Hippocampus	Wald ratio	0.080739476	0.05	0.21029672
	volume Right-Amygdala	Wald ratio	0.104732347	0.05	0.062710352
	volume Right-VentralDC	Wald ratio	0.751273841	0.05	1
	volume CC Posterior	Wald ratio	0.661386283	0.05	1
	IDP dMRI TBSS MD Superior corona radiata L	Inverse variance weighted	0.903098334	0.05	1
	IDP dMRI TBSS MD Posterior corona radiata R	Wald ratio	0.569160445	0.05	1
	volume BrainSegVol-to-eTIV	Wald ratio	0.738224167	0.05	1
	IDP dMRI TBSS MD Posterior corona radiata L	Wald ratio	0.569160445	0.05	1
	volume rhSurfaceHoles	Wald ratio	0.543522414	0.05	1
	DKTatlas lh cuneus area	Wald ratio	0.972732165	0.05	1
	IDP dMRI TBSS MD Posterior thalamic radiation R	Wald ratio	0.569160445	0.05	1
	DKTatlas lh lateraloccipital area	Wald ratio	0.972732165	0.05	1
	DKTatlas lh lingual area	Wald ratio	0.969722601	0.05	1
	IDP dMRI TBSS MD Posterior thalamic radiation L	Wald ratio	0.569160445	0.05	1
	DKTatlas lh parstriangularis area	Wald ratio	0.1384877	0.05	0.824997123
	DKTatlas lh pericalcarine area	Inverse variance weighted	0.813747328	0.05	1
	DKTatlas lh postcentral area	Inverse variance weighted	0.393011921	0.05	1
	DKTatlas lh posteriorcingulate area	Wald ratio	0.322595478	0.05	0.771565852
	DKTatlas lh precentral area	Wald ratio	0.572582414	0.05	1
	DKTatlas lh precuneus area	Wald ratio	0.160784187	0.05	0.308188659
	IDP dMRI TBSS MD Sagittal stratum R	Wald ratio	0.569160445	0.05	1
	DKTatlas lh superiorparietal area	Wald ratio	0.721760878	0.05	1

	DKTatlas lh superiortemporal area	Wald ratio	0.665139345	0.05	1
	DKTatlas lh supramarginal area	Wald ratio	0.386566742	0.05	0.999727602
	DKTatlas lh WhiteSurfArea area	Wald ratio	0.672267743	0.05	0.999999999
	IDP dMRI TBSS MD Sagittal stratum L	Wald ratio	0.569160445	0.05	1
	a2009s lh G&S subcentral area	Wald ratio	0.572582414	0.05	1
	a2009s lh G cuneus area	Wald ratio	0.958937792	0.05	1
	a2009s lh G front inf-Opercular area	Wald ratio	0.377566796	0.05	1
	IDP dMRI TBSS MD External capsule R	Wald ratio	0.78336801	0.05	1
	IDP dMRI TBSS MD External capsule L	Inverse variance weighted	0.624931713	0.05	1
	a2009s lh G pariet inf-Supramar area	Wald ratio	0.386566742	0.05	0.999981943
	a2009s lh G parietal sup area	Wald ratio	0.723119595	0.05	1
	a2009s lh G postcentral area	Wald ratio	0.724772932	0.05	1
	a2009s lh G precentral area	Wald ratio	0.572582414	0.05	1
	a2009s lh G precuneus area	Wald ratio	0.958937792	0.05	1
	IDP dMRI TBSS MD Cingulum cingulate gyrus R	MR Egger	0.695537013	0.05	0.953109095
		Weighted median	0.817154528	0.05	1
		Inverse variance weighted	0.909715252	0.05	1
		Simple mode	0.712279676	0.05	1
		Weighted mode	0.765131417	0.05	1
	IDP dMRI TBSS MD Cingulum cingulate gyrus L	MR Egger	0.686089595	0.05	0.964027457
		Weighted median	0.811019872	0.05	1
		Inverse variance weighted	0.906351015	0.05	1
		Simple mode	0.682524581	0.05	1

		Weighted mode	0.753199803	0.05	1
	a2009s lh S calcarine area	Wald ratio	0.969722601	0.05	1
	a2009s lh S central area	Wald ratio	0.574794751	0.05	1
	a2009s lh S collat transv ant area	Wald ratio	0.654525238	0.05	1
	a2009s lh S front middle area	Wald ratio	0.407061052	0.05	1
	IDP dMRI TBSS MD Cingulum hippocampus R	Wald ratio	0.569160445	0.05	1
	a2009s lh S intrapariet&P trans area	Wald ratio	0.723119595	0.05	1
	IDP dMRI TBSS MD Cingulum hippocampus L	Wald ratio	0.569160445	0.05	1
	a2009s lh S subparietal area	Wald ratio	0.969722601	0.05	1
	DKTatlas rh cuneus area	Wald ratio	0.969722601	0.05	1
	DKTatlas rh lingual area	Wald ratio	0.971468606	0.05	1
	DKTatlas rh parstriangularis area	Wald ratio	0.027767576	0.05	0.198287262
	DKTatlas rh pericalcarine area	Inverse variance weighted	0.39821427	0.05	1
	IDP dMRI TBSS MD Superior longitudinal fasciculus R	Wald ratio	0.569160445	0.05	1
	DKTatlas rh postcentral area	Wald ratio	0.574794751	0.05	1
	DKTatlas rh precentral area	Wald ratio	0.572582414	0.05	1
	IDP dMRI TBSS MD Superior longitudinal fasciculus L	Wald ratio	0.569160445	0.05	1
	a2009s rh G&S subcentral area	Wald ratio	0.574794751	0.05	1
	a2009s rh G&S cingul-Mid-Post area	Wald ratio	0.338587593	0.05	0.999369981
	a2009s rh G cuneus area	Wald ratio	0.969722601	0.05	1
	IDP dMRI TBSS MD Superior fronto-occipital fasciculus L	Wald ratio	0.662854426	0.05	1
	a2009s rh G oc-temp med-Lingual area	Wald ratio	0.971468606	0.05	1

	a2009s rh G parietal sup area	Wald ratio	0.296397561	0.05	0.988932768
	IDP dMRI TBSS MD Uncinate fasciculus R	Wald ratio	0.657446023	0.05	1
	a2009s rh G precentral area	Wald ratio	0.572582414	0.05	1
	a2009s rh G precuneus area	Wald ratio	0.288037317	0.05	0.986997254
	IDP dMRI TBSS MD Uncinate fasciculus L	Inverse variance weighted	0.640876812	0.05	1
	a2009s rh Pole occipital area	Wald ratio	0.969722601	0.05	1
	a2009s rh S calcarine area	Wald ratio	0.971468606	0.05	1
	a2009s rh S central area	Wald ratio	0.572582414	0.05	1
	a2009s rh S collat transv ant area	Wald ratio	0.654525238	0.05	1
	a2009s rh S orbital med-olfact area	Wald ratio	0.735825923	0.05	1
	IDP dMRI TBSS MO Pontine crossing tract	MR Egger	0.646581837	0.05	0.999999454
		Weighted median	0.828491558	0.05	1
		Inverse variance weighted	0.644522741	0.05	1
		Simple mode	0.5898313	0.05	1
		Weighted mode	0.656778873	0.05	1
	DKTatlas lh postcentral thickness	Wald ratio	0.574794751	0.05	1
	a2009s lh G insular short thickness	Wald ratio	0.160402997	0.05	1
	a2009s lh G postcentral thickness	Wald ratio	0.574794751	0.05	1
	IDP T1 SIENAX CSF normalised volume	Inverse variance weighted	0.502719597	0.05	1
	IDP dMRI TBSS MO Medial lemniscus R	Wald ratio	0.868266811	0.05	1
	a2009s lh S postcentral thickness	Wald ratio	0.450699545	0.05	1
	DKTatlas rh lateraloccipital thickness	Wald ratio	0.719300517	0.05	1
	DKTatlas rh parstriangularis thickness	Wald ratio	0.765414105	0.05	1

	DKTatlas rh postcentral thickness	Wald ratio	0.574794751	0.05	1
	DKTatlas rh posteriorcingulate thickness	Inverse variance weighted	0.215616755	0.05	1
	a2009s rh G&S cingul-Mid-Ant thickness	Wald ratio	0.708579899	0.05	1
	a2009s rh G cuneus thickness	Wald ratio	0.719300517	0.05	1
	a2009s rh G postcentral thickness	Wald ratio	0.574794751	0.05	1
	a2009s rh Pole occipital thickness	Wald ratio	0.719300517	0.05	1
	a2009s rh S oc sup&transversal thickness	Wald ratio	0.459896014	0.05	1
	a2009s rh S parieto occipital thickness	Wald ratio	0.355209665	0.05	0.137716987
	IDP dMRI TBSS MO Anterior corona radiata R	Wald ratio	0.211115854	0.05	1
	IDP dMRI TBSS MO Superior corona radiata R	Wald ratio	0.674069594	0.05	1
	IDP dMRI TBSS MO Cingulum cingulate gyrus L	Inverse variance weighted	0.396672649	0.05	1
	IDP dMRI TBSS L1 Anterior limb of internal capsule L	Wald ratio	0.664991075	0.05	1
	IDP dMRI TBSS L1 Posterior limb of internal capsule R	Wald ratio	0.332745286	0.05	1
	IDP dMRI TBSS L1 Posterior limb of internal capsule L	Wald ratio	0.186983151	0.05	1
	IDP dMRI TBSS L1 Anterior corona radiata R	Wald ratio	0.78336801	0.05	1
	IDP dMRI TBSS L1 Anterior corona radiata L	Wald ratio	0.78336801	0.05	1
	IDP dMRI TBSS L1 Posterior corona radiata R	Wald ratio	0.153729285	0.05	1
	IDP dMRI TBSS L1 Posterior corona radiata L	Wald ratio	0.78336801	0.05	1
	IDP dMRI TBSS L1 Sagittal stratum L	Wald ratio	0.78336801	0.05	1
	IDP dMRI TBSS L1 External capsule R	Wald ratio	0.78336801	0.05	1

	IDP dMRI TBSS L1 External capsule L	Inverse variance weighted	0.749566398	0.05	1
	IDP dMRI TBSS L1 Fornix cres+Stria terminalis L	Wald ratio	0.310690554	0.05	1
	IDP T1 FAST ROIs L precentral gyrus	Wald ratio	0.574794751	0.05	1
	IDP dMRI TBSS L1 Superior longitudinal fasciculus R	Inverse variance weighted	0.934570332	0.05	1
	IDP dMRI TBSS L1 Uncinate fasciculus L	Wald ratio	0.572555206	0.05	1
	IDP dMRI TBSS L2 Pontine crossing tract	Wald ratio	0.868266811	0.05	1
	IDP dMRI TBSS L2 Genu of corpus callosum	MR Egger	0.352033981	0.05	0.003337888
		Weighted median	0.298763135	0.05	1
		Inverse variance weighted	0.283394017	0.05	1
		Simple mode	0.437231927	0.05	1
		Weighted mode	0.799562004	0.05	1
	IDP dMRI TBSS L2 Body of corpus callosum	Wald ratio	0.78336801	0.05	1
	IDP dMRI TBSS L2 Splenium of corpus callosum	MR Egger	0.427906135	0.05	1
		Weighted median	0.350844649	0.05	1
		Inverse variance weighted	0.03158213	0.05	1
		Simple mode	0.590368859	0.05	1
		Weighted mode	0.591291784	0.05	1
	IDP dMRI TBSS L2 Corticospinal tract R	Wald ratio	0.709735791	0.05	1
	IDP T1 SIENAX CSF unnormalised volume	MR Egger	0.781093999	0.05	1
		Weighted median	0.287273389	0.05	0.999975613
		Inverse variance weighted	0.147647953	0.05	0.998187196
		Simple mode	0.653864287	0.05	1

		Weighted mode	0.623148478	0.05	1
	IDP T1 FAST ROIs L temporal pole	Wald ratio	0.40224303	0.05	1
	IDP dMRI TBSS L2 Superior cerebellar peduncle R	Inverse variance weighted	0.15785334	0.05	1
	IDP dMRI TBSS L2 Superior cerebellar peduncle L	Wald ratio	0.0866451	0.05	1
	IDP dMRI TBSS L2 Posterior limb of internal capsule R	Inverse variance weighted	0.330215917	0.05	1
	IDP dMRI TBSS L2 Posterior limb of internal capsule L	Wald ratio	0.011390782	0.05	1
	IDP dMRI TBSS L2 Retrolenticular part of internal capsule R	Inverse variance weighted	0.848993835	0.05	1
	IDP dMRI TBSS L2 Anterior corona radiata R	Inverse variance weighted	0.90374445	0.05	1
	IDP dMRI TBSS L2 Anterior corona radiata L	Wald ratio	0.78336801	0.05	1
	IDP dMRI TBSS L2 Superior corona radiata R	Inverse variance weighted	0.785173029	0.05	1
	IDP dMRI TBSS L2 Superior corona radiata L	Wald ratio	0.569160445	0.05	1
	IDP dMRI TBSS L2 Posterior corona radiata R	Wald ratio	0.569160445	0.05	1
	IDP dMRI TBSS L2 Posterior corona radiata L	Wald ratio	0.569160445	0.05	1
	IDP dMRI TBSS L2 Posterior thalamic radiation R	Wald ratio	0.569795936	0.05	1
	IDP dMRI TBSS L2 Posterior thalamic radiation L	Wald ratio	0.569160445	0.05	1
	IDP dMRI TBSS L2 Sagittal stratum R	Wald ratio	0.569160445	0.05	1
	IDP dMRI TBSS L2 Cingulum cingulate gyrus R	Wald ratio	0.78336801	0.05	1
	IDP dMRI TBSS L2 Cingulum hippocampus R	Wald ratio	0.569160445	0.05	1
	IDP dMRI TBSS L2 Cingulum hippocampus L	Wald ratio	0.78336801	0.05	1

	IDP dMRI TBSS L2 Uncinate fasciculus L	Wald ratio	0.78336801	0.05	1
	IDP dMRI TBSS L3 Genu of corpus callosum	Wald ratio	0.78336801	0.05	1
	IDP dMRI TBSS L3 Body of corpus callosum	Wald ratio	0.78336801	0.05	1
	IDP dMRI TBSS L3 Splenium of corpus callosum	Wald ratio	0.014704291	0.05	1
	IDP dMRI TBSS L3 Inferior cerebellar peduncle R	Wald ratio	0.78336801	0.05	1
	IDP dMRI TBSS L3 Inferior cerebellar peduncle L	Wald ratio	0.78336801	0.05	1
	IDP dMRI TBSS L3 Superior cerebellar peduncle R	Inverse variance weighted	0.150070589	0.05	1
	IDP dMRI TBSS L3 Superior cerebellar peduncle L	Wald ratio	0.0866451	0.05	1
	IDP dMRI TBSS L3 Cerebral peduncle R	Wald ratio	0.114873449	0.05	1
	IDP dMRI TBSS L3 Anterior limb of internal capsule R	Wald ratio	0.690132325	0.05	1
	IDP dMRI TBSS L3 Anterior limb of internal capsule L	Wald ratio	0.674510601	0.05	1
	IDP dMRI TBSS L3 Retrolenticular part of internal capsule R	Wald ratio	0.569160445	0.05	1
	IDP dMRI TBSS L3 Anterior corona radiata R	Inverse variance weighted	0.902680108	0.05	1
	IDP dMRI TBSS L3 Anterior corona radiata L	Inverse variance weighted	0.927309432	0.05	1
	IDP dMRI TBSS L3 Posterior corona radiata R	Inverse variance weighted	0.342425112	0.05	1
	IDP dMRI TBSS L3 Posterior thalamic radiation R	Wald ratio	0.569795936	0.05	1
	IDP dMRI TBSS L3 Posterior thalamic radiation L	Wald ratio	0.569160445	0.05	1
	IDP dMRI TBSS L3 Sagittal stratum R	Wald ratio	0.569160445	0.05	1
	IDP dMRI TBSS L3 Sagittal stratum L	Wald ratio	0.569160445	0.05	1

	IDP dMRI TBSS L3 External capsule R	Wald ratio	0.78336801	0.05	1
	IDP dMRI TBSS L3 External capsule L	Wald ratio	0.78336801	0.05	1
	IDP dMRI TBSS L3 Cingulum cingulate gyrus R	Inverse variance weighted	0.919438902	0.05	1
	IDP dMRI TBSS L3 Cingulum cingulate gyrus L	Wald ratio	0.78336801	0.05	1
	IDP dMRI TBSS L3 Cingulum hippocampus R	Wald ratio	0.78336801	0.05	1
	IDP dMRI TBSS L3 Cingulum hippocampus L	Wald ratio	0.78336801	0.05	1
	IDP dMRI TBSS L3 Fornix cres+Stria terminalis R	Wald ratio	0.715132493	0.05	1
	IDP dMRI TBSS L3 Superior longitudinal fasciculus R	Wald ratio	0.569160445	0.05	1
	IDP dMRI TBSS L3 Superior longitudinal fasciculus L	Wald ratio	0.569160445	0.05	1
	IDP dMRI TBSS L3 Uncinate fasciculus R	Wald ratio	0.78336801	0.05	1
	IDP dMRI TBSS L3 Uncinate fasciculus L	Inverse variance weighted	0.710006671	0.05	1
	IDP dMRI TBSS ICVF Middle cerebellar peduncle	Wald ratio	0.569160445	0.05	1
	IDP dMRI TBSS ICVF Genu of corpus callosum	MR Egger	0.914561138	0.05	1
		Weighted median	0.328843089	0.05	1
		Inverse variance weighted	0.156577785	0.05	1
		Simple mode	0.848152361	0.05	1
		Weighted mode	0.963369428	0.05	1
	IDP dMRI TBSS ICVF Body of corpus callosum	MR Egger	0.371725106	0.05	0.38346079
		Weighted median	0.014749466	0.05	0.99952548
		Inverse variance weighted	0.003106754	0.05	0.999416215

		Simple mode	0.179366209	0.05	0.999761952
		Weighted mode	0.128071941	0.05	0.996832685
	IDP dMRI TBSS ICVF Splenium of corpus callosum	MR Egger	0.136618975	0.05	0.004083181
		Weighted median	0.00452535	0.05	0.883071108
		Inverse variance weighted	0.041846619	0.05	0.999900135
		Simple mode	0.133246777	0.05	0.90122803
		Weighted mode	0.080630827	0.05	0.866827267
	IDP dMRI TBSS ICVF Fornix	Wald ratio	0.662854426	0.05	1
	IDP dMRI TBSS ICVF Medial lemniscus R	Inverse variance weighted	0.196246374	0.05	1
	IDP dMRI TBSS ICVF Medial lemniscus L	Wald ratio	0.100070574	0.05	1
	IDP dMRI TBSS ICVF Inferior cerebellar peduncle R	Inverse variance weighted	0.329678155	0.05	1
	IDP dMRI TBSS ICVF Inferior cerebellar peduncle L	Inverse variance weighted	0.329916092	0.05	1
	IDP dMRI TBSS ICVF Superior cerebellar peduncle R	MR Egger	0.432072035	0.05	0.034693491
		Weighted median	0.855609009	0.05	1
		Inverse variance weighted	0.526532503	0.05	1
		Simple mode	0.763489533	0.05	1
		Weighted mode	0.78965489	0.05	1
	IDP dMRI TBSS ICVF Superior cerebellar peduncle L	Inverse variance weighted	0.358631556	0.05	1
	IDP dMRI TBSS ICVF Cerebral peduncle R	MR Egger	0.265687267	0.05	0.005443798
		Weighted median	0.326105262	0.05	1
		Inverse variance weighted	0.417460656	0.05	1
		Simple mode	0.27208413	0.05	0.969068943

		Weighted mode	0.44958411	0.05	1
	IDP dMRI TBSS ICVF Cerebral peduncle L	Wald ratio	0.392397715	0.05	0.977980285
	IDP dMRI TBSS ICVF Anterior limb of internal capsule R	Wald ratio	0.78336801	0.05	1
	IDP dMRI TBSS ICVF Anterior limb of internal capsule L	MR Egger	0.964025825	0.05	1
		Weighted median	0.641023993	0.05	1
		Inverse variance weighted	0.530003773	0.05	1
		Simple mode	0.801660667	0.05	1
		Weighted mode	0.753994196	0.05	1
	IDP dMRI TBSS ICVF Posterior limb of internal capsule R	MR Egger	0.617395597	0.05	1
		Weighted median	0.162460501	0.05	0.999927975
		Inverse variance weighted	0.015957069	0.05	0.968966638
		Simple mode	0.461226814	0.05	0.99999997
		Weighted mode	0.478386884	0.05	0.999999982
	IDP dMRI TBSS ICVF Posterior limb of internal capsule L	MR Egger	0.530946152	0.05	1
		Weighted median	0.695473474	0.05	1
		Inverse variance weighted	0.599793466	0.05	1
		Simple mode	0.481116082	0.05	1
		Weighted mode	0.933906423	0.05	1
	IDP dMRI TBSS ICVF Retrolenticular part of internal capsule R	MR Egger	0.358944994	0.05	1
		Weighted median	0.539119044	0.05	1
		Inverse variance weighted	0.335194298	0.05	1
		Simple mode	0.466391337	0.05	1

		Weighted mode	0.852789909	0.05	1
	IDP dMRI TBSS ICVF Retrolenticular part of internal capsule L	MR Egger	0.520845907	0.05	1
		Weighted median	0.796726874	0.05	1
		Inverse variance weighted	0.669477576	0.05	1
		Simple mode	0.572761125	0.05	1
		Weighted mode	0.949583402	0.05	1
	IDP dMRI TBSS ICVF Anterior corona radiata R	MR Egger	0.519309483	0.05	1
		Weighted median	0.757115586	0.05	1
		Inverse variance weighted	0.62368911	0.05	1
		Simple mode	0.583517392	0.05	1
		Weighted mode	0.973579439	0.05	1
	IDP dMRI TBSS ICVF Anterior corona radiata L	MR Egger	0.524128798	0.05	1
		Weighted median	0.7679949	0.05	1
		Inverse variance weighted	0.64346845	0.05	1
		Simple mode	0.533439907	0.05	1
		Weighted mode	0.946938403	0.05	1
	IDP dMRI TBSS ICVF Superior corona radiata R	MR Egger	0.515841896	0.05	1
		Weighted median	0.743563216	0.05	1
		Inverse variance weighted	0.613158094	0.05	1
		Simple mode	0.615750049	0.05	1
		Weighted mode	0.994991635	0.05	1
		MR Egger	0.517781521	0.05	1

	IDP dMRI TBSS ICVF Superior corona radiata L	Weighted median	0.756825743	0.05	1
		Inverse variance weighted	0.621169407	0.05	1
		Simple mode	0.603940615	0.05	1
		Weighted mode	0.986544102	0.05	1
	IDP dMRI TBSS ICVF Posterior corona radiata R	MR Egger	0.970820955	0.05	1
		Weighted median	0.766894601	0.05	1
		Inverse variance weighted	0.710507124	0.05	1
		Simple mode	0.845807629	0.05	1
		Weighted mode	0.823491374	0.05	1
	IDP dMRI TBSS ICVF Posterior corona radiata L	MR Egger	0.920548265	0.05	1
		Weighted median	0.808268352	0.05	1
		Inverse variance weighted	0.732490797	0.05	1
		Simple mode	0.939427465	0.05	1
		Weighted mode	0.842711671	0.05	1
	IDP dMRI TBSS ICVF Posterior thalamic radiation R	Wald ratio	0.569160445	0.05	1
	IDP dMRI TBSS ICVF Posterior thalamic radiation L	Inverse variance weighted	0.929625772	0.05	1
	IDP dMRI TBSS ICVF Sagittal stratum R	MR Egger	0.499429452	0.05	0.003902709
		Weighted median	0.976054486	0.05	1
		Inverse variance weighted	0.876063319	0.05	1
		Simple mode	0.957514001	0.05	1
		Weighted mode	0.793959362	0.05	1
	IDP dMRI TBSS ICVF Sagittal stratum L	Inverse variance weighted	0.926451551	0.05	1

	IDP dMRI TBSS ICVF External capsule R	MR Egger	0.674292593	0.05	1
		Weighted median	0.757548539	0.05	1
		Inverse variance weighted	0.863437426	0.05	1
		Simple mode	0.683362028	0.05	1
		Weighted mode	0.760023996	0.05	1
	IDP dMRI TBSS ICVF External capsule L	Inverse variance weighted	0.660608822	0.05	1
	IDP dMRI TBSS ICVF Cingulum cingulate gyrus R	Inverse variance weighted	0.98317997	0.05	1
	IDP dMRI TBSS ICVF Cingulum cingulate gyrus L	Wald ratio	0.520001527	0.05	1
	IDP dMRI TBSS ICVF Cingulum hippocampus R	MR Egger	0.919184319	0.05	1
		Weighted median	0.822289364	0.05	1
		Inverse variance weighted	0.841738874	0.05	1
		Simple mode	0.723481829	0.05	1
		Weighted mode	0.81482214	0.05	1
	IDP dMRI TBSS ICVF Cingulum hippocampus L	MR Egger	0.524345045	0.05	1
		Weighted median	0.827608496	0.05	1
		Inverse variance weighted	0.69978929	0.05	1
		Simple mode	0.554996197	0.05	1
		Weighted mode	0.91537507	0.05	1
	IDP dMRI TBSS ICVF Fornix cres+Stria terminalis R	Wald ratio	0.575315089	0.05	1
	IDP dMRI TBSS ICVF Superior longitudinal fasciculus R	Inverse variance weighted	0.274703274	0.05	1
		MR Egger	0.514671656	0.05	1

	IDP dMRI TBSS ICVF Superior longitudinal fasciculus L	Weighted median	0.769994874	0.05	1
		Inverse variance weighted	0.627136854	0.05	1
		Simple mode	0.691028009	0.05	1
		Weighted mode	0.967030793	0.05	1
	IDP dMRI TBSS ICVF Superior fronto-occipital fasciculus R	Inverse variance weighted	0.70165683	0.05	1
	IDP dMRI TBSS ICVF Uncinate fasciculus R	Wald ratio	0.78336801	0.05	1
	IDP dMRI TBSS ICVF Uncinate fasciculus L	Wald ratio	0.78336801	0.05	1
	IDP dMRI TBSS OD Pontine crossing tract	MR Egger	0.407112027	0.05	1
		Weighted median	0.899297188	0.05	1
		Inverse variance weighted	0.663692687	0.05	1
		Simple mode	0.88060674	0.05	1
		Weighted mode	0.853683263	0.05	1
	IDP dMRI TBSS OD Cerebral peduncle R	Wald ratio	0.790384219	0.05	1
	IDP dMRI TBSS OD Cerebral peduncle L	Wald ratio	0.790384219	0.05	1
	IDP dMRI TBSS OD Posterior limb of internal capsule L	Wald ratio	0.186983151	0.05	0.980661407
	IDP dMRI TBSS OD External capsule R	Inverse variance weighted	0.590712793	0.05	1
	IDP dMRI TBSS OD External capsule L	Wald ratio	0.759081795	0.05	1
	IDP dMRI TBSS OD Superior longitudinal fasciculus R	Wald ratio	0.938069051	0.05	1
	IDP dMRI TBSS ISOVF Fornix	Wald ratio	0.097229227	0.05	0.820447514
	IDP dMRI TBSS ISOVF External capsule R	Wald ratio	0.181607821	0.05	1
	IDP dMRI TBSS ISOVF Cingulum cingulate gyrus R	Wald ratio	0.424063091	0.05	1
	IDP dMRI ProbtrackX FA ar l	Wald ratio	0.78336801	0.05	1

	IDP dMRI ProbtrackX FA atr l	Wald ratio	0.78336801	0.05	1
	IDP dMRI ProbtrackX FA fmi	MR Egger	0.268711816	0.05	1
		Weighted median	0.45584518	0.05	1
		Inverse variance weighted	0.230699897	0.05	1
		Simple mode	0.554078444	0.05	1
		Weighted mode	0.838979692	0.05	1
	IDP dMRI ProbtrackX FA ifo l	Wald ratio	0.569160445	0.05	1
	IDP dMRI ProbtrackX FA ifo r	Wald ratio	0.691781486	0.05	1
	IDP dMRI ProbtrackX FA ml l	Wald ratio	0.354006686	0.05	1
	IDP dMRI ProbtrackX FA ptr r	Wald ratio	0.571296941	0.05	1
	IDP dMRI ProbtrackX FA slf l	Wald ratio	0.691781486	0.05	1
	IDP dMRI ProbtrackX FA str l	Wald ratio	0.437899027	0.05	1
	IDP dMRI ProbtrackX FA unc l	Wald ratio	0.78336801	0.05	1
	IDP dMRI ProbtrackX FA unc r	Wald ratio	0.78336801	0.05	1
	IDP dMRI ProbtrackX MD ar l	Wald ratio	0.78336801	0.05	1
	IDP dMRI ProbtrackX MD ar r	Wald ratio	0.78336801	0.05	1
	IDP dMRI ProbtrackX MD atr l	Wald ratio	0.78336801	0.05	1
	IDP dMRI ProbtrackX MD atr r	Wald ratio	0.78336801	0.05	1
	IDP dMRI ProbtrackX MD cgc l	MR Egger	0.648456917	0.05	0.981419106
		Weighted median	0.732226686	0.05	1
		Inverse variance weighted	0.879357739	0.05	1
		Simple mode	0.69118144	0.05	1
		Weighted mode	0.715482589	0.05	1

	IDP dMRI ProbtrackX MD cgc r	Inverse variance weighted	0.89114883	0.05	1
	IDP dMRI ProbtrackX MD fmi	Inverse variance weighted	0.920378508	0.05	1
	IDP dMRI ProbtrackX MD ifo l	Wald ratio	0.569160445	0.05	1
	IDP dMRI ProbtrackX MD ifo r	Wald ratio	0.569160445	0.05	1
	IDP dMRI ProbtrackX MD ilf l	Wald ratio	0.569160445	0.05	1
	IDP dMRI ProbtrackX MD ilf r	Wald ratio	0.569160445	0.05	1
	IDP dMRI ProbtrackX MD ptr l	Wald ratio	0.78336801	0.05	1
	IDP dMRI ProbtrackX MD ptr r	Wald ratio	0.78336801	0.05	1
	IDP dMRI ProbtrackX MD slf l	Inverse variance weighted	0.906622646	0.05	1
	IDP dMRI ProbtrackX MD slf r	Wald ratio	0.569160445	0.05	1
	IDP dMRI ProbtrackX MD str l	Wald ratio	0.260692302	0.05	1
	IDP dMRI ProbtrackX MD str r	Wald ratio	0.78336801	0.05	1
	IDP dMRI ProbtrackX MD unc l	Wald ratio	0.78336801	0.05	1
	IDP dMRI ProbtrackX MD unc r	Inverse variance weighted	0.836674854	0.05	1
	IDP dMRI ProbtrackX MO atr l	Wald ratio	0.629824346	0.05	1
	IDP dMRI ProbtrackX MO ml r	Wald ratio	0.65072791	0.05	1
	IDP T1 FAST ROIs L intracalc cortex	Wald ratio	0.969722601	0.05	1
	IDP dMRI ProbtrackX L1 ilf l	Wald ratio	0.78336801	0.05	1
	IDP T1 FAST ROIs R intracalc cortex	Wald ratio	0.969722601	0.05	1
	IDP dMRI ProbtrackX L1 slf l	Wald ratio	0.78336801	0.05	1
	IDP dMRI ProbtrackX L1 slf r	Wald ratio	0.973338265	0.05	1
	IDP dMRI ProbtrackX L1 str r	Wald ratio	0.842595729	0.05	1

	IDP dMRI ProbtrackX L1 unc l	Inverse variance weighted	0.62931158	0.05	1
	IDP dMRI ProbtrackX L1 unc r	Wald ratio	0.965320293	0.05	1
	IDP dMRI ProbtrackX L2 ar l	Wald ratio	0.78336801	0.05	1
	IDP dMRI ProbtrackX L2 ar r	Wald ratio	0.78336801	0.05	1
	IDP dMRI ProbtrackX L2 atr l	Wald ratio	0.78336801	0.05	1
	IDP dMRI ProbtrackX L2 atr r	Wald ratio	0.78336801	0.05	1
	IDP dMRI ProbtrackX L2 cgh l	Wald ratio	0.584904748	0.05	1
	IDP dMRI ProbtrackX L2 fma	Wald ratio	0.656757894	0.05	1
	IDP dMRI ProbtrackX L2 fmi	Inverse variance weighted	0.919151067	0.05	1
	IDP dMRI ProbtrackX L2 ifo l	Wald ratio	0.569160445	0.05	1
	IDP dMRI ProbtrackX L2 ifo r	Wald ratio	0.569160445	0.05	1
	IDP dMRI ProbtrackX L2 ilf l	Wald ratio	0.569160445	0.05	1
	IDP dMRI ProbtrackX L2 ilf r	Wald ratio	0.569160445	0.05	1
	IDP dMRI ProbtrackX L2 ptr l	Wald ratio	0.78336801	0.05	1
	IDP dMRI ProbtrackX L2 ptr r	Wald ratio	0.791154685	0.05	1
	IDP dMRI ProbtrackX L2 slf l	Inverse variance weighted	0.903114342	0.05	1
	IDP dMRI ProbtrackX L2 slf r	Wald ratio	0.569160445	0.05	1
	IDP dMRI ProbtrackX L2 str l	Wald ratio	0.260692302	0.05	1
	IDP dMRI ProbtrackX L2 str r	Wald ratio	0.260692302	0.05	1
	IDP dMRI ProbtrackX L2 unc l	Wald ratio	0.78336801	0.05	1
	IDP dMRI ProbtrackX L2 unc r	Inverse variance weighted	0.833689915	0.05	1
	IDP dMRI ProbtrackX L3 ar l	Wald ratio	0.78336801	0.05	1

	IDP dMRI ProbtrackX L3 ar r	Wald ratio	0.78336801	0.05	1
	IDP dMRI ProbtrackX L3 atr l	Wald ratio	0.78336801	0.05	1
	IDP dMRI ProbtrackX L3 atr r	Wald ratio	0.78336801	0.05	1
	IDP dMRI ProbtrackX L3 fmi	MR Egger	0.517658993	0.05	0.559333658
		Weighted median	0.693894693	0.05	1
		Inverse variance weighted	0.579747751	0.05	1
		Simple mode	0.541095559	0.05	1
		Weighted mode	0.994678582	0.05	1
	IDP dMRI ProbtrackX L3 ifo l	Inverse variance weighted	0.942042852	0.05	1
	IDP dMRI ProbtrackX L3 ifo r	Wald ratio	0.569160445	0.05	1
	IDP dMRI ProbtrackX L3 ilf l	Wald ratio	0.569160445	0.05	1
	IDP dMRI ProbtrackX L3 ilf r	Wald ratio	0.569160445	0.05	1
	IDP dMRI ProbtrackX L3 ptr l	Wald ratio	0.78336801	0.05	1
	IDP dMRI ProbtrackX L3 ptr r	Wald ratio	0.791154685	0.05	1
	IDP dMRI ProbtrackX L3 slf l	Inverse variance weighted	0.913280904	0.05	1
	IDP dMRI ProbtrackX L3 slf r	Wald ratio	0.569160445	0.05	1
	IDP dMRI ProbtrackX L3 str l	Wald ratio	0.791154685	0.05	1
	IDP dMRI ProbtrackX L3 unc l	Wald ratio	0.78336801	0.05	1
	IDP dMRI ProbtrackX L3 unc r	Inverse variance weighted	0.841864717	0.05	1
	IDP dMRI ProbtrackX ICVF ar l	MR Egger	0.533874403	0.05	1
		Weighted median	0.756821328	0.05	1
		Inverse variance weighted	0.678762611	0.05	1

		Simple mode	0.368024575	0.05	1
		Weighted mode	0.883791784	0.05	1
	IDP dMRI ProbtrackX ICVF ar r	MR Egger	0.528813743	0.05	1
		Weighted median	0.773756604	0.05	1
		Inverse variance weighted	0.669049477	0.05	1
		Simple mode	0.396822262	0.05	1
		Weighted mode	0.885626248	0.05	1
	IDP dMRI ProbtrackX ICVF atr l	MR Egger	0.532282575	0.05	1
		Weighted median	0.782499708	0.05	1
		Inverse variance weighted	0.677279891	0.05	1
		Simple mode	0.436474408	0.05	1
		Weighted mode	0.905280799	0.05	1
	IDP dMRI ProbtrackX ICVF atr r	MR Egger	0.534117913	0.05	1
		Weighted median	0.76079885	0.05	1
		Inverse variance weighted	0.657655072	0.05	1
		Simple mode	0.451399102	0.05	1
		Weighted mode	0.902303	0.05	1
	IDP dMRI ProbtrackX ICVF cgc l	Wald ratio	0.520001527	0.05	1
	IDP dMRI ProbtrackX ICVF cgh l	MR Egger	0.534521259	0.05	1
		Weighted median	0.778291698	0.05	1
		Inverse variance weighted	0.677184275	0.05	1
		Simple mode	0.447197593	0.05	1

		Weighted mode	0.90466454	0.05	1
	IDP dMRI ProbtrackX ICVF cgh r	Wald ratio	0.78336801	0.05	1
	IDP dMRI ProbtrackX ICVF cst l	MR Egger	0.529655645	0.05	1
		Weighted median	0.692049528	0.05	1
		Inverse variance weighted	0.603571	0.05	1
		Simple mode	0.42570044	0.05	1
		Weighted mode	0.911509576	0.05	1
	IDP dMRI ProbtrackX ICVF cst r	MR Egger	0.822011618	0.05	1
		Weighted median	0.955088365	0.05	1
		Inverse variance weighted	0.886361717	0.05	1
		Simple mode	0.665798538	0.05	1
		Weighted mode	0.917736734	0.05	1
	IDP dMRI ProbtrackX ICVF fma	Inverse variance weighted	0.07858014	0.05	0.98366039
	IDP dMRI ProbtrackX ICVF fmi	MR Egger	0.307365235	0.05	1
		Weighted median	0.552058882	0.05	1
		Inverse variance weighted	0.28716265	0.05	1
		Simple mode	0.554609201	0.05	1
		Weighted mode	0.923493177	0.05	1
	IDP dMRI ProbtrackX ICVF ifo l	Inverse variance weighted	0.97083875	0.05	1
	IDP dMRI ProbtrackX ICVF ifo r	Inverse variance weighted	0.968195044	0.05	1
	IDP dMRI ProbtrackX ICVF ilf l	Inverse variance weighted	0.960002976	0.05	1
	IDP dMRI ProbtrackX ICVF ilf r	Wald ratio	0.569160445	0.05	1

	IDP dMRI ProbtrackX ICVF mcp	Wald ratio	0.588824311	0.05	1
	IDP dMRI ProbtrackX ICVF ml l	Wald ratio	0.572555206	0.05	1
	IDP dMRI ProbtrackX ICVF ml r	Wald ratio	0.78336801	0.05	1
	IDP dMRI ProbtrackX ICVF ptr l	Wald ratio	0.569160445	0.05	1
	IDP dMRI ProbtrackX ICVF ptr r	Inverse variance weighted	0.742283889	0.05	1
	IDP dMRI ProbtrackX ICVF slf l	MR Egger	0.517521363	0.05	1
		Weighted median	0.773329407	0.05	1
		Inverse variance weighted	0.638250721	0.05	1
		Simple mode	0.589835385	0.05	1
		Weighted mode	0.984302223	0.05	1
	IDP dMRI ProbtrackX ICVF slf r	Inverse variance weighted	0.271260097	0.05	1
	IDP dMRI ProbtrackX ICVF str l	MR Egger	0.527836342	0.05	1
		Weighted median	0.72121805	0.05	1
		Inverse variance weighted	0.613163431	0.05	1
		Simple mode	0.472017063	0.05	1
		Weighted mode	0.946190012	0.05	1
	IDP dMRI ProbtrackX ICVF str r	MR Egger	0.520515865	0.05	1
		Weighted median	0.740766914	0.05	1
		Inverse variance weighted	0.621005777	0.05	1
		Simple mode	0.564583129	0.05	1
		Weighted mode	0.972009243	0.05	1
	IDP dMRI ProbtrackX ICVF unc l	Inverse variance weighted	0.864640094	0.05	1

	IDP dMRI ProbtrackX ICVF unc r	Inverse variance weighted	0.986930266	0.05	1
	IDP dMRI ProbtrackX OD atr r	Wald ratio	0.429224218	0.05	1
	IDP dMRI ProbtrackX OD ilf l	Wald ratio	0.262863023	0.05	1
	IDP dMRI ProbtrackX OD slf l	Wald ratio	0.695527243	0.05	1
	IDP dMRI ProbtrackX OD str l	Inverse variance weighted	0.645653873	0.05	1
	IDP dMRI ProbtrackX ISOVF atr r	Wald ratio	0.78336801	0.05	1
	IDP dMRI ProbtrackX ISOVF cgc l	Wald ratio	0.879838689	0.05	1
	IDP dMRI ProbtrackX ISOVF ifo r	Wald ratio	0.261277574	0.05	1
	IDP dMRI ProbtrackX ISOVF ilf l	Wald ratio	0.658021547	0.05	1
	IDP dMRI ProbtrackX ISOVF ilf r	MR Egger	0.440972436	0.05	0.00254261
		Weighted median	0.891500206	0.05	1
		Inverse variance weighted	0.984081122	0.05	1
		Simple mode	0.771343585	0.05	1
		Weighted mode	0.765426233	0.05	1
	IDP dMRI ProbtrackX ISOVF slf l	Inverse variance weighted	0.896487235	0.05	1
	IDP dMRI ProbtrackX ISOVF slf r	MR Egger	0.345217973	0.05	0.002542611
		Weighted median	0.927581105	0.05	1
		Inverse variance weighted	0.805344502	0.05	1
		Simple mode	0.791657972	0.05	1
		Weighted mode	0.859995721	0.05	1
	IDP dMRI ProbtrackX ISOVF unc l	Wald ratio	0.988682868	0.05	1
	IDP dMRI ProbtrackX ISOVF unc r	Wald ratio	0.965320293	0.05	1

	IDP T1 SIENAX brain-normalised volume	Wald ratio	0.243814805	0.05	1
Diseases of the musculoskeletal system and connective tissue	IDP T1 SIENAX peripheral grey normalised volume	Wald ratio	0.128883735	0.05	1
	IDP T1 FAST ROIs R temp occ fusif cortex	Wald ratio	0.117877461	0.05	1
	IDP T1 FAST ROIs R occ pole	Inverse variance weighted	0.246131486	0.05	1
	IDP T1 FAST ROIs L thalamus	Inverse variance weighted	0.657888031	0.05	1
	IDP T1 FAST ROIs R thalamus	Wald ratio	0.526209515	0.05	1
	IDP T1 FAST ROIs L putamen	Wald ratio	0.00755939	0.05	1
	IDP T1 FAST ROIs R putamen	Wald ratio	0.00755939	0.05	1
	IDP T1 FAST ROIs L pallidum	Wald ratio	0.932653304	0.05	1
	IDP T1 FIRST left caudate volume	Wald ratio	0.592503776	0.05	1
	IDP T1 FAST ROIs L hippocampus	Inverse variance weighted	0.364602478	0.05	1
	IDP T1 FAST ROIs R hippocampus	Wald ratio	0.240573828	0.05	1
	IDP T1 FAST ROIs L ventral striatum	Wald ratio	0.00755939	0.05	1
	IDP T1 FAST ROIs R ventral striatum	Inverse variance weighted	0.867044979	0.05	1
	IDP T1 FAST ROIs L cerebellum VI	Wald ratio	0.488100389	0.05	1
	IDP T1 FAST ROIs L cerebellum crus I	MR Egger	0.782727212	0.05	1
		Weighted median	0.495527256	0.05	1
		Inverse variance weighted	0.234962756	0.05	1
		Simple mode	0.702347457	0.05	1
		Weighted mode	0.612442545	0.05	1
	IDP T1 FAST ROIs R cerebellum crus I	Inverse variance weighted	0.497211892	0.05	1
	IDP T1 FAST ROIs L cerebellum crus II	MR Egger	0.787049439	0.05	1

		Weighted median	0.257102311	0.05	1
		Inverse variance weighted	0.089668526	0.05	1
		Simple mode	0.526402399	0.05	1
		Weighted mode	0.523504665	0.05	1
	IDP T1 FAST ROIs V cerebellum crus II	Inverse variance weighted	0.496581418	0.05	1
	IDP T1 FAST ROIs R cerebellum crus II	Inverse variance weighted	0.240010336	0.05	1
	IDP T1 FIRST left putamen volume	Inverse variance weighted	0.17375923	0.05	1
	IDP T1 FAST ROIs R cerebellum VIIb	Wald ratio	0.838585048	0.05	1
	IDP T1 FAST ROIs V cerebellum VIIIa	MR Egger	0.922053529	0.05	1
		Weighted median	0.225400775	0.05	1
		Inverse variance weighted	0.780808986	0.05	1
		Simple mode	0.327145775	0.05	1
		Weighted mode	0.317553924	0.05	1
	IDP T1 FAST ROIs L cerebellum VIIIb	Wald ratio	0.012748945	0.05	1
	IDP T1 FAST ROIs V cerebellum VIIIb	Inverse variance weighted	0.96795733	0.05	1
	IDP T1 FAST ROIs R cerebellum VIIIb	Wald ratio	0.00755939	0.05	1
	IDP T1 FAST ROIs L cerebellum IX	Inverse variance weighted	0.627320546	0.05	1
	IDP T1 FIRST right putamen volume	Wald ratio	0.588824311	0.05	1
	IDP T1 FAST ROIs V cerebellum IX	Inverse variance weighted	0.499796975	0.05	1
	IDP T1 FAST ROIs R cerebellum IX	Inverse variance weighted	0.614542959	0.05	1
	IDP T1 FAST ROIs V cerebellum X	Wald ratio	0.00755939	0.05	1

	IDP T2 FLAIR BIANCA WMH volume	Inverse variance weighted	0.687803222	0.05	1
	IDP SWI T2star left caudate	MR Egger	0.260992446	0.05	1
		Weighted median	0.562467657	0.05	1
		Inverse variance weighted	0.900839628	0.05	1
		Simple mode	0.668811705	0.05	1
		Weighted mode	0.247806199	0.05	1
	IDP SWI T2star right caudate	MR Egger	0.937875395	0.05	1
		Weighted median	0.840828676	0.05	1
		Inverse variance weighted	0.407405094	0.05	1
		Simple mode	0.280471264	0.05	1
		Weighted mode	0.851876276	0.05	1
	IDP T1 FIRST left pallidum volume	Wald ratio	0.095580705	0.05	1
	IDP SWI T2star left putamen	MR Egger	0.932889067	0.05	1
		Weighted median	0.370824442	0.05	1
		Inverse variance weighted	0.094344484	0.05	1
		Simple mode	0.703355631	0.05	1
		Weighted mode	0.860670659	0.05	1
	IDP SWI T2star right putamen	MR Egger	0.983058274	0.05	1
		Weighted median	0.653624324	0.05	1
		Inverse variance weighted	0.559975694	0.05	1
		Simple mode	0.643930681	0.05	1
		Weighted mode	0.610165636	0.05	1

	IDP SWI T2star left pallidum	MR Egger	0.611776876	0.05	1
		Weighted median	0.046003338	0.05	1
		Inverse variance weighted	0.031801206	0.05	1
		Simple mode	0.516764927	0.05	1
		Weighted mode	0.19085096	0.05	1
	IDP SWI T2star right pallidum	MR Egger	0.140442927	0.05	1
		Weighted median	0.971306753	0.05	1
		Inverse variance weighted	0.542465339	0.05	1
		Simple mode	0.631392896	0.05	1
		Weighted mode	0.751683166	0.05	1
	IDP T1 FIRST right pallidum volume	Wald ratio	0.095580705	0.05	1
	IDP dMRI TBSS FA Genu of corpus callosum	Wald ratio	0.990026703	0.05	1
	IDP dMRI TBSS FA Splenium of corpus callosum	Inverse variance weighted	0.402715199	0.05	1
	IDP dMRI TBSS FA Corticospinal tract L	Wald ratio	0.713867673	0.05	1
	IDP dMRI TBSS FA Superior cerebellar peduncle R	Inverse variance weighted	0.324974282	0.05	1
	IDP dMRI TBSS FA Superior cerebellar peduncle L	Inverse variance weighted	0.261471082	0.05	1
	IDP dMRI TBSS FA Posterior limb of internal capsule R	Wald ratio	0.563625518	0.05	1
	IDP dMRI TBSS FA Retrolenticular part of internal capsule R	Inverse variance weighted	0.769114064	0.05	1
	IDP dMRI TBSS FA Anterior corona radiata R	Wald ratio	0.990026703	0.05	1
	IDP dMRI TBSS FA Anterior corona radiata L	Wald ratio	0.990026703	0.05	1

	IDP dMRI TBSS FA Posterior thalamic radiation R	Wald ratio	0.948225702	0.05	1
	IDP dMRI TBSS FA Posterior thalamic radiation L	Wald ratio	0.937890037	0.05	1
	IDP dMRI TBSS FA Sagittal stratum R	Wald ratio	0.625688056	0.05	1
	IDP dMRI TBSS FA Cingulum cingulate gyrus R	Inverse variance weighted	0.757305265	0.05	1
	IDP dMRI TBSS FA Superior longitudinal fasciculus L	Inverse variance weighted	0.675903305	0.05	1
	IDP dMRI TBSS FA Uncinate fasciculus L	Wald ratio	0.065459754	0.05	1
	IDP dMRI TBSS MD Genu of corpus callosum	Wald ratio	0.990026703	0.05	1
	IDP dMRI TBSS MD Body of corpus callosum	Wald ratio	0.13838868	0.05	1
	IDP dMRI TBSS MD Splenium of corpus callosum	Inverse variance weighted	0.353421574	0.05	1
	IDP dMRI TBSS MD Corticospinal tract R	Wald ratio	0.672634405	0.05	1
	IDP dMRI TBSS MD Superior cerebellar peduncle R	Wald ratio	0.085659903	0.05	1
	IDP dMRI TBSS MD Superior cerebellar peduncle L	Wald ratio	0.113157674	0.05	1
	IDP dMRI TBSS MD Anterior limb of internal capsule L	Wald ratio	0.890829316	0.05	1
	IDP dMRI TBSS MD Retrolenticular part of internal capsule R	Wald ratio	0.958570086	0.05	1
	IDP T1 FIRST left caudate volume plus IDP T1 FIRST right caudate volume	Wald ratio	0.167664607	0.05	1
	IDP T1 FIRST left putamen volume plus IDP T1 FIRST right putamen volume	Inverse variance weighted	0.251131081	0.05	1
	IDP T1 FIRST left pallidum volume plus IDP T1 FIRST right pallidum volume	Wald ratio	0.095580705	0.05	1
	IDP SWI T2star left thalamus plus IDP SWI T2star right thalamus	MR Egger	0.392477577	0.05	1
		Weighted median	0.759122074	0.05	1

		Inverse variance weighted	0.405980317	0.05	1
		Simple mode	0.906029819	0.05	1
		Weighted mode	0.886515386	0.05	1
	IDP dMRI TBSS MD Retrolenticular part of internal capsule L	Wald ratio	0.958570086	0.05	1
	IDP SWI T2star left caudate plus IDP SWI T2star right caudate	MR Egger	0.843460383	0.05	1
		Weighted median	0.868744895	0.05	1
		Inverse variance weighted	0.447456735	0.05	1
		Simple mode	0.743698787	0.05	1
		Weighted mode	0.673952159	0.05	1
	IDP SWI T2star left putamen plus IDP SWI T2star right putamen	MR Egger	0.770450639	0.05	1
		Weighted median	0.856047372	0.05	1
		Inverse variance weighted	0.399224795	0.05	1
		Simple mode	0.775468519	0.05	1
		Weighted mode	0.738288365	0.05	1
	IDP SWI T2star left pallidum plus IDP SWI T2star right pallidum	MR Egger	0.418954429	0.05	1
		Weighted median	0.900491773	0.05	1
		Inverse variance weighted	0.449661636	0.05	1
		Simple mode	0.919162769	0.05	1
		Weighted mode	0.960142323	0.05	1
	volume Left-Lateral-Ventricle	MR Egger	0.575716519	0.05	1
		Weighted median	0.020350925	0.05	1
		Inverse variance weighted	0.005967069	0.05	1

		Simple mode	0.146296495	0.05	1
		Weighted mode	0.206432541	0.05	1
	volume Left-Cerebellum-White-Matter	Inverse variance weighted	0.997691607	0.05	1
	volume Left-Cerebellum-Cortex	Inverse variance weighted	0.682520083	0.05	1
	IDP dMRI TBSS MD Anterior corona radiata R	Inverse variance weighted	0.982942024	0.05	1
	volume Left-Putamen	Inverse variance weighted	0.928071783	0.05	1
	volume 4th-Ventricle	Inverse variance weighted	0.260747021	0.05	1
	volume Brain-Stem	Wald ratio	0.019156792	0.05	1
	volume Left-Hippocampus	Inverse variance weighted	0.606549327	0.05	1
	volume CSF	MR Egger	0.785911994	0.05	1
		Weighted median	0.932433022	0.05	1
		Inverse variance weighted	0.872910423	0.05	1
		Simple mode	0.999747992	0.05	1
		Weighted mode	0.992040678	0.05	1
	IDP dMRI TBSS MD Anterior corona radiata L	Inverse variance weighted	0.984686856	0.05	1
	volume Left-Accumbens-area	Wald ratio	0.00755939	0.05	1
	volume Right-Lateral-Ventricle	Inverse variance weighted	0.013871673	0.05	1
	volume Right-Inf-Lat-Vent	Wald ratio	0.232423524	0.05	1
	volume Right-Cerebellum-White-Matter	Wald ratio	0.023804252	0.05	1
	volume Right-Cerebellum-Cortex	MR Egger	0.749915383	0.05	1
		Weighted median	0.64279795	0.05	1

		Inverse variance weighted	0.608059108	0.05	1
		Simple mode	0.851032803	0.05	1
		Weighted mode	0.663552584	0.05	1
	IDP dMRI TBSS MD Superior corona radiata R	Inverse variance weighted	0.982976306	0.05	1
	volume Right-Putamen	Wald ratio	0.588824311	0.05	1
	volume Right-Hippocampus	Wald ratio	0.240573828	0.05	1
	volume Right-Amygdala	Wald ratio	0.285085208	0.05	1
	volume Right-VentralDC	Wald ratio	0.067075347	0.05	1
	volume CC Posterior	Wald ratio	0.617075077	0.05	1
	IDP dMRI TBSS MD Superior corona radiata L	Inverse variance weighted	0.983160611	0.05	1
	IDP dMRI TBSS MD Posterior corona radiata R	Wald ratio	0.958570086	0.05	1
	volume BrainSegVol-to-eTIV	Wald ratio	0.164454826	0.05	1
	IDP dMRI TBSS MD Posterior corona radiata L	Wald ratio	0.937890037	0.05	1
	volume rhSurfaceHoles	Wald ratio	0.691175539	0.05	1
	DKTatlas lh cuneus area	Wald ratio	0.233292932	0.05	1
	IDP dMRI TBSS MD Posterior thalamic radiation R	Wald ratio	0.937890037	0.05	1
	DKTatlas lh lateraloccipital area	Wald ratio	0.233292932	0.05	1
	DKTatlas lh lingual area	Wald ratio	0.253097909	0.05	1
	IDP dMRI TBSS MD Posterior thalamic radiation L	Wald ratio	0.958570086	0.05	1
	DKTatlas lh parstriangularis area	Wald ratio	0.453254705	0.05	1
	DKTatlas lh pericalcarine area	Inverse variance weighted	0.032217589	0.05	1

	DKTatlas lh postcentral area	Inverse variance weighted	0.793798589	0.05	1
	DKTatlas lh posteriorcingulate area	Wald ratio	0.571627267	0.05	1
	DKTatlas lh precentral area	Wald ratio	0.225748991	0.05	1
	DKTatlas lh precuneus area	Wald ratio	0.585440935	0.05	1
	IDP dMRI TBSS MD Sagittal stratum R	Wald ratio	0.958570086	0.05	1
	DKTatlas lh superiorparietal area	Wald ratio	0.201492498	0.05	1
	DKTatlas lh superiortemporal area	Wald ratio	0.21821348	0.05	1
	DKTatlas lh supramarginal area	Wald ratio	0.069248678	0.05	1
	DKTatlas lh WhiteSurfArea area	Wald ratio	0.081358572	0.05	1
	IDP dMRI TBSS MD Sagittal stratum L	Wald ratio	0.958570086	0.05	1
	a2009s lh G&S subcentral area	Wald ratio	0.225748991	0.05	1
	a2009s lh G cuneus area	Wald ratio	0.206723525	0.05	1
	a2009s lh G front inf-Opercular area	Wald ratio	0.116083134	0.05	1
	IDP dMRI TBSS MD External capsule R	Wald ratio	0.990026703	0.05	1
	IDP dMRI TBSS MD External capsule L	Inverse variance weighted	0.930568297	0.05	1
	a2009s lh G pariet inf-Supramar area	Wald ratio	0.069248678	0.05	1
	a2009s lh G parietal sup area	Wald ratio	0.204474035	0.05	1
	a2009s lh G postcentral area	Wald ratio	0.201492498	0.05	1
	a2009s lh G precentral area	Wald ratio	0.225748991	0.05	1
	a2009s lh G precuneus area	Wald ratio	0.206723525	0.05	1
	IDP dMRI TBSS MD Cingulum cingulate gyrus R	MR Egger	0.96049817	0.05	1
		Weighted median	0.961578489	0.05	1
		Inverse variance weighted	0.931015713	0.05	1

		Simple mode	0.968417587	0.05	1
		Weighted mode	0.98859896	0.05	1
	IDP dMRI TBSS MD Cingulum cingulate gyrus L	MR Egger	0.963039142	0.05	1
		Weighted median	0.963599234	0.05	1
		Inverse variance weighted	0.930479042	0.05	1
		Simple mode	0.96482137	0.05	1
		Weighted mode	0.988538975	0.05	1
	a2009s lh S calcarine area	Wald ratio	0.253097909	0.05	1
	a2009s lh S central area	Wald ratio	0.225748991	0.05	1
	a2009s lh S collat transv ant area	Wald ratio	0.212429187	0.05	1
	a2009s lh S front middle area	Wald ratio	0.391281243	0.05	1
	IDP dMRI TBSS MD Cingulum hippocampus R	Wald ratio	0.958570086	0.05	1
	a2009s lh S intrapariet&P trans area	Wald ratio	0.204474035	0.05	1
	IDP dMRI TBSS MD Cingulum hippocampus L	Wald ratio	0.958570086	0.05	1
	a2009s lh S subparietal area	Wald ratio	0.253097909	0.05	1
	DKTatlas rh cuneus area	Wald ratio	0.253097909	0.05	1
	DKTatlas rh lingual area	Wald ratio	0.253097909	0.05	1
	DKTatlas rh parstriangularis area	Wald ratio	0.036640839	0.05	1
	DKTatlas rh pericalcarine area	Inverse variance weighted	0.554055266	0.05	1
	IDP dMRI TBSS MD Superior longitudinal fasciculus R	Wald ratio	0.958570086	0.05	1
	DKTatlas rh postcentral area	Wald ratio	0.225748991	0.05	1
	DKTatlas rh precentral area	Wald ratio	0.225748991	0.05	1

	IDP dMRI TBSS MD Superior longitudinal fasciculus L	Wald ratio	0.958570086	0.05	1
	a2009s rh G&S subcentral area	Wald ratio	0.225748991	0.05	1
	a2009s rh G&S cingul-Mid-Post area	Wald ratio	0.223243461	0.05	1
	a2009s rh G cuneus area	Wald ratio	0.253097909	0.05	1
	IDP dMRI TBSS MD Superior fronto-occipital fasciculus L	Wald ratio	0.142978567	0.05	1
	a2009s rh G oc-temp med-Lingual area	Wald ratio	0.253097909	0.05	1
	a2009s rh G parietal sup area	Wald ratio	0.070790633	0.05	1
	IDP dMRI TBSS MD Uncinate fasciculus R	Wald ratio	0.875353021	0.05	1
	a2009s rh G precentral area	Wald ratio	0.225748991	0.05	1
	a2009s rh G precuneus area	Wald ratio	0.955072525	0.05	1
	IDP dMRI TBSS MD Uncinate fasciculus L	Inverse variance weighted	0.94222247	0.05	1
	a2009s rh Pole occipital area	Wald ratio	0.253097909	0.05	1
	a2009s rh S calcarine area	Wald ratio	0.253097909	0.05	1
	a2009s rh S central area	Wald ratio	0.225748991	0.05	1
	a2009s rh S collat transv ant area	Wald ratio	0.212429187	0.05	1
	a2009s rh S orbital med-olfact area	Wald ratio	0.250582302	0.05	1
	IDP dMRI TBSS MO Pontine crossing tract	MR Egger	0.793313689	0.05	1
		Weighted median	0.313582002	0.05	1
		Inverse variance weighted	0.548068616	0.05	1
		Simple mode	0.579098738	0.05	1
		Weighted mode	0.464153371	0.05	1
	DKTatlas lh postcentral thickness	Wald ratio	0.225748991	0.05	1

	a2009s lh G insular short thickness	Wald ratio	0.295380199	0.05	1
	a2009s lh G postcentral thickness	Wald ratio	0.225748991	0.05	1
	IDP T1 SIENAX CSF normalised volume	Inverse variance weighted	0.047463831	0.05	1
	IDP dMRI TBSS MO Medial lemniscus R	Wald ratio	0.094312154	0.05	1
	a2009s lh S postcentral thickness	Wald ratio	0.716795308	0.05	1
	DKTatlas rh lateraloccipital thickness	Wald ratio	0.00755939	0.05	1
	DKTatlas rh parstriangularis thickness	Wald ratio	0.310347685	0.05	1
	DKTatlas rh postcentral thickness	Wald ratio	0.225748991	0.05	1
	DKTatlas rh posteriorcingulate thickness	Inverse variance weighted	0.67148463	0.05	1
	a2009s rh G&S cingul-Mid-Ant thickness	Wald ratio	0.992844181	0.05	1
	a2009s rh G cuneus thickness	Wald ratio	0.00755939	0.05	1
	a2009s rh G postcentral thickness	Wald ratio	0.225748991	0.05	1
	a2009s rh Pole occipital thickness	Wald ratio	0.00755939	0.05	1
	a2009s rh S oc sup&transversal thickness	Wald ratio	0.399019591	0.05	1
	a2009s rh S parieto occipital thickness	Wald ratio	0.867483476	0.05	1
	IDP dMRI TBSS MO Anterior corona radiata R	Wald ratio	0.039749945	0.05	1
	IDP dMRI TBSS MO Superior corona radiata R	Wald ratio	0.817494086	0.05	1
	IDP dMRI TBSS MO Cingulum cingulate gyrus L	Inverse variance weighted	0.017434779	0.05	1
	IDP dMRI TBSS L1 Anterior limb of internal capsule L	Wald ratio	0.890829316	0.05	1
	IDP dMRI TBSS L1 Posterior limb of internal capsule R	Wald ratio	0.605258128	0.05	1
	IDP dMRI TBSS L1 Posterior limb of internal capsule L	Wald ratio	0.966296597	0.05	1

	IDP dMRI TBSS L1 Anterior corona radiata R	Wald ratio	0.990026703	0.05	1
	IDP dMRI TBSS L1 Anterior corona radiata L	Wald ratio	0.990026703	0.05	1
	IDP dMRI TBSS L1 Posterior corona radiata R	Wald ratio	0.598275729	0.05	1
	IDP dMRI TBSS L1 Posterior corona radiata L	Wald ratio	0.990026703	0.05	1
	IDP dMRI TBSS L1 Sagittal stratum L	Wald ratio	0.990026703	0.05	1
	IDP dMRI TBSS L1 External capsule R	Wald ratio	0.990026703	0.05	1
	IDP dMRI TBSS L1 External capsule L	Inverse variance weighted	0.696124445	0.05	1
	IDP dMRI TBSS L1 Fornix cres+Stria terminalis L	Wald ratio	0.873400488	0.05	1
	IDP T1 FAST ROIs L precentral gyrus	Wald ratio	0.225748991	0.05	1
	IDP dMRI TBSS L1 Superior longitudinal fasciculus R	Inverse variance weighted	0.474882047	0.05	1
	IDP dMRI TBSS L1 Uncinate fasciculus L	Wald ratio	0.827469068	0.05	1
	IDP dMRI TBSS L2 Pontine crossing tract	Wald ratio	0.094312154	0.05	1
	IDP dMRI TBSS L2 Genu of corpus callosum	MR Egger	0.551520096	0.05	1
		Weighted median	0.243513157	0.05	1
		Inverse variance weighted	0.25939923	0.05	1
		Simple mode	0.30535992	0.05	1
		Weighted mode	0.991504636	0.05	1
	IDP dMRI TBSS L2 Body of corpus callosum	Wald ratio	0.990026703	0.05	1
	IDP dMRI TBSS L2 Splenium of corpus callosum	MR Egger	0.630364909	0.05	1
		Weighted median	0.863400837	0.05	1
		Inverse variance weighted	0.523877037	0.05	1

		Simple mode	0.989589546	0.05	1
		Weighted mode	0.98710475	0.05	1
	IDP dMRI TBSS L2 Corticospinal tract R	Wald ratio	0.672634405	0.05	1
	IDP T1 SIENAX CSF unnormalised volume	MR Egger	0.627444665	0.05	1
		Weighted median	0.018509655	0.05	1
		Inverse variance weighted	0.006247677	0.05	1
		Simple mode	0.145831484	0.05	1
		Weighted mode	0.222949603	0.05	1
	IDP T1 FAST ROIs L temporal pole	Wald ratio	0.13202281	0.05	1
	IDP dMRI TBSS L2 Superior cerebellar peduncle R	Inverse variance weighted	0.303424887	0.05	1
	IDP dMRI TBSS L2 Superior cerebellar peduncle L	Wald ratio	0.113157674	0.05	1
	IDP dMRI TBSS L2 Posterior limb of internal capsule R	Inverse variance weighted	0.235810088	0.05	1
	IDP dMRI TBSS L2 Posterior limb of internal capsule L	Wald ratio	0.274247473	0.05	1
	IDP dMRI TBSS L2 Retrolenticular part of internal capsule R	Inverse variance weighted	0.978887293	0.05	1
	IDP dMRI TBSS L2 Anterior corona radiata R	Inverse variance weighted	0.983212063	0.05	1
	IDP dMRI TBSS L2 Anterior corona radiata L	Wald ratio	0.990026703	0.05	1
	IDP dMRI TBSS L2 Superior corona radiata R	Inverse variance weighted	0.540187764	0.05	1
	IDP dMRI TBSS L2 Superior corona radiata L	Wald ratio	0.958570086	0.05	1
	IDP dMRI TBSS L2 Posterior corona radiata R	Wald ratio	0.937890037	0.05	1
	IDP dMRI TBSS L2 Posterior corona radiata L	Wald ratio	0.937890037	0.05	1

	IDP dMRI TBSS L2 Posterior thalamic radiation R	Wald ratio	0.948225702	0.05	1
	IDP dMRI TBSS L2 Posterior thalamic radiation L	Wald ratio	0.937890037	0.05	1
	IDP dMRI TBSS L2 Sagittal stratum R	Wald ratio	0.958570086	0.05	1
	IDP dMRI TBSS L2 Cingulum cingulate gyrus R	Wald ratio	0.990026703	0.05	1
	IDP dMRI TBSS L2 Cingulum hippocampus R	Wald ratio	0.958570086	0.05	1
	IDP dMRI TBSS L2 Cingulum hippocampus L	Wald ratio	0.990026703	0.05	1
	IDP dMRI TBSS L2 Uncinate fasciculus L	Wald ratio	0.990026703	0.05	1
	IDP dMRI TBSS L3 Genu of corpus callosum	Wald ratio	0.990026703	0.05	1
	IDP dMRI TBSS L3 Body of corpus callosum	Wald ratio	0.990026703	0.05	1
	IDP dMRI TBSS L3 Splenium of corpus callosum	Wald ratio	0.298339901	0.05	1
	IDP dMRI TBSS L3 Inferior cerebellar peduncle R	Wald ratio	0.990026703	0.05	1
	IDP dMRI TBSS L3 Inferior cerebellar peduncle L	Wald ratio	0.990026703	0.05	1
	IDP dMRI TBSS L3 Superior cerebellar peduncle R	Inverse variance weighted	0.276113414	0.05	1
	IDP dMRI TBSS L3 Superior cerebellar peduncle L	Wald ratio	0.113157674	0.05	1
	IDP dMRI TBSS L3 Cerebral peduncle R	Wald ratio	0.7873263	0.05	1
	IDP dMRI TBSS L3 Anterior limb of internal capsule R	Wald ratio	0.215518824	0.05	1
	IDP dMRI TBSS L3 Anterior limb of internal capsule L	Wald ratio	0.022739821	0.05	1
	IDP dMRI TBSS L3 Retrolenticular part of internal capsule R	Wald ratio	0.958570086	0.05	1
	IDP dMRI TBSS L3 Anterior corona radiata R	Inverse variance weighted	0.983127311	0.05	1

	IDP dMRI TBSS L3 Anterior corona radiata L	Inverse variance weighted	0.985095872	0.05	1
	IDP dMRI TBSS L3 Posterior corona radiata R	Inverse variance weighted	0.438322556	0.05	1
	IDP dMRI TBSS L3 Posterior thalamic radiation R	Wald ratio	0.948225702	0.05	1
	IDP dMRI TBSS L3 Posterior thalamic radiation L	Wald ratio	0.958570086	0.05	1
	IDP dMRI TBSS L3 Sagittal stratum R	Wald ratio	0.958570086	0.05	1
	IDP dMRI TBSS L3 Sagittal stratum L	Wald ratio	0.958570086	0.05	1
	IDP dMRI TBSS L3 External capsule R	Wald ratio	0.990026703	0.05	1
	IDP dMRI TBSS L3 External capsule L	Wald ratio	0.990026703	0.05	1
	IDP dMRI TBSS L3 Cingulum cingulate gyrus R	Inverse variance weighted	0.9844651	0.05	1
	IDP dMRI TBSS L3 Cingulum cingulate gyrus L	Wald ratio	0.990026703	0.05	1
	IDP dMRI TBSS L3 Cingulum hippocampus R	Wald ratio	0.990026703	0.05	1
	IDP dMRI TBSS L3 Cingulum hippocampus L	Wald ratio	0.990026703	0.05	1
	IDP dMRI TBSS L3 Fornix cres+Stria terminalis R	Wald ratio	0.943056671	0.05	1
	IDP dMRI TBSS L3 Superior longitudinal fasciculus R	Wald ratio	0.958570086	0.05	1
	IDP dMRI TBSS L3 Superior longitudinal fasciculus L	Wald ratio	0.958570086	0.05	1
	IDP dMRI TBSS L3 Uncinate fasciculus R	Wald ratio	0.990026703	0.05	1
	IDP dMRI TBSS L3 Uncinate fasciculus L	Inverse variance weighted	0.420600143	0.05	1
	IDP dMRI TBSS ICVF Middle cerebellar peduncle	Wald ratio	0.958570086	0.05	1
	IDP dMRI TBSS ICVF Genu of corpus callosum	MR Egger	0.914911897	0.05	1
		Weighted median	0.587675711	0.05	1

		Inverse variance weighted	0.305637253	0.05	1
		Simple mode	0.840716125	0.05	1
		Weighted mode	0.886525568	0.05	1
	IDP dMRI TBSS ICVF Body of corpus callosum	MR Egger	0.592532824	0.05	1
		Weighted median	0.564124611	0.05	1
		Inverse variance weighted	0.40287873	0.05	1
		Simple mode	0.951032661	0.05	1
		Weighted mode	0.442104152	0.05	1
	IDP dMRI TBSS ICVF Splenium of corpus callosum	MR Egger	0.575370516	0.05	1
		Weighted median	0.178461142	0.05	1
		Inverse variance weighted	0.435261746	0.05	1
		Simple mode	0.361228765	0.05	1
		Weighted mode	0.328208652	0.05	1
	IDP dMRI TBSS ICVF Fornix	Wald ratio	0.142978567	0.05	1
	IDP dMRI TBSS ICVF Medial lemniscus R	Inverse variance weighted	0.968505642	0.05	1
	IDP dMRI TBSS ICVF Medial lemniscus L	Wald ratio	0.455378189	0.05	1
	IDP dMRI TBSS ICVF Inferior cerebellar peduncle R	Inverse variance weighted	0.579959112	0.05	1
	IDP dMRI TBSS ICVF Inferior cerebellar peduncle L	Inverse variance weighted	0.584276799	0.05	1
	IDP dMRI TBSS ICVF Superior cerebellar peduncle R	MR Egger	0.412209814	0.05	1
		Weighted median	0.665495234	0.05	1
		Inverse variance weighted	0.426342233	0.05	1
		Simple mode	0.82764736	0.05	1

		Weighted mode	0.902373044	0.05	1
	IDP dMRI TBSS ICVF Superior cerebellar peduncle L	Inverse variance weighted	0.217881723	0.05	1
	IDP dMRI TBSS ICVF Cerebral peduncle R	MR Egger	0.186650156	0.05	1
		Weighted median	0.022203701	0.05	1
		Inverse variance weighted	0.002466912	0.05	1
		Simple mode	0.150705071	0.05	1
		Weighted mode	0.139438721	0.05	1
	IDP dMRI TBSS ICVF Cerebral peduncle L	Wald ratio	0.012748945	0.05	1
	IDP dMRI TBSS ICVF Anterior limb of internal capsule R	Wald ratio	0.990026703	0.05	1
	IDP dMRI TBSS ICVF Anterior limb of internal capsule L	MR Egger	0.580973703	0.05	1
		Weighted median	0.934759843	0.05	1
		Inverse variance weighted	0.729026213	0.05	1
		Simple mode	0.802047955	0.05	1
		Weighted mode	0.925900827	0.05	1
	IDP dMRI TBSS ICVF Posterior limb of internal capsule R	MR Egger	0.515971056	0.05	1
		Weighted median	0.127235916	0.05	1
		Inverse variance weighted	0.198430509	0.05	1
		Simple mode	0.334506163	0.05	1
		Weighted mode	0.303913857	0.05	1
	IDP dMRI TBSS ICVF Posterior limb of internal capsule L	MR Egger	0.712967741	0.05	1
		Weighted median	0.992309764	0.05	1
		Inverse variance weighted	0.628549994	0.05	1

		Simple mode	0.976779801	0.05	1
		Weighted mode	0.991143297	0.05	1
	IDP dMRI TBSS ICVF Retrolenticular part of internal capsule R	MR Egger	0.835322489	0.05	1
		Weighted median	0.990605366	0.05	1
		Inverse variance weighted	0.807944851	0.05	1
		Simple mode	0.996285243	0.05	1
		Weighted mode	0.995166763	0.05	1
	IDP dMRI TBSS ICVF Retrolenticular part of internal capsule L	MR Egger	0.666182319	0.05	1
		Weighted median	0.9978333	0.05	1
		Inverse variance weighted	0.69029824	0.05	1
		Simple mode	0.978254977	0.05	1
		Weighted mode	0.991928428	0.05	1
	IDP dMRI TBSS ICVF Anterior corona radiata R	MR Egger	0.657085252	0.05	1
		Weighted median	0.997490164	0.05	1
		Inverse variance weighted	0.663155489	0.05	1
		Simple mode	0.981066565	0.05	1
		Weighted mode	0.992997658	0.05	1
	IDP dMRI TBSS ICVF Anterior corona radiata L	MR Egger	0.683197703	0.05	1
		Weighted median	0.995608117	0.05	1
		Inverse variance weighted	0.666004905	0.05	1
		Simple mode	0.976237106	0.05	1
		Weighted mode	0.992672461	0.05	1

	IDP dMRI TBSS ICVF Superior corona radiata R	MR Egger	0.629898487	0.05	1
		Weighted median	0.999705516	0.05	1
		Inverse variance weighted	0.666819693	0.05	1
		Simple mode	0.977718418	0.05	1
		Weighted mode	0.991912766	0.05	1
	IDP dMRI TBSS ICVF Superior corona radiata L	MR Egger	0.646790158	0.05	1
		Weighted median	0.998452765	0.05	1
		Inverse variance weighted	0.665545263	0.05	1
		Simple mode	0.975664617	0.05	1
		Weighted mode	0.989824647	0.05	1
	IDP dMRI TBSS ICVF Posterior corona radiata R	MR Egger	0.76922239	0.05	1
		Weighted median	0.960347468	0.05	1
		Inverse variance weighted	0.714579081	0.05	1
		Simple mode	0.977603016	0.05	1
		Weighted mode	0.993547924	0.05	1
	IDP dMRI TBSS ICVF Posterior corona radiata L	MR Egger	0.751278447	0.05	1
		Weighted median	0.965450351	0.05	1
		Inverse variance weighted	0.733782958	0.05	1
		Simple mode	0.97652206	0.05	1
		Weighted mode	0.992448796	0.05	1
	IDP dMRI TBSS ICVF Posterior thalamic radiation R	Wald ratio	0.958570086	0.05	1
	IDP dMRI TBSS ICVF Posterior thalamic radiation L	Inverse variance weighted	0.679895536	0.05	1

	IDP dMRI TBSS ICVF Sagittal stratum R	MR Egger	0.864282067	0.05	1
		Weighted median	0.3486648	0.05	1
		Inverse variance weighted	0.190559922	0.05	1
		Simple mode	0.515633747	0.05	1
		Weighted mode	0.689268681	0.05	1
	IDP dMRI TBSS ICVF Sagittal stratum L	Inverse variance weighted	0.617494888	0.05	1
	IDP dMRI TBSS ICVF External capsule R	MR Egger	0.549073418	0.05	1
		Weighted median	0.908476702	0.05	1
		Inverse variance weighted	0.59907788	0.05	1
		Simple mode	0.910536747	0.05	1
		Weighted mode	0.97512802	0.05	1
	IDP dMRI TBSS ICVF External capsule L	Inverse variance weighted	0.959523709	0.05	1
	IDP dMRI TBSS ICVF Cingulum cingulate gyrus R	Inverse variance weighted	0.989623692	0.05	1
	IDP dMRI TBSS ICVF Cingulum cingulate gyrus L	Wald ratio	0.185395355	0.05	1
	IDP dMRI TBSS ICVF Cingulum hippocampus R	MR Egger	0.699680984	0.05	1
		Weighted median	0.994578484	0.05	1
		Inverse variance weighted	0.699186347	0.05	1
		Simple mode	0.969437529	0.05	1
		Weighted mode	0.993934153	0.05	1
	IDP dMRI TBSS ICVF Cingulum hippocampus L	MR Egger	0.684237031	0.05	1
		Weighted median	0.996640144	0.05	1

		Inverse variance weighted	0.701741326	0.05	1
		Simple mode	0.972691219	0.05	1
		Weighted mode	0.996208274	0.05	1
	IDP dMRI TBSS ICVF Fornix cres+Stria terminalis R	Wald ratio	0.390255045	0.05	1
	IDP dMRI TBSS ICVF Superior longitudinal fasciculus R	Inverse variance weighted	0.532422766	0.05	1
	IDP dMRI TBSS ICVF Superior longitudinal fasciculus L	MR Egger	0.603233355	0.05	1
		Weighted median	0.997136348	0.05	1
		Inverse variance weighted	0.690223321	0.05	1
		Simple mode	0.980646109	0.05	1
		Weighted mode	0.991577599	0.05	1
	IDP dMRI TBSS ICVF Superior fronto-occipital fasciculus R	Inverse variance weighted	0.52009797	0.05	1
	IDP dMRI TBSS ICVF Uncinate fasciculus R	Wald ratio	0.990026703	0.05	1
	IDP dMRI TBSS ICVF Uncinate fasciculus L	Wald ratio	0.990026703	0.05	1
	IDP dMRI TBSS OD Pontine crossing tract	MR Egger	0.429056871	0.05	1
		Weighted median	0.49871437	0.05	1
		Inverse variance weighted	0.575199644	0.05	1
		Simple mode	0.555811965	0.05	1
		Weighted mode	0.551170645	0.05	1
	IDP dMRI TBSS OD Cerebral peduncle R	Wald ratio	0.027459847	0.05	1
	IDP dMRI TBSS OD Cerebral peduncle L	Wald ratio	0.027459847	0.05	1
	IDP dMRI TBSS OD Posterior limb of internal capsule L	Wald ratio	0.966296597	0.05	1

	IDP dMRI TBSS OD External capsule R	Inverse variance weighted	0.513983003	0.05	1
	IDP dMRI TBSS OD External capsule L	Wald ratio	0.708970247	0.05	1
	IDP dMRI TBSS OD Superior longitudinal fasciculus R	Wald ratio	0.012832939	0.05	1
	IDP dMRI TBSS ISOVF Fornix	Wald ratio	0.473030324	0.05	1
	IDP dMRI TBSS ISOVF External capsule R	Wald ratio	0.491256523	0.05	1
	IDP dMRI TBSS ISOVF Cingulum cingulate gyrus R	Wald ratio	0.89912991	0.05	1
	IDP dMRI ProbtrackX FA ar l	Wald ratio	0.990026703	0.05	1
	IDP dMRI ProbtrackX FA atr l	Wald ratio	0.990026703	0.05	1
	IDP dMRI ProbtrackX FA fmi	MR Egger	0.552597888	0.05	1
		Weighted median	0.56749133	0.05	1
		Inverse variance weighted	0.341452138	0.05	1
		Simple mode	0.23090637	0.05	1
		Weighted mode	0.971608501	0.05	1
	IDP dMRI ProbtrackX FA ifo l	Wald ratio	0.958570086	0.05	1
	IDP dMRI ProbtrackX FA ifo r	Wald ratio	0.979278056	0.05	1
	IDP dMRI ProbtrackX FA ml l	Wald ratio	0.701588108	0.05	1
	IDP dMRI ProbtrackX FA ptr r	Wald ratio	0.817494086	0.05	1
	IDP dMRI ProbtrackX FA slf l	Wald ratio	0.979278056	0.05	1
	IDP dMRI ProbtrackX FA str l	Wald ratio	0.000465258	0.05	1
	IDP dMRI ProbtrackX FA unc l	Wald ratio	0.990026703	0.05	1
	IDP dMRI ProbtrackX FA unc r	Wald ratio	0.990026703	0.05	1
	IDP dMRI ProbtrackX MD ar l	Wald ratio	0.990026703	0.05	1

	IDP dMRI ProbtrackX MD ar r	Wald ratio	0.990026703	0.05	1
	IDP dMRI ProbtrackX MD atr l	Wald ratio	0.990026703	0.05	1
	IDP dMRI ProbtrackX MD atr r	Wald ratio	0.990026703	0.05	1
	IDP dMRI ProbtrackX MD cgc l	MR Egger	0.974735826	0.05	1
		Weighted median	0.957625901	0.05	1
		Inverse variance weighted	0.93680347	0.05	1
		Simple mode	0.952397566	0.05	1
		Weighted mode	0.992114479	0.05	1
	IDP dMRI ProbtrackX MD cgc r	Inverse variance weighted	0.88621223	0.05	1
	IDP dMRI ProbtrackX MD fmi	Inverse variance weighted	0.984540318	0.05	1
	IDP dMRI ProbtrackX MD ifo l	Wald ratio	0.958570086	0.05	1
	IDP dMRI ProbtrackX MD ifo r	Wald ratio	0.958570086	0.05	1
	IDP dMRI ProbtrackX MD ilf l	Wald ratio	0.958570086	0.05	1
	IDP dMRI ProbtrackX MD ilf r	Wald ratio	0.958570086	0.05	1
	IDP dMRI ProbtrackX MD ptr l	Wald ratio	0.990026703	0.05	1
	IDP dMRI ProbtrackX MD ptr r	Wald ratio	0.990026703	0.05	1
	IDP dMRI ProbtrackX MD slf l	Inverse variance weighted	0.98344139	0.05	1
	IDP dMRI ProbtrackX MD slf r	Wald ratio	0.958570086	0.05	1
	IDP dMRI ProbtrackX MD str l	Wald ratio	0.641392007	0.05	1
	IDP dMRI ProbtrackX MD str r	Wald ratio	0.990026703	0.05	1
	IDP dMRI ProbtrackX MD unc l	Wald ratio	0.990026703	0.05	1
	IDP dMRI ProbtrackX MD unc r	Inverse variance weighted	0.689914452	0.05	1

	IDP dMRI ProbtrackX MO atr l	Wald ratio	0.026451898	0.05	1
	IDP dMRI ProbtrackX MO ml r	Wald ratio	0.216172927	0.05	1
	IDP T1 FAST ROIs L intracalc cortex	Wald ratio	0.253097909	0.05	1
	IDP dMRI ProbtrackX L1 ilf l	Wald ratio	0.990026703	0.05	1
	IDP T1 FAST ROIs R intracalc cortex	Wald ratio	0.253097909	0.05	1
	IDP dMRI ProbtrackX L1 slf l	Wald ratio	0.990026703	0.05	1
	IDP dMRI ProbtrackX L1 slf r	Wald ratio	0.004346535	0.05	1
	IDP dMRI ProbtrackX L1 str r	Wald ratio	0.977206399	0.05	1
	IDP dMRI ProbtrackX L1 unc l	Inverse variance weighted	0.934001889	0.05	1
	IDP dMRI ProbtrackX L1 unc r	Wald ratio	0.455378189	0.05	1
	IDP dMRI ProbtrackX L2 ar l	Wald ratio	0.990026703	0.05	1
	IDP dMRI ProbtrackX L2 ar r	Wald ratio	0.990026703	0.05	1
	IDP dMRI ProbtrackX L2 atr l	Wald ratio	0.990026703	0.05	1
	IDP dMRI ProbtrackX L2 atr r	Wald ratio	0.990026703	0.05	1
	IDP dMRI ProbtrackX L2 cgh l	Wald ratio	0.05157471	0.05	1
	IDP dMRI ProbtrackX L2 fma	Wald ratio	0.308265673	0.05	1
	IDP dMRI ProbtrackX L2 fmi	Inverse variance weighted	0.984442062	0.05	1
	IDP dMRI ProbtrackX L2 ifo l	Wald ratio	0.958570086	0.05	1
	IDP dMRI ProbtrackX L2 ifo r	Wald ratio	0.958570086	0.05	1
	IDP dMRI ProbtrackX L2 ilf l	Wald ratio	0.958570086	0.05	1
	IDP dMRI ProbtrackX L2 ilf r	Wald ratio	0.958570086	0.05	1
	IDP dMRI ProbtrackX L2 ptr l	Wald ratio	0.990026703	0.05	1
	IDP dMRI ProbtrackX L2 ptr r	Wald ratio	0.990026703	0.05	1

	IDP dMRI ProbtrackX L2 slf l	Inverse variance weighted	0.983161885	0.05	1
	IDP dMRI ProbtrackX L2 slf r	Wald ratio	0.958570086	0.05	1
	IDP dMRI ProbtrackX L2 str l	Wald ratio	0.641392007	0.05	1
	IDP dMRI ProbtrackX L2 str r	Wald ratio	0.641392007	0.05	1
	IDP dMRI ProbtrackX L2 unc l	Wald ratio	0.990026703	0.05	1
	IDP dMRI ProbtrackX L2 unc r	Inverse variance weighted	0.699621052	0.05	1
	IDP dMRI ProbtrackX L3 ar l	Wald ratio	0.990026703	0.05	1
	IDP dMRI ProbtrackX L3 ar r	Wald ratio	0.990026703	0.05	1
	IDP dMRI ProbtrackX L3 atr l	Wald ratio	0.990026703	0.05	1
	IDP dMRI ProbtrackX L3 atr r	Wald ratio	0.990026703	0.05	1
	IDP dMRI ProbtrackX L3 fmi	MR Egger	0.645886351	0.05	1
		Weighted median	0.997102629	0.05	1
		Inverse variance weighted	0.636555753	0.05	1
		Simple mode	0.978826024	0.05	1
		Weighted mode	0.990572501	0.05	1
	IDP dMRI ProbtrackX L3 ifo l	Inverse variance weighted	0.986281134	0.05	1
	IDP dMRI ProbtrackX L3 ifo r	Wald ratio	0.958570086	0.05	1
	IDP dMRI ProbtrackX L3 ilf l	Wald ratio	0.958570086	0.05	1
	IDP dMRI ProbtrackX L3 ilf r	Wald ratio	0.958570086	0.05	1
	IDP dMRI ProbtrackX L3 ptr l	Wald ratio	0.990026703	0.05	1
	IDP dMRI ProbtrackX L3 ptr r	Wald ratio	0.990026703	0.05	1
	IDP dMRI ProbtrackX L3 slf l	Inverse variance weighted	0.983972701	0.05	1

	IDP dMRI ProbtrackX L3 slf r	Wald ratio	0.958570086	0.05	1
	IDP dMRI ProbtrackX L3 str l	Wald ratio	0.990026703	0.05	1
	IDP dMRI ProbtrackX L3 unc l	Wald ratio	0.990026703	0.05	1
	IDP dMRI ProbtrackX L3 unc r	Inverse variance weighted	0.67396044	0.05	1
	IDP dMRI ProbtrackX ICVF ar l	MR Egger	0.957980709	0.05	1
		Weighted median	0.992910486	0.05	1
		Inverse variance weighted	0.954637073	0.05	1
		Simple mode	0.813933397	0.05	1
		Weighted mode	0.984428891	0.05	1
	IDP dMRI ProbtrackX ICVF ar r	MR Egger	0.986686891	0.05	1
		Weighted median	0.942885205	0.05	1
		Inverse variance weighted	0.932982584	0.05	1
		Simple mode	0.895202008	0.05	1
		Weighted mode	0.984264357	0.05	1
	IDP dMRI ProbtrackX ICVF atr l	MR Egger	0.71826293	0.05	1
		Weighted median	0.992791815	0.05	1
		Inverse variance weighted	0.672920293	0.05	1
		Simple mode	0.971708732	0.05	1
		Weighted mode	0.998386146	0.05	1
	IDP dMRI ProbtrackX ICVF atr r	MR Egger	0.725326112	0.05	1
		Weighted median	0.991463484	0.05	1
		Inverse variance weighted	0.658603011	0.05	1

		Simple mode	0.974350946	0.05	1
		Weighted mode	0.995519567	0.05	1
	IDP dMRI ProbtrackX ICVF cgc l	Wald ratio	0.185395355	0.05	1
	IDP dMRI ProbtrackX ICVF cgh l	MR Egger	0.726848833	0.05	1
		Weighted median	0.991971805	0.05	1
		Inverse variance weighted	0.66941595	0.05	1
		Simple mode	0.970703418	0.05	1
		Weighted mode	0.996816264	0.05	1
	IDP dMRI ProbtrackX ICVF cgh r	Wald ratio	0.990026703	0.05	1
	IDP dMRI ProbtrackX ICVF cst l	MR Egger	0.981616198	0.05	1
		Weighted median	0.950492675	0.05	1
		Inverse variance weighted	0.931799282	0.05	1
		Simple mode	0.884995361	0.05	1
		Weighted mode	0.988312846	0.05	1
	IDP dMRI ProbtrackX ICVF cst r	MR Egger	0.60163883	0.05	1
		Weighted median	0.793801883	0.05	1
		Inverse variance weighted	0.478592877	0.05	1
		Simple mode	0.908760427	0.05	1
		Weighted mode	0.956903696	0.05	1
	IDP dMRI ProbtrackX ICVF fma	Inverse variance weighted	0.464057543	0.05	1
	IDP dMRI ProbtrackX ICVF fmi	MR Egger	0.564002781	0.05	1
		Weighted median	0.704760575	0.05	1

		Inverse variance weighted	0.408497543	0.05	1
		Simple mode	0.937980863	0.05	1
		Weighted mode	0.965448413	0.05	1
	IDP dMRI ProbtrackX ICVF ifo l	Inverse variance weighted	0.988615519	0.05	1
	IDP dMRI ProbtrackX ICVF ifo r	Inverse variance weighted	0.988400171	0.05	1
	IDP dMRI ProbtrackX ICVF ilf l	Inverse variance weighted	0.987734225	0.05	1
	IDP dMRI ProbtrackX ICVF ilf r	Wald ratio	0.958570086	0.05	1
	IDP dMRI ProbtrackX ICVF mcp	Wald ratio	0.834968706	0.05	1
	IDP dMRI ProbtrackX ICVF ml l	Wald ratio	0.827469068	0.05	1
	IDP dMRI ProbtrackX ICVF ml r	Wald ratio	0.990026703	0.05	1
	IDP dMRI ProbtrackX ICVF ptr l	Wald ratio	0.958570086	0.05	1
	IDP dMRI ProbtrackX ICVF ptr r	Inverse variance weighted	0.824386869	0.05	1
	IDP dMRI ProbtrackX ICVF slf l	MR Egger	0.64485331	0.05	1
		Weighted median	0.999054349	0.05	1
		Inverse variance weighted	0.678428164	0.05	1
		Simple mode	0.97850487	0.05	1
		Weighted mode	0.99180577	0.05	1
	IDP dMRI ProbtrackX ICVF slf r	Inverse variance weighted	0.52332118	0.05	1
	IDP dMRI ProbtrackX ICVF str l	MR Egger	0.700050875	0.05	1
		Weighted median	0.993245502	0.05	1
		Inverse variance weighted	0.640783121	0.05	1

		Simple mode	0.976407677	0.05	1
		Weighted mode	0.9948869	0.05	1
	IDP dMRI ProbtrackX ICVF str r	MR Egger	0.664307106	0.05	1
		Weighted median	0.996708679	0.05	1
		Inverse variance weighted	0.658521556	0.05	1
		Simple mode	0.976375779	0.05	1
		Weighted mode	0.993098585	0.05	1
	IDP dMRI ProbtrackX ICVF unc l	Inverse variance weighted	0.647641647	0.05	1
	IDP dMRI ProbtrackX ICVF unc r	Inverse variance weighted	0.992086355	0.05	1
	IDP dMRI ProbtrackX OD atr r	Wald ratio	0.592156029	0.05	1
	IDP dMRI ProbtrackX OD ilf l	Wald ratio	0.725324775	0.05	1
	IDP dMRI ProbtrackX OD slf l	Wald ratio	0.147276503	0.05	1
	IDP dMRI ProbtrackX OD str l	Inverse variance weighted	0.110705202	0.05	1
	IDP dMRI ProbtrackX ISOVF atr r	Wald ratio	0.990026703	0.05	1
	IDP dMRI ProbtrackX ISOVF cgc l	Wald ratio	0.175333729	0.05	1
	IDP dMRI ProbtrackX ISOVF ifo r	Wald ratio	0.311066364	0.05	1
	IDP dMRI ProbtrackX ISOVF ilf l	Wald ratio	0.920870108	0.05	1
	IDP dMRI ProbtrackX ISOVF ilf r	MR Egger	0.778574649	0.05	1
		Weighted median	0.188796762	0.05	1
		Inverse variance weighted	0.120028384	0.05	1
		Simple mode	0.333780989	0.05	1
		Weighted mode	0.336292473	0.05	1

	IDP dMRI ProbtrackX ISOVF slf l	Inverse variance weighted	0.637273343	0.05	1
	IDP dMRI ProbtrackX ISOVF slf r	MR Egger	0.344780551	0.05	1
		Weighted median	0.58619229	0.05	1
		Inverse variance weighted	0.447014114	0.05	1
		Simple mode	0.879020683	0.05	1
		Weighted mode	0.868007186	0.05	1
	IDP dMRI ProbtrackX ISOVF unc l	Wald ratio	0.51705783	0.05	1
	IDP dMRI ProbtrackX ISOVF unc r	Wald ratio	0.455378189	0.05	1
	IDP T1 SIENAX brain-normalised volume	Wald ratio	0.368658041	0.05	1
Drug-induced systemic lupus erythematosus	IDP T1 SIENAX peripheral grey normalised volume	Wald ratio	0.091178447	0.05	0.010993962
	IDP T1 FAST ROIs R temp occ fusif cortex	Wald ratio	0.421126008	0.05	1
	IDP T1 FAST ROIs R occ pole	Inverse variance weighted	0.516549647	0.05	0.999986968
	IDP T1 FAST ROIs L thalamus	Inverse variance weighted	0.92713754	0.05	1
	IDP T1 FAST ROIs R thalamus	Wald ratio	0.970372697	0.05	1
	IDP T1 FAST ROIs L putamen	Wald ratio	0.878021619	0.05	1
	IDP T1 FAST ROIs R putamen	Wald ratio	0.878021619	0.05	1
	IDP T1 FAST ROIs L pallidum	Wald ratio	0.114187243	0.05	1
	IDP T1 FIRST left caudate volume	Wald ratio	0.371006889	0.05	1
	IDP T1 FAST ROIs L hippocampus	Inverse variance weighted	0.313316964	0.05	1
	IDP T1 FAST ROIs R hippocampus	Wald ratio	0.060785609	0.05	1
	IDP T1 FAST ROIs L ventral striatum	Wald ratio	0.878021619	0.05	1
	IDP T1 FAST ROIs R ventral striatum	Inverse variance weighted	0.914044026	0.05	1

	IDP T1 FAST ROIs L cerebellum VI	Wald ratio	0.686540004	0.05	1
	IDP T1 FAST ROIs L cerebellum crus I	MR Egger	0.564272576	0.05	1
		Weighted median	0.958829097	0.05	1
		Inverse variance weighted	0.807652768	0.05	1
		Simple mode	0.893372593	0.05	1
		Weighted mode	0.829645099	0.05	1
	IDP T1 FAST ROIs R cerebellum crus I	Inverse variance weighted	0.756862064	0.05	1
	IDP T1 FAST ROIs L cerebellum crus II	MR Egger	0.821487305	0.05	1
		Weighted median	0.969763871	0.05	1
		Inverse variance weighted	0.745224179	0.05	1
		Simple mode	0.953199043	0.05	1
		Weighted mode	0.934248259	0.05	1
	IDP T1 FAST ROIs V cerebellum crus II	Inverse variance weighted	0.846677014	0.05	1
	IDP T1 FAST ROIs R cerebellum crus II	Inverse variance weighted	0.686341619	0.05	1
	IDP T1 FIRST left putamen volume	Inverse variance weighted	0.694399739	0.05	1
	IDP T1 FAST ROIs R cerebellum VIIb	Wald ratio	0.435793172	0.05	1
	IDP T1 FAST ROIs V cerebellum VIIIa	MR Egger	0.624973663	0.05	1
		Weighted median	0.202712256	0.05	0.851404664
		Inverse variance weighted	0.219923137	0.05	0.97054626
		Simple mode	0.590353862	0.05	0.996791534
		Weighted mode	0.290428806	0.05	0.656373543
	IDP T1 FAST ROIs L cerebellum VIIIb	Wald ratio	0.971317503	0.05	1

	IDP T1 FAST ROIs V cerebellum VIIIb	Inverse variance weighted	0.197772669	0.05	0.62286694
	IDP T1 FAST ROIs R cerebellum VIIIb	Wald ratio	0.878021619	0.05	1
	IDP T1 FAST ROIs L cerebellum IX	Inverse variance weighted	0.809500175	0.05	1
	IDP T1 FIRST right putamen volume	Wald ratio	0.575442618	0.05	0.999998854
	IDP T1 FAST ROIs V cerebellum IX	Inverse variance weighted	0.950674976	0.05	1
	IDP T1 FAST ROIs R cerebellum IX	Inverse variance weighted	0.866339635	0.05	1
	IDP T1 FAST ROIs V cerebellum X	Wald ratio	0.878021619	0.05	1
	IDP T2 FLAIR BIANCA WMH volume	Inverse variance weighted	0.824227135	0.05	1
	IDP SWI T2star left caudate	MR Egger	0.33560698	0.05	0.003473224
		Weighted median	0.873721954	0.05	1
		Inverse variance weighted	0.512563169	0.05	1
		Simple mode	0.785683353	0.05	1
		Weighted mode	0.85334805	0.05	1
	IDP SWI T2star right caudate	MR Egger	0.442098103	0.05	0.259025829
		Weighted median	0.201171909	0.05	1
		Inverse variance weighted	0.061454578	0.05	1
		Simple mode	0.115892024	0.05	1
		Weighted mode	0.756171439	0.05	1
	IDP T1 FIRST left pallidum volume	Wald ratio	0.141742898	0.05	0.556962221
	IDP SWI T2star left putamen	MR Egger	0.856816226	0.05	1
		Weighted median	0.805236341	0.05	1

		Inverse variance weighted	0.535784463	0.05	1
		Simple mode	0.246739547	0.05	1
		Weighted mode	0.957336154	0.05	1
	IDP SWI T2star right putamen	MR Egger	0.697289731	0.05	0.954450631
		Weighted median	0.908992783	0.05	1
		Inverse variance weighted	0.623318407	0.05	1
		Simple mode	0.98264873	0.05	1
		Weighted mode	0.954558614	0.05	1
	IDP SWI T2star left pallidum	MR Egger	0.99045752	0.05	1
		Weighted median	0.859208328	0.05	1
		Inverse variance weighted	0.904327472	0.05	1
		Simple mode	0.877638374	0.05	1
		Weighted mode	0.922739615	0.05	1
	IDP SWI T2star right pallidum	MR Egger	0.765653402	0.05	1
		Weighted median	0.9785363	0.05	1
		Inverse variance weighted	0.928219346	0.05	1
		Simple mode	0.775854283	0.05	1
		Weighted mode	0.860207364	0.05	1
	IDP T1 FIRST right pallidum volume	Wald ratio	0.141742898	0.05	0.238096004
	IDP dMRI TBSS FA Genu of corpus callosum	Wald ratio	0.056308689	0.05	0.115770955
	IDP dMRI TBSS FA Splenium of corpus callosum	Inverse variance weighted	0.702565416	0.05	1
	IDP dMRI TBSS FA Corticospinal tract L	Wald ratio	0.225795078	0.05	1

	IDP dMRI TBSS FA Superior cerebellar peduncle R	Inverse variance weighted	0.395681737	0.05	1
	IDP dMRI TBSS FA Superior cerebellar peduncle L	Inverse variance weighted	0.431273648	0.05	1
	IDP dMRI TBSS FA Posterior limb of internal capsule R	Wald ratio	0.884744046	0.05	1
	IDP dMRI TBSS FA Retrolenticular part of internal capsule R	Inverse variance weighted	0.475363603	0.05	0.999989261
	IDP dMRI TBSS FA Anterior corona radiata R	Wald ratio	0.056308689	0.05	0.092141107
	IDP dMRI TBSS FA Anterior corona radiata L	Wald ratio	0.056308689	0.05	0.081421351
	IDP dMRI TBSS FA Posterior thalamic radiation R	Wald ratio	0.698374054	0.05	1
	IDP dMRI TBSS FA Posterior thalamic radiation L	Wald ratio	0.699336805	0.05	1
	IDP dMRI TBSS FA Sagittal stratum R	Wald ratio	0.132195263	0.05	0.14340653
	IDP dMRI TBSS FA Cingulum cingulate gyrus R	Inverse variance weighted	0.258798988	0.05	0.963846263
	IDP dMRI TBSS FA Superior longitudinal fasciculus L	Inverse variance weighted	0.027012902	0.05	0.202251897
	IDP dMRI TBSS FA Uncinate fasciculus L	Wald ratio	0.107019904	0.05	0.292409836
	IDP dMRI TBSS MD Genu of corpus callosum	Wald ratio	0.056308689	0.05	1
	IDP dMRI TBSS MD Body of corpus callosum	Wald ratio	0.542869215	0.05	1
	IDP dMRI TBSS MD Splenium of corpus callosum	Inverse variance weighted	0.922983957	0.05	1
	IDP dMRI TBSS MD Corticospinal tract R	Wald ratio	0.48608947	0.05	0.999970786
	IDP dMRI TBSS MD Superior cerebellar peduncle R	Wald ratio	0.815340312	0.05	1
	IDP dMRI TBSS MD Superior cerebellar peduncle L	Wald ratio	0.939906866	0.05	1
	IDP dMRI TBSS MD Anterior limb of internal capsule L	Wald ratio	0.672550201	0.05	1

	IDP dMRI TBSS MD Retrolenticular part of internal capsule R	Wald ratio	0.6792227	0.05	1
	IDP T1 FIRST left caudate volume plus IDP T1 FIRST right caudate volume	Wald ratio	0.135092475	0.05	1
	IDP T1 FIRST left putamen volume plus IDP T1 FIRST right putamen volume	Inverse variance weighted	0.621115369	0.05	1
	IDP T1 FIRST left pallidum volume plus IDP T1 FIRST right pallidum volume	Wald ratio	0.141742898	0.05	0.999990356
	IDP SWI T2star left thalamus plus IDP SWI T2star right thalamus	MR Egger	0.451253792	0.05	1
		Weighted median	0.182047197	0.05	1
		Inverse variance weighted	0.093716835	0.05	1
		Simple mode	0.326350699	0.05	1
		Weighted mode	0.464647438	0.05	1
	IDP dMRI TBSS MD Retrolenticular part of internal capsule L	Wald ratio	0.6792227	0.05	1
	IDP SWI T2star left caudate plus IDP SWI T2star right caudate	MR Egger	0.74830406	0.05	1
		Weighted median	0.808160712	0.05	1
		Inverse variance weighted	0.315255426	0.05	1
		Simple mode	0.738644449	0.05	1
		Weighted mode	0.746551709	0.05	1
	IDP SWI T2star left putamen plus IDP SWI T2star right putamen	MR Egger	0.927078784	0.05	1
		Weighted median	0.655252483	0.05	1
		Inverse variance weighted	0.556629537	0.05	1
		Simple mode	0.283212539	0.05	1
		Weighted mode	0.398548428	0.05	1
		MR Egger	0.907838229	0.05	1

	IDP SWI T2star left pallidum plus IDP SWI T2star right pallidum	Weighted median	0.996118788	0.05	1
		Inverse variance weighted	0.794651141	0.05	1
		Simple mode	0.884267542	0.05	1
		Weighted mode	0.871671453	0.05	1
	volume Left-Lateral-Ventricle	MR Egger	0.551283585	0.05	0.002758797
		Weighted median	0.771513337	0.05	1
		Inverse variance weighted	0.810748489	0.05	1
		Simple mode	0.524308883	0.05	0.97898856
		Weighted mode	0.595769058	0.05	0.99996048
	volume Left-Cerebellum-White-Matter	Inverse variance weighted	0.92841076	0.05	1
	volume Left-Cerebellum-Cortex	Inverse variance weighted	0.508656793	0.05	1
	IDP dMRI TBSS MD Anterior corona radiata R	Inverse variance weighted	0.393609737	0.05	1
	volume Left-Putamen	Inverse variance weighted	0.821556958	0.05	1
	volume 4th-Ventricle	Inverse variance weighted	0.686856655	0.05	1
	volume Brain-Stem	Wald ratio	0.014379581	0.05	1
	volume Left-Hippocampus	Inverse variance weighted	0.402392822	0.05	1
	volume CSF	MR Egger	0.613564225	0.05	1
		Weighted median	0.364742408	0.05	1
		Inverse variance weighted	0.54661278	0.05	1
		Simple mode	0.485575824	0.05	1
		Weighted mode	0.492318443	0.05	1

	IDP dMRI TBSS MD Anterior corona radiata L	Inverse variance weighted	0.350931219	0.05	1
	volume Left-Accumbens-area	Wald ratio	0.878021619	0.05	1
	volume Right-Lateral-Ventricle	Inverse variance weighted	0.8320317	0.05	1
	volume Right-Inf-Lat-Vent	Wald ratio	0.938813916	0.05	1
	volume Right-Cerebellum-White-Matter	Wald ratio	0.026278581	0.05	1
	volume Right-Cerebellum-Cortex	MR Egger	0.995364468	0.05	1
		Weighted median	0.732758782	0.05	1
		Inverse variance weighted	0.505373461	0.05	1
		Simple mode	0.948078093	0.05	1
		Weighted mode	0.86847906	0.05	1
	IDP dMRI TBSS MD Superior corona radiata R	Inverse variance weighted	0.392767404	0.05	1
	volume Right-Putamen	Wald ratio	0.575442618	0.05	0.999999986
	volume Right-Hippocampus	Wald ratio	0.060785609	0.05	1
	volume Right-Amygdala	Wald ratio	0.006046373	0.05	0.002753169
	volume Right-VentralDC	Wald ratio	0.545822893	0.05	1
	volume CC Posterior	Wald ratio	0.885931607	0.05	1
	IDP dMRI TBSS MD Superior corona radiata L	Inverse variance weighted	0.388241316	0.05	1
	IDP dMRI TBSS MD Posterior corona radiata R	Wald ratio	0.6792227	0.05	1
	volume BrainSegVol-to-eTIV	Wald ratio	0.610734511	0.05	1
	IDP dMRI TBSS MD Posterior corona radiata L	Wald ratio	0.699336805	0.05	1
	volume rhSurfaceHoles	Wald ratio	0.472691317	0.05	0.997298393
	DKTatlas lh cuneus area	Wald ratio	0.057986268	0.05	1

	IDP dMRI TBSS MD Posterior thalamic radiation R	Wald ratio	0.699336805	0.05	1
	DKTatlas lh lateraloccipital area	Wald ratio	0.057986268	0.05	1
	DKTatlas lh lingual area	Wald ratio	0.049417175	0.05	1
	IDP dMRI TBSS MD Posterior thalamic radiation L	Wald ratio	0.6792227	0.05	1
	DKTatlas lh parstriangularis area	Wald ratio	0.609018203	0.05	1
	DKTatlas lh pericalcarine area	Inverse variance weighted	0.041871549	0.05	1
	DKTatlas lh postcentral area	Inverse variance weighted	0.601071643	0.05	1
	DKTatlas lh posteriorcingulate area	Wald ratio	0.502015321	0.05	0.662608889
	DKTatlas lh precentral area	Wald ratio	0.63207003	0.05	1
	DKTatlas lh precuneus area	Wald ratio	0.5933198	0.05	1
	IDP dMRI TBSS MD Sagittal stratum R	Wald ratio	0.6792227	0.05	1
	DKTatlas lh superiorparietal area	Wald ratio	0.568121036	0.05	1
	DKTatlas lh superiortemporal area	Wald ratio	0.643381897	0.05	0.360231209
	DKTatlas lh supramarginal area	Wald ratio	0.287297929	0.05	1
	DKTatlas lh WhiteSurfArea area	Wald ratio	0.799850123	0.05	1
	IDP dMRI TBSS MD Sagittal stratum L	Wald ratio	0.6792227	0.05	1
	a2009s lh G&S subcentral area	Wald ratio	0.63207003	0.05	1
	a2009s lh G cuneus area	Wald ratio	0.06199987	0.05	1
	a2009s lh G front inf-Opercular area	Wald ratio	0.25113312	0.05	1
	IDP dMRI TBSS MD External capsule R	Wald ratio	0.056308689	0.05	1
	IDP dMRI TBSS MD External capsule L	Inverse variance weighted	0.077707672	0.05	1
	a2009s lh G pariet inf-Supramar area	Wald ratio	0.287297929	0.05	1

	a2009s lh G parietal sup area	Wald ratio	0.568121036	0.05	1
	a2009s lh G postcentral area	Wald ratio	0.564500137	0.05	0.999997512
	a2009s lh G precentral area	Wald ratio	0.63207003	0.05	1
	a2009s lh G precuneus area	Wald ratio	0.06199987	0.05	0.102244848
	IDP dMRI TBSS MD Cingulum cingulate gyrus R	MR Egger	0.344300904	0.05	1
		Weighted median	0.169730516	0.05	1
		Inverse variance weighted	0.167838839	0.05	1
		Simple mode	0.567561426	0.05	1
		Weighted mode	0.220839822	0.05	1
	IDP dMRI TBSS MD Cingulum cingulate gyrus L	MR Egger	0.343796711	0.05	1
		Weighted median	0.164712272	0.05	1
		Inverse variance weighted	0.166484893	0.05	1
		Simple mode	0.618465736	0.05	1
		Weighted mode	0.224927504	0.05	1
	a2009s lh S calcarine area	Wald ratio	0.049417175	0.05	1
	a2009s lh S central area	Wald ratio	0.63396658	0.05	1
	a2009s lh S collat transv ant area	Wald ratio	0.811581486	0.05	1
	a2009s lh S front middle area	Wald ratio	0.392208887	0.05	0.013710697
	IDP dMRI TBSS MD Cingulum hippocampus R	Wald ratio	0.6792227	0.05	1
	a2009s lh S intrapariet&P trans area	Wald ratio	0.568121036	0.05	1
	IDP dMRI TBSS MD Cingulum hippocampus L	Wald ratio	0.6792227	0.05	1
	a2009s lh S subparietal area	Wald ratio	0.049417175	0.05	0.063459033

	DKTatlas rh cuneus area	Wald ratio	0.049417175	0.05	1
	DKTatlas rh lingual area	Wald ratio	0.049241175	0.05	1
	DKTatlas rh parstriangularis area	Wald ratio	0.365720045	0.05	1
	DKTatlas rh pericalcarine area	Inverse variance weighted	0.193146138	0.05	1
	IDP dMRI TBSS MD Superior longitudinal fasciculus R	Wald ratio	0.6792227	0.05	1
	DKTatlas rh postcentral area	Wald ratio	0.63396658	0.05	0.999999239
	DKTatlas rh precentral area	Wald ratio	0.63207003	0.05	1
	IDP dMRI TBSS MD Superior longitudinal fasciculus L	Wald ratio	0.6792227	0.05	1
	a2009s rh G&S subcentral area	Wald ratio	0.63396658	0.05	1
	a2009s rh G&S cingul-Mid-Post area	Wald ratio	0.405074008	0.05	1
	a2009s rh G cuneus area	Wald ratio	0.049417175	0.05	1
	IDP dMRI TBSS MD Superior fronto-occipital fasciculus L	Wald ratio	0.548037004	0.05	0.999972092
	a2009s rh G oc-temp med-Lingual area	Wald ratio	0.049241175	0.05	1
	a2009s rh G parietal sup area	Wald ratio	0.806245671	0.05	1
	IDP dMRI TBSS MD Uncinate fasciculus R	Wald ratio	0.674865904	0.05	1
	a2009s rh G precentral area	Wald ratio	0.63207003	0.05	1
	a2009s rh G precuneus area	Wald ratio	0.893192448	0.05	1
	IDP dMRI TBSS MD Uncinate fasciculus L	Inverse variance weighted	0.065137536	0.05	1
	a2009s rh Pole occipital area	Wald ratio	0.049417175	0.05	1
	a2009s rh S calcarine area	Wald ratio	0.049241175	0.05	1
	a2009s rh S central area	Wald ratio	0.63207003	0.05	1
	a2009s rh S collat transv ant area	Wald ratio	0.811581486	0.05	1

	a2009s rh S orbital med-olfact area	Wald ratio	0.52933351	0.05	0.999590167
	IDP dMRI TBSS MO Pontine crossing tract	MR Egger	0.423407894	0.05	0.039479643
		Weighted median	0.64555383	0.05	1
		Inverse variance weighted	0.431228148	0.05	1
		Simple mode	0.636394259	0.05	1
		Weighted mode	0.845833619	0.05	1
	DKTatlas lh postcentral thickness	Wald ratio	0.63396658	0.05	1
	a2009s lh G insular short thickness	Wald ratio	0.390897311	0.05	1
	a2009s lh G postcentral thickness	Wald ratio	0.63396658	0.05	1
	IDP T1 SIENAX CSF normalised volume	Inverse variance weighted	0.386773786	0.05	0.99881848
	IDP dMRI TBSS MO Medial lemniscus R	Wald ratio	0.162383066	0.05	1
	a2009s lh S postcentral thickness	Wald ratio	0.613782129	0.05	1
	DKTatlas rh lateraloccipital thickness	Wald ratio	0.878021619	0.05	1
	DKTatlas rh parstriangularis thickness	Wald ratio	0.89987679	0.05	1
	DKTatlas rh postcentral thickness	Wald ratio	0.63396658	0.05	1
	DKTatlas rh posteriorcingulate thickness	Inverse variance weighted	0.009701928	0.05	1
	a2009s rh G&S cingul-Mid-Ant thickness	Wald ratio	0.903230132	0.05	1
	a2009s rh G cuneus thickness	Wald ratio	0.878021619	0.05	1
	a2009s rh G postcentral thickness	Wald ratio	0.63396658	0.05	1
	a2009s rh Pole occipital thickness	Wald ratio	0.878021619	0.05	1
	a2009s rh S oc sup&transversal thickness	Wald ratio	0.693427452	0.05	1
	a2009s rh S parieto occipital thickness	Wald ratio	0.337157786	0.05	1

	IDP dMRI TBSS MO Anterior corona radiata R	Wald ratio	0.535644051	0.05	0.999999208
	IDP dMRI TBSS MO Superior corona radiata R	Wald ratio	0.566782195	0.05	1
	IDP dMRI TBSS MO Cingulum cingulate gyrus L	Inverse variance weighted	0.890957595	0.05	1
	IDP dMRI TBSS L1 Anterior limb of internal capsule L	Wald ratio	0.672550201	0.05	1
	IDP dMRI TBSS L1 Posterior limb of internal capsule R	Wald ratio	0.688828831	0.05	1
	IDP dMRI TBSS L1 Posterior limb of internal capsule L	Wald ratio	0.712421821	0.05	1
	IDP dMRI TBSS L1 Anterior corona radiata R	Wald ratio	0.056308689	0.05	1
	IDP dMRI TBSS L1 Anterior corona radiata L	Wald ratio	0.056308689	0.05	1
	IDP dMRI TBSS L1 Posterior corona radiata R	Wald ratio	0.22391353	0.05	1
	IDP dMRI TBSS L1 Posterior corona radiata L	Wald ratio	0.056308689	0.05	1
	IDP dMRI TBSS L1 Sagittal stratum L	Wald ratio	0.056308689	0.05	1
	IDP dMRI TBSS L1 External capsule R	Wald ratio	0.056308689	0.05	1
	IDP dMRI TBSS L1 External capsule L	Inverse variance weighted	0.106954368	0.05	1
	IDP dMRI TBSS L1 Fornix cres+Stria terminalis L	Wald ratio	0.489911021	0.05	0.999661714
	IDP T1 FAST ROIs L precentral gyrus	Wald ratio	0.63396658	0.05	1
	IDP dMRI TBSS L1 Superior longitudinal fasciculus R	Inverse variance weighted	0.732436742	0.05	1
	IDP dMRI TBSS L1 Uncinate fasciculus L	Wald ratio	0.072611407	0.05	1
	IDP dMRI TBSS L2 Pontine crossing tract	Wald ratio	0.162383066	0.05	0.117919856
	IDP dMRI TBSS L2 Genu of corpus callosum	MR Egger	0.382477969	0.05	1
		Weighted median	0.116572591	0.05	1

		Inverse variance weighted	0.184885407	0.05	1
		Simple mode	0.343699043	0.05	1
		Weighted mode	0.266468888	0.05	1
	IDP dMRI TBSS L2 Body of corpus callosum	Wald ratio	0.056308689	0.05	1
	IDP dMRI TBSS L2 Splenium of corpus callosum	MR Egger	0.348675354	0.05	0.002542772
		Weighted median	0.544141588	0.05	1
		Inverse variance weighted	0.786556625	0.05	1
		Simple mode	0.667085094	0.05	1
		Weighted mode	0.645665999	0.05	1
	IDP dMRI TBSS L2 Corticospinal tract R	Wald ratio	0.48608947	0.05	0.999956357
	IDP T1 SIENAX CSF unnormalised volume	MR Egger	0.503808203	0.05	0.002610393
		Weighted median	0.745222363	0.05	1
		Inverse variance weighted	0.786787399	0.05	1
		Simple mode	0.513246962	0.05	0.948424555
		Weighted mode	0.565354332	0.05	0.999744433
	IDP T1 FAST ROIs L temporal pole	Wald ratio	0.092824599	0.05	0.016711799
	IDP dMRI TBSS L2 Superior cerebellar peduncle R	Inverse variance weighted	0.407404613	0.05	1
	IDP dMRI TBSS L2 Superior cerebellar peduncle L	Wald ratio	0.939906866	0.05	1
	IDP dMRI TBSS L2 Posterior limb of internal capsule R	Inverse variance weighted	0.785738062	0.05	1
	IDP dMRI TBSS L2 Posterior limb of internal capsule L	Wald ratio	0.601111784	0.05	1
	IDP dMRI TBSS L2 Retrolenticular part of internal capsule R	Inverse variance weighted	0.494187606	0.05	1

	IDP dMRI TBSS L2 Anterior corona radiata R	Inverse variance weighted	0.386978507	0.05	1
	IDP dMRI TBSS L2 Anterior corona radiata L	Wald ratio	0.056308689	0.05	1
	IDP dMRI TBSS L2 Superior corona radiata R	Inverse variance weighted	0.576899243	0.05	1
	IDP dMRI TBSS L2 Superior corona radiata L	Wald ratio	0.6792227	0.05	1
	IDP dMRI TBSS L2 Posterior corona radiata R	Wald ratio	0.699336805	0.05	1
	IDP dMRI TBSS L2 Posterior corona radiata L	Wald ratio	0.699336805	0.05	1
	IDP dMRI TBSS L2 Posterior thalamic radiation R	Wald ratio	0.698374054	0.05	1
	IDP dMRI TBSS L2 Posterior thalamic radiation L	Wald ratio	0.699336805	0.05	1
	IDP dMRI TBSS L2 Sagittal stratum R	Wald ratio	0.6792227	0.05	1
	IDP dMRI TBSS L2 Cingulum cingulate gyrus R	Wald ratio	0.056308689	0.05	1
	IDP dMRI TBSS L2 Cingulum hippocampus R	Wald ratio	0.6792227	0.05	1
	IDP dMRI TBSS L2 Cingulum hippocampus L	Wald ratio	0.056308689	0.05	1
	IDP dMRI TBSS L2 Uncinate fasciculus L	Wald ratio	0.056308689	0.05	1
	IDP dMRI TBSS L3 Genu of corpus callosum	Wald ratio	0.056308689	0.05	1
	IDP dMRI TBSS L3 Body of corpus callosum	Wald ratio	0.056308689	0.05	1
	IDP dMRI TBSS L3 Splenium of corpus callosum	Wald ratio	0.60295302	0.05	1
	IDP dMRI TBSS L3 Inferior cerebellar peduncle R	Wald ratio	0.056308689	0.05	1
	IDP dMRI TBSS L3 Inferior cerebellar peduncle L	Wald ratio	0.056308689	0.05	1
	IDP dMRI TBSS L3 Superior cerebellar peduncle R	Inverse variance weighted	0.422797058	0.05	1

	IDP dMRI TBSS L3 Superior cerebellar peduncle L	Wald ratio	0.939906866	0.05	1
	IDP dMRI TBSS L3 Cerebral peduncle R	Wald ratio	0.206932032	0.05	0.223847517
	IDP dMRI TBSS L3 Anterior limb of internal capsule R	Wald ratio	0.461908669	0.05	0.897201894
	IDP dMRI TBSS L3 Anterior limb of internal capsule L	Wald ratio	0.824674177	0.05	1
	IDP dMRI TBSS L3 Retrolenticular part of internal capsule R	Wald ratio	0.6792227	0.05	1
	IDP dMRI TBSS L3 Anterior corona radiata R	Inverse variance weighted	0.38905876	0.05	1
	IDP dMRI TBSS L3 Anterior corona radiata L	Inverse variance weighted	0.340990066	0.05	1
	IDP dMRI TBSS L3 Posterior corona radiata R	Inverse variance weighted	0.005273313	0.05	1
	IDP dMRI TBSS L3 Posterior thalamic radiation R	Wald ratio	0.698374054	0.05	1
	IDP dMRI TBSS L3 Posterior thalamic radiation L	Wald ratio	0.6792227	0.05	1
	IDP dMRI TBSS L3 Sagittal stratum R	Wald ratio	0.6792227	0.05	1
	IDP dMRI TBSS L3 Sagittal stratum L	Wald ratio	0.6792227	0.05	1
	IDP dMRI TBSS L3 External capsule R	Wald ratio	0.056308689	0.05	1
	IDP dMRI TBSS L3 External capsule L	Wald ratio	0.056308689	0.05	1
	IDP dMRI TBSS L3 Cingulum cingulate gyrus R	Inverse variance weighted	0.356331901	0.05	1
	IDP dMRI TBSS L3 Cingulum cingulate gyrus L	Wald ratio	0.056308689	0.05	1
	IDP dMRI TBSS L3 Cingulum hippocampus R	Wald ratio	0.056308689	0.05	1
	IDP dMRI TBSS L3 Cingulum hippocampus L	Wald ratio	0.056308689	0.05	1
	IDP dMRI TBSS L3 Fornix cres+Stria terminalis R	Wald ratio	0.193992111	0.05	0.175037537

	IDP dMRI TBSS L3 Superior longitudinal fasciculus R	Wald ratio	0.6792227	0.05	1
	IDP dMRI TBSS L3 Superior longitudinal fasciculus L	Wald ratio	0.6792227	0.05	1
	IDP dMRI TBSS L3 Uncinate fasciculus R	Wald ratio	0.056308689	0.05	1
	IDP dMRI TBSS L3 Uncinate fasciculus L	Inverse variance weighted	0.012576102	0.05	1
	IDP dMRI TBSS ICVF Middle cerebellar peduncle	Wald ratio	0.6792227	0.05	1
	IDP dMRI TBSS ICVF Genu of corpus callosum	MR Egger	0.216353616	0.05	0.005539544
		Weighted median	0.3217751	0.05	1
		Inverse variance weighted	0.835222654	0.05	1
		Simple mode	0.715762218	0.05	1
		Weighted mode	0.500611812	0.05	1
	IDP dMRI TBSS ICVF Body of corpus callosum	MR Egger	0.959541332	0.05	1
		Weighted median	0.837152228	0.05	1
		Inverse variance weighted	0.939782808	0.05	1
		Simple mode	0.68462742	0.05	1
		Weighted mode	0.657708609	0.05	1
	IDP dMRI TBSS ICVF Splenium of corpus callosum	MR Egger	0.801781763	0.05	0.999999998
		Weighted median	0.960325309	0.05	1
		Inverse variance weighted	0.924623369	0.05	1
		Simple mode	0.579253543	0.05	1
		Weighted mode	0.735965182	0.05	1
	IDP dMRI TBSS ICVF Fornix	Wald ratio	0.548037004	0.05	1

	IDP dMRI TBSS ICVF Medial lemniscus R	Inverse variance weighted	0.635324688	0.05	1
	IDP dMRI TBSS ICVF Medial lemniscus L	Wald ratio	0.497552224	0.05	0.999985731
	IDP dMRI TBSS ICVF Inferior cerebellar peduncle R	Inverse variance weighted	0.856265651	0.05	1
	IDP dMRI TBSS ICVF Inferior cerebellar peduncle L	Inverse variance weighted	0.862200733	0.05	1
	IDP dMRI TBSS ICVF Superior cerebellar peduncle R	MR Egger	0.867330489	0.05	1
		Weighted median	0.099200393	0.05	0.902412799
		Inverse variance weighted	0.08664491	0.05	0.99733471
		Simple mode	0.25547096	0.05	0.58648206
		Weighted mode	0.237526027	0.05	0.451394721
	IDP dMRI TBSS ICVF Superior cerebellar peduncle L	Inverse variance weighted	0.394565352	0.05	0.999999099
	IDP dMRI TBSS ICVF Cerebral peduncle R	MR Egger	0.530932346	0.05	0.031573288
		Weighted median	0.400203348	0.05	1
		Inverse variance weighted	0.443013581	0.05	1
		Simple mode	0.961124035	0.05	1
		Weighted mode	0.422254867	0.05	0.999991178
	IDP dMRI TBSS ICVF Cerebral peduncle L	Wald ratio	0.971317503	0.05	1
	IDP dMRI TBSS ICVF Anterior limb of internal capsule R	Wald ratio	0.056308689	0.05	0.642419553
	IDP dMRI TBSS ICVF Anterior limb of internal capsule L	MR Egger	0.109005996	0.05	0.002543055
		Weighted median	0.308898618	0.05	0.999999973
		Inverse variance weighted	0.504093348	0.05	1
		Simple mode	0.726680296	0.05	1

		Weighted mode	0.180511548	0.05	0.970795497
	IDP dMRI TBSS ICVF Posterior limb of internal capsule R	MR Egger	0.634974505	0.05	1
		Weighted median	0.578060775	0.05	1
		Inverse variance weighted	0.962804121	0.05	1
		Simple mode	0.650494841	0.05	1
		Weighted mode	0.641077078	0.05	1
	IDP dMRI TBSS ICVF Posterior limb of internal capsule L	MR Egger	0.407379887	0.05	0.019613213
		Weighted median	0.06049782	0.05	0.952363246
		Inverse variance weighted	0.100635372	0.05	0.999877149
		Simple mode	0.312735691	0.05	0.947011782
		Weighted mode	0.212748553	0.05	0.866715043
	IDP dMRI TBSS ICVF Retrolenticular part of internal capsule R	MR Egger	0.491107318	0.05	0.999793557
		Weighted median	0.026787944	0.05	0.989726058
		Inverse variance weighted	0.032529533	0.05	0.999910614
		Simple mode	0.285125662	0.05	0.998876362
		Weighted mode	0.11797712	0.05	0.987187795
	IDP dMRI TBSS ICVF Retrolenticular part of internal capsule L	MR Egger	0.480916311	0.05	0.479193996
		Weighted median	0.037919065	0.05	0.990887718
		Inverse variance weighted	0.096265504	0.05	0.999999292
		Simple mode	0.300286032	0.05	0.990936689
		Weighted mode	0.184034395	0.05	0.99043534
		MR Egger	0.497532403	0.05	0.13475286

	IDP dMRI TBSS ICVF Anterior corona radiata R	Weighted median	0.047226431	0.05	0.977059726
		Inverse variance weighted	0.106622367	0.05	0.999996021
		Simple mode	0.296796454	0.05	0.974500033
		Weighted mode	0.21868579	0.05	0.950050189
	IDP dMRI TBSS ICVF Anterior corona radiata L	MR Egger	0.451381144	0.05	0.139526335
		Weighted median	0.039962801	0.05	0.971662179
		Inverse variance weighted	0.096831645	0.05	0.999984012
		Simple mode	0.286739098	0.05	0.973838447
		Weighted mode	0.203922355	0.05	0.954495918
	IDP dMRI TBSS ICVF Superior corona radiata R	MR Egger	0.55139851	0.05	0.147926774
		Weighted median	0.047947689	0.05	0.962534185
		Inverse variance weighted	0.120280112	0.05	0.999995965
		Simple mode	0.286354095	0.05	0.95635982
		Weighted mode	0.210922889	0.05	0.914900406
	IDP dMRI TBSS ICVF Superior corona radiata L	MR Egger	0.517130136	0.05	0.11690294
		Weighted median	0.048322369	0.05	0.956424121
		Inverse variance weighted	0.10970176	0.05	0.999989141
		Simple mode	0.31064902	0.05	0.950111868
		Weighted mode	0.201921697	0.05	0.911234022
	IDP dMRI TBSS ICVF Posterior corona radiata R	MR Egger	0.309768551	0.05	0.003905302
		Weighted median	0.623407582	0.05	1
		Inverse variance weighted	0.381940451	0.05	1

		Simple mode	0.71949609	0.05	1
		Weighted mode	0.200907939	0.05	0.733532863
	IDP dMRI TBSS ICVF Posterior corona radiata L	MR Egger	0.31339062	0.05	0.005978399
		Weighted median	0.519160927	0.05	1
		Inverse variance weighted	0.353336309	0.05	1
		Simple mode	0.714323708	0.05	1
		Weighted mode	0.184446251	0.05	0.83156486
	IDP dMRI TBSS ICVF Posterior thalamic radiation R	Wald ratio	0.6792227	0.05	1
	IDP dMRI TBSS ICVF Posterior thalamic radiation L	Inverse variance weighted	0.385683116	0.05	1
	IDP dMRI TBSS ICVF Sagittal stratum R	MR Egger	0.789132935	0.05	0.140960394
		Weighted median	0.194777397	0.05	1
		Inverse variance weighted	0.204196926	0.05	1
		Simple mode	0.322913368	0.05	1
		Weighted mode	0.346672805	0.05	1
	IDP dMRI TBSS ICVF Sagittal stratum L	Inverse variance weighted	0.331185908	0.05	1
	IDP dMRI TBSS ICVF External capsule R	MR Egger	0.445090162	0.05	0.577690158
		Weighted median	0.07978289	0.05	0.999793764
		Inverse variance weighted	0.055058758	0.05	0.999596935
		Simple mode	0.532677008	0.05	1
		Weighted mode	0.203691987	0.05	0.98717417
	IDP dMRI TBSS ICVF External capsule L	Inverse variance weighted	0.057921468	0.05	0.997727932

	IDP dMRI TBSS ICVF Cingulum cingulate gyrus R	Inverse variance weighted	0.233434107	0.05	0.999999963
	IDP dMRI TBSS ICVF Cingulum cingulate gyrus L	Wald ratio	0.706904808	0.05	1
	IDP dMRI TBSS ICVF Cingulum hippocampus R	MR Egger	0.285709686	0.05	0.045963074
		Weighted median	0.276208545	0.05	1
		Inverse variance weighted	0.34950122	0.05	1
		Simple mode	0.578122062	0.05	1
		Weighted mode	0.206525236	0.05	0.997928946
	IDP dMRI TBSS ICVF Cingulum hippocampus L	MR Egger	0.449636295	0.05	0.646224195
		Weighted median	0.067145794	0.05	0.999752934
		Inverse variance weighted	0.087417651	0.05	0.999999177
		Simple mode	0.266644914	0.05	0.99343968
		Weighted mode	0.202449767	0.05	0.995763759
	IDP dMRI TBSS ICVF Fornix cres+Stria terminalis R	Wald ratio	0.568521299	0.05	1
	IDP dMRI TBSS ICVF Superior longitudinal fasciculus R	Inverse variance weighted	0.877489454	0.05	1
	IDP dMRI TBSS ICVF Superior longitudinal fasciculus L	MR Egger	0.612337298	0.05	0.608551242
		Weighted median	0.033908495	0.05	0.982011657
		Inverse variance weighted	0.134729869	0.05	0.999999906
		Simple mode	0.296063913	0.05	0.979129296
		Weighted mode	0.212901479	0.05	0.966880838
	IDP dMRI TBSS ICVF Superior fronto-occipital fasciculus R	Inverse variance weighted	0.087938775	0.05	0.724407062
	IDP dMRI TBSS ICVF Uncinate fasciculus R	Wald ratio	0.056308689	0.05	0.961294287

	IDP dMRI TBSS ICVF Uncinate fasciculus L	Wald ratio	0.056308689	0.05	0.764700445
	IDP dMRI TBSS OD Pontine crossing tract	MR Egger	0.187157914	0.05	1
		Weighted median	0.874835957	0.05	1
		Inverse variance weighted	0.686444422	0.05	1
		Simple mode	0.610401497	0.05	0.999999981
		Weighted mode	0.686059961	0.05	1
	IDP dMRI TBSS OD Cerebral peduncle R	Wald ratio	0.761231564	0.05	1
	IDP dMRI TBSS OD Cerebral peduncle L	Wald ratio	0.761231564	0.05	1
	IDP dMRI TBSS OD Posterior limb of internal capsule L	Wald ratio	0.712421821	0.05	1
	IDP dMRI TBSS OD External capsule R	Inverse variance weighted	0.312197884	0.05	0.95631233
	IDP dMRI TBSS OD External capsule L	Wald ratio	0.747934218	0.05	1
	IDP dMRI TBSS OD Superior longitudinal fasciculus R	Wald ratio	0.42455505	0.05	1
	IDP dMRI TBSS ISOVF Fornix	Wald ratio	0.40339624	0.05	1
	IDP dMRI TBSS ISOVF External capsule R	Wald ratio	0.714497931	0.05	1
	IDP dMRI TBSS ISOVF Cingulum cingulate gyrus R	Wald ratio	0.481242934	0.05	1
	IDP dMRI ProbtrackX FA ar l	Wald ratio	0.056308689	0.05	0.070485422
	IDP dMRI ProbtrackX FA atr l	Wald ratio	0.056308689	0.05	0.33628552
	IDP dMRI ProbtrackX FA fmi	MR Egger	0.24081206	0.05	0.004235061
		Weighted median	0.097042835	0.05	0.99525306
		Inverse variance weighted	0.24167039	0.05	1
		Simple mode	0.728271087	0.05	1
		Weighted mode	0.173871237	0.05	0.865266779

	IDP dMRI ProbtrackX FA ifo l	Wald ratio	0.6792227	0.05	1
	IDP dMRI ProbtrackX FA ifo r	Wald ratio	0.72680261	0.05	1
	IDP dMRI ProbtrackX FA ml l	Wald ratio	0.29888578	0.05	1
	IDP dMRI ProbtrackX FA ptr r	Wald ratio	0.070976685	0.05	0.870227454
	IDP dMRI ProbtrackX FA slf l	Wald ratio	0.72680261	0.05	1
	IDP dMRI ProbtrackX FA str l	Wald ratio	0.684635604	0.05	1
	IDP dMRI ProbtrackX FA unc l	Wald ratio	0.056308689	0.05	0.128560412
	IDP dMRI ProbtrackX FA unc r	Wald ratio	0.056308689	0.05	0.238472403
	IDP dMRI ProbtrackX MD ar l	Wald ratio	0.056308689	0.05	1
	IDP dMRI ProbtrackX MD ar r	Wald ratio	0.056308689	0.05	1
	IDP dMRI ProbtrackX MD atr l	Wald ratio	0.056308689	0.05	1
	IDP dMRI ProbtrackX MD atr r	Wald ratio	0.056308689	0.05	1
	IDP dMRI ProbtrackX MD cgc l	MR Egger	0.345590588	0.05	1
		Weighted median	0.164344101	0.05	1
		Inverse variance weighted	0.146618129	0.05	1
		Simple mode	0.603620344	0.05	1
		Weighted mode	0.225272905	0.05	1
	IDP dMRI ProbtrackX MD cgc r	Inverse variance weighted	0.971942636	0.05	1
	IDP dMRI ProbtrackX MD fmi	Inverse variance weighted	0.354499204	0.05	1
	IDP dMRI ProbtrackX MD ifo l	Wald ratio	0.6792227	0.05	1
	IDP dMRI ProbtrackX MD ifo r	Wald ratio	0.6792227	0.05	1
	IDP dMRI ProbtrackX MD ilf l	Wald ratio	0.6792227	0.05	1
	IDP dMRI ProbtrackX MD ilf r	Wald ratio	0.6792227	0.05	1

	IDP dMRI ProbtrackX MD ptr l	Wald ratio	0.056308689	0.05	1
	IDP dMRI ProbtrackX MD ptr r	Wald ratio	0.056308689	0.05	1
	IDP dMRI ProbtrackX MD slf l	Inverse variance weighted	0.381354117	0.05	1
	IDP dMRI ProbtrackX MD slf r	Wald ratio	0.6792227	0.05	1
	IDP dMRI ProbtrackX MD str l	Wald ratio	0.680715848	0.05	1
	IDP dMRI ProbtrackX MD str r	Wald ratio	0.056308689	0.05	1
	IDP dMRI ProbtrackX MD unc l	Wald ratio	0.056308689	0.05	1
	IDP dMRI ProbtrackX MD unc r	Inverse variance weighted	0.090170354	0.05	1
	IDP dMRI ProbtrackX MO atr l	Wald ratio	0.381951419	0.05	0.971068468
	IDP dMRI ProbtrackX MO ml r	Wald ratio	0.859055262	0.05	1
	IDP T1 FAST ROIs L intracalc cortex	Wald ratio	0.049417175	0.05	1
	IDP dMRI ProbtrackX L1 ilf l	Wald ratio	0.056308689	0.05	1
	IDP T1 FAST ROIs R intracalc cortex	Wald ratio	0.049417175	0.05	1
	IDP dMRI ProbtrackX L1 slf l	Wald ratio	0.056308689	0.05	1
	IDP dMRI ProbtrackX L1 slf r	Wald ratio	0.542371488	0.05	0.948578093
	IDP dMRI ProbtrackX L1 str r	Wald ratio	0.454212957	0.05	0.938570117
	IDP dMRI ProbtrackX L1 unc l	Inverse variance weighted	0.073367092	0.05	1
	IDP dMRI ProbtrackX L1 unc r	Wald ratio	0.848857283	0.05	1
	IDP dMRI ProbtrackX L2 ar l	Wald ratio	0.056308689	0.05	1
	IDP dMRI ProbtrackX L2 ar r	Wald ratio	0.056308689	0.05	1
	IDP dMRI ProbtrackX L2 atr l	Wald ratio	0.056308689	0.05	1
	IDP dMRI ProbtrackX L2 atr r	Wald ratio	0.056308689	0.05	1
	IDP dMRI ProbtrackX L2 cgh l	Wald ratio	0.63179072	0.05	1

	IDP dMRI ProbtrackX L2 fma	Wald ratio	0.106601512	0.05	0.051140244
	IDP dMRI ProbtrackX L2 fmi	Inverse variance weighted	0.356893378	0.05	1
	IDP dMRI ProbtrackX L2 ifo l	Wald ratio	0.6792227	0.05	1
	IDP dMRI ProbtrackX L2 ifo r	Wald ratio	0.6792227	0.05	1
	IDP dMRI ProbtrackX L2 ilf l	Wald ratio	0.6792227	0.05	1
	IDP dMRI ProbtrackX L2 ilf r	Wald ratio	0.6792227	0.05	1
	IDP dMRI ProbtrackX L2 ptr l	Wald ratio	0.056308689	0.05	1
	IDP dMRI ProbtrackX L2 ptr r	Wald ratio	0.05362453	0.05	1
	IDP dMRI ProbtrackX L2 slf l	Inverse variance weighted	0.388210026	0.05	1
	IDP dMRI ProbtrackX L2 slf r	Wald ratio	0.6792227	0.05	1
	IDP dMRI ProbtrackX L2 str l	Wald ratio	0.680715848	0.05	1
	IDP dMRI ProbtrackX L2 str r	Wald ratio	0.680715848	0.05	1
	IDP dMRI ProbtrackX L2 unc l	Wald ratio	0.056308689	0.05	1
	IDP dMRI ProbtrackX L2 unc r	Inverse variance weighted	0.086762526	0.05	1
	IDP dMRI ProbtrackX L3 ar l	Wald ratio	0.056308689	0.05	1
	IDP dMRI ProbtrackX L3 ar r	Wald ratio	0.056308689	0.05	1
	IDP dMRI ProbtrackX L3 atr l	Wald ratio	0.056308689	0.05	1
	IDP dMRI ProbtrackX L3 atr r	Wald ratio	0.056308689	0.05	1
	IDP dMRI ProbtrackX L3 fmi	MR Egger	0.518894137	0.05	1
		Weighted median	0.057965953	0.05	1
		Inverse variance weighted	0.127167605	0.05	1
		Simple mode	0.342948745	0.05	1

		Weighted mode	0.206347834	0.05	1
	IDP dMRI ProbtrackX L3 ifo l	Inverse variance weighted	0.312346754	0.05	1
	IDP dMRI ProbtrackX L3 ifo r	Wald ratio	0.6792227	0.05	1
	IDP dMRI ProbtrackX L3 ilf l	Wald ratio	0.6792227	0.05	1
	IDP dMRI ProbtrackX L3 ilf r	Wald ratio	0.6792227	0.05	1
	IDP dMRI ProbtrackX L3 ptr l	Wald ratio	0.056308689	0.05	1
	IDP dMRI ProbtrackX L3 ptr r	Wald ratio	0.05362453	0.05	1
	IDP dMRI ProbtrackX L3 slf l	Inverse variance weighted	0.368349519	0.05	1
	IDP dMRI ProbtrackX L3 slf r	Wald ratio	0.6792227	0.05	1
	IDP dMRI ProbtrackX L3 str l	Wald ratio	0.05362453	0.05	1
	IDP dMRI ProbtrackX L3 unc l	Wald ratio	0.056308689	0.05	1
	IDP dMRI ProbtrackX L3 unc r	Inverse variance weighted	0.096481911	0.05	1
	IDP dMRI ProbtrackX ICVF ar l	MR Egger	0.463109048	0.05	0.94153713
		Weighted median	0.033121192	0.05	0.998233865
		Inverse variance weighted	0.038431662	0.05	0.999801334
		Simple mode	0.283297681	0.05	0.998239389
		Weighted mode	0.181014079	0.05	0.998239389
	IDP dMRI ProbtrackX ICVF ar r	MR Egger	0.467773618	0.05	0.871397458
		Weighted median	0.033910093	0.05	0.994328143
		Inverse variance weighted	0.039540662	0.05	0.999447985
		Simple mode	0.269193039	0.05	0.994364211
		Weighted mode	0.201885368	0.05	0.994226541

	IDP dMRI ProbtrackX ICVF atr l	MR Egger	0.40459299	0.05	0.18141141
		Weighted median	0.039305245	0.05	0.96366492
		Inverse variance weighted	0.083845474	0.05	0.999897732
		Simple mode	0.281932096	0.05	0.966524621
		Weighted mode	0.192760946	0.05	0.957129946
	IDP dMRI ProbtrackX ICVF atr r	MR Egger	0.401061854	0.05	0.111168723
		Weighted median	0.038205626	0.05	0.96661418
		Inverse variance weighted	0.085996274	0.05	0.999904464
		Simple mode	0.296063103	0.05	0.970840365
		Weighted mode	0.194340031	0.05	0.952506733
	IDP dMRI ProbtrackX ICVF cgc l	Wald ratio	0.706904808	0.05	1
	IDP dMRI ProbtrackX ICVF cgh l	MR Egger	0.400327079	0.05	0.362561318
		Weighted median	0.047234926	0.05	0.996483506
		Inverse variance weighted	0.082303584	0.05	0.999997831
		Simple mode	0.29833151	0.05	0.996921414
		Weighted mode	0.201964383	0.05	0.995541693
	IDP dMRI ProbtrackX ICVF cgh r	Wald ratio	0.056308689	0.05	0.989136539
	IDP dMRI ProbtrackX ICVF cst l	MR Egger	0.466896639	0.05	0.313553492
		Weighted median	0.049479014	0.05	0.98600453
		Inverse variance weighted	0.042575544	0.05	0.997753745
		Simple mode	0.271466522	0.05	0.98751487
		Weighted mode	0.227789092	0.05	0.966106283

	IDP dMRI ProbtrackX ICVF cst r	MR Egger	0.211697457	0.05	0.012448311
		Weighted median	0.053440346	0.05	0.981150942
		Inverse variance weighted	0.31352122	0.05	1
		Simple mode	0.408623363	0.05	0.998296869
		Weighted mode	0.174739929	0.05	0.966279981
	IDP dMRI ProbtrackX ICVF fma	Inverse variance weighted	0.507542092	0.05	1
	IDP dMRI ProbtrackX ICVF fmi	MR Egger	0.269081583	0.05	0.110086124
		Weighted median	0.042604177	0.05	0.998366877
		Inverse variance weighted	0.207303405	0.05	1
		Simple mode	0.706072716	0.05	1
		Weighted mode	0.155309752	0.05	0.997234911
	IDP dMRI ProbtrackX ICVF ifo l	Inverse variance weighted	0.25686993	0.05	0.999999997
	IDP dMRI ProbtrackX ICVF ifo r	Inverse variance weighted	0.26192281	0.05	0.999999993
	IDP dMRI ProbtrackX ICVF ilf l	Inverse variance weighted	0.277640253	0.05	1
	IDP dMRI ProbtrackX ICVF ilf r	Wald ratio	0.6792227	0.05	1
	IDP dMRI ProbtrackX ICVF mcp	Wald ratio	0.904373127	0.05	1
	IDP dMRI ProbtrackX ICVF ml l	Wald ratio	0.072611407	0.05	0.847167651
	IDP dMRI ProbtrackX ICVF ml r	Wald ratio	0.056308689	0.05	0.60672219
	IDP dMRI ProbtrackX ICVF ptr l	Wald ratio	0.6792227	0.05	1
	IDP dMRI ProbtrackX ICVF ptr r	Inverse variance weighted	0.359989385	0.05	1
	IDP dMRI ProbtrackX ICVF slf l	MR Egger	0.520919403	0.05	0.385819524
		Weighted median	0.035526707	0.05	0.992667031

		Inverse variance weighted	0.107059043	0.05	0.999999825
		Simple mode	0.300953787	0.05	0.992714286
		Weighted mode	0.203249853	0.05	0.986248808
	IDP dMRI ProbtrackX ICVF slf r	Inverse variance weighted	0.866882512	0.05	1
	IDP dMRI ProbtrackX ICVF str l	MR Egger	0.423837976	0.05	0.043902061
		Weighted median	0.041212459	0.05	0.972445721
		Inverse variance weighted	0.099748474	0.05	0.999972046
		Simple mode	0.29072179	0.05	0.970127723
		Weighted mode	0.193554135	0.05	0.931364998
	IDP dMRI ProbtrackX ICVF str r	MR Egger	0.484291133	0.05	0.076176798
		Weighted median	0.04558613	0.05	0.952034706
		Inverse variance weighted	0.105450718	0.05	0.999972845
		Simple mode	0.297995131	0.05	0.950808412
		Weighted mode	0.210945448	0.05	0.904767605
	IDP dMRI ProbtrackX ICVF unc l	Inverse variance weighted	0.037014552	0.05	0.975219036
	IDP dMRI ProbtrackX ICVF unc r	Inverse variance weighted	0.178152505	0.05	0.999998682
	IDP dMRI ProbtrackX OD atr r	Wald ratio	0.901496287	0.05	1
	IDP dMRI ProbtrackX OD ilf l	Wald ratio	0.482331743	0.05	1
	IDP dMRI ProbtrackX OD slf l	Wald ratio	0.32509042	0.05	0.19889431
	IDP dMRI ProbtrackX OD str l	Inverse variance weighted	0.542495302	0.05	1
	IDP dMRI ProbtrackX ISOVF atr r	Wald ratio	0.056308689	0.05	0.045343096
	IDP dMRI ProbtrackX ISOVF cgc l	Wald ratio	0.12205181	0.05	0.145483073

	IDP dMRI ProbtrackX ISOVF ifo r	Wald ratio	0.719118031	0.05	1
	IDP dMRI ProbtrackX ISOVF ilf l	Wald ratio	0.950954694	0.05	1
	IDP dMRI ProbtrackX ISOVF ilf r	MR Egger	0.689865973	0.05	0.002542767
		Weighted median	0.850478517	0.05	1
		Inverse variance weighted	0.847836014	0.05	1
		Simple mode	0.854441597	0.05	1
		Weighted mode	0.849045818	0.05	1
	IDP dMRI ProbtrackX ISOVF slf l	Inverse variance weighted	0.594535154	0.05	1
	IDP dMRI ProbtrackX ISOVF slf r	MR Egger	0.990201671	0.05	1
		Weighted median	0.758306437	0.05	1
		Inverse variance weighted	0.585310006	0.05	1
		Simple mode	0.915845146	0.05	1
		Weighted mode	0.911749714	0.05	1
	IDP dMRI ProbtrackX ISOVF unc l	Wald ratio	0.933119231	0.05	1
	IDP dMRI ProbtrackX ISOVF unc r	Wald ratio	0.854922723	0.05	1
	IDP T1 SIENAX brain-normalised volume	Wald ratio	0.134759599	0.05	1
Fibromyalgia	IDP T1 SIENAX peripheral grey normalised volume	Wald ratio	0.203434002	0.05	1
	IDP T1 FAST ROIs R temp occ fusif cortex	Wald ratio	0.280458775	0.05	1
	IDP T1 FAST ROIs R occ pole	Inverse variance weighted	0.741438425	0.05	1
	IDP T1 FAST ROIs L thalamus	Inverse variance weighted	0.985576327	0.05	1
	IDP T1 FAST ROIs R thalamus	Wald ratio	0.294321215	0.05	1
	IDP T1 FAST ROIs L putamen	Wald ratio	0.625703132	0.05	1

	IDP T1 FAST ROIs R putamen	Wald ratio	0.625703132	0.05	1
	IDP T1 FAST ROIs L pallidum	Wald ratio	0.828030303	0.05	1
	IDP T1 FIRST left caudate volume	Wald ratio	0.85801016	0.05	1
	IDP T1 FAST ROIs L hippocampus	Inverse variance weighted	0.789667693	0.05	1
	IDP T1 FAST ROIs R hippocampus	Wald ratio	0.087404447	0.05	1
	IDP T1 FAST ROIs L ventral striatum	Wald ratio	0.625703132	0.05	1
	IDP T1 FAST ROIs R ventral striatum	Inverse variance weighted	0.338652028	0.05	1
	IDP T1 FAST ROIs L cerebellum VI	Wald ratio	0.535884989	0.05	1
	IDP T1 FAST ROIs L cerebellum crus I	MR Egger	0.279284092	0.05	1
		Weighted median	0.208190474	0.05	1
		Inverse variance weighted	0.276639982	0.05	1
		Simple mode	0.631328674	0.05	1
		Weighted mode	0.194402645	0.05	1
	IDP T1 FAST ROIs R cerebellum crus I	Inverse variance weighted	0.324190783	0.05	1
	IDP T1 FAST ROIs L cerebellum crus II	MR Egger	0.706872753	0.05	1
		Weighted median	0.871720216	0.05	1
		Inverse variance weighted	0.411552629	0.05	1
		Simple mode	0.943276256	0.05	1
		Weighted mode	0.964000486	0.05	1
	IDP T1 FAST ROIs V cerebellum crus II	Inverse variance weighted	0.250346481	0.05	1
	IDP T1 FAST ROIs R cerebellum crus II	Inverse variance weighted	0.941450342	0.05	1

	IDP T1 FIRST left putamen volume	Inverse variance weighted	0.661101004	0.05	1
	IDP T1 FAST ROIs R cerebellum VIIb	Wald ratio	0.582658968	0.05	1
	IDP T1 FAST ROIs V cerebellum VIIIa	MR Egger	0.314027317	0.05	1
		Weighted median	0.069195482	0.05	1
		Inverse variance weighted	0.036082417	0.05	1
		Simple mode	0.312327787	0.05	1
		Weighted mode	0.284606168	0.05	1
	IDP T1 FAST ROIs L cerebellum VIIIb	Wald ratio	0.848287155	0.05	1
	IDP T1 FAST ROIs V cerebellum VIIIb	Inverse variance weighted	0.189221318	0.05	1
	IDP T1 FAST ROIs R cerebellum VIIIb	Wald ratio	0.625703132	0.05	1
	IDP T1 FAST ROIs L cerebellum IX	Inverse variance weighted	0.653560285	0.05	1
	IDP T1 FIRST right putamen volume	Wald ratio	0.298606414	0.05	1
	IDP T1 FAST ROIs V cerebellum IX	Inverse variance weighted	0.55870976	0.05	1
	IDP T1 FAST ROIs R cerebellum IX	Inverse variance weighted	0.418517677	0.05	1
	IDP T1 FAST ROIs V cerebellum X	Wald ratio	0.625703132	0.05	1
	IDP T2 FLAIR BIANCA WMH volume	Inverse variance weighted	0.700470958	0.05	1
	IDP SWI T2star left caudate	MR Egger	0.335587494	0.05	1
		Weighted median	0.965635929	0.05	1
		Inverse variance weighted	0.935999162	0.05	1
		Simple mode	0.978273845	0.05	1
		Weighted mode	0.669772141	0.05	1

	IDP SWI T2star right caudate	MR Egger	0.796354187	0.05	1
		Weighted median	0.600542745	0.05	1
		Inverse variance weighted	0.853895483	0.05	1
		Simple mode	0.656867826	0.05	1
		Weighted mode	0.508338333	0.05	1
	IDP T1 FIRST left pallidum volume	Wald ratio	0.756057767	0.05	1
	IDP SWI T2star left putamen	MR Egger	0.669953019	0.05	1
		Weighted median	0.952776029	0.05	1
		Inverse variance weighted	0.582140166	0.05	1
		Simple mode	0.993116577	0.05	1
		Weighted mode	0.942790338	0.05	1
	IDP SWI T2star right putamen	MR Egger	0.140461834	0.05	1
		Weighted median	0.880207653	0.05	1
		Inverse variance weighted	0.718689089	0.05	1
		Simple mode	0.706181296	0.05	1
		Weighted mode	0.697041044	0.05	1
	IDP SWI T2star left pallidum	MR Egger	0.468676367	0.05	1
		Weighted median	0.083423604	0.05	1
		Inverse variance weighted	0.022296175	0.05	1
		Simple mode	0.213415845	0.05	1
		Weighted mode	0.196496711	0.05	1
	IDP SWI T2star right pallidum	MR Egger	0.341948496	0.05	0.999999992

		Weighted median	0.133100151	0.05	1
		Inverse variance weighted	0.08740218	0.05	1
		Simple mode	0.892953874	0.05	1
		Weighted mode	0.298914024	0.05	1
	IDP T1 FIRST right pallidum volume	Wald ratio	0.756057767	0.05	1
	IDP dMRI TBSS FA Genu of corpus callosum	Wald ratio	0.776730664	0.05	1
	IDP dMRI TBSS FA Splenium of corpus callosum	Inverse variance weighted	0.241250115	0.05	1
	IDP dMRI TBSS FA Corticospinal tract L	Wald ratio	0.13549747	0.05	1
	IDP dMRI TBSS FA Superior cerebellar peduncle R	Inverse variance weighted	0.412185674	0.05	1
	IDP dMRI TBSS FA Superior cerebellar peduncle L	Inverse variance weighted	0.400903191	0.05	1
	IDP dMRI TBSS FA Posterior limb of internal capsule R	Wald ratio	0.917965943	0.05	1
	IDP dMRI TBSS FA Retrolenticular part of internal capsule R	Inverse variance weighted	0.48997147	0.05	1
	IDP dMRI TBSS FA Anterior corona radiata R	Wald ratio	0.776730664	0.05	1
	IDP dMRI TBSS FA Anterior corona radiata L	Wald ratio	0.776730664	0.05	1
	IDP dMRI TBSS FA Posterior thalamic radiation R	Wald ratio	0.295081901	0.05	1
	IDP dMRI TBSS FA Posterior thalamic radiation L	Wald ratio	0.287120397	0.05	1
	IDP dMRI TBSS FA Sagittal stratum R	Wald ratio	0.156266122	0.05	1
	IDP dMRI TBSS FA Cingulum cingulate gyrus R	Inverse variance weighted	0.559240305	0.05	1
	IDP dMRI TBSS FA Superior longitudinal fasciculus L	Inverse variance weighted	0.785658881	0.05	1
	IDP dMRI TBSS FA Uncinate fasciculus L	Wald ratio	0.044559691	0.05	1

	IDP dMRI TBSS MD Genu of corpus callosum	Wald ratio	0.776730664	0.05	1
	IDP dMRI TBSS MD Body of corpus callosum	Wald ratio	0.354438538	0.05	1
	IDP dMRI TBSS MD Splenium of corpus callosum	Inverse variance weighted	0.169248361	0.05	1
	IDP dMRI TBSS MD Corticospinal tract R	Wald ratio	0.677839201	0.05	1
	IDP dMRI TBSS MD Superior cerebellar peduncle R	Wald ratio	0.300865964	0.05	1
	IDP dMRI TBSS MD Superior cerebellar peduncle L	Wald ratio	0.401085237	0.05	1
	IDP dMRI TBSS MD Anterior limb of internal capsule L	Wald ratio	0.247545895	0.05	1
	IDP dMRI TBSS MD Retrolenticular part of internal capsule R	Wald ratio	0.295081901	0.05	1
	IDP T1 FIRST left caudate volume plus IDP T1 FIRST right caudate volume	Wald ratio	0.724206288	0.05	1
	IDP T1 FIRST left putamen volume plus IDP T1 FIRST right putamen volume	Inverse variance weighted	0.84162363	0.05	1
	IDP T1 FIRST left pallidum volume plus IDP T1 FIRST right pallidum volume	Wald ratio	0.756057767	0.05	1
	IDP SWI T2star left thalamus plus IDP SWI T2star right thalamus	MR Egger	0.34050398	0.05	1
		Weighted median	0.565523696	0.05	1
		Inverse variance weighted	0.470847542	0.05	1
		Simple mode	0.571275041	0.05	1
		Weighted mode	0.755415621	0.05	1
	IDP dMRI TBSS MD Retrolenticular part of internal capsule L	Wald ratio	0.295081901	0.05	1
	IDP SWI T2star left caudate plus IDP SWI T2star right caudate	MR Egger	0.612567989	0.05	1
		Weighted median	0.782261876	0.05	1
		Inverse variance weighted	0.973449151	0.05	1

		Simple mode	0.85657418	0.05	1
		Weighted mode	0.705006944	0.05	1
	IDP SWI T2star left putamen plus IDP SWI T2star right putamen	MR Egger	0.68174025	0.05	1
		Weighted median	0.837583918	0.05	1
		Inverse variance weighted	0.983106522	0.05	1
		Simple mode	0.664397317	0.05	1
		Weighted mode	0.654390993	0.05	1
	IDP SWI T2star left pallidum plus IDP SWI T2star right pallidum	MR Egger	0.318819586	0.05	1
		Weighted median	0.072218876	0.05	1
		Inverse variance weighted	0.034953347	0.05	1
		Simple mode	0.178732655	0.05	1
		Weighted mode	0.188243703	0.05	1
	volume Left-Lateral-Ventricle	MR Egger	0.999936744	0.05	1
		Weighted median	0.971293271	0.05	1
		Inverse variance weighted	0.603521899	0.05	1
		Simple mode	0.863998952	0.05	1
		Weighted mode	0.806819127	0.05	1
	volume Left-Cerebellum-White-Matter	Inverse variance weighted	0.820356272	0.05	1
	volume Left-Cerebellum-Cortex	Inverse variance weighted	0.737181633	0.05	1
	IDP dMRI TBSS MD Anterior corona radiata R	Inverse variance weighted	0.388966655	0.05	1
	volume Left-Putamen	Inverse variance weighted	0.186176052	0.05	1

	volume 4th-Ventricle	Inverse variance weighted	0.743133227	0.05	1
	volume Brain-Stem	Wald ratio	0.212132467	0.05	1
	volume Left-Hippocampus	Inverse variance weighted	0.354108532	0.05	1
	volume CSF	MR Egger	0.738529457	0.05	1
		Weighted median	0.802668986	0.05	1
		Inverse variance weighted	0.680631683	0.05	1
		Simple mode	0.992116767	0.05	1
		Weighted mode	0.987560736	0.05	1
	IDP dMRI TBSS MD Anterior corona radiata L	Inverse variance weighted	0.405758908	0.05	1
	volume Left-Accumbens-area	Wald ratio	0.625703132	0.05	1
	volume Right-Lateral-Ventricle	Inverse variance weighted	0.741494912	0.05	1
	volume Right-Inf-Lat-Vent	Wald ratio	0.038685093	0.05	1
	volume Right-Cerebellum-White-Matter	Wald ratio	0.143829241	0.05	1
	volume Right-Cerebellum-Cortex	MR Egger	0.319385838	0.05	1
		Weighted median	0.445629131	0.05	1
		Inverse variance weighted	0.356147064	0.05	1
		Simple mode	0.695855135	0.05	1
		Weighted mode	0.208994098	0.05	1
	IDP dMRI TBSS MD Superior corona radiata R	Inverse variance weighted	0.389286306	0.05	1
	volume Right-Putamen	Wald ratio	0.298606414	0.05	1
	volume Right-Hippocampus	Wald ratio	0.087404447	0.05	1
	volume Right-Amygdala	Wald ratio	0.194230595	0.05	1

	volume Right-VentralDC	Wald ratio	0.869894021	0.05	1
	volume CC Posterior	Wald ratio	0.471616385	0.05	1
	IDP dMRI TBSS MD Superior corona radiata L	Inverse variance weighted	0.391011848	0.05	1
	IDP dMRI TBSS MD Posterior corona radiata R	Wald ratio	0.295081901	0.05	1
	volume BrainSegVol-to-eTIV	Wald ratio	0.106606324	0.05	1
	IDP dMRI TBSS MD Posterior corona radiata L	Wald ratio	0.287120397	0.05	1
	volume rhSurfaceHoles	Wald ratio	0.115056141	0.05	1
	DKTatlas lh cuneus area	Wald ratio	0.323216016	0.05	1
	IDP dMRI TBSS MD Posterior thalamic radiation R	Wald ratio	0.287120397	0.05	1
	DKTatlas lh lateraloccipital area	Wald ratio	0.323216016	0.05	1
	DKTatlas lh lingual area	Wald ratio	0.417621381	0.05	1
	IDP dMRI TBSS MD Posterior thalamic radiation L	Wald ratio	0.295081901	0.05	1
	DKTatlas lh parstriangularis area	Wald ratio	0.22850249	0.05	1
	DKTatlas lh pericalcarine area	Inverse variance weighted	0.28132997	0.05	1
	DKTatlas lh postcentral area	Inverse variance weighted	0.944952866	0.05	1
	DKTatlas lh posteriorcingulate area	Wald ratio	0.698716112	0.05	1
	DKTatlas lh precentral area	Wald ratio	0.452441232	0.05	1
	DKTatlas lh precuneus area	Wald ratio	0.847448983	0.05	1
	IDP dMRI TBSS MD Sagittal stratum R	Wald ratio	0.295081901	0.05	1
	DKTatlas lh superiorparietal area	Wald ratio	0.338882387	0.05	1
	DKTatlas lh superiortemporal area	Wald ratio	0.286724019	0.05	1
	DKTatlas lh supramarginal area	Wald ratio	0.787842611	0.05	1

	DKTatlas lh WhiteSurfArea area	Wald ratio	0.966764427	0.05	1
	IDP dMRI TBSS MD Sagittal stratum L	Wald ratio	0.295081901	0.05	1
	a2009s lh G&S subcentral area	Wald ratio	0.452441232	0.05	1
	a2009s lh G cuneus area	Wald ratio	0.546718552	0.05	1
	a2009s lh G front inf-Opercular area	Wald ratio	0.786000055	0.05	1
	IDP dMRI TBSS MD External capsule R	Wald ratio	0.776730664	0.05	1
	IDP dMRI TBSS MD External capsule L	Inverse variance weighted	0.66475996	0.05	1
	a2009s lh G pariet inf-Supramar area	Wald ratio	0.787842611	0.05	1
	a2009s lh G parietal sup area	Wald ratio	0.338882387	0.05	1
	a2009s lh G postcentral area	Wald ratio	0.338317768	0.05	1
	a2009s lh G precentral area	Wald ratio	0.452441232	0.05	1
	a2009s lh G precuneus area	Wald ratio	0.546718552	0.05	1
	IDP dMRI TBSS MD Cingulum cingulate gyrus R	MR Egger	0.863207613	0.05	1
		Weighted median	0.664831981	0.05	1
		Inverse variance weighted	0.811004211	0.05	1
		Simple mode	0.603370464	0.05	1
		Weighted mode	0.685476594	0.05	1
	IDP dMRI TBSS MD Cingulum cingulate gyrus L	MR Egger	0.841664818	0.05	1
		Weighted median	0.685545876	0.05	1
		Inverse variance weighted	0.818406846	0.05	1
		Simple mode	0.613688456	0.05	1
		Weighted mode	0.704034953	0.05	1
	a2009s lh S calcarine area	Wald ratio	0.417621381	0.05	1

	a2009s lh S central area	Wald ratio	0.454394952	0.05	1
	a2009s lh S collat transv ant area	Wald ratio	0.004405842	0.05	0.925152539
	a2009s lh S front middle area	Wald ratio	0.545242108	0.05	0.999993838
	IDP dMRI TBSS MD Cingulum hippocampus R	Wald ratio	0.295081901	0.05	1
	a2009s lh S intrapariet&P trans area	Wald ratio	0.338882387	0.05	1
	IDP dMRI TBSS MD Cingulum hippocampus L	Wald ratio	0.295081901	0.05	1
	a2009s lh S subparietal area	Wald ratio	0.417621381	0.05	1
	DKTatlas rh cuneus area	Wald ratio	0.417621381	0.05	1
	DKTatlas rh lingual area	Wald ratio	0.416844433	0.05	1
	DKTatlas rh parstriangularis area	Wald ratio	0.40997841	0.05	1
	DKTatlas rh pericalcarine area	Inverse variance weighted	0.426613148	0.05	1
	IDP dMRI TBSS MD Superior longitudinal fasciculus R	Wald ratio	0.295081901	0.05	1
	DKTatlas rh postcentral area	Wald ratio	0.454394952	0.05	1
	DKTatlas rh precentral area	Wald ratio	0.452441232	0.05	1
	IDP dMRI TBSS MD Superior longitudinal fasciculus L	Wald ratio	0.295081901	0.05	1
	a2009s rh G&S subcentral area	Wald ratio	0.454394952	0.05	1
	a2009s rh G&S cingul-Mid-Post area	Wald ratio	0.49055913	0.05	1
	a2009s rh G cuneus area	Wald ratio	0.417621381	0.05	1
	IDP dMRI TBSS MD Superior fronto-occipital fasciculus L	Wald ratio	0.185530566	0.05	1
	a2009s rh G oc-temp med-Lingual area	Wald ratio	0.416844433	0.05	1
	a2009s rh G parietal sup area	Wald ratio	0.376332618	0.05	1
	IDP dMRI TBSS MD Uncinate fasciculus R	Wald ratio	0.274951238	0.05	1

	a2009s rh G precentral area	Wald ratio	0.452441232	0.05	1
	a2009s rh G precuneus area	Wald ratio	0.752535018	0.05	1
	IDP dMRI TBSS MD Uncinate fasciculus L	Inverse variance weighted	0.756215533	0.05	1
	a2009s rh Pole occipital area	Wald ratio	0.417621381	0.05	1
	a2009s rh S calcarine area	Wald ratio	0.416844433	0.05	1
	a2009s rh S central area	Wald ratio	0.452441232	0.05	1
	a2009s rh S collat transv ant area	Wald ratio	0.004405842	0.05	0.948027538
	a2009s rh S orbital med-olfact area	Wald ratio	0.380234236	0.05	1
	IDP dMRI TBSS MO Pontine crossing tract	MR Egger	0.419339674	0.05	1
		Weighted median	0.642787309	0.05	1
		Inverse variance weighted	0.155979254	0.05	1
		Simple mode	0.990213508	0.05	1
		Weighted mode	0.885240286	0.05	1
	DKTatlas lh postcentral thickness	Wald ratio	0.454394952	0.05	1
	a2009s lh G insular short thickness	Wald ratio	0.032950303	0.05	1
	a2009s lh G postcentral thickness	Wald ratio	0.454394952	0.05	1
	IDP T1 SIENAX CSF normalised volume	Inverse variance weighted	0.333816266	0.05	1
	IDP dMRI TBSS MO Medial lemniscus R	Wald ratio	0.042286397	0.05	0.997743205
	a2009s lh S postcentral thickness	Wald ratio	0.874485603	0.05	1
	DKTatlas rh lateraloccipital thickness	Wald ratio	0.625703132	0.05	1
	DKTatlas rh parstriangularis thickness	Wald ratio	0.766239545	0.05	1
	DKTatlas rh postcentral thickness	Wald ratio	0.454394952	0.05	1

	DKTatlas rh posteriorcingulate thickness	Inverse variance weighted	0.494142098	0.05	1
	a2009s rh G&S cingul-Mid-Ant thickness	Wald ratio	0.101923683	0.05	0.999982788
	a2009s rh G cuneus thickness	Wald ratio	0.625703132	0.05	1
	a2009s rh G postcentral thickness	Wald ratio	0.454394952	0.05	1
	a2009s rh Pole occipital thickness	Wald ratio	0.625703132	0.05	1
	a2009s rh S oc sup&transversal thickness	Wald ratio	0.751779418	0.05	1
	a2009s rh S parieto occipital thickness	Wald ratio	0.824114883	0.05	1
	IDP dMRI TBSS MO Anterior corona radiata R	Wald ratio	0.717783092	0.05	1
	IDP dMRI TBSS MO Superior corona radiata R	Wald ratio	0.963250404	0.05	1
	IDP dMRI TBSS MO Cingulum cingulate gyrus L	Inverse variance weighted	0.459954981	0.05	1
	IDP dMRI TBSS L1 Anterior limb of internal capsule L	Wald ratio	0.247545895	0.05	1
	IDP dMRI TBSS L1 Posterior limb of internal capsule R	Wald ratio	0.146435216	0.05	1
	IDP dMRI TBSS L1 Posterior limb of internal capsule L	Wald ratio	0.77203074	0.05	1
	IDP dMRI TBSS L1 Anterior corona radiata R	Wald ratio	0.776730664	0.05	1
	IDP dMRI TBSS L1 Anterior corona radiata L	Wald ratio	0.776730664	0.05	1
	IDP dMRI TBSS L1 Posterior corona radiata R	Wald ratio	0.646289093	0.05	1
	IDP dMRI TBSS L1 Posterior corona radiata L	Wald ratio	0.776730664	0.05	1
	IDP dMRI TBSS L1 Sagittal stratum L	Wald ratio	0.776730664	0.05	1
	IDP dMRI TBSS L1 External capsule R	Wald ratio	0.776730664	0.05	1
	IDP dMRI TBSS L1 External capsule L	Inverse variance weighted	0.917004739	0.05	1

	IDP dMRI TBSS L1 Fornix cres+Stria terminalis L	Wald ratio	0.257033713	0.05	1
	IDP T1 FAST ROIs L precentral gyrus	Wald ratio	0.454394952	0.05	1
	IDP dMRI TBSS L1 Superior longitudinal fasciculus R	Inverse variance weighted	0.99188862	0.05	1
	IDP dMRI TBSS L1 Uncinate fasciculus L	Wald ratio	0.820151264	0.05	1
	IDP dMRI TBSS L2 Pontine crossing tract	Wald ratio	0.042286397	0.05	1
	IDP dMRI TBSS L2 Genu of corpus callosum	MR Egger	0.79485102	0.05	1
		Weighted median	0.73296873	0.05	1
		Inverse variance weighted	0.344679191	0.05	1
		Simple mode	0.841518671	0.05	1
		Weighted mode	0.815469496	0.05	1
	IDP dMRI TBSS L2 Body of corpus callosum	Wald ratio	0.776730664	0.05	1
	IDP dMRI TBSS L2 Splenium of corpus callosum	MR Egger	0.559068666	0.05	0.088996653
		Weighted median	0.115115988	0.05	1
		Inverse variance weighted	0.124590916	0.05	1
		Simple mode	0.301516566	0.05	1
		Weighted mode	0.290252484	0.05	1
	IDP dMRI TBSS L2 Corticospinal tract R	Wald ratio	0.677839201	0.05	1
	IDP T1 SIENAX CSF unnormalised volume	MR Egger	0.870209129	0.05	1
		Weighted median	0.861998992	0.05	1
		Inverse variance weighted	0.580831434	0.05	1
		Simple mode	0.858956457	0.05	1
		Weighted mode	0.787474649	0.05	1

	IDP T1 FAST ROIs L temporal pole	Wald ratio	0.197474971	0.05	1
	IDP dMRI TBSS L2 Superior cerebellar peduncle R	Inverse variance weighted	0.410105259	0.05	1
	IDP dMRI TBSS L2 Superior cerebellar peduncle L	Wald ratio	0.401085237	0.05	1
	IDP dMRI TBSS L2 Posterior limb of internal capsule R	Inverse variance weighted	0.418973217	0.05	1
	IDP dMRI TBSS L2 Posterior limb of internal capsule L	Wald ratio	0.218001981	0.05	1
	IDP dMRI TBSS L2 Retrolenticular part of internal capsule R	Inverse variance weighted	0.354038863	0.05	1
	IDP dMRI TBSS L2 Anterior corona radiata R	Inverse variance weighted	0.39149569	0.05	1
	IDP dMRI TBSS L2 Anterior corona radiata L	Wald ratio	0.776730664	0.05	1
	IDP dMRI TBSS L2 Superior corona radiata R	Inverse variance weighted	0.25584906	0.05	1
	IDP dMRI TBSS L2 Superior corona radiata L	Wald ratio	0.295081901	0.05	1
	IDP dMRI TBSS L2 Posterior corona radiata R	Wald ratio	0.287120397	0.05	1
	IDP dMRI TBSS L2 Posterior corona radiata L	Wald ratio	0.287120397	0.05	1
	IDP dMRI TBSS L2 Posterior thalamic radiation R	Wald ratio	0.295081901	0.05	1
	IDP dMRI TBSS L2 Posterior thalamic radiation L	Wald ratio	0.287120397	0.05	1
	IDP dMRI TBSS L2 Sagittal stratum R	Wald ratio	0.295081901	0.05	1
	IDP dMRI TBSS L2 Cingulum cingulate gyrus R	Wald ratio	0.776730664	0.05	1
	IDP dMRI TBSS L2 Cingulum hippocampus R	Wald ratio	0.295081901	0.05	1
	IDP dMRI TBSS L2 Cingulum hippocampus L	Wald ratio	0.776730664	0.05	1
	IDP dMRI TBSS L2 Uncinate fasciculus L	Wald ratio	0.776730664	0.05	1

	IDP dMRI TBSS L3 Genu of corpus callosum	Wald ratio	0.776730664	0.05	1
	IDP dMRI TBSS L3 Body of corpus callosum	Wald ratio	0.776730664	0.05	1
	IDP dMRI TBSS L3 Splenium of corpus callosum	Wald ratio	0.1896874	0.05	1
	IDP dMRI TBSS L3 Inferior cerebellar peduncle R	Wald ratio	0.776730664	0.05	1
	IDP dMRI TBSS L3 Inferior cerebellar peduncle L	Wald ratio	0.776730664	0.05	1
	IDP dMRI TBSS L3 Superior cerebellar peduncle R	Inverse variance weighted	0.405313101	0.05	1
	IDP dMRI TBSS L3 Superior cerebellar peduncle L	Wald ratio	0.401085237	0.05	1
	IDP dMRI TBSS L3 Cerebral peduncle R	Wald ratio	0.573693381	0.05	1
	IDP dMRI TBSS L3 Anterior limb of internal capsule R	Wald ratio	0.764177156	0.05	1
	IDP dMRI TBSS L3 Anterior limb of internal capsule L	Wald ratio	0.730196818	0.05	1
	IDP dMRI TBSS L3 Retrolenticular part of internal capsule R	Wald ratio	0.295081901	0.05	1
	IDP dMRI TBSS L3 Anterior corona radiata R	Inverse variance weighted	0.390699207	0.05	1
	IDP dMRI TBSS L3 Anterior corona radiata L	Inverse variance weighted	0.409850341	0.05	1
	IDP dMRI TBSS L3 Posterior corona radiata R	Inverse variance weighted	0.890786466	0.05	1
	IDP dMRI TBSS L3 Posterior thalamic radiation R	Wald ratio	0.295081901	0.05	1
	IDP dMRI TBSS L3 Posterior thalamic radiation L	Wald ratio	0.295081901	0.05	1
	IDP dMRI TBSS L3 Sagittal stratum R	Wald ratio	0.295081901	0.05	1
	IDP dMRI TBSS L3 Sagittal stratum L	Wald ratio	0.295081901	0.05	1
	IDP dMRI TBSS L3 External capsule R	Wald ratio	0.776730664	0.05	1

	IDP dMRI TBSS L3 External capsule L	Wald ratio	0.776730664	0.05	1
	IDP dMRI TBSS L3 Cingulum cingulate gyrus R	Inverse variance weighted	0.403565358	0.05	1
	IDP dMRI TBSS L3 Cingulum cingulate gyrus L	Wald ratio	0.776730664	0.05	1
	IDP dMRI TBSS L3 Cingulum hippocampus R	Wald ratio	0.776730664	0.05	1
	IDP dMRI TBSS L3 Cingulum hippocampus L	Wald ratio	0.776730664	0.05	1
	IDP dMRI TBSS L3 Fornix cres+Stria terminalis R	Wald ratio	0.616327791	0.05	1
	IDP dMRI TBSS L3 Superior longitudinal fasciculus R	Wald ratio	0.295081901	0.05	1
	IDP dMRI TBSS L3 Superior longitudinal fasciculus L	Wald ratio	0.295081901	0.05	1
	IDP dMRI TBSS L3 Uncinate fasciculus R	Wald ratio	0.776730664	0.05	1
	IDP dMRI TBSS L3 Uncinate fasciculus L	Inverse variance weighted	0.275770564	0.05	1
	IDP dMRI TBSS ICVF Middle cerebellar peduncle	Wald ratio	0.295081901	0.05	1
	IDP dMRI TBSS ICVF Genu of corpus callosum	MR Egger	0.966895875	0.05	1
		Weighted median	0.344220891	0.05	1
		Inverse variance weighted	0.159485357	0.05	1
		Simple mode	0.669041078	0.05	1
		Weighted mode	0.741661802	0.05	1
	IDP dMRI TBSS ICVF Body of corpus callosum	MR Egger	0.841195498	0.05	1
		Weighted median	0.256527526	0.05	1
		Inverse variance weighted	0.229193274	0.05	1
		Simple mode	0.322615572	0.05	1

		Weighted mode	0.28632566	0.05	1
	IDP dMRI TBSS ICVF Splenium of corpus callosum	MR Egger	0.44442306	0.05	1
		Weighted median	0.752323537	0.05	1
		Inverse variance weighted	0.426714581	0.05	1
		Simple mode	0.967893673	0.05	1
		Weighted mode	0.947548366	0.05	1
	IDP dMRI TBSS ICVF Fornix	Wald ratio	0.185530566	0.05	1
	IDP dMRI TBSS ICVF Medial lemniscus R	Inverse variance weighted	0.6213313	0.05	1
	IDP dMRI TBSS ICVF Medial lemniscus L	Wald ratio	0.256551572	0.05	1
	IDP dMRI TBSS ICVF Inferior cerebellar peduncle R	Inverse variance weighted	0.836720769	0.05	1
	IDP dMRI TBSS ICVF Inferior cerebellar peduncle L	Inverse variance weighted	0.846444749	0.05	1
	IDP dMRI TBSS ICVF Superior cerebellar peduncle R	MR Egger	0.741699958	0.05	1
		Weighted median	0.871923757	0.05	1
		Inverse variance weighted	0.699566039	0.05	1
		Simple mode	0.9933842	0.05	1
		Weighted mode	0.929786505	0.05	1
	IDP dMRI TBSS ICVF Superior cerebellar peduncle L	Inverse variance weighted	0.62843818	0.05	1
	IDP dMRI TBSS ICVF Cerebral peduncle R	MR Egger	0.949804273	0.05	1
		Weighted median	0.962232918	0.05	1
		Inverse variance weighted	0.984308316	0.05	1
		Simple mode	0.972981636	0.05	1
		Weighted mode	0.962585254	0.05	1

	IDP dMRI TBSS ICVF Cerebral peduncle L	Wald ratio	0.848287155	0.05	1
	IDP dMRI TBSS ICVF Anterior limb of internal capsule R	Wald ratio	0.776730664	0.05	1
	IDP dMRI TBSS ICVF Anterior limb of internal capsule L	MR Egger	0.500953667	0.05	1
		Weighted median	0.979126497	0.05	1
		Inverse variance weighted	0.478323387	0.05	1
		Simple mode	0.931617933	0.05	1
		Weighted mode	0.964186289	0.05	1
	IDP dMRI TBSS ICVF Posterior limb of internal capsule R	MR Egger	0.752290203	0.05	1
		Weighted median	0.149460618	0.05	1
		Inverse variance weighted	0.144644176	0.05	1
		Simple mode	0.264638593	0.05	1
		Weighted mode	0.320096702	0.05	1
	IDP dMRI TBSS ICVF Posterior limb of internal capsule L	MR Egger	0.883459099	0.05	1
		Weighted median	0.69408066	0.05	1
		Inverse variance weighted	0.442998591	0.05	1
		Simple mode	0.835993393	0.05	1
		Weighted mode	0.797379143	0.05	1
	IDP dMRI TBSS ICVF Retrolenticular part of internal capsule R	MR Egger	0.81005537	0.05	1
		Weighted median	0.734535362	0.05	1
		Inverse variance weighted	0.223378326	0.05	1
		Simple mode	0.761511231	0.05	1
		Weighted mode	0.750420218	0.05	1

	IDP dMRI TBSS ICVF Retrolenticular part of internal capsule L	MR Egger	0.934070819	0.05	1
		Weighted median	0.730209355	0.05	1
		Inverse variance weighted	0.446721382	0.05	1
		Simple mode	0.829678448	0.05	1
		Weighted mode	0.808457307	0.05	1
	IDP dMRI TBSS ICVF Anterior corona radiata R	MR Egger	0.94525152	0.05	1
		Weighted median	0.622738089	0.05	1
		Inverse variance weighted	0.430932463	0.05	1
		Simple mode	0.820544122	0.05	1
		Weighted mode	0.807203375	0.05	1
	IDP dMRI TBSS ICVF Anterior corona radiata L	MR Egger	0.914417379	0.05	1
		Weighted median	0.64559528	0.05	1
		Inverse variance weighted	0.446237661	0.05	1
		Simple mode	0.837225754	0.05	1
		Weighted mode	0.807950555	0.05	1
	IDP dMRI TBSS ICVF Superior corona radiata R	MR Egger	0.981915793	0.05	1
		Weighted median	0.596635838	0.05	1
		Inverse variance weighted	0.418354122	0.05	1
		Simple mode	0.814669479	0.05	1
		Weighted mode	0.810563819	0.05	1
	IDP dMRI TBSS ICVF Superior corona radiata L	MR Egger	0.95852959	0.05	1
		Weighted median	0.603289787	0.05	1

		Inverse variance weighted	0.426468353	0.05	1
		Simple mode	0.835026438	0.05	1
		Weighted mode	0.794874871	0.05	1
	IDP dMRI TBSS ICVF Posterior corona radiata R	MR Egger	0.779038404	0.05	1
		Weighted median	0.401751704	0.05	1
		Inverse variance weighted	0.316331371	0.05	1
		Simple mode	0.387771027	0.05	1
		Weighted mode	0.699041535	0.05	1
	IDP dMRI TBSS ICVF Posterior corona radiata L	MR Egger	0.796770602	0.05	1
		Weighted median	0.403632597	0.05	1
		Inverse variance weighted	0.322164597	0.05	1
		Simple mode	0.382746268	0.05	1
		Weighted mode	0.742248011	0.05	1
	IDP dMRI TBSS ICVF Posterior thalamic radiation R	Wald ratio	0.295081901	0.05	1
	IDP dMRI TBSS ICVF Posterior thalamic radiation L	Inverse variance weighted	0.539169193	0.05	1
	IDP dMRI TBSS ICVF Sagittal stratum R	MR Egger	0.582921835	0.05	1
		Weighted median	0.965548429	0.05	1
		Inverse variance weighted	0.720766192	0.05	1
		Simple mode	0.718808454	0.05	1
		Weighted mode	0.753229395	0.05	1
	IDP dMRI TBSS ICVF Sagittal stratum L	Inverse variance weighted	0.505973578	0.05	1
	IDP dMRI TBSS ICVF External capsule R	MR Egger	0.821825406	0.05	1

		Weighted median	0.814383169	0.05	1
		Inverse variance weighted	0.812288281	0.05	1
		Simple mode	0.873281656	0.05	1
		Weighted mode	0.84009962	0.05	1
	IDP dMRI TBSS ICVF External capsule L	Inverse variance weighted	0.819178808	0.05	1
	IDP dMRI TBSS ICVF Cingulum cingulate gyrus R	Inverse variance weighted	0.459142572	0.05	1
	IDP dMRI TBSS ICVF Cingulum cingulate gyrus L	Wald ratio	0.201257064	0.05	1
	IDP dMRI TBSS ICVF Cingulum hippocampus R	MR Egger	0.796697888	0.05	1
		Weighted median	0.676911104	0.05	1
		Inverse variance weighted	0.850514068	0.05	1
		Simple mode	0.587194231	0.05	1
		Weighted mode	0.722354217	0.05	1
	IDP dMRI TBSS ICVF Cingulum hippocampus L	MR Egger	0.913266413	0.05	1
		Weighted median	0.740494239	0.05	1
		Inverse variance weighted	0.464196414	0.05	1
		Simple mode	0.825020637	0.05	1
		Weighted mode	0.793453685	0.05	1
	IDP dMRI TBSS ICVF Fornix cres+Stria terminalis R	Wald ratio	0.844691942	0.05	1
	IDP dMRI TBSS ICVF Superior longitudinal fasciculus R	Inverse variance weighted	0.352195473	0.05	1
	IDP dMRI TBSS ICVF Superior longitudinal fasciculus L	MR Egger	0.97632974	0.05	1
		Weighted median	0.540794416	0.05	1

		Inverse variance weighted	0.409681017	0.05	1
		Simple mode	0.819474794	0.05	1
		Weighted mode	0.801968789	0.05	1
	IDP dMRI TBSS ICVF Superior fronto-occipital fasciculus R	Inverse variance weighted	0.806907806	0.05	1
	IDP dMRI TBSS ICVF Uncinate fasciculus R	Wald ratio	0.776730664	0.05	1
	IDP dMRI TBSS ICVF Uncinate fasciculus L	Wald ratio	0.776730664	0.05	1
	IDP dMRI TBSS OD Pontine crossing tract	MR Egger	0.676275637	0.05	0.465572251
		Weighted median	0.438999562	0.05	1
		Inverse variance weighted	0.216464667	0.05	1
		Simple mode	0.909585581	0.05	1
		Weighted mode	0.790567331	0.05	1
	IDP dMRI TBSS OD Cerebral peduncle R	Wald ratio	0.266826698	0.05	1
	IDP dMRI TBSS OD Cerebral peduncle L	Wald ratio	0.266826698	0.05	1
	IDP dMRI TBSS OD Posterior limb of internal capsule L	Wald ratio	0.77203074	0.05	1
	IDP dMRI TBSS OD External capsule R	Inverse variance weighted	0.641976251	0.05	1
	IDP dMRI TBSS OD External capsule L	Wald ratio	0.864055856	0.05	1
	IDP dMRI TBSS OD Superior longitudinal fasciculus R	Wald ratio	0.798829824	0.05	1
	IDP dMRI TBSS ISOVF Fornix	Wald ratio	0.022064956	0.05	0.998314306
	IDP dMRI TBSS ISOVF External capsule R	Wald ratio	0.097671201	0.05	0.999997975
	IDP dMRI TBSS ISOVF Cingulum cingulate gyrus R	Wald ratio	0.935458427	0.05	1
	IDP dMRI ProbtrackX FA ar l	Wald ratio	0.776730664	0.05	1
	IDP dMRI ProbtrackX FA atr l	Wald ratio	0.776730664	0.05	1

	IDP dMRI ProbtrackX FA fmi	MR Egger	0.706710575	0.05	1
		Weighted median	0.401076581	0.05	1
		Inverse variance weighted	0.199284128	0.05	1
		Simple mode	0.749182633	0.05	1
		Weighted mode	0.740897356	0.05	1
	IDP dMRI ProbtrackX FA ifo l	Wald ratio	0.295081901	0.05	1
	IDP dMRI ProbtrackX FA ifo r	Wald ratio	0.255274738	0.05	1
	IDP dMRI ProbtrackX FA ml l	Wald ratio	0.151149261	0.05	1
	IDP dMRI ProbtrackX FA ptr r	Wald ratio	0.825471789	0.05	1
	IDP dMRI ProbtrackX FA slf l	Wald ratio	0.255274738	0.05	1
	IDP dMRI ProbtrackX FA str l	Wald ratio	0.628518624	0.05	1
	IDP dMRI ProbtrackX FA unc l	Wald ratio	0.776730664	0.05	1
	IDP dMRI ProbtrackX FA unc r	Wald ratio	0.776730664	0.05	1
	IDP dMRI ProbtrackX MD ar l	Wald ratio	0.776730664	0.05	1
	IDP dMRI ProbtrackX MD ar r	Wald ratio	0.776730664	0.05	1
	IDP dMRI ProbtrackX MD atr l	Wald ratio	0.776730664	0.05	1
	IDP dMRI ProbtrackX MD atr r	Wald ratio	0.776730664	0.05	1
	IDP dMRI ProbtrackX MD cgc l	MR Egger	0.750448998	0.05	1
		Weighted median	0.771354282	0.05	1
		Inverse variance weighted	0.894666973	0.05	1
		Simple mode	0.632648801	0.05	1
		Weighted mode	0.747066015	0.05	1
	IDP dMRI ProbtrackX MD cgc r	Inverse variance weighted	0.963304161	0.05	1

	IDP dMRI ProbtrackX MD fmi	Inverse variance weighted	0.404307448	0.05	1
	IDP dMRI ProbtrackX MD ifo l	Wald ratio	0.295081901	0.05	1
	IDP dMRI ProbtrackX MD ifo r	Wald ratio	0.295081901	0.05	1
	IDP dMRI ProbtrackX MD ilf l	Wald ratio	0.295081901	0.05	1
	IDP dMRI ProbtrackX MD ilf r	Wald ratio	0.295081901	0.05	1
	IDP dMRI ProbtrackX MD ptr l	Wald ratio	0.776730664	0.05	1
	IDP dMRI ProbtrackX MD ptr r	Wald ratio	0.776730664	0.05	1
	IDP dMRI ProbtrackX MD slf l	Inverse variance weighted	0.393663467	0.05	1
	IDP dMRI ProbtrackX MD slf r	Wald ratio	0.295081901	0.05	1
	IDP dMRI ProbtrackX MD str l	Wald ratio	0.476815487	0.05	1
	IDP dMRI ProbtrackX MD str r	Wald ratio	0.776730664	0.05	1
	IDP dMRI ProbtrackX MD unc l	Wald ratio	0.776730664	0.05	1
	IDP dMRI ProbtrackX MD unc r	Inverse variance weighted	0.495832956	0.05	1
	IDP dMRI ProbtrackX MO atr l	Wald ratio	0.71699026	0.05	1
	IDP dMRI ProbtrackX MO ml r	Wald ratio	0.008214035	0.05	1
	IDP T1 FAST ROIs L intracalc cortex	Wald ratio	0.417621381	0.05	1
	IDP dMRI ProbtrackX L1 ilf l	Wald ratio	0.776730664	0.05	1
	IDP T1 FAST ROIs R intracalc cortex	Wald ratio	0.417621381	0.05	1
	IDP dMRI ProbtrackX L1 slf l	Wald ratio	0.776730664	0.05	1
	IDP dMRI ProbtrackX L1 slf r	Wald ratio	0.862266854	0.05	1
	IDP dMRI ProbtrackX L1 str r	Wald ratio	0.500967736	0.05	1
	IDP dMRI ProbtrackX L1 unc l	Inverse variance weighted	0.692688457	0.05	1

	IDP dMRI ProbtrackX L1 unc r	Wald ratio	0.419142622	0.05	1
	IDP dMRI ProbtrackX L2 ar l	Wald ratio	0.776730664	0.05	1
	IDP dMRI ProbtrackX L2 ar r	Wald ratio	0.776730664	0.05	1
	IDP dMRI ProbtrackX L2 atr l	Wald ratio	0.776730664	0.05	1
	IDP dMRI ProbtrackX L2 atr r	Wald ratio	0.776730664	0.05	1
	IDP dMRI ProbtrackX L2 cgh l	Wald ratio	0.54451006	0.05	1
	IDP dMRI ProbtrackX L2 fma	Wald ratio	0.803712522	0.05	1
	IDP dMRI ProbtrackX L2 fmi	Inverse variance weighted	0.403338474	0.05	1
	IDP dMRI ProbtrackX L2 ifo l	Wald ratio	0.295081901	0.05	1
	IDP dMRI ProbtrackX L2 ifo r	Wald ratio	0.295081901	0.05	1
	IDP dMRI ProbtrackX L2 ilf l	Wald ratio	0.295081901	0.05	1
	IDP dMRI ProbtrackX L2 ilf r	Wald ratio	0.295081901	0.05	1
	IDP dMRI ProbtrackX L2 ptr l	Wald ratio	0.776730664	0.05	1
	IDP dMRI ProbtrackX L2 ptr r	Wald ratio	0.665654777	0.05	1
	IDP dMRI ProbtrackX L2 slf l	Inverse variance weighted	0.391023824	0.05	1
	IDP dMRI ProbtrackX L2 slf r	Wald ratio	0.295081901	0.05	1
	IDP dMRI ProbtrackX L2 str l	Wald ratio	0.476815487	0.05	1
	IDP dMRI ProbtrackX L2 str r	Wald ratio	0.476815487	0.05	1
	IDP dMRI ProbtrackX L2 unc l	Wald ratio	0.776730664	0.05	1
	IDP dMRI ProbtrackX L2 unc r	Inverse variance weighted	0.502726685	0.05	1
	IDP dMRI ProbtrackX L3 ar l	Wald ratio	0.776730664	0.05	1
	IDP dMRI ProbtrackX L3 ar r	Wald ratio	0.776730664	0.05	1
	IDP dMRI ProbtrackX L3 atr l	Wald ratio	0.776730664	0.05	1

	IDP dMRI ProbtrackX L3 atr r	Wald ratio	0.776730664	0.05	1
	IDP dMRI ProbtrackX L3 fmi	MR Egger	0.959728844	0.05	1
		Weighted median	0.636228165	0.05	1
		Inverse variance weighted	0.417235129	0.05	1
		Simple mode	0.826483594	0.05	1
		Weighted mode	0.814094711	0.05	1
	IDP dMRI ProbtrackX L3 ifo l	Inverse variance weighted	0.422041848	0.05	1
	IDP dMRI ProbtrackX L3 ifo r	Wald ratio	0.295081901	0.05	1
	IDP dMRI ProbtrackX L3 ilf l	Wald ratio	0.295081901	0.05	1
	IDP dMRI ProbtrackX L3 ilf r	Wald ratio	0.295081901	0.05	1
	IDP dMRI ProbtrackX L3 ptr l	Wald ratio	0.776730664	0.05	1
	IDP dMRI ProbtrackX L3 ptr r	Wald ratio	0.665654777	0.05	1
	IDP dMRI ProbtrackX L3 slf l	Inverse variance weighted	0.398756641	0.05	1
	IDP dMRI ProbtrackX L3 slf r	Wald ratio	0.295081901	0.05	1
	IDP dMRI ProbtrackX L3 str l	Wald ratio	0.665654777	0.05	1
	IDP dMRI ProbtrackX L3 unc l	Wald ratio	0.776730664	0.05	1
	IDP dMRI ProbtrackX L3 unc r	Inverse variance weighted	0.484753925	0.05	1
	IDP dMRI ProbtrackX ICVF ar l	MR Egger	0.796854555	0.05	1
		Weighted median	0.751481213	0.05	1
		Inverse variance weighted	0.435452511	0.05	1
		Simple mode	0.837790284	0.05	1
		Weighted mode	0.803286172	0.05	1

	IDP dMRI ProbtrackX ICVF ar r	MR Egger	0.817692151	0.05	1
		Weighted median	0.748613269	0.05	1
		Inverse variance weighted	0.420495571	0.05	1
		Simple mode	0.846609411	0.05	1
		Weighted mode	0.804226517	0.05	1
	IDP dMRI ProbtrackX ICVF atr l	MR Egger	0.878354935	0.05	1
		Weighted median	0.7408096	0.05	1
		Inverse variance weighted	0.471792539	0.05	1
		Simple mode	0.834561661	0.05	1
		Weighted mode	0.80132693	0.05	1
	IDP dMRI ProbtrackX ICVF atr r	MR Egger	0.871717963	0.05	1
		Weighted median	0.713050595	0.05	1
		Inverse variance weighted	0.467465444	0.05	1
		Simple mode	0.842180046	0.05	1
		Weighted mode	0.808797471	0.05	1
	IDP dMRI ProbtrackX ICVF cgc l	Wald ratio	0.201257064	0.05	1
	IDP dMRI ProbtrackX ICVF cgh l	MR Egger	0.870312013	0.05	1
		Weighted median	0.752445695	0.05	1
		Inverse variance weighted	0.475431909	0.05	1
		Simple mode	0.833769937	0.05	1
		Weighted mode	0.80758336	0.05	1
	IDP dMRI ProbtrackX ICVF cgh r	Wald ratio	0.776730664	0.05	1

	IDP dMRI ProbtrackX ICVF cst l	MR Egger	0.813953819	0.05	1
		Weighted median	0.708457669	0.05	1
		Inverse variance weighted	0.393445439	0.05	1
		Simple mode	0.826925699	0.05	1
		Weighted mode	0.811866948	0.05	1
	IDP dMRI ProbtrackX ICVF cst r	MR Egger	0.835568581	0.05	1
		Weighted median	0.741822992	0.05	1
		Inverse variance weighted	0.556899934	0.05	1
		Simple mode	0.877403318	0.05	1
		Weighted mode	0.806896288	0.05	1
	IDP dMRI ProbtrackX ICVF fma	Inverse variance weighted	0.09417487	0.05	1
	IDP dMRI ProbtrackX ICVF fmi	MR Egger	0.784102523	0.05	1
		Weighted median	0.371464914	0.05	1
		Inverse variance weighted	0.214297645	0.05	1
		Simple mode	0.761524686	0.05	1
		Weighted mode	0.744538203	0.05	1
	IDP dMRI ProbtrackX ICVF ifo l	Inverse variance weighted	0.44752543	0.05	1
	IDP dMRI ProbtrackX ICVF ifo r	Inverse variance weighted	0.445092065	0.05	1
	IDP dMRI ProbtrackX ICVF ilf l	Inverse variance weighted	0.437673586	0.05	1
	IDP dMRI ProbtrackX ICVF ilf r	Wald ratio	0.295081901	0.05	1
	IDP dMRI ProbtrackX ICVF mcp	Wald ratio	0.757663719	0.05	1
	IDP dMRI ProbtrackX ICVF ml l	Wald ratio	0.820151264	0.05	1

	IDP dMRI ProbtrackX ICVF ml r	Wald ratio	0.776730664	0.05	1
	IDP dMRI ProbtrackX ICVF ptr l	Wald ratio	0.295081901	0.05	1
	IDP dMRI ProbtrackX ICVF ptr r	Inverse variance weighted	0.364681324	0.05	1
	IDP dMRI ProbtrackX ICVF slf l	MR Egger	0.961106457	0.05	1
		Weighted median	0.605952469	0.05	1
		Inverse variance weighted	0.429628989	0.05	1
		Simple mode	0.823911397	0.05	1
		Weighted mode	0.789406095	0.05	1
	IDP dMRI ProbtrackX ICVF slf r	Inverse variance weighted	0.356025215	0.05	1
	IDP dMRI ProbtrackX ICVF str l	MR Egger	0.896399402	0.05	1
		Weighted median	0.674798842	0.05	1
		Inverse variance weighted	0.443105319	0.05	1
		Simple mode	0.826408696	0.05	1
		Weighted mode	0.813600372	0.05	1
	IDP dMRI ProbtrackX ICVF str r	MR Egger	0.936335194	0.05	1
		Weighted median	0.616054296	0.05	1
		Inverse variance weighted	0.432920533	0.05	1
		Simple mode	0.826625854	0.05	1
		Weighted mode	0.794404579	0.05	1
	IDP dMRI ProbtrackX ICVF unc l	Inverse variance weighted	0.753401186	0.05	1
	IDP dMRI ProbtrackX ICVF unc r	Inverse variance weighted	0.489095396	0.05	1
	IDP dMRI ProbtrackX OD atr r	Wald ratio	0.550925375	0.05	1

	IDP dMRI ProbtrackX OD ilf l	Wald ratio	0.378390121	0.05	1
	IDP dMRI ProbtrackX OD slf l	Wald ratio	0.622592559	0.05	1
	IDP dMRI ProbtrackX OD str l	Inverse variance weighted	0.251089753	0.05	1
	IDP dMRI ProbtrackX ISOVF atr r	Wald ratio	0.776730664	0.05	1
	IDP dMRI ProbtrackX ISOVF cgc l	Wald ratio	0.506851733	0.05	1
	IDP dMRI ProbtrackX ISOVF ifo r	Wald ratio	0.09719225	0.05	0.999996753
	IDP dMRI ProbtrackX ISOVF ilf l	Wald ratio	0.912465621	0.05	1
	IDP dMRI ProbtrackX ISOVF ilf r	MR Egger	0.950115454	0.05	1
		Weighted median	0.721665929	0.05	1
		Inverse variance weighted	0.701899069	0.05	1
		Simple mode	0.752133881	0.05	1
		Weighted mode	0.754990719	0.05	1
	IDP dMRI ProbtrackX ISOVF slf l	Inverse variance weighted	0.930884711	0.05	1
	IDP dMRI ProbtrackX ISOVF slf r	MR Egger	0.587666956	0.05	1
		Weighted median	0.303023085	0.05	1
		Inverse variance weighted	0.25790492	0.05	1
		Simple mode	0.385153127	0.05	1
		Weighted mode	0.385081665	0.05	1
	IDP dMRI ProbtrackX ISOVF unc l	Wald ratio	0.428096685	0.05	1
	IDP dMRI ProbtrackX ISOVF unc r	Wald ratio	0.420227647	0.05	1
	IDP T1 SIENAX brain-normalised volume	Wald ratio	0.857582043	0.05	1
Fibromyalgia related co-morbidities	IDP T1 SIENAX peripheral grey normalised volume	Wald ratio	0.647206797	0.05	1

	IDP T1 FAST ROIs R temp occ fusif cortex	Wald ratio	0.042888233	0.05	1
	IDP T1 FAST ROIs R occ pole	Inverse variance weighted	0.621920952	0.05	1
	IDP T1 FAST ROIs L thalamus	Inverse variance weighted	0.533899998	0.05	1
	IDP T1 FAST ROIs R thalamus	Wald ratio	0.28545507	0.05	1
	IDP T1 FAST ROIs L caudate	Wald ratio	0.208785286	0.05	1
	IDP T1 FAST ROIs R caudate	Wald ratio	0.208785286	0.05	1
	IDP T1 FAST ROIs L putamen	Wald ratio	0.320494872	0.05	1
	IDP T1 FAST ROIs R putamen	Wald ratio	0.320494872	0.05	1
	IDP T1 FAST ROIs L pallidum	Wald ratio	0.380001703	0.05	1
	IDP T1 FIRST left caudate volume	Wald ratio	0.175491363	0.05	1
	IDP T1 FAST ROIs L hippocampus	Inverse variance weighted	0.296336676	0.05	1
	IDP T1 FAST ROIs R hippocampus	Wald ratio	0.147752786	0.05	1
	IDP T1 FAST ROIs L ventral striatum	Wald ratio	0.320494872	0.05	1
	IDP T1 FAST ROIs R ventral striatum	Inverse variance weighted	0.431641485	0.05	1
	IDP T1 FAST ROIs L cerebellum VI	Wald ratio	0.419463162	0.05	1
	IDP T1 FAST ROIs L cerebellum crus I	MR Egger	0.61796684	0.05	1
		Weighted median	0.026564439	0.05	1
		Inverse variance weighted	0.011949903	0.05	1
		Simple mode	0.171468721	0.05	1
		Weighted mode	0.157466063	0.05	1
	IDP T1 FAST ROIs R cerebellum crus I	Inverse variance weighted	0.132744649	0.05	1
	IDP T1 FAST ROIs L cerebellum crus II	MR Egger	0.806668077	0.05	1

		Weighted median	0.452918989	0.05	1
		Inverse variance weighted	0.434146191	0.05	1
		Simple mode	0.564124392	0.05	1
		Weighted mode	0.581578029	0.05	1
	IDP T1 FAST ROIs V cerebellum crus II	Inverse variance weighted	0.537331068	0.05	1
	IDP T1 FAST ROIs R cerebellum crus II	Inverse variance weighted	0.456982829	0.05	1
	IDP T1 FIRST left putamen volume	Wald ratio	0.060045972	0.05	1
	IDP T1 FAST ROIs R cerebellum VIIb	Wald ratio	0.415947465	0.05	1
	IDP T1 FAST ROIs V cerebellum VIIIa	MR Egger	0.553030976	0.05	1
		Weighted median	0.503274227	0.05	1
		Inverse variance weighted	0.700317551	0.05	1
		Simple mode	0.498322188	0.05	1
		Weighted mode	0.468665186	0.05	1
	IDP T1 FAST ROIs L cerebellum VIIIb	Wald ratio	0.386670879	0.05	1
	IDP T1 FAST ROIs V cerebellum VIIIb	Inverse variance weighted	0.824019453	0.05	1
	IDP T1 FAST ROIs R cerebellum VIIIb	Wald ratio	0.320494872	0.05	1
	IDP T1 FAST ROIs L cerebellum IX	Inverse variance weighted	0.107941892	0.05	1
	IDP T1 FAST ROIs V cerebellum IX	Inverse variance weighted	0.135970221	0.05	1
	IDP T1 FAST ROIs R cerebellum IX	Inverse variance weighted	0.067166496	0.05	1
	IDP T1 FAST ROIs V cerebellum X	Wald ratio	0.320494872	0.05	1
	IDP T2 FLAIR BIANCA WMH volume	MR Egger	0.810765719	0.05	1

		Weighted median	0.099840805	0.05	1
		Inverse variance weighted	0.116095769	0.05	1
		Simple mode	0.260558578	0.05	1
		Weighted mode	0.249613347	0.05	1
	IDP SWI T2star left caudate	MR Egger	0.848124007	0.05	1
		Weighted median	0.756451326	0.05	1
		Inverse variance weighted	0.321611306	0.05	1
		Simple mode	0.916434607	0.05	1
		Weighted mode	0.920275341	0.05	1
	IDP SWI T2star right caudate	MR Egger	0.875077943	0.05	1
		Weighted median	0.807838595	0.05	1
		Inverse variance weighted	0.792562139	0.05	1
		Simple mode	0.85211377	0.05	1
		Weighted mode	0.847825353	0.05	1
	IDP T1 FIRST left pallidum volume	Wald ratio	0.638730382	0.05	1
	IDP SWI T2star left putamen	MR Egger	0.786009556	0.05	1
		Weighted median	0.706145888	0.05	1
		Inverse variance weighted	0.514436833	0.05	1
		Simple mode	0.835212466	0.05	1
		Weighted mode	0.780071615	0.05	1
	IDP SWI T2star right putamen	MR Egger	0.533343479	0.05	1
		Weighted median	0.487189853	0.05	1

		Inverse variance weighted	0.523080225	0.05	1
		Simple mode	0.481204403	0.05	1
		Weighted mode	0.54898282	0.05	1
	IDP SWI T2star left pallidum	MR Egger	0.751532991	0.05	1
		Weighted median	0.955598365	0.05	1
		Inverse variance weighted	0.439152218	0.05	1
		Simple mode	0.659193671	0.05	1
		Weighted mode	0.658878711	0.05	1
	IDP SWI T2star right pallidum	MR Egger	0.479661649	0.05	1
		Weighted median	0.326353569	0.05	1
		Inverse variance weighted	0.177520792	0.05	1
		Simple mode	0.447528221	0.05	1
		Weighted mode	0.440843892	0.05	1
	IDP T1 FIRST right pallidum volume	Wald ratio	0.638730382	0.05	1
	IDP dMRI TBSS FA Genu of corpus callosum	Wald ratio	0.611261108	0.05	1
	IDP dMRI TBSS FA Splenium of corpus callosum	MR Egger	0.762034354	0.05	1
		Weighted median	0.360401225	0.05	1
		Inverse variance weighted	0.159076497	0.05	1
		Simple mode	0.632331464	0.05	1
		Weighted mode	0.616263775	0.05	1
	IDP dMRI TBSS FA Corticospinal tract L	Wald ratio	0.867198075	0.05	1
	IDP dMRI TBSS FA Superior cerebellar peduncle R	Inverse variance weighted	0.043834904	0.05	1

	IDP dMRI TBSS FA Superior cerebellar peduncle L	Inverse variance weighted	0.043301233	0.05	1
	IDP dMRI TBSS FA Posterior limb of internal capsule R	Wald ratio	0.365116533	0.05	1
	IDP dMRI TBSS FA Retrolenticular part of internal capsule R	Inverse variance weighted	0.273162701	0.05	1
	IDP dMRI TBSS FA Retrolenticular part of internal capsule L	Wald ratio	0.584547668	0.05	1
	IDP dMRI TBSS FA Anterior corona radiata R	Wald ratio	0.611261108	0.05	1
	IDP dMRI TBSS FA Anterior corona radiata L	Wald ratio	0.611261108	0.05	1
	IDP dMRI TBSS FA Posterior corona radiata L	Wald ratio	0.584547668	0.05	1
	IDP dMRI TBSS FA Posterior thalamic radiation R	Inverse variance weighted	0.311034095	0.05	1
	IDP dMRI TBSS FA Posterior thalamic radiation L	Inverse variance weighted	0.323353097	0.05	1
	IDP dMRI TBSS FA Sagittal stratum R	Inverse variance weighted	0.900709919	0.05	1
	IDP dMRI TBSS FA Sagittal stratum L	Wald ratio	0.603632054	0.05	1
	IDP dMRI TBSS FA Cingulum cingulate gyrus R	Inverse variance weighted	0.295129289	0.05	1
	IDP dMRI TBSS FA Superior longitudinal fasciculus L	Inverse variance weighted	0.678909187	0.05	1
	IDP dMRI TBSS FA Uncinate fasciculus L	Wald ratio	0.610465365	0.05	1
	IDP dMRI TBSS MD Genu of corpus callosum	Wald ratio	0.611261108	0.05	1
	IDP dMRI TBSS MD Body of corpus callosum	Wald ratio	0.778574686	0.05	1
	IDP dMRI TBSS MD Splenium of corpus callosum	Inverse variance weighted	0.022572484	0.05	1
	IDP dMRI TBSS MD Corticospinal tract R	Wald ratio	0.160422524	0.05	1
	IDP dMRI TBSS MD Inferior cerebellar peduncle R	Inverse variance weighted	0.353011885	0.05	1

	IDP dMRI TBSS MD Inferior cerebellar peduncle L	Wald ratio	0.584547668	0.05	1
	IDP dMRI TBSS MD Superior cerebellar peduncle R	Wald ratio	0.050060878	0.05	1
	IDP dMRI TBSS MD Superior cerebellar peduncle L	Wald ratio	0.133988667	0.05	1
	IDP dMRI TBSS MD Anterior limb of internal capsule L	Wald ratio	0.338445965	0.05	1
	IDP dMRI TBSS MD Retrolenticular part of internal capsule R	Inverse variance weighted	0.304435798	0.05	1
	IDP T1 FIRST left caudate volume plus IDP T1 FIRST right caudate volume	Wald ratio	0.197577149	0.05	1
	IDP T1 FIRST left putamen volume plus IDP T1 FIRST right putamen volume	Wald ratio	0.040783759	0.05	1
	IDP T1 FIRST left pallidum volume plus IDP T1 FIRST right pallidum volume	Wald ratio	0.638730382	0.05	1
	IDP SWI T2star left thalamus plus IDP SWI T2star right thalamus	MR Egger	0.775997286	0.05	1
		Weighted median	0.424138614	0.05	1
		Inverse variance weighted	0.443443148	0.05	1
		Simple mode	0.433362848	0.05	1
		Weighted mode	0.428044427	0.05	1
	IDP dMRI TBSS MD Retrolenticular part of internal capsule L	Inverse variance weighted	0.312336149	0.05	1
	IDP SWI T2star left caudate plus IDP SWI T2star right caudate	MR Egger	0.906333974	0.05	1
		Weighted median	0.803887015	0.05	1
		Inverse variance weighted	0.808209293	0.05	1
		Simple mode	0.572008447	0.05	1
		Weighted mode	0.710835346	0.05	1
		MR Egger	0.846117968	0.05	1

	IDP SWI T2star left putamen plus IDP SWI T2star right putamen	Weighted median	0.70278438	0.05	1
		Inverse variance weighted	0.906688358	0.05	1
		Simple mode	0.963350949	0.05	1
		Weighted mode	0.727668534	0.05	1
	IDP SWI T2star left pallidum plus IDP SWI T2star right pallidum	MR Egger	0.70562565	0.05	1
		Weighted median	0.443105663	0.05	1
		Inverse variance weighted	0.280149848	0.05	1
		Simple mode	0.90399915	0.05	1
		Weighted mode	0.831341546	0.05	1
	volume Left-Lateral-Ventricle	MR Egger	0.922462636	0.05	1
		Weighted median	0.559668092	0.05	1
		Inverse variance weighted	0.557280102	0.05	1
		Simple mode	0.529703559	0.05	1
		Weighted mode	0.569721622	0.05	1
	volume Left-Inf-Lat-Vent	Wald ratio	0.317785654	0.05	1
	volume Left-Cerebellum-White-Matter	Inverse variance weighted	0.827135823	0.05	1
	volume Left-Cerebellum-Cortex	Inverse variance weighted	0.043944873	0.05	1
	IDP dMRI TBSS MD Anterior corona radiata R	Inverse variance weighted	0.32600237	0.05	1
	volume Left-Putamen	Wald ratio	0.48998803	0.05	1
	volume 3rd-Ventricle	Wald ratio	0.356141615	0.05	1
	volume 4th-Ventricle	Inverse variance weighted	0.751762122	0.05	1

	volume Brain-Stem	Inverse variance weighted	0.100993353	0.05	1
	volume Left-Hippocampus	Inverse variance weighted	0.855393611	0.05	1
	volume CSF	MR Egger	0.5483442	0.05	1
		Weighted median	0.93288125	0.05	1
		Inverse variance weighted	0.779739021	0.05	1
		Simple mode	0.849618334	0.05	1
		Weighted mode	0.849067868	0.05	1
	IDP dMRI TBSS MD Anterior corona radiata L	Inverse variance weighted	0.335523333	0.05	1
	volume Left-Accumbens-area	Wald ratio	0.320494872	0.05	1
	volume Right-Lateral-Ventricle	Inverse variance weighted	0.295811825	0.05	1
	volume Right-Inf-Lat-Vent	Wald ratio	0.927917656	0.05	1
	volume Right-Cerebellum-White-Matter	Wald ratio	0.236188473	0.05	1
	volume Right-Cerebellum-Cortex	MR Egger	0.32879264	0.05	1
		Weighted median	0.898851765	0.05	1
		Inverse variance weighted	0.607909121	0.05	1
		Simple mode	0.65475429	0.05	1
		Weighted mode	0.695676901	0.05	1
	IDP dMRI TBSS MD Superior corona radiata R	Inverse variance weighted	0.326179352	0.05	1
	volume Right-Hippocampus	Wald ratio	0.147752786	0.05	1
	volume Right-Amygdala	Wald ratio	0.965878491	0.05	1
	volume Right-VentralDC	Wald ratio	0.215053352	0.05	1
	volume CC Posterior	Wald ratio	0.836292354	0.05	1

	IDP dMRI TBSS MD Superior corona radiata L	Inverse variance weighted	0.327137739	0.05	1
	IDP dMRI TBSS MD Posterior corona radiata R	Inverse variance weighted	0.327099887	0.05	1
	volume BrainSegVol-to-eTIV	Wald ratio	0.607842939	0.05	1
	IDP dMRI TBSS MD Posterior corona radiata L	Inverse variance weighted	0.32730567	0.05	1
	volume rhSurfaceHoles	Wald ratio	0.359290243	0.05	1
	DKTatlas lh cuneus area	Wald ratio	0.764797534	0.05	1
	IDP dMRI TBSS MD Posterior thalamic radiation R	Inverse variance weighted	0.317495958	0.05	1
	DKTatlas lh lateraloccipital area	Wald ratio	0.764797534	0.05	1
	DKTatlas lh lingual area	Wald ratio	0.867812718	0.05	1
	IDP dMRI TBSS MD Posterior thalamic radiation L	Inverse variance weighted	0.321713372	0.05	1
	DKTatlas lh parstriangularis area	Wald ratio	0.238934218	0.05	1
	DKTatlas lh pericalcarine area	Inverse variance weighted	0.9240564	0.05	1
	DKTatlas lh postcentral area	Inverse variance weighted	0.942060155	0.05	1
	DKTatlas lh posteriorcingulate area	Wald ratio	0.837558885	0.05	1
	DKTatlas lh precentral area	Wald ratio	0.456517714	0.05	1
	DKTatlas lh precuneus area	Wald ratio	0.112433721	0.05	1
	IDP dMRI TBSS MD Sagittal stratum R	Inverse variance weighted	0.317453967	0.05	1
	DKTatlas lh superiorparietal area	Wald ratio	0.394866064	0.05	1
	DKTatlas lh superiortemporal area	Wald ratio	0.577305472	0.05	1
	DKTatlas lh supramarginal area	Wald ratio	0.285736682	0.05	1
	DKTatlas lh WhiteSurfArea area	Wald ratio	0.829713187	0.05	1

	IDP dMRI TBSS MD Sagittal stratum L	Inverse variance weighted	0.342306187	0.05	1
	a2009s lh G&S subcentral area	Wald ratio	0.456517714	0.05	1
	a2009s lh G cuneus area	Wald ratio	0.973843025	0.05	1
	a2009s lh G front inf-Opercular area	Wald ratio	0.034242065	0.05	1
	IDP dMRI TBSS MD External capsule R	Wald ratio	0.611261108	0.05	1
	IDP dMRI TBSS MD External capsule L	Inverse variance weighted	0.799786158	0.05	1
	a2009s lh G pariet inf-Supramar area	Wald ratio	0.285736682	0.05	1
	a2009s lh G parietal sup area	Wald ratio	0.400753003	0.05	1
	a2009s lh G postcentral area	Wald ratio	0.407092623	0.05	1
	a2009s lh G precentral area	Wald ratio	0.456517714	0.05	1
	a2009s lh G precuneus area	Wald ratio	0.973843025	0.05	1
	IDP dMRI TBSS MD Cingulum cingulate gyrus R	MR Egger	0.962405236	0.05	1
		Weighted median	0.524264021	0.05	1
		Inverse variance weighted	0.714382494	0.05	1
		Simple mode	0.493147605	0.05	1
		Weighted mode	0.551964586	0.05	1
	IDP dMRI TBSS MD Cingulum cingulate gyrus L	MR Egger	0.939421202	0.05	1
		Weighted median	0.515123854	0.05	1
		Inverse variance weighted	0.722180832	0.05	1
		Simple mode	0.516834718	0.05	1
		Weighted mode	0.542069793	0.05	1
	a2009s lh S calcarine area	Wald ratio	0.867812718	0.05	1

	a2009s lh S central area	Wald ratio	0.449459604	0.05	1
	a2009s lh S collat transv ant area	Wald ratio	0.474010173	0.05	1
	a2009s lh S front middle area	Wald ratio	0.238348621	0.05	1
	IDP dMRI TBSS MD Cingulum hippocampus R	Inverse variance weighted	0.340675624	0.05	1
	a2009s lh S intrapariet&P trans area	Wald ratio	0.400753003	0.05	1
	IDP dMRI TBSS MD Cingulum hippocampus L	Inverse variance weighted	0.34521114	0.05	1
	a2009s lh S subparietal area	Wald ratio	0.867812718	0.05	1
	DKTatlas rh cuneus area	Wald ratio	0.867812718	0.05	1
	DKTatlas rh lateraloccipital area	Inverse variance weighted	0.500343166	0.05	1
	DKTatlas rh lingual area	Wald ratio	0.858500087	0.05	1
	DKTatlas rh parstriangularis area	Wald ratio	0.034721139	0.05	1
	DKTatlas rh pericalcarine area	Inverse variance weighted	0.409808788	0.05	1
	IDP dMRI TBSS MD Superior longitudinal fasciculus R	Inverse variance weighted	0.303186036	0.05	1
	DKTatlas rh postcentral area	Wald ratio	0.449459604	0.05	1
	DKTatlas rh precentral area	Wald ratio	0.456517714	0.05	1
	IDP dMRI TBSS MD Superior longitudinal fasciculus L	Inverse variance weighted	0.313685102	0.05	1
	a2009s rh G&S subcentral area	Wald ratio	0.449459604	0.05	1
	a2009s rh G&S cingul-Mid-Post area	Wald ratio	0.966674013	0.05	1
	a2009s rh G cuneus area	Wald ratio	0.867812718	0.05	1
	IDP dMRI TBSS MD Superior fronto-occipital fasciculus L	Wald ratio	0.646060573	0.05	1
	a2009s rh G oc-temp med-Lingual area	Wald ratio	0.858500087	0.05	1
	a2009s rh G parietal sup area	Wald ratio	0.2941628	0.05	1

	IDP dMRI TBSS MD Uncinate fasciculus R	Inverse variance weighted	0.903495739	0.05	1
	a2009s rh G precentral area	Wald ratio	0.456517714	0.05	1
	a2009s rh G precuneus area	Wald ratio	0.189470498	0.05	1
	IDP dMRI TBSS MD Uncinate fasciculus L	Inverse variance weighted	0.890302455	0.05	1
	a2009s rh Pole occipital area	Wald ratio	0.867812718	0.05	1
	a2009s rh S calcarine area	Wald ratio	0.858500087	0.05	1
	a2009s rh S central area	Wald ratio	0.456517714	0.05	1
	a2009s rh S collat transv ant area	Wald ratio	0.474010173	0.05	1
	a2009s rh S orbital med-olfact area	Wald ratio	0.14389634	0.05	1
	IDP dMRI TBSS MO Pontine crossing tract	MR Egger	0.809423067	0.05	1
		Weighted median	0.463155216	0.05	1
		Inverse variance weighted	0.294888234	0.05	1
		Simple mode	0.668386593	0.05	1
		Weighted mode	0.674518874	0.05	1
	DKTatlas lh postcentral thickness	Wald ratio	0.449459604	0.05	1
	a2009s lh G insular short thickness	Wald ratio	0.245668702	0.05	1
	a2009s lh G postcentral thickness	Wald ratio	0.449459604	0.05	1
	IDP T1 SIENAX CSF normalised volume	Inverse variance weighted	0.982835551	0.05	1
	IDP dMRI TBSS MO Medial lemniscus R	Wald ratio	0.93506565	0.05	1
	a2009s lh S postcentral thickness	Wald ratio	0.733116463	0.05	1
	DKTatlas rh lateraloccipital thickness	Wald ratio	0.320494872	0.05	1
	DKTatlas rh parstriangularis thickness	Wald ratio	0.67859367	0.05	1

	DKTatlas rh postcentral thickness	Wald ratio	0.449459604	0.05	1
	DKTatlas rh posteriorcingulate thickness	Inverse variance weighted	0.168971248	0.05	1
	a2009s rh G&S cingul-Mid-Ant thickness	Wald ratio	0.147538755	0.05	1
	a2009s rh G cuneus thickness	Wald ratio	0.320494872	0.05	1
	a2009s rh G postcentral thickness	Wald ratio	0.398837865	0.05	1
	a2009s rh Pole occipital thickness	Wald ratio	0.320494872	0.05	1
	a2009s rh S circular insula ant thickness	Wald ratio	0.031570855	0.05	1
	a2009s rh S oc sup&transversal thickness	Wald ratio	0.124824465	0.05	1
	a2009s rh S parieto occipital thickness	Wald ratio	0.768263402	0.05	1
	IDP dMRI TBSS MO Anterior corona radiata R	Wald ratio	0.455829478	0.05	1
	IDP dMRI TBSS MO Superior corona radiata R	Wald ratio	0.149135655	0.05	1
	IDP dMRI TBSS MO Cingulum cingulate gyrus L	Inverse variance weighted	0.926658525	0.05	1
	IDP dMRI TBSS L1 Genu of corpus callosum	Wald ratio	0.603632054	0.05	1
	IDP dMRI TBSS L1 Anterior limb of internal capsule L	Wald ratio	0.338445965	0.05	1
	IDP dMRI TBSS L1 Posterior limb of internal capsule R	Wald ratio	0.345086393	0.05	1
	IDP dMRI TBSS L1 Posterior limb of internal capsule L	Wald ratio	0.930655517	0.05	1
	IDP dMRI TBSS L1 Anterior corona radiata R	Wald ratio	0.611261108	0.05	1
	IDP dMRI TBSS L1 Anterior corona radiata L	Wald ratio	0.611261108	0.05	1
	IDP dMRI TBSS L1 Posterior corona radiata R	Wald ratio	0.707426431	0.05	1
	IDP dMRI TBSS L1 Posterior corona radiata L	Wald ratio	0.611261108	0.05	1

	IDP dMRI TBSS L1 Sagittal stratum L	Wald ratio	0.611261108	0.05	1
	IDP dMRI TBSS L1 External capsule R	Wald ratio	0.611261108	0.05	1
	IDP dMRI TBSS L1 External capsule L	MR Egger	0.808099994	0.05	1
		Weighted median	0.237757169	0.05	1
		Inverse variance weighted	0.444514176	0.05	1
		Simple mode	0.390971513	0.05	1
		Weighted mode	0.421398983	0.05	1
	IDP dMRI TBSS L1 Cingulum hippocampus L	Wald ratio	0.603632054	0.05	1
	IDP dMRI TBSS L1 Fornix cres+Stria terminalis L	Wald ratio	0.962268741	0.05	1
	IDP T1 FAST ROIs L precentral gyrus	Wald ratio	0.449459604	0.05	1
	IDP dMRI TBSS L1 Superior longitudinal fasciculus R	Inverse variance weighted	0.281643916	0.05	1
	IDP dMRI TBSS L1 Uncinate fasciculus R	Wald ratio	0.584547668	0.05	1
	IDP dMRI TBSS L1 Uncinate fasciculus L	Wald ratio	0.57691435	0.05	1
	IDP dMRI TBSS L2 Pontine crossing tract	Wald ratio	0.93506565	0.05	1
	IDP dMRI TBSS L2 Genu of corpus callosum	MR Egger	0.860043476	0.05	1
		Weighted median	0.504407796	0.05	1
		Inverse variance weighted	0.197318348	0.05	1
		Simple mode	0.677254615	0.05	1
		Weighted mode	0.675699196	0.05	1
	IDP dMRI TBSS L2 Body of corpus callosum	Wald ratio	0.611261108	0.05	1
	IDP dMRI TBSS L2 Splenium of corpus callosum	MR Egger	0.806202657	0.05	1
		Weighted median	0.192653723	0.05	1

		Inverse variance weighted	0.096149563	0.05	1
		Simple mode	0.564298872	0.05	1
		Weighted mode	0.550815651	0.05	1
	IDP dMRI TBSS L2 Corticospinal tract R	Wald ratio	0.160422524	0.05	1
	IDP T1 SIENAX CSF unnormalised volume	MR Egger	0.99826388	0.05	1
		Weighted median	0.628204067	0.05	1
		Inverse variance weighted	0.599680226	0.05	1
		Simple mode	0.527512051	0.05	1
		Weighted mode	0.618659241	0.05	1
	IDP T1 FAST ROIs L temporal pole	Wald ratio	0.644416596	0.05	1
	IDP dMRI TBSS L2 Superior cerebellar peduncle R	Inverse variance weighted	0.064657067	0.05	1
	IDP dMRI TBSS L2 Superior cerebellar peduncle L	Wald ratio	0.133988667	0.05	1
	IDP dMRI TBSS L2 Posterior limb of internal capsule R	Inverse variance weighted	0.814036113	0.05	1
	IDP dMRI TBSS L2 Posterior limb of internal capsule L	Wald ratio	0.129525924	0.05	1
	IDP dMRI TBSS L2 Retrolenticular part of internal capsule R	Inverse variance weighted	0.307889675	0.05	1
	IDP dMRI TBSS L2 Retrolenticular part of internal capsule L	Wald ratio	0.584547668	0.05	1
	IDP dMRI TBSS L2 Anterior corona radiata R	Inverse variance weighted	0.327407371	0.05	1
	IDP dMRI TBSS L2 Anterior corona radiata L	Wald ratio	0.611261108	0.05	1
	IDP dMRI TBSS L2 Superior corona radiata R	MR Egger	0.934387389	0.05	1
		Weighted median	0.375129111	0.05	1

		Inverse variance weighted	0.250454363	0.05	1
		Simple mode	0.559145634	0.05	1
		Weighted mode	0.554445014	0.05	1
	IDP dMRI TBSS L2 Superior corona radiata L	Inverse variance weighted	0.309533558	0.05	1
	IDP dMRI TBSS L2 Posterior corona radiata R	Inverse variance weighted	0.310173783	0.05	1
	IDP dMRI TBSS L2 Posterior corona radiata L	Inverse variance weighted	0.313081459	0.05	1
	IDP dMRI TBSS L2 Posterior thalamic radiation R	Inverse variance weighted	0.300085498	0.05	1
	IDP dMRI TBSS L2 Posterior thalamic radiation L	Inverse variance weighted	0.317253235	0.05	1
	IDP dMRI TBSS L2 Sagittal stratum R	Inverse variance weighted	0.3053237	0.05	1
	IDP dMRI TBSS L2 Sagittal stratum L	Wald ratio	0.603632054	0.05	1
	IDP dMRI TBSS L2 Cingulum cingulate gyrus R	Wald ratio	0.611261108	0.05	1
	IDP dMRI TBSS L2 Cingulum cingulate gyrus L	Wald ratio	0.603632054	0.05	1
	IDP dMRI TBSS L2 Cingulum hippocampus R	Inverse variance weighted	0.330578567	0.05	1
	IDP dMRI TBSS L2 Cingulum hippocampus L	Wald ratio	0.611261108	0.05	1
	IDP dMRI TBSS L2 Uncinate fasciculus L	Wald ratio	0.611261108	0.05	1
	IDP dMRI TBSS L3 Middle cerebellar peduncle	Wald ratio	0.584547668	0.05	1
	IDP dMRI TBSS L3 Genu of corpus callosum	Wald ratio	0.611261108	0.05	1
	IDP dMRI TBSS L3 Body of corpus callosum	Wald ratio	0.611261108	0.05	1
	IDP dMRI TBSS L3 Splenium of corpus callosum	Inverse variance weighted	0.14975559	0.05	1

	IDP dMRI TBSS L3 Inferior cerebellar peduncle R	Wald ratio	0.611261108	0.05	1
	IDP dMRI TBSS L3 Inferior cerebellar peduncle L	Wald ratio	0.611261108	0.05	1
	IDP dMRI TBSS L3 Superior cerebellar peduncle R	Inverse variance weighted	0.06482328	0.05	1
	IDP dMRI TBSS L3 Superior cerebellar peduncle L	Wald ratio	0.133988667	0.05	1
	IDP dMRI TBSS L3 Cerebral peduncle R	Wald ratio	0.84666929	0.05	1
	IDP dMRI TBSS L3 Anterior limb of internal capsule R	Wald ratio	0.475804813	0.05	1
	IDP dMRI TBSS L3 Anterior limb of internal capsule L	Wald ratio	0.701423891	0.05	1
	IDP dMRI TBSS L3 Retrolenticular part of internal capsule R	Inverse variance weighted	0.307675594	0.05	1
	IDP dMRI TBSS L3 Retrolenticular part of internal capsule L	Wald ratio	0.584547668	0.05	1
	IDP dMRI TBSS L3 Anterior corona radiata R	Inverse variance weighted	0.326963722	0.05	1
	IDP dMRI TBSS L3 Anterior corona radiata L	Inverse variance weighted	0.337907697	0.05	1
	IDP dMRI TBSS L3 Superior corona radiata R	Wald ratio	0.603632054	0.05	1
	IDP dMRI TBSS L3 Superior corona radiata L	Wald ratio	0.603632054	0.05	1
	IDP dMRI TBSS L3 Posterior corona radiata R	Inverse variance weighted	0.800843718	0.05	1
	IDP dMRI TBSS L3 Posterior corona radiata L	Wald ratio	0.584547668	0.05	1
	IDP dMRI TBSS L3 Posterior thalamic radiation R	Inverse variance weighted	0.312903464	0.05	1
	IDP dMRI TBSS L3 Posterior thalamic radiation L	Inverse variance weighted	0.32771161	0.05	1
	IDP dMRI TBSS L3 Sagittal stratum R	Inverse variance weighted	0.326901311	0.05	1

	IDP dMRI TBSS L3 Sagittal stratum L	Inverse variance weighted	0.348863973	0.05	1
	IDP dMRI TBSS L3 External capsule R	Wald ratio	0.611261108	0.05	1
	IDP dMRI TBSS L3 External capsule L	Wald ratio	0.611261108	0.05	1
	IDP dMRI TBSS L3 Cingulum cingulate gyrus R	Inverse variance weighted	0.334254946	0.05	1
	IDP dMRI TBSS L3 Cingulum cingulate gyrus L	Wald ratio	0.611261108	0.05	1
	IDP dMRI TBSS L3 Cingulum hippocampus R	Wald ratio	0.611261108	0.05	1
	IDP dMRI TBSS L3 Cingulum hippocampus L	Wald ratio	0.611261108	0.05	1
	IDP dMRI TBSS L3 Fornix cres+Stria terminalis R	Wald ratio	0.805524475	0.05	1
	IDP dMRI TBSS L3 Superior longitudinal fasciculus R	Inverse variance weighted	0.305578539	0.05	1
	IDP dMRI TBSS L3 Superior longitudinal fasciculus L	Inverse variance weighted	0.31676795	0.05	1
	IDP dMRI TBSS L3 Uncinate fasciculus R	Wald ratio	0.611261108	0.05	1
	IDP dMRI TBSS L3 Uncinate fasciculus L	Inverse variance weighted	0.447863108	0.05	1
	IDP dMRI TBSS ICVF Middle cerebellar peduncle	Inverse variance weighted	0.330464047	0.05	1
	IDP dMRI TBSS ICVF Genu of corpus callosum	MR Egger	0.7637867	0.05	1
		Weighted median	0.280861573	0.05	1
		Inverse variance weighted	0.090258711	0.05	1
		Simple mode	0.557458304	0.05	1
		Weighted mode	0.568178245	0.05	1
	IDP dMRI TBSS ICVF Body of corpus callosum	MR Egger	0.806598522	0.05	1
		Weighted median	0.307864022	0.05	1

		Inverse variance weighted	0.174883241	0.05	1
		Simple mode	0.413070921	0.05	1
		Weighted mode	0.464286239	0.05	1
	IDP dMRI TBSS ICVF Splenium of corpus callosum	MR Egger	0.301740136	0.05	1
		Weighted median	0.466915781	0.05	1
		Inverse variance weighted	0.629906882	0.05	1
		Simple mode	0.653780915	0.05	1
		Weighted mode	0.454646862	0.05	1
	IDP dMRI TBSS ICVF Fornix	Wald ratio	0.646060573	0.05	1
	IDP dMRI TBSS ICVF Medial lemniscus R	Inverse variance weighted	0.37887786	0.05	1
	IDP dMRI TBSS ICVF Medial lemniscus L	Wald ratio	0.961743698	0.05	1
	IDP dMRI TBSS ICVF Inferior cerebellar peduncle R	MR Egger	0.644706433	0.05	1
		Weighted median	0.574592848	0.05	1
		Inverse variance weighted	0.613129081	0.05	1
		Simple mode	0.550369163	0.05	1
		Weighted mode	0.632463939	0.05	1
	IDP dMRI TBSS ICVF Inferior cerebellar peduncle L	MR Egger	0.609734475	0.05	1
		Weighted median	0.548602657	0.05	1
		Inverse variance weighted	0.613831397	0.05	1
		Simple mode	0.534916217	0.05	1
		Weighted mode	0.582651983	0.05	1
		MR Egger	0.718262334	0.05	1

	IDP dMRI TBSS ICVF Superior cerebellar peduncle R	Weighted median	0.248145224	0.05	1
		Inverse variance weighted	0.288053171	0.05	1
		Simple mode	0.462620521	0.05	1
		Weighted mode	0.366732347	0.05	1
	IDP dMRI TBSS ICVF Superior cerebellar peduncle L	Inverse variance weighted	0.40116154	0.05	1
	IDP dMRI TBSS ICVF Cerebral peduncle R	MR Egger	0.826010034	0.05	1
		Weighted median	0.264222312	0.05	1
		Inverse variance weighted	0.388051324	0.05	1
		Simple mode	0.441633093	0.05	1
		Weighted mode	0.398733754	0.05	1
	IDP dMRI TBSS ICVF Cerebral peduncle L	Inverse variance weighted	0.30757476	0.05	1
	IDP dMRI TBSS ICVF Anterior limb of internal capsule R	Wald ratio	0.611261108	0.05	1
	IDP dMRI TBSS ICVF Anterior limb of internal capsule L	MR Egger	0.733302033	0.05	1
		Weighted median	0.184405508	0.05	1
		Inverse variance weighted	0.188695662	0.05	1
		Simple mode	0.343806148	0.05	1
		Weighted mode	0.426549852	0.05	1
	IDP dMRI TBSS ICVF Posterior limb of internal capsule R	MR Egger	0.86386486	0.05	1
		Weighted median	0.324085197	0.05	1
		Inverse variance weighted	0.14104455	0.05	1
		Simple mode	0.597156616	0.05	1
		Weighted mode	0.57851736	0.05	1

	IDP dMRI TBSS ICVF Posterior limb of internal capsule L	MR Egger	0.993513897	0.05	1
		Weighted median	0.536993603	0.05	1
		Inverse variance weighted	0.329385759	0.05	1
		Simple mode	0.661821887	0.05	1
		Weighted mode	0.640895368	0.05	1
	IDP dMRI TBSS ICVF Retrolenticular part of internal capsule R	MR Egger	0.70176805	0.05	1
		Weighted median	0.457258906	0.05	1
		Inverse variance weighted	0.400794089	0.05	1
		Simple mode	0.610751212	0.05	1
		Weighted mode	0.596772445	0.05	1
	IDP dMRI TBSS ICVF Retrolenticular part of internal capsule L	MR Egger	0.956610249	0.05	1
		Weighted median	0.501528464	0.05	1
		Inverse variance weighted	0.331946021	0.05	1
		Simple mode	0.652725855	0.05	1
		Weighted mode	0.656725899	0.05	1
	IDP dMRI TBSS ICVF Anterior corona radiata R	MR Egger	0.948829642	0.05	1
		Weighted median	0.505472527	0.05	1
		Inverse variance weighted	0.322435661	0.05	1
		Simple mode	0.66151421	0.05	1
		Weighted mode	0.661198921	0.05	1
	IDP dMRI TBSS ICVF Anterior corona radiata L	MR Egger	0.970603691	0.05	1
		Weighted median	0.524686544	0.05	1

		Inverse variance weighted	0.331260687	0.05	1
		Simple mode	0.689078897	0.05	1
		Weighted mode	0.645265547	0.05	1
	IDP dMRI TBSS ICVF Superior corona radiata R	MR Egger	0.924175213	0.05	1
		Weighted median	0.495824277	0.05	1
		Inverse variance weighted	0.315441625	0.05	1
		Simple mode	0.656879599	0.05	1
		Weighted mode	0.649874239	0.05	1
	IDP dMRI TBSS ICVF Superior corona radiata L	MR Egger	0.939752263	0.05	1
		Weighted median	0.496697204	0.05	1
		Inverse variance weighted	0.319936276	0.05	1
		Simple mode	0.648673199	0.05	1
		Weighted mode	0.651479461	0.05	1
	IDP dMRI TBSS ICVF Posterior corona radiata R	MR Egger	0.931711887	0.05	1
		Weighted median	0.337995381	0.05	1
		Inverse variance weighted	0.252558931	0.05	1
		Simple mode	0.432327161	0.05	1
		Weighted mode	0.53891021	0.05	1
	IDP dMRI TBSS ICVF Posterior corona radiata L	MR Egger	0.945739886	0.05	1
		Weighted median	0.322955846	0.05	1
		Inverse variance weighted	0.255892329	0.05	1
		Simple mode	0.389178891	0.05	1

		Weighted mode	0.588873862	0.05	1
	IDP dMRI TBSS ICVF Posterior thalamic radiation R	Inverse variance weighted	0.330700125	0.05	1
	IDP dMRI TBSS ICVF Posterior thalamic radiation L	MR Egger	0.521892132	0.05	1
		Weighted median	0.459715983	0.05	1
		Inverse variance weighted	0.842976981	0.05	1
		Simple mode	0.488222206	0.05	1
		Weighted mode	0.511616045	0.05	1
	IDP dMRI TBSS ICVF Sagittal stratum R	MR Egger	0.358545078	0.05	1
		Weighted median	0.649111257	0.05	1
		Inverse variance weighted	0.949534328	0.05	1
		Simple mode	0.702401637	0.05	1
		Weighted mode	0.562403527	0.05	1
	IDP dMRI TBSS ICVF Sagittal stratum L	MR Egger	0.571791345	0.05	1
		Weighted median	0.477635326	0.05	1
		Inverse variance weighted	0.92473286	0.05	1
		Simple mode	0.433554193	0.05	1
		Weighted mode	0.528066483	0.05	1
	IDP dMRI TBSS ICVF External capsule R	MR Egger	0.81991917	0.05	1
		Weighted median	0.635901697	0.05	1
		Inverse variance weighted	0.629439136	0.05	1
		Simple mode	0.932630489	0.05	1
		Weighted mode	0.724288834	0.05	1

	IDP dMRI TBSS ICVF External capsule L	Inverse variance weighted	0.984493406	0.05	1
	IDP dMRI TBSS ICVF Cingulum cingulate gyrus R	Inverse variance weighted	0.36828802	0.05	1
	IDP dMRI TBSS ICVF Cingulum cingulate gyrus L	Inverse variance weighted	0.283059936	0.05	1
	IDP dMRI TBSS ICVF Cingulum hippocampus R	MR Egger	0.792375424	0.05	1
		Weighted median	0.53366631	0.05	1
		Inverse variance weighted	0.491715099	0.05	1
		Simple mode	0.756440414	0.05	1
		Weighted mode	0.62928063	0.05	1
	IDP dMRI TBSS ICVF Cingulum hippocampus L	MR Egger	0.971436084	0.05	1
		Weighted median	0.513214339	0.05	1
		Inverse variance weighted	0.34276888	0.05	1
		Simple mode	0.649802281	0.05	1
		Weighted mode	0.659165492	0.05	1
	IDP dMRI TBSS ICVF Fornix cres+Stria terminalis R	Inverse variance weighted	0.234521852	0.05	1
	IDP dMRI TBSS ICVF Fornix cres+Stria terminalis L	Wald ratio	0.603632054	0.05	1
	IDP dMRI TBSS ICVF Superior longitudinal fasciculus R	MR Egger	0.887391082	0.05	1
		Weighted median	0.490297826	0.05	1
		Inverse variance weighted	0.303838554	0.05	1
		Simple mode	0.655723116	0.05	1
		Weighted mode	0.641381099	0.05	1
		MR Egger	0.897571785	0.05	1

	IDP dMRI TBSS ICVF Superior longitudinal fasciculus L	Weighted median	0.461473719	0.05	1
		Inverse variance weighted	0.310904128	0.05	1
		Simple mode	0.630018969	0.05	1
		Weighted mode	0.637002859	0.05	1
	IDP dMRI TBSS ICVF Superior fronto-occipital fasciculus R	Inverse variance weighted	0.991041003	0.05	1
	IDP dMRI TBSS ICVF Superior fronto-occipital fasciculus L	Wald ratio	0.603632054	0.05	1
	IDP dMRI TBSS ICVF Uncinate fasciculus R	Wald ratio	0.611261108	0.05	1
	IDP dMRI TBSS ICVF Uncinate fasciculus L	Wald ratio	0.611261108	0.05	1
	IDP dMRI TBSS ICVF Tapetum R	Wald ratio	0.584547668	0.05	1
	IDP dMRI TBSS ICVF Tapetum L	Wald ratio	0.584547668	0.05	1
	IDP dMRI TBSS OD Pontine crossing tract	MR Egger	0.905918247	0.05	1
		Weighted median	0.689803047	0.05	1
		Inverse variance weighted	0.249596534	0.05	1
		Simple mode	0.823967952	0.05	1
		Weighted mode	0.824451476	0.05	1
	IDP dMRI TBSS OD Genu of corpus callosum	Wald ratio	0.190378304	0.05	1
	IDP dMRI TBSS OD Cerebral peduncle R	Wald ratio	0.415970001	0.05	1
	IDP dMRI TBSS OD Cerebral peduncle L	Wald ratio	0.415970001	0.05	1
	IDP dMRI TBSS OD Posterior limb of internal capsule L	Wald ratio	0.930655517	0.05	1
	IDP dMRI TBSS OD Posterior corona radiata R	Wald ratio	0.912660938	0.05	1
	IDP dMRI TBSS OD External capsule R	Inverse variance weighted	0.90051514	0.05	1

	IDP dMRI TBSS OD External capsule L	Wald ratio	0.585383998	0.05	1
	IDP dMRI TBSS OD Superior longitudinal fasciculus R	Wald ratio	0.541018598	0.05	1
	IDP dMRI TBSS ISOVF Fornix	Wald ratio	0.727898491	0.05	1
	IDP dMRI TBSS ISOVF External capsule R	Wald ratio	0.08065677	0.05	1
	IDP dMRI TBSS ISOVF Cingulum cingulate gyrus R	Inverse variance weighted	0.594284511	0.05	1
	IDP dMRI ProbtrackX FA ar l	Wald ratio	0.611261108	0.05	1
	IDP dMRI ProbtrackX FA atr l	Wald ratio	0.611261108	0.05	1
	IDP dMRI ProbtrackX FA atr r	Wald ratio	0.584547668	0.05	1
	IDP dMRI ProbtrackX FA fma	Wald ratio	0.584547668	0.05	1
	IDP dMRI ProbtrackX FA fmi	MR Egger	0.841026562	0.05	1
		Weighted median	0.338892565	0.05	1
		Inverse variance weighted	0.137068976	0.05	1
		Simple mode	0.617375304	0.05	1
		Weighted mode	0.592842638	0.05	1
	IDP dMRI ProbtrackX FA ifo l	Inverse variance weighted	0.332447105	0.05	1
	IDP dMRI ProbtrackX FA ifo r	Inverse variance weighted	0.226224771	0.05	1
	IDP dMRI ProbtrackX FA ilf l	Inverse variance weighted	0.239694672	0.05	1
	IDP dMRI ProbtrackX FA ilf r	Inverse variance weighted	0.237411945	0.05	1
	IDP dMRI ProbtrackX FA ml l	Wald ratio	0.531260546	0.05	1
	IDP dMRI ProbtrackX FA ptr l	Wald ratio	0.603632054	0.05	1
	IDP dMRI ProbtrackX FA ptr r	Wald ratio	0.615322156	0.05	1

	IDP dMRI ProbtrackX FA slf l	Inverse variance weighted	0.219538769	0.05	1
	IDP dMRI ProbtrackX FA slf r	Wald ratio	0.584547668	0.05	1
	IDP dMRI ProbtrackX FA str l	Wald ratio	0.319373043	0.05	1
	IDP dMRI ProbtrackX FA unc l	Wald ratio	0.611261108	0.05	1
	IDP dMRI ProbtrackX FA unc r	Wald ratio	0.611261108	0.05	1
	IDP dMRI ProbtrackX MD ar l	Wald ratio	0.611261108	0.05	1
	IDP dMRI ProbtrackX MD ar r	Wald ratio	0.611261108	0.05	1
	IDP dMRI ProbtrackX MD atr l	Wald ratio	0.611261108	0.05	1
	IDP dMRI ProbtrackX MD atr r	Wald ratio	0.611261108	0.05	1
	IDP dMRI ProbtrackX MD cgc l	MR Egger	0.830479143	0.05	1
		Weighted median	0.621111622	0.05	1
		Inverse variance weighted	0.745886867	0.05	1
		Simple mode	0.540066095	0.05	1
		Weighted mode	0.616125257	0.05	1
	IDP dMRI ProbtrackX MD cgc r	MR Egger	0.98029949	0.05	1
		Weighted median	0.514464591	0.05	1
		Inverse variance weighted	0.705338411	0.05	1
		Simple mode	0.499168734	0.05	1
		Weighted mode	0.534641076	0.05	1
	IDP dMRI ProbtrackX MD fmi	Inverse variance weighted	0.334683258	0.05	1
	IDP dMRI ProbtrackX MD ifo l	Inverse variance weighted	0.329945889	0.05	1
	IDP dMRI ProbtrackX MD ifo r	Inverse variance weighted	0.325721286	0.05	1

	IDP dMRI ProbtrackX MD ilf l	Inverse variance weighted	0.328943181	0.05	1
	IDP dMRI ProbtrackX MD ilf r	Inverse variance weighted	0.339710963	0.05	1
	IDP dMRI ProbtrackX MD ptr l	Wald ratio	0.611261108	0.05	1
	IDP dMRI ProbtrackX MD ptr r	Wald ratio	0.611261108	0.05	1
	IDP dMRI ProbtrackX MD slf l	Inverse variance weighted	0.328620178	0.05	1
	IDP dMRI ProbtrackX MD slf r	Inverse variance weighted	0.319926839	0.05	1
	IDP dMRI ProbtrackX MD str l	Wald ratio	0.156456101	0.05	1
	IDP dMRI ProbtrackX MD str r	Wald ratio	0.611261108	0.05	1
	IDP dMRI ProbtrackX MD unc l	Wald ratio	0.611261108	0.05	1
	IDP dMRI ProbtrackX MD unc r	Inverse variance weighted	0.346210368	0.05	1
	IDP dMRI ProbtrackX MO atr l	Wald ratio	0.408512187	0.05	1
	IDP dMRI ProbtrackX MO ml r	Wald ratio	0.808579427	0.05	1
	IDP T1 FAST ROIs L intracalc cortex	Wald ratio	0.867812718	0.05	1
	IDP dMRI ProbtrackX L1 fmi	Wald ratio	0.603632054	0.05	1
	IDP dMRI ProbtrackX L1 ifo l	Wald ratio	0.603632054	0.05	1
	IDP dMRI ProbtrackX L1 ifo r	Wald ratio	0.603632054	0.05	1
	IDP dMRI ProbtrackX L1 ilf l	Wald ratio	0.611261108	0.05	1
	IDP dMRI ProbtrackX L1 ilf r	Wald ratio	0.603632054	0.05	1
	IDP T1 FAST ROIs R intracalc cortex	Wald ratio	0.867812718	0.05	1
	IDP dMRI ProbtrackX L1 slf l	Wald ratio	0.611261108	0.05	1
	IDP dMRI ProbtrackX L1 slf r	Inverse variance weighted	0.874762122	0.05	1
	IDP dMRI ProbtrackX L1 str r	Wald ratio	0.300919008	0.05	1

	IDP dMRI ProbtrackX L1 unc l	Inverse variance weighted	0.827089629	0.05	1
	IDP dMRI ProbtrackX L1 unc r	Inverse variance weighted	0.341877712	0.05	1
	IDP dMRI ProbtrackX L2 ar l	Wald ratio	0.611261108	0.05	1
	IDP dMRI ProbtrackX L2 ar r	Wald ratio	0.611261108	0.05	1
	IDP dMRI ProbtrackX L2 atr l	Wald ratio	0.611261108	0.05	1
	IDP dMRI ProbtrackX L2 atr r	Wald ratio	0.611261108	0.05	1
	IDP dMRI ProbtrackX L2 cgc l	Wald ratio	0.603632054	0.05	1
	IDP dMRI ProbtrackX L2 cgc r	Wald ratio	0.603632054	0.05	1
	IDP dMRI ProbtrackX L2 cgh l	Wald ratio	0.758068356	0.05	1
	IDP dMRI ProbtrackX L2 fma	Wald ratio	0.648159083	0.05	1
	IDP dMRI ProbtrackX L2 fmi	Inverse variance weighted	0.334124157	0.05	1
	IDP dMRI ProbtrackX L2 ifo l	Inverse variance weighted	0.326316026	0.05	1
	IDP dMRI ProbtrackX L2 ifo r	Inverse variance weighted	0.320111072	0.05	1
	IDP dMRI ProbtrackX L2 ilf l	Inverse variance weighted	0.329919355	0.05	1
	IDP dMRI ProbtrackX L2 ilf r	Inverse variance weighted	0.336542225	0.05	1
	IDP dMRI ProbtrackX L2 ptr l	Wald ratio	0.611261108	0.05	1
	IDP dMRI ProbtrackX L2 ptr r	Wald ratio	0.587362522	0.05	1
	IDP dMRI ProbtrackX L2 slf l	Inverse variance weighted	0.327144408	0.05	1
	IDP dMRI ProbtrackX L2 slf r	Inverse variance weighted	0.314158255	0.05	1
	IDP dMRI ProbtrackX L2 str l	Wald ratio	0.156456101	0.05	1
	IDP dMRI ProbtrackX L2 str r	Wald ratio	0.156456101	0.05	1

	IDP dMRI ProbtrackX L2 unc l	Wald ratio	0.611261108	0.05	1
	IDP dMRI ProbtrackX L2 unc r	Inverse variance weighted	0.351032047	0.05	1
	IDP dMRI ProbtrackX L3 ar l	Wald ratio	0.611261108	0.05	1
	IDP dMRI ProbtrackX L3 ar r	Wald ratio	0.611261108	0.05	1
	IDP dMRI ProbtrackX L3 atr l	Wald ratio	0.611261108	0.05	1
	IDP dMRI ProbtrackX L3 atr r	Wald ratio	0.611261108	0.05	1
	IDP dMRI ProbtrackX L3 cgc l	Wald ratio	0.584547668	0.05	1
	IDP dMRI ProbtrackX L3 cgc r	Wald ratio	0.584547668	0.05	1
	IDP dMRI ProbtrackX L3 fmi	MR Egger	0.9389409	0.05	1
		Weighted median	0.479986623	0.05	1
		Inverse variance weighted	0.314985007	0.05	1
		Simple mode	0.656363122	0.05	1
		Weighted mode	0.654788369	0.05	1
	IDP dMRI ProbtrackX L3 ifo l	Inverse variance weighted	0.34514858	0.05	1
	IDP dMRI ProbtrackX L3 ifo r	Inverse variance weighted	0.333117315	0.05	1
	IDP dMRI ProbtrackX L3 ilf l	Inverse variance weighted	0.337723079	0.05	1
	IDP dMRI ProbtrackX L3 ilf r	Inverse variance weighted	0.346824513	0.05	1
	IDP dMRI ProbtrackX L3 ptr l	Wald ratio	0.611261108	0.05	1
	IDP dMRI ProbtrackX L3 ptr r	Wald ratio	0.587362522	0.05	1
	IDP dMRI ProbtrackX L3 slf l	Inverse variance weighted	0.331499505	0.05	1
	IDP dMRI ProbtrackX L3 slf r	Inverse variance weighted	0.324251403	0.05	1

	IDP dMRI ProbtrackX L3 str l	Wald ratio	0.587362522	0.05	1
	IDP dMRI ProbtrackX L3 str r	Wald ratio	0.603632054	0.05	1
	IDP dMRI ProbtrackX L3 unc l	Wald ratio	0.611261108	0.05	1
	IDP dMRI ProbtrackX L3 unc r	Inverse variance weighted	0.338726099	0.05	1
	IDP dMRI ProbtrackX ICVF ar l	MR Egger	0.829930845	0.05	1
		Weighted median	0.543178724	0.05	1
		Inverse variance weighted	0.222576013	0.05	1
		Simple mode	0.689480281	0.05	1
		Weighted mode	0.644934337	0.05	1
	IDP dMRI ProbtrackX ICVF ar r	MR Egger	0.856429649	0.05	1
		Weighted median	0.5090371	0.05	1
		Inverse variance weighted	0.210860402	0.05	1
		Simple mode	0.667432638	0.05	1
		Weighted mode	0.648152664	0.05	1
	IDP dMRI ProbtrackX ICVF atr l	MR Egger	0.997398422	0.05	1
		Weighted median	0.530729269	0.05	1
		Inverse variance weighted	0.346687782	0.05	1
		Simple mode	0.686378527	0.05	1
		Weighted mode	0.624350684	0.05	1
	IDP dMRI ProbtrackX ICVF atr r	MR Egger	0.99750331	0.05	1
		Weighted median	0.525759924	0.05	1
		Inverse variance weighted	0.343824425	0.05	1

		Simple mode	0.650845459	0.05	1
		Weighted mode	0.630341135	0.05	1
	IDP dMRI ProbtrackX ICVF cgc l	Inverse variance weighted	0.257977103	0.05	1
	IDP dMRI ProbtrackX ICVF cgc r	Wald ratio	0.603632054	0.05	1
	IDP dMRI ProbtrackX ICVF cgh l	MR Egger	0.996416382	0.05	1
		Weighted median	0.512273625	0.05	1
		Inverse variance weighted	0.348859875	0.05	1
		Simple mode	0.664463525	0.05	1
		Weighted mode	0.633570691	0.05	1
	IDP dMRI ProbtrackX ICVF cgh r	Wald ratio	0.611261108	0.05	1
	IDP dMRI ProbtrackX ICVF cst l	MR Egger	0.851683645	0.05	1
		Weighted median	0.5207834	0.05	1
		Inverse variance weighted	0.190813906	0.05	1
		Simple mode	0.702782727	0.05	1
		Weighted mode	0.646932002	0.05	1
	IDP dMRI ProbtrackX ICVF cst r	MR Egger	0.523646071	0.05	1
		Weighted median	0.530719245	0.05	1
		Inverse variance weighted	0.748179814	0.05	1
		Simple mode	0.621918527	0.05	1
		Weighted mode	0.591043515	0.05	1
	IDP dMRI ProbtrackX ICVF fma	MR Egger	0.739518919	0.05	1
		Weighted median	0.186206898	0.05	1

		Inverse variance weighted	0.128387816	0.05	1
		Simple mode	0.280245661	0.05	1
		Weighted mode	0.551065365	0.05	1
	IDP dMRI ProbtrackX ICVF fmi	MR Egger	0.905356269	0.05	1
		Weighted median	0.477885523	0.05	1
		Inverse variance weighted	0.130082811	0.05	1
		Simple mode	0.615054636	0.05	1
		Weighted mode	0.566380857	0.05	1
	IDP dMRI ProbtrackX ICVF ifo l	Inverse variance weighted	0.360877796	0.05	1
	IDP dMRI ProbtrackX ICVF ifo r	Inverse variance weighted	0.359343798	0.05	1
	IDP dMRI ProbtrackX ICVF ilf l	Inverse variance weighted	0.354707601	0.05	1
	IDP dMRI ProbtrackX ICVF ilf r	Inverse variance weighted	0.353126512	0.05	1
	IDP dMRI ProbtrackX ICVF mcp	Inverse variance weighted	0.181409833	0.05	1
	IDP dMRI ProbtrackX ICVF ml l	Wald ratio	0.57691435	0.05	1
	IDP dMRI ProbtrackX ICVF ml r	Wald ratio	0.611261108	0.05	1
	IDP dMRI ProbtrackX ICVF ptr l	Inverse variance weighted	0.357585536	0.05	1
	IDP dMRI ProbtrackX ICVF ptr r	MR Egger	0.835526769	0.05	1
		Weighted median	0.484884726	0.05	1
		Inverse variance weighted	0.936412748	0.05	1
		Simple mode	0.441651269	0.05	1
		Weighted mode	0.514778038	0.05	1

	IDP dMRI ProbtrackX ICVF slf l	MR Egger	0.938010577	0.05	1
		Weighted median	0.489607984	0.05	1
		Inverse variance weighted	0.32183304	0.05	1
		Simple mode	0.650545386	0.05	1
		Weighted mode	0.632199183	0.05	1
	IDP dMRI ProbtrackX ICVF slf r	MR Egger	0.922412422	0.05	1
		Weighted median	0.491598679	0.05	1
		Inverse variance weighted	0.315738314	0.05	1
		Simple mode	0.647056028	0.05	1
		Weighted mode	0.627962629	0.05	1
	IDP dMRI ProbtrackX ICVF str l	MR Egger	0.983803981	0.05	1
		Weighted median	0.517068573	0.05	1
		Inverse variance weighted	0.329358261	0.05	1
		Simple mode	0.683808594	0.05	1
		Weighted mode	0.647426214	0.05	1
	IDP dMRI ProbtrackX ICVF str r	MR Egger	0.955024145	0.05	1
		Weighted median	0.517142176	0.05	1
		Inverse variance weighted	0.323541608	0.05	1
		Simple mode	0.671212643	0.05	1
		Weighted mode	0.624549008	0.05	1
	IDP dMRI ProbtrackX ICVF unc l	Inverse variance weighted	0.548469052	0.05	1
	IDP dMRI ProbtrackX ICVF unc r	Inverse variance weighted	0.388010398	0.05	1

	IDP dMRI ProbtrackX OD atr r	Wald ratio	0.492486932	0.05	1
	IDP dMRI ProbtrackX OD ilf l	Wald ratio	0.334550731	0.05	1
	IDP dMRI ProbtrackX OD slf l	Wald ratio	0.964218824	0.05	1
	IDP dMRI ProbtrackX OD str l	Inverse variance weighted	0.875321218	0.05	1
	IDP dMRI ProbtrackX ISOVF atr r	Wald ratio	0.611261108	0.05	1
	IDP dMRI ProbtrackX ISOVF cgc l	Wald ratio	0.74544332	0.05	1
	IDP dMRI ProbtrackX ISOVF ifo r	Inverse variance weighted	0.740891497	0.05	1
	IDP dMRI ProbtrackX ISOVF ilf l	Wald ratio	0.615848725	0.05	1
	IDP dMRI ProbtrackX ISOVF ilf r	MR Egger	0.57039616	0.05	1
		Weighted median	0.213611925	0.05	1
		Inverse variance weighted	0.116367332	0.05	1
		Simple mode	0.392798654	0.05	1
		Weighted mode	0.382394974	0.05	1
	IDP dMRI ProbtrackX ISOVF slf l	Inverse variance weighted	0.915297858	0.05	1
	IDP dMRI ProbtrackX ISOVF slf r	MR Egger	0.399880504	0.05	1
		Weighted median	0.312117569	0.05	1
		Inverse variance weighted	0.527565471	0.05	1
		Simple mode	0.380609957	0.05	1
		Weighted mode	0.38169235	0.05	1
	IDP dMRI ProbtrackX ISOVF unc l	Wald ratio	0.353883183	0.05	1
	IDP dMRI ProbtrackX ISOVF unc r	Wald ratio	0.368310963	0.05	1
	IDP T1 SIENAX brain-normalised volume	Wald ratio	0.485766911	0.05	1

Giant cell arteritis	IDP T1 SIENAX peripheral grey normalised volume	Wald ratio	0.77466788	0.05	1
	IDP T1 FAST ROIs R temp occ fusif cortex	Wald ratio	0.217905461	0.05	0.812082601
	IDP T1 FAST ROIs R occ pole	Inverse variance weighted	0.373330509	0.05	1
	IDP T1 FAST ROIs L thalamus	Inverse variance weighted	0.155560765	0.05	0.999999983
	IDP T1 FAST ROIs R thalamus	Wald ratio	0.920698229	0.05	1
	IDP T1 FAST ROIs L putamen	Wald ratio	0.816303714	0.05	1
	IDP T1 FAST ROIs R putamen	Wald ratio	0.816303714	0.05	1
	IDP T1 FAST ROIs L pallidum	Wald ratio	0.441892426	0.05	1
	IDP T1 FIRST left caudate volume	Wald ratio	0.141735916	0.05	1
	IDP T1 FAST ROIs L hippocampus	Inverse variance weighted	0.614487301	0.05	1
	IDP T1 FAST ROIs R hippocampus	Wald ratio	0.747034212	0.05	1
	IDP T1 FAST ROIs L ventral striatum	Wald ratio	0.816303714	0.05	1
	IDP T1 FAST ROIs R ventral striatum	Inverse variance weighted	0.883030472	0.05	1
	IDP T1 FAST ROIs L cerebellum VI	Wald ratio	0.915231213	0.05	1
	IDP T1 FAST ROIs L cerebellum crus I	MR Egger	0.833289602	0.05	1
		Weighted median	0.629854941	0.05	1
		Inverse variance weighted	0.581650161	0.05	1
		Simple mode	0.79241739	0.05	1
		Weighted mode	0.697356772	0.05	1
	IDP T1 FAST ROIs R cerebellum crus I	Inverse variance weighted	0.652889926	0.05	1
	IDP T1 FAST ROIs L cerebellum crus II	MR Egger	0.495841897	0.05	0.998616119
		Weighted median	0.296574268	0.05	1

		Inverse variance weighted	0.243781723	0.05	1
		Simple mode	0.5759358	0.05	1
		Weighted mode	0.401200937	0.05	1
	IDP T1 FAST ROIs V cerebellum crus II	Inverse variance weighted	0.953761754	0.05	1
	IDP T1 FAST ROIs R cerebellum crus II	Inverse variance weighted	0.200476676	0.05	1
	IDP T1 FIRST left putamen volume	Inverse variance weighted	0.910238117	0.05	1
	IDP T1 FAST ROIs R cerebellum VIIb	Wald ratio	0.107238212	0.05	0.999960339
	IDP T1 FAST ROIs V cerebellum VIIIa	MR Egger	0.789126544	0.05	1
		Weighted median	0.957107475	0.05	1
		Inverse variance weighted	0.993127572	0.05	1
		Simple mode	0.946295527	0.05	1
		Weighted mode	0.922979416	0.05	1
	IDP T1 FAST ROIs L cerebellum VIIIb	Wald ratio	0.646490072	0.05	1
	IDP T1 FAST ROIs V cerebellum VIIIb	Inverse variance weighted	0.962156522	0.05	1
	IDP T1 FAST ROIs R cerebellum VIIIb	Wald ratio	0.816303714	0.05	1
	IDP T1 FAST ROIs L cerebellum IX	Inverse variance weighted	0.297470133	0.05	0.999999999
	IDP T1 FIRST right putamen volume	Wald ratio	0.497700944	0.05	1
	IDP T1 FAST ROIs V cerebellum IX	Inverse variance weighted	0.274929556	0.05	0.999998509
	IDP T1 FAST ROIs R cerebellum IX	Inverse variance weighted	0.395921008	0.05	0.999999996
	IDP T1 FAST ROIs V cerebellum X	Wald ratio	0.816303714	0.05	1
	IDP T2 FLAIR BIANCA WMH volume	Inverse variance weighted	0.802269854	0.05	1

	IDP SWI T2star left caudate	MR Egger	0.209565895	0.05	1
		Weighted median	0.338929989	0.05	1
		Inverse variance weighted	0.326126871	0.05	1
		Simple mode	0.483840551	0.05	1
		Weighted mode	0.519451953	0.05	1
	IDP SWI T2star right caudate	MR Egger	0.868665086	0.05	1
		Weighted median	0.442404042	0.05	1
		Inverse variance weighted	0.520282691	0.05	1
		Simple mode	0.353303537	0.05	1
		Weighted mode	0.475099197	0.05	1
	IDP T1 FIRST left pallidum volume	Wald ratio	0.615023717	0.05	1
	IDP SWI T2star left putamen	MR Egger	0.749215562	0.05	1
		Weighted median	0.297841068	0.05	1
		Inverse variance weighted	0.494688967	0.05	1
		Simple mode	0.429947444	0.05	1
		Weighted mode	0.364669684	0.05	1
	IDP SWI T2star right putamen	MR Egger	0.461168042	0.05	1
		Weighted median	0.193416187	0.05	1
		Inverse variance weighted	0.506734051	0.05	1
		Simple mode	0.385701526	0.05	1
		Weighted mode	0.360718372	0.05	1
	IDP SWI T2star left pallidum	MR Egger	0.494809627	0.05	1

		Weighted median	0.360810839	0.05	1
		Inverse variance weighted	0.377089194	0.05	1
		Simple mode	0.625296063	0.05	1
		Weighted mode	0.309894842	0.05	1
	IDP SWI T2star right pallidum	MR Egger	0.874762011	0.05	1
		Weighted median	0.329133027	0.05	1
		Inverse variance weighted	0.209300663	0.05	1
		Simple mode	0.606629006	0.05	1
		Weighted mode	0.517412268	0.05	1
	IDP T1 FIRST right pallidum volume	Wald ratio	0.615023717	0.05	1
	IDP dMRI TBSS FA Genu of corpus callosum	Wald ratio	0.648056079	0.05	1
	IDP dMRI TBSS FA Splenium of corpus callosum	Inverse variance weighted	0.23361856	0.05	1
	IDP dMRI TBSS FA Corticospinal tract L	Wald ratio	0.918398039	0.05	1
	IDP dMRI TBSS FA Superior cerebellar peduncle R	Inverse variance weighted	0.069115303	0.05	1
	IDP dMRI TBSS FA Superior cerebellar peduncle L	Inverse variance weighted	0.069584695	0.05	1
	IDP dMRI TBSS FA Posterior limb of internal capsule R	Wald ratio	0.871336277	0.05	1
	IDP dMRI TBSS FA Retrolenticular part of internal capsule R	Inverse variance weighted	0.820342263	0.05	1
	IDP dMRI TBSS FA Anterior corona radiata R	Wald ratio	0.648056079	0.05	1
	IDP dMRI TBSS FA Anterior corona radiata L	Wald ratio	0.648056079	0.05	1
	IDP dMRI TBSS FA Posterior thalamic radiation R	Wald ratio	0.314647852	0.05	1

	IDP dMRI TBSS FA Posterior thalamic radiation L	Wald ratio	0.314647852	0.05	1
	IDP dMRI TBSS FA Sagittal stratum R	Wald ratio	0.278092038	0.05	1
	IDP dMRI TBSS FA Cingulum cingulate gyrus R	Inverse variance weighted	0.752930968	0.05	1
	IDP dMRI TBSS FA Superior longitudinal fasciculus L	Inverse variance weighted	0.479133592	0.05	1
	IDP dMRI TBSS FA Uncinate fasciculus L	Wald ratio	0.303981364	0.05	1
	IDP dMRI TBSS MD Genu of corpus callosum	Wald ratio	0.648056079	0.05	1
	IDP dMRI TBSS MD Body of corpus callosum	Wald ratio	0.672909295	0.05	1
	IDP dMRI TBSS MD Splenium of corpus callosum	Inverse variance weighted	0.267314526	0.05	1
	IDP dMRI TBSS MD Corticospinal tract R	Wald ratio	0.755439234	0.05	1
	IDP dMRI TBSS MD Superior cerebellar peduncle R	Wald ratio	0.203967224	0.05	0.999997221
	IDP dMRI TBSS MD Superior cerebellar peduncle L	Wald ratio	0.153990618	0.05	0.999184534
	IDP dMRI TBSS MD Anterior limb of internal capsule L	Wald ratio	0.694912568	0.05	1
	IDP dMRI TBSS MD Retrolenticular part of internal capsule R	Wald ratio	0.314647852	0.05	1
	IDP T1 FIRST left caudate volume plus IDP T1 FIRST right caudate volume	Wald ratio	0.307766252	0.05	1
	IDP T1 FIRST left putamen volume plus IDP T1 FIRST right putamen volume	Inverse variance weighted	0.580772567	0.05	1
	IDP T1 FIRST left pallidum volume plus IDP T1 FIRST right pallidum volume	Wald ratio	0.615023717	0.05	1
	IDP SWI T2star left thalamus plus IDP SWI T2star right thalamus	MR Egger	0.462334135	0.05	1
		Weighted median	0.290119664	0.05	1
		Inverse variance weighted	0.176669356	0.05	1
		Simple mode	0.461333115	0.05	1

		Weighted mode	0.561380071	0.05	1
	IDP dMRI TBSS MD Retrolenticular part of internal capsule L	Wald ratio	0.314647852	0.05	1
	IDP SWI T2star left caudate plus IDP SWI T2star right caudate	MR Egger	0.911549959	0.05	1
		Weighted median	0.325400293	0.05	1
		Inverse variance weighted	0.302021243	0.05	1
		Simple mode	0.465838092	0.05	1
		Weighted mode	0.415680047	0.05	1
	IDP SWI T2star left putamen plus IDP SWI T2star right putamen	MR Egger	0.842232143	0.05	1
		Weighted median	0.257051337	0.05	1
		Inverse variance weighted	0.53469087	0.05	1
		Simple mode	0.471959792	0.05	1
		Weighted mode	0.383222803	0.05	1
	IDP SWI T2star left pallidum plus IDP SWI T2star right pallidum	MR Egger	0.369668201	0.05	1
		Weighted median	0.370512083	0.05	1
		Inverse variance weighted	0.309196857	0.05	1
		Simple mode	0.637822016	0.05	1
		Weighted mode	0.522145962	0.05	1
	volume Left-Lateral-Ventricle	MR Egger	0.921673013	0.05	1
		Weighted median	0.35976121	0.05	1
		Inverse variance weighted	0.912135415	0.05	1
		Simple mode	0.453517789	0.05	1
		Weighted mode	0.479096011	0.05	1

	volume Left-Cerebellum-White-Matter	Inverse variance weighted	0.575770659	0.05	1
	volume Left-Cerebellum-Cortex	Inverse variance weighted	0.825303945	0.05	1
	IDP dMRI TBSS MD Anterior corona radiata R	Inverse variance weighted	0.819203012	0.05	1
	volume Left-Putamen	Inverse variance weighted	0.645835156	0.05	1
	volume 4th-Ventricle	Inverse variance weighted	0.050134865	0.05	1
	volume Brain-Stem	Wald ratio	0.795310976	0.05	1
	volume Left-Hippocampus	Inverse variance weighted	0.8783681	0.05	1
	volume CSF	MR Egger	0.633909567	0.05	0.002542695
		Weighted median	0.691667837	0.05	1
		Inverse variance weighted	0.65970639	0.05	1
		Simple mode	0.677433631	0.05	1
		Weighted mode	0.673465666	0.05	1
	IDP dMRI TBSS MD Anterior corona radiata L	Inverse variance weighted	0.855308587	0.05	1
	volume Left-Accumbens-area	Wald ratio	0.816303714	0.05	1
	volume Right-Lateral-Ventricle	Inverse variance weighted	0.13621635	0.05	1
	volume Right-Inf-Lat-Vent	Wald ratio	0.614876391	0.05	1
	volume Right-Cerebellum-White-Matter	Wald ratio	0.822007502	0.05	1
	volume Right-Cerebellum-Cortex	MR Egger	0.965760099	0.05	1
		Weighted median	0.573882412	0.05	1
		Inverse variance weighted	0.369032855	0.05	1
		Simple mode	0.724757019	0.05	1

		Weighted mode	0.706438691	0.05	1
	IDP dMRI TBSS MD Superior corona radiata R	Inverse variance weighted	0.819921875	0.05	1
	volume Right-Putamen	Wald ratio	0.497700944	0.05	1
	volume Right-Hippocampus	Wald ratio	0.747034212	0.05	1
	volume Right-Amygdala	Wald ratio	0.46631684	0.05	0.999999997
	volume Right-VentralDC	Wald ratio	0.887019578	0.05	1
	volume CC Posterior	Wald ratio	0.070707266	0.05	1
	IDP dMRI TBSS MD Superior corona radiata L	Inverse variance weighted	0.823779732	0.05	1
	IDP dMRI TBSS MD Posterior corona radiata R	Wald ratio	0.314647852	0.05	1
	volume BrainSegVol-to-eTIV	Wald ratio	0.295462659	0.05	1
	IDP dMRI TBSS MD Posterior corona radiata L	Wald ratio	0.314647852	0.05	1
	volume rhSurfaceHoles	Wald ratio	0.800340098	0.05	1
	DKTatlas lh cuneus area	Wald ratio	0.461013206	0.05	1
	IDP dMRI TBSS MD Posterior thalamic radiation R	Wald ratio	0.314647852	0.05	1
	DKTatlas lh lateraloccipital area	Wald ratio	0.461013206	0.05	1
	DKTatlas lh lingual area	Wald ratio	0.434103601	0.05	1
	IDP dMRI TBSS MD Posterior thalamic radiation L	Wald ratio	0.314647852	0.05	1
	DKTatlas lh parstriangularis area	Wald ratio	0.585528119	0.05	1
	DKTatlas lh pericalcarine area	Inverse variance weighted	0.765967882	0.05	1
	DKTatlas lh postcentral area	Inverse variance weighted	0.914282592	0.05	1
	DKTatlas lh posteriorcingulate area	Wald ratio	0.956823522	0.05	1

	DKTatlas lh precentral area	Wald ratio	0.952004974	0.05	1
	DKTatlas lh precuneus area	Wald ratio	0.676163539	0.05	1
	IDP dMRI TBSS MD Sagittal stratum R	Wald ratio	0.314647852	0.05	1
	DKTatlas lh superiorparietal area	Wald ratio	0.710885821	0.05	1
	DKTatlas lh superiortemporal area	Wald ratio	0.920573781	0.05	1
	DKTatlas lh supramarginal area	Wald ratio	0.512950101	0.05	1
	DKTatlas lh WhiteSurfArea area	Wald ratio	0.291209388	0.05	0.985664764
	IDP dMRI TBSS MD Sagittal stratum L	Wald ratio	0.314647852	0.05	1
	a2009s lh G&S subcentral area	Wald ratio	0.952004974	0.05	1
	a2009s lh G cuneus area	Wald ratio	0.442436625	0.05	1
	a2009s lh G front inf-Opercular area	Wald ratio	0.98017882	0.05	1
	IDP dMRI TBSS MD External capsule R	Wald ratio	0.648056079	0.05	1
	IDP dMRI TBSS MD External capsule L	Inverse variance weighted	0.896058746	0.05	1
	a2009s lh G pariet inf-Supramar area	Wald ratio	0.512950101	0.05	1
	a2009s lh G parietal sup area	Wald ratio	0.711548074	0.05	1
	a2009s lh G postcentral area	Wald ratio	0.711965925	0.05	1
	a2009s lh G precentral area	Wald ratio	0.952004974	0.05	1
	a2009s lh G precuneus area	Wald ratio	0.442436625	0.05	1
	IDP dMRI TBSS MD Cingulum cingulate gyrus R	MR Egger	0.489513436	0.05	1
		Weighted median	0.822614286	0.05	1
		Inverse variance weighted	0.740103782	0.05	1
		Simple mode	0.573765541	0.05	1
		Weighted mode	0.800865997	0.05	1

	IDP dMRI TBSS MD Cingulum cingulate gyrus L	MR Egger	0.489605278	0.05	1
		Weighted median	0.829097242	0.05	1
		Inverse variance weighted	0.741061448	0.05	1
		Simple mode	0.56648559	0.05	1
		Weighted mode	0.817020537	0.05	1
	a2009s lh S calcarine area	Wald ratio	0.434103601	0.05	1
	a2009s lh S central area	Wald ratio	0.963682754	0.05	1
	a2009s lh S collat transv ant area	Wald ratio	0.043569868	0.05	0.986081765
	a2009s lh S front middle area	Wald ratio	0.22846807	0.05	1
	IDP dMRI TBSS MD Cingulum hippocampus R	Wald ratio	0.314647852	0.05	1
	a2009s lh S intrapariet&P trans area	Wald ratio	0.711548074	0.05	1
	IDP dMRI TBSS MD Cingulum hippocampus L	Wald ratio	0.314647852	0.05	1
	a2009s lh S subparietal area	Wald ratio	0.434103601	0.05	1
	DKTatlas rh cuneus area	Wald ratio	0.434103601	0.05	1
	DKTatlas rh lingual area	Wald ratio	0.434734343	0.05	1
	DKTatlas rh parstriangularis area	Wald ratio	0.582645143	0.05	1
	DKTatlas rh pericalcarine area	Inverse variance weighted	0.841144265	0.05	1
	IDP dMRI TBSS MD Superior longitudinal fasciculus R	Wald ratio	0.314647852	0.05	1
	DKTatlas rh postcentral area	Wald ratio	0.963682754	0.05	1
	DKTatlas rh precentral area	Wald ratio	0.952004974	0.05	1
	IDP dMRI TBSS MD Superior longitudinal fasciculus L	Wald ratio	0.314647852	0.05	1
	a2009s rh G&S subcentral area	Wald ratio	0.963682754	0.05	1

	a2009s rh G&S cingul-Mid-Post area	Wald ratio	0.27074377	0.05	1
	a2009s rh G cuneus area	Wald ratio	0.434103601	0.05	1
	IDP dMRI TBSS MD Superior fronto-occipital fasciculus L	Wald ratio	0.149833519	0.05	0.999204745
	a2009s rh G oc-temp med-Lingual area	Wald ratio	0.434734343	0.05	1
	a2009s rh G parietal sup area	Wald ratio	0.195551279	0.05	1
	IDP dMRI TBSS MD Uncinate fasciculus R	Wald ratio	0.709379226	0.05	1
	a2009s rh G precentral area	Wald ratio	0.952004974	0.05	1
	a2009s rh G precuneus area	Wald ratio	0.70449535	0.05	1
	IDP dMRI TBSS MD Uncinate fasciculus L	Inverse variance weighted	0.847943902	0.05	1
	a2009s rh Pole occipital area	Wald ratio	0.434103601	0.05	1
	a2009s rh S calcarine area	Wald ratio	0.434734343	0.05	1
	a2009s rh S central area	Wald ratio	0.952004974	0.05	1
	a2009s rh S collat transv ant area	Wald ratio	0.043569868	0.05	0.991677118
	a2009s rh S orbital med-olfact area	Wald ratio	0.916632065	0.05	1
	IDP dMRI TBSS MO Pontine crossing tract	MR Egger	0.979894906	0.05	1
		Weighted median	0.325421373	0.05	1
		Inverse variance weighted	0.884303615	0.05	1
		Simple mode	0.490270849	0.05	1
		Weighted mode	0.452636503	0.05	1
	DKTatlas lh postcentral thickness	Wald ratio	0.963682754	0.05	1
	a2009s lh G insular short thickness	Wald ratio	0.054576271	0.05	1
	a2009s lh G postcentral thickness	Wald ratio	0.963682754	0.05	1

	IDP T1 SIENAX CSF normalised volume	Inverse variance weighted	0.466772403	0.05	1
	IDP dMRI TBSS MO Medial lemniscus R	Wald ratio	0.407725866	0.05	1
	a2009s lh S postcentral thickness	Wald ratio	0.172088289	0.05	1
	DKTatlas rh lateraloccipital thickness	Wald ratio	0.816303714	0.05	1
	DKTatlas rh parstriangularis thickness	Wald ratio	0.711458522	0.05	1
	DKTatlas rh postcentral thickness	Wald ratio	0.963682754	0.05	1
	DKTatlas rh posteriorcingulate thickness	Inverse variance weighted	0.007888502	0.05	0.950389481
	a2009s rh G&S cingul-Mid-Ant thickness	Wald ratio	0.289412803	0.05	0.999999989
	a2009s rh G cuneus thickness	Wald ratio	0.816303714	0.05	1
	a2009s rh G postcentral thickness	Wald ratio	0.96299801	0.05	1
	a2009s rh Pole occipital thickness	Wald ratio	0.816303714	0.05	1
	a2009s rh S oc sup&transversal thickness	Wald ratio	0.705658459	0.05	1
	a2009s rh S parieto occipital thickness	Wald ratio	0.497723969	0.05	1
	IDP dMRI TBSS MO Anterior corona radiata R	Wald ratio	0.659464358	0.05	1
	IDP dMRI TBSS MO Superior corona radiata R	Wald ratio	0.475767887	0.05	1
	IDP dMRI TBSS MO Cingulum cingulate gyrus L	Inverse variance weighted	0.408002289	0.05	1
	IDP dMRI TBSS L1 Anterior limb of internal capsule L	Wald ratio	0.694912568	0.05	1
	IDP dMRI TBSS L1 Posterior limb of internal capsule R	Wald ratio	0.096210969	0.05	0.998078279
	IDP dMRI TBSS L1 Posterior limb of internal capsule L	Wald ratio	0.02642482	0.05	0.937837635
	IDP dMRI TBSS L1 Anterior corona radiata R	Wald ratio	0.648056079	0.05	1
	IDP dMRI TBSS L1 Anterior corona radiata L	Wald ratio	0.648056079	0.05	1

	IDP dMRI TBSS L1 Posterior corona radiata R	Wald ratio	0.759966912	0.05	1
	IDP dMRI TBSS L1 Posterior corona radiata L	Wald ratio	0.648056079	0.05	1
	IDP dMRI TBSS L1 Sagittal stratum L	Wald ratio	0.648056079	0.05	1
	IDP dMRI TBSS L1 External capsule R	Wald ratio	0.648056079	0.05	1
	IDP dMRI TBSS L1 External capsule L	Inverse variance weighted	0.658068726	0.05	1
	IDP dMRI TBSS L1 Fornix cres+Stria terminalis L	Wald ratio	0.512849322	0.05	1
	IDP T1 FAST ROIs L precentral gyrus	Wald ratio	0.963682754	0.05	1
	IDP dMRI TBSS L1 Superior longitudinal fasciculus R	Inverse variance weighted	0.980017673	0.05	1
	IDP dMRI TBSS L1 Uncinate fasciculus L	Wald ratio	0.541825807	0.05	1
	IDP dMRI TBSS L2 Pontine crossing tract	Wald ratio	0.407725866	0.05	1
	IDP dMRI TBSS L2 Genu of corpus callosum	MR Egger	0.824839508	0.05	1
		Weighted median	0.612433444	0.05	1
		Inverse variance weighted	0.890761838	0.05	1
		Simple mode	0.522678466	0.05	1
		Weighted mode	0.60959205	0.05	1
	IDP dMRI TBSS L2 Body of corpus callosum	Wald ratio	0.648056079	0.05	1
	IDP dMRI TBSS L2 Splenium of corpus callosum	MR Egger	0.559123369	0.05	0.019637044
		Weighted median	0.124918833	0.05	1
		Inverse variance weighted	0.126633748	0.05	1
		Simple mode	0.300722631	0.05	1
		Weighted mode	0.29050283	0.05	1

	IDP dMRI TBSS L2 Corticospinal tract R	Wald ratio	0.755439234	0.05	1
	IDP T1 SIENAX CSF unnormalised volume	MR Egger	0.949279164	0.05	1
		Weighted median	0.510385326	0.05	1
		Inverse variance weighted	0.946047442	0.05	1
		Simple mode	0.468203331	0.05	1
		Weighted mode	0.463162458	0.05	1
	IDP T1 FAST ROIs L temporal pole	Wald ratio	0.794762943	0.05	1
	IDP dMRI TBSS L2 Superior cerebellar peduncle R	Inverse variance weighted	0.068005625	0.05	1
	IDP dMRI TBSS L2 Superior cerebellar peduncle L	Wald ratio	0.153990618	0.05	1
	IDP dMRI TBSS L2 Posterior limb of internal capsule R	Inverse variance weighted	0.285578255	0.05	1
	IDP dMRI TBSS L2 Posterior limb of internal capsule L	Wald ratio	0.181351315	0.05	0.999999913
	IDP dMRI TBSS L2 Retrolenticular part of internal capsule R	Inverse variance weighted	0.730857228	0.05	1
	IDP dMRI TBSS L2 Anterior corona radiata R	Inverse variance weighted	0.824854681	0.05	1
	IDP dMRI TBSS L2 Anterior corona radiata L	Wald ratio	0.648056079	0.05	1
	IDP dMRI TBSS L2 Superior corona radiata R	Inverse variance weighted	0.372951851	0.05	1
	IDP dMRI TBSS L2 Superior corona radiata L	Wald ratio	0.314647852	0.05	1
	IDP dMRI TBSS L2 Posterior corona radiata R	Wald ratio	0.314647852	0.05	1
	IDP dMRI TBSS L2 Posterior corona radiata L	Wald ratio	0.314647852	0.05	1
	IDP dMRI TBSS L2 Posterior thalamic radiation R	Wald ratio	0.314647852	0.05	1
	IDP dMRI TBSS L2 Posterior thalamic radiation L	Wald ratio	0.314647852	0.05	1

	IDP dMRI TBSS L2 Sagittal stratum R	Wald ratio	0.314647852	0.05	1
	IDP dMRI TBSS L2 Cingulum cingulate gyrus R	Wald ratio	0.648056079	0.05	1
	IDP dMRI TBSS L2 Cingulum hippocampus R	Wald ratio	0.314647852	0.05	1
	IDP dMRI TBSS L2 Cingulum hippocampus L	Wald ratio	0.648056079	0.05	1
	IDP dMRI TBSS L2 Uncinate fasciculus L	Wald ratio	0.648056079	0.05	1
	IDP dMRI TBSS L3 Genu of corpus callosum	Wald ratio	0.648056079	0.05	1
	IDP dMRI TBSS L3 Body of corpus callosum	Wald ratio	0.648056079	0.05	1
	IDP dMRI TBSS L3 Splenium of corpus callosum	Wald ratio	0.184115102	0.05	1
	IDP dMRI TBSS L3 Inferior cerebellar peduncle R	Wald ratio	0.648056079	0.05	1
	IDP dMRI TBSS L3 Inferior cerebellar peduncle L	Wald ratio	0.648056079	0.05	1
	IDP dMRI TBSS L3 Superior cerebellar peduncle R	Inverse variance weighted	0.068197403	0.05	1
	IDP dMRI TBSS L3 Superior cerebellar peduncle L	Wald ratio	0.153990618	0.05	1
	IDP dMRI TBSS L3 Cerebral peduncle R	Wald ratio	0.945634122	0.05	1
	IDP dMRI TBSS L3 Anterior limb of internal capsule R	Wald ratio	0.6977574	0.05	1
	IDP dMRI TBSS L3 Anterior limb of internal capsule L	Wald ratio	0.760279968	0.05	1
	IDP dMRI TBSS L3 Retrolenticular part of internal capsule R	Wald ratio	0.314647852	0.05	1
	IDP dMRI TBSS L3 Anterior corona radiata R	Inverse variance weighted	0.823083567	0.05	1
	IDP dMRI TBSS L3 Anterior corona radiata L	Inverse variance weighted	0.863640931	0.05	1
	IDP dMRI TBSS L3 Posterior corona radiata R	Inverse variance weighted	0.464602521	0.05	1

	IDP dMRI TBSS L3 Posterior thalamic radiation R	Wald ratio	0.314647852	0.05	1
	IDP dMRI TBSS L3 Posterior thalamic radiation L	Wald ratio	0.314647852	0.05	1
	IDP dMRI TBSS L3 Sagittal stratum R	Wald ratio	0.314647852	0.05	1
	IDP dMRI TBSS L3 Sagittal stratum L	Wald ratio	0.314647852	0.05	1
	IDP dMRI TBSS L3 External capsule R	Wald ratio	0.648056079	0.05	1
	IDP dMRI TBSS L3 External capsule L	Wald ratio	0.648056079	0.05	1
	IDP dMRI TBSS L3 Cingulum cingulate gyrus R	Inverse variance weighted	0.850771398	0.05	1
	IDP dMRI TBSS L3 Cingulum cingulate gyrus L	Wald ratio	0.648056079	0.05	1
	IDP dMRI TBSS L3 Cingulum hippocampus R	Wald ratio	0.648056079	0.05	1
	IDP dMRI TBSS L3 Cingulum hippocampus L	Wald ratio	0.648056079	0.05	1
	IDP dMRI TBSS L3 Fornix cres+Stria terminalis R	Wald ratio	0.135349954	0.05	0.996632183
	IDP dMRI TBSS L3 Superior longitudinal fasciculus R	Wald ratio	0.314647852	0.05	1
	IDP dMRI TBSS L3 Superior longitudinal fasciculus L	Wald ratio	0.314647852	0.05	1
	IDP dMRI TBSS L3 Uncinate fasciculus R	Wald ratio	0.648056079	0.05	1
	IDP dMRI TBSS L3 Uncinate fasciculus L	Inverse variance weighted	0.327818969	0.05	1
	IDP dMRI TBSS ICVF Middle cerebellar peduncle	Wald ratio	0.314647852	0.05	1
	IDP dMRI TBSS ICVF Genu of corpus callosum	MR Egger	0.907990559	0.05	1
		Weighted median	0.893864394	0.05	1
		Inverse variance weighted	0.759072701	0.05	1
		Simple mode	0.637559913	0.05	1

		Weighted mode	0.712945998	0.05	1
	IDP dMRI TBSS ICVF Body of corpus callosum	MR Egger	0.771612781	0.05	1
		Weighted median	0.155194173	0.05	1
		Inverse variance weighted	0.488898003	0.05	1
		Simple mode	0.344732082	0.05	1
		Weighted mode	0.289371605	0.05	1
	IDP dMRI TBSS ICVF Splenium of corpus callosum	MR Egger	0.188849199	0.05	1
		Weighted median	0.969128548	0.05	1
		Inverse variance weighted	0.821259563	0.05	1
		Simple mode	0.418545793	0.05	1
		Weighted mode	0.500536	0.05	1
	IDP dMRI TBSS ICVF Fornix	Wald ratio	0.149833519	0.05	1
	IDP dMRI TBSS ICVF Medial lemniscus R	Inverse variance weighted	0.822832812	0.05	1
	IDP dMRI TBSS ICVF Medial lemniscus L	Wald ratio	0.173192266	0.05	0.999997595
	IDP dMRI TBSS ICVF Inferior cerebellar peduncle R	Inverse variance weighted	0.779229272	0.05	1
	IDP dMRI TBSS ICVF Inferior cerebellar peduncle L	Inverse variance weighted	0.769309324	0.05	1
	IDP dMRI TBSS ICVF Superior cerebellar peduncle R	MR Egger	0.706339015	0.05	1
		Weighted median	0.632469234	0.05	1
		Inverse variance weighted	0.465310281	0.05	1
		Simple mode	0.801849291	0.05	1
		Weighted mode	0.953448685	0.05	1
	IDP dMRI TBSS ICVF Superior cerebellar peduncle L	Inverse variance weighted	0.504367594	0.05	1

	IDP dMRI TBSS ICVF Cerebral peduncle R	MR Egger	0.582894717	0.05	1
		Weighted median	0.694327839	0.05	1
		Inverse variance weighted	0.576004536	0.05	1
		Simple mode	0.762672355	0.05	1
		Weighted mode	0.734271473	0.05	1
	IDP dMRI TBSS ICVF Cerebral peduncle L	Wald ratio	0.646490072	0.05	1
	IDP dMRI TBSS ICVF Anterior limb of internal capsule R	Wald ratio	0.648056079	0.05	1
	IDP dMRI TBSS ICVF Anterior limb of internal capsule L	MR Egger	0.791777603	0.05	0.9998827
		Weighted median	0.935707038	0.05	1
		Inverse variance weighted	0.450145649	0.05	1
		Simple mode	0.566573695	0.05	1
		Weighted mode	0.634159939	0.05	1
	IDP dMRI TBSS ICVF Posterior limb of internal capsule R	MR Egger	0.618367447	0.05	1
		Weighted median	0.211322639	0.05	1
		Inverse variance weighted	0.575352526	0.05	1
		Simple mode	0.382582216	0.05	1
		Weighted mode	0.375971656	0.05	1
	IDP dMRI TBSS ICVF Posterior limb of internal capsule L	MR Egger	0.897600842	0.05	1
		Weighted median	0.673107033	0.05	1
		Inverse variance weighted	0.7408987	0.05	1
		Simple mode	0.569167385	0.05	1
		Weighted mode	0.669343314	0.05	1

	IDP dMRI TBSS ICVF Retrolenticular part of internal capsule R	MR Egger	0.807357081	0.05	1
		Weighted median	0.939380854	0.05	1
		Inverse variance weighted	0.963154098	0.05	1
		Simple mode	0.495083589	0.05	1
		Weighted mode	0.814013471	0.05	1
	IDP dMRI TBSS ICVF Retrolenticular part of internal capsule L	MR Egger	0.974700468	0.05	1
		Weighted median	0.775765566	0.05	1
		Inverse variance weighted	0.781089515	0.05	1
		Simple mode	0.630034911	0.05	1
		Weighted mode	0.743784668	0.05	1
	IDP dMRI TBSS ICVF Anterior corona radiata R	MR Egger	0.991302899	0.05	1
		Weighted median	0.781519094	0.05	1
		Inverse variance weighted	0.788231734	0.05	1
		Simple mode	0.572946278	0.05	1
		Weighted mode	0.767142453	0.05	1
	IDP dMRI TBSS ICVF Anterior corona radiata L	MR Egger	0.945196635	0.05	1
		Weighted median	0.739717868	0.05	1
		Inverse variance weighted	0.763089363	0.05	1
		Simple mode	0.586673467	0.05	1
		Weighted mode	0.70016198	0.05	1
	IDP dMRI TBSS ICVF Superior corona radiata R	MR Egger	0.954855156	0.05	1
		Weighted median	0.812232983	0.05	1

		Inverse variance weighted	0.814522819	0.05	1
		Simple mode	0.607193791	0.05	1
		Weighted mode	0.788714303	0.05	1
	IDP dMRI TBSS ICVF Superior corona radiata L	MR Egger	0.989111742	0.05	1
		Weighted median	0.790703886	0.05	1
		Inverse variance weighted	0.798257315	0.05	1
		Simple mode	0.601802622	0.05	1
		Weighted mode	0.770042507	0.05	1
	IDP dMRI TBSS ICVF Posterior corona radiata R	MR Egger	0.587268497	0.05	0.999999745
		Weighted median	0.971451415	0.05	1
		Inverse variance weighted	0.822565679	0.05	1
		Simple mode	0.958815948	0.05	1
		Weighted mode	0.820809243	0.05	1
	IDP dMRI TBSS ICVF Posterior corona radiata L	MR Egger	0.611216146	0.05	1
		Weighted median	0.964121152	0.05	1
		Inverse variance weighted	0.83061684	0.05	1
		Simple mode	0.950533094	0.05	1
		Weighted mode	0.79158336	0.05	1
	IDP dMRI TBSS ICVF Posterior thalamic radiation R	Wald ratio	0.314647852	0.05	1
	IDP dMRI TBSS ICVF Posterior thalamic radiation L	Inverse variance weighted	0.909044065	0.05	1
	IDP dMRI TBSS ICVF Sagittal stratum R	MR Egger	0.342105271	0.05	1
		Weighted median	0.402632002	0.05	1

		Inverse variance weighted	0.696426193	0.05	1
		Simple mode	0.336226101	0.05	1
		Weighted mode	0.387723985	0.05	1
	IDP dMRI TBSS ICVF Sagittal stratum L	Inverse variance weighted	0.92153823	0.05	1
	IDP dMRI TBSS ICVF External capsule R	MR Egger	0.730697692	0.05	1
		Weighted median	0.786925296	0.05	1
		Inverse variance weighted	0.831040095	0.05	1
		Simple mode	0.860175662	0.05	1
		Weighted mode	0.722162567	0.05	1
	IDP dMRI TBSS ICVF External capsule L	Inverse variance weighted	0.816355247	0.05	1
	IDP dMRI TBSS ICVF Cingulum cingulate gyrus R	Inverse variance weighted	0.953334175	0.05	1
	IDP dMRI TBSS ICVF Cingulum cingulate gyrus L	Wald ratio	0.931596065	0.05	1
	IDP dMRI TBSS ICVF Cingulum hippocampus R	MR Egger	0.572765883	0.05	1
		Weighted median	0.906170267	0.05	1
		Inverse variance weighted	0.912535408	0.05	1
		Simple mode	0.866939798	0.05	1
		Weighted mode	0.755317585	0.05	1
	IDP dMRI TBSS ICVF Cingulum hippocampus L	MR Egger	0.943453915	0.05	1
		Weighted median	0.758792925	0.05	1
		Inverse variance weighted	0.761098329	0.05	1
		Simple mode	0.636774171	0.05	1

		Weighted mode	0.739464661	0.05	1
	IDP dMRI TBSS ICVF Fornix cres+Stria terminalis R	Wald ratio	0.319459756	0.05	1
	IDP dMRI TBSS ICVF Superior longitudinal fasciculus R	Inverse variance weighted	0.895595711	0.05	1
	IDP dMRI TBSS ICVF Superior longitudinal fasciculus L	MR Egger	0.893906091	0.05	1
		Weighted median	0.881605702	0.05	1
		Inverse variance weighted	0.848773991	0.05	1
		Simple mode	0.690035318	0.05	1
		Weighted mode	0.85339476	0.05	1
	IDP dMRI TBSS ICVF Superior fronto-occipital fasciculus R	Inverse variance weighted	0.912032286	0.05	1
	IDP dMRI TBSS ICVF Uncinate fasciculus R	Wald ratio	0.648056079	0.05	1
	IDP dMRI TBSS ICVF Uncinate fasciculus L	Wald ratio	0.648056079	0.05	1
	IDP dMRI TBSS OD Pontine crossing tract	MR Egger	0.428462666	0.05	0.012549233
		Weighted median	0.153878147	0.05	1
		Inverse variance weighted	0.171415152	0.05	1
		Simple mode	0.466966038	0.05	1
		Weighted mode	0.323545044	0.05	1
	IDP dMRI TBSS OD Cerebral peduncle R	Wald ratio	0.934531885	0.05	1
	IDP dMRI TBSS OD Cerebral peduncle L	Wald ratio	0.934531885	0.05	1
	IDP dMRI TBSS OD Posterior limb of internal capsule L	Wald ratio	0.02642482	0.05	1
	IDP dMRI TBSS OD External capsule R	Inverse variance weighted	0.922493991	0.05	1
	IDP dMRI TBSS OD External capsule L	Wald ratio	0.817138637	0.05	1

	IDP dMRI TBSS OD Superior longitudinal fasciculus R	Wald ratio	0.997390407	0.05	1
	IDP dMRI TBSS ISOVF Fornix	Wald ratio	0.218362642	0.05	0.999999997
	IDP dMRI TBSS ISOVF External capsule R	Wald ratio	0.317975719	0.05	1
	IDP dMRI TBSS ISOVF Cingulum cingulate gyrus R	Wald ratio	0.421121194	0.05	1
	IDP dMRI ProbtrackX FA ar l	Wald ratio	0.648056079	0.05	1
	IDP dMRI ProbtrackX FA atr l	Wald ratio	0.648056079	0.05	1
	IDP dMRI ProbtrackX FA fmi	MR Egger	0.649449951	0.05	0.999999638
		Weighted median	0.858748484	0.05	1
		Inverse variance weighted	0.831137544	0.05	1
		Simple mode	0.381733045	0.05	1
		Weighted mode	0.738742732	0.05	1
	IDP dMRI ProbtrackX FA ifo l	Wald ratio	0.314647852	0.05	1
	IDP dMRI ProbtrackX FA ifo r	Wald ratio	0.346198297	0.05	1
	IDP dMRI ProbtrackX FA ml l	Wald ratio	0.920579444	0.05	1
	IDP dMRI ProbtrackX FA ptr r	Wald ratio	0.544529189	0.05	1
	IDP dMRI ProbtrackX FA slf l	Wald ratio	0.346198297	0.05	1
	IDP dMRI ProbtrackX FA str l	Wald ratio	0.797381352	0.05	1
	IDP dMRI ProbtrackX FA unc l	Wald ratio	0.648056079	0.05	1
	IDP dMRI ProbtrackX FA unc r	Wald ratio	0.648056079	0.05	1
	IDP dMRI ProbtrackX MD ar l	Wald ratio	0.648056079	0.05	1
	IDP dMRI ProbtrackX MD ar r	Wald ratio	0.648056079	0.05	1
	IDP dMRI ProbtrackX MD atr l	Wald ratio	0.648056079	0.05	1
	IDP dMRI ProbtrackX MD atr r	Wald ratio	0.648056079	0.05	1

	IDP dMRI ProbtrackX MD cgc l	MR Egger	0.495045387	0.05	1
		Weighted median	0.884550312	0.05	1
		Inverse variance weighted	0.765056525	0.05	1
		Simple mode	0.626149937	0.05	1
		Weighted mode	0.81807673	0.05	1
	IDP dMRI ProbtrackX MD cgc r	Inverse variance weighted	0.316556172	0.05	1
	IDP dMRI ProbtrackX MD fmi	Inverse variance weighted	0.852311956	0.05	1
	IDP dMRI ProbtrackX MD ifo l	Wald ratio	0.314647852	0.05	1
	IDP dMRI ProbtrackX MD ifo r	Wald ratio	0.314647852	0.05	1
	IDP dMRI ProbtrackX MD ilf l	Wald ratio	0.314647852	0.05	1
	IDP dMRI ProbtrackX MD ilf r	Wald ratio	0.314647852	0.05	1
	IDP dMRI ProbtrackX MD ptr l	Wald ratio	0.648056079	0.05	1
	IDP dMRI ProbtrackX MD ptr r	Wald ratio	0.648056079	0.05	1
	IDP dMRI ProbtrackX MD slf l	Inverse variance weighted	0.829635084	0.05	1
	IDP dMRI ProbtrackX MD slf r	Wald ratio	0.314647852	0.05	1
	IDP dMRI ProbtrackX MD str l	Wald ratio	0.593075918	0.05	1
	IDP dMRI ProbtrackX MD str r	Wald ratio	0.648056079	0.05	1
	IDP dMRI ProbtrackX MD unc l	Wald ratio	0.648056079	0.05	1
	IDP dMRI ProbtrackX MD unc r	Inverse variance weighted	0.945650206	0.05	1
	IDP dMRI ProbtrackX MO atr l	Wald ratio	0.649770073	0.05	1
	IDP dMRI ProbtrackX MO ml r	Wald ratio	0.853709998	0.05	1
	IDP T1 FAST ROIs L intracalc cortex	Wald ratio	0.434103601	0.05	1

	IDP dMRI ProbtrackX L1 ilf l	Wald ratio	0.648056079	0.05	1
	IDP T1 FAST ROIs R intracalc cortex	Wald ratio	0.434103601	0.05	1
	IDP dMRI ProbtrackX L1 slf l	Wald ratio	0.648056079	0.05	1
	IDP dMRI ProbtrackX L1 slf r	Wald ratio	0.437309712	0.05	0.999999507
	IDP dMRI ProbtrackX L1 str r	Wald ratio	0.801138408	0.05	1
	IDP dMRI ProbtrackX L1 unc l	Inverse variance weighted	0.881341113	0.05	1
	IDP dMRI ProbtrackX L1 unc r	Wald ratio	0.413253375	0.05	1
	IDP dMRI ProbtrackX L2 ar l	Wald ratio	0.648056079	0.05	1
	IDP dMRI ProbtrackX L2 ar r	Wald ratio	0.648056079	0.05	1
	IDP dMRI ProbtrackX L2 atr l	Wald ratio	0.648056079	0.05	1
	IDP dMRI ProbtrackX L2 atr r	Wald ratio	0.648056079	0.05	1
	IDP dMRI ProbtrackX L2 cgh l	Wald ratio	0.27145453	0.05	1
	IDP dMRI ProbtrackX L2 fma	Wald ratio	0.346594526	0.05	0.999999999
	IDP dMRI ProbtrackX L2 fmi	Inverse variance weighted	0.850299236	0.05	1
	IDP dMRI ProbtrackX L2 ifo l	Wald ratio	0.314647852	0.05	1
	IDP dMRI ProbtrackX L2 ifo r	Wald ratio	0.314647852	0.05	1
	IDP dMRI ProbtrackX L2 ilf l	Wald ratio	0.314647852	0.05	1
	IDP dMRI ProbtrackX L2 ilf r	Wald ratio	0.314647852	0.05	1
	IDP dMRI ProbtrackX L2 ptr l	Wald ratio	0.648056079	0.05	1
	IDP dMRI ProbtrackX L2 ptr r	Wald ratio	0.645648623	0.05	1
	IDP dMRI ProbtrackX L2 slf l	Inverse variance weighted	0.823806374	0.05	1
	IDP dMRI ProbtrackX L2 slf r	Wald ratio	0.314647852	0.05	1
	IDP dMRI ProbtrackX L2 str l	Wald ratio	0.593075918	0.05	1

	IDP dMRI ProbtrackX L2 str r	Wald ratio	0.593075918	0.05	1
	IDP dMRI ProbtrackX L2 unc l	Wald ratio	0.648056079	0.05	1
	IDP dMRI ProbtrackX L2 unc r	Inverse variance weighted	0.961074387	0.05	1
	IDP dMRI ProbtrackX L3 ar l	Wald ratio	0.648056079	0.05	1
	IDP dMRI ProbtrackX L3 ar r	Wald ratio	0.648056079	0.05	1
	IDP dMRI ProbtrackX L3 atr l	Wald ratio	0.648056079	0.05	1
	IDP dMRI ProbtrackX L3 atr r	Wald ratio	0.648056079	0.05	1
	IDP dMRI ProbtrackX L3 fmi	MR Egger	0.987348655	0.05	1
		Weighted median	0.762000405	0.05	1
		Inverse variance weighted	0.794909748	0.05	1
		Simple mode	0.549202854	0.05	1
		Weighted mode	0.72487627	0.05	1
	IDP dMRI ProbtrackX L3 ifo l	Inverse variance weighted	0.887539974	0.05	1
	IDP dMRI ProbtrackX L3 ifo r	Wald ratio	0.314647852	0.05	1
	IDP dMRI ProbtrackX L3 ilf l	Wald ratio	0.314647852	0.05	1
	IDP dMRI ProbtrackX L3 ilf r	Wald ratio	0.314647852	0.05	1
	IDP dMRI ProbtrackX L3 ptr l	Wald ratio	0.648056079	0.05	1
	IDP dMRI ProbtrackX L3 ptr r	Wald ratio	0.645648623	0.05	1
	IDP dMRI ProbtrackX L3 slf l	Inverse variance weighted	0.840645217	0.05	1
	IDP dMRI ProbtrackX L3 slf r	Wald ratio	0.314647852	0.05	1
	IDP dMRI ProbtrackX L3 str l	Wald ratio	0.645648623	0.05	1
	IDP dMRI ProbtrackX L3 unc l	Wald ratio	0.648056079	0.05	1

	IDP dMRI ProbtrackX L3 unc r	Inverse variance weighted	0.919771937	0.05	1
	IDP dMRI ProbtrackX ICVF ar l	MR Egger	0.995093844	0.05	1
		Weighted median	0.608168403	0.05	1
		Inverse variance weighted	0.451016338	0.05	1
		Simple mode	0.920679715	0.05	1
		Weighted mode	0.739361879	0.05	1
	IDP dMRI ProbtrackX ICVF ar r	MR Egger	0.980839022	0.05	1
		Weighted median	0.62134118	0.05	1
		Inverse variance weighted	0.456818646	0.05	1
		Simple mode	0.914747844	0.05	1
		Weighted mode	0.753801826	0.05	1
	IDP dMRI ProbtrackX ICVF atr l	MR Egger	0.889587971	0.05	1
		Weighted median	0.674949368	0.05	1
		Inverse variance weighted	0.725853804	0.05	1
		Simple mode	0.561718811	0.05	1
		Weighted mode	0.695600708	0.05	1
	IDP dMRI ProbtrackX ICVF atr r	MR Egger	0.879086568	0.05	1
		Weighted median	0.666854233	0.05	1
		Inverse variance weighted	0.721639202	0.05	1
		Simple mode	0.584257643	0.05	1
		Weighted mode	0.663110283	0.05	1
	IDP dMRI ProbtrackX ICVF cgc l	Wald ratio	0.931596065	0.05	1

	IDP dMRI ProbtrackX ICVF cgh l	MR Egger	0.876849477	0.05	1
		Weighted median	0.65436754	0.05	1
		Inverse variance weighted	0.717272424	0.05	1
		Simple mode	0.574491461	0.05	1
		Weighted mode	0.683101928	0.05	1
	IDP dMRI ProbtrackX ICVF cgh r	Wald ratio	0.648056079	0.05	1
	IDP dMRI ProbtrackX ICVF cst l	MR Egger	0.985086998	0.05	1
		Weighted median	0.6256726	0.05	1
		Inverse variance weighted	0.440375232	0.05	1
		Simple mode	0.892512732	0.05	1
		Weighted mode	0.767001029	0.05	1
	IDP dMRI ProbtrackX ICVF cst r	MR Egger	0.765607775	0.05	1
		Weighted median	0.422351303	0.05	1
		Inverse variance weighted	0.439236475	0.05	1
		Simple mode	0.275821935	0.05	1
		Weighted mode	0.607056662	0.05	1
	IDP dMRI ProbtrackX ICVF fma	Inverse variance weighted	0.097336114	0.05	1
	IDP dMRI ProbtrackX ICVF fmi	MR Egger	0.765068406	0.05	1
		Weighted median	0.784757143	0.05	1
		Inverse variance weighted	0.821072613	0.05	1
		Simple mode	0.379112265	0.05	1
		Weighted mode	0.843442867	0.05	1

	IDP dMRI ProbtrackX ICVF ifo l	Inverse variance weighted	0.933699882	0.05	1
	IDP dMRI ProbtrackX ICVF ifo r	Inverse variance weighted	0.929484919	0.05	1
	IDP dMRI ProbtrackX ICVF ilf l	Inverse variance weighted	0.916398692	0.05	1
	IDP dMRI ProbtrackX ICVF ilf r	Wald ratio	0.314647852	0.05	1
	IDP dMRI ProbtrackX ICVF mcp	Wald ratio	0.141613615	0.05	1
	IDP dMRI ProbtrackX ICVF ml l	Wald ratio	0.541825807	0.05	1
	IDP dMRI ProbtrackX ICVF ml r	Wald ratio	0.648056079	0.05	1
	IDP dMRI ProbtrackX ICVF ptr l	Wald ratio	0.314647852	0.05	1
	IDP dMRI ProbtrackX ICVF ptr r	Inverse variance weighted	0.175528485	0.05	1
	IDP dMRI ProbtrackX ICVF slf l	MR Egger	0.985324398	0.05	1
		Weighted median	0.816789625	0.05	1
		Inverse variance weighted	0.80213679	0.05	1
		Simple mode	0.619986826	0.05	1
		Weighted mode	0.770434211	0.05	1
	IDP dMRI ProbtrackX ICVF slf r	Inverse variance weighted	0.907307207	0.05	1
	IDP dMRI ProbtrackX ICVF str l	MR Egger	0.917690613	0.05	1
		Weighted median	0.695520452	0.05	1
		Inverse variance weighted	0.749644762	0.05	1
		Simple mode	0.561070561	0.05	1
		Weighted mode	0.677982177	0.05	1
	IDP dMRI ProbtrackX ICVF str r	MR Egger	0.978072282	0.05	1
		Weighted median	0.754961711	0.05	1

		Inverse variance weighted	0.781138856	0.05	1
		Simple mode	0.577576139	0.05	1
		Weighted mode	0.739972834	0.05	1
	IDP dMRI ProbtrackX ICVF unc l	Inverse variance weighted	0.39261959	0.05	1
	IDP dMRI ProbtrackX ICVF unc r	Inverse variance weighted	0.999255914	0.05	1
	IDP dMRI ProbtrackX OD atr r	Wald ratio	0.891425428	0.05	1
	IDP dMRI ProbtrackX OD ilf l	Wald ratio	0.48383771	0.05	1
	IDP dMRI ProbtrackX OD slf l	Wald ratio	0.234746448	0.05	0.990002564
	IDP dMRI ProbtrackX OD str l	Inverse variance weighted	0.498739569	0.05	1
	IDP dMRI ProbtrackX ISOVF atr r	Wald ratio	0.648056079	0.05	1
	IDP dMRI ProbtrackX ISOVF cgc l	Wald ratio	0.830015694	0.05	1
	IDP dMRI ProbtrackX ISOVF ifo r	Wald ratio	0.768975044	0.05	1
	IDP dMRI ProbtrackX ISOVF ilf l	Wald ratio	0.214778331	0.05	1
	IDP dMRI ProbtrackX ISOVF ilf r	MR Egger	0.491861343	0.05	1
		Weighted median	0.720373968	0.05	1
		Inverse variance weighted	0.875917063	0.05	1
		Simple mode	0.670237674	0.05	1
		Weighted mode	0.678116136	0.05	1
	IDP dMRI ProbtrackX ISOVF slf l	Inverse variance weighted	0.646164577	0.05	1
	IDP dMRI ProbtrackX ISOVF slf r	MR Egger	0.413286395	0.05	1
		Weighted median	0.410121246	0.05	1
		Inverse variance weighted	0.69758344	0.05	1

		Simple mode	0.476941954	0.05	1
		Weighted mode	0.459061606	0.05	1
	IDP dMRI ProbtrackX ISOVF unc l	Wald ratio	0.373706362	0.05	1
	IDP dMRI ProbtrackX ISOVF unc r	Wald ratio	0.413253375	0.05	1
	IDP T1 SIENAX brain-normalised volume	Wald ratio	0.52810507	0.05	0.999999981
Giant cell arteritis with polymyalgia rheumatica	IDP T1 SIENAX peripheral grey normalised volume	Wald ratio	0.842068659	0.05	1
	IDP T1 FAST ROIs R temp occ fusif cortex	Wald ratio	0.274075598	0.05	0.915095219
	IDP T1 FAST ROIs R occ pole	Inverse variance weighted	0.222057699	0.05	1
	IDP T1 FAST ROIs L thalamus	Inverse variance weighted	0.174013893	0.05	0.999999985
	IDP T1 FAST ROIs R thalamus	Wald ratio	0.954496332	0.05	1
	IDP T1 FAST ROIs L putamen	Wald ratio	0.56737924	0.05	1
	IDP T1 FAST ROIs R putamen	Wald ratio	0.56737924	0.05	1
	IDP T1 FAST ROIs L pallidum	Wald ratio	0.374319164	0.05	1
	IDP T1 FIRST left caudate volume	Wald ratio	0.117371426	0.05	1
	IDP T1 FAST ROIs L hippocampus	Inverse variance weighted	0.776975832	0.05	1
	IDP T1 FAST ROIs R hippocampus	Wald ratio	0.742688209	0.05	1
	IDP T1 FAST ROIs L ventral striatum	Wald ratio	0.56737924	0.05	1
	IDP T1 FAST ROIs R ventral striatum	Inverse variance weighted	0.747503081	0.05	1
	IDP T1 FAST ROIs L cerebellum VI	Wald ratio	0.994637913	0.05	1
	IDP T1 FAST ROIs L cerebellum crus I	MR Egger	0.571527901	0.05	1
		Weighted median	0.39343727	0.05	1
		Inverse variance weighted	0.359991756	0.05	1

		Simple mode	0.558643016	0.05	1
		Weighted mode	0.503029542	0.05	1
	IDP T1 FAST ROIs R cerebellum crus I	Inverse variance weighted	0.325311931	0.05	1
	IDP T1 FAST ROIs L cerebellum crus II	MR Egger	0.464419275	0.05	0.977677825
		Weighted median	0.243882314	0.05	1
		Inverse variance weighted	0.221723188	0.05	1
		Simple mode	0.43803817	0.05	1
		Weighted mode	0.348329835	0.05	1
	IDP T1 FAST ROIs V cerebellum crus II	Inverse variance weighted	0.919565583	0.05	1
	IDP T1 FAST ROIs R cerebellum crus II	Inverse variance weighted	0.159870339	0.05	1
	IDP T1 FIRST left putamen volume	Inverse variance weighted	0.995167211	0.05	1
	IDP T1 FAST ROIs R cerebellum VIIb	Wald ratio	0.137915368	0.05	0.999989488
	IDP T1 FAST ROIs V cerebellum VIIIa	MR Egger	0.617290113	0.05	1
		Weighted median	0.873539304	0.05	1
		Inverse variance weighted	0.838169352	0.05	1
		Simple mode	0.505707865	0.05	1
		Weighted mode	0.607628949	0.05	1
	IDP T1 FAST ROIs L cerebellum VIIIb	Wald ratio	0.392492964	0.05	1
	IDP T1 FAST ROIs V cerebellum VIIIb	Inverse variance weighted	0.836786139	0.05	1
	IDP T1 FAST ROIs R cerebellum VIIIb	Wald ratio	0.56737924	0.05	1
	IDP T1 FAST ROIs L cerebellum IX	Inverse variance weighted	0.569253743	0.05	1

	IDP T1 FIRST right putamen volume	Wald ratio	0.629199957	0.05	1
	IDP T1 FAST ROIs V cerebellum IX	Inverse variance weighted	0.440270591	0.05	0.999999999
	IDP T1 FAST ROIs R cerebellum IX	Inverse variance weighted	0.641489887	0.05	1
	IDP T1 FAST ROIs V cerebellum X	Wald ratio	0.56737924	0.05	1
	IDP T2 FLAIR BIANCA WMH volume	Inverse variance weighted	0.690212685	0.05	1
	IDP SWI T2star left caudate	MR Egger	0.675035561	0.05	1
		Weighted median	0.362213882	0.05	1
		Inverse variance weighted	0.186952288	0.05	1
		Simple mode	0.488707575	0.05	1
		Weighted mode	0.578882737	0.05	1
	IDP SWI T2star right caudate	MR Egger	0.767382862	0.05	1
		Weighted median	0.271043972	0.05	1
		Inverse variance weighted	0.473495406	0.05	1
		Simple mode	0.500480574	0.05	1
		Weighted mode	0.411474887	0.05	1
	IDP T1 FIRST left pallidum volume	Wald ratio	0.736069456	0.05	1
	IDP SWI T2star left putamen	MR Egger	0.836945875	0.05	1
		Weighted median	0.348195307	0.05	1
		Inverse variance weighted	0.597018392	0.05	1
		Simple mode	0.588396308	0.05	1
		Weighted mode	0.498438676	0.05	1
	IDP SWI T2star right putamen	MR Egger	0.366280286	0.05	1

		Weighted median	0.319288066	0.05	1
		Inverse variance weighted	0.38400047	0.05	1
		Simple mode	0.427417573	0.05	1
		Weighted mode	0.384863166	0.05	1
	IDP SWI T2star left pallidum	MR Egger	0.601659713	0.05	1
		Weighted median	0.164426892	0.05	1
		Inverse variance weighted	0.376292659	0.05	1
		Simple mode	0.352634762	0.05	1
		Weighted mode	0.322213222	0.05	1
	IDP SWI T2star right pallidum	MR Egger	0.503959376	0.05	1
		Weighted median	0.177311175	0.05	1
		Inverse variance weighted	0.134727169	0.05	1
		Simple mode	0.398307188	0.05	1
		Weighted mode	0.387055435	0.05	1
	IDP T1 FIRST right pallidum volume	Wald ratio	0.736069456	0.05	1
	IDP dMRI TBSS FA Genu of corpus callosum	Wald ratio	0.706013693	0.05	1
	IDP dMRI TBSS FA Splenium of corpus callosum	Inverse variance weighted	0.379506028	0.05	1
	IDP dMRI TBSS FA Corticospinal tract L	Wald ratio	0.828872367	0.05	1
	IDP dMRI TBSS FA Superior cerebellar peduncle R	Inverse variance weighted	0.08573183	0.05	1
	IDP dMRI TBSS FA Superior cerebellar peduncle L	Inverse variance weighted	0.087304935	0.05	1
	IDP dMRI TBSS FA Posterior limb of internal capsule R	Wald ratio	0.647011885	0.05	1

	IDP dMRI TBSS FA Retrolenticular part of internal capsule R	Inverse variance weighted	0.783392724	0.05	1
	IDP dMRI TBSS FA Anterior corona radiata R	Wald ratio	0.706013693	0.05	1
	IDP dMRI TBSS FA Anterior corona radiata L	Wald ratio	0.706013693	0.05	1
	IDP dMRI TBSS FA Posterior thalamic radiation R	Wald ratio	0.299055233	0.05	1
	IDP dMRI TBSS FA Posterior thalamic radiation L	Wald ratio	0.298438499	0.05	1
	IDP dMRI TBSS FA Sagittal stratum R	Wald ratio	0.15844539	0.05	1
	IDP dMRI TBSS FA Cingulum cingulate gyrus R	Inverse variance weighted	0.818521771	0.05	1
	IDP dMRI TBSS FA Superior longitudinal fasciculus L	Inverse variance weighted	0.586784602	0.05	1
	IDP dMRI TBSS FA Uncinate fasciculus L	Wald ratio	0.27579582	0.05	1
	IDP dMRI TBSS MD Genu of corpus callosum	Wald ratio	0.706013693	0.05	1
	IDP dMRI TBSS MD Body of corpus callosum	Wald ratio	0.537555492	0.05	1
	IDP dMRI TBSS MD Splenium of corpus callosum	Inverse variance weighted	0.640896377	0.05	1
	IDP dMRI TBSS MD Corticospinal tract R	Wald ratio	0.878165542	0.05	1
	IDP dMRI TBSS MD Superior cerebellar peduncle R	Wald ratio	0.203421046	0.05	0.999988141
	IDP dMRI TBSS MD Superior cerebellar peduncle L	Wald ratio	0.19619709	0.05	0.999810465
	IDP dMRI TBSS MD Anterior limb of internal capsule L	Wald ratio	0.657116592	0.05	1
	IDP dMRI TBSS MD Retrolenticular part of internal capsule R	Wald ratio	0.298438499	0.05	1
	IDP T1 FIRST left caudate volume plus IDP T1 FIRST right caudate volume	Wald ratio	0.295010168	0.05	1
	IDP T1 FIRST left putamen volume plus IDP T1 FIRST right putamen volume	Inverse variance weighted	0.682313294	0.05	1

	IDP T1 FIRST left pallidum volume plus IDP T1 FIRST right pallidum volume	Wald ratio	0.736069456	0.05	1
	IDP SWI T2star left thalamus plus IDP SWI T2star right thalamus	MR Egger	0.406989794	0.05	1
		Weighted median	0.164682307	0.05	1
		Inverse variance weighted	0.066435969	0.05	1
		Simple mode	0.301513905	0.05	1
		Weighted mode	0.454223584	0.05	1
	IDP dMRI TBSS MD Retrolenticular part of internal capsule L	Wald ratio	0.298438499	0.05	1
	IDP SWI T2star left caudate plus IDP SWI T2star right caudate	MR Egger	0.649953614	0.05	1
		Weighted median	0.191382363	0.05	1
		Inverse variance weighted	0.306521179	0.05	1
		Simple mode	0.349676649	0.05	1
		Weighted mode	0.267043744	0.05	1
	IDP SWI T2star left putamen plus IDP SWI T2star right putamen	MR Egger	0.870119206	0.05	1
		Weighted median	0.331573373	0.05	1
		Inverse variance weighted	0.424019647	0.05	1
		Simple mode	0.753096021	0.05	1
		Weighted mode	0.506985189	0.05	1
	IDP SWI T2star left pallidum plus IDP SWI T2star right pallidum	MR Egger	0.571670673	0.05	1
		Weighted median	0.181756973	0.05	1
		Inverse variance weighted	0.211964911	0.05	1
		Simple mode	0.387576873	0.05	1
		Weighted mode	0.383269294	0.05	1

	volume Left-Lateral-Ventricle	MR Egger	0.845164169	0.05	1
		Weighted median	0.172144546	0.05	1
		Inverse variance weighted	0.770842918	0.05	1
		Simple mode	0.382398125	0.05	1
		Weighted mode	0.420235045	0.05	1
	volume Left-Cerebellum-White-Matter	Inverse variance weighted	0.65471593	0.05	1
	volume Left-Cerebellum-Cortex	Inverse variance weighted	0.83105857	0.05	1
	IDP dMRI TBSS MD Anterior corona radiata R	Inverse variance weighted	0.75384454	0.05	1
	volume Left-Putamen	Inverse variance weighted	0.517672447	0.05	1
	volume 4th-Ventricle	Inverse variance weighted	0.021560737	0.05	1
	volume Brain-Stem	Wald ratio	0.695929859	0.05	1
	volume Left-Hippocampus	Inverse variance weighted	0.784768268	0.05	1
	volume CSF	MR Egger	0.814997582	0.05	0.002698321
		Weighted median	0.670387393	0.05	1
		Inverse variance weighted	0.445754054	0.05	1
		Simple mode	0.864253067	0.05	1
		Weighted mode	0.866749481	0.05	1
	IDP dMRI TBSS MD Anterior corona radiata L	Inverse variance weighted	0.790641745	0.05	1
	volume Left-Accumbens-area	Wald ratio	0.56737924	0.05	1
	volume Right-Lateral-Ventricle	Inverse variance weighted	0.109731245	0.05	1
	volume Right-Inf-Lat-Vent	Wald ratio	0.928322957	0.05	1

	volume Right-Cerebellum-White-Matter	Wald ratio	0.79666919	0.05	1
	volume Right-Cerebellum-Cortex	MR Egger	0.660747008	0.05	1
		Weighted median	0.393585822	0.05	1
		Inverse variance weighted	0.420302458	0.05	1
		Simple mode	0.554480276	0.05	1
		Weighted mode	0.514106709	0.05	1
	IDP dMRI TBSS MD Superior corona radiata R	Inverse variance weighted	0.754578188	0.05	1
	volume Right-Putamen	Wald ratio	0.629199957	0.05	1
	volume Right-Hippocampus	Wald ratio	0.742688209	0.05	1
	volume Right-Amygdala	Wald ratio	0.478136886	0.05	0.999999994
	volume Right-VentralDC	Wald ratio	0.687419759	0.05	1
	volume CC Posterior	Wald ratio	0.027687035	0.05	1
	IDP dMRI TBSS MD Superior corona radiata L	Inverse variance weighted	0.758514702	0.05	1
	IDP dMRI TBSS MD Posterior corona radiata R	Wald ratio	0.298438499	0.05	0.999999997
	volume BrainSegVol-to-eTIV	Wald ratio	0.177886645	0.05	0.999999999
	IDP dMRI TBSS MD Posterior corona radiata L	Wald ratio	0.298438499	0.05	1
	volume rhSurfaceHoles	Wald ratio	0.772808721	0.05	1
	DKTatlas lh cuneus area	Wald ratio	0.563938068	0.05	1
	IDP dMRI TBSS MD Posterior thalamic radiation R	Wald ratio	0.298438499	0.05	1
	DKTatlas lh lateraloccipital area	Wald ratio	0.563938068	0.05	1
	DKTatlas lh lingual area	Wald ratio	0.536208434	0.05	1
	IDP dMRI TBSS MD Posterior thalamic radiation L	Wald ratio	0.298438499	0.05	1

	DKTatlas lh parstriangularis area	Wald ratio	0.620378072	0.05	1
	DKTatlas lh pericalcarine area	Inverse variance weighted	0.746044056	0.05	1
	DKTatlas lh postcentral area	Inverse variance weighted	0.826127978	0.05	1
	DKTatlas lh posteriorcingulate area	Wald ratio	0.962600159	0.05	1
	DKTatlas lh precentral area	Wald ratio	0.762856948	0.05	1
	DKTatlas lh precuneus area	Wald ratio	0.706427909	0.05	1
	IDP dMRI TBSS MD Sagittal stratum R	Wald ratio	0.298438499	0.05	1
	DKTatlas lh superiorparietal area	Wald ratio	0.418003875	0.05	1
	DKTatlas lh superiortemporal area	Wald ratio	0.720984862	0.05	1
	DKTatlas lh supramarginal area	Wald ratio	0.389532705	0.05	0.999999999
	DKTatlas lh WhiteSurfArea area	Wald ratio	0.285085208	0.05	0.964921164
	IDP dMRI TBSS MD Sagittal stratum L	Wald ratio	0.298438499	0.05	1
	a2009s lh G&S subcentral area	Wald ratio	0.762856948	0.05	1
	a2009s lh G cuneus area	Wald ratio	0.53415435	0.05	1
	a2009s lh G front inf-Opercular area	Wald ratio	0.98704347	0.05	1
	IDP dMRI TBSS MD External capsule R	Wald ratio	0.706013693	0.05	1
	IDP dMRI TBSS MD External capsule L	Inverse variance weighted	0.982258567	0.05	1
	a2009s lh G pariet inf-Supramar area	Wald ratio	0.389532705	0.05	1
	a2009s lh G parietal sup area	Wald ratio	0.418003875	0.05	1
	a2009s lh G postcentral area	Wald ratio	0.419475447	0.05	1
	a2009s lh G precentral area	Wald ratio	0.762856948	0.05	1
	a2009s lh G precuneus area	Wald ratio	0.53415435	0.05	1
		MR Egger	0.498884368	0.05	1

	IDP dMRI TBSS MD Cingulum cingulate gyrus R	Weighted median	0.725439753	0.05	1
		Inverse variance weighted	0.658726705	0.05	1
		Simple mode	0.526771796	0.05	1
		Weighted mode	0.84386513	0.05	1
	IDP dMRI TBSS MD Cingulum cingulate gyrus L	MR Egger	0.499330996	0.05	1
		Weighted median	0.729860182	0.05	1
		Inverse variance weighted	0.659490175	0.05	1
		Simple mode	0.508923359	0.05	1
		Weighted mode	0.850836147	0.05	1
	a2009s lh S calcarine area	Wald ratio	0.536208434	0.05	1
	a2009s lh S central area	Wald ratio	0.751767434	0.05	1
	a2009s lh S collat transv ant area	Wald ratio	0.016052018	0.05	0.778792016
	a2009s lh S front middle area	Wald ratio	0.247658189	0.05	1
	IDP dMRI TBSS MD Cingulum hippocampus R	Wald ratio	0.298438499	0.05	1
	a2009s lh S intrapariet&P trans area	Wald ratio	0.418003875	0.05	1
	IDP dMRI TBSS MD Cingulum hippocampus L	Wald ratio	0.298438499	0.05	1
	a2009s lh S subparietal area	Wald ratio	0.536208434	0.05	1
	DKTatlas rh cuneus area	Wald ratio	0.536208434	0.05	1
	DKTatlas rh lingual area	Wald ratio	0.536208434	0.05	1
	DKTatlas rh parstriangularis area	Wald ratio	0.703790774	0.05	1
	DKTatlas rh pericalcarine area	Inverse variance weighted	0.818231103	0.05	1
	IDP dMRI TBSS MD Superior longitudinal fasciculus R	Wald ratio	0.298438499	0.05	1

	DKTatlas rh postcentral area	Wald ratio	0.751767434	0.05	1
	DKTatlas rh precentral area	Wald ratio	0.762856948	0.05	1
	IDP dMRI TBSS MD Superior longitudinal fasciculus L	Wald ratio	0.298438499	0.05	1
	a2009s rh G&S subcentral area	Wald ratio	0.751767434	0.05	1
	a2009s rh G&S cingul-Mid-Post area	Wald ratio	0.457949219	0.05	1
	a2009s rh G cuneus area	Wald ratio	0.536208434	0.05	1
	IDP dMRI TBSS MD Superior fronto-occipital fasciculus L	Wald ratio	0.22581612	0.05	0.999980723
	a2009s rh G oc-temp med-Lingual area	Wald ratio	0.536208434	0.05	1
	a2009s rh G parietal sup area	Wald ratio	0.180282933	0.05	1
	IDP dMRI TBSS MD Uncinate fasciculus R	Wald ratio	0.654665224	0.05	1
	a2009s rh G precentral area	Wald ratio	0.762856948	0.05	1
	a2009s rh G precuneus area	Wald ratio	0.894903398	0.05	1
	IDP dMRI TBSS MD Uncinate fasciculus L	Inverse variance weighted	0.932217728	0.05	1
	a2009s rh Pole occipital area	Wald ratio	0.536208434	0.05	1
	a2009s rh S calcarine area	Wald ratio	0.536208434	0.05	1
	a2009s rh S central area	Wald ratio	0.762856948	0.05	1
	a2009s rh S collat transv ant area	Wald ratio	0.016052018	0.05	0.826899392
	a2009s rh S orbital med-olfact area	Wald ratio	0.937901062	0.05	1
	IDP dMRI TBSS MO Pontine crossing tract	MR Egger	0.946429074	0.05	1
		Weighted median	0.798530223	0.05	1
		Inverse variance weighted	0.71122241	0.05	1
		Simple mode	0.773269595	0.05	1

		Weighted mode	0.551294186	0.05	1
	DKTatlas lh postcentral thickness	Wald ratio	0.751767434	0.05	1
	a2009s lh G insular short thickness	Wald ratio	0.202439128	0.05	1
	a2009s lh G postcentral thickness	Wald ratio	0.751767434	0.05	1
	IDP T1 SIENAX CSF normalised volume	Inverse variance weighted	0.661467624	0.05	1
	IDP dMRI TBSS MO Medial lemniscus R	Wald ratio	0.253968785	0.05	0.999999809
	a2009s lh S postcentral thickness	Wald ratio	0.195382084	0.05	1
	DKTatlas rh lateraloccipital thickness	Wald ratio	0.56737924	0.05	0.999996308
	DKTatlas rh parstriangularis thickness	Wald ratio	0.593825453	0.05	1
	DKTatlas rh postcentral thickness	Wald ratio	0.751767434	0.05	1
	DKTatlas rh posteriorcingulate thickness	Inverse variance weighted	0.023476945	0.05	0.992686429
	a2009s rh G&S cingul-Mid-Ant thickness	Wald ratio	0.499559835	0.05	1
	a2009s rh G cuneus thickness	Wald ratio	0.56737924	0.05	0.999999937
	a2009s rh G postcentral thickness	Wald ratio	0.752392682	0.05	1
	a2009s rh Pole occipital thickness	Wald ratio	0.56737924	0.05	0.999999968
	a2009s rh S oc sup&transversal thickness	Wald ratio	0.393771304	0.05	1
	a2009s rh S parieto occipital thickness	Wald ratio	0.407078181	0.05	1
	IDP dMRI TBSS MO Anterior corona radiata R	Wald ratio	0.666014762	0.05	1
	IDP dMRI TBSS MO Superior corona radiata R	Wald ratio	0.217774488	0.05	1
	IDP dMRI TBSS MO Cingulum cingulate gyrus L	Inverse variance weighted	0.259078048	0.05	1
	IDP dMRI TBSS L1 Anterior limb of internal capsule L	Wald ratio	0.657116592	0.05	1
	IDP dMRI TBSS L1 Posterior limb of internal capsule R	Wald ratio	0.044405909	0.05	0.906134861

	IDP dMRI TBSS L1 Posterior limb of internal capsule L	Wald ratio	0.038337768	0.05	0.957049418
	IDP dMRI TBSS L1 Anterior corona radiata R	Wald ratio	0.706013693	0.05	1
	IDP dMRI TBSS L1 Anterior corona radiata L	Wald ratio	0.706013693	0.05	1
	IDP dMRI TBSS L1 Posterior corona radiata R	Wald ratio	0.682147727	0.05	1
	IDP dMRI TBSS L1 Posterior corona radiata L	Wald ratio	0.706013693	0.05	1
	IDP dMRI TBSS L1 Sagittal stratum L	Wald ratio	0.706013693	0.05	1
	IDP dMRI TBSS L1 External capsule R	Wald ratio	0.706013693	0.05	1
	IDP dMRI TBSS L1 External capsule L	Inverse variance weighted	0.417960833	0.05	1
	IDP dMRI TBSS L1 Fornix cres+Stria terminalis L	Wald ratio	0.626396409	0.05	1
	IDP T1 FAST ROIs L precentral gyrus	Wald ratio	0.751767434	0.05	1
	IDP dMRI TBSS L1 Superior longitudinal fasciculus R	Inverse variance weighted	0.821288468	0.05	1
	IDP dMRI TBSS L1 Uncinate fasciculus L	Wald ratio	0.570112978	0.05	1
	IDP dMRI TBSS L2 Pontine crossing tract	Wald ratio	0.253968785	0.05	1
	IDP dMRI TBSS L2 Genu of corpus callosum	MR Egger	0.80541692	0.05	1
		Weighted median	0.672821385	0.05	1
		Inverse variance weighted	0.825690926	0.05	1
		Simple mode	0.589983414	0.05	1
		Weighted mode	0.651685123	0.05	1
	IDP dMRI TBSS L2 Body of corpus callosum	Wald ratio	0.706013693	0.05	1
	IDP dMRI TBSS L2 Splenium of corpus callosum	MR Egger	0.791615091	0.05	0.981361027
		Weighted median	0.267444315	0.05	1

		Inverse variance weighted	0.190743495	0.05	1
		Simple mode	0.471273167	0.05	1
		Weighted mode	0.472469775	0.05	1
	IDP dMRI TBSS L2 Corticospinal tract R	Wald ratio	0.878165542	0.05	1
	IDP T1 SIENAX CSF unnormalised volume	MR Egger	0.974493714	0.05	1
		Weighted median	0.282228208	0.05	1
		Inverse variance weighted	0.803593262	0.05	1
		Simple mode	0.412228769	0.05	1
		Weighted mode	0.400926569	0.05	1
	IDP T1 FAST ROIs L temporal pole	Wald ratio	0.864675075	0.05	1
	IDP dMRI TBSS L2 Superior cerebellar peduncle R	Inverse variance weighted	0.084789674	0.05	1
	IDP dMRI TBSS L2 Superior cerebellar peduncle L	Wald ratio	0.19619709	0.05	1
	IDP dMRI TBSS L2 Posterior limb of internal capsule R	Inverse variance weighted	0.374773414	0.05	1
	IDP dMRI TBSS L2 Posterior limb of internal capsule L	Wald ratio	0.427149988	0.05	1
	IDP dMRI TBSS L2 Retrolenticular part of internal capsule R	Inverse variance weighted	0.663401849	0.05	1
	IDP dMRI TBSS L2 Anterior corona radiata R	Inverse variance weighted	0.759611361	0.05	1
	IDP dMRI TBSS L2 Anterior corona radiata L	Wald ratio	0.706013693	0.05	1
	IDP dMRI TBSS L2 Superior corona radiata R	Inverse variance weighted	0.316613716	0.05	1
	IDP dMRI TBSS L2 Superior corona radiata L	Wald ratio	0.298438499	0.05	1
	IDP dMRI TBSS L2 Posterior corona radiata R	Wald ratio	0.298438499	0.05	1

	IDP dMRI TBSS L2 Posterior corona radiata L	Wald ratio	0.298438499	0.05	1
	IDP dMRI TBSS L2 Posterior thalamic radiation R	Wald ratio	0.299055233	0.05	1
	IDP dMRI TBSS L2 Posterior thalamic radiation L	Wald ratio	0.298438499	0.05	1
	IDP dMRI TBSS L2 Sagittal stratum R	Wald ratio	0.298438499	0.05	1
	IDP dMRI TBSS L2 Cingulum cingulate gyrus R	Wald ratio	0.706013693	0.05	1
	IDP dMRI TBSS L2 Cingulum hippocampus R	Wald ratio	0.298438499	0.05	1
	IDP dMRI TBSS L2 Cingulum hippocampus L	Wald ratio	0.706013693	0.05	1
	IDP dMRI TBSS L2 Uncinate fasciculus L	Wald ratio	0.706013693	0.05	1
	IDP dMRI TBSS L3 Genu of corpus callosum	Wald ratio	0.706013693	0.05	1
	IDP dMRI TBSS L3 Body of corpus callosum	Wald ratio	0.706013693	0.05	1
	IDP dMRI TBSS L3 Splenium of corpus callosum	Wald ratio	0.43186591	0.05	1
	IDP dMRI TBSS L3 Inferior cerebellar peduncle R	Wald ratio	0.706013693	0.05	1
	IDP dMRI TBSS L3 Inferior cerebellar peduncle L	Wald ratio	0.706013693	0.05	1
	IDP dMRI TBSS L3 Superior cerebellar peduncle R	Inverse variance weighted	0.085448803	0.05	1
	IDP dMRI TBSS L3 Superior cerebellar peduncle L	Wald ratio	0.19619709	0.05	1
	IDP dMRI TBSS L3 Cerebral peduncle R	Wald ratio	0.689581391	0.05	1
	IDP dMRI TBSS L3 Anterior limb of internal capsule R	Wald ratio	0.717013143	0.05	1
	IDP dMRI TBSS L3 Anterior limb of internal capsule L	Wald ratio	0.739487542	0.05	1
	IDP dMRI TBSS L3 Retrolenticular part of internal capsule R	Wald ratio	0.298438499	0.05	1

	IDP dMRI TBSS L3 Anterior corona radiata R	Inverse variance weighted	0.757804429	0.05	1
	IDP dMRI TBSS L3 Anterior corona radiata L	Inverse variance weighted	0.799118483	0.05	1
	IDP dMRI TBSS L3 Posterior corona radiata R	Inverse variance weighted	0.714526429	0.05	1
	IDP dMRI TBSS L3 Posterior thalamic radiation R	Wald ratio	0.299055233	0.05	1
	IDP dMRI TBSS L3 Posterior thalamic radiation L	Wald ratio	0.298438499	0.05	1
	IDP dMRI TBSS L3 Sagittal stratum R	Wald ratio	0.298438499	0.05	1
	IDP dMRI TBSS L3 Sagittal stratum L	Wald ratio	0.298438499	0.05	1
	IDP dMRI TBSS L3 External capsule R	Wald ratio	0.706013693	0.05	1
	IDP dMRI TBSS L3 External capsule L	Wald ratio	0.706013693	0.05	1
	IDP dMRI TBSS L3 Cingulum cingulate gyrus R	Inverse variance weighted	0.786023481	0.05	1
	IDP dMRI TBSS L3 Cingulum cingulate gyrus L	Wald ratio	0.706013693	0.05	1
	IDP dMRI TBSS L3 Cingulum hippocampus R	Wald ratio	0.706013693	0.05	1
	IDP dMRI TBSS L3 Cingulum hippocampus L	Wald ratio	0.706013693	0.05	1
	IDP dMRI TBSS L3 Fornix cres+Stria terminalis R	Wald ratio	0.124643498	0.05	0.986898386
	IDP dMRI TBSS L3 Superior longitudinal fasciculus R	Wald ratio	0.298438499	0.05	1
	IDP dMRI TBSS L3 Superior longitudinal fasciculus L	Wald ratio	0.298438499	0.05	1
	IDP dMRI TBSS L3 Uncinate fasciculus R	Wald ratio	0.706013693	0.05	1
	IDP dMRI TBSS L3 Uncinate fasciculus L	Inverse variance weighted	0.339714843	0.05	1
	IDP dMRI TBSS ICVF Middle cerebellar peduncle	Wald ratio	0.298438499	0.05	1
		MR Egger	0.870209923	0.05	1

	IDP dMRI TBSS ICVF Genu of corpus callosum	Weighted median	0.987862986	0.05	1
		Inverse variance weighted	0.962335149	0.05	1
		Simple mode	0.424920574	0.05	1
		Weighted mode	0.863118005	0.05	1
	IDP dMRI TBSS ICVF Body of corpus callosum	MR Egger	0.921384169	0.05	1
		Weighted median	0.526172192	0.05	1
		Inverse variance weighted	0.568829144	0.05	1
		Simple mode	0.527270618	0.05	1
		Weighted mode	0.525117497	0.05	1
	IDP dMRI TBSS ICVF Splenium of corpus callosum	MR Egger	0.238486706	0.05	1
		Weighted median	0.913355176	0.05	1
		Inverse variance weighted	0.5943861	0.05	1
		Simple mode	0.855536151	0.05	1
		Weighted mode	0.783391946	0.05	1
	IDP dMRI TBSS ICVF Fornix	Wald ratio	0.22581612	0.05	1
	IDP dMRI TBSS ICVF Medial lemniscus R	Inverse variance weighted	0.963938432	0.05	1
	IDP dMRI TBSS ICVF Medial lemniscus L	Wald ratio	0.107655849	0.05	0.998878759
	IDP dMRI TBSS ICVF Inferior cerebellar peduncle R	Inverse variance weighted	0.987547578	0.05	1
	IDP dMRI TBSS ICVF Inferior cerebellar peduncle L	Inverse variance weighted	0.977986628	0.05	1
	IDP dMRI TBSS ICVF Superior cerebellar peduncle R	MR Egger	0.983745564	0.05	1
		Weighted median	0.233454473	0.05	1
		Inverse variance weighted	0.301877208	0.05	1

		Simple mode	0.310417463	0.05	1
		Weighted mode	0.340221492	0.05	1
	IDP dMRI TBSS ICVF Superior cerebellar peduncle L	Inverse variance weighted	0.48063086	0.05	1
	IDP dMRI TBSS ICVF Cerebral peduncle R	MR Egger	0.625550943	0.05	1
		Weighted median	0.604080878	0.05	1
		Inverse variance weighted	0.701866711	0.05	1
		Simple mode	0.647653425	0.05	1
		Weighted mode	0.646206266	0.05	1
	IDP dMRI TBSS ICVF Cerebral peduncle L	Wald ratio	0.392492964	0.05	1
	IDP dMRI TBSS ICVF Anterior limb of internal capsule R	Wald ratio	0.706013693	0.05	1
	IDP dMRI TBSS ICVF Anterior limb of internal capsule L	MR Egger	0.759097577	0.05	0.998830054
		Weighted median	0.999775077	0.05	1
		Inverse variance weighted	0.614047225	0.05	1
		Simple mode	0.527940961	0.05	1
		Weighted mode	0.941342054	0.05	1
	IDP dMRI TBSS ICVF Posterior limb of internal capsule R	MR Egger	0.495350341	0.05	1
		Weighted median	0.298581572	0.05	1
		Inverse variance weighted	0.527980327	0.05	1
		Simple mode	0.428447393	0.05	1
		Weighted mode	0.412275521	0.05	1
	IDP dMRI TBSS ICVF Posterior limb of internal capsule L	MR Egger	0.831455181	0.05	1
		Weighted median	0.715589644	0.05	1

		Inverse variance weighted	0.904097729	0.05	1
		Simple mode	0.581835592	0.05	1
		Weighted mode	0.673551726	0.05	1
	IDP dMRI TBSS ICVF Retrolenticular part of internal capsule R	MR Egger	0.706706793	0.05	1
		Weighted median	0.879118367	0.05	1
		Inverse variance weighted	0.793188809	0.05	1
		Simple mode	0.348591891	0.05	1
		Weighted mode	0.816527698	0.05	1
	IDP dMRI TBSS ICVF Retrolenticular part of internal capsule L	MR Egger	0.908628292	0.05	1
		Weighted median	0.844727059	0.05	1
		Inverse variance weighted	0.928237213	0.05	1
		Simple mode	0.596710993	0.05	1
		Weighted mode	0.74651031	0.05	1
	IDP dMRI TBSS ICVF Anterior corona radiata R	MR Egger	0.925249213	0.05	1
		Weighted median	0.838678918	0.05	1
		Inverse variance weighted	0.944105285	0.05	1
		Simple mode	0.594883576	0.05	1
		Weighted mode	0.741706385	0.05	1
	IDP dMRI TBSS ICVF Anterior corona radiata L	MR Egger	0.879093825	0.05	1
		Weighted median	0.790855132	0.05	1
		Inverse variance weighted	0.916346007	0.05	1
		Simple mode	0.586589668	0.05	1

		Weighted mode	0.735364197	0.05	1
	IDP dMRI TBSS ICVF Superior corona radiata R	MR Egger	0.979156615	0.05	1
		Weighted median	0.881858216	0.05	1
		Inverse variance weighted	0.97122688	0.05	1
		Simple mode	0.602159433	0.05	1
		Weighted mode	0.768183141	0.05	1
	IDP dMRI TBSS ICVF Superior corona radiata L	MR Egger	0.944857458	0.05	1
		Weighted median	0.849312547	0.05	1
		Inverse variance weighted	0.954139213	0.05	1
		Simple mode	0.614142097	0.05	1
		Weighted mode	0.734621049	0.05	1
	IDP dMRI TBSS ICVF Posterior corona radiata R	MR Egger	0.591443152	0.05	0.999999282
		Weighted median	0.913248192	0.05	1
		Inverse variance weighted	0.729754878	0.05	1
		Simple mode	0.862717794	0.05	1
		Weighted mode	0.881640205	0.05	1
	IDP dMRI TBSS ICVF Posterior corona radiata L	MR Egger	0.614170912	0.05	1
		Weighted median	0.915371072	0.05	1
		Inverse variance weighted	0.738840508	0.05	1
		Simple mode	0.823744399	0.05	1
		Weighted mode	0.85037056	0.05	1
	IDP dMRI TBSS ICVF Posterior thalamic radiation R	Wald ratio	0.298438499	0.05	1

	IDP dMRI TBSS ICVF Posterior thalamic radiation L	Inverse variance weighted	0.875976841	0.05	1
	IDP dMRI TBSS ICVF Sagittal stratum R	MR Egger	0.316772462	0.05	1
		Weighted median	0.371966917	0.05	1
		Inverse variance weighted	0.759701023	0.05	1
		Simple mode	0.294124777	0.05	1
		Weighted mode	0.380648638	0.05	1
	IDP dMRI TBSS ICVF Sagittal stratum L	Inverse variance weighted	0.889769751	0.05	1
	IDP dMRI TBSS ICVF External capsule R	MR Egger	0.679279787	0.05	1
		Weighted median	0.999718516	0.05	1
		Inverse variance weighted	0.977005509	0.05	1
		Simple mode	0.556529362	0.05	1
		Weighted mode	0.738123482	0.05	1
	IDP dMRI TBSS ICVF External capsule L	Inverse variance weighted	0.889911831	0.05	1
	IDP dMRI TBSS ICVF Cingulum cingulate gyrus R	Inverse variance weighted	0.889978991	0.05	1
	IDP dMRI TBSS ICVF Cingulum cingulate gyrus L	Wald ratio	0.740611284	0.05	1
	IDP dMRI TBSS ICVF Cingulum hippocampus R	MR Egger	0.602776459	0.05	1
		Weighted median	0.926614116	0.05	1
		Inverse variance weighted	0.858991424	0.05	1
		Simple mode	0.89759551	0.05	1
		Weighted mode	0.813543688	0.05	1
		MR Egger	0.877349389	0.05	1

	IDP dMRI TBSS ICVF Cingulum hippocampus L	Weighted median	0.817020522	0.05	1
		Inverse variance weighted	0.903312778	0.05	1
		Simple mode	0.584841877	0.05	1
		Weighted mode	0.744134763	0.05	1
	IDP dMRI TBSS ICVF Fornix cres+Stria terminalis R	Wald ratio	0.241915357	0.05	0.999999997
	IDP dMRI TBSS ICVF Superior longitudinal fasciculus R	Inverse variance weighted	0.722436497	0.05	1
	IDP dMRI TBSS ICVF Superior longitudinal fasciculus L	MR Egger	0.959811974	0.05	1
		Weighted median	0.950450943	0.05	1
		Inverse variance weighted	0.998834958	0.05	1
		Simple mode	0.622068932	0.05	1
		Weighted mode	0.816335429	0.05	1
	IDP dMRI TBSS ICVF Superior fronto-occipital fasciculus R	Inverse variance weighted	0.833362143	0.05	1
	IDP dMRI TBSS ICVF Uncinate fasciculus R	Wald ratio	0.706013693	0.05	1
	IDP dMRI TBSS ICVF Uncinate fasciculus L	Wald ratio	0.706013693	0.05	1
	IDP dMRI TBSS OD Pontine crossing tract	MR Egger	0.51337888	0.05	0.059041615
		Weighted median	0.054251414	0.05	1
		Inverse variance weighted	0.032479054	0.05	1
		Simple mode	0.251356969	0.05	1
		Weighted mode	0.206575381	0.05	1
	IDP dMRI TBSS OD Cerebral peduncle R	Wald ratio	0.620284611	0.05	1
	IDP dMRI TBSS OD Cerebral peduncle L	Wald ratio	0.620284611	0.05	1
	IDP dMRI TBSS OD Posterior limb of internal capsule L	Wald ratio	0.038337768	0.05	1

	IDP dMRI TBSS OD External capsule R	Inverse variance weighted	0.684807006	0.05	1
	IDP dMRI TBSS OD External capsule L	Wald ratio	0.7775124	0.05	1
	IDP dMRI TBSS OD Superior longitudinal fasciculus R	Wald ratio	0.968840559	0.05	1
	IDP dMRI TBSS ISOVF Fornix	Wald ratio	0.360758818	0.05	1
	IDP dMRI TBSS ISOVF External capsule R	Wald ratio	0.195142799	0.05	1
	IDP dMRI TBSS ISOVF Cingulum cingulate gyrus R	Wald ratio	0.703541913	0.05	1
	IDP dMRI ProbtrackX FA ar l	Wald ratio	0.706013693	0.05	1
	IDP dMRI ProbtrackX FA atr l	Wald ratio	0.706013693	0.05	1
	IDP dMRI ProbtrackX FA fmi	MR Egger	0.632969682	0.05	1
		Weighted median	0.870099622	0.05	1
		Inverse variance weighted	0.814104873	0.05	1
		Simple mode	0.413453118	0.05	1
		Weighted mode	0.708743183	0.05	1
	IDP dMRI ProbtrackX FA ifo l	Wald ratio	0.298438499	0.05	1
	IDP dMRI ProbtrackX FA ifo r	Wald ratio	0.329001374	0.05	1
	IDP dMRI ProbtrackX FA ml l	Wald ratio	0.769093682	0.05	1
	IDP dMRI ProbtrackX FA ptr r	Wald ratio	0.574163988	0.05	1
	IDP dMRI ProbtrackX FA slf l	Wald ratio	0.329001374	0.05	1
	IDP dMRI ProbtrackX FA str l	Wald ratio	0.947930268	0.05	1
	IDP dMRI ProbtrackX FA unc l	Wald ratio	0.706013693	0.05	1
	IDP dMRI ProbtrackX FA unc r	Wald ratio	0.706013693	0.05	1
	IDP dMRI ProbtrackX MD ar l	Wald ratio	0.706013693	0.05	1

	IDP dMRI ProbtrackX MD ar r	Wald ratio	0.706013693	0.05	1
	IDP dMRI ProbtrackX MD atr l	Wald ratio	0.706013693	0.05	1
	IDP dMRI ProbtrackX MD atr r	Wald ratio	0.706013693	0.05	1
	IDP dMRI ProbtrackX MD cgc l	MR Egger	0.506097689	0.05	1
		Weighted median	0.805147214	0.05	1
		Inverse variance weighted	0.689598946	0.05	1
		Simple mode	0.568728094	0.05	1
		Weighted mode	0.874050201	0.05	1
	IDP dMRI ProbtrackX MD cgc r	Inverse variance weighted	0.281535764	0.05	1
	IDP dMRI ProbtrackX MD fmi	Inverse variance weighted	0.78759176	0.05	1
	IDP dMRI ProbtrackX MD ifo l	Wald ratio	0.298438499	0.05	1
	IDP dMRI ProbtrackX MD ifo r	Wald ratio	0.298438499	0.05	1
	IDP dMRI ProbtrackX MD ilf l	Wald ratio	0.298438499	0.05	1
	IDP dMRI ProbtrackX MD ilf r	Wald ratio	0.298438499	0.05	1
	IDP dMRI ProbtrackX MD ptr l	Wald ratio	0.706013693	0.05	1
	IDP dMRI ProbtrackX MD ptr r	Wald ratio	0.706013693	0.05	1
	IDP dMRI ProbtrackX MD slf l	Inverse variance weighted	0.764487206	0.05	1
	IDP dMRI ProbtrackX MD slf r	Wald ratio	0.298438499	0.05	1
	IDP dMRI ProbtrackX MD str l	Wald ratio	0.834387033	0.05	1
	IDP dMRI ProbtrackX MD str r	Wald ratio	0.706013693	0.05	1
	IDP dMRI ProbtrackX MD unc l	Wald ratio	0.706013693	0.05	1
	IDP dMRI ProbtrackX MD unc r	Inverse variance weighted	0.90317253	0.05	1

	IDP dMRI ProbtrackX MO atr l	Wald ratio	0.837354469	0.05	1
	IDP dMRI ProbtrackX MO ml r	Wald ratio	0.901354266	0.05	1
	IDP T1 FAST ROIs L intracalc cortex	Wald ratio	0.536208434	0.05	1
	IDP dMRI ProbtrackX L1 ilf l	Wald ratio	0.706013693	0.05	1
	IDP T1 FAST ROIs R intracalc cortex	Wald ratio	0.536208434	0.05	1
	IDP dMRI ProbtrackX L1 slf l	Wald ratio	0.706013693	0.05	1
	IDP dMRI ProbtrackX L1 slf r	Wald ratio	0.502621718	0.05	0.999999991
	IDP dMRI ProbtrackX L1 str r	Wald ratio	0.753772399	0.05	1
	IDP dMRI ProbtrackX L1 unc l	Inverse variance weighted	0.967096004	0.05	1
	IDP dMRI ProbtrackX L1 unc r	Wald ratio	0.426198122	0.05	1
	IDP dMRI ProbtrackX L2 ar l	Wald ratio	0.706013693	0.05	1
	IDP dMRI ProbtrackX L2 ar r	Wald ratio	0.706013693	0.05	1
	IDP dMRI ProbtrackX L2 atr l	Wald ratio	0.706013693	0.05	1
	IDP dMRI ProbtrackX L2 atr r	Wald ratio	0.706013693	0.05	1
	IDP dMRI ProbtrackX L2 cgh l	Wald ratio	0.198207367	0.05	0.999999549
	IDP dMRI ProbtrackX L2 fma	Wald ratio	0.546137667	0.05	1
	IDP dMRI ProbtrackX L2 fmi	Inverse variance weighted	0.785542784	0.05	1
	IDP dMRI ProbtrackX L2 ifo l	Wald ratio	0.298438499	0.05	1
	IDP dMRI ProbtrackX L2 ifo r	Wald ratio	0.298438499	0.05	1
	IDP dMRI ProbtrackX L2 ilf l	Wald ratio	0.298438499	0.05	1
	IDP dMRI ProbtrackX L2 ilf r	Wald ratio	0.298438499	0.05	1
	IDP dMRI ProbtrackX L2 ptr l	Wald ratio	0.706013693	0.05	1
	IDP dMRI ProbtrackX L2 ptr r	Wald ratio	0.696994672	0.05	1

	IDP dMRI ProbtrackX L2 slf l	Inverse variance weighted	0.758541884	0.05	1
	IDP dMRI ProbtrackX L2 slf r	Wald ratio	0.298438499	0.05	1
	IDP dMRI ProbtrackX L2 str l	Wald ratio	0.834387033	0.05	1
	IDP dMRI ProbtrackX L2 str r	Wald ratio	0.834387033	0.05	1
	IDP dMRI ProbtrackX L2 unc l	Wald ratio	0.706013693	0.05	1
	IDP dMRI ProbtrackX L2 unc r	Inverse variance weighted	0.91750661	0.05	1
	IDP dMRI ProbtrackX L3 ar l	Wald ratio	0.706013693	0.05	1
	IDP dMRI ProbtrackX L3 ar r	Wald ratio	0.706013693	0.05	1
	IDP dMRI ProbtrackX L3 atr l	Wald ratio	0.706013693	0.05	1
	IDP dMRI ProbtrackX L3 atr r	Wald ratio	0.706013693	0.05	1
	IDP dMRI ProbtrackX L3 fmi	MR Egger	0.946622659	0.05	1
		Weighted median	0.826303769	0.05	1
		Inverse variance weighted	0.959206537	0.05	1
		Simple mode	0.596962232	0.05	1
		Weighted mode	0.71498752	0.05	1
	IDP dMRI ProbtrackX L3 ifo l	Inverse variance weighted	0.823398721	0.05	1
	IDP dMRI ProbtrackX L3 ifo r	Wald ratio	0.298438499	0.05	1
	IDP dMRI ProbtrackX L3 ilf l	Wald ratio	0.298438499	0.05	1
	IDP dMRI ProbtrackX L3 ilf r	Wald ratio	0.298438499	0.05	1
	IDP dMRI ProbtrackX L3 ptr l	Wald ratio	0.706013693	0.05	1
	IDP dMRI ProbtrackX L3 ptr r	Wald ratio	0.696994672	0.05	1
	IDP dMRI ProbtrackX L3 slf l	Inverse variance weighted	0.775710216	0.05	1

	IDP dMRI ProbtrackX L3 slf r	Wald ratio	0.298438499	0.05	1
	IDP dMRI ProbtrackX L3 str l	Wald ratio	0.696994672	0.05	1
	IDP dMRI ProbtrackX L3 unc l	Wald ratio	0.706013693	0.05	1
	IDP dMRI ProbtrackX L3 unc r	Inverse variance weighted	0.879194287	0.05	1
	IDP dMRI ProbtrackX ICVF ar l	MR Egger	0.890446044	0.05	1
		Weighted median	0.688605603	0.05	1
		Inverse variance weighted	0.643685381	0.05	1
		Simple mode	0.871734502	0.05	1
		Weighted mode	0.745092193	0.05	1
	IDP dMRI ProbtrackX ICVF ar r	MR Egger	0.91167658	0.05	1
		Weighted median	0.714121199	0.05	1
		Inverse variance weighted	0.65321563	0.05	1
		Simple mode	0.888937709	0.05	1
		Weighted mode	0.771081612	0.05	1
	IDP dMRI ProbtrackX ICVF atr l	MR Egger	0.823435951	0.05	1
		Weighted median	0.734379413	0.05	1
		Inverse variance weighted	0.874629477	0.05	1
		Simple mode	0.589770973	0.05	1
		Weighted mode	0.684875812	0.05	1
	IDP dMRI ProbtrackX ICVF atr r	MR Egger	0.812926603	0.05	1
		Weighted median	0.72304986	0.05	1
		Inverse variance weighted	0.874563637	0.05	1

		Simple mode	0.572358133	0.05	1
		Weighted mode	0.695342711	0.05	1
	IDP dMRI ProbtrackX ICVF cgc l	Wald ratio	0.740611284	0.05	1
	IDP dMRI ProbtrackX ICVF cgh l	MR Egger	0.810687879	0.05	1
		Weighted median	0.723498247	0.05	1
		Inverse variance weighted	0.866632122	0.05	1
		Simple mode	0.580109901	0.05	1
		Weighted mode	0.687082202	0.05	1
	IDP dMRI ProbtrackX ICVF cgh r	Wald ratio	0.706013693	0.05	1
	IDP dMRI ProbtrackX ICVF cst l	MR Egger	0.907896206	0.05	1
		Weighted median	0.694717858	0.05	1
		Inverse variance weighted	0.650509953	0.05	1
		Simple mode	0.789466252	0.05	1
		Weighted mode	0.77096791	0.05	1
	IDP dMRI ProbtrackX ICVF cst r	MR Egger	0.749908062	0.05	1
		Weighted median	0.545009369	0.05	1
		Inverse variance weighted	0.520173694	0.05	1
		Simple mode	0.546795036	0.05	1
		Weighted mode	0.702860667	0.05	1
	IDP dMRI ProbtrackX ICVF fma	Inverse variance weighted	0.199436719	0.05	1
	IDP dMRI ProbtrackX ICVF fmi	MR Egger	0.720117359	0.05	1
		Weighted median	0.823705283	0.05	1

		Inverse variance weighted	0.813718857	0.05	1
		Simple mode	0.404570685	0.05	1
		Weighted mode	0.808875911	0.05	1
	IDP dMRI ProbtrackX ICVF ifo l	Inverse variance weighted	0.870151559	0.05	1
	IDP dMRI ProbtrackX ICVF ifo r	Inverse variance weighted	0.865890483	0.05	1
	IDP dMRI ProbtrackX ICVF ilf l	Inverse variance weighted	0.852650712	0.05	1
	IDP dMRI ProbtrackX ICVF ilf r	Wald ratio	0.298438499	0.05	1
	IDP dMRI ProbtrackX ICVF mcp	Wald ratio	0.17512074	0.05	1
	IDP dMRI ProbtrackX ICVF ml l	Wald ratio	0.570112978	0.05	1
	IDP dMRI ProbtrackX ICVF ml r	Wald ratio	0.706013693	0.05	1
	IDP dMRI ProbtrackX ICVF ptr l	Wald ratio	0.298438499	0.05	1
	IDP dMRI ProbtrackX ICVF ptr r	Inverse variance weighted	0.13606809	0.05	1
	IDP dMRI ProbtrackX ICVF slf l	MR Egger	0.948649355	0.05	1
		Weighted median	0.873360747	0.05	1
		Inverse variance weighted	0.954438696	0.05	1
		Simple mode	0.618303957	0.05	1
		Weighted mode	0.743768984	0.05	1
	IDP dMRI ProbtrackX ICVF slf r	Inverse variance weighted	0.734689369	0.05	1
	IDP dMRI ProbtrackX ICVF str l	MR Egger	0.851562	0.05	1
		Weighted median	0.739780164	0.05	1
		Inverse variance weighted	0.909673482	0.05	1

		Simple mode	0.567816658	0.05	1
		Weighted mode	0.67538195	0.05	1
	IDP dMRI ProbtrackX ICVF str r	MR Egger	0.912003792	0.05	1
		Weighted median	0.824945815	0.05	1
		Inverse variance weighted	0.937904034	0.05	1
		Simple mode	0.598907099	0.05	1
		Weighted mode	0.726805446	0.05	1
	IDP dMRI ProbtrackX ICVF unc l	Inverse variance weighted	0.522122518	0.05	1
	IDP dMRI ProbtrackX ICVF unc r	Inverse variance weighted	0.937706171	0.05	1
	IDP dMRI ProbtrackX OD atr r	Wald ratio	0.848154258	0.05	1
	IDP dMRI ProbtrackX OD ilf l	Wald ratio	0.374967086	0.05	1
	IDP dMRI ProbtrackX OD slf l	Wald ratio	0.379073893	0.05	0.999986906
	IDP dMRI ProbtrackX OD str l	Inverse variance weighted	0.70050268	0.05	1
	IDP dMRI ProbtrackX ISOVF atr r	Wald ratio	0.706013693	0.05	1
	IDP dMRI ProbtrackX ISOVF cgc l	Wald ratio	0.832546935	0.05	1
	IDP dMRI ProbtrackX ISOVF ifo r	Wald ratio	0.55183033	0.05	1
	IDP dMRI ProbtrackX ISOVF ilf l	Wald ratio	0.171379311	0.05	1
	IDP dMRI ProbtrackX ISOVF ilf r	MR Egger	0.590721812	0.05	1
		Weighted median	0.788971731	0.05	1
		Inverse variance weighted	0.756805741	0.05	1
		Simple mode	0.807286431	0.05	1
		Weighted mode	0.816035635	0.05	1

	IDP dMRI ProbtrackX ISOVF slf l	Inverse variance weighted	0.767989038	0.05	1
	IDP dMRI ProbtrackX ISOVF slf r	MR Egger	0.40437322	0.05	1
		Weighted median	0.356728137	0.05	1
		Inverse variance weighted	0.692873322	0.05	1
		Simple mode	0.477795503	0.05	1
		Weighted mode	0.466120266	0.05	1
	IDP dMRI ProbtrackX ISOVF unc l	Wald ratio	0.387537595	0.05	1
	IDP dMRI ProbtrackX ISOVF unc r	Wald ratio	0.426198122	0.05	1
	IDP T1 SIENAX brain-normalised volume	Wald ratio	0.705366396	0.05	1
multiple sclerosis	IDP T1 SIENAX peripheral grey normalised volume	Wald ratio	0.967200003	0.05	1
	IDP T1 FAST ROIs R occ pole	Wald ratio	0.801000436	0.05	1
	IDP T1 FAST ROIs L thalamus	Wald ratio	0.914600162	0.05	1
	IDP T1 FAST ROIs R thalamus	Wald ratio	0.485100328	0.05	1
	IDP T1 FAST ROIs L putamen	Wald ratio	0.002734958	0.05	1
	IDP T1 FAST ROIs R putamen	Wald ratio	0.002734958	0.05	1
	IDP T1 FAST ROIs L pallidum	Wald ratio	0.641899544	0.05	1
	IDP T1 FIRST left caudate volume	Wald ratio	0.585097891	0.05	1
	IDP T1 FAST ROIs L hippocampus	Inverse variance weighted	0.981316055	0.05	1
	IDP T1 FAST ROIs R hippocampus	Wald ratio	0.443100323	0.05	1
	IDP T1 FAST ROIs L ventral striatum	Wald ratio	0.002734958	0.05	1
	IDP T1 FAST ROIs R ventral striatum	Inverse variance weighted	0.001632419	0.05	1
	IDP T1 FAST ROIs L cerebellum crus I	MR Egger	0.544755789	0.05	1

		Weighted median	0.049806028	0.05	1
		Inverse variance weighted	0.071134686	0.05	1
		Simple mode	0.20646184	0.05	1
		Weighted mode	0.222837269	0.05	1
	IDP T1 FAST ROIs R cerebellum crus I	Wald ratio	0.815499551	0.05	1
	IDP T1 FAST ROIs L cerebellum crus II	Inverse variance weighted	0.819027283	0.05	1
	IDP T1 FAST ROIs V cerebellum crus II	Inverse variance weighted	0.708584482	0.05	1
	IDP T1 FAST ROIs R cerebellum crus II	Wald ratio	0.378801158	0.05	1
	IDP T1 FAST ROIs V cerebellum VIIIa	Wald ratio	0.001945932	0.05	1
	IDP T1 FAST ROIs L cerebellum VIIIb	Wald ratio	0.002443968	0.05	1
	IDP T1 FAST ROIs V cerebellum VIIIb	Wald ratio	0.002443968	0.05	1
	IDP T1 FAST ROIs R cerebellum VIIIb	Wald ratio	0.002734958	0.05	1
	IDP T1 FAST ROIs L cerebellum IX	Wald ratio	0.002443968	0.05	1
	IDP T1 FAST ROIs V cerebellum IX	Inverse variance weighted	0.38804765	0.05	1
	IDP T1 FAST ROIs R cerebellum IX	Inverse variance weighted	0.31509406	0.05	1
	IDP T1 FAST ROIs V cerebellum X	Wald ratio	0.002734958	0.05	1
	IDP SWI T2star left caudate	MR Egger	0.471811693	0.05	1
		Weighted median	0.015763148	0.05	1
		Inverse variance weighted	0.000835472	0.05	1
		Simple mode	0.131143996	0.05	1
		Weighted mode	0.141571333	0.05	1
	IDP SWI T2star right caudate	MR Egger	0.886621701	0.05	1

		Weighted median	0.024354459	0.05	1
		Inverse variance weighted	0.043444139	0.05	1
		Simple mode	0.15324492	0.05	1
		Weighted mode	0.191417348	0.05	1
	IDP T1 FIRST left pallidum volume	Wald ratio	0.535600761	0.05	1
	IDP SWI T2star left putamen	MR Egger	0.382017095	0.05	1
		Weighted median	0.325835812	0.05	1
		Inverse variance weighted	0.367497594	0.05	1
		Simple mode	0.445309644	0.05	1
		Weighted mode	0.452044775	0.05	1
	IDP SWI T2star right putamen	MR Egger	0.666675702	0.05	1
		Weighted median	0.351700813	0.05	1
		Inverse variance weighted	0.135292032	0.05	1
		Simple mode	0.585407665	0.05	1
		Weighted mode	0.536841774	0.05	1
	IDP SWI T2star left pallidum	MR Egger	0.37442117	0.05	1
		Weighted median	0.829466275	0.05	1
		Inverse variance weighted	0.514190664	0.05	1
		Simple mode	0.77680849	0.05	1
		Weighted mode	0.738638597	0.05	1
	IDP SWI T2star right pallidum	MR Egger	0.923637246	0.05	1
		Weighted median	0.295641882	0.05	1

		Inverse variance weighted	0.206496648	0.05	1
		Simple mode	0.614544284	0.05	1
		Weighted mode	0.628663131	0.05	1
	IDP T1 FIRST right pallidum volume	Wald ratio	0.535600761	0.05	1
	IDP dMRI TBSS FA Genu of corpus callosum	Wald ratio	0.734400143	0.05	1
	IDP dMRI TBSS FA Splenium of corpus callosum	MR Egger	0.691494055	0.05	1
		Weighted median	0.522481022	0.05	1
		Inverse variance weighted	0.322509111	0.05	1
		Simple mode	0.689905368	0.05	1
		Weighted mode	0.718385539	0.05	1
	IDP dMRI TBSS FA Corticospinal tract L	Wald ratio	0.713800575	0.05	1
	IDP dMRI TBSS FA Superior cerebellar peduncle R	Inverse variance weighted	0.855861206	0.05	1
	IDP dMRI TBSS FA Superior cerebellar peduncle L	Inverse variance weighted	0.898236254	0.05	1
	IDP dMRI TBSS FA Posterior limb of internal capsule R	Wald ratio	0.004303967	0.05	1
	IDP dMRI TBSS FA Retrolenticular part of internal capsule R	Inverse variance weighted	0.67588548	0.05	1
	IDP dMRI TBSS FA Retrolenticular part of internal capsule L	Wald ratio	0.817299967	0.05	1
	IDP dMRI TBSS FA Anterior corona radiata R	Wald ratio	0.734400143	0.05	1
	IDP dMRI TBSS FA Anterior corona radiata L	Wald ratio	0.734400143	0.05	1
	IDP dMRI TBSS FA Posterior corona radiata L	Wald ratio	0.817299967	0.05	1
	IDP dMRI TBSS FA Posterior thalamic radiation R	Inverse variance weighted	0.684178456	0.05	1

	IDP dMRI TBSS FA Posterior thalamic radiation L	Inverse variance weighted	0.681346408	0.05	1
	IDP dMRI TBSS FA Sagittal stratum R	Inverse variance weighted	0.325581551	0.05	1
	IDP dMRI TBSS FA Sagittal stratum L	Wald ratio	0.722300433	0.05	1
	IDP dMRI TBSS FA Cingulum cingulate gyrus R	Wald ratio	0.734400143	0.05	1
	IDP dMRI TBSS FA Superior longitudinal fasciculus L	Inverse variance weighted	0.846926429	0.05	1
	IDP dMRI TBSS FA Uncinate fasciculus L	Wald ratio	0.862399928	0.05	1
	IDP dMRI TBSS MD Genu of corpus callosum	Wald ratio	0.734400143	0.05	1
	IDP dMRI TBSS MD Body of corpus callosum	Wald ratio	0.726198808	0.05	1
	IDP dMRI TBSS MD Splenium of corpus callosum	Inverse variance weighted	0.720031628	0.05	1
	IDP dMRI TBSS MD Corticospinal tract R	Wald ratio	0.612600885	0.05	1
	IDP dMRI TBSS MD Inferior cerebellar peduncle R	Inverse variance weighted	0.980937776	0.05	1
	IDP dMRI TBSS MD Inferior cerebellar peduncle L	Wald ratio	0.817299967	0.05	1
	IDP dMRI TBSS MD Superior cerebellar peduncle L	Wald ratio	0.751000131	0.05	1
	IDP dMRI TBSS MD Anterior limb of internal capsule L	Wald ratio	0.770799464	0.05	1
	IDP dMRI TBSS MD Retrolenticular part of internal capsule R	Inverse variance weighted	0.664052124	0.05	1
	IDP T1 FIRST left caudate volume plus IDP T1 FIRST right caudate volume	Wald ratio	0.762299797	0.05	1
	IDP T1 FIRST left pallidum volume plus IDP T1 FIRST right pallidum volume	Wald ratio	0.535600761	0.05	1
	IDP SWI T2star left thalamus plus IDP SWI T2star right thalamus	Inverse variance weighted	0.512911605	0.05	1
	IDP dMRI TBSS MD Retrolenticular part of internal capsule L	Inverse variance weighted	0.669108483	0.05	1

	IDP SWI T2star left caudate plus IDP SWI T2star right caudate	MR Egger	0.654573172	0.05	1
		Weighted median	0.047214594	0.05	1
		Inverse variance weighted	0.024529457	0.05	1
		Simple mode	0.164322204	0.05	1
		Weighted mode	0.125893003	0.05	1
	IDP SWI T2star left putamen plus IDP SWI T2star right putamen	MR Egger	0.255887374	0.05	1
		Weighted median	0.361722548	0.05	1
		Inverse variance weighted	0.313138619	0.05	1
		Simple mode	0.627055276	0.05	1
		Weighted mode	0.533797486	0.05	1
	IDP SWI T2star left pallidum plus IDP SWI T2star right pallidum	MR Egger	0.440821417	0.05	1
		Weighted median	0.311986105	0.05	1
		Inverse variance weighted	0.104158537	0.05	1
		Simple mode	0.764059924	0.05	1
		Weighted mode	0.738239122	0.05	1
	volume Left-Lateral-Ventricle	MR Egger	0.438933931	0.05	1
		Weighted median	0.007472909	0.05	1
		Inverse variance weighted	0.038823378	0.05	1
		Simple mode	0.142005368	0.05	1
		Weighted mode	0.141101785	0.05	1
	volume Left-Cerebellum-White-Matter	Inverse variance weighted	0.182324839	0.05	1
	volume Left-Cerebellum-Cortex	Wald ratio	0.815499551	0.05	1

	IDP dMRI TBSS MD Anterior corona radiata R	Inverse variance weighted	0.608518028	0.05	1
	volume Left-Putamen	Wald ratio	0.712599798	0.05	1
	volume 3rd-Ventricle	Wald ratio	0.042169875	0.05	1
	volume 4th-Ventricle	Inverse variance weighted	0.761403368	0.05	1
	volume Brain-Stem	Inverse variance weighted	0.450112195	0.05	1
	volume Left-Hippocampus	Wald ratio	0.148800187	0.05	1
	volume CSF	MR Egger	0.875423702	0.05	1
		Weighted median	0.038156378	0.05	1
		Inverse variance weighted	0.631457017	0.05	1
		Simple mode	0.144390062	0.05	1
		Weighted mode	0.14667453	0.05	1
	IDP dMRI TBSS MD Anterior corona radiata L	Inverse variance weighted	0.611648001	0.05	1
	volume Left-Accumbens-area	Wald ratio	0.002734958	0.05	1
	volume Right-Lateral-Ventricle	Inverse variance weighted	0.355334961	0.05	1
	volume Right-Cerebellum-White-Matter	Wald ratio	0.202798806	0.05	1
	volume Right-Cerebellum-Cortex	MR Egger	0.69070658	0.05	1
		Weighted median	0.313071159	0.05	1
		Inverse variance weighted	0.335162252	0.05	1
		Simple mode	0.473681516	0.05	1
		Weighted mode	0.437994532	0.05	1
	IDP dMRI TBSS MD Superior corona radiata R	Inverse variance weighted	0.608572671	0.05	1

	volume Right-Hippocampus	Wald ratio	0.443100323	0.05	1
	volume CC Posterior	Wald ratio	0.382399842	0.05	1
	IDP dMRI TBSS MD Superior corona radiata L	Inverse variance weighted	0.608871184	0.05	1
	IDP dMRI TBSS MD Posterior corona radiata R	Inverse variance weighted	0.601511831	0.05	1
	IDP dMRI TBSS MD Posterior corona radiata L	Inverse variance weighted	0.683698359	0.05	1
	volume rhSurfaceHoles	Wald ratio	0.588201031	0.05	1
	DKTatlas lh cuneus area	Wald ratio	0.474801009	0.05	1
	IDP dMRI TBSS MD Posterior thalamic radiation R	Inverse variance weighted	0.677844655	0.05	1
	DKTatlas lh lateraloccipital area	Wald ratio	0.474801009	0.05	1
	IDP dMRI TBSS MD Posterior thalamic radiation L	Inverse variance weighted	0.599926895	0.05	1
	DKTatlas lh parstriangularis area	Wald ratio	0.607499312	0.05	1
	DKTatlas lh pericalcarine area	Wald ratio	0.149598865	0.05	1
	DKTatlas lh postcentral area	Wald ratio	0.293999916	0.05	1
	DKTatlas lh posteriorcingulate area	Wald ratio	0.572600047	0.05	1
	DKTatlas lh precentral area	Wald ratio	0.2760999	0.05	1
	DKTatlas lh precuneus area	Wald ratio	0.440899711	0.05	1
	IDP dMRI TBSS MD Sagittal stratum R	Inverse variance weighted	0.598790523	0.05	1
	DKTatlas lh supramarginal area	Wald ratio	0.535000631	0.05	1
	DKTatlas lh WhiteSurfArea area	Wald ratio	0.227701799	0.05	1
	IDP dMRI TBSS MD Sagittal stratum L	Inverse variance weighted	0.606619201	0.05	1
	a2009s lh G&S subcentral area	Wald ratio	0.2760999	0.05	1
	a2009s lh G front inf-Opercular area	Wald ratio	0.230299358	0.05	1

	IDP dMRI TBSS MD External capsule R	Wald ratio	0.734400143	0.05	1
	IDP dMRI TBSS MD External capsule L	Inverse variance weighted	0.666969537	0.05	1
	a2009s lh G pariet inf-Supramar area	Wald ratio	0.535000631	0.05	1
	a2009s lh G parietal sup area	Wald ratio	0.280999168	0.05	1
	a2009s lh G postcentral area	Wald ratio	0.293999916	0.05	1
	a2009s lh G precentral area	Wald ratio	0.2760999	0.05	1
	IDP dMRI TBSS MD Cingulum cingulate gyrus R	MR Egger	0.998207419	0.05	1
		Weighted median	0.610645223	0.05	1
		Inverse variance weighted	0.567473643	0.05	1
		Simple mode	0.721673825	0.05	1
		Weighted mode	0.729545344	0.05	1
	IDP dMRI TBSS MD Cingulum cingulate gyrus L	MR Egger	0.997636341	0.05	1
		Weighted median	0.627261536	0.05	1
		Inverse variance weighted	0.56769265	0.05	1
		Simple mode	0.723932593	0.05	1
		Weighted mode	0.744242028	0.05	1
	a2009s lh S central area	Wald ratio	0.339998968	0.05	1
	a2009s lh S collat transv ant area	Wald ratio	0.868400113	0.05	1
	IDP dMRI TBSS MD Cingulum hippocampus R	Inverse variance weighted	0.606036613	0.05	1
	a2009s lh S intrapariet&P trans area	Wald ratio	0.280999168	0.05	1
	IDP dMRI TBSS MD Cingulum hippocampus L	Inverse variance weighted	0.607673843	0.05	1
	DKTatlas rh parstriangularis area	Wald ratio	0.414898807	0.05	1

	DKTatlas rh pericalcarine area	Wald ratio	0.583799415	0.05	1
	IDP dMRI TBSS MD Superior longitudinal fasciculus R	Inverse variance weighted	0.663247034	0.05	1
	DKTatlas rh postcentral area	Wald ratio	0.339998968	0.05	1
	DKTatlas rh precentral area	Wald ratio	0.2760999	0.05	1
	IDP dMRI TBSS MD Superior longitudinal fasciculus L	Inverse variance weighted	0.597892437	0.05	1
	a2009s rh G&S subcentral area	Wald ratio	0.339998968	0.05	1
	a2009s rh G&S cingul-Mid-Post area	Wald ratio	0.785600387	0.05	1
	IDP dMRI TBSS MD Superior fronto-occipital fasciculus L	Wald ratio	0.007547002	0.05	1
	a2009s rh G parietal sup area	Wald ratio	0.171299296	0.05	1
	IDP dMRI TBSS MD Uncinate fasciculus R	Inverse variance weighted	0.732970539	0.05	1
	a2009s rh G precentral area	Wald ratio	0.2760999	0.05	1
	a2009s rh G precuneus area	Wald ratio	0.766499568	0.05	1
	IDP dMRI TBSS MD Uncinate fasciculus L	Inverse variance weighted	0.667324817	0.05	1
	a2009s rh S central area	Wald ratio	0.2760999	0.05	1
	a2009s rh S collat transv ant area	Wald ratio	0.868400113	0.05	1
	a2009s rh S orbital med-olfact area	Wald ratio	0.735899833	0.05	1
	IDP dMRI TBSS MO Pontine crossing tract	MR Egger	0.611608443	0.05	1
		Weighted median	0.628217452	0.05	1
		Inverse variance weighted	0.692641962	0.05	1
		Simple mode	0.821565517	0.05	1
		Weighted mode	0.754311311	0.05	1
	DKTatlas lh postcentral thickness	Wald ratio	0.339998968	0.05	1

	a2009s lh G insular short thickness	Wald ratio	0.284900338	0.05	1
	a2009s lh G postcentral thickness	Wald ratio	0.339998968	0.05	1
	IDP T1 SIENAX CSF normalised volume	Inverse variance weighted	0.000477618	0.05	1
	IDP dMRI TBSS MO Medial lemniscus R	Wald ratio	0.705300308	0.05	1
	DKTatlas rh lateraloccipital thickness	Wald ratio	0.002734958	0.05	1
	DKTatlas rh postcentral thickness	Wald ratio	0.339998968	0.05	1
	a2009s rh G cuneus thickness	Wald ratio	0.002734958	0.05	1
	a2009s rh G postcentral thickness	Wald ratio	0.282300479	0.05	1
	a2009s rh Pole occipital thickness	Wald ratio	0.002734958	0.05	1
	a2009s rh S circular insula ant thickness	Wald ratio	0.842999837	0.05	1
	a2009s rh S oc sup&transversal thickness	Wald ratio	0.532797768	0.05	1
	IDP dMRI TBSS MO Anterior corona radiata R	Wald ratio	0.776000159	0.05	1
	IDP dMRI TBSS MO Superior corona radiata R	Wald ratio	0.955900134	0.05	1
	IDP dMRI TBSS MO Cingulum cingulate gyrus L	Inverse variance weighted	0.566354719	0.05	1
	IDP dMRI TBSS L1 Genu of corpus callosum	Wald ratio	0.722300433	0.05	1
	IDP dMRI TBSS L1 Anterior limb of internal capsule L	Wald ratio	0.770799464	0.05	1
	IDP dMRI TBSS L1 Posterior limb of internal capsule L	Wald ratio	0.147201541	0.05	1
	IDP dMRI TBSS L1 Anterior corona radiata R	Wald ratio	0.734400143	0.05	1
	IDP dMRI TBSS L1 Anterior corona radiata L	Wald ratio	0.734400143	0.05	1
	IDP dMRI TBSS L1 Posterior corona radiata R	Wald ratio	0.434000032	0.05	1

	IDP dMRI TBSS L1 Posterior corona radiata L	Wald ratio	0.734400143	0.05	1
	IDP dMRI TBSS L1 Sagittal stratum L	Wald ratio	0.734400143	0.05	1
	IDP dMRI TBSS L1 External capsule R	Wald ratio	0.734400143	0.05	1
	IDP dMRI TBSS L1 External capsule L	MR Egger	0.502109555	0.05	1
		Weighted median	0.993788722	0.05	1
		Inverse variance weighted	0.395114701	0.05	1
		Simple mode	0.900217132	0.05	1
		Weighted mode	0.900383552	0.05	1
	IDP dMRI TBSS L1 Cingulum hippocampus L	Wald ratio	0.722300433	0.05	1
	IDP dMRI TBSS L1 Fornix cres+Stria terminalis L	Wald ratio	0.200800554	0.05	1
	IDP T1 FAST ROIs L precentral gyrus	Wald ratio	0.339998968	0.05	1
	IDP dMRI TBSS L1 Superior longitudinal fasciculus R	Inverse variance weighted	0.172602504	0.05	1
	IDP dMRI TBSS L1 Uncinate fasciculus R	Wald ratio	0.817299967	0.05	1
	IDP dMRI TBSS L1 Uncinate fasciculus L	Wald ratio	0.815899756	0.05	1
	IDP dMRI TBSS L2 Pontine crossing tract	Wald ratio	0.705300308	0.05	1
	IDP dMRI TBSS L2 Genu of corpus callosum	MR Egger	0.819784536	0.05	1
		Weighted median	0.615567905	0.05	1
		Inverse variance weighted	0.819713385	0.05	1
		Simple mode	0.665979408	0.05	1
		Weighted mode	0.668024353	0.05	1
	IDP dMRI TBSS L2 Body of corpus callosum	Wald ratio	0.734400143	0.05	1
		MR Egger	0.678428627	0.05	1

	IDP dMRI TBSS L2 Splenium of corpus callosum	Weighted median	0.428326768	0.05	1
		Inverse variance weighted	0.280337041	0.05	1
		Simple mode	0.628442239	0.05	1
		Weighted mode	0.656800496	0.05	1
	IDP dMRI TBSS L2 Corticospinal tract R	Wald ratio	0.612600885	0.05	1
	IDP T1 SIENAX CSF unnormalised volume	MR Egger	0.342740249	0.05	1
		Weighted median	0.004741697	0.05	1
		Inverse variance weighted	0.028910193	0.05	1
		Simple mode	0.143873541	0.05	1
		Weighted mode	0.135583847	0.05	1
	IDP T1 FAST ROIs L temporal pole	Wald ratio	0.958099744	0.05	1
	IDP dMRI TBSS L2 Superior cerebellar peduncle R	Inverse variance weighted	0.870121597	0.05	1
	IDP dMRI TBSS L2 Superior cerebellar peduncle L	Wald ratio	0.751000131	0.05	1
	IDP dMRI TBSS L2 Posterior limb of internal capsule R	Inverse variance weighted	0.506540135	0.05	1
	IDP dMRI TBSS L2 Posterior limb of internal capsule L	Wald ratio	0.631200835	0.05	1
	IDP dMRI TBSS L2 Retrolenticular part of internal capsule R	Inverse variance weighted	0.604062234	0.05	1
	IDP dMRI TBSS L2 Retrolenticular part of internal capsule L	Wald ratio	0.817299967	0.05	1
	IDP dMRI TBSS L2 Anterior corona radiata R	Inverse variance weighted	0.608955947	0.05	1
	IDP dMRI TBSS L2 Anterior corona radiata L	Wald ratio	0.734400143	0.05	1
	IDP dMRI TBSS L2 Superior corona radiata R	MR Egger	0.749067984	0.05	1
		Weighted median	0.613237609	0.05	1

		Inverse variance weighted	0.721881711	0.05	1
		Simple mode	0.687221711	0.05	1
		Weighted mode	0.663135912	0.05	1
	IDP dMRI TBSS L2 Superior corona radiata L	Inverse variance weighted	0.597049768	0.05	1
	IDP dMRI TBSS L2 Posterior corona radiata R	Inverse variance weighted	0.67344587	0.05	1
	IDP dMRI TBSS L2 Posterior corona radiata L	Inverse variance weighted	0.675194684	0.05	1
	IDP dMRI TBSS L2 Posterior thalamic radiation R	Inverse variance weighted	0.678149726	0.05	1
	IDP dMRI TBSS L2 Posterior thalamic radiation L	Inverse variance weighted	0.677699161	0.05	1
	IDP dMRI TBSS L2 Sagittal stratum R	Inverse variance weighted	0.664623247	0.05	1
	IDP dMRI TBSS L2 Sagittal stratum L	Wald ratio	0.722300433	0.05	1
	IDP dMRI TBSS L2 Cingulum cingulate gyrus R	Wald ratio	0.734400143	0.05	1
	IDP dMRI TBSS L2 Cingulum cingulate gyrus L	Wald ratio	0.722300433	0.05	1
	IDP dMRI TBSS L2 Cingulum hippocampus R	Inverse variance weighted	0.602607425	0.05	1
	IDP dMRI TBSS L2 Cingulum hippocampus L	Wald ratio	0.734400143	0.05	1
	IDP dMRI TBSS L2 Uncinate fasciculus L	Wald ratio	0.734400143	0.05	1
	IDP dMRI TBSS L3 Middle cerebellar peduncle	Wald ratio	0.817299967	0.05	1
	IDP dMRI TBSS L3 Genu of corpus callosum	Wald ratio	0.734400143	0.05	1
	IDP dMRI TBSS L3 Body of corpus callosum	Wald ratio	0.734400143	0.05	1
	IDP dMRI TBSS L3 Splenium of corpus callosum	Inverse variance weighted	0.601840677	0.05	1

	IDP dMRI TBSS L3 Inferior cerebellar peduncle R	Wald ratio	0.734400143	0.05	1
	IDP dMRI TBSS L3 Inferior cerebellar peduncle L	Wald ratio	0.734400143	0.05	1
	IDP dMRI TBSS L3 Superior cerebellar peduncle R	Inverse variance weighted	0.888327173	0.05	1
	IDP dMRI TBSS L3 Superior cerebellar peduncle L	Wald ratio	0.751000131	0.05	1
	IDP dMRI TBSS L3 Anterior limb of internal capsule R	Wald ratio	0.060769219	0.05	1
	IDP dMRI TBSS L3 Anterior limb of internal capsule L	Wald ratio	0.028710167	0.05	1
	IDP dMRI TBSS L3 Retrolenticular part of internal capsule R	Inverse variance weighted	0.666132579	0.05	1
	IDP dMRI TBSS L3 Retrolenticular part of internal capsule L	Wald ratio	0.817299967	0.05	1
	IDP dMRI TBSS L3 Anterior corona radiata R	Inverse variance weighted	0.608816659	0.05	1
	IDP dMRI TBSS L3 Anterior corona radiata L	Inverse variance weighted	0.612483683	0.05	1
	IDP dMRI TBSS L3 Superior corona radiata R	Wald ratio	0.722300433	0.05	1
	IDP dMRI TBSS L3 Superior corona radiata L	Wald ratio	0.722300433	0.05	1
	IDP dMRI TBSS L3 Posterior corona radiata R	Wald ratio	0.734400143	0.05	1
	IDP dMRI TBSS L3 Posterior corona radiata L	Wald ratio	0.817299967	0.05	1
	IDP dMRI TBSS L3 Posterior thalamic radiation R	Inverse variance weighted	0.685206147	0.05	1
	IDP dMRI TBSS L3 Posterior thalamic radiation L	Inverse variance weighted	0.601700737	0.05	1
	IDP dMRI TBSS L3 Sagittal stratum R	Inverse variance weighted	0.60145087	0.05	1
	IDP dMRI TBSS L3 Sagittal stratum L	Inverse variance weighted	0.609027855	0.05	1

	IDP dMRI TBSS L3 External capsule R	Wald ratio	0.734400143	0.05	1
	IDP dMRI TBSS L3 External capsule L	Wald ratio	0.734400143	0.05	1
	IDP dMRI TBSS L3 Cingulum cingulate gyrus R	Inverse variance weighted	0.611211004	0.05	1
	IDP dMRI TBSS L3 Cingulum cingulate gyrus L	Wald ratio	0.734400143	0.05	1
	IDP dMRI TBSS L3 Cingulum hippocampus R	Wald ratio	0.734400143	0.05	1
	IDP dMRI TBSS L3 Cingulum hippocampus L	Wald ratio	0.734400143	0.05	1
	IDP dMRI TBSS L3 Fornix cres+Stria terminalis R	Wald ratio	0.050160123	0.05	1
	IDP dMRI TBSS L3 Superior longitudinal fasciculus R	Inverse variance weighted	0.664787034	0.05	1
	IDP dMRI TBSS L3 Superior longitudinal fasciculus L	Inverse variance weighted	0.598618881	0.05	1
	IDP dMRI TBSS L3 Uncinate fasciculus R	Wald ratio	0.734400143	0.05	1
	IDP dMRI TBSS L3 Uncinate fasciculus L	Inverse variance weighted	0.734816855	0.05	1
	IDP dMRI TBSS ICVF Middle cerebellar peduncle	Inverse variance weighted	0.680494135	0.05	1
	IDP dMRI TBSS ICVF Genu of corpus callosum	MR Egger	0.613132812	0.05	1
		Weighted median	0.505836713	0.05	1
		Inverse variance weighted	0.276548424	0.05	1
		Simple mode	0.61371536	0.05	1
		Weighted mode	0.569468403	0.05	1
	IDP dMRI TBSS ICVF Body of corpus callosum	MR Egger	0.755436416	0.05	1
		Weighted median	0.512966723	0.05	1
		Inverse variance weighted	0.698501525	0.05	1

		Simple mode	0.585227039	0.05	1
		Weighted mode	0.58287505	0.05	1
	IDP dMRI TBSS ICVF Splenium of corpus callosum	MR Egger	0.445998214	0.05	1
		Weighted median	0.61832379	0.05	1
		Inverse variance weighted	0.40083382	0.05	1
		Simple mode	0.904226015	0.05	1
		Weighted mode	0.707702846	0.05	1
	IDP dMRI TBSS ICVF Fornix	Wald ratio	0.007547002	0.05	1
	IDP dMRI TBSS ICVF Medial lemniscus R	Inverse variance weighted	0.634969399	0.05	1
	IDP dMRI TBSS ICVF Medial lemniscus L	Wald ratio	0.634500314	0.05	1
	IDP dMRI TBSS ICVF Inferior cerebellar peduncle R	MR Egger	0.74911757	0.05	1
		Weighted median	0.651511564	0.05	1
		Inverse variance weighted	0.361281908	0.05	1
		Simple mode	0.76738814	0.05	1
		Weighted mode	0.781096064	0.05	1
	IDP dMRI TBSS ICVF Inferior cerebellar peduncle L	MR Egger	0.686144566	0.05	1
		Weighted median	0.637186585	0.05	1
		Inverse variance weighted	0.341106469	0.05	1
		Simple mode	0.766475235	0.05	1
		Weighted mode	0.75101764	0.05	1
	IDP dMRI TBSS ICVF Superior cerebellar peduncle R	Inverse variance weighted	0.985387153	0.05	1
	IDP dMRI TBSS ICVF Superior cerebellar peduncle L	Inverse variance weighted	0.939305696	0.05	1

	IDP dMRI TBSS ICVF Cerebral peduncle R	MR Egger	0.282648986	0.05	1
		Weighted median	0.432702324	0.05	1
		Inverse variance weighted	0.445558349	0.05	1
		Simple mode	0.771646646	0.05	1
		Weighted mode	0.911065041	0.05	1
	IDP dMRI TBSS ICVF Cerebral peduncle L	Inverse variance weighted	0.372245501	0.05	1
	IDP dMRI TBSS ICVF Anterior limb of internal capsule R	Wald ratio	0.734400143	0.05	1
	IDP dMRI TBSS ICVF Anterior limb of internal capsule L	MR Egger	0.681835408	0.05	1
		Weighted median	0.826460787	0.05	1
		Inverse variance weighted	0.434380458	0.05	1
		Simple mode	0.802442135	0.05	1
		Weighted mode	0.991285994	0.05	1
	IDP dMRI TBSS ICVF Posterior limb of internal capsule R	MR Egger	0.949638202	0.05	1
		Weighted median	0.616083854	0.05	1
		Inverse variance weighted	0.720723098	0.05	1
		Simple mode	0.579258321	0.05	1
		Weighted mode	0.702359177	0.05	1
	IDP dMRI TBSS ICVF Posterior limb of internal capsule L	MR Egger	0.758684255	0.05	1
		Weighted median	0.652081012	0.05	1
		Inverse variance weighted	0.833593292	0.05	1
		Simple mode	0.708387985	0.05	1
		Weighted mode	0.709894845	0.05	1

	IDP dMRI TBSS ICVF Retrolenticular part of internal capsule R	MR Egger	0.774065692	0.05	1
		Weighted median	0.575065561	0.05	1
		Inverse variance weighted	0.764746586	0.05	1
		Simple mode	0.507535275	0.05	1
		Weighted mode	0.591211394	0.05	1
	IDP dMRI TBSS ICVF Retrolenticular part of internal capsule L	MR Egger	0.710050975	0.05	1
		Weighted median	0.650538941	0.05	1
		Inverse variance weighted	0.792452592	0.05	1
		Simple mode	0.678088623	0.05	1
		Weighted mode	0.710499208	0.05	1
	IDP dMRI TBSS ICVF Anterior corona radiata R	MR Egger	0.700146304	0.05	1
		Weighted median	0.638871819	0.05	1
		Inverse variance weighted	0.806409401	0.05	1
		Simple mode	0.687049902	0.05	1
		Weighted mode	0.705212603	0.05	1
	IDP dMRI TBSS ICVF Anterior corona radiata L	MR Egger	0.728169596	0.05	1
		Weighted median	0.653078602	0.05	1
		Inverse variance weighted	0.808333809	0.05	1
		Simple mode	0.710856795	0.05	1
		Weighted mode	0.711650589	0.05	1
	IDP dMRI TBSS ICVF Superior corona radiata R	MR Egger	0.669581338	0.05	1
		Weighted median	0.636371952	0.05	1

		Inverse variance weighted	0.801039294	0.05	1
		Simple mode	0.720987173	0.05	1
		Weighted mode	0.685931968	0.05	1
	IDP dMRI TBSS ICVF Superior corona radiata L	MR Egger	0.688745768	0.05	1
		Weighted median	0.640418992	0.05	1
		Inverse variance weighted	0.803737351	0.05	1
		Simple mode	0.708566161	0.05	1
		Weighted mode	0.709048437	0.05	1
	IDP dMRI TBSS ICVF Posterior corona radiata R	Inverse variance weighted	0.615044112	0.05	1
	IDP dMRI TBSS ICVF Posterior corona radiata L	Inverse variance weighted	0.615694683	0.05	1
	IDP dMRI TBSS ICVF Posterior thalamic radiation R	Inverse variance weighted	0.680640449	0.05	1
	IDP dMRI TBSS ICVF Posterior thalamic radiation L	MR Egger	0.429872007	0.05	1
		Weighted median	0.662490567	0.05	1
		Inverse variance weighted	0.342531916	0.05	1
		Simple mode	0.759350121	0.05	1
		Weighted mode	0.741764345	0.05	1
	IDP dMRI TBSS ICVF Sagittal stratum R	MR Egger	0.491237761	0.05	1
		Weighted median	0.590061648	0.05	1
		Inverse variance weighted	0.33249065	0.05	1
		Simple mode	0.679338237	0.05	1
		Weighted mode	0.66759375	0.05	1
	IDP dMRI TBSS ICVF Sagittal stratum L	MR Egger	0.506722233	0.05	1

		Weighted median	0.597183727	0.05	1
		Inverse variance weighted	0.3992639	0.05	1
		Simple mode	0.695875679	0.05	1
		Weighted mode	0.681177289	0.05	1
	IDP dMRI TBSS ICVF External capsule R	Inverse variance weighted	0.672994456	0.05	1
	IDP dMRI TBSS ICVF External capsule L	Inverse variance weighted	0.661453426	0.05	1
	IDP dMRI TBSS ICVF Cingulum cingulate gyrus R	Inverse variance weighted	0.624265183	0.05	1
	IDP dMRI TBSS ICVF Cingulum cingulate gyrus L	Wald ratio	0.722300433	0.05	1
	IDP dMRI TBSS ICVF Cingulum hippocampus R	MR Egger	0.773938781	0.05	1
		Weighted median	0.676136556	0.05	1
		Inverse variance weighted	0.769104342	0.05	1
		Simple mode	0.686498779	0.05	1
		Weighted mode	0.717098271	0.05	1
	IDP dMRI TBSS ICVF Cingulum hippocampus L	MR Egger	0.729259707	0.05	1
		Weighted median	0.648609551	0.05	1
		Inverse variance weighted	0.789879087	0.05	1
		Simple mode	0.697752009	0.05	1
		Weighted mode	0.709188725	0.05	1
	IDP dMRI TBSS ICVF Fornix cres+Stria terminalis R	Inverse variance weighted	0.709323791	0.05	1
	IDP dMRI TBSS ICVF Fornix cres+Stria terminalis L	Wald ratio	0.722300433	0.05	1
		MR Egger	0.627372047	0.05	1

	IDP dMRI TBSS ICVF Superior longitudinal fasciculus R	Weighted median	0.617876272	0.05	1
		Inverse variance weighted	0.786474236	0.05	1
		Simple mode	0.699560325	0.05	1
		Weighted mode	0.702810127	0.05	1
	IDP dMRI TBSS ICVF Superior longitudinal fasciculus L	MR Egger	0.638107868	0.05	1
		Weighted median	0.634938317	0.05	1
		Inverse variance weighted	0.783751043	0.05	1
		Simple mode	0.714334758	0.05	1
		Weighted mode	0.703639468	0.05	1
	IDP dMRI TBSS ICVF Superior fronto-occipital fasciculus R	Inverse variance weighted	0.550019968	0.05	1
	IDP dMRI TBSS ICVF Superior fronto-occipital fasciculus L	Wald ratio	0.722300433	0.05	1
	IDP dMRI TBSS ICVF Uncinate fasciculus R	Wald ratio	0.734400143	0.05	1
	IDP dMRI TBSS ICVF Uncinate fasciculus L	Wald ratio	0.734400143	0.05	1
	IDP dMRI TBSS ICVF Tapetum R	Wald ratio	0.817299967	0.05	1
	IDP dMRI TBSS ICVF Tapetum L	Wald ratio	0.817299967	0.05	1
	IDP dMRI TBSS OD Pontine crossing tract	Inverse variance weighted	0.463993439	0.05	1
	IDP dMRI TBSS OD Posterior limb of internal capsule L	Wald ratio	0.147201541	0.05	1
	IDP dMRI TBSS OD External capsule R	Wald ratio	0.963199939	0.05	1
	IDP dMRI TBSS OD Superior longitudinal fasciculus R	Wald ratio	0.00625605	0.05	1
	IDP dMRI TBSS ISOVF Fornix	Wald ratio	0.514400325	0.05	1
	IDP dMRI TBSS ISOVF External capsule R	Wald ratio	0.34109928	0.05	1

	IDP dMRI TBSS ISOVF Cingulum cingulate gyrus R	Inverse variance weighted	0.876452372	0.05	1
	IDP dMRI ProbtrackX FA ar l	Wald ratio	0.734400143	0.05	1
	IDP dMRI ProbtrackX FA atr l	Wald ratio	0.734400143	0.05	1
	IDP dMRI ProbtrackX FA atr r	Wald ratio	0.817299967	0.05	1
	IDP dMRI ProbtrackX FA fma	Wald ratio	0.817299967	0.05	1
	IDP dMRI ProbtrackX FA fmi	MR Egger	0.872507164	0.05	1
		Weighted median	0.566115837	0.05	1
		Inverse variance weighted	0.664238035	0.05	1
		Simple mode	0.596297403	0.05	1
		Weighted mode	0.656160731	0.05	1
	IDP dMRI ProbtrackX FA ifo l	Inverse variance weighted	0.681721641	0.05	1
	IDP dMRI ProbtrackX FA ifo r	Inverse variance weighted	0.678209093	0.05	1
	IDP dMRI ProbtrackX FA ilf l	Wald ratio	0.817299967	0.05	1
	IDP dMRI ProbtrackX FA ilf r	Wald ratio	0.817299967	0.05	1
	IDP dMRI ProbtrackX FA ml l	Wald ratio	0.340600785	0.05	1
	IDP dMRI ProbtrackX FA ptr l	Wald ratio	0.722300433	0.05	1
	IDP dMRI ProbtrackX FA ptr r	Wald ratio	0.80690005	0.05	1
	IDP dMRI ProbtrackX FA slf l	Inverse variance weighted	0.66243468	0.05	1
	IDP dMRI ProbtrackX FA slf r	Wald ratio	0.817299967	0.05	1
	IDP dMRI ProbtrackX FA str l	Wald ratio	0.307399432	0.05	1
	IDP dMRI ProbtrackX FA unc l	Wald ratio	0.734400143	0.05	1
	IDP dMRI ProbtrackX FA unc r	Wald ratio	0.734400143	0.05	1

	IDP dMRI ProbtrackX MD ar l	Wald ratio	0.734400143	0.05	1
	IDP dMRI ProbtrackX MD ar r	Wald ratio	0.734400143	0.05	1
	IDP dMRI ProbtrackX MD atr l	Wald ratio	0.734400143	0.05	1
	IDP dMRI ProbtrackX MD atr r	Wald ratio	0.734400143	0.05	1
	IDP dMRI ProbtrackX MD cgc l	MR Egger	0.996380692	0.05	1
		Weighted median	0.618878967	0.05	1
		Inverse variance weighted	0.56310736	0.05	1
		Simple mode	0.720018455	0.05	1
		Weighted mode	0.743715625	0.05	1
	IDP dMRI ProbtrackX MD cgc r	MR Egger	0.994103414	0.05	1
		Weighted median	0.622028568	0.05	1
		Inverse variance weighted	0.560556378	0.05	1
		Simple mode	0.716541544	0.05	1
		Weighted mode	0.730062677	0.05	1
	IDP dMRI ProbtrackX MD fmi	Inverse variance weighted	0.611357959	0.05	1
	IDP dMRI ProbtrackX MD ifo l	Inverse variance weighted	0.602404428	0.05	1
	IDP dMRI ProbtrackX MD ifo r	Inverse variance weighted	0.601092381	0.05	1
	IDP dMRI ProbtrackX MD ilf l	Inverse variance weighted	0.602086026	0.05	1
	IDP dMRI ProbtrackX MD ilf r	Inverse variance weighted	0.605695324	0.05	1
	IDP dMRI ProbtrackX MD ptr l	Wald ratio	0.734400143	0.05	1
	IDP dMRI ProbtrackX MD ptr r	Wald ratio	0.734400143	0.05	1

	IDP dMRI ProbtrackX MD slf l	Inverse variance weighted	0.609341295	0.05	1
	IDP dMRI ProbtrackX MD slf r	Inverse variance weighted	0.599436222	0.05	1
	IDP dMRI ProbtrackX MD str l	Wald ratio	0.614699587	0.05	1
	IDP dMRI ProbtrackX MD str r	Wald ratio	0.734400143	0.05	1
	IDP dMRI ProbtrackX MD unc l	Wald ratio	0.734400143	0.05	1
	IDP dMRI ProbtrackX MD unc r	Inverse variance weighted	0.77310111	0.05	1
	IDP dMRI ProbtrackX L1 fmi	Wald ratio	0.722300433	0.05	1
	IDP dMRI ProbtrackX L1 ifo l	Wald ratio	0.722300433	0.05	1
	IDP dMRI ProbtrackX L1 ifo r	Wald ratio	0.722300433	0.05	1
	IDP dMRI ProbtrackX L1 ilf l	Wald ratio	0.734400143	0.05	1
	IDP dMRI ProbtrackX L1 ilf r	Wald ratio	0.722300433	0.05	1
	IDP dMRI ProbtrackX L1 slf l	Wald ratio	0.734400143	0.05	1
	IDP dMRI ProbtrackX L1 slf r	Wald ratio	0.722300433	0.05	1
	IDP dMRI ProbtrackX L1 str r	Wald ratio	0.834499821	0.05	1
	IDP dMRI ProbtrackX L1 unc l	Inverse variance weighted	0.666670968	0.05	1
	IDP dMRI ProbtrackX L1 unc r	Inverse variance weighted	0.79414945	0.05	1
	IDP dMRI ProbtrackX L2 ar l	Wald ratio	0.734400143	0.05	1
	IDP dMRI ProbtrackX L2 ar r	Wald ratio	0.734400143	0.05	1
	IDP dMRI ProbtrackX L2 atr l	Wald ratio	0.734400143	0.05	1
	IDP dMRI ProbtrackX L2 atr r	Wald ratio	0.734400143	0.05	1
	IDP dMRI ProbtrackX L2 cgc l	Wald ratio	0.722300433	0.05	1
	IDP dMRI ProbtrackX L2 cgc r	Wald ratio	0.722300433	0.05	1

	IDP dMRI ProbtrackX L2 cgh l	Wald ratio	0.615099764	0.05	1
	IDP dMRI ProbtrackX L2 fma	Wald ratio	0.562100293	0.05	1
	IDP dMRI ProbtrackX L2 fmi	Inverse variance weighted	0.611166256	0.05	1
	IDP dMRI ProbtrackX L2 ifo l	Inverse variance weighted	0.601272247	0.05	1
	IDP dMRI ProbtrackX L2 ifo r	Inverse variance weighted	0.599485924	0.05	1
	IDP dMRI ProbtrackX L2 ilf l	Inverse variance weighted	0.60239595	0.05	1
	IDP dMRI ProbtrackX L2 ilf r	Inverse variance weighted	0.604593123	0.05	1
	IDP dMRI ProbtrackX L2 ptr l	Wald ratio	0.734400143	0.05	1
	IDP dMRI ProbtrackX L2 ptr r	Wald ratio	0.733899313	0.05	1
	IDP dMRI ProbtrackX L2 slf l	Inverse variance weighted	0.608873277	0.05	1
	IDP dMRI ProbtrackX L2 slf r	Inverse variance weighted	0.59799886	0.05	1
	IDP dMRI ProbtrackX L2 str l	Wald ratio	0.614699587	0.05	1
	IDP dMRI ProbtrackX L2 str r	Wald ratio	0.614699587	0.05	1
	IDP dMRI ProbtrackX L2 unc l	Wald ratio	0.734400143	0.05	1
	IDP dMRI ProbtrackX L2 unc r	Inverse variance weighted	0.789099679	0.05	1
	IDP dMRI ProbtrackX L3 ar l	Wald ratio	0.734400143	0.05	1
	IDP dMRI ProbtrackX L3 ar r	Wald ratio	0.734400143	0.05	1
	IDP dMRI ProbtrackX L3 atr l	Wald ratio	0.734400143	0.05	1
	IDP dMRI ProbtrackX L3 atr r	Wald ratio	0.734400143	0.05	1
	IDP dMRI ProbtrackX L3 cgc l	Wald ratio	0.817299967	0.05	1
	IDP dMRI ProbtrackX L3 cgc r	Wald ratio	0.817299967	0.05	1

	IDP dMRI ProbtrackX L3 fmi	MR Egger	0.687734973	0.05	1
		Weighted median	0.640469934	0.05	1
		Inverse variance weighted	0.821784044	0.05	1
		Simple mode	0.710234673	0.05	1
		Weighted mode	0.719654456	0.05	1
	IDP dMRI ProbtrackX L3 ifo l	Inverse variance weighted	0.615121224	0.05	1
	IDP dMRI ProbtrackX L3 ifo r	Inverse variance weighted	0.603437415	0.05	1
	IDP dMRI ProbtrackX L3 ilf l	Inverse variance weighted	0.605000367	0.05	1
	IDP dMRI ProbtrackX L3 ilf r	Inverse variance weighted	0.608268234	0.05	1
	IDP dMRI ProbtrackX L3 ptr l	Wald ratio	0.734400143	0.05	1
	IDP dMRI ProbtrackX L3 ptr r	Wald ratio	0.733899313	0.05	1
	IDP dMRI ProbtrackX L3 slf l	Inverse variance weighted	0.610281296	0.05	1
	IDP dMRI ProbtrackX L3 slf r	Inverse variance weighted	0.600655227	0.05	1
	IDP dMRI ProbtrackX L3 str l	Wald ratio	0.733899313	0.05	1
	IDP dMRI ProbtrackX L3 str r	Wald ratio	0.722300433	0.05	1
	IDP dMRI ProbtrackX L3 unc l	Wald ratio	0.734400143	0.05	1
	IDP dMRI ProbtrackX L3 unc r	Inverse variance weighted	0.746766392	0.05	1
	IDP dMRI ProbtrackX ICVF ar l	Inverse variance weighted	0.937242088	0.05	1
	IDP dMRI ProbtrackX ICVF ar r	Inverse variance weighted	0.937587736	0.05	1
	IDP dMRI ProbtrackX ICVF atr l	MR Egger	0.763965016	0.05	1
		Weighted median	0.653630427	0.05	1

		Inverse variance weighted	0.810769881	0.05	1
		Simple mode	0.690189247	0.05	1
		Weighted mode	0.716049072	0.05	1
	IDP dMRI ProbtrackX ICVF atr r	MR Egger	0.770944047	0.05	1
		Weighted median	0.666208204	0.05	1
		Inverse variance weighted	0.819166383	0.05	1
		Simple mode	0.69361904	0.05	1
		Weighted mode	0.715586949	0.05	1
	IDP dMRI ProbtrackX ICVF cgc l	Wald ratio	0.722300433	0.05	1
	IDP dMRI ProbtrackX ICVF cgc r	Wald ratio	0.722300433	0.05	1
	IDP dMRI ProbtrackX ICVF cgh l	MR Egger	0.772439105	0.05	1
		Weighted median	0.665848124	0.05	1
		Inverse variance weighted	0.8141686	0.05	1
		Simple mode	0.695029466	0.05	1
		Weighted mode	0.713131898	0.05	1
	IDP dMRI ProbtrackX ICVF cgh r	Wald ratio	0.734400143	0.05	1
	IDP dMRI ProbtrackX ICVF cst l	Inverse variance weighted	0.975903569	0.05	1
	IDP dMRI ProbtrackX ICVF cst r	MR Egger	0.634859531	0.05	1
		Weighted median	0.601483508	0.05	1
		Inverse variance weighted	0.352232414	0.05	1
		Simple mode	0.886035232	0.05	1
		Weighted mode	0.721561657	0.05	1

	IDP dMRI ProbtrackX ICVF fma	MR Egger	0.912388031	0.05	1
		Weighted median	0.577326983	0.05	1
		Inverse variance weighted	0.55785004	0.05	1
		Simple mode	0.533832065	0.05	1
		Weighted mode	0.826363574	0.05	1
	IDP dMRI ProbtrackX ICVF fmi	MR Egger	0.786385244	0.05	1
		Weighted median	0.564379878	0.05	1
		Inverse variance weighted	0.657656384	0.05	1
		Simple mode	0.566156881	0.05	1
		Weighted mode	0.654486842	0.05	1
	IDP dMRI ProbtrackX ICVF ifo l	Inverse variance weighted	0.621243908	0.05	1
	IDP dMRI ProbtrackX ICVF ifo r	Inverse variance weighted	0.620627935	0.05	1
	IDP dMRI ProbtrackX ICVF ilf l	Inverse variance weighted	0.618788694	0.05	1
	IDP dMRI ProbtrackX ICVF ilf r	Inverse variance weighted	0.610642937	0.05	1
	IDP dMRI ProbtrackX ICVF mcp	Inverse variance weighted	0.464684973	0.05	1
	IDP dMRI ProbtrackX ICVF ml l	Wald ratio	0.815899756	0.05	1
	IDP dMRI ProbtrackX ICVF ml r	Wald ratio	0.734400143	0.05	1
	IDP dMRI ProbtrackX ICVF ptr l	Inverse variance weighted	0.612368201	0.05	1
	IDP dMRI ProbtrackX ICVF ptr r	MR Egger	0.810406236	0.05	1
		Weighted median	0.746094133	0.05	1
		Inverse variance weighted	0.748149337	0.05	1

		Simple mode	0.720780958	0.05	1
		Weighted mode	0.756530612	0.05	1
	IDP dMRI ProbtrackX ICVF slf l	MR Egger	0.686577653	0.05	1
		Weighted median	0.648112441	0.05	1
		Inverse variance weighted	0.7958987	0.05	1
		Simple mode	0.696081819	0.05	1
		Weighted mode	0.706442205	0.05	1
	IDP dMRI ProbtrackX ICVF slf r	MR Egger	0.668414865	0.05	1
		Weighted median	0.619430262	0.05	1
		Inverse variance weighted	0.780156628	0.05	1
		Simple mode	0.70241015	0.05	1
		Weighted mode	0.683338091	0.05	1
	IDP dMRI ProbtrackX ICVF str l	MR Egger	0.745621028	0.05	1
		Weighted median	0.650329282	0.05	1
		Inverse variance weighted	0.82489006	0.05	1
		Simple mode	0.72429908	0.05	1
		Weighted mode	0.721411204	0.05	1
	IDP dMRI ProbtrackX ICVF str r	MR Egger	0.708022003	0.05	1
		Weighted median	0.63817606	0.05	1
		Inverse variance weighted	0.810039756	0.05	1
		Simple mode	0.717695041	0.05	1
		Weighted mode	0.692789662	0.05	1

	IDP dMRI ProbtrackX ICVF unc l	Inverse variance weighted	0.939706262	0.05	1
	IDP dMRI ProbtrackX ICVF unc r	Inverse variance weighted	0.632595203	0.05	1
	IDP dMRI ProbtrackX OD ilf l	Wald ratio	0.811500437	0.05	1
	IDP dMRI ProbtrackX OD slf l	Wald ratio	0.128900143	0.05	1
	IDP dMRI ProbtrackX OD str l	Inverse variance weighted	5.21E-05	0.05	1
	IDP dMRI ProbtrackX ISOVF atr r	Wald ratio	0.734400143	0.05	1
	IDP dMRI ProbtrackX ISOVF ifo r	Inverse variance weighted	0.351671886	0.05	1
	IDP dMRI ProbtrackX ISOVF ilf l	Wald ratio	0.603699479	0.05	1
	IDP dMRI ProbtrackX ISOVF ilf r	MR Egger	0.324181637	0.05	0.003256825
		Weighted median	0.840099481	0.05	1
		Inverse variance weighted	0.624048795	0.05	1
		Simple mode	0.742777551	0.05	1
		Weighted mode	0.742804157	0.05	1
	IDP dMRI ProbtrackX ISOVF slf l	Inverse variance weighted	0.298583363	0.05	1
	IDP dMRI ProbtrackX ISOVF slf r	MR Egger	0.653668774	0.05	0.219077854
		Weighted median	0.226770595	0.05	1
		Inverse variance weighted	0.800855606	0.05	1
		Simple mode	0.348645931	0.05	1
		Weighted mode	0.328827733	0.05	1
	IDP dMRI ProbtrackX ISOVF unc l	Wald ratio	0.347999187	0.05	1
	IDP dMRI ProbtrackX ISOVF unc r	Wald ratio	0.344798974	0.05	1
	IDP T1 SIENAX brain-normalised volume	Wald ratio	0.080109617	0.05	1

Polyarteritis nodosa	IDP T1 SIENAX peripheral grey normalised volume	Wald ratio	0.782491522	0.05	1
	IDP T1 FAST ROIs R temp occ fusif cortex	Wald ratio	0.383418576	0.05	0.040221245
	IDP T1 FAST ROIs R occ pole	Inverse variance weighted	0.240924812	0.05	0.303957193
	IDP T1 FAST ROIs L thalamus	Inverse variance weighted	0.031265378	0.05	1
	IDP T1 FAST ROIs R thalamus	Wald ratio	0.054054954	0.05	1
	IDP T1 FAST ROIs L putamen	Wald ratio	0.928243565	0.05	1
	IDP T1 FAST ROIs R putamen	Wald ratio	0.928243565	0.05	1
	IDP T1 FAST ROIs L pallidum	Wald ratio	0.834039351	0.05	1
	IDP T1 FIRST left caudate volume	Wald ratio	0.981729431	0.05	1
	IDP T1 FAST ROIs L hippocampus	Inverse variance weighted	0.175153336	0.05	0.115342005
	IDP T1 FAST ROIs R hippocampus	Wald ratio	0.034314467	0.05	0.00733304
	IDP T1 FAST ROIs L ventral striatum	Wald ratio	0.928243565	0.05	1
	IDP T1 FAST ROIs R ventral striatum	Inverse variance weighted	0.668854758	0.05	1
	IDP T1 FAST ROIs L cerebellum VI	Wald ratio	0.748968331	0.05	1
	IDP T1 FAST ROIs L cerebellum crus I	MR Egger	0.186294423	0.05	1
		Weighted median	0.198849665	0.05	1
		Inverse variance weighted	0.098417642	0.05	1
		Simple mode	0.987000865	0.05	1
		Weighted mode	0.104712623	0.05	1
	IDP T1 FAST ROIs R cerebellum crus I	Inverse variance weighted	0.064763235	0.05	1
	IDP T1 FAST ROIs L cerebellum crus II	MR Egger	0.831497289	0.05	0.862813974
		Weighted median	0.532491727	0.05	0.999999944

		Inverse variance weighted	0.684180191	0.05	1
		Simple mode	0.507039068	0.05	0.920724166
		Weighted mode	0.588978809	0.05	0.999512904
	IDP T1 FAST ROIs V cerebellum crus II	Inverse variance weighted	0.055580499	0.05	0.048519889
	IDP T1 FAST ROIs R cerebellum crus II	Inverse variance weighted	0.123470375	0.05	0.507952278
	IDP T1 FIRST left putamen volume	Inverse variance weighted	0.310526647	0.05	0.225668396
	IDP T1 FAST ROIs R cerebellum VIIb	Wald ratio	0.858496724	0.05	1
	IDP T1 FAST ROIs V cerebellum VIIIa	MR Egger	0.406053806	0.05	0.002542754
		Weighted median	0.698879706	0.05	1
		Inverse variance weighted	0.909555759	0.05	1
		Simple mode	0.964242416	0.05	1
		Weighted mode	0.669060095	0.05	1
	IDP T1 FAST ROIs L cerebellum VIIIb	Wald ratio	0.830440316	0.05	1
	IDP T1 FAST ROIs V cerebellum VIIIb	Inverse variance weighted	0.486162026	0.05	1
	IDP T1 FAST ROIs R cerebellum VIIIb	Wald ratio	0.928243565	0.05	1
	IDP T1 FAST ROIs L cerebellum IX	Inverse variance weighted	0.248296725	0.05	1
	IDP T1 FIRST right putamen volume	Wald ratio	0.043185533	0.05	0.008314668
	IDP T1 FAST ROIs V cerebellum IX	Inverse variance weighted	0.952540818	0.05	1
	IDP T1 FAST ROIs R cerebellum IX	Inverse variance weighted	0.462417429	0.05	1
	IDP T1 FAST ROIs V cerebellum X	Wald ratio	0.928243565	0.05	1
	IDP T2 FLAIR BIANCA WMH volume	Inverse variance weighted	0.478505128	0.05	1

	IDP SWI T2star left caudate	MR Egger	0.350979792	0.05	1
		Weighted median	0.951118462	0.05	1
		Inverse variance weighted	0.374063548	0.05	1
		Simple mode	0.87515251	0.05	1
		Weighted mode	0.911065472	0.05	1
	IDP SWI T2star right caudate	MR Egger	0.045750188	0.05	1
		Weighted median	0.094487348	0.05	1
		Inverse variance weighted	0.214080525	0.05	1
		Simple mode	0.875533756	0.05	1
		Weighted mode	0.115104886	0.05	1
	IDP T1 FIRST left pallidum volume	Wald ratio	0.854658693	0.05	1
	IDP SWI T2star left putamen	MR Egger	0.290565503	0.05	1
		Weighted median	0.753472348	0.05	1
		Inverse variance weighted	0.494790671	0.05	1
		Simple mode	0.154277122	0.05	1
		Weighted mode	0.668499013	0.05	1
	IDP SWI T2star right putamen	MR Egger	0.273913832	0.05	1
		Weighted median	0.935599637	0.05	1
		Inverse variance weighted	0.57238583	0.05	1
		Simple mode	0.729555734	0.05	1
		Weighted mode	0.708120331	0.05	1
	IDP SWI T2star left pallidum	MR Egger	0.937965919	0.05	1

		Weighted median	0.803982964	0.05	1
		Inverse variance weighted	0.760497182	0.05	1
		Simple mode	0.918282001	0.05	1
		Weighted mode	0.732519227	0.05	1
	IDP SWI T2star right pallidum	MR Egger	0.213378255	0.05	1
		Weighted median	0.469397472	0.05	1
		Inverse variance weighted	0.938369891	0.05	1
		Simple mode	0.714361801	0.05	1
		Weighted mode	0.584035992	0.05	1
	IDP T1 FIRST right pallidum volume	Wald ratio	0.854658693	0.05	1
	IDP dMRI TBSS FA Genu of corpus callosum	Wald ratio	0.563507363	0.05	0.999999997
	IDP dMRI TBSS FA Splenium of corpus callosum	Inverse variance weighted	0.634174448	0.05	1
	IDP dMRI TBSS FA Corticospinal tract L	Wald ratio	0.701001079	0.05	1
	IDP dMRI TBSS FA Superior cerebellar peduncle R	Inverse variance weighted	0.848877267	0.05	1
	IDP dMRI TBSS FA Superior cerebellar peduncle L	Inverse variance weighted	0.895329605	0.05	1
	IDP dMRI TBSS FA Posterior limb of internal capsule R	Wald ratio	0.419099338	0.05	1
	IDP dMRI TBSS FA Retrolenticular part of internal capsule R	Inverse variance weighted	0.898676512	0.05	1
	IDP dMRI TBSS FA Anterior corona radiata R	Wald ratio	0.563507363	0.05	0.999999986
	IDP dMRI TBSS FA Anterior corona radiata L	Wald ratio	0.563507363	0.05	0.999999972
	IDP dMRI TBSS FA Posterior thalamic radiation R	Wald ratio	0.346295587	0.05	1

	IDP dMRI TBSS FA Posterior thalamic radiation L	Wald ratio	0.346295587	0.05	1
	IDP dMRI TBSS FA Sagittal stratum R	Wald ratio	0.833234568	0.05	1
	IDP dMRI TBSS FA Cingulum cingulate gyrus R	Inverse variance weighted	0.891733907	0.05	1
	IDP dMRI TBSS FA Superior longitudinal fasciculus L	Inverse variance weighted	0.770022217	0.05	1
	IDP dMRI TBSS FA Uncinate fasciculus L	Wald ratio	0.533483284	0.05	1
	IDP dMRI TBSS MD Genu of corpus callosum	Wald ratio	0.563507363	0.05	1
	IDP dMRI TBSS MD Body of corpus callosum	Wald ratio	0.789600158	0.05	1
	IDP dMRI TBSS MD Splenium of corpus callosum	Inverse variance weighted	0.889120548	0.05	1
	IDP dMRI TBSS MD Corticospinal tract R	Wald ratio	0.913882661	0.05	1
	IDP dMRI TBSS MD Superior cerebellar peduncle R	Wald ratio	0.964525257	0.05	1
	IDP dMRI TBSS MD Superior cerebellar peduncle L	Wald ratio	0.672963922	0.05	0.999999996
	IDP dMRI TBSS MD Anterior limb of internal capsule L	Wald ratio	0.007509507	0.05	1
	IDP dMRI TBSS MD Retrolenticular part of internal capsule R	Wald ratio	0.346295587	0.05	0.998202555
	IDP T1 FIRST left caudate volume plus IDP T1 FIRST right caudate volume	Wald ratio	0.154262905	0.05	1
	IDP T1 FIRST left putamen volume plus IDP T1 FIRST right putamen volume	Inverse variance weighted	0.313742375	0.05	0.999866487
	IDP T1 FIRST left pallidum volume plus IDP T1 FIRST right pallidum volume	Wald ratio	0.854658693	0.05	1
	IDP SWI T2star left thalamus plus IDP SWI T2star right thalamus	MR Egger	0.355800837	0.05	1
		Weighted median	0.664275523	0.05	1
		Inverse variance weighted	0.973203445	0.05	1
		Simple mode	0.754010888	0.05	1

		Weighted mode	0.589438887	0.05	1
	IDP dMRI TBSS MD Retrolenticular part of internal capsule L	Wald ratio	0.346295587	0.05	0.943094974
	IDP SWI T2star left caudate plus IDP SWI T2star right caudate	MR Egger	0.028201026	0.05	1
		Weighted median	0.555822595	0.05	1
		Inverse variance weighted	0.153003528	0.05	1
		Simple mode	0.844087221	0.05	1
		Weighted mode	0.806462787	0.05	1
	IDP SWI T2star left putamen plus IDP SWI T2star right putamen	MR Egger	0.146959988	0.05	1
		Weighted median	0.820603056	0.05	1
		Inverse variance weighted	0.420634969	0.05	1
		Simple mode	0.982994659	0.05	1
		Weighted mode	0.901043928	0.05	1
	IDP SWI T2star left pallidum plus IDP SWI T2star right pallidum	MR Egger	0.801760728	0.05	1
		Weighted median	0.472653655	0.05	1
		Inverse variance weighted	0.757536774	0.05	1
		Simple mode	0.262733127	0.05	1
		Weighted mode	0.319668587	0.05	1
	volume Left-Lateral-Ventricle	MR Egger	0.748938615	0.05	0.010563378
		Weighted median	0.565357576	0.05	0.999999997
		Inverse variance weighted	0.545363007	0.05	1
		Simple mode	0.528312882	0.05	0.993088698
		Weighted mode	0.512279959	0.05	0.998665581

	volume Left-Cerebellum-White-Matter	Inverse variance weighted	0.318269309	0.05	1
	volume Left-Cerebellum-Cortex	Inverse variance weighted	0.261182302	0.05	1
	IDP dMRI TBSS MD Anterior corona radiata R	Inverse variance weighted	0.921146347	0.05	1
	volume Left-Putamen	Inverse variance weighted	0.156130561	0.05	0.301192198
	volume 4th-Ventricle	Inverse variance weighted	0.266068532	0.05	0.998255458
	volume Brain-Stem	Wald ratio	0.750686726	0.05	1
	volume Left-Hippocampus	Inverse variance weighted	0.317025637	0.05	0.233500662
	volume CSF	MR Egger	0.476877553	0.05	0.00254261
		Weighted median	0.476819975	0.05	0.999887113
		Inverse variance weighted	0.998145697	0.05	1
		Simple mode	0.576820631	0.05	0.998736809
		Weighted mode	0.583487304	0.05	0.998736809
	IDP dMRI TBSS MD Anterior corona radiata L	Inverse variance weighted	0.956586853	0.05	1
	volume Left-Accumbens-area	Wald ratio	0.928243565	0.05	1
	volume Right-Lateral-Ventricle	Inverse variance weighted	0.796644243	0.05	1
	volume Right-Inf-Lat-Vent	Wald ratio	0.258296054	0.05	0.064570944
	volume Right-Cerebellum-White-Matter	Wald ratio	0.64335818	0.05	1
	volume Right-Cerebellum-Cortex	MR Egger	0.435641261	0.05	1
		Weighted median	0.012518628	0.05	1
		Inverse variance weighted	0.002870233	0.05	1
		Simple mode	0.165695505	0.05	1

		Weighted mode	0.137495206	0.05	1
	IDP dMRI TBSS MD Superior corona radiata R	Inverse variance weighted	0.921850255	0.05	1
	volume Right-Putamen	Wald ratio	0.043185533	0.05	0.014424504
	volume Right-Hippocampus	Wald ratio	0.034314467	0.05	0.004987238
	volume Right-Amygdala	Wald ratio	0.233241813	0.05	0.02256683
	volume Right-VentralDC	Wald ratio	0.539568993	0.05	1
	volume CC Posterior	Wald ratio	0.980992636	0.05	1
	IDP dMRI TBSS MD Superior corona radiata L	Inverse variance weighted	0.925629026	0.05	1
	IDP dMRI TBSS MD Posterior corona radiata R	Wald ratio	0.346295587	0.05	0.795024652
	volume BrainSegVol-to-eTIV	Wald ratio	0.926380066	0.05	1
	IDP dMRI TBSS MD Posterior corona radiata L	Wald ratio	0.346295587	0.05	0.867983897
	volume rhSurfaceHoles	Wald ratio	0.770502115	0.05	1
	DKTatlas lh cuneus area	Wald ratio	0.061563466	0.05	1
	IDP dMRI TBSS MD Posterior thalamic radiation R	Wald ratio	0.346295587	0.05	0.980038838
	DKTatlas lh lateraloccipital area	Wald ratio	0.061563466	0.05	1
	DKTatlas lh lingual area	Wald ratio	0.058114516	0.05	1
	IDP dMRI TBSS MD Posterior thalamic radiation L	Wald ratio	0.346295587	0.05	0.947924286
	DKTatlas lh parstriangularis area	Wald ratio	0.118495192	0.05	0.054109204
	DKTatlas lh pericalcarine area	Inverse variance weighted	0.010688503	0.05	1
	DKTatlas lh postcentral area	Inverse variance weighted	0.225148462	0.05	0.676467995
	DKTatlas lh posteriorcingulate area	Wald ratio	0.470667731	0.05	0.441856486

	DKTatlas lh precentral area	Wald ratio	0.126675204	0.05	0.93486071
	DKTatlas lh precuneus area	Wald ratio	0.081367366	0.05	0.005752614
	IDP dMRI TBSS MD Sagittal stratum R	Wald ratio	0.346295587	0.05	0.993647924
	DKTatlas lh superiorparietal area	Wald ratio	0.129872024	0.05	1
	DKTatlas lh superiortemporal area	Wald ratio	0.199368175	0.05	1
	DKTatlas lh supramarginal area	Wald ratio	0.801065409	0.05	1
	DKTatlas lh WhiteSurfArea area	Wald ratio	0.630663019	0.05	0.959758692
	IDP dMRI TBSS MD Sagittal stratum L	Wald ratio	0.346295587	0.05	0.975572187
	a2009s lh G&S subcentral area	Wald ratio	0.126675204	0.05	0.102639665
	a2009s lh G cuneus area	Wald ratio	0.067611677	0.05	1
	a2009s lh G front inf-Opercular area	Wald ratio	0.389645551	0.05	1
	IDP dMRI TBSS MD External capsule R	Wald ratio	0.563507363	0.05	1
	IDP dMRI TBSS MD External capsule L	Inverse variance weighted	0.233674098	0.05	1
	a2009s lh G pariet inf-Supramar area	Wald ratio	0.801065409	0.05	1
	a2009s lh G parietal sup area	Wald ratio	0.129872024	0.05	1
	a2009s lh G postcentral area	Wald ratio	0.129872024	0.05	0.037715257
	a2009s lh G precentral area	Wald ratio	0.126675204	0.05	0.915231442
	a2009s lh G precuneus area	Wald ratio	0.067611677	0.05	0.056899586
	IDP dMRI TBSS MD Cingulum cingulate gyrus R	MR Egger	0.830363106	0.05	1
		Weighted median	0.684987128	0.05	1
		Inverse variance weighted	0.546868457	0.05	1
		Simple mode	0.94802958	0.05	1
		Weighted mode	0.871532004	0.05	1

	IDP dMRI TBSS MD Cingulum cingulate gyrus L	MR Egger	0.808893741	0.05	1
		Weighted median	0.664066708	0.05	1
		Inverse variance weighted	0.539581601	0.05	1
		Simple mode	0.948024888	0.05	1
		Weighted mode	0.837395877	0.05	1
	a2009s lh S calcarine area	Wald ratio	0.058114516	0.05	1
	a2009s lh S central area	Wald ratio	0.127987672	0.05	0.999387316
	a2009s lh S collat transv ant area	Wald ratio	0.015125385	0.05	1
	a2009s lh S front middle area	Wald ratio	0.62478339	0.05	1
	IDP dMRI TBSS MD Cingulum hippocampus R	Wald ratio	0.346295587	0.05	0.974867354
	a2009s lh S intrapariet&P trans area	Wald ratio	0.129872024	0.05	1
	IDP dMRI TBSS MD Cingulum hippocampus L	Wald ratio	0.346295587	0.05	0.974144812
	a2009s lh S subparietal area	Wald ratio	0.058114516	0.05	0.039974238
	DKTatlas rh cuneus area	Wald ratio	0.058114516	0.05	1
	DKTatlas rh lingual area	Wald ratio	0.058662015	0.05	1
	DKTatlas rh parstriangularis area	Wald ratio	0.342082746	0.05	0.891970079
	DKTatlas rh pericalcarine area	Inverse variance weighted	0.232863626	0.05	1
	IDP dMRI TBSS MD Superior longitudinal fasciculus R	Wald ratio	0.346295587	0.05	0.986660034
	DKTatlas rh postcentral area	Wald ratio	0.127987672	0.05	0.014115972
	DKTatlas rh precentral area	Wald ratio	0.126675204	0.05	0.886139151
	IDP dMRI TBSS MD Superior longitudinal fasciculus L	Wald ratio	0.346295587	0.05	0.993543559
	a2009s rh G&S subcentral area	Wald ratio	0.127987672	0.05	0.19444205

	a2009s rh G&S cingul-Mid-Post area	Wald ratio	0.284977688	0.05	0.340588137
	a2009s rh G cuneus area	Wald ratio	0.058114516	0.05	1
	IDP dMRI TBSS MD Superior fronto-occipital fasciculus L	Wald ratio	0.548761609	0.05	0.99956872
	a2009s rh G oc-temp med-Lingual area	Wald ratio	0.058662015	0.05	1
	a2009s rh G parietal sup area	Wald ratio	0.234790602	0.05	0.12930377
	IDP dMRI TBSS MD Uncinate fasciculus R	Wald ratio	0.007519289	0.05	1
	a2009s rh G precentral area	Wald ratio	0.126675204	0.05	0.656915707
	a2009s rh G precuneus area	Wald ratio	0.314869457	0.05	0.382583169
	IDP dMRI TBSS MD Uncinate fasciculus L	Inverse variance weighted	0.337333853	0.05	1
	a2009s rh Pole occipital area	Wald ratio	0.058114516	0.05	1
	a2009s rh S calcarine area	Wald ratio	0.058662015	0.05	1
	a2009s rh S central area	Wald ratio	0.126675204	0.05	0.998713539
	a2009s rh S collat transv ant area	Wald ratio	0.015125385	0.05	1
	a2009s rh S orbital med-olfact area	Wald ratio	0.043086755	0.05	0.006241048
	IDP dMRI TBSS MO Pontine crossing tract	MR Egger	0.238856533	0.05	0.044636897
		Weighted median	0.133375624	0.05	0.999866483
		Inverse variance weighted	0.098423157	0.05	0.9999998
		Simple mode	0.29847673	0.05	0.970258938
		Weighted mode	0.197658327	0.05	0.842800469
	DKTatlas lh postcentral thickness	Wald ratio	0.127987672	0.05	0.443370063
	a2009s lh G insular short thickness	Wald ratio	0.188093066	0.05	1
	a2009s lh G postcentral thickness	Wald ratio	0.127987672	0.05	0.994020152

	IDP T1 SIENAX CSF normalised volume	Inverse variance weighted	0.439883896	0.05	0.998987732
	IDP dMRI TBSS MO Medial lemniscus R	Wald ratio	0.134849096	0.05	0.063288831
	a2009s lh S postcentral thickness	Wald ratio	0.615674365	0.05	1
	DKTatlas rh lateraloccipital thickness	Wald ratio	0.928243565	0.05	1
	DKTatlas rh parstriangularis thickness	Wald ratio	0.367340047	0.05	1
	DKTatlas rh postcentral thickness	Wald ratio	0.127987672	0.05	0.348026216
	DKTatlas rh posteriorcingulate thickness	Inverse variance weighted	0.536519684	0.05	1
	a2009s rh G&S cingul-Mid-Ant thickness	Wald ratio	0.888525751	0.05	1
	a2009s rh G cuneus thickness	Wald ratio	0.928243565	0.05	1
	a2009s rh G postcentral thickness	Wald ratio	0.127987672	0.05	0.973026303
	a2009s rh Pole occipital thickness	Wald ratio	0.928243565	0.05	1
	a2009s rh S oc sup&transversal thickness	Wald ratio	0.449415796	0.05	1
	a2009s rh S parieto occipital thickness	Wald ratio	0.232796144	0.05	1
	IDP dMRI TBSS MO Anterior corona radiata R	Wald ratio	0.439275205	0.05	1
	IDP dMRI TBSS MO Superior corona radiata R	Wald ratio	0.28179285	0.05	0.600540159
	IDP dMRI TBSS MO Cingulum cingulate gyrus L	Inverse variance weighted	0.50686136	0.05	1
	IDP dMRI TBSS L1 Anterior limb of internal capsule L	Wald ratio	0.007509507	0.05	1
	IDP dMRI TBSS L1 Posterior limb of internal capsule R	Wald ratio	0.182422439	0.05	0.181460204
	IDP dMRI TBSS L1 Posterior limb of internal capsule L	Wald ratio	0.293151408	0.05	0.803456336
	IDP dMRI TBSS L1 Anterior corona radiata R	Wald ratio	0.563507363	0.05	1
	IDP dMRI TBSS L1 Anterior corona radiata L	Wald ratio	0.563507363	0.05	1

	IDP dMRI TBSS L1 Posterior corona radiata R	Wald ratio	0.874599795	0.05	1
	IDP dMRI TBSS L1 Posterior corona radiata L	Wald ratio	0.563507363	0.05	1
	IDP dMRI TBSS L1 Sagittal stratum L	Wald ratio	0.563507363	0.05	1
	IDP dMRI TBSS L1 External capsule R	Wald ratio	0.563507363	0.05	1
	IDP dMRI TBSS L1 External capsule L	Inverse variance weighted	0.27660992	0.05	1
	IDP dMRI TBSS L1 Fornix cres+Stria terminalis L	Wald ratio	0.254446293	0.05	0.362996622
	IDP T1 FAST ROIs L precentral gyrus	Wald ratio	0.127987672	0.05	0.574466293
	IDP dMRI TBSS L1 Superior longitudinal fasciculus R	Inverse variance weighted	0.73523397	0.05	1
	IDP dMRI TBSS L1 Uncinate fasciculus L	Wald ratio	0.511452675	0.05	1
	IDP dMRI TBSS L2 Pontine crossing tract	Wald ratio	0.134849096	0.05	1
	IDP dMRI TBSS L2 Genu of corpus callosum	MR Egger	0.880886169	0.05	0.999996148
		Weighted median	0.323414727	0.05	1
		Inverse variance weighted	0.207082552	0.05	1
		Simple mode	0.517801742	0.05	1
		Weighted mode	0.554626349	0.05	1
	IDP dMRI TBSS L2 Body of corpus callosum	Wald ratio	0.563507363	0.05	1
	IDP dMRI TBSS L2 Splenium of corpus callosum	MR Egger	0.828618978	0.05	1
		Weighted median	0.950545462	0.05	1
		Inverse variance weighted	0.873868767	0.05	1
		Simple mode	0.885847619	0.05	1
		Weighted mode	0.813904156	0.05	1

	IDP dMRI TBSS L2 Corticospinal tract R	Wald ratio	0.913882661	0.05	1
	IDP T1 SIENAX CSF unnormalised volume	MR Egger	0.714963583	0.05	0.005759179
		Weighted median	0.528546333	0.05	0.999999793
		Inverse variance weighted	0.535466832	0.05	0.999999994
		Simple mode	0.52683357	0.05	0.976429894
		Weighted mode	0.539164314	0.05	0.996363463
	IDP T1 FAST ROIs L temporal pole	Wald ratio	0.785491655	0.05	1
	IDP dMRI TBSS L2 Superior cerebellar peduncle R	Inverse variance weighted	0.865237447	0.05	1
	IDP dMRI TBSS L2 Superior cerebellar peduncle L	Wald ratio	0.672963922	0.05	1
	IDP dMRI TBSS L2 Posterior limb of internal capsule R	Inverse variance weighted	0.894185276	0.05	1
	IDP dMRI TBSS L2 Posterior limb of internal capsule L	Wald ratio	0.545475628	0.05	1
	IDP dMRI TBSS L2 Retrolenticular part of internal capsule R	Inverse variance weighted	0.835134269	0.05	1
	IDP dMRI TBSS L2 Anterior corona radiata R	Inverse variance weighted	0.926682292	0.05	1
	IDP dMRI TBSS L2 Anterior corona radiata L	Wald ratio	0.563507363	0.05	1
	IDP dMRI TBSS L2 Superior corona radiata R	Inverse variance weighted	0.177451682	0.05	0.959476526
	IDP dMRI TBSS L2 Superior corona radiata L	Wald ratio	0.346295587	0.05	0.933961483
	IDP dMRI TBSS L2 Posterior corona radiata R	Wald ratio	0.346295587	0.05	0.988560243
	IDP dMRI TBSS L2 Posterior corona radiata L	Wald ratio	0.346295587	0.05	0.986660034
	IDP dMRI TBSS L2 Posterior thalamic radiation R	Wald ratio	0.346295587	0.05	0.960934308
	IDP dMRI TBSS L2 Posterior thalamic radiation L	Wald ratio	0.346295587	0.05	0.958798163

	IDP dMRI TBSS L2 Sagittal stratum R	Wald ratio	0.346295587	0.05	0.959341273
	IDP dMRI TBSS L2 Cingulum cingulate gyrus R	Wald ratio	0.563507363	0.05	1
	IDP dMRI TBSS L2 Cingulum hippocampus R	Wald ratio	0.346295587	0.05	0.940173434
	IDP dMRI TBSS L2 Cingulum hippocampus L	Wald ratio	0.563507363	0.05	1
	IDP dMRI TBSS L2 Uncinate fasciculus L	Wald ratio	0.563507363	0.05	1
	IDP dMRI TBSS L3 Genu of corpus callosum	Wald ratio	0.563507363	0.05	1
	IDP dMRI TBSS L3 Body of corpus callosum	Wald ratio	0.563507363	0.05	1
	IDP dMRI TBSS L3 Splenium of corpus callosum	Wald ratio	0.537145019	0.05	1
	IDP dMRI TBSS L3 Inferior cerebellar peduncle R	Wald ratio	0.563507363	0.05	1
	IDP dMRI TBSS L3 Inferior cerebellar peduncle L	Wald ratio	0.563507363	0.05	1
	IDP dMRI TBSS L3 Superior cerebellar peduncle R	Inverse variance weighted	0.885277088	0.05	1
	IDP dMRI TBSS L3 Superior cerebellar peduncle L	Wald ratio	0.672963922	0.05	1
	IDP dMRI TBSS L3 Cerebral peduncle R	Wald ratio	0.783774311	0.05	1
	IDP dMRI TBSS L3 Anterior limb of internal capsule R	Wald ratio	0.600763168	0.05	0.995995597
	IDP dMRI TBSS L3 Anterior limb of internal capsule L	Wald ratio	0.681039473	0.05	0.999627091
	IDP dMRI TBSS L3 Retrolenticular part of internal capsule R	Wald ratio	0.346295587	0.05	0.994784872
	IDP dMRI TBSS L3 Anterior corona radiata R	Inverse variance weighted	0.924946986	0.05	1
	IDP dMRI TBSS L3 Anterior corona radiata L	Inverse variance weighted	0.964791111	0.05	1
	IDP dMRI TBSS L3 Posterior corona radiata R	Inverse variance weighted	0.426955341	0.05	1

	IDP dMRI TBSS L3 Posterior thalamic radiation R	Wald ratio	0.346295587	0.05	0.967237592
	IDP dMRI TBSS L3 Posterior thalamic radiation L	Wald ratio	0.346295587	0.05	0.947255952
	IDP dMRI TBSS L3 Sagittal stratum R	Wald ratio	0.346295587	0.05	0.985607327
	IDP dMRI TBSS L3 Sagittal stratum L	Wald ratio	0.346295587	0.05	0.949887183
	IDP dMRI TBSS L3 External capsule R	Wald ratio	0.563507363	0.05	1
	IDP dMRI TBSS L3 External capsule L	Wald ratio	0.563507363	0.05	1
	IDP dMRI TBSS L3 Cingulum cingulate gyrus R	Inverse variance weighted	0.952123464	0.05	1
	IDP dMRI TBSS L3 Cingulum cingulate gyrus L	Wald ratio	0.563507363	0.05	1
	IDP dMRI TBSS L3 Cingulum hippocampus R	Wald ratio	0.563507363	0.05	1
	IDP dMRI TBSS L3 Cingulum hippocampus L	Wald ratio	0.563507363	0.05	1
	IDP dMRI TBSS L3 Fornix cres+Stria terminalis R	Wald ratio	0.973142198	0.05	1
	IDP dMRI TBSS L3 Superior longitudinal fasciculus R	Wald ratio	0.346295587	0.05	0.988381875
	IDP dMRI TBSS L3 Superior longitudinal fasciculus L	Wald ratio	0.346295587	0.05	0.996206925
	IDP dMRI TBSS L3 Uncinate fasciculus R	Wald ratio	0.563507363	0.05	1
	IDP dMRI TBSS L3 Uncinate fasciculus L	Inverse variance weighted	0.956528809	0.05	1
	IDP dMRI TBSS ICVF Middle cerebellar peduncle	Wald ratio	0.346295587	0.05	1
	IDP dMRI TBSS ICVF Genu of corpus callosum	MR Egger	0.958553212	0.05	1
		Weighted median	0.356966759	0.05	1
		Inverse variance weighted	0.23811582	0.05	1
		Simple mode	0.374233889	0.05	0.999999667

		Weighted mode	0.426322533	0.05	1
	IDP dMRI TBSS ICVF Body of corpus callosum	MR Egger	0.744989039	0.05	0.999803554
		Weighted median	0.357951096	0.05	1
		Inverse variance weighted	0.49157835	0.05	1
		Simple mode	0.501417901	0.05	0.999999999
		Weighted mode	0.513585701	0.05	1
	IDP dMRI TBSS ICVF Splenium of corpus callosum	MR Egger	0.759097101	0.05	0.999887363
		Weighted median	0.335075976	0.05	1
		Inverse variance weighted	0.236649843	0.05	0.999999993
		Simple mode	0.528615609	0.05	1
		Weighted mode	0.520263061	0.05	1
	IDP dMRI TBSS ICVF Fornix	Wald ratio	0.548761609	0.05	1
	IDP dMRI TBSS ICVF Medial lemniscus R	Inverse variance weighted	0.88755298	0.05	1
	IDP dMRI TBSS ICVF Medial lemniscus L	Wald ratio	0.718186703	0.05	1
	IDP dMRI TBSS ICVF Inferior cerebellar peduncle R	Inverse variance weighted	0.857115478	0.05	1
	IDP dMRI TBSS ICVF Inferior cerebellar peduncle L	Inverse variance weighted	0.847416996	0.05	1
	IDP dMRI TBSS ICVF Superior cerebellar peduncle R	MR Egger	0.612894058	0.05	1
		Weighted median	0.616472718	0.05	1
		Inverse variance weighted	0.643018822	0.05	1
		Simple mode	0.504728291	0.05	0.99999834
		Weighted mode	0.580155848	0.05	1
	IDP dMRI TBSS ICVF Superior cerebellar peduncle L	Inverse variance weighted	0.973770218	0.05	1

	IDP dMRI TBSS ICVF Cerebral peduncle R	MR Egger	0.446275886	0.05	0.005567644
		Weighted median	0.838295758	0.05	1
		Inverse variance weighted	0.81552506	0.05	1
		Simple mode	0.866351223	0.05	1
		Weighted mode	0.725398043	0.05	1
	IDP dMRI TBSS ICVF Cerebral peduncle L	Wald ratio	0.830440316	0.05	1
	IDP dMRI TBSS ICVF Anterior limb of internal capsule R	Wald ratio	0.563507363	0.05	1
	IDP dMRI TBSS ICVF Anterior limb of internal capsule L	MR Egger	0.838131589	0.05	1
		Weighted median	0.57247531	0.05	1
		Inverse variance weighted	0.426870522	0.05	0.999999998
		Simple mode	0.832073613	0.05	1
		Weighted mode	0.598271583	0.05	1
	IDP dMRI TBSS ICVF Posterior limb of internal capsule R	MR Egger	0.374981379	0.05	1
		Weighted median	0.43653916	0.05	0.999997994
		Inverse variance weighted	0.681568586	0.05	1
		Simple mode	0.436588042	0.05	0.97978925
		Weighted mode	0.454256042	0.05	0.98339894
	IDP dMRI TBSS ICVF Posterior limb of internal capsule L	MR Egger	0.861394436	0.05	0.999999985
		Weighted median	0.565940932	0.05	1
		Inverse variance weighted	0.647882662	0.05	1
		Simple mode	0.481235631	0.05	0.999999619
		Weighted mode	0.595317394	0.05	1

	IDP dMRI TBSS ICVF Retrolenticular part of internal capsule R	MR Egger	0.705529263	0.05	1
		Weighted median	0.704212381	0.05	1
		Inverse variance weighted	0.944732948	0.05	1
		Simple mode	0.98767445	0.05	1
		Weighted mode	0.749691661	0.05	1
	IDP dMRI TBSS ICVF Retrolenticular part of internal capsule L	MR Egger	0.938285227	0.05	1
		Weighted median	0.67462848	0.05	1
		Inverse variance weighted	0.685073566	0.05	1
		Simple mode	0.548110097	0.05	1
		Weighted mode	0.664646323	0.05	1
	IDP dMRI TBSS ICVF Anterior corona radiata R	MR Egger	0.954844554	0.05	1
		Weighted median	0.677672346	0.05	1
		Inverse variance weighted	0.694088223	0.05	1
		Simple mode	0.532257659	0.05	0.999999998
		Weighted mode	0.651080532	0.05	1
	IDP dMRI TBSS ICVF Anterior corona radiata L	MR Egger	0.908859909	0.05	1
		Weighted median	0.637059793	0.05	1
		Inverse variance weighted	0.668084097	0.05	1
		Simple mode	0.505726797	0.05	0.999999987
		Weighted mode	0.645140508	0.05	1
	IDP dMRI TBSS ICVF Superior corona radiata R	MR Egger	0.991446344	0.05	1
		Weighted median	0.719929375	0.05	1

		Inverse variance weighted	0.720916689	0.05	1
		Simple mode	0.545871931	0.05	0.999999997
		Weighted mode	0.667813276	0.05	1
	IDP dMRI TBSS ICVF Superior corona radiata L	MR Egger	0.974380262	0.05	1
		Weighted median	0.690856448	0.05	1
		Inverse variance weighted	0.704242072	0.05	1
		Simple mode	0.514029793	0.05	0.999999981
		Weighted mode	0.658973301	0.05	1
	IDP dMRI TBSS ICVF Posterior corona radiata R	MR Egger	0.445241013	0.05	0.015574301
		Weighted median	0.475945928	0.05	1
		Inverse variance weighted	0.657045965	0.05	1
		Simple mode	0.384753414	0.05	1
		Weighted mode	0.608490377	0.05	1
	IDP dMRI TBSS ICVF Posterior corona radiata L	MR Egger	0.450335624	0.05	0.041348718
		Weighted median	0.553542006	0.05	1
		Inverse variance weighted	0.684655044	0.05	1
		Simple mode	0.402108305	0.05	1
		Weighted mode	0.615113336	0.05	1
	IDP dMRI TBSS ICVF Posterior thalamic radiation R	Wald ratio	0.346295587	0.05	1
	IDP dMRI TBSS ICVF Posterior thalamic radiation L	Inverse variance weighted	0.483397461	0.05	1
	IDP dMRI TBSS ICVF Sagittal stratum R	MR Egger	0.764902906	0.05	1
		Weighted median	0.558033428	0.05	1

		Inverse variance weighted	0.493646288	0.05	1
		Simple mode	0.881926575	0.05	1
		Weighted mode	0.581702696	0.05	1
	IDP dMRI TBSS ICVF Sagittal stratum L	Inverse variance weighted	0.376070646	0.05	1
	IDP dMRI TBSS ICVF External capsule R	MR Egger	0.853339567	0.05	0.99998415
		Weighted median	0.555371413	0.05	1
		Inverse variance weighted	0.490675148	0.05	0.999999997
		Simple mode	0.797099871	0.05	1
		Weighted mode	0.734411431	0.05	1
	IDP dMRI TBSS ICVF External capsule L	Inverse variance weighted	0.423373962	0.05	0.998073111
	IDP dMRI TBSS ICVF Cingulum cingulate gyrus R	Inverse variance weighted	0.946265819	0.05	1
	IDP dMRI TBSS ICVF Cingulum cingulate gyrus L	Wald ratio	0.449037427	0.05	1
	IDP dMRI TBSS ICVF Cingulum hippocampus R	MR Egger	0.580742577	0.05	0.999510092
		Weighted median	0.700114301	0.05	1
		Inverse variance weighted	0.924899606	0.05	1
		Simple mode	0.881903511	0.05	1
		Weighted mode	0.67263132	0.05	1
	IDP dMRI TBSS ICVF Cingulum hippocampus L	MR Egger	0.907121892	0.05	1
		Weighted median	0.65656319	0.05	1
		Inverse variance weighted	0.664080054	0.05	1
		Simple mode	0.553260631	0.05	1

		Weighted mode	0.661965849	0.05	1
	IDP dMRI TBSS ICVF Fornix cres+Stria terminalis R	Wald ratio	0.271744675	0.05	1
	IDP dMRI TBSS ICVF Superior longitudinal fasciculus R	Inverse variance weighted	0.920596092	0.05	1
	IDP dMRI TBSS ICVF Superior longitudinal fasciculus L	MR Egger	0.930631206	0.05	1
		Weighted median	0.77104936	0.05	1
		Inverse variance weighted	0.754605726	0.05	1
		Simple mode	0.568494825	0.05	1
		Weighted mode	0.740360317	0.05	1
	IDP dMRI TBSS ICVF Superior fronto-occipital fasciculus R	Inverse variance weighted	0.519895904	0.05	1
	IDP dMRI TBSS ICVF Uncinate fasciculus R	Wald ratio	0.563507363	0.05	1
	IDP dMRI TBSS ICVF Uncinate fasciculus L	Wald ratio	0.563507363	0.05	1
	IDP dMRI TBSS OD Pontine crossing tract	MR Egger	0.676811611	0.05	1
		Weighted median	0.269673712	0.05	1
		Inverse variance weighted	0.4157479	0.05	1
		Simple mode	0.403725167	0.05	1
		Weighted mode	0.364192261	0.05	1
	IDP dMRI TBSS OD Cerebral peduncle R	Wald ratio	0.863029674	0.05	1
	IDP dMRI TBSS OD Cerebral peduncle L	Wald ratio	0.863029674	0.05	1
	IDP dMRI TBSS OD Posterior limb of internal capsule L	Wald ratio	0.293151408	0.05	1
	IDP dMRI TBSS OD External capsule R	Inverse variance weighted	0.313871103	0.05	0.994556095
	IDP dMRI TBSS OD External capsule L	Wald ratio	0.284319713	0.05	0.288361682

	IDP dMRI TBSS OD Superior longitudinal fasciculus R	Wald ratio	0.283082965	0.05	1
	IDP dMRI TBSS ISOVF Fornix	Wald ratio	0.950834082	0.05	1
	IDP dMRI TBSS ISOVF External capsule R	Wald ratio	0.616044215	0.05	0.999999967
	IDP dMRI TBSS ISOVF Cingulum cingulate gyrus R	Wald ratio	0.368798563	0.05	0.876486175
	IDP dMRI ProbtrackX FA ar l	Wald ratio	0.563507363	0.05	0.999999934
	IDP dMRI ProbtrackX FA atr l	Wald ratio	0.563507363	0.05	1
	IDP dMRI ProbtrackX FA fmi	MR Egger	0.905411625	0.05	1
		Weighted median	0.401909988	0.05	1
		Inverse variance weighted	0.495927758	0.05	1
		Simple mode	0.471974072	0.05	0.99998645
		Weighted mode	0.488496483	0.05	0.999999999
	IDP dMRI ProbtrackX FA ifo l	Wald ratio	0.346295587	0.05	1
	IDP dMRI ProbtrackX FA ifo r	Wald ratio	0.358495695	0.05	1
	IDP dMRI ProbtrackX FA ml l	Wald ratio	0.358120356	0.05	0.670747723
	IDP dMRI ProbtrackX FA ptr r	Wald ratio	0.511452675	0.05	1
	IDP dMRI ProbtrackX FA slf l	Wald ratio	0.358803672	0.05	1
	IDP dMRI ProbtrackX FA str l	Wald ratio	0.469598196	0.05	0.999906034
	IDP dMRI ProbtrackX FA unc l	Wald ratio	0.563507363	0.05	0.999999998
	IDP dMRI ProbtrackX FA unc r	Wald ratio	0.563507363	0.05	1
	IDP dMRI ProbtrackX MD ar l	Wald ratio	0.563507363	0.05	1
	IDP dMRI ProbtrackX MD ar r	Wald ratio	0.563507363	0.05	1
	IDP dMRI ProbtrackX MD atr l	Wald ratio	0.563507363	0.05	1
	IDP dMRI ProbtrackX MD atr r	Wald ratio	0.563507363	0.05	1

	IDP dMRI ProbtrackX MD cgc l	MR Egger	0.709997096	0.05	1
		Weighted median	0.580085796	0.05	1
		Inverse variance weighted	0.492140743	0.05	1
		Simple mode	0.957766626	0.05	1
		Weighted mode	0.77093819	0.05	1
	IDP dMRI ProbtrackX MD cgc r	Inverse variance weighted	0.689109728	0.05	1
	IDP dMRI ProbtrackX MD fmi	Inverse variance weighted	0.953638644	0.05	1
	IDP dMRI ProbtrackX MD ifo l	Wald ratio	0.346295587	0.05	0.995731187
	IDP dMRI ProbtrackX MD ifo r	Wald ratio	0.346295587	0.05	0.983276064
	IDP dMRI ProbtrackX MD ilf l	Wald ratio	0.346295587	0.05	0.981449648
	IDP dMRI ProbtrackX MD ilf r	Wald ratio	0.346295587	0.05	0.951788002
	IDP dMRI ProbtrackX MD ptr l	Wald ratio	0.563507363	0.05	1
	IDP dMRI ProbtrackX MD ptr r	Wald ratio	0.563507363	0.05	1
	IDP dMRI ProbtrackX MD slf l	Inverse variance weighted	0.931368141	0.05	1
	IDP dMRI ProbtrackX MD slf r	Wald ratio	0.346295587	0.05	0.901532036
	IDP dMRI ProbtrackX MD str l	Wald ratio	0.731336945	0.05	1
	IDP dMRI ProbtrackX MD str r	Wald ratio	0.563507363	0.05	1
	IDP dMRI ProbtrackX MD unc l	Wald ratio	0.563507363	0.05	1
	IDP dMRI ProbtrackX MD unc r	Inverse variance weighted	0.820470689	0.05	1
	IDP dMRI ProbtrackX MO atr l	Wald ratio	0.593852848	0.05	0.999998453
	IDP dMRI ProbtrackX MO ml r	Wald ratio	0.195107188	0.05	1
	IDP T1 FAST ROIs L intracalc cortex	Wald ratio	0.058114516	0.05	1

	IDP dMRI ProbtrackX L1 ilf l	Wald ratio	0.563507363	0.05	1
	IDP T1 FAST ROIs R intracalc cortex	Wald ratio	0.058114516	0.05	1
	IDP dMRI ProbtrackX L1 slf l	Wald ratio	0.563507363	0.05	1
	IDP dMRI ProbtrackX L1 slf r	Wald ratio	0.094683559	0.05	0.003984655
	IDP dMRI ProbtrackX L1 str r	Wald ratio	0.687085577	0.05	0.999999922
	IDP dMRI ProbtrackX L1 unc l	Inverse variance weighted	0.264728631	0.05	1
	IDP dMRI ProbtrackX L1 unc r	Wald ratio	0.121011872	0.05	0.02819068
	IDP dMRI ProbtrackX L2 ar l	Wald ratio	0.563507363	0.05	1
	IDP dMRI ProbtrackX L2 ar r	Wald ratio	0.563507363	0.05	1
	IDP dMRI ProbtrackX L2 atr l	Wald ratio	0.563507363	0.05	1
	IDP dMRI ProbtrackX L2 atr r	Wald ratio	0.563507363	0.05	1
	IDP dMRI ProbtrackX L2 cgh l	Wald ratio	0.102529119	0.05	1
	IDP dMRI ProbtrackX L2 fma	Wald ratio	0.359110009	0.05	0.596608695
	IDP dMRI ProbtrackX L2 fmi	Inverse variance weighted	0.951659145	0.05	1
	IDP dMRI ProbtrackX L2 ifo l	Wald ratio	0.346295587	0.05	0.99748896
	IDP dMRI ProbtrackX L2 ifo r	Wald ratio	0.346295587	0.05	0.992650199
	IDP dMRI ProbtrackX L2 ilf l	Wald ratio	0.346295587	0.05	0.982513877
	IDP dMRI ProbtrackX L2 ilf r	Wald ratio	0.346295587	0.05	0.985607327
	IDP dMRI ProbtrackX L2 ptr l	Wald ratio	0.563507363	0.05	1
	IDP dMRI ProbtrackX L2 ptr r	Wald ratio	0.557265867	0.05	1
	IDP dMRI ProbtrackX L2 slf l	Inverse variance weighted	0.925655129	0.05	1
	IDP dMRI ProbtrackX L2 slf r	Wald ratio	0.346295587	0.05	0.94923985
	IDP dMRI ProbtrackX L2 str l	Wald ratio	0.731336945	0.05	1

	IDP dMRI ProbtrackX L2 str r	Wald ratio	0.731336945	0.05	1
	IDP dMRI ProbtrackX L2 unc l	Wald ratio	0.563507363	0.05	1
	IDP dMRI ProbtrackX L2 unc r	Inverse variance weighted	0.837343811	0.05	1
	IDP dMRI ProbtrackX L3 ar l	Wald ratio	0.563507363	0.05	1
	IDP dMRI ProbtrackX L3 ar r	Wald ratio	0.563507363	0.05	1
	IDP dMRI ProbtrackX L3 atr l	Wald ratio	0.563507363	0.05	1
	IDP dMRI ProbtrackX L3 atr r	Wald ratio	0.563507363	0.05	1
	IDP dMRI ProbtrackX L3 fmi	MR Egger	0.976138949	0.05	1
		Weighted median	0.672100699	0.05	1
		Inverse variance weighted	0.702988696	0.05	1
		Simple mode	0.523012356	0.05	1
		Weighted mode	0.640326088	0.05	1
	IDP dMRI ProbtrackX L3 ifo l	Inverse variance weighted	0.988376916	0.05	1
	IDP dMRI ProbtrackX L3 ifo r	Wald ratio	0.346295587	0.05	0.987257935
	IDP dMRI ProbtrackX L3 ilf l	Wald ratio	0.346295587	0.05	0.984940526
	IDP dMRI ProbtrackX L3 ilf r	Wald ratio	0.346295587	0.05	0.963961539
	IDP dMRI ProbtrackX L3 ptr l	Wald ratio	0.563507363	0.05	1
	IDP dMRI ProbtrackX L3 ptr r	Wald ratio	0.557265867	0.05	1
	IDP dMRI ProbtrackX L3 slf l	Inverse variance weighted	0.942172214	0.05	1
	IDP dMRI ProbtrackX L3 slf r	Wald ratio	0.346295587	0.05	0.8970208
	IDP dMRI ProbtrackX L3 str l	Wald ratio	0.557265867	0.05	1
	IDP dMRI ProbtrackX L3 unc l	Wald ratio	0.563507363	0.05	1

	IDP dMRI ProbtrackX L3 unc r	Inverse variance weighted	0.791868131	0.05	1
	IDP dMRI ProbtrackX ICVF ar l	MR Egger	0.817790335	0.05	1
		Weighted median	0.56045798	0.05	1
		Inverse variance weighted	0.549683803	0.05	1
		Simple mode	0.547067118	0.05	1
		Weighted mode	0.611745595	0.05	1
	IDP dMRI ProbtrackX ICVF ar r	MR Egger	0.846465093	0.05	1
		Weighted median	0.590366881	0.05	1
		Inverse variance weighted	0.566954511	0.05	1
		Simple mode	0.566866977	0.05	1
		Weighted mode	0.640259788	0.05	1
	IDP dMRI ProbtrackX ICVF atr l	MR Egger	0.85340378	0.05	1
		Weighted median	0.597715924	0.05	1
		Inverse variance weighted	0.629643762	0.05	1
		Simple mode	0.48829408	0.05	0.999999985
		Weighted mode	0.599988958	0.05	1
	IDP dMRI ProbtrackX ICVF atr r	MR Egger	0.842931552	0.05	1
		Weighted median	0.576611517	0.05	1
		Inverse variance weighted	0.626143054	0.05	1
		Simple mode	0.515491201	0.05	0.999999905
		Weighted mode	0.593507069	0.05	1
	IDP dMRI ProbtrackX ICVF cgc l	Wald ratio	0.449037427	0.05	1

	IDP dMRI ProbtrackX ICVF cgh l	MR Egger	0.840700683	0.05	1
		Weighted median	0.587533634	0.05	1
		Inverse variance weighted	0.621092602	0.05	1
		Simple mode	0.509877246	0.05	1
		Weighted mode	0.613498554	0.05	1
	IDP dMRI ProbtrackX ICVF cgh r	Wald ratio	0.563507363	0.05	1
	IDP dMRI ProbtrackX ICVF cst l	MR Egger	0.841399601	0.05	1
		Weighted median	0.56213695	0.05	1
		Inverse variance weighted	0.571506845	0.05	1
		Simple mode	0.531515949	0.05	1
		Weighted mode	0.622490442	0.05	1
	IDP dMRI ProbtrackX ICVF cst r	MR Egger	0.820706375	0.05	1
		Weighted median	0.624517291	0.05	1
		Inverse variance weighted	0.66875619	0.05	1
		Simple mode	0.780380922	0.05	1
		Weighted mode	0.6359977	0.05	1
	IDP dMRI ProbtrackX ICVF fma	Inverse variance weighted	0.8680327	0.05	1
	IDP dMRI ProbtrackX ICVF fmi	MR Egger	0.934814042	0.05	1
		Weighted median	0.427912727	0.05	1
		Inverse variance weighted	0.510250329	0.05	1
		Simple mode	0.450078848	0.05	0.999999995
		Weighted mode	0.494646872	0.05	1

	IDP dMRI ProbtrackX ICVF ifo l	Inverse variance weighted	0.965835981	0.05	1
	IDP dMRI ProbtrackX ICVF ifo r	Inverse variance weighted	0.97002978	0.05	1
	IDP dMRI ProbtrackX ICVF ilf l	Inverse variance weighted	0.98303372	0.05	1
	IDP dMRI ProbtrackX ICVF ilf r	Wald ratio	0.346295587	0.05	1
	IDP dMRI ProbtrackX ICVF mcp	Wald ratio	0.841185836	0.05	1
	IDP dMRI ProbtrackX ICVF ml l	Wald ratio	0.511452675	0.05	1
	IDP dMRI ProbtrackX ICVF ml r	Wald ratio	0.563507363	0.05	1
	IDP dMRI ProbtrackX ICVF ptr l	Wald ratio	0.346295587	0.05	1
	IDP dMRI ProbtrackX ICVF ptr r	Inverse variance weighted	0.097623665	0.05	1
	IDP dMRI ProbtrackX ICVF slf l	MR Egger	0.978158169	0.05	1
		Weighted median	0.710271671	0.05	1
		Inverse variance weighted	0.707368302	0.05	1
		Simple mode	0.533610562	0.05	1
		Weighted mode	0.699249418	0.05	1
	IDP dMRI ProbtrackX ICVF slf r	Inverse variance weighted	0.932269546	0.05	1
	IDP dMRI ProbtrackX ICVF str l	MR Egger	0.881428789	0.05	1
		Weighted median	0.60538207	0.05	1
		Inverse variance weighted	0.655976299	0.05	1
		Simple mode	0.520903529	0.05	0.999999977
		Weighted mode	0.625656876	0.05	1
	IDP dMRI ProbtrackX ICVF str r	MR Egger	0.941648218	0.05	1
		Weighted median	0.664680192	0.05	1

		Inverse variance weighted	0.687111196	0.05	1
		Simple mode	0.495931013	0.05	0.999999956
		Weighted mode	0.66096627	0.05	1
	IDP dMRI ProbtrackX ICVF unc l	Inverse variance weighted	0.335465005	0.05	1
	IDP dMRI ProbtrackX ICVF unc r	Inverse variance weighted	0.898775562	0.05	1
	IDP dMRI ProbtrackX OD atr r	Wald ratio	0.203207406	0.05	0.032243865
	IDP dMRI ProbtrackX OD ilf l	Wald ratio	0.56901417	0.05	1
	IDP dMRI ProbtrackX OD slf l	Wald ratio	0.956772388	0.05	1
	IDP dMRI ProbtrackX OD str l	Inverse variance weighted	0.569824841	0.05	1
	IDP dMRI ProbtrackX ISOVF atr r	Wald ratio	0.563507363	0.05	0.999999225
	IDP dMRI ProbtrackX ISOVF cgc l	Wald ratio	0.114707896	0.05	0.072287801
	IDP dMRI ProbtrackX ISOVF ifo r	Wald ratio	0.431253861	0.05	0.981512915
	IDP dMRI ProbtrackX ISOVF ilf l	Wald ratio	0.900316464	0.05	1
	IDP dMRI ProbtrackX ISOVF ilf r	MR Egger	0.566093216	0.05	1
		Weighted median	0.355759713	0.05	1
		Inverse variance weighted	0.72065299	0.05	1
		Simple mode	0.482542234	0.05	1
		Weighted mode	0.495808318	0.05	1
	IDP dMRI ProbtrackX ISOVF slf l	Inverse variance weighted	0.090259718	0.05	0.312692237
	IDP dMRI ProbtrackX ISOVF slf r	MR Egger	0.873583145	0.05	0.00300647
		Weighted median	0.212436612	0.05	0.598092365
		Inverse variance weighted	0.391727179	0.05	0.999636954

		Simple mode	0.317473497	0.05	0.144677435
		Weighted mode	0.340410315	0.05	0.164543393
	IDP dMRI ProbtrackX ISOVF unc l	Wald ratio	0.120302969	0.05	0.216852168
	IDP dMRI ProbtrackX ISOVF unc r	Wald ratio	0.121469924	0.05	0.514651742
	IDP T1 SIENAX brain-normalised volume	Wald ratio	0.872227453	0.05	1
Polymyositis	IDP T1 SIENAX peripheral grey normalised volume	Wald ratio	0.966075743	0.05	1
	IDP T1 FAST ROIs R temp occ fusif cortex	Wald ratio	0.05083399	0.05	1
	IDP T1 FAST ROIs R occ pole	Inverse variance weighted	0.03880285	0.05	1
	IDP T1 FAST ROIs L thalamus	Inverse variance weighted	0.93893609	0.05	1
	IDP T1 FAST ROIs R thalamus	Wald ratio	0.679485534	0.05	0.999683196
	IDP T1 FAST ROIs L putamen	Wald ratio	0.321971153	0.05	0.999950849
	IDP T1 FAST ROIs R putamen	Wald ratio	0.321971153	0.05	0.999936305
	IDP T1 FAST ROIs L pallidum	Wald ratio	0.778188207	0.05	1
	IDP T1 FIRST left caudate volume	Wald ratio	0.426597865	0.05	0.893721975
	IDP T1 FAST ROIs L hippocampus	Inverse variance weighted	0.827715168	0.05	1
	IDP T1 FAST ROIs R hippocampus	Wald ratio	0.718753767	0.05	1
	IDP T1 FAST ROIs L ventral striatum	Wald ratio	0.321971153	0.05	0.999413526
	IDP T1 FAST ROIs R ventral striatum	Inverse variance weighted	0.997802735	0.05	1
	IDP T1 FAST ROIs L cerebellum VI	Wald ratio	0.02303497	0.05	0.005380772
	IDP T1 FAST ROIs L cerebellum crus I	MR Egger	0.937298608	0.05	1
		Weighted median	0.86879704	0.05	1
		Inverse variance weighted	0.825423588	0.05	1

		Simple mode	0.900883071	0.05	1
		Weighted mode	0.940303013	0.05	1
	IDP T1 FAST ROIs R cerebellum crus I	Inverse variance weighted	0.855219083	0.05	1
	IDP T1 FAST ROIs L cerebellum crus II	MR Egger	0.340826927	0.05	1
		Weighted median	0.943884975	0.05	1
		Inverse variance weighted	0.534775952	0.05	1
		Simple mode	0.939016021	0.05	1
		Weighted mode	0.934680999	0.05	1
	IDP T1 FAST ROIs V cerebellum crus II	Inverse variance weighted	0.525012024	0.05	0.905647439
	IDP T1 FAST ROIs R cerebellum crus II	Inverse variance weighted	0.662424345	0.05	1
	IDP T1 FIRST left putamen volume	Wald ratio	0.826233066	0.05	1
	IDP T1 FAST ROIs R cerebellum VIIb	Wald ratio	0.111663086	0.05	1
	IDP T1 FAST ROIs V cerebellum VIIIa	MR Egger	0.562928499	0.05	0.002749068
		Weighted median	0.157863182	0.05	0.393140347
		Inverse variance weighted	0.097451147	0.05	0.270492583
		Simple mode	0.36205895	0.05	0.289409449
		Weighted mode	0.406112251	0.05	0.637100034
	IDP T1 FAST ROIs L cerebellum VIIIb	Wald ratio	0.526912366	0.05	0.990035121
	IDP T1 FAST ROIs V cerebellum VIIIb	Inverse variance weighted	0.277905832	0.05	0.719338006
	IDP T1 FAST ROIs R cerebellum VIIIb	Wald ratio	0.321971153	0.05	0.365369167
	IDP T1 FAST ROIs L cerebellum IX	Inverse variance weighted	0.594372353	0.05	1

	IDP T1 FAST ROIs V cerebellum IX	Inverse variance weighted	0.959465845	0.05	1
	IDP T1 FAST ROIs R cerebellum IX	Inverse variance weighted	0.661230774	0.05	1
	IDP T1 FAST ROIs V cerebellum X	Wald ratio	0.321971153	0.05	0.772693154
	IDP T2 FLAIR BIANCA WMH volume	Inverse variance weighted	0.340697537	0.05	0.999924743
	IDP SWI T2star left caudate	MR Egger	0.481132799	0.05	1
		Weighted median	0.447785952	0.05	1
		Inverse variance weighted	0.916788407	0.05	1
		Simple mode	0.380494719	0.05	1
		Weighted mode	0.536687895	0.05	1
	IDP SWI T2star right caudate	MR Egger	0.609288833	0.05	1
		Weighted median	0.644580882	0.05	1
		Inverse variance weighted	0.533030661	0.05	1
		Simple mode	0.587586352	0.05	1
		Weighted mode	0.7837048	0.05	1
	IDP T1 FIRST left pallidum volume	Wald ratio	0.475240478	0.05	0.999999956
	IDP SWI T2star left putamen	MR Egger	0.332182075	0.05	0.521586815
		Weighted median	0.547418989	0.05	1
		Inverse variance weighted	0.84924992	0.05	1
		Simple mode	0.615361074	0.05	1
		Weighted mode	0.667313789	0.05	1
	IDP SWI T2star right putamen	MR Egger	0.712319718	0.05	0.998161665
		Weighted median	0.696920897	0.05	1

		Inverse variance weighted	0.778302586	0.05	1
		Simple mode	0.627664506	0.05	1
		Weighted mode	0.77607985	0.05	1
	IDP SWI T2star left pallidum	MR Egger	0.22106931	0.05	1
		Weighted median	0.582511062	0.05	1
		Inverse variance weighted	0.224801428	0.05	1
		Simple mode	0.979849157	0.05	1
		Weighted mode	0.917417645	0.05	1
	IDP SWI T2star right pallidum	MR Egger	0.949923633	0.05	1
		Weighted median	0.98734643	0.05	1
		Inverse variance weighted	0.79265332	0.05	1
		Simple mode	0.634041812	0.05	1
		Weighted mode	0.816750475	0.05	1
	IDP T1 FIRST right pallidum volume	Wald ratio	0.475240478	0.05	0.999982672
	IDP dMRI TBSS FA Genu of corpus callosum	Wald ratio	0.176683308	0.05	1
	IDP dMRI TBSS FA Splenium of corpus callosum	MR Egger	0.284734888	0.05	1
		Weighted median	0.829666715	0.05	1
		Inverse variance weighted	0.562794229	0.05	1
		Simple mode	0.919658138	0.05	1
		Weighted mode	0.942388048	0.05	1
	IDP dMRI TBSS FA Corticospinal tract L	Wald ratio	0.584196575	0.05	0.999999892
	IDP dMRI TBSS FA Superior cerebellar peduncle R	Inverse variance weighted	0.638106889	0.05	1

	IDP dMRI TBSS FA Superior cerebellar peduncle L	Inverse variance weighted	0.603265566	0.05	1
	IDP dMRI TBSS FA Posterior limb of internal capsule R	Wald ratio	0.001861255	0.05	1
	IDP dMRI TBSS FA Retrolenticular part of internal capsule R	Inverse variance weighted	0.478909091	0.05	1
	IDP dMRI TBSS FA Retrolenticular part of internal capsule L	Wald ratio	0.02411879	0.05	1
	IDP dMRI TBSS FA Anterior corona radiata R	Wald ratio	0.176683308	0.05	1
	IDP dMRI TBSS FA Anterior corona radiata L	Wald ratio	0.176683308	0.05	1
	IDP dMRI TBSS FA Posterior corona radiata L	Wald ratio	0.02411879	0.05	1
	IDP dMRI TBSS FA Posterior thalamic radiation R	Inverse variance weighted	0.597328075	0.05	1
	IDP dMRI TBSS FA Posterior thalamic radiation L	Inverse variance weighted	0.616012682	0.05	1
	IDP dMRI TBSS FA Sagittal stratum R	Inverse variance weighted	0.147670067	0.05	1
	IDP dMRI TBSS FA Sagittal stratum L	Wald ratio	0.026610718	0.05	1
	IDP dMRI TBSS FA Cingulum cingulate gyrus R	Inverse variance weighted	0.20033232	0.05	1
	IDP dMRI TBSS FA Superior longitudinal fasciculus L	Inverse variance weighted	0.140748117	0.05	1
	IDP dMRI TBSS FA Uncinate fasciculus L	Wald ratio	0.901140594	0.05	1
	IDP dMRI TBSS MD Genu of corpus callosum	Wald ratio	0.176683308	0.05	0.921850275
	IDP dMRI TBSS MD Body of corpus callosum	Wald ratio	0.33990402	0.05	0.999489804
	IDP dMRI TBSS MD Splenium of corpus callosum	Inverse variance weighted	0.722160691	0.05	1
	IDP dMRI TBSS MD Corticospinal tract R	Wald ratio	0.433821804	0.05	1
	IDP dMRI TBSS MD Inferior cerebellar peduncle R	Inverse variance weighted	0.195648465	0.05	0.072167771

	IDP dMRI TBSS MD Inferior cerebellar peduncle L	Wald ratio	0.02411879	0.05	0.004079301
	IDP dMRI TBSS MD Superior cerebellar peduncle R	Wald ratio	0.424128115	0.05	0.933435655
	IDP dMRI TBSS MD Superior cerebellar peduncle L	Wald ratio	0.384175267	0.05	0.77705274
	IDP dMRI TBSS MD Anterior limb of internal capsule L	Wald ratio	0.616852128	0.05	1
	IDP dMRI TBSS MD Retrolenticular part of internal capsule R	Inverse variance weighted	0.602195938	0.05	0.999998155
	IDP T1 FIRST left caudate volume plus IDP T1 FIRST right caudate volume	Wald ratio	0.270132002	0.05	0.9999749
	IDP T1 FIRST left putamen volume plus IDP T1 FIRST right putamen volume	Wald ratio	0.900954865	0.05	1
	IDP T1 FIRST left pallidum volume plus IDP T1 FIRST right pallidum volume	Wald ratio	0.475240478	0.05	1
	IDP SWI T2star left thalamus plus IDP SWI T2star right thalamus	MR Egger	0.483081978	0.05	1
		Weighted median	0.191848647	0.05	1
		Inverse variance weighted	0.084032498	0.05	1
		Simple mode	0.408922676	0.05	1
		Weighted mode	0.431455636	0.05	1
	IDP dMRI TBSS MD Retrolenticular part of internal capsule L	Inverse variance weighted	0.562410327	0.05	0.997969659
	IDP SWI T2star left caudate plus IDP SWI T2star right caudate	MR Egger	0.482111837	0.05	1
		Weighted median	0.704560696	0.05	1
		Inverse variance weighted	0.895776508	0.05	1
		Simple mode	0.173353141	0.05	1
		Weighted mode	0.80138834	0.05	1
		MR Egger	0.438028456	0.05	1

	IDP SWI T2star left putamen plus IDP SWI T2star right putamen	Weighted median	0.624085389	0.05	1
		Inverse variance weighted	0.953109795	0.05	1
		Simple mode	0.28739289	0.05	1
		Weighted mode	0.655044985	0.05	1
	IDP SWI T2star left pallidum plus IDP SWI T2star right pallidum	MR Egger	0.379140838	0.05	1
		Weighted median	0.865845374	0.05	1
		Inverse variance weighted	0.246810092	0.05	1
		Simple mode	0.844402053	0.05	1
		Weighted mode	0.870827317	0.05	1
	volume Left-Lateral-Ventricle	MR Egger	0.3219429	0.05	0.002542684
		Weighted median	0.061223278	0.05	1
		Inverse variance weighted	0.180062765	0.05	1
		Simple mode	0.308438619	0.05	1
		Weighted mode	0.219384377	0.05	1
	volume Left-Inf-Lat-Vent	Wald ratio	0.008259483	0.05	1
	volume Left-Cerebellum-White-Matter	Inverse variance weighted	0.550916525	0.05	1
	volume Left-Cerebellum-Cortex	Wald ratio	0.903622035	0.05	1
	IDP dMRI TBSS MD Anterior corona radiata R	Inverse variance weighted	0.410587261	0.05	1
	volume Left-Putamen	Wald ratio	0.455657788	0.05	1
	volume 3rd-Ventricle	Wald ratio	0.135890538	0.05	1
	volume 4th-Ventricle	Inverse variance weighted	0.054130802	0.05	1
	volume Brain-Stem	Inverse variance weighted	0.357205406	0.05	0.976732099

	volume Left-Hippocampus	Inverse variance weighted	0.36237182	0.05	1
	volume CSF	MR Egger	0.653362513	0.05	1
		Weighted median	0.71732545	0.05	1
		Inverse variance weighted	0.97969501	0.05	1
		Simple mode	0.742837976	0.05	1
		Weighted mode	0.738800067	0.05	1
	IDP dMRI TBSS MD Anterior corona radiata L	Inverse variance weighted	0.367222583	0.05	1
	volume Left-Accumbens-area	Wald ratio	0.321971153	0.05	0.289683444
	volume Right-Lateral-Ventricle	Inverse variance weighted	0.385779167	0.05	1
	volume Right-Inf-Lat-Vent	Wald ratio	0.567583368	0.05	1
	volume Right-Cerebellum-White-Matter	Wald ratio	0.657540004	0.05	1
	volume Right-Cerebellum-Cortex	MR Egger	0.844971836	0.05	1
		Weighted median	0.193235721	0.05	0.740726399
		Inverse variance weighted	0.192965092	0.05	0.933587138
		Simple mode	0.338243866	0.05	0.511398857
		Weighted mode	0.350023625	0.05	0.511398857
	IDP dMRI TBSS MD Superior corona radiata R	Inverse variance weighted	0.409734733	0.05	0.999999997
	volume Right-Hippocampus	Wald ratio	0.718753767	0.05	1
	volume Right-Amygdala	Wald ratio	0.287469775	0.05	1
	volume Right-VentralDC	Wald ratio	0.031719168	0.05	1
	volume CC Posterior	Wald ratio	0.285952397	0.05	1
	IDP dMRI TBSS MD Superior corona radiata L	Inverse variance weighted	0.405151591	0.05	0.999999996

	IDP dMRI TBSS MD Posterior corona radiata R	Inverse variance weighted	0.544176976	0.05	0.979624106
	volume BrainSegVol-to-eTIV	Wald ratio	0.661069652	0.05	1
	IDP dMRI TBSS MD Posterior corona radiata L	Inverse variance weighted	0.597055582	0.05	0.998405169
	volume rhSurfaceHoles	Wald ratio	0.220984071	0.05	0.110648429
	DKTatlas lh cuneus area	Wald ratio	0.97000201	0.05	1
	IDP dMRI TBSS MD Posterior thalamic radiation R	Inverse variance weighted	0.64549129	0.05	0.999996392
	DKTatlas lh lateraloccipital area	Wald ratio	0.97000201	0.05	1
	DKTatlas lh lingual area	Wald ratio	0.95910077	0.05	1
	IDP dMRI TBSS MD Posterior thalamic radiation L	Inverse variance weighted	0.568275382	0.05	0.999023655
	DKTatlas lh parstriangularis area	Wald ratio	0.515521952	0.05	1
	DKTatlas lh pericalcarine area	Inverse variance weighted	0.303210598	0.05	1
	DKTatlas lh postcentral area	Inverse variance weighted	0.583418302	0.05	1
	DKTatlas lh posteriorcingulate area	Wald ratio	0.64671298	0.05	1
	DKTatlas lh precentral area	Wald ratio	0.912837237	0.05	1
	DKTatlas lh precuneus area	Wald ratio	0.105060381	0.05	0.003701833
	IDP dMRI TBSS MD Sagittal stratum R	Inverse variance weighted	0.588065987	0.05	0.99998598
	DKTatlas lh superiorparietal area	Wald ratio	0.684941887	0.05	1
	DKTatlas lh superiortemporal area	Wald ratio	0.477839823	0.05	1
	DKTatlas lh supramarginal area	Wald ratio	0.458502212	0.05	1
	DKTatlas lh WhiteSurfArea area	Wald ratio	0.164726368	0.05	0.007816989
	IDP dMRI TBSS MD Sagittal stratum L	Inverse variance weighted	0.480113048	0.05	0.998382286

	a2009s lh G&S subcentral area	Wald ratio	0.912837237	0.05	1
	a2009s lh G cuneus area	Wald ratio	0.940035403	0.05	1
	a2009s lh G front inf-Opercular area	Wald ratio	0.438127577	0.05	1
	IDP dMRI TBSS MD External capsule R	Wald ratio	0.176683308	0.05	0.634917382
	IDP dMRI TBSS MD External capsule L	Inverse variance weighted	0.658472645	0.05	1
	a2009s lh G pariet inf-Supramar area	Wald ratio	0.458502212	0.05	1
	a2009s lh G parietal sup area	Wald ratio	0.642483374	0.05	0.999993837
	a2009s lh G postcentral area	Wald ratio	0.876912336	0.05	1
	a2009s lh G precentral area	Wald ratio	0.912837237	0.05	1
	a2009s lh G precuneus area	Wald ratio	0.940035403	0.05	1
	IDP dMRI TBSS MD Cingulum cingulate gyrus R	MR Egger	0.422344724	0.05	0.00350386
		Weighted median	0.97028555	0.05	1
		Inverse variance weighted	0.602951577	0.05	1
		Simple mode	0.693218765	0.05	1
		Weighted mode	0.713533937	0.05	1
	IDP dMRI TBSS MD Cingulum cingulate gyrus L	MR Egger	0.429072237	0.05	0.004055659
		Weighted median	0.988078412	0.05	1
		Inverse variance weighted	0.60572962	0.05	1
		Simple mode	0.710862205	0.05	1
		Weighted mode	0.722975154	0.05	1
	a2009s lh S calcarine area	Wald ratio	0.95910077	0.05	1
	a2009s lh S central area	Wald ratio	0.912380355	0.05	1
	a2009s lh S collat transv ant area	Wald ratio	0.202532946	0.05	0.587454233

	a2009s lh S front middle area	Wald ratio	0.331919237	0.05	1
	IDP dMRI TBSS MD Cingulum hippocampus R	Inverse variance weighted	0.486762713	0.05	0.998455002
	a2009s lh S intrapariet&P trans area	Wald ratio	0.642483374	0.05	0.999993393
	IDP dMRI TBSS MD Cingulum hippocampus L	Inverse variance weighted	0.468373035	0.05	0.997985659
	a2009s lh S subparietal area	Wald ratio	0.95910077	0.05	1
	DKTatlas rh cuneus area	Wald ratio	0.95910077	0.05	1
	DKTatlas rh lateraloccipital area	Inverse variance weighted	0.597025931	0.05	0.999999996
	DKTatlas rh lingual area	Wald ratio	0.972071017	0.05	1
	DKTatlas rh parstriangularis area	Wald ratio	0.743469444	0.05	1
	DKTatlas rh pericalcarine area	Inverse variance weighted	0.554156766	0.05	1
	IDP dMRI TBSS MD Superior longitudinal fasciculus R	Inverse variance weighted	0.608837328	0.05	0.999964568
	DKTatlas rh postcentral area	Wald ratio	0.912380355	0.05	1
	DKTatlas rh precentral area	Wald ratio	0.912837237	0.05	1
	IDP dMRI TBSS MD Superior longitudinal fasciculus L	Inverse variance weighted	0.606256175	0.05	0.999993566
	a2009s rh G&S subcentral area	Wald ratio	0.912380355	0.05	1
	a2009s rh G&S cingul-Mid-Post area	Wald ratio	0.593206954	0.05	1
	a2009s rh G cuneus area	Wald ratio	0.95910077	0.05	1
	IDP dMRI TBSS MD Superior fronto-occipital fasciculus L	Wald ratio	0.204490189	0.05	1
	a2009s rh G oc-temp med-Lingual area	Wald ratio	0.972071017	0.05	1
	a2009s rh G parietal sup area	Wald ratio	0.785870926	0.05	1
	IDP dMRI TBSS MD Uncinate fasciculus R	Inverse variance weighted	0.667496294	0.05	0.999999737

	a2009s rh G precentral area	Wald ratio	0.912837237	0.05	1
	a2009s rh G precuneus area	Wald ratio	0.390664378	0.05	1
	IDP dMRI TBSS MD Uncinate fasciculus L	Inverse variance weighted	0.55712504	0.05	1
	a2009s rh Pole occipital area	Wald ratio	0.95910077	0.05	1
	a2009s rh S calcarine area	Wald ratio	0.972071017	0.05	1
	a2009s rh S central area	Wald ratio	0.912837237	0.05	1
	a2009s rh S collat transv ant area	Wald ratio	0.202532946	0.05	0.64908408
	a2009s rh S orbital med-olfact area	Wald ratio	0.03238636	0.05	0.002989726
	IDP dMRI TBSS MO Pontine crossing tract	MR Egger	0.926032862	0.05	1
		Weighted median	0.737563259	0.05	1
		Inverse variance weighted	0.49616739	0.05	1
		Simple mode	0.856133997	0.05	1
		Weighted mode	0.916120093	0.05	1
	DKTatlas lh postcentral thickness	Wald ratio	0.912380355	0.05	1
	a2009s lh G insular short thickness	Wald ratio	0.855328674	0.05	1
	a2009s lh G postcentral thickness	Wald ratio	0.912380355	0.05	1
	IDP T1 SIENAX CSF normalised volume	Inverse variance weighted	0.708425919	0.05	1
	IDP dMRI TBSS MO Medial lemniscus R	Wald ratio	0.51711242	0.05	1
	a2009s lh S postcentral thickness	Wald ratio	0.714086091	0.05	1
	DKTatlas rh lateraloccipital thickness	Wald ratio	0.321971153	0.05	1
	DKTatlas rh parstriangularis thickness	Wald ratio	0.567950061	0.05	1
	DKTatlas rh postcentral thickness	Wald ratio	0.912380355	0.05	1

	DKTatlas rh posteriorcingulate thickness	Inverse variance weighted	0.404635217	0.05	1
	a2009s rh G&S cingul-Mid-Ant thickness	Wald ratio	0.124599464	0.05	0.007058475
	a2009s rh G cuneus thickness	Wald ratio	0.321971153	0.05	1
	a2009s rh G postcentral thickness	Wald ratio	0.75173172	0.05	1
	a2009s rh Pole occipital thickness	Wald ratio	0.321971153	0.05	1
	a2009s rh S circular insula ant thickness	Wald ratio	0.605042747	0.05	1
	a2009s rh S oc sup&transversal thickness	Wald ratio	0.040082482	0.05	0.006525215
	a2009s rh S parieto occipital thickness	Wald ratio	0.705052485	0.05	0.999996892
	a2009s rh S temporal transverse thickness	Wald ratio	0.107116964	0.05	1
	IDP dMRI TBSS MO Anterior corona radiata R	Wald ratio	0.05482962	0.05	0.036955285
	IDP dMRI TBSS MO Superior corona radiata R	Wald ratio	0.97809789	0.05	1
	IDP dMRI TBSS MO Cingulum cingulate gyrus L	Inverse variance weighted	0.724333356	0.05	1
	IDP dMRI TBSS L1 Genu of corpus callosum	Wald ratio	0.026610718	0.05	0.003534381
	IDP dMRI TBSS L1 Anterior limb of internal capsule L	Wald ratio	0.616852128	0.05	1
	IDP dMRI TBSS L1 Posterior limb of internal capsule R	Wald ratio	0.049637098	0.05	0.00492618
	IDP dMRI TBSS L1 Posterior limb of internal capsule L	Wald ratio	0.576055989	0.05	0.999999999
	IDP dMRI TBSS L1 Anterior corona radiata R	Wald ratio	0.176683308	0.05	0.691213185
	IDP dMRI TBSS L1 Anterior corona radiata L	Wald ratio	0.176683308	0.05	0.793814017
	IDP dMRI TBSS L1 Posterior corona radiata R	Wald ratio	0.108242281	0.05	0.031216749
	IDP dMRI TBSS L1 Posterior corona radiata L	Wald ratio	0.176683308	0.05	0.529217686

	IDP dMRI TBSS L1 Sagittal stratum L	Wald ratio	0.176683308	0.05	0.793814017
	IDP dMRI TBSS L1 External capsule R	Wald ratio	0.176683308	0.05	0.381602728
	IDP dMRI TBSS L1 External capsule L	MR Egger	0.540107081	0.05	1
		Weighted median	0.896988877	0.05	1
		Inverse variance weighted	0.495859981	0.05	0.999999993
		Simple mode	0.892852292	0.05	1
		Weighted mode	0.721811741	0.05	1
	IDP dMRI TBSS L1 Cingulum hippocampus L	Wald ratio	0.026610718	0.05	0.003811567
	IDP dMRI TBSS L1 Fornix cres+Stria terminalis L	Wald ratio	0.357177749	0.05	0.268719459
	IDP T1 FAST ROIs L precentral gyrus	Wald ratio	0.912380355	0.05	1
	IDP dMRI TBSS L1 Superior longitudinal fasciculus R	Inverse variance weighted	0.251248353	0.05	0.582048485
	IDP dMRI TBSS L1 Uncinate fasciculus R	Wald ratio	0.02411879	0.05	0.003129882
	IDP dMRI TBSS L1 Uncinate fasciculus L	Wald ratio	0.244021532	0.05	0.773828061
	IDP dMRI TBSS L2 Pontine crossing tract	Wald ratio	0.51711242	0.05	1
	IDP dMRI TBSS L2 Genu of corpus callosum	MR Egger	0.403675539	0.05	0.002613932
		Weighted median	0.386411703	0.05	0.999999949
		Inverse variance weighted	0.772298356	0.05	1
		Simple mode	0.559778246	0.05	0.999947704
		Weighted mode	0.348949089	0.05	0.995083062
	IDP dMRI TBSS L2 Body of corpus callosum	Wald ratio	0.176683308	0.05	0.67578563
	IDP dMRI TBSS L2 Splenium of corpus callosum	MR Egger	0.156395394	0.05	0.002543305
		Weighted median	0.891561796	0.05	1

		Inverse variance weighted	0.684306551	0.05	1
		Simple mode	0.765136318	0.05	1
		Weighted mode	0.772235366	0.05	1
	IDP dMRI TBSS L2 Corticospinal tract R	Wald ratio	0.433821804	0.05	1
	IDP T1 SIENAX CSF unnormalised volume	MR Egger	0.298084068	0.05	0.002542627
		Weighted median	0.075895746	0.05	1
		Inverse variance weighted	0.196876081	0.05	1
		Simple mode	0.348011063	0.05	1
		Weighted mode	0.247689546	0.05	1
	IDP T1 FAST ROIs L temporal pole	Wald ratio	0.96732982	0.05	1
	IDP dMRI TBSS L2 Superior cerebellar peduncle R	Inverse variance weighted	0.618491761	0.05	1
	IDP dMRI TBSS L2 Superior cerebellar peduncle L	Wald ratio	0.384175267	0.05	0.999879356
	IDP dMRI TBSS L2 Posterior limb of internal capsule R	Inverse variance weighted	0.293488712	0.05	0.041481788
	IDP dMRI TBSS L2 Posterior limb of internal capsule L	Wald ratio	0.722017608	0.05	1
	IDP dMRI TBSS L2 Retrolenticular part of internal capsule R	Inverse variance weighted	0.511474032	0.05	1
	IDP dMRI TBSS L2 Retrolenticular part of internal capsule L	Wald ratio	0.02411879	0.05	0.006277483
	IDP dMRI TBSS L2 Anterior corona radiata R	Inverse variance weighted	0.403872179	0.05	1
	IDP dMRI TBSS L2 Anterior corona radiata L	Wald ratio	0.176683308	0.05	0.939970697
	IDP dMRI TBSS L2 Superior corona radiata R	MR Egger	0.364193749	0.05	0.002542736
		Weighted median	0.597890323	0.05	1

		Inverse variance weighted	0.875562699	0.05	1
		Simple mode	0.578647299	0.05	1
		Weighted mode	0.581003909	0.05	1
	IDP dMRI TBSS L2 Superior corona radiata L	Inverse variance weighted	0.627226043	0.05	0.999803986
	IDP dMRI TBSS L2 Posterior corona radiata R	Inverse variance weighted	0.685717577	0.05	0.999999936
	IDP dMRI TBSS L2 Posterior corona radiata L	Inverse variance weighted	0.669177218	0.05	0.99999967
	IDP dMRI TBSS L2 Posterior thalamic radiation R	Inverse variance weighted	0.647182378	0.05	0.999986595
	IDP dMRI TBSS L2 Posterior thalamic radiation L	Inverse variance weighted	0.646756356	0.05	0.999984095
	IDP dMRI TBSS L2 Sagittal stratum R	Inverse variance weighted	0.597543947	0.05	0.999601639
	IDP dMRI TBSS L2 Sagittal stratum L	Wald ratio	0.026610718	0.05	0.007370793
	IDP dMRI TBSS L2 Cingulum cingulate gyrus R	Wald ratio	0.176683308	0.05	0.97559011
	IDP dMRI TBSS L2 Cingulum cingulate gyrus L	Wald ratio	0.026610718	0.05	0.004581653
	IDP dMRI TBSS L2 Cingulum hippocampus R	Inverse variance weighted	0.529064924	0.05	0.997007594
	IDP dMRI TBSS L2 Cingulum hippocampus L	Wald ratio	0.176683308	0.05	0.976335326
	IDP dMRI TBSS L2 Uncinate fasciculus L	Wald ratio	0.176683308	0.05	0.4286983
	IDP dMRI TBSS L3 Middle cerebellar peduncle	Wald ratio	0.02411879	0.05	0.003454463
	IDP dMRI TBSS L3 Genu of corpus callosum	Wald ratio	0.176683308	0.05	0.796510896
	IDP dMRI TBSS L3 Body of corpus callosum	Wald ratio	0.176683308	0.05	0.498201644
	IDP dMRI TBSS L3 Splenium of corpus callosum	Inverse variance weighted	0.323288521	0.05	0.693260958

	IDP dMRI TBSS L3 Inferior cerebellar peduncle R	Wald ratio	0.176683308	0.05	0.932590687
	IDP dMRI TBSS L3 Inferior cerebellar peduncle L	Wald ratio	0.176683308	0.05	0.873824912
	IDP dMRI TBSS L3 Superior cerebellar peduncle R	Inverse variance weighted	0.603416391	0.05	1
	IDP dMRI TBSS L3 Superior cerebellar peduncle L	Wald ratio	0.384175267	0.05	0.999979149
	IDP dMRI TBSS L3 Cerebral peduncle R	Wald ratio	0.628555875	0.05	1
	IDP dMRI TBSS L3 Anterior limb of internal capsule R	Wald ratio	0.294601378	0.05	0.087340342
	IDP dMRI TBSS L3 Anterior limb of internal capsule L	Wald ratio	0.328651352	0.05	0.115708419
	IDP dMRI TBSS L3 Retrolenticular part of internal capsule R	Inverse variance weighted	0.585463443	0.05	0.999979677
	IDP dMRI TBSS L3 Retrolenticular part of internal capsule L	Wald ratio	0.02411879	0.05	0.007060988
	IDP dMRI TBSS L3 Anterior corona radiata R	Inverse variance weighted	0.405979622	0.05	1
	IDP dMRI TBSS L3 Anterior corona radiata L	Inverse variance weighted	0.357070769	0.05	0.999999998
	IDP dMRI TBSS L3 Superior corona radiata R	Wald ratio	0.026610718	0.05	0.004609949
	IDP dMRI TBSS L3 Superior corona radiata L	Wald ratio	0.026610718	0.05	0.004299414
	IDP dMRI TBSS L3 Posterior corona radiata R	Inverse variance weighted	0.282388802	0.05	0.999915169
	IDP dMRI TBSS L3 Posterior corona radiata L	Wald ratio	0.02411879	0.05	0.004951879
	IDP dMRI TBSS L3 Posterior thalamic radiation R	Inverse variance weighted	0.589222356	0.05	0.999885329
	IDP dMRI TBSS L3 Posterior thalamic radiation L	Inverse variance weighted	0.54149606	0.05	0.998099715
	IDP dMRI TBSS L3 Sagittal stratum R	Inverse variance weighted	0.545049516	0.05	0.999784561

	IDP dMRI TBSS L3 Sagittal stratum L	Inverse variance weighted	0.453790377	0.05	0.994025081
	IDP dMRI TBSS L3 External capsule R	Wald ratio	0.176683308	0.05	0.732156617
	IDP dMRI TBSS L3 External capsule L	Wald ratio	0.176683308	0.05	0.803155077
	IDP dMRI TBSS L3 Cingulum cingulate gyrus R	Inverse variance weighted	0.372729525	0.05	1
	IDP dMRI TBSS L3 Cingulum cingulate gyrus L	Wald ratio	0.176683308	0.05	0.987157192
	IDP dMRI TBSS L3 Cingulum hippocampus R	Wald ratio	0.176683308	0.05	0.989850907
	IDP dMRI TBSS L3 Cingulum hippocampus L	Wald ratio	0.176683308	0.05	0.991834908
	IDP dMRI TBSS L3 Fornix cres+Stria terminalis R	Wald ratio	0.373193223	0.05	1
	IDP dMRI TBSS L3 Superior longitudinal fasciculus R	Inverse variance weighted	0.596218425	0.05	0.999952091
	IDP dMRI TBSS L3 Superior longitudinal fasciculus L	Inverse variance weighted	0.591325253	0.05	0.999994701
	IDP dMRI TBSS L3 Uncinate fasciculus R	Wald ratio	0.176683308	0.05	0.594207557
	IDP dMRI TBSS L3 Uncinate fasciculus L	Inverse variance weighted	0.27962173	0.05	0.997716789
	IDP dMRI TBSS ICVF Middle cerebellar peduncle	Inverse variance weighted	0.480224024	0.05	1
	IDP dMRI TBSS ICVF Genu of corpus callosum	MR Egger	0.166487158	0.05	1
		Weighted median	0.655017519	0.05	1
		Inverse variance weighted	0.888894825	0.05	1
		Simple mode	0.72502861	0.05	1
		Weighted mode	0.440256056	0.05	1
	IDP dMRI TBSS ICVF Body of corpus callosum	MR Egger	0.175196334	0.05	1
		Weighted median	0.942475052	0.05	1

		Inverse variance weighted	0.833443694	0.05	1
		Simple mode	0.542092028	0.05	0.999999999
		Weighted mode	0.864000394	0.05	1
	IDP dMRI TBSS ICVF Splenium of corpus callosum	MR Egger	0.125050101	0.05	1
		Weighted median	0.665211929	0.05	1
		Inverse variance weighted	0.919133282	0.05	1
		Simple mode	0.309543305	0.05	0.942733801
		Weighted mode	0.327990012	0.05	0.9515105
	IDP dMRI TBSS ICVF Fornix	Wald ratio	0.204490189	0.05	0.264906201
	IDP dMRI TBSS ICVF Medial lemniscus R	Inverse variance weighted	0.045419645	0.05	1
	IDP dMRI TBSS ICVF Medial lemniscus L	Wald ratio	0.0966751	0.05	1
	IDP dMRI TBSS ICVF Inferior cerebellar peduncle R	MR Egger	0.384428861	0.05	1
		Weighted median	0.895622655	0.05	1
		Inverse variance weighted	0.37118923	0.05	1
		Simple mode	0.904571694	0.05	1
		Weighted mode	0.844194259	0.05	1
	IDP dMRI TBSS ICVF Inferior cerebellar peduncle L	MR Egger	0.440331006	0.05	1
		Weighted median	0.97633333	0.05	1
		Inverse variance weighted	0.416347093	0.05	1
		Simple mode	0.907650603	0.05	1
		Weighted mode	0.838079368	0.05	1
		MR Egger	0.379235884	0.05	1

	IDP dMRI TBSS ICVF Superior cerebellar peduncle R	Weighted median	0.168104785	0.05	1
		Inverse variance weighted	0.319899804	0.05	1
		Simple mode	0.37135429	0.05	1
		Weighted mode	0.318522648	0.05	1
	IDP dMRI TBSS ICVF Superior cerebellar peduncle L	Inverse variance weighted	0.12655413	0.05	1
	IDP dMRI TBSS ICVF Cerebral peduncle R	MR Egger	0.775958883	0.05	1
		Weighted median	0.329098209	0.05	0.999989326
		Inverse variance weighted	0.838940736	0.05	1
		Simple mode	0.432822421	0.05	0.999664221
		Weighted mode	0.432296691	0.05	0.99997561
	IDP dMRI TBSS ICVF Cerebral peduncle L	Inverse variance weighted	0.690930827	0.05	1
	IDP dMRI TBSS ICVF Anterior limb of internal capsule R	Wald ratio	0.176683308	0.05	1
	IDP dMRI TBSS ICVF Anterior limb of internal capsule L	MR Egger	0.818361435	0.05	0.687027051
		Weighted median	0.423467964	0.05	1
		Inverse variance weighted	0.512324028	0.05	1
		Simple mode	0.275180164	0.05	0.823857291
		Weighted mode	0.358639754	0.05	1
	IDP dMRI TBSS ICVF Posterior limb of internal capsule R	MR Egger	0.139382949	0.05	1
		Weighted median	0.937066758	0.05	1
		Inverse variance weighted	0.725707811	0.05	1
		Simple mode	0.869078912	0.05	1
		Weighted mode	0.799803592	0.05	1

	IDP dMRI TBSS ICVF Posterior limb of internal capsule L	MR Egger	0.322807642	0.05	1
		Weighted median	0.650580981	0.05	1
		Inverse variance weighted	0.832205589	0.05	1
		Simple mode	0.851123206	0.05	1
		Weighted mode	0.432201614	0.05	1
	IDP dMRI TBSS ICVF Retrolenticular part of internal capsule R	MR Egger	0.187719182	0.05	1
		Weighted median	0.396797298	0.05	1
		Inverse variance weighted	0.839128484	0.05	1
		Simple mode	0.893445691	0.05	1
		Weighted mode	0.338883368	0.05	1
	IDP dMRI TBSS ICVF Retrolenticular part of internal capsule L	MR Egger	0.313087627	0.05	1
		Weighted median	0.494768281	0.05	1
		Inverse variance weighted	0.73475751	0.05	1
		Simple mode	0.996989932	0.05	1
		Weighted mode	0.409204244	0.05	1
	IDP dMRI TBSS ICVF Anterior corona radiata R	MR Egger	0.311607805	0.05	1
		Weighted median	0.592296964	0.05	1
		Inverse variance weighted	0.795952175	0.05	1
		Simple mode	0.965358977	0.05	1
		Weighted mode	0.433275575	0.05	1
	IDP dMRI TBSS ICVF Anterior corona radiata L	MR Egger	0.316241563	0.05	1
		Weighted median	0.54329188	0.05	1

		Inverse variance weighted	0.770453438	0.05	1
		Simple mode	0.963188981	0.05	1
		Weighted mode	0.40723187	0.05	1
	IDP dMRI TBSS ICVF Superior corona radiata R	MR Egger	0.308216053	0.05	1
		Weighted median	0.592433729	0.05	1
		Inverse variance weighted	0.809492812	0.05	1
		Simple mode	0.982654321	0.05	1
		Weighted mode	0.447415246	0.05	1
	IDP dMRI TBSS ICVF Superior corona radiata L	MR Egger	0.310128113	0.05	1
		Weighted median	0.579175924	0.05	1
		Inverse variance weighted	0.798969193	0.05	1
		Simple mode	0.975326767	0.05	1
		Weighted mode	0.436759233	0.05	1
	IDP dMRI TBSS ICVF Posterior corona radiata R	MR Egger	0.363695466	0.05	1
		Weighted median	0.722489419	0.05	1
		Inverse variance weighted	0.632814577	0.05	1
		Simple mode	0.650844169	0.05	1
		Weighted mode	0.312916205	0.05	1
	IDP dMRI TBSS ICVF Posterior corona radiata L	MR Egger	0.362393667	0.05	1
		Weighted median	0.633212788	0.05	1
		Inverse variance weighted	0.596223435	0.05	1
		Simple mode	0.669577453	0.05	1

		Weighted mode	0.347318898	0.05	1
	IDP dMRI TBSS ICVF Posterior thalamic radiation R	Inverse variance weighted	0.479209784	0.05	1
	IDP dMRI TBSS ICVF Posterior thalamic radiation L	MR Egger	0.269141963	0.05	1
		Weighted median	0.728739183	0.05	1
		Inverse variance weighted	0.723092302	0.05	1
		Simple mode	0.567937115	0.05	1
		Weighted mode	0.619258514	0.05	1
	IDP dMRI TBSS ICVF Sagittal stratum R	MR Egger	0.138693755	0.05	1
		Weighted median	0.978202945	0.05	1
		Inverse variance weighted	0.665284905	0.05	1
		Simple mode	0.881072236	0.05	1
		Weighted mode	0.670694865	0.05	1
	IDP dMRI TBSS ICVF Sagittal stratum L	MR Egger	0.248749823	0.05	1
		Weighted median	0.766117679	0.05	1
		Inverse variance weighted	0.66582546	0.05	1
		Simple mode	0.605613909	0.05	0.999999993
		Weighted mode	0.756343576	0.05	1
	IDP dMRI TBSS ICVF External capsule R	MR Egger	0.810520233	0.05	1
		Weighted median	0.230009888	0.05	1
		Inverse variance weighted	0.211351314	0.05	1
		Simple mode	0.278688173	0.05	1
		Weighted mode	0.342816776	0.05	1

	IDP dMRI TBSS ICVF External capsule L	Inverse variance weighted	0.468814637	0.05	1
	IDP dMRI TBSS ICVF Cingulum cingulate gyrus R	Inverse variance weighted	0.296694328	0.05	1
	IDP dMRI TBSS ICVF Cingulum cingulate gyrus L	Inverse variance weighted	0.013479139	0.05	1
	IDP dMRI TBSS ICVF Cingulum hippocampus R	MR Egger	0.35432058	0.05	1
		Weighted median	0.393550446	0.05	1
		Inverse variance weighted	0.475017177	0.05	1
		Simple mode	0.806919607	0.05	1
		Weighted mode	0.356571808	0.05	1
	IDP dMRI TBSS ICVF Cingulum hippocampus L	MR Egger	0.31644929	0.05	1
		Weighted median	0.440496115	0.05	1
		Inverse variance weighted	0.696848659	0.05	1
		Simple mode	0.997575679	0.05	1
		Weighted mode	0.386033733	0.05	1
	IDP dMRI TBSS ICVF Fornix cres+Stria terminalis R	Inverse variance weighted	0.263578272	0.05	1
	IDP dMRI TBSS ICVF Fornix cres+Stria terminalis L	Wald ratio	0.026610718	0.05	1
	IDP dMRI TBSS ICVF Superior longitudinal fasciculus R	MR Egger	0.244264793	0.05	1
		Weighted median	0.954887265	0.05	1
		Inverse variance weighted	0.846937194	0.05	1
		Simple mode	0.603549323	0.05	0.999999999
		Weighted mode	0.753536409	0.05	1
		MR Egger	0.306881037	0.05	1

	IDP dMRI TBSS ICVF Superior longitudinal fasciculus L	Weighted median	0.583655938	0.05	1
		Inverse variance weighted	0.789261422	0.05	1
		Simple mode	0.964536693	0.05	1
		Weighted mode	0.471495571	0.05	1
	IDP dMRI TBSS ICVF Superior fronto-occipital fasciculus R	Inverse variance weighted	0.094914833	0.05	1
	IDP dMRI TBSS ICVF Superior fronto-occipital fasciculus L	Wald ratio	0.026610718	0.05	1
	IDP dMRI TBSS ICVF Uncinate fasciculus R	Wald ratio	0.176683308	0.05	1
	IDP dMRI TBSS ICVF Uncinate fasciculus L	Wald ratio	0.176683308	0.05	1
	IDP dMRI TBSS ICVF Tapetum R	Wald ratio	0.02411879	0.05	1
	IDP dMRI TBSS ICVF Tapetum L	Wald ratio	0.02411879	0.05	1
	IDP dMRI TBSS OD Pontine crossing tract	MR Egger	0.663112665	0.05	1
		Weighted median	0.236723489	0.05	1
		Inverse variance weighted	0.151692608	0.05	1
		Simple mode	0.413284319	0.05	1
		Weighted mode	0.437729203	0.05	1
	IDP dMRI TBSS OD Genu of corpus callosum	Wald ratio	0.177287381	0.05	1
	IDP dMRI TBSS OD Cerebral peduncle R	Wald ratio	0.365143154	0.05	0.597238655
	IDP dMRI TBSS OD Cerebral peduncle L	Wald ratio	0.365143154	0.05	0.569451879
	IDP dMRI TBSS OD Posterior limb of internal capsule L	Wald ratio	0.576055989	0.05	1
	IDP dMRI TBSS OD Posterior corona radiata R	Wald ratio	0.40617903	0.05	1
	IDP dMRI TBSS OD External capsule R	Inverse variance weighted	0.085115326	0.05	1

	IDP dMRI TBSS OD External capsule L	Wald ratio	0.451437815	0.05	1
	IDP dMRI TBSS OD Superior longitudinal fasciculus R	Wald ratio	0.487506153	0.05	1
	IDP dMRI TBSS ISOVF Fornix	Wald ratio	0.049762082	0.05	1
	IDP dMRI TBSS ISOVF External capsule R	Wald ratio	0.910722674	0.05	1
	IDP dMRI TBSS ISOVF Cingulum cingulate gyrus R	Inverse variance weighted	0.726006983	0.05	1
	IDP dMRI ProbtrackX FA ar l	Wald ratio	0.176683308	0.05	1
	IDP dMRI ProbtrackX FA atr l	Wald ratio	0.176683308	0.05	1
	IDP dMRI ProbtrackX FA atr r	Wald ratio	0.02411879	0.05	1
	IDP dMRI ProbtrackX FA fma	Wald ratio	0.02411879	0.05	1
	IDP dMRI ProbtrackX FA fmi	MR Egger	0.259949297	0.05	1
		Weighted median	0.448636067	0.05	1
		Inverse variance weighted	0.778931348	0.05	1
		Simple mode	0.681559834	0.05	1
		Weighted mode	0.401586595	0.05	1
	IDP dMRI ProbtrackX FA ifo l	Inverse variance weighted	0.471739022	0.05	1
	IDP dMRI ProbtrackX FA ifo r	Inverse variance weighted	0.634027349	0.05	1
	IDP dMRI ProbtrackX FA ilf l	Inverse variance weighted	0.182015705	0.05	1
	IDP dMRI ProbtrackX FA ilf r	Inverse variance weighted	0.188787085	0.05	1
	IDP dMRI ProbtrackX FA ml l	Wald ratio	0.960975349	0.05	1
	IDP dMRI ProbtrackX FA ptr l	Wald ratio	0.026610718	0.05	1
	IDP dMRI ProbtrackX FA ptr r	Wald ratio	0.360735191	0.05	1

	IDP dMRI ProbtrackX FA slf l	Inverse variance weighted	0.650975836	0.05	1
	IDP dMRI ProbtrackX FA slf r	Wald ratio	0.02411879	0.05	1
	IDP dMRI ProbtrackX FA str l	Wald ratio	0.154142363	0.05	1
	IDP dMRI ProbtrackX FA unc l	Wald ratio	0.176683308	0.05	1
	IDP dMRI ProbtrackX FA unc r	Wald ratio	0.176683308	0.05	1
	IDP dMRI ProbtrackX MD ar l	Wald ratio	0.176683308	0.05	0.633098048
	IDP dMRI ProbtrackX MD ar r	Wald ratio	0.176683308	0.05	0.599836355
	IDP dMRI ProbtrackX MD atr l	Wald ratio	0.176683308	0.05	0.702969799
	IDP dMRI ProbtrackX MD atr r	Wald ratio	0.176683308	0.05	0.558113294
	IDP dMRI ProbtrackX MD cgc l	MR Egger	0.470019617	0.05	0.012924998
		Weighted median	0.94996969	0.05	1
		Inverse variance weighted	0.601296601	0.05	1
		Simple mode	0.697811904	0.05	1
		Weighted mode	0.698338257	0.05	1
	IDP dMRI ProbtrackX MD cgc r	MR Egger	0.338014326	0.05	0.002550026
		Weighted median	0.666477921	0.05	1
		Inverse variance weighted	0.680227821	0.05	1
		Simple mode	0.729967174	0.05	1
		Weighted mode	0.697234649	0.05	1
	IDP dMRI ProbtrackX MD fmi	Inverse variance weighted	0.370861412	0.05	1
	IDP dMRI ProbtrackX MD ifo l	Inverse variance weighted	0.531790018	0.05	0.999950855
	IDP dMRI ProbtrackX MD ifo r	Inverse variance weighted	0.550258085	0.05	0.999762382

	IDP dMRI ProbtrackX MD ilf l	Inverse variance weighted	0.536129679	0.05	0.999601263
	IDP dMRI ProbtrackX MD ilf r	Inverse variance weighted	0.490718176	0.05	0.996143421
	IDP dMRI ProbtrackX MD ptr l	Wald ratio	0.176683308	0.05	0.739983219
	IDP dMRI ProbtrackX MD ptr r	Wald ratio	0.176683308	0.05	0.871880204
	IDP dMRI ProbtrackX MD slf l	Inverse variance weighted	0.398170193	0.05	1
	IDP dMRI ProbtrackX MD slf r	Inverse variance weighted	0.576487748	0.05	0.997452663
	IDP dMRI ProbtrackX MD str l	Wald ratio	0.341821646	0.05	0.096129589
	IDP dMRI ProbtrackX MD str r	Wald ratio	0.176683308	0.05	0.291677737
	IDP dMRI ProbtrackX MD unc l	Wald ratio	0.176683308	0.05	0.88860736
	IDP dMRI ProbtrackX MD unc r	Inverse variance weighted	0.167739091	0.05	0.999582623
	IDP dMRI ProbtrackX MO atr l	Wald ratio	0.220951176	0.05	1
	IDP dMRI ProbtrackX MO ml r	Wald ratio	0.372008203	0.05	0.418816881
	IDP T1 FAST ROIs L intracalc cortex	Wald ratio	0.95910077	0.05	1
	IDP dMRI ProbtrackX L1 fmi	Wald ratio	0.026610718	0.05	0.003609694
	IDP dMRI ProbtrackX L1 ifo l	Wald ratio	0.026610718	0.05	0.004234174
	IDP dMRI ProbtrackX L1 ifo r	Wald ratio	0.026610718	0.05	0.003909816
	IDP dMRI ProbtrackX L1 ilf l	Wald ratio	0.176683308	0.05	0.579096528
	IDP dMRI ProbtrackX L1 ilf r	Wald ratio	0.026610718	0.05	0.004178739
	IDP T1 FAST ROIs R intracalc cortex	Wald ratio	0.95910077	0.05	1
	IDP dMRI ProbtrackX L1 slf l	Wald ratio	0.176683308	0.05	0.536947435
	IDP dMRI ProbtrackX L1 slf r	Inverse variance weighted	0.133433167	0.05	0.015112943
	IDP dMRI ProbtrackX L1 str r	Wald ratio	0.034881546	0.05	1

	IDP dMRI ProbtrackX L1 unc l	Inverse variance weighted	0.629017094	0.05	1
	IDP dMRI ProbtrackX L1 unc r	Inverse variance weighted	0.098980146	0.05	0.056902998
	IDP dMRI ProbtrackX L2 ar l	Wald ratio	0.176683308	0.05	0.565767935
	IDP dMRI ProbtrackX L2 ar r	Wald ratio	0.176683308	0.05	0.540806551
	IDP dMRI ProbtrackX L2 atr l	Wald ratio	0.176683308	0.05	0.825906298
	IDP dMRI ProbtrackX L2 atr r	Wald ratio	0.176683308	0.05	0.747682729
	IDP dMRI ProbtrackX L2 cgc l	Wald ratio	0.026610718	0.05	0.005053502
	IDP dMRI ProbtrackX L2 cgc r	Wald ratio	0.026610718	0.05	0.008585417
	IDP dMRI ProbtrackX L2 cgh l	Wald ratio	0.719947897	0.05	1
	IDP dMRI ProbtrackX L2 fma	Wald ratio	0.093208244	0.05	1
	IDP dMRI ProbtrackX L2 fmi	Inverse variance weighted	0.373301721	0.05	1
	IDP dMRI ProbtrackX L2 ifo l	Inverse variance weighted	0.547627832	0.05	0.999985575
	IDP dMRI ProbtrackX L2 ifo r	Inverse variance weighted	0.575635219	0.05	0.999971256
	IDP dMRI ProbtrackX L2 ilf l	Inverse variance weighted	0.531904524	0.05	0.999598914
	IDP dMRI ProbtrackX L2 ilf r	Inverse variance weighted	0.503830379	0.05	0.999473759
	IDP dMRI ProbtrackX L2 ptr l	Wald ratio	0.176683308	0.05	0.87285528
	IDP dMRI ProbtrackX L2 ptr r	Wald ratio	0.184238032	0.05	0.974333249
	IDP dMRI ProbtrackX L2 slf l	Inverse variance weighted	0.405119893	0.05	1
	IDP dMRI ProbtrackX L2 slf r	Inverse variance weighted	0.603932413	0.05	0.99970351
	IDP dMRI ProbtrackX L2 str l	Wald ratio	0.341821646	0.05	0.259839839
	IDP dMRI ProbtrackX L2 str r	Wald ratio	0.341821646	0.05	0.187206081

	IDP dMRI ProbtrackX L2 unc l	Wald ratio	0.176683308	0.05	0.882308306
	IDP dMRI ProbtrackX L2 unc r	Inverse variance weighted	0.166058995	0.05	0.99898633
	IDP dMRI ProbtrackX L3 ar l	Wald ratio	0.176683308	0.05	0.687814545
	IDP dMRI ProbtrackX L3 ar r	Wald ratio	0.176683308	0.05	0.594207557
	IDP dMRI ProbtrackX L3 atr l	Wald ratio	0.176683308	0.05	0.883224077
	IDP dMRI ProbtrackX L3 atr r	Wald ratio	0.176683308	0.05	0.817276533
	IDP dMRI ProbtrackX L3 cgc l	Wald ratio	0.02411879	0.05	0.006261888
	IDP dMRI ProbtrackX L3 cgc r	Wald ratio	0.02411879	0.05	0.007288232
	IDP dMRI ProbtrackX L3 fmi	MR Egger	0.310008846	0.05	0.003251635
		Weighted median	0.694330118	0.05	1
		Inverse variance weighted	0.857870478	0.05	1
		Simple mode	0.895585585	0.05	1
		Weighted mode	0.513483963	0.05	1
	IDP dMRI ProbtrackX L3 ifo l	Inverse variance weighted	0.335242709	0.05	1
	IDP dMRI ProbtrackX L3 ifo r	Inverse variance weighted	0.518226458	0.05	0.999665573
	IDP dMRI ProbtrackX L3 ilf l	Inverse variance weighted	0.498921957	0.05	0.999388778
	IDP dMRI ProbtrackX L3 ilf r	Inverse variance weighted	0.461908505	0.05	0.996539887
	IDP dMRI ProbtrackX L3 ptr l	Wald ratio	0.176683308	0.05	0.914203488
	IDP dMRI ProbtrackX L3 ptr r	Wald ratio	0.184238032	0.05	0.970927229
	IDP dMRI ProbtrackX L3 slf l	Inverse variance weighted	0.384963166	0.05	1
	IDP dMRI ProbtrackX L3 slf r	Inverse variance weighted	0.5568049	0.05	0.995581718

	IDP dMRI ProbtrackX L3 str l	Wald ratio	0.184238032	0.05	0.339279519
	IDP dMRI ProbtrackX L3 str r	Wald ratio	0.026610718	0.05	0.003302918
	IDP dMRI ProbtrackX L3 unc l	Wald ratio	0.176683308	0.05	0.851221248
	IDP dMRI ProbtrackX L3 unc r	Inverse variance weighted	0.170981363	0.05	0.999606609
	IDP dMRI ProbtrackX ICVF ar l	MR Egger	0.629664862	0.05	1
		Weighted median	0.156350119	0.05	1
		Inverse variance weighted	0.445542485	0.05	1
		Simple mode	0.294385226	0.05	1
		Weighted mode	0.280912353	0.05	1
	IDP dMRI ProbtrackX ICVF ar r	MR Egger	0.600566991	0.05	1
		Weighted median	0.136668275	0.05	1
		Inverse variance weighted	0.435040212	0.05	1
		Simple mode	0.279856311	0.05	1
		Weighted mode	0.281134197	0.05	1
	IDP dMRI ProbtrackX ICVF atr l	MR Egger	0.324100839	0.05	1
		Weighted median	0.490040527	0.05	1
		Inverse variance weighted	0.728695228	0.05	1
		Simple mode	0.951103079	0.05	1
		Weighted mode	0.378995479	0.05	1
	IDP dMRI ProbtrackX ICVF atr r	MR Egger	0.325880827	0.05	1
		Weighted median	0.52490013	0.05	1
		Inverse variance weighted	0.75477593	0.05	1

		Simple mode	0.91800514	0.05	1
		Weighted mode	0.424684921	0.05	1
	IDP dMRI ProbtrackX ICVF cgc l	Inverse variance weighted	0.014050859	0.05	1
	IDP dMRI ProbtrackX ICVF cgc r	Wald ratio	0.026610718	0.05	1
	IDP dMRI ProbtrackX ICVF cgh l	MR Egger	0.326272661	0.05	1
		Weighted median	0.510042634	0.05	1
		Inverse variance weighted	0.729620579	0.05	1
		Simple mode	0.933433258	0.05	1
		Weighted mode	0.37176387	0.05	1
	IDP dMRI ProbtrackX ICVF cgh r	Wald ratio	0.176683308	0.05	1
	IDP dMRI ProbtrackX ICVF cst l	MR Egger	0.605701308	0.05	1
		Weighted median	0.141444143	0.05	1
		Inverse variance weighted	0.478990903	0.05	1
		Simple mode	0.285381503	0.05	1
		Weighted mode	0.290615646	0.05	1
	IDP dMRI ProbtrackX ICVF cst r	MR Egger	0.2032559	0.05	1
		Weighted median	0.937563665	0.05	1
		Inverse variance weighted	0.990720642	0.05	1
		Simple mode	0.484598689	0.05	0.999983129
		Weighted mode	0.338167646	0.05	1
	IDP dMRI ProbtrackX ICVF fma	MR Egger	0.282368389	0.05	1
		Weighted median	0.516369571	0.05	1

		Inverse variance weighted	0.258368731	0.05	1
		Simple mode	0.94823888	0.05	1
		Weighted mode	0.974099132	0.05	1
	IDP dMRI ProbtrackX ICVF fmi	MR Egger	0.231006014	0.05	1
		Weighted median	0.278235215	0.05	1
		Inverse variance weighted	0.656645969	0.05	1
		Simple mode	0.66733116	0.05	1
		Weighted mode	0.312084865	0.05	1
	IDP dMRI ProbtrackX ICVF ifo l	Inverse variance weighted	0.307584311	0.05	1
	IDP dMRI ProbtrackX ICVF ifo r	Inverse variance weighted	0.309990284	0.05	1
	IDP dMRI ProbtrackX ICVF ilf l	Inverse variance weighted	0.317613589	0.05	1
	IDP dMRI ProbtrackX ICVF ilf r	Inverse variance weighted	0.43700449	0.05	1
	IDP dMRI ProbtrackX ICVF mcp	Inverse variance weighted	0.537144229	0.05	1
	IDP dMRI ProbtrackX ICVF ml l	Wald ratio	0.244021532	0.05	1
	IDP dMRI ProbtrackX ICVF ml r	Wald ratio	0.176683308	0.05	1
	IDP dMRI ProbtrackX ICVF ptr l	Inverse variance weighted	0.419687075	0.05	1
	IDP dMRI ProbtrackX ICVF ptr r	MR Egger	0.27503451	0.05	1
		Weighted median	0.401357505	0.05	1
		Inverse variance weighted	0.338559475	0.05	1
		Simple mode	0.790698424	0.05	1
		Weighted mode	0.837368	0.05	1

	IDP dMRI ProbtrackX ICVF slf l	MR Egger	0.309874729	0.05	1
		Weighted median	0.549676058	0.05	1
		Inverse variance weighted	0.775390275	0.05	1
		Simple mode	0.999393664	0.05	1
		Weighted mode	0.426153636	0.05	1
	IDP dMRI ProbtrackX ICVF slf r	MR Egger	0.236063584	0.05	1
		Weighted median	0.854997834	0.05	1
		Inverse variance weighted	0.787531641	0.05	1
		Simple mode	0.602833174	0.05	0.999999983
		Weighted mode	0.846957793	0.05	1
	IDP dMRI ProbtrackX ICVF str l	MR Egger	0.319806974	0.05	1
		Weighted median	0.606368513	0.05	1
		Inverse variance weighted	0.812829887	0.05	1
		Simple mode	0.899823273	0.05	1
		Weighted mode	0.388728191	0.05	1
	IDP dMRI ProbtrackX ICVF str r	MR Egger	0.312770135	0.05	1
		Weighted median	0.597374636	0.05	1
		Inverse variance weighted	0.800017993	0.05	1
		Simple mode	0.960079257	0.05	1
		Weighted mode	0.42356338	0.05	1
	IDP dMRI ProbtrackX ICVF unc l	Inverse variance weighted	0.712320014	0.05	1
	IDP dMRI ProbtrackX ICVF unc r	Inverse variance weighted	0.272546604	0.05	1

	IDP dMRI ProbtrackX OD atr r	Wald ratio	0.872687938	0.05	1
	IDP dMRI ProbtrackX OD ilf l	Wald ratio	0.939160466	0.05	1
	IDP dMRI ProbtrackX OD slf l	Wald ratio	0.48331444	0.05	1
	IDP dMRI ProbtrackX OD str l	Inverse variance weighted	0.648298292	0.05	0.99998291
	IDP dMRI ProbtrackX ISOVF atr r	Wald ratio	0.176683308	0.05	1
	IDP dMRI ProbtrackX ISOVF cgc l	Wald ratio	0.940414624	0.05	1
	IDP dMRI ProbtrackX ISOVF ifo r	Inverse variance weighted	0.172893582	0.05	1
	IDP dMRI ProbtrackX ISOVF ilf l	Wald ratio	0.067735641	0.05	0.005031556
	IDP dMRI ProbtrackX ISOVF ilf r	MR Egger	0.849871015	0.05	0.002544838
		Weighted median	0.862840025	0.05	1
		Inverse variance weighted	0.867568172	0.05	1
		Simple mode	0.566365623	0.05	0.998986212
		Weighted mode	0.596375289	0.05	0.999878166
	IDP dMRI ProbtrackX ISOVF slf l	Inverse variance weighted	0.811695685	0.05	1
	IDP dMRI ProbtrackX ISOVF slf r	MR Egger	0.474890712	0.05	1
		Weighted median	0.591678686	0.05	1
		Inverse variance weighted	0.337558126	0.05	0.999995529
		Simple mode	0.81097203	0.05	1
		Weighted mode	0.960968557	0.05	1
	IDP dMRI ProbtrackX ISOVF unc l	Wald ratio	0.685887833	0.05	1
	IDP dMRI ProbtrackX ISOVF unc r	Wald ratio	0.682375588	0.05	1
	IDP T1 SIENAX brain-normalised volume	Wald ratio	0.898036169	0.05	1

Sjogren's syndrome (Firth correction)	IDP T1 SIENAX peripheral grey normalised volume	Wald ratio	0.309627019	0.05	0.999878742
	IDP T1 FAST ROIs R temp occ fusif cortex	Wald ratio	0.664628896	0.05	1
	IDP T1 FAST ROIs R occ pole	Wald ratio	0.00590805	0.05	1
	IDP T1 FAST ROIs L thalamus	Inverse variance weighted	0.240037959	0.05	1
	IDP T1 FAST ROIs R thalamus	Wald ratio	0.123439701	0.05	1
	IDP T1 FAST ROIs L putamen	Wald ratio	0.653778307	0.05	1
	IDP T1 FAST ROIs R putamen	Wald ratio	0.653778307	0.05	1
	IDP T1 FAST ROIs L pallidum	Wald ratio	0.909259828	0.05	1
	IDP T1 FIRST left caudate volume	Wald ratio	0.66878646	0.05	1
	IDP T1 FAST ROIs L hippocampus	Inverse variance weighted	0.175179698	0.05	1
	IDP T1 FAST ROIs R hippocampus	Wald ratio	0.615143272	0.05	1
	IDP T1 FAST ROIs L ventral striatum	Wald ratio	0.653778307	0.05	1
	IDP T1 FAST ROIs R ventral striatum	Inverse variance weighted	0.554961952	0.05	1
	IDP T1 FAST ROIs L cerebellum crus I	MR Egger	0.788301657	0.05	1
		Weighted median	0.535970884	0.05	1
		Inverse variance weighted	0.91981875	0.05	1
		Simple mode	0.601147263	0.05	1
		Weighted mode	0.590029067	0.05	1
	IDP T1 FAST ROIs R cerebellum crus I	Inverse variance weighted	0.634066218	0.05	1
	IDP T1 FAST ROIs L cerebellum crus II	MR Egger	0.380219824	0.05	0.223889041
		Weighted median	0.909297958	0.05	1
		Inverse variance weighted	0.518956565	0.05	1

		Simple mode	0.833313681	0.05	1
		Weighted mode	0.841480444	0.05	1
	IDP T1 FAST ROIs V cerebellum crus II	Inverse variance weighted	0.244507789	0.05	1
	IDP T1 FAST ROIs R cerebellum crus II	Inverse variance weighted	0.463808649	0.05	1
	IDP T1 FAST ROIs V cerebellum VIIIa	MR Egger	0.634806621	0.05	1
		Weighted median	0.620003205	0.05	1
		Inverse variance weighted	0.442109139	0.05	1
		Simple mode	0.967326305	0.05	1
		Weighted mode	0.954377412	0.05	1
	IDP T1 FAST ROIs L cerebellum VIIIb	Wald ratio	0.952995872	0.05	1
	IDP T1 FAST ROIs V cerebellum VIIIb	Inverse variance weighted	0.455732074	0.05	1
	IDP T1 FAST ROIs R cerebellum VIIIb	Wald ratio	0.653778307	0.05	1
	IDP T1 FAST ROIs L cerebellum IX	Wald ratio	0.952995872	0.05	1
	IDP T1 FAST ROIs V cerebellum IX	Inverse variance weighted	0.24498095	0.05	1
	IDP T1 FAST ROIs R cerebellum IX	Inverse variance weighted	0.352108962	0.05	1
	IDP T1 FAST ROIs V cerebellum X	Wald ratio	0.653778307	0.05	1
	IDP T2 FLAIR BIANCA WMH volume	Inverse variance weighted	0.506717162	0.05	1
	IDP SWI T2star left caudate	MR Egger	0.445557822	0.05	1
		Weighted median	0.165305356	0.05	1
		Inverse variance weighted	0.285581872	0.05	1
		Simple mode	0.553135813	0.05	1

		Weighted mode	0.163827153	0.05	1
	IDP SWI T2star right caudate	MR Egger	0.592652534	0.05	1
		Weighted median	0.41540045	0.05	1
		Inverse variance weighted	0.726107168	0.05	1
		Simple mode	0.720635673	0.05	1
		Weighted mode	0.484260137	0.05	1
	IDP T1 FIRST left pallidum volume	Wald ratio	0.64255429	0.05	1
	IDP SWI T2star left putamen	MR Egger	0.586823808	0.05	1
		Weighted median	0.28460598	0.05	1
		Inverse variance weighted	0.484246457	0.05	1
		Simple mode	0.491948607	0.05	1
		Weighted mode	0.433510408	0.05	1
	IDP SWI T2star right putamen	MR Egger	0.400601723	0.05	0.999977805
		Weighted median	0.184212814	0.05	1
		Inverse variance weighted	0.200968111	0.05	1
		Simple mode	0.375484487	0.05	1
		Weighted mode	0.381368068	0.05	1
	IDP SWI T2star left pallidum	MR Egger	0.65012788	0.05	1
		Weighted median	0.743960559	0.05	1
		Inverse variance weighted	0.302259321	0.05	1
		Simple mode	0.218100472	0.05	1
		Weighted mode	0.786767182	0.05	1

	IDP SWI T2star right pallidum	MR Egger	0.716913761	0.05	1
		Weighted median	0.095626297	0.05	1
		Inverse variance weighted	0.168176309	0.05	1
		Simple mode	0.25851442	0.05	1
		Weighted mode	0.238443399	0.05	1
	IDP T1 FIRST right pallidum volume	Wald ratio	0.64255429	0.05	1
	IDP dMRI TBSS FA Genu of corpus callosum	Wald ratio	0.962479881	0.05	1
	IDP dMRI TBSS FA Splenium of corpus callosum	MR Egger	0.737938395	0.05	0.999999984
		Weighted median	0.875061012	0.05	1
		Inverse variance weighted	0.6329541	0.05	1
		Simple mode	0.928743718	0.05	1
		Weighted mode	0.926679824	0.05	1
	IDP dMRI TBSS FA Corticospinal tract L	Wald ratio	0.454827554	0.05	1
	IDP dMRI TBSS FA Superior cerebellar peduncle R	Inverse variance weighted	0.692009832	0.05	1
	IDP dMRI TBSS FA Superior cerebellar peduncle L	Inverse variance weighted	0.74573576	0.05	1
	IDP dMRI TBSS FA Posterior limb of internal capsule R	Wald ratio	0.710926917	0.05	1
	IDP dMRI TBSS FA Retrolenticular part of internal capsule R	Inverse variance weighted	0.194318867	0.05	1
	IDP dMRI TBSS FA Retrolenticular part of internal capsule L	Wald ratio	0.905356301	0.05	1
	IDP dMRI TBSS FA Anterior corona radiata R	Wald ratio	0.962479881	0.05	1
	IDP dMRI TBSS FA Anterior corona radiata L	Wald ratio	0.962479881	0.05	1

	IDP dMRI TBSS FA Posterior corona radiata L	Wald ratio	0.905356301	0.05	1
	IDP dMRI TBSS FA Posterior thalamic radiation R	Inverse variance weighted	0.365984754	0.05	1
	IDP dMRI TBSS FA Posterior thalamic radiation L	Inverse variance weighted	0.350035492	0.05	1
	IDP dMRI TBSS FA Sagittal stratum R	Inverse variance weighted	0.965979935	0.05	1
	IDP dMRI TBSS FA Sagittal stratum L	Wald ratio	0.988397064	0.05	1
	IDP dMRI TBSS FA Cingulum cingulate gyrus R	Inverse variance weighted	0.970233377	0.05	1
	IDP dMRI TBSS FA Superior longitudinal fasciculus L	Inverse variance weighted	0.895914281	0.05	1
	IDP dMRI TBSS FA Uncinate fasciculus L	Wald ratio	0.31812375	0.05	1
	IDP dMRI TBSS MD Genu of corpus callosum	Wald ratio	0.962479881	0.05	1
	IDP dMRI TBSS MD Body of corpus callosum	Wald ratio	0.828801408	0.05	1
	IDP dMRI TBSS MD Splenium of corpus callosum	Inverse variance weighted	0.873361697	0.05	1
	IDP dMRI TBSS MD Corticospinal tract R	Wald ratio	0.082484342	0.05	1
	IDP dMRI TBSS MD Inferior cerebellar peduncle R	Inverse variance weighted	0.540601902	0.05	1
	IDP dMRI TBSS MD Inferior cerebellar peduncle L	Wald ratio	0.905356301	0.05	1
	IDP dMRI TBSS MD Superior cerebellar peduncle L	Wald ratio	0.554387065	0.05	1
	IDP dMRI TBSS MD Anterior limb of internal capsule L	Wald ratio	0.378551231	0.05	1
	IDP dMRI TBSS MD Retrolenticular part of internal capsule R	Inverse variance weighted	0.29933982	0.05	1
	IDP T1 FIRST left caudate volume plus IDP T1 FIRST right caudate volume	Wald ratio	0.566380432	0.05	1
	IDP T1 FIRST left putamen volume plus IDP T1 FIRST right putamen volume	Wald ratio	0.402467648	0.05	1

	IDP T1 FIRST left pallidum volume plus IDP T1 FIRST right pallidum volume	Wald ratio	0.64255429	0.05	1
	IDP SWI T2star left thalamus plus IDP SWI T2star right thalamus	MR Egger	0.496764146	0.05	0.999991802
		Weighted median	0.621467338	0.05	1
		Inverse variance weighted	0.490039042	0.05	1
		Simple mode	0.675660109	0.05	1
		Weighted mode	0.779078592	0.05	1
	IDP dMRI TBSS MD Retrolenticular part of internal capsule L	Inverse variance weighted	0.344258867	0.05	1
	IDP SWI T2star left caudate plus IDP SWI T2star right caudate	MR Egger	0.629966819	0.05	1
		Weighted median	0.231818961	0.05	1
		Inverse variance weighted	0.864886057	0.05	1
		Simple mode	0.529365789	0.05	1
		Weighted mode	0.365182581	0.05	1
	IDP SWI T2star left putamen plus IDP SWI T2star right putamen	MR Egger	0.507182342	0.05	1
		Weighted median	0.239996249	0.05	1
		Inverse variance weighted	0.289600577	0.05	1
		Simple mode	0.440292253	0.05	1
		Weighted mode	0.423758037	0.05	1
	IDP SWI T2star left pallidum plus IDP SWI T2star right pallidum	MR Egger	0.727664516	0.05	1
		Weighted median	0.07666658	0.05	1
		Inverse variance weighted	0.12810976	0.05	1
		Simple mode	0.160948154	0.05	1
		Weighted mode	0.191756249	0.05	1

	volume Left-Lateral-Ventricle	MR Egger	0.339202911	0.05	1
		Weighted median	0.126214803	0.05	0.999899647
		Inverse variance weighted	0.517380583	0.05	1
		Simple mode	0.321055095	0.05	0.999576237
		Weighted mode	0.323309209	0.05	0.999511638
	volume Left-Cerebellum-White-Matter	Inverse variance weighted	0.056226025	0.05	1
	volume Left-Cerebellum-Cortex	Wald ratio	0.619077279	0.05	1
	IDP dMRI TBSS MD Anterior corona radiata R	Inverse variance weighted	0.384211957	0.05	1
	volume Left-Putamen	Wald ratio	0.861061942	0.05	1
	volume 3rd-Ventricle	Wald ratio	0.419463061	0.05	0.999999993
	volume 4th-Ventricle	Inverse variance weighted	0.15043181	0.05	1
	volume Brain-Stem	Inverse variance weighted	0.839472062	0.05	1
	volume Left-Hippocampus	Inverse variance weighted	0.969874679	0.05	1
	volume CSF	MR Egger	0.896585307	0.05	0.007065327
		Weighted median	0.449094161	0.05	1
		Inverse variance weighted	0.295879886	0.05	1
		Simple mode	0.652012752	0.05	1
		Weighted mode	0.614752216	0.05	1
	IDP dMRI TBSS MD Anterior corona radiata L	Inverse variance weighted	0.427405886	0.05	1
	volume Left-Accumbens-area	Wald ratio	0.653778307	0.05	1
	volume Right-Lateral-Ventricle	Inverse variance weighted	0.95719212	0.05	1

	volume Right-Inf-Lat-Vent	Wald ratio	0.018511076	0.05	1
	volume Right-Cerebellum-White-Matter	Wald ratio	0.148541807	0.05	1
	volume Right-Cerebellum-Cortex	MR Egger	0.853109837	0.05	1
		Weighted median	0.624534334	0.05	1
		Inverse variance weighted	0.710989537	0.05	1
		Simple mode	0.544808991	0.05	1
		Weighted mode	0.60846866	0.05	1
	IDP dMRI TBSS MD Superior corona radiata R	Inverse variance weighted	0.38507014	0.05	1
	volume Right-Hippocampus	Wald ratio	0.615143272	0.05	1
	volume Right-Amygdala	Wald ratio	0.899573732	0.05	1
	volume CC Posterior	Wald ratio	0.899834625	0.05	1
	IDP dMRI TBSS MD Superior corona radiata L	Inverse variance weighted	0.38967743	0.05	1
	IDP dMRI TBSS MD Posterior corona radiata R	Inverse variance weighted	0.429033753	0.05	1
	volume BrainSegVol-to-eTIV	Wald ratio	0.960558714	0.05	1
	IDP dMRI TBSS MD Posterior corona radiata L	Inverse variance weighted	0.370742828	0.05	1
	volume rhSurfaceHoles	Wald ratio	0.75237458	0.05	1
	DKTatlas lh cuneus area	Wald ratio	0.327421424	0.05	1
	IDP dMRI TBSS MD Posterior thalamic radiation R	Inverse variance weighted	0.316484081	0.05	1
	DKTatlas lh lateraloccipital area	Wald ratio	0.327421424	0.05	1
	DKTatlas lh lingual area	Wald ratio	0.373188369	0.05	1
	IDP dMRI TBSS MD Posterior thalamic radiation L	Inverse variance weighted	0.403369672	0.05	1
	DKTatlas lh parstriangularis area	Wald ratio	0.270964985	0.05	1

	DKTatlas lh pericalcarine area	Inverse variance weighted	0.300391833	0.05	1
	DKTatlas lh postcentral area	Inverse variance weighted	0.259498558	0.05	1
	DKTatlas lh posteriorcingulate area	Wald ratio	0.372645934	0.05	0.999999997
	DKTatlas lh precentral area	Wald ratio	0.417238574	0.05	1
	DKTatlas lh precuneus area	Wald ratio	0.453652472	0.05	0.999999999
	IDP dMRI TBSS MD Sagittal stratum R	Inverse variance weighted	0.381426039	0.05	1
	DKTatlas lh superiortemporal area	Wald ratio	0.529259457	0.05	1
	DKTatlas lh supramarginal area	Wald ratio	0.040057223	0.05	0.479163056
	DKTatlas lh WhiteSurfArea area	Wald ratio	0.633152834	0.05	1
	IDP dMRI TBSS MD Sagittal stratum L	Inverse variance weighted	0.49198995	0.05	1
	a2009s lh G&S subcentral area	Wald ratio	0.417238574	0.05	1
	a2009s lh G cuneus area	Wald ratio	0.477517438	0.05	1
	a2009s lh G front inf-Opercular area	Wald ratio	0.235910563	0.05	0.999945377
	IDP dMRI TBSS MD External capsule R	Wald ratio	0.962479881	0.05	1
	IDP dMRI TBSS MD External capsule L	Inverse variance weighted	0.535958714	0.05	1
	a2009s lh G pariet inf-Supramar area	Wald ratio	0.040057223	0.05	0.6819242
	a2009s lh G parietal sup area	Wald ratio	0.578944217	0.05	1
	a2009s lh G postcentral area	Wald ratio	0.453694811	0.05	1
	a2009s lh G precentral area	Wald ratio	0.417238574	0.05	1
	a2009s lh G precuneus area	Wald ratio	0.477517438	0.05	1
	IDP dMRI TBSS MD Cingulum cingulate gyrus R	MR Egger	0.494571965	0.05	1
		Weighted median	0.295852728	0.05	1

		Inverse variance weighted	0.205795468	0.05	1
		Simple mode	0.312545174	0.05	0.99999891
		Weighted mode	0.79901004	0.05	1
	IDP dMRI TBSS MD Cingulum cingulate gyrus L	MR Egger	0.4954403	0.05	1
		Weighted median	0.279885833	0.05	1
		Inverse variance weighted	0.20618677	0.05	1
		Simple mode	0.334134363	0.05	0.999999878
		Weighted mode	0.789927819	0.05	1
	a2009s lh S calcarine area	Wald ratio	0.373188369	0.05	1
	a2009s lh S central area	Wald ratio	0.461190612	0.05	1
	a2009s lh S collat transv ant area	Wald ratio	0.034529977	0.05	1
	IDP dMRI TBSS MD Cingulum hippocampus R	Inverse variance weighted	0.48578909	0.05	1
	a2009s lh S intrapariet&P trans area	Wald ratio	0.578944217	0.05	1
	IDP dMRI TBSS MD Cingulum hippocampus L	Inverse variance weighted	0.50276167	0.05	1
	a2009s lh S subparietal area	Wald ratio	0.373188369	0.05	1
	DKTatlas rh cuneus area	Wald ratio	0.373188369	0.05	1
	DKTatlas rh lateraloccipital area	Inverse variance weighted	0.008592915	0.05	1
	DKTatlas rh lingual area	Wald ratio	0.368272458	0.05	1
	DKTatlas rh parstriangularis area	Wald ratio	0.349025456	0.05	1
	DKTatlas rh pericalcarine area	Inverse variance weighted	0.291750066	0.05	1
	IDP dMRI TBSS MD Superior longitudinal fasciculus R	Inverse variance weighted	0.291568556	0.05	1
	DKTatlas rh postcentral area	Wald ratio	0.461190612	0.05	1

	DKTatlas rh precentral area	Wald ratio	0.417238574	0.05	1
	IDP dMRI TBSS MD Superior longitudinal fasciculus L	Inverse variance weighted	0.360550975	0.05	1
	a2009s rh G&S subcentral area	Wald ratio	0.461190612	0.05	1
	a2009s rh G&S cingul-Mid-Post area	Wald ratio	0.488197071	0.05	1
	a2009s rh G cuneus area	Wald ratio	0.373188369	0.05	1
	IDP dMRI TBSS MD Superior fronto-occipital fasciculus L	Wald ratio	0.215491265	0.05	0.999741702
	a2009s rh G oc-temp med-Lingual area	Wald ratio	0.368272458	0.05	1
	a2009s rh G parietal sup area	Wald ratio	0.325002051	0.05	0.999999882
	IDP dMRI TBSS MD Uncinate fasciculus R	Inverse variance weighted	0.540443076	0.05	1
	a2009s rh G precentral area	Wald ratio	0.417238574	0.05	1
	a2009s rh G precuneus area	Wald ratio	0.96457379	0.05	1
	IDP dMRI TBSS MD Uncinate fasciculus L	Inverse variance weighted	0.586454564	0.05	1
	a2009s rh Pole occipital area	Wald ratio	0.373188369	0.05	1
	a2009s rh S calcarine area	Wald ratio	0.368272458	0.05	1
	a2009s rh S central area	Wald ratio	0.417238574	0.05	1
	a2009s rh S collat transv ant area	Wald ratio	0.034529977	0.05	1
	a2009s rh S orbital med-olfact area	Wald ratio	0.493278433	0.05	1
	IDP dMRI TBSS MO Pontine crossing tract	MR Egger	0.96928315	0.05	1
		Weighted median	0.109061944	0.05	1
		Inverse variance weighted	0.076332659	0.05	1
		Simple mode	0.255859578	0.05	1
		Weighted mode	0.327333004	0.05	1

	DKTatlas lh postcentral thickness	Wald ratio	0.461190612	0.05	1
	a2009s lh G insular short thickness	Wald ratio	0.966370679	0.05	1
	a2009s lh G postcentral thickness	Wald ratio	0.461190612	0.05	1
	IDP T1 SIENAX CSF normalised volume	Inverse variance weighted	0.861359302	0.05	1
	IDP dMRI TBSS MO Medial lemniscus R	Wald ratio	0.41649938	0.05	1
	a2009s lh S postcentral thickness	Wald ratio	0.477343601	0.05	1
	DKTatlas rh lateraloccipital thickness	Wald ratio	0.653778307	0.05	1
	DKTatlas rh parstriangularis thickness	Wald ratio	0.231270056	0.05	1
	DKTatlas rh postcentral thickness	Wald ratio	0.461190612	0.05	1
	DKTatlas rh posteriorcingulate thickness	Inverse variance weighted	0.296664436	0.05	1
	a2009s rh G&S cingul-Mid-Ant thickness	Wald ratio	0.409605252	0.05	1
	a2009s rh G cuneus thickness	Wald ratio	0.653778307	0.05	1
	a2009s rh G postcentral thickness	Wald ratio	0.510134674	0.05	1
	a2009s rh Pole occipital thickness	Wald ratio	0.653778307	0.05	1
	a2009s rh S circular insula ant thickness	Wald ratio	0.14647246	0.05	1
	a2009s rh S oc sup&transversal thickness	Wald ratio	0.729438922	0.05	1
	a2009s rh S parieto occipital thickness	Wald ratio	0.525138264	0.05	1
	a2009s rh S temporal transverse thickness	Wald ratio	0.483763924	0.05	1
	IDP dMRI TBSS MO Anterior corona radiata R	Wald ratio	0.474467317	0.05	1
	IDP dMRI TBSS MO Superior corona radiata R	Wald ratio	0.074252242	0.05	1
	IDP dMRI TBSS MO Cingulum cingulate gyrus L	Inverse variance weighted	0.529224092	0.05	1
	IDP dMRI TBSS L1 Genu of corpus callosum	Wald ratio	0.988397064	0.05	1

	IDP dMRI TBSS L1 Anterior limb of internal capsule L	Wald ratio	0.378551231	0.05	0.999999999
	IDP dMRI TBSS L1 Posterior limb of internal capsule L	Wald ratio	0.871307951	0.05	1
	IDP dMRI TBSS L1 Anterior corona radiata R	Wald ratio	0.962479881	0.05	1
	IDP dMRI TBSS L1 Anterior corona radiata L	Wald ratio	0.962479881	0.05	1
	IDP dMRI TBSS L1 Posterior corona radiata R	Wald ratio	0.525896059	0.05	1
	IDP dMRI TBSS L1 Posterior corona radiata L	Wald ratio	0.962479881	0.05	1
	IDP dMRI TBSS L1 Sagittal stratum L	Wald ratio	0.962479881	0.05	1
	IDP dMRI TBSS L1 External capsule R	Wald ratio	0.962479881	0.05	1
	IDP dMRI TBSS L1 External capsule L	MR Egger	0.783966323	0.05	1
		Weighted median	0.379755822	0.05	1
		Inverse variance weighted	0.238897007	0.05	0.999993626
		Simple mode	0.73746118	0.05	1
		Weighted mode	0.738167309	0.05	1
	IDP dMRI TBSS L1 Cingulum hippocampus L	Wald ratio	0.988397064	0.05	1
	IDP dMRI TBSS L1 Fornix cres+Stria terminalis L	Wald ratio	0.931242537	0.05	1
	IDP T1 FAST ROIs L precentral gyrus	Wald ratio	0.461190612	0.05	1
	IDP dMRI TBSS L1 Superior longitudinal fasciculus R	Inverse variance weighted	0.032165286	0.05	0.710365799
	IDP dMRI TBSS L1 Uncinate fasciculus R	Wald ratio	0.905356301	0.05	1
	IDP dMRI TBSS L1 Uncinate fasciculus L	Wald ratio	0.974147209	0.05	1
	IDP dMRI TBSS L2 Pontine crossing tract	Wald ratio	0.41649938	0.05	1
		MR Egger	0.784077587	0.05	0.999999124

	IDP dMRI TBSS L2 Genu of corpus callosum	Weighted median	0.992308282	0.05	1
		Inverse variance weighted	0.676392434	0.05	1
		Simple mode	0.955298096	0.05	1
		Weighted mode	0.959085477	0.05	1
	IDP dMRI TBSS L2 Body of corpus callosum	Wald ratio	0.962479881	0.05	1
	IDP dMRI TBSS L2 Splenium of corpus callosum	MR Egger	0.631076883	0.05	1
		Weighted median	0.571500664	0.05	1
		Inverse variance weighted	0.238670957	0.05	1
		Simple mode	0.857452248	0.05	1
		Weighted mode	0.87373828	0.05	1
	IDP dMRI TBSS L2 Corticospinal tract R	Wald ratio	0.082484342	0.05	1
	IDP T1 SIENAX CSF unnormalised volume	MR Egger	0.34968146	0.05	1
		Weighted median	0.104343919	0.05	0.997018142
		Inverse variance weighted	0.515286903	0.05	1
		Simple mode	0.331042715	0.05	0.995805653
		Weighted mode	0.331738991	0.05	0.995805653
	IDP T1 FAST ROIs L temporal pole	Wald ratio	0.311052938	0.05	0.999988327
	IDP dMRI TBSS L2 Superior cerebellar peduncle R	Inverse variance weighted	0.759765031	0.05	1
	IDP dMRI TBSS L2 Superior cerebellar peduncle L	Wald ratio	0.554387065	0.05	1
	IDP dMRI TBSS L2 Posterior limb of internal capsule R	Inverse variance weighted	0.786572158	0.05	1
	IDP dMRI TBSS L2 Posterior limb of internal capsule L	Wald ratio	0.995423977	0.05	1

	IDP dMRI TBSS L2 Retrolenticular part of internal capsule R	Inverse variance weighted	0.280318651	0.05	1
	IDP dMRI TBSS L2 Retrolenticular part of internal capsule L	Wald ratio	0.905356301	0.05	1
	IDP dMRI TBSS L2 Anterior corona radiata R	Inverse variance weighted	0.390961702	0.05	1
	IDP dMRI TBSS L2 Anterior corona radiata L	Wald ratio	0.962479881	0.05	1
	IDP dMRI TBSS L2 Superior corona radiata R	MR Egger	0.813442463	0.05	0.997660435
		Weighted median	0.885489566	0.05	1
		Inverse variance weighted	0.475625969	0.05	1
		Simple mode	0.852148522	0.05	1
		Weighted mode	0.873373091	0.05	1
	IDP dMRI TBSS L2 Superior corona radiata L	Inverse variance weighted	0.335640711	0.05	1
	IDP dMRI TBSS L2 Posterior corona radiata R	Inverse variance weighted	0.268140869	0.05	1
	IDP dMRI TBSS L2 Posterior corona radiata L	Inverse variance weighted	0.288360049	0.05	1
	IDP dMRI TBSS L2 Posterior thalamic radiation R	Inverse variance weighted	0.310116735	0.05	1
	IDP dMRI TBSS L2 Posterior thalamic radiation L	Inverse variance weighted	0.315007767	0.05	1
	IDP dMRI TBSS L2 Sagittal stratum R	Inverse variance weighted	0.304737741	0.05	1
	IDP dMRI TBSS L2 Sagittal stratum L	Wald ratio	0.988397064	0.05	1
	IDP dMRI TBSS L2 Cingulum cingulate gyrus R	Wald ratio	0.962479881	0.05	1
	IDP dMRI TBSS L2 Cingulum cingulate gyrus L	Wald ratio	0.988397064	0.05	1
	IDP dMRI TBSS L2 Cingulum hippocampus R	Inverse variance weighted	0.444554914	0.05	1

	IDP dMRI TBSS L2 Cingulum hippocampus L	Wald ratio	0.962479881	0.05	1
	IDP dMRI TBSS L2 Uncinate fasciculus L	Wald ratio	0.962479881	0.05	1
	IDP dMRI TBSS L3 Middle cerebellar peduncle	Wald ratio	0.905356301	0.05	1
	IDP dMRI TBSS L3 Genu of corpus callosum	Wald ratio	0.962479881	0.05	1
	IDP dMRI TBSS L3 Body of corpus callosum	Wald ratio	0.962479881	0.05	1
	IDP dMRI TBSS L3 Splenium of corpus callosum	Inverse variance weighted	0.905714088	0.05	1
	IDP dMRI TBSS L3 Inferior cerebellar peduncle R	Wald ratio	0.962479881	0.05	1
	IDP dMRI TBSS L3 Inferior cerebellar peduncle L	Wald ratio	0.962479881	0.05	1
	IDP dMRI TBSS L3 Superior cerebellar peduncle R	Inverse variance weighted	0.782439906	0.05	1
	IDP dMRI TBSS L3 Superior cerebellar peduncle L	Wald ratio	0.554387065	0.05	1
	IDP dMRI TBSS L3 Cerebral peduncle R	Wald ratio	0.504091713	0.05	1
	IDP dMRI TBSS L3 Anterior limb of internal capsule R	Wald ratio	0.005712511	0.05	0.127916596
	IDP dMRI TBSS L3 Anterior limb of internal capsule L	Wald ratio	0.00481336	0.05	0.083366728
	IDP dMRI TBSS L3 Retrolenticular part of internal capsule R	Inverse variance weighted	0.318577072	0.05	1
	IDP dMRI TBSS L3 Retrolenticular part of internal capsule L	Wald ratio	0.905356301	0.05	1
	IDP dMRI TBSS L3 Anterior corona radiata R	Inverse variance weighted	0.388845816	0.05	1
	IDP dMRI TBSS L3 Anterior corona radiata L	Inverse variance weighted	0.437386518	0.05	1
	IDP dMRI TBSS L3 Superior corona radiata R	Wald ratio	0.988397064	0.05	1
	IDP dMRI TBSS L3 Superior corona radiata L	Wald ratio	0.988397064	0.05	1

	IDP dMRI TBSS L3 Posterior corona radiata R	Wald ratio	0.962479881	0.05	1
	IDP dMRI TBSS L3 Posterior corona radiata L	Wald ratio	0.905356301	0.05	1
	IDP dMRI TBSS L3 Posterior thalamic radiation R	Inverse variance weighted	0.374629432	0.05	1
	IDP dMRI TBSS L3 Posterior thalamic radiation L	Inverse variance weighted	0.431818906	0.05	1
	IDP dMRI TBSS L3 Sagittal stratum R	Inverse variance weighted	0.428124309	0.05	1
	IDP dMRI TBSS L3 Sagittal stratum L	Inverse variance weighted	0.515839121	0.05	1
	IDP dMRI TBSS L3 External capsule R	Wald ratio	0.962479881	0.05	1
	IDP dMRI TBSS L3 External capsule L	Wald ratio	0.962479881	0.05	1
	IDP dMRI TBSS L3 Cingulum cingulate gyrus R	Inverse variance weighted	0.421971612	0.05	1
	IDP dMRI TBSS L3 Cingulum cingulate gyrus L	Wald ratio	0.962479881	0.05	1
	IDP dMRI TBSS L3 Cingulum hippocampus R	Wald ratio	0.962479881	0.05	1
	IDP dMRI TBSS L3 Cingulum hippocampus L	Wald ratio	0.962479881	0.05	1
	IDP dMRI TBSS L3 Fornix cres+Stria terminalis R	Wald ratio	0.619299735	0.05	1
	IDP dMRI TBSS L3 Superior longitudinal fasciculus R	Inverse variance weighted	0.306268871	0.05	1
	IDP dMRI TBSS L3 Superior longitudinal fasciculus L	Inverse variance weighted	0.377735713	0.05	1
	IDP dMRI TBSS L3 Uncinate fasciculus R	Wald ratio	0.962479881	0.05	1
	IDP dMRI TBSS L3 Uncinate fasciculus L	Inverse variance weighted	0.501853852	0.05	1
	IDP dMRI TBSS ICVF Middle cerebellar peduncle	Inverse variance weighted	0.428098273	0.05	1
		MR Egger	0.909108735	0.05	1

	IDP dMRI TBSS ICVF Genu of corpus callosum	Weighted median	0.943840075	0.05	1
		Inverse variance weighted	0.950477192	0.05	1
		Simple mode	0.589621856	0.05	1
		Weighted mode	0.820775975	0.05	1
	IDP dMRI TBSS ICVF Body of corpus callosum	MR Egger	0.731459479	0.05	1
		Weighted median	0.870242854	0.05	1
		Inverse variance weighted	0.462311461	0.05	1
		Simple mode	0.796576262	0.05	1
		Weighted mode	0.852020443	0.05	1
	IDP dMRI TBSS ICVF Splenium of corpus callosum	MR Egger	0.751070094	0.05	1
		Weighted median	0.881323498	0.05	1
		Inverse variance weighted	0.955202301	0.05	1
		Simple mode	0.870354085	0.05	1
		Weighted mode	0.907520852	0.05	1
	IDP dMRI TBSS ICVF Fornix	Wald ratio	0.215491265	0.05	1
	IDP dMRI TBSS ICVF Medial lemniscus R	Inverse variance weighted	0.454946149	0.05	1
	IDP dMRI TBSS ICVF Medial lemniscus L	Wald ratio	0.432882285	0.05	1
	IDP dMRI TBSS ICVF Inferior cerebellar peduncle R	MR Egger	0.54107613	0.05	0.999983534
		Weighted median	0.662819	0.05	1
		Inverse variance weighted	0.337153738	0.05	1
		Simple mode	0.787283168	0.05	1
		Weighted mode	0.825910843	0.05	1

	IDP dMRI TBSS ICVF Inferior cerebellar peduncle L	MR Egger	0.556448362	0.05	0.999127852
		Weighted median	0.658165548	0.05	1
		Inverse variance weighted	0.316132568	0.05	1
		Simple mode	0.782661841	0.05	1
		Weighted mode	0.817727725	0.05	1
	IDP dMRI TBSS ICVF Superior cerebellar peduncle R	MR Egger	0.630786205	0.05	1
		Weighted median	0.815231461	0.05	1
		Inverse variance weighted	0.902751732	0.05	1
		Simple mode	0.849637859	0.05	1
		Weighted mode	0.795694249	0.05	1
	IDP dMRI TBSS ICVF Superior cerebellar peduncle L	Inverse variance weighted	0.67329813	0.05	1
	IDP dMRI TBSS ICVF Cerebral peduncle R	MR Egger	0.659385644	0.05	1
		Weighted median	0.797829336	0.05	1
		Inverse variance weighted	0.861384264	0.05	1
		Simple mode	0.668665723	0.05	1
		Weighted mode	0.768386654	0.05	1
	IDP dMRI TBSS ICVF Cerebral peduncle L	Inverse variance weighted	0.905545861	0.05	1
	IDP dMRI TBSS ICVF Anterior limb of internal capsule R	Wald ratio	0.962479881	0.05	1
	IDP dMRI TBSS ICVF Anterior limb of internal capsule L	MR Egger	0.62081516	0.05	1
		Weighted median	0.897215697	0.05	1
		Inverse variance weighted	0.874820556	0.05	1
		Simple mode	0.873107138	0.05	1

		Weighted mode	0.904860855	0.05	1
	IDP dMRI TBSS ICVF Posterior limb of internal capsule R	MR Egger	0.942659723	0.05	1
		Weighted median	0.836524095	0.05	1
		Inverse variance weighted	0.657466399	0.05	1
		Simple mode	0.958116095	0.05	1
		Weighted mode	0.936042597	0.05	1
	IDP dMRI TBSS ICVF Posterior limb of internal capsule L	MR Egger	0.978547037	0.05	1
		Weighted median	0.968932035	0.05	1
		Inverse variance weighted	0.760641687	0.05	1
		Simple mode	0.790587139	0.05	1
		Weighted mode	0.924916022	0.05	1
	IDP dMRI TBSS ICVF Retrolenticular part of internal capsule R	MR Egger	0.693288369	0.05	0.999998702
		Weighted median	0.470182387	0.05	1
		Inverse variance weighted	0.334870677	0.05	1
		Simple mode	0.378067035	0.05	1
		Weighted mode	0.707693254	0.05	1
	IDP dMRI TBSS ICVF Retrolenticular part of internal capsule L	MR Egger	0.939600319	0.05	1
		Weighted median	0.798172943	0.05	1
		Inverse variance weighted	0.719189812	0.05	1
		Simple mode	0.790131559	0.05	1
		Weighted mode	0.964579686	0.05	1
		MR Egger	0.922119984	0.05	1

	IDP dMRI TBSS ICVF Anterior corona radiata R	Weighted median	0.806812583	0.05	1
		Inverse variance weighted	0.710743828	0.05	1
		Simple mode	0.78619717	0.05	1
		Weighted mode	0.98517392	0.05	1
	IDP dMRI TBSS ICVF Anterior corona radiata L	MR Egger	0.970797867	0.05	1
		Weighted median	0.867639251	0.05	1
		Inverse variance weighted	0.737518391	0.05	1
		Simple mode	0.78102525	0.05	1
		Weighted mode	0.980562509	0.05	1
	IDP dMRI TBSS ICVF Superior corona radiata R	MR Egger	0.865834404	0.05	1
		Weighted median	0.740162012	0.05	1
		Inverse variance weighted	0.683185363	0.05	1
		Simple mode	0.818712044	0.05	1
		Weighted mode	0.928407304	0.05	1
	IDP dMRI TBSS ICVF Superior corona radiata L	MR Egger	0.901572575	0.05	1
		Weighted median	0.782927249	0.05	1
		Inverse variance weighted	0.700195542	0.05	1
		Simple mode	0.805941198	0.05	1
		Weighted mode	0.965841042	0.05	1
	IDP dMRI TBSS ICVF Posterior corona radiata R	MR Egger	0.91603491	0.05	1
		Weighted median	0.967192478	0.05	1
		Inverse variance weighted	0.557357254	0.05	1

		Simple mode	0.837238817	0.05	1
		Weighted mode	0.901870956	0.05	1
	IDP dMRI TBSS ICVF Posterior corona radiata L	MR Egger	0.969113253	0.05	1
		Weighted median	0.892753802	0.05	1
		Inverse variance weighted	0.5402753	0.05	1
		Simple mode	0.82011858	0.05	1
		Weighted mode	0.92967872	0.05	1
	IDP dMRI TBSS ICVF Posterior thalamic radiation R	Inverse variance weighted	0.429060876	0.05	1
	IDP dMRI TBSS ICVF Posterior thalamic radiation L	MR Egger	0.93873411	0.05	1
		Weighted median	0.797476369	0.05	1
		Inverse variance weighted	0.331858698	0.05	1
		Simple mode	0.884111154	0.05	1
		Weighted mode	0.885144098	0.05	1
	IDP dMRI TBSS ICVF Sagittal stratum R	MR Egger	0.778020776	0.05	1
		Weighted median	0.791115148	0.05	1
		Inverse variance weighted	0.332174972	0.05	1
		Simple mode	0.841169087	0.05	1
		Weighted mode	0.915532727	0.05	1
	IDP dMRI TBSS ICVF Sagittal stratum L	MR Egger	0.759343053	0.05	1
		Weighted median	0.643853506	0.05	1
		Inverse variance weighted	0.357580394	0.05	1
		Simple mode	0.736910458	0.05	1

		Weighted mode	0.895348632	0.05	1
	IDP dMRI TBSS ICVF External capsule R	MR Egger	0.534155698	0.05	1
		Weighted median	0.435847796	0.05	1
		Inverse variance weighted	0.325993337	0.05	1
		Simple mode	0.289641413	0.05	1
		Weighted mode	0.840873531	0.05	1
	IDP dMRI TBSS ICVF External capsule L	Inverse variance weighted	0.638366279	0.05	1
	IDP dMRI TBSS ICVF Cingulum cingulate gyrus R	Inverse variance weighted	0.544286069	0.05	1
	IDP dMRI TBSS ICVF Cingulum cingulate gyrus L	Inverse variance weighted	0.565400276	0.05	1
	IDP dMRI TBSS ICVF Cingulum hippocampus R	MR Egger	0.979619539	0.05	1
		Weighted median	0.879690704	0.05	1
		Inverse variance weighted	0.779543199	0.05	1
		Simple mode	0.737919769	0.05	1
		Weighted mode	0.991505405	0.05	1
	IDP dMRI TBSS ICVF Cingulum hippocampus L	MR Egger	0.972645738	0.05	1
		Weighted median	0.829170901	0.05	1
		Inverse variance weighted	0.742063632	0.05	1
		Simple mode	0.768736876	0.05	1
		Weighted mode	0.978824385	0.05	1
	IDP dMRI TBSS ICVF Fornix cres+Stria terminalis R	Inverse variance weighted	0.795199418	0.05	1
	IDP dMRI TBSS ICVF Fornix cres+Stria terminalis L	Wald ratio	0.988397064	0.05	1

	IDP dMRI TBSS ICVF Superior longitudinal fasciculus R	MR Egger	0.800707573	0.05	1
		Weighted median	0.694437761	0.05	1
		Inverse variance weighted	0.66599866	0.05	1
		Simple mode	0.789194594	0.05	1
		Weighted mode	0.910042655	0.05	1
	IDP dMRI TBSS ICVF Superior longitudinal fasciculus L	MR Egger	0.802921469	0.05	1
		Weighted median	0.645290172	0.05	1
		Inverse variance weighted	0.64707186	0.05	1
		Simple mode	0.835473179	0.05	1
		Weighted mode	0.857517113	0.05	1
	IDP dMRI TBSS ICVF Superior fronto-occipital fasciculus R	Inverse variance weighted	0.431799736	0.05	1
	IDP dMRI TBSS ICVF Superior fronto-occipital fasciculus L	Wald ratio	0.988397064	0.05	1
	IDP dMRI TBSS ICVF Uncinate fasciculus R	Wald ratio	0.962479881	0.05	1
	IDP dMRI TBSS ICVF Uncinate fasciculus L	Wald ratio	0.962479881	0.05	1
	IDP dMRI TBSS ICVF Tapetum R	Wald ratio	0.905356301	0.05	1
	IDP dMRI TBSS ICVF Tapetum L	Wald ratio	0.905356301	0.05	1
	IDP dMRI TBSS OD Pontine crossing tract	MR Egger	0.789126016	0.05	1
		Weighted median	0.108385293	0.05	1
		Inverse variance weighted	0.055766688	0.05	1
		Simple mode	0.304325819	0.05	1
		Weighted mode	0.261789531	0.05	1
	IDP dMRI TBSS OD Posterior limb of internal capsule L	Wald ratio	0.871307951	0.05	1

	IDP dMRI TBSS OD Posterior corona radiata R	Wald ratio	0.003098043	0.05	1
	IDP dMRI TBSS OD External capsule R	Wald ratio	0.801164528	0.05	1
	IDP dMRI TBSS OD Superior longitudinal fasciculus R	Wald ratio	0.00070816	0.05	1
	IDP dMRI TBSS ISOVF Fornix	Wald ratio	0.56518584	0.05	1
	IDP dMRI TBSS ISOVF External capsule R	Wald ratio	0.342623203	0.05	1
	IDP dMRI TBSS ISOVF Cingulum cingulate gyrus R	Inverse variance weighted	0.866700917	0.05	1
	IDP dMRI ProbtrackX FA ar l	Wald ratio	0.962479881	0.05	1
	IDP dMRI ProbtrackX FA atr l	Wald ratio	0.962479881	0.05	1
	IDP dMRI ProbtrackX FA atr r	Wald ratio	0.905356301	0.05	1
	IDP dMRI ProbtrackX FA fma	Wald ratio	0.905356301	0.05	1
	IDP dMRI ProbtrackX FA fmi	MR Egger	0.988085688	0.05	1
		Weighted median	0.874357866	0.05	1
		Inverse variance weighted	0.656840361	0.05	1
		Simple mode	0.984361265	0.05	1
		Weighted mode	0.981826859	0.05	1
	IDP dMRI ProbtrackX FA ifo l	Inverse variance weighted	0.436100407	0.05	1
	IDP dMRI ProbtrackX FA ifo r	Inverse variance weighted	0.376763037	0.05	1
	IDP dMRI ProbtrackX FA ilf l	Inverse variance weighted	0.4278827	0.05	1
	IDP dMRI ProbtrackX FA ilf r	Inverse variance weighted	0.420832497	0.05	1
	IDP dMRI ProbtrackX FA ml l	Wald ratio	0.970232419	0.05	1
	IDP dMRI ProbtrackX FA ptr l	Wald ratio	0.988397064	0.05	1

	IDP dMRI ProbtrackX FA ptr r	Wald ratio	0.938919777	0.05	1
	IDP dMRI ProbtrackX FA slf l	Inverse variance weighted	0.357565893	0.05	1
	IDP dMRI ProbtrackX FA slf r	Wald ratio	0.905356301	0.05	1
	IDP dMRI ProbtrackX FA str l	Wald ratio	0.372653245	0.05	1
	IDP dMRI ProbtrackX FA unc l	Wald ratio	0.962479881	0.05	1
	IDP dMRI ProbtrackX FA unc r	Wald ratio	0.962479881	0.05	1
	IDP dMRI ProbtrackX MD ar l	Wald ratio	0.962479881	0.05	1
	IDP dMRI ProbtrackX MD ar r	Wald ratio	0.962479881	0.05	1
	IDP dMRI ProbtrackX MD atr l	Wald ratio	0.962479881	0.05	1
	IDP dMRI ProbtrackX MD atr r	Wald ratio	0.962479881	0.05	1
	IDP dMRI ProbtrackX MD cgc l	MR Egger	0.504668704	0.05	1
		Weighted median	0.350049104	0.05	1
		Inverse variance weighted	0.224977753	0.05	1
		Simple mode	0.378170287	0.05	1
		Weighted mode	0.728969528	0.05	1
	IDP dMRI ProbtrackX MD cgc r	MR Egger	0.486353099	0.05	1
		Weighted median	0.264361464	0.05	1
		Inverse variance weighted	0.219692258	0.05	1
		Simple mode	0.322192693	0.05	0.999999729
		Weighted mode	0.817038493	0.05	1
	IDP dMRI ProbtrackX MD fmi	Inverse variance weighted	0.423816689	0.05	1
	IDP dMRI ProbtrackX MD ifo l	Inverse variance weighted	0.441787897	0.05	1

	IDP dMRI ProbtrackX MD ifo r	Inverse variance weighted	0.422664858	0.05	1
	IDP dMRI ProbtrackX MD ilf l	Inverse variance weighted	0.437352712	0.05	1
	IDP dMRI ProbtrackX MD ilf r	Inverse variance weighted	0.482065653	0.05	1
	IDP dMRI ProbtrackX MD ptr l	Wald ratio	0.962479881	0.05	1
	IDP dMRI ProbtrackX MD ptr r	Wald ratio	0.962479881	0.05	1
	IDP dMRI ProbtrackX MD slf l	Inverse variance weighted	0.396675371	0.05	1
	IDP dMRI ProbtrackX MD slf r	Inverse variance weighted	0.394360202	0.05	1
	IDP dMRI ProbtrackX MD str l	Wald ratio	0.731706859	0.05	1
	IDP dMRI ProbtrackX MD str r	Wald ratio	0.962479881	0.05	1
	IDP dMRI ProbtrackX MD unc l	Wald ratio	0.962479881	0.05	1
	IDP dMRI ProbtrackX MD unc r	Inverse variance weighted	0.967796077	0.05	1
	IDP dMRI ProbtrackX MO atr l	Wald ratio	0.462244187	0.05	1
	IDP dMRI ProbtrackX MO ml r	Wald ratio	0.037351171	0.05	1
	IDP T1 FAST ROIs L intracalc cortex	Wald ratio	0.373188369	0.05	1
	IDP dMRI ProbtrackX L1 fmi	Wald ratio	0.988397064	0.05	1
	IDP dMRI ProbtrackX L1 ifo l	Wald ratio	0.988397064	0.05	1
	IDP dMRI ProbtrackX L1 ifo r	Wald ratio	0.988397064	0.05	1
	IDP dMRI ProbtrackX L1 ilf l	Wald ratio	0.962479881	0.05	1
	IDP dMRI ProbtrackX L1 ilf r	Wald ratio	0.988397064	0.05	1
	IDP T1 FAST ROIs R intracalc cortex	Wald ratio	0.373188369	0.05	1
	IDP dMRI ProbtrackX L1 slf l	Wald ratio	0.962479881	0.05	1
	IDP dMRI ProbtrackX L1 slf r	Inverse variance weighted	0.352545587	0.05	0.887518525

	IDP dMRI ProbtrackX L1 str r	Wald ratio	0.171504278	0.05	1
	IDP dMRI ProbtrackX L1 unc l	Inverse variance weighted	0.550329802	0.05	1
	IDP dMRI ProbtrackX L1 unc r	Inverse variance weighted	0.948554146	0.05	1
	IDP dMRI ProbtrackX L2 ar l	Wald ratio	0.962479881	0.05	1
	IDP dMRI ProbtrackX L2 ar r	Wald ratio	0.962479881	0.05	1
	IDP dMRI ProbtrackX L2 atr l	Wald ratio	0.962479881	0.05	1
	IDP dMRI ProbtrackX L2 atr r	Wald ratio	0.962479881	0.05	1
	IDP dMRI ProbtrackX L2 cgc l	Wald ratio	0.988397064	0.05	1
	IDP dMRI ProbtrackX L2 cgc r	Wald ratio	0.988397064	0.05	1
	IDP dMRI ProbtrackX L2 cgh l	Wald ratio	0.164927075	0.05	0.999898421
	IDP dMRI ProbtrackX L2 fma	Wald ratio	0.42522665	0.05	1
	IDP dMRI ProbtrackX L2 fmi	Inverse variance weighted	0.421406138	0.05	1
	IDP dMRI ProbtrackX L2 ifo l	Inverse variance weighted	0.425428367	0.05	1
	IDP dMRI ProbtrackX L2 ifo r	Inverse variance weighted	0.395301805	0.05	1
	IDP dMRI ProbtrackX L2 ilf l	Inverse variance weighted	0.441671325	0.05	1
	IDP dMRI ProbtrackX L2 ilf r	Inverse variance weighted	0.469531983	0.05	1
	IDP dMRI ProbtrackX L2 ptr l	Wald ratio	0.962479881	0.05	1
	IDP dMRI ProbtrackX L2 ptr r	Wald ratio	0.998188581	0.05	1
	IDP dMRI ProbtrackX L2 slf l	Inverse variance weighted	0.389709258	0.05	1
	IDP dMRI ProbtrackX L2 slf r	Inverse variance weighted	0.363255677	0.05	1
	IDP dMRI ProbtrackX L2 str l	Wald ratio	0.731706859	0.05	1

	IDP dMRI ProbtrackX L2 str r	Wald ratio	0.731706859	0.05	1
	IDP dMRI ProbtrackX L2 unc l	Wald ratio	0.962479881	0.05	1
	IDP dMRI ProbtrackX L2 unc r	Inverse variance weighted	0.970113836	0.05	1
	IDP dMRI ProbtrackX L3 ar l	Wald ratio	0.962479881	0.05	1
	IDP dMRI ProbtrackX L3 ar r	Wald ratio	0.962479881	0.05	1
	IDP dMRI ProbtrackX L3 atr l	Wald ratio	0.962479881	0.05	1
	IDP dMRI ProbtrackX L3 atr r	Wald ratio	0.962479881	0.05	1
	IDP dMRI ProbtrackX L3 cgc l	Wald ratio	0.905356301	0.05	1
	IDP dMRI ProbtrackX L3 cgc r	Wald ratio	0.905356301	0.05	1
	IDP dMRI ProbtrackX L3 fmi	MR Egger	0.899726903	0.05	1
		Weighted median	0.82807854	0.05	1
		Inverse variance weighted	0.70462443	0.05	1
		Simple mode	0.819756564	0.05	1
		Weighted mode	0.999654919	0.05	1
	IDP dMRI ProbtrackX L3 ifo l	Inverse variance weighted	0.465998247	0.05	1
	IDP dMRI ProbtrackX L3 ifo r	Inverse variance weighted	0.455424048	0.05	1
	IDP dMRI ProbtrackX L3 ilf l	Inverse variance weighted	0.474258523	0.05	1
	IDP dMRI ProbtrackX L3 ilf r	Inverse variance weighted	0.508599505	0.05	1
	IDP dMRI ProbtrackX L3 ptr l	Wald ratio	0.962479881	0.05	1
	IDP dMRI ProbtrackX L3 ptr r	Wald ratio	0.998188581	0.05	1
	IDP dMRI ProbtrackX L3 slf l	Inverse variance weighted	0.409847164	0.05	1

	IDP dMRI ProbtrackX L3 slf r	Inverse variance weighted	0.415727786	0.05	1
	IDP dMRI ProbtrackX L3 str l	Wald ratio	0.998188581	0.05	1
	IDP dMRI ProbtrackX L3 str r	Wald ratio	0.988397064	0.05	1
	IDP dMRI ProbtrackX L3 unc l	Wald ratio	0.962479881	0.05	1
	IDP dMRI ProbtrackX L3 unc r	Inverse variance weighted	0.963949569	0.05	1
	IDP dMRI ProbtrackX ICVF ar l	MR Egger	0.921710274	0.05	1
		Weighted median	0.952718322	0.05	1
		Inverse variance weighted	0.802579196	0.05	1
		Simple mode	0.740299413	0.05	1
		Weighted mode	0.963769674	0.05	1
	IDP dMRI ProbtrackX ICVF ar r	MR Egger	0.953267856	0.05	1
		Weighted median	0.890795892	0.05	1
		Inverse variance weighted	0.777049206	0.05	1
		Simple mode	0.757678001	0.05	1
		Weighted mode	0.997116612	0.05	1
	IDP dMRI ProbtrackX ICVF atr l	MR Egger	0.969983497	0.05	1
		Weighted median	0.958813237	0.05	1
		Inverse variance weighted	0.778418472	0.05	1
		Simple mode	0.739011502	0.05	1
		Weighted mode	0.955800616	0.05	1
	IDP dMRI ProbtrackX ICVF atr r	MR Egger	0.958746355	0.05	1
		Weighted median	0.975081841	0.05	1

		Inverse variance weighted	0.781965458	0.05	1
		Simple mode	0.766255059	0.05	1
		Weighted mode	0.935656883	0.05	1
	IDP dMRI ProbtrackX ICVF cgc l	Inverse variance weighted	0.541805841	0.05	1
	IDP dMRI ProbtrackX ICVF cgc r	Wald ratio	0.988397064	0.05	1
	IDP dMRI ProbtrackX ICVF cgh l	MR Egger	0.956350545	0.05	1
		Weighted median	0.973942303	0.05	1
		Inverse variance weighted	0.787576294	0.05	1
		Simple mode	0.74217305	0.05	1
		Weighted mode	0.956997708	0.05	1
	IDP dMRI ProbtrackX ICVF cgh r	Wald ratio	0.962479881	0.05	1
	IDP dMRI ProbtrackX ICVF cst l	MR Egger	0.947694889	0.05	1
		Weighted median	0.90564543	0.05	1
		Inverse variance weighted	0.765807655	0.05	1
		Simple mode	0.793485051	0.05	1
		Weighted mode	0.980517841	0.05	1
	IDP dMRI ProbtrackX ICVF cst r	MR Egger	0.935518098	0.05	1
		Weighted median	0.884602832	0.05	1
		Inverse variance weighted	0.656297795	0.05	1
		Simple mode	0.997619828	0.05	1
		Weighted mode	0.976475756	0.05	1
	IDP dMRI ProbtrackX ICVF fma	MR Egger	0.675810045	0.05	1

		Weighted median	0.892274665	0.05	1
		Inverse variance weighted	0.425618293	0.05	1
		Simple mode	0.933389437	0.05	1
		Weighted mode	0.91723809	0.05	1
	IDP dMRI ProbtrackX ICVF fmi	MR Egger	0.923119037	0.05	1
		Weighted median	0.945133321	0.05	1
		Inverse variance weighted	0.690971472	0.05	1
		Simple mode	0.955832023	0.05	1
		Weighted mode	0.998500631	0.05	1
	IDP dMRI ProbtrackX ICVF ifo l	Inverse variance weighted	0.521032489	0.05	1
	IDP dMRI ProbtrackX ICVF ifo r	Inverse variance weighted	0.516026376	0.05	1
	IDP dMRI ProbtrackX ICVF ilf l	Inverse variance weighted	0.500455847	0.05	1
	IDP dMRI ProbtrackX ICVF ilf r	Inverse variance weighted	0.530496191	0.05	1
	IDP dMRI ProbtrackX ICVF mcp	Inverse variance weighted	0.589873806	0.05	1
	IDP dMRI ProbtrackX ICVF ml l	Wald ratio	0.974147209	0.05	1
	IDP dMRI ProbtrackX ICVF ml r	Wald ratio	0.962479881	0.05	1
	IDP dMRI ProbtrackX ICVF ptr l	Inverse variance weighted	0.545196836	0.05	1
	IDP dMRI ProbtrackX ICVF ptr r	MR Egger	0.543702437	0.05	0.412273018
		Weighted median	0.217483931	0.05	1
		Inverse variance weighted	0.303951287	0.05	1
		Simple mode	0.426491565	0.05	1

		Weighted mode	0.511783702	0.05	1
	IDP dMRI ProbtrackX ICVF slf l	MR Egger	0.897608658	0.05	1
		Weighted median	0.759605278	0.05	1
		Inverse variance weighted	0.696148734	0.05	1
		Simple mode	0.77301007	0.05	1
		Weighted mode	0.952813123	0.05	1
	IDP dMRI ProbtrackX ICVF slf r	MR Egger	0.884520832	0.05	1
		Weighted median	0.759932791	0.05	1
		Inverse variance weighted	0.698749908	0.05	1
		Simple mode	0.779283958	0.05	1
		Weighted mode	0.941636748	0.05	1
	IDP dMRI ProbtrackX ICVF str l	MR Egger	0.999974051	0.05	1
		Weighted median	0.931108078	0.05	1
		Inverse variance weighted	0.751381041	0.05	1
		Simple mode	0.785623336	0.05	1
		Weighted mode	0.952109133	0.05	1
	IDP dMRI ProbtrackX ICVF str r	MR Egger	0.936045697	0.05	1
		Weighted median	0.826386812	0.05	1
		Inverse variance weighted	0.718214366	0.05	1
		Simple mode	0.795931545	0.05	1
		Weighted mode	0.996792387	0.05	1
	IDP dMRI ProbtrackX ICVF unc l	Inverse variance weighted	0.728135331	0.05	1

	IDP dMRI ProbtrackX ICVF unc r	Inverse variance weighted	0.599895355	0.05	1
	IDP dMRI ProbtrackX OD ilf l	Wald ratio	0.742158774	0.05	1
	IDP dMRI ProbtrackX OD slf l	Wald ratio	0.332990636	0.05	0.999999738
	IDP dMRI ProbtrackX OD str l	Inverse variance weighted	0.892421373	0.05	1
	IDP dMRI ProbtrackX ISOVF atr r	Wald ratio	0.962479881	0.05	1
	IDP dMRI ProbtrackX ISOVF ifo r	Inverse variance weighted	0.66416296	0.05	1
	IDP dMRI ProbtrackX ISOVF ilf l	Wald ratio	0.685845716	0.05	1
	IDP dMRI ProbtrackX ISOVF ilf r	MR Egger	0.400169338	0.05	1
		Weighted median	0.625440357	0.05	1
		Inverse variance weighted	0.305209064	0.05	1
		Simple mode	0.85153314	0.05	1
		Weighted mode	0.856310367	0.05	1
	IDP dMRI ProbtrackX ISOVF slf l	Inverse variance weighted	0.718058888	0.05	1
	IDP dMRI ProbtrackX ISOVF slf r	MR Egger	0.570577197	0.05	0.002754711
		Weighted median	0.763413528	0.05	1
		Inverse variance weighted	0.875316397	0.05	1
		Simple mode	0.690841633	0.05	1
		Weighted mode	0.695870739	0.05	1
	IDP dMRI ProbtrackX ISOVF unc l	Wald ratio	0.808575404	0.05	1
	IDP dMRI ProbtrackX ISOVF unc r	Wald ratio	0.894898908	0.05	1
	IDP T1 SIENAX brain-normalised volume	Wald ratio	0.539472178	0.05	1
Systemic connective tissue disorders	IDP T1 SIENAX peripheral grey normalised volume	Wald ratio	0.431477022	0.05	1

	IDP T1 FAST ROIs R temp occ fusif cortex	Wald ratio	0.450266028	0.05	1
	IDP T1 FAST ROIs R occ pole	Inverse variance weighted	0.481710504	0.05	1
	IDP T1 FAST ROIs L thalamus	Inverse variance weighted	0.414657984	0.05	1
	IDP T1 FAST ROIs R thalamus	Wald ratio	0.955695871	0.05	1
	IDP T1 FAST ROIs L putamen	Wald ratio	0.975010113	0.05	1
	IDP T1 FAST ROIs R putamen	Wald ratio	0.975010113	0.05	1
	IDP T1 FAST ROIs L pallidum	Wald ratio	0.517978486	0.05	1
	IDP T1 FIRST left caudate volume	Wald ratio	0.851019906	0.05	1
	IDP T1 FAST ROIs L hippocampus	Inverse variance weighted	0.510619677	0.05	1
	IDP T1 FAST ROIs R hippocampus	Wald ratio	0.6541018	0.05	1
	IDP T1 FAST ROIs L ventral striatum	Wald ratio	0.975010113	0.05	1
	IDP T1 FAST ROIs R ventral striatum	Inverse variance weighted	0.323770271	0.05	1
	IDP T1 FAST ROIs L cerebellum VI	Wald ratio	0.984803118	0.05	1
	IDP T1 FAST ROIs L cerebellum crus I	MR Egger	0.11499933	0.05	1
		Weighted median	0.938816207	0.05	1
		Inverse variance weighted	0.979784813	0.05	1
		Simple mode	0.293922258	0.05	1
		Weighted mode	0.145214075	0.05	1
	IDP T1 FAST ROIs R cerebellum crus I	Inverse variance weighted	0.254756173	0.05	1
	IDP T1 FAST ROIs L cerebellum crus II	MR Egger	0.784709873	0.05	1
		Weighted median	0.419135543	0.05	1
		Inverse variance weighted	0.397469492	0.05	1

		Simple mode	0.428217722	0.05	1
		Weighted mode	0.424368466	0.05	1
	IDP T1 FAST ROIs V cerebellum crus II	Inverse variance weighted	0.652103757	0.05	1
	IDP T1 FAST ROIs R cerebellum crus II	Inverse variance weighted	0.526596735	0.05	1
	IDP T1 FIRST left putamen volume	Inverse variance weighted	0.10612974	0.05	1
	IDP T1 FAST ROIs R cerebellum VIIb	Wald ratio	0.559668927	0.05	1
	IDP T1 FAST ROIs V cerebellum VIIIa	MR Egger	0.701975968	0.05	1
		Weighted median	0.449142898	0.05	1
		Inverse variance weighted	0.369474407	0.05	1
		Simple mode	0.565640576	0.05	1
		Weighted mode	0.580846759	0.05	1
	IDP T1 FAST ROIs L cerebellum VIIIb	Wald ratio	0.831729927	0.05	1
	IDP T1 FAST ROIs V cerebellum VIIIb	Inverse variance weighted	0.523815471	0.05	1
	IDP T1 FAST ROIs R cerebellum VIIIb	Wald ratio	0.975010113	0.05	1
	IDP T1 FAST ROIs L cerebellum IX	Inverse variance weighted	0.861285987	0.05	1
	IDP T1 FIRST right putamen volume	Wald ratio	0.65867188	0.05	1
	IDP T1 FAST ROIs V cerebellum IX	Inverse variance weighted	0.836986354	0.05	1
	IDP T1 FAST ROIs R cerebellum IX	Inverse variance weighted	0.931018531	0.05	1
	IDP T1 FAST ROIs V cerebellum X	Wald ratio	0.975010113	0.05	1
	IDP T2 FLAIR BIANCA WMH volume	Inverse variance weighted	0.640265903	0.05	1
	IDP SWI T2star left caudate	MR Egger	0.583148386	0.05	1

		Weighted median	0.981134452	0.05	1
		Inverse variance weighted	0.383669465	0.05	1
		Simple mode	0.810994065	0.05	1
		Weighted mode	0.917764087	0.05	1
	IDP SWI T2star right caudate	MR Egger	0.552130171	0.05	1
		Weighted median	0.125729482	0.05	1
		Inverse variance weighted	0.090620696	0.05	1
		Simple mode	0.361029591	0.05	1
		Weighted mode	0.260061148	0.05	1
	IDP T1 FIRST left pallidum volume	Wald ratio	0.650738624	0.05	1
	IDP SWI T2star left putamen	MR Egger	0.438536594	0.05	1
		Weighted median	0.012777455	0.05	1
		Inverse variance weighted	0.003674576	0.05	1
		Simple mode	0.062617463	0.05	1
		Weighted mode	0.101962849	0.05	1
	IDP SWI T2star right putamen	MR Egger	0.549636906	0.05	1
		Weighted median	0.198321058	0.05	1
		Inverse variance weighted	0.391633685	0.05	1
		Simple mode	0.230923758	0.05	1
		Weighted mode	0.25016395	0.05	1
	IDP SWI T2star left pallidum	MR Egger	0.542509059	0.05	1
		Weighted median	0.667891914	0.05	1

		Inverse variance weighted	0.888828937	0.05	1
		Simple mode	0.623103595	0.05	1
		Weighted mode	0.667071857	0.05	1
	IDP SWI T2star right pallidum	MR Egger	0.713071737	0.05	1
		Weighted median	0.901630878	0.05	1
		Inverse variance weighted	0.767532141	0.05	1
		Simple mode	0.447552218	0.05	1
		Weighted mode	0.527557055	0.05	1
	IDP T1 FIRST right pallidum volume	Wald ratio	0.650738624	0.05	1
	IDP dMRI TBSS FA Genu of corpus callosum	Wald ratio	0.521356628	0.05	1
	IDP dMRI TBSS FA Splenium of corpus callosum	Inverse variance weighted	0.51480705	0.05	1
	IDP dMRI TBSS FA Corticospinal tract L	Wald ratio	0.408902902	0.05	1
	IDP dMRI TBSS FA Superior cerebellar peduncle R	Inverse variance weighted	0.508082951	0.05	1
	IDP dMRI TBSS FA Superior cerebellar peduncle L	Inverse variance weighted	0.567818499	0.05	1
	IDP dMRI TBSS FA Posterior limb of internal capsule R	Wald ratio	0.544474509	0.05	1
	IDP dMRI TBSS FA Retrolenticular part of internal capsule R	Inverse variance weighted	0.600197262	0.05	1
	IDP dMRI TBSS FA Anterior corona radiata R	Wald ratio	0.521356628	0.05	1
	IDP dMRI TBSS FA Anterior corona radiata L	Wald ratio	0.521356628	0.05	1
	IDP dMRI TBSS FA Posterior thalamic radiation R	Wald ratio	0.493062519	0.05	1
	IDP dMRI TBSS FA Posterior thalamic radiation L	Wald ratio	0.493062519	0.05	1

	IDP dMRI TBSS FA Sagittal stratum R	Wald ratio	0.094427919	0.05	1
	IDP dMRI TBSS FA Cingulum cingulate gyrus R	Inverse variance weighted	0.574826892	0.05	1
	IDP dMRI TBSS FA Superior longitudinal fasciculus L	Inverse variance weighted	0.514642256	0.05	1
	IDP dMRI TBSS FA Uncinate fasciculus L	Wald ratio	0.582481917	0.05	1
	IDP dMRI TBSS MD Genu of corpus callosum	Wald ratio	0.521356628	0.05	1
	IDP dMRI TBSS MD Body of corpus callosum	Wald ratio	0.911815241	0.05	1
	IDP dMRI TBSS MD Splenium of corpus callosum	Inverse variance weighted	0.595984436	0.05	1
	IDP dMRI TBSS MD Corticospinal tract R	Wald ratio	0.168934814	0.05	1
	IDP dMRI TBSS MD Superior cerebellar peduncle R	Wald ratio	0.708008676	0.05	1
	IDP dMRI TBSS MD Superior cerebellar peduncle L	Wald ratio	0.889224378	0.05	1
	IDP dMRI TBSS MD Anterior limb of internal capsule L	Wald ratio	0.486551461	0.05	1
	IDP dMRI TBSS MD Retrolenticular part of internal capsule R	Wald ratio	0.493062519	0.05	1
	IDP T1 FIRST left caudate volume plus IDP T1 FIRST right caudate volume	Wald ratio	0.599661832	0.05	1
	IDP T1 FIRST left putamen volume plus IDP T1 FIRST right putamen volume	Inverse variance weighted	0.178361423	0.05	1
	IDP T1 FIRST left pallidum volume plus IDP T1 FIRST right pallidum volume	Wald ratio	0.650738624	0.05	1
	IDP SWI T2star left thalamus plus IDP SWI T2star right thalamus	MR Egger	0.43733797	0.05	1
		Weighted median	0.049712806	0.05	1
		Inverse variance weighted	0.020453611	0.05	1
		Simple mode	0.182993559	0.05	1
		Weighted mode	0.229584486	0.05	1

	IDP dMRI TBSS MD Retrolenticular part of internal capsule L	Wald ratio	0.493062519	0.05	1
	IDP SWI T2star left caudate plus IDP SWI T2star right caudate	MR Egger	0.363731799	0.05	1
		Weighted median	0.056716961	0.05	1
		Inverse variance weighted	0.022272911	0.05	1
		Simple mode	0.111857196	0.05	1
		Weighted mode	0.131631772	0.05	1
	IDP SWI T2star left putamen plus IDP SWI T2star right putamen	MR Egger	0.576270941	0.05	1
		Weighted median	0.011361517	0.05	1
		Inverse variance weighted	0.001270826	0.05	1
		Simple mode	0.1353695	0.05	1
		Weighted mode	0.118493605	0.05	1
	IDP SWI T2star left pallidum plus IDP SWI T2star right pallidum	MR Egger	0.817117539	0.05	1
		Weighted median	0.444393736	0.05	1
		Inverse variance weighted	0.866538344	0.05	1
		Simple mode	0.514029893	0.05	1
		Weighted mode	0.447953815	0.05	1
	volume Left-Lateral-Ventricle	MR Egger	0.720256633	0.05	0.999999999
		Weighted median	0.377708543	0.05	1
		Inverse variance weighted	0.464554323	0.05	1
		Simple mode	0.301594131	0.05	1
		Weighted mode	0.341135402	0.05	1
	volume Left-Cerebellum-White-Matter	Inverse variance weighted	0.177600862	0.05	1

	volume Left-Cerebellum-Cortex	Inverse variance weighted	0.189129519	0.05	1
	IDP dMRI TBSS MD Anterior corona radiata R	Inverse variance weighted	0.354678362	0.05	1
	volume Left-Putamen	Inverse variance weighted	0.484006629	0.05	1
	volume 4th-Ventricle	Inverse variance weighted	0.261613795	0.05	1
	volume Brain-Stem	Wald ratio	0.364722088	0.05	1
	volume Left-Hippocampus	Inverse variance weighted	0.178193336	0.05	1
	volume CSF	MR Egger	0.870311529	0.05	1
		Weighted median	0.873677222	0.05	1
		Inverse variance weighted	0.454695239	0.05	1
		Simple mode	0.843373126	0.05	1
		Weighted mode	0.845585963	0.05	1
	IDP dMRI TBSS MD Anterior corona radiata L	Inverse variance weighted	0.358767722	0.05	1
	volume Left-Accumbens-area	Wald ratio	0.975010113	0.05	1
	volume Right-Lateral-Ventricle	Inverse variance weighted	0.080429496	0.05	1
	volume Right-Inf-Lat-Vent	Wald ratio	0.309038616	0.05	1
	volume Right-Cerebellum-White-Matter	Wald ratio	0.722829483	0.05	1
	volume Right-Cerebellum-Cortex	MR Egger	0.572681505	0.05	1
		Weighted median	0.033026459	0.05	1
		Inverse variance weighted	0.064980532	0.05	1
		Simple mode	0.208808848	0.05	1
		Weighted mode	0.180697885	0.05	1

	IDP dMRI TBSS MD Superior corona radiata R	Inverse variance weighted	0.354749956	0.05	1
	volume Right-Putamen	Wald ratio	0.65867188	0.05	1
	volume Right-Hippocampus	Wald ratio	0.6541018	0.05	1
	volume Right-Amygdala	Wald ratio	0.248954485	0.05	1
	volume Right-VentralDC	Wald ratio	0.104534731	0.05	1
	volume CC Posterior	Wald ratio	0.277921655	0.05	1
	IDP dMRI TBSS MD Superior corona radiata L	Inverse variance weighted	0.35514093	0.05	1
	IDP dMRI TBSS MD Posterior corona radiata R	Wald ratio	0.493062519	0.05	1
	volume BrainSegVol-to-eTIV	Wald ratio	0.710504184	0.05	1
	IDP dMRI TBSS MD Posterior corona radiata L	Wald ratio	0.493062519	0.05	1
	volume rhSurfaceHoles	Wald ratio	0.433479595	0.05	1
	DKTatlas lh cuneus area	Wald ratio	0.646821821	0.05	1
	IDP dMRI TBSS MD Posterior thalamic radiation R	Wald ratio	0.493062519	0.05	1
	DKTatlas lh lateraloccipital area	Wald ratio	0.646821821	0.05	1
	DKTatlas lh lingual area	Wald ratio	0.670903433	0.05	1
	IDP dMRI TBSS MD Posterior thalamic radiation L	Wald ratio	0.493062519	0.05	1
	DKTatlas lh parstriangularis area	Wald ratio	0.943326089	0.05	1
	DKTatlas lh pericalcarine area	Inverse variance weighted	0.431517423	0.05	1
	DKTatlas lh postcentral area	Inverse variance weighted	0.210612278	0.05	1
	DKTatlas lh posteriorcingulate area	Wald ratio	0.115256445	0.05	1
	DKTatlas lh precentral area	Wald ratio	0.148159213	0.05	1

	DKTatlas lh precuneus area	Wald ratio	0.535443259	0.05	1
	IDP dMRI TBSS MD Sagittal stratum R	Wald ratio	0.493062519	0.05	1
	DKTatlas lh superiorparietal area	Wald ratio	0.061891885	0.05	1
	DKTatlas lh superiortemporal area	Wald ratio	0.361779014	0.05	1
	DKTatlas lh supramarginal area	Wald ratio	0.160364667	0.05	1
	DKTatlas lh WhiteSurfArea area	Wald ratio	0.268232169	0.05	1
	IDP dMRI TBSS MD Sagittal stratum L	Wald ratio	0.493062519	0.05	1
	a2009s lh G&S subcentral area	Wald ratio	0.148159213	0.05	1
	a2009s lh G cuneus area	Wald ratio	0.716129569	0.05	1
	a2009s lh G front inf-Opercular area	Wald ratio	0.918185967	0.05	1
	IDP dMRI TBSS MD External capsule R	Wald ratio	0.521356628	0.05	1
	IDP dMRI TBSS MD External capsule L	Inverse variance weighted	0.945708851	0.05	1
	a2009s lh G pariet inf-Supramar area	Wald ratio	0.160364667	0.05	1
	a2009s lh G parietal sup area	Wald ratio	0.061471233	0.05	1
	a2009s lh G postcentral area	Wald ratio	0.061471233	0.05	1
	a2009s lh G precentral area	Wald ratio	0.148159213	0.05	1
	a2009s lh G precuneus area	Wald ratio	0.716129569	0.05	1
	IDP dMRI TBSS MD Cingulum cingulate gyrus R	MR Egger	0.918331423	0.05	1
		Weighted median	0.445734871	0.05	1
		Inverse variance weighted	0.612209281	0.05	1
		Simple mode	0.50923674	0.05	1
		Weighted mode	0.526021893	0.05	1
		MR Egger	0.939905145	0.05	1

	IDP dMRI TBSS MD Cingulum cingulate gyrus L	Weighted median	0.449646962	0.05	1
		Inverse variance weighted	0.617033071	0.05	1
		Simple mode	0.552092635	0.05	1
		Weighted mode	0.518518882	0.05	1
	a2009s lh S calcarine area	Wald ratio	0.670903433	0.05	1
	a2009s lh S central area	Wald ratio	0.144916097	0.05	1
	a2009s lh S collat transv ant area	Wald ratio	0.454588276	0.05	1
	a2009s lh S front middle area	Wald ratio	0.554866465	0.05	1
	IDP dMRI TBSS MD Cingulum hippocampus R	Wald ratio	0.493062519	0.05	1
	a2009s lh S intrapariet&P trans area	Wald ratio	0.061471233	0.05	1
	IDP dMRI TBSS MD Cingulum hippocampus L	Wald ratio	0.493062519	0.05	1
	a2009s lh S subparietal area	Wald ratio	0.670903433	0.05	1
	DKTatlas rh cuneus area	Wald ratio	0.670903433	0.05	1
	DKTatlas rh lingual area	Wald ratio	0.668235142	0.05	1
	DKTatlas rh parstriangularis area	Wald ratio	0.992646329	0.05	1
	DKTatlas rh pericalcarine area	Inverse variance weighted	0.359196203	0.05	1
	IDP dMRI TBSS MD Superior longitudinal fasciculus R	Wald ratio	0.493062519	0.05	1
	DKTatlas rh postcentral area	Wald ratio	0.144916097	0.05	1
	DKTatlas rh precentral area	Wald ratio	0.148159213	0.05	1
	IDP dMRI TBSS MD Superior longitudinal fasciculus L	Wald ratio	0.493062519	0.05	1
	a2009s rh G&S subcentral area	Wald ratio	0.144916097	0.05	1
	a2009s rh G&S cingul-Mid-Post area	Wald ratio	0.116692838	0.05	1

	a2009s rh G cuneus area	Wald ratio	0.670903433	0.05	1
	IDP dMRI TBSS MD Superior fronto-occipital fasciculus L	Wald ratio	0.428431715	0.05	1
	a2009s rh G oc-temp med-Lingual area	Wald ratio	0.668235142	0.05	1
	a2009s rh G parietal sup area	Wald ratio	0.805758375	0.05	1
	IDP dMRI TBSS MD Uncinate fasciculus R	Wald ratio	0.477834241	0.05	1
	a2009s rh G precentral area	Wald ratio	0.148159213	0.05	1
	a2009s rh G precuneus area	Wald ratio	0.063085863	0.05	1
	IDP dMRI TBSS MD Uncinate fasciculus L	Inverse variance weighted	0.86462994	0.05	1
	a2009s rh Pole occipital area	Wald ratio	0.670903433	0.05	1
	a2009s rh S calcarine area	Wald ratio	0.668235142	0.05	1
	a2009s rh S central area	Wald ratio	0.148159213	0.05	1
	a2009s rh S collat transv ant area	Wald ratio	0.454588276	0.05	1
	a2009s rh S orbital med-olfact area	Wald ratio	0.861154326	0.05	1
	IDP dMRI TBSS MO Pontine crossing tract	MR Egger	0.591705509	0.05	1
		Weighted median	0.6713852	0.05	1
		Inverse variance weighted	0.277988348	0.05	1
		Simple mode	0.87349712	0.05	1
		Weighted mode	0.955288691	0.05	1
	DKTatlas lh postcentral thickness	Wald ratio	0.144916097	0.05	1
	a2009s lh G insular short thickness	Wald ratio	0.965963308	0.05	1
	a2009s lh G postcentral thickness	Wald ratio	0.144916097	0.05	1
	IDP T1 SIENAX CSF normalised volume	Inverse variance weighted	0.984105094	0.05	1

	IDP dMRI TBSS MO Medial lemniscus R	Wald ratio	0.79523747	0.05	1
	a2009s lh S postcentral thickness	Wald ratio	0.823835285	0.05	1
	DKTatlas rh lateraloccipital thickness	Wald ratio	0.975010113	0.05	1
	DKTatlas rh parstriangularis thickness	Wald ratio	0.169534085	0.05	1
	DKTatlas rh postcentral thickness	Wald ratio	0.144916097	0.05	1
	DKTatlas rh posteriorcingulate thickness	Inverse variance weighted	0.202128821	0.05	1
	a2009s rh G&S cingul-Mid-Ant thickness	Wald ratio	0.09644509	0.05	1
	a2009s rh G cuneus thickness	Wald ratio	0.975010113	0.05	1
	a2009s rh G postcentral thickness	Wald ratio	0.144916097	0.05	1
	a2009s rh Pole occipital thickness	Wald ratio	0.975010113	0.05	1
	a2009s rh S oc sup&transversal thickness	Wald ratio	0.789283318	0.05	1
	a2009s rh S parieto occipital thickness	Wald ratio	0.89121531	0.05	1
	IDP dMRI TBSS MO Anterior corona radiata R	Wald ratio	0.129734038	0.05	1
	IDP dMRI TBSS MO Superior corona radiata R	Wald ratio	0.131833102	0.05	1
	IDP dMRI TBSS MO Cingulum cingulate gyrus L	Inverse variance weighted	0.217063166	0.05	1
	IDP dMRI TBSS L1 Anterior limb of internal capsule L	Wald ratio	0.486551461	0.05	1
	IDP dMRI TBSS L1 Posterior limb of internal capsule R	Wald ratio	0.929312641	0.05	1
	IDP dMRI TBSS L1 Posterior limb of internal capsule L	Wald ratio	0.344942579	0.05	1
	IDP dMRI TBSS L1 Anterior corona radiata R	Wald ratio	0.521356628	0.05	1
	IDP dMRI TBSS L1 Anterior corona radiata L	Wald ratio	0.521356628	0.05	1
	IDP dMRI TBSS L1 Posterior corona radiata R	Wald ratio	0.6361583	0.05	1

	IDP dMRI TBSS L1 Posterior corona radiata L	Wald ratio	0.521356628	0.05	1
	IDP dMRI TBSS L1 Sagittal stratum L	Wald ratio	0.521356628	0.05	1
	IDP dMRI TBSS L1 External capsule R	Wald ratio	0.521356628	0.05	1
	IDP dMRI TBSS L1 External capsule L	Inverse variance weighted	0.737500468	0.05	1
	IDP dMRI TBSS L1 Fornix cres+Stria terminalis L	Wald ratio	0.661480653	0.05	1
	IDP T1 FAST ROIs L precentral gyrus	Wald ratio	0.144916097	0.05	1
	IDP dMRI TBSS L1 Superior longitudinal fasciculus R	Inverse variance weighted	0.343496708	0.05	1
	IDP dMRI TBSS L1 Uncinate fasciculus L	Wald ratio	0.944635853	0.05	1
	IDP dMRI TBSS L2 Pontine crossing tract	Wald ratio	0.79523747	0.05	1
	IDP dMRI TBSS L2 Genu of corpus callosum	MR Egger	0.56431563	0.05	1
		Weighted median	0.634578038	0.05	1
		Inverse variance weighted	0.81857082	0.05	1
		Simple mode	0.710290619	0.05	1
		Weighted mode	0.591172681	0.05	1
	IDP dMRI TBSS L2 Body of corpus callosum	Wald ratio	0.521356628	0.05	1
	IDP dMRI TBSS L2 Splenium of corpus callosum	MR Egger	0.323646384	0.05	0.112751172
		Weighted median	0.733511004	0.05	1
		Inverse variance weighted	0.635570711	0.05	1
		Simple mode	0.664066574	0.05	1
		Weighted mode	0.651139284	0.05	1
	IDP dMRI TBSS L2 Corticospinal tract R	Wald ratio	0.168934814	0.05	1
	IDP T1 SIENAX CSF unnormalised volume	MR Egger	0.849323578	0.05	1

		Weighted median	0.384210897	0.05	1
		Inverse variance weighted	0.493124818	0.05	1
		Simple mode	0.299223199	0.05	1
		Weighted mode	0.56031557	0.05	1
	IDP T1 FAST ROIs L temporal pole	Wald ratio	0.441959274	0.05	1
	IDP dMRI TBSS L2 Superior cerebellar peduncle R	Inverse variance weighted	0.537652709	0.05	1
	IDP dMRI TBSS L2 Superior cerebellar peduncle L	Wald ratio	0.889224378	0.05	1
	IDP dMRI TBSS L2 Posterior limb of internal capsule R	Inverse variance weighted	0.572860299	0.05	1
	IDP dMRI TBSS L2 Posterior limb of internal capsule L	Wald ratio	0.845206557	0.05	1
	IDP dMRI TBSS L2 Retrolenticular part of internal capsule R	Inverse variance weighted	0.348800177	0.05	1
	IDP dMRI TBSS L2 Anterior corona radiata R	Inverse variance weighted	0.355251903	0.05	1
	IDP dMRI TBSS L2 Anterior corona radiata L	Wald ratio	0.521356628	0.05	1
	IDP dMRI TBSS L2 Superior corona radiata R	Inverse variance weighted	0.886653056	0.05	1
	IDP dMRI TBSS L2 Superior corona radiata L	Wald ratio	0.493062519	0.05	1
	IDP dMRI TBSS L2 Posterior corona radiata R	Wald ratio	0.493062519	0.05	1
	IDP dMRI TBSS L2 Posterior corona radiata L	Wald ratio	0.493062519	0.05	1
	IDP dMRI TBSS L2 Posterior thalamic radiation R	Wald ratio	0.493062519	0.05	1
	IDP dMRI TBSS L2 Posterior thalamic radiation L	Wald ratio	0.493062519	0.05	1
	IDP dMRI TBSS L2 Sagittal stratum R	Wald ratio	0.493062519	0.05	1

	IDP dMRI TBSS L2 Cingulum cingulate gyrus R	Wald ratio	0.521356628	0.05	1
	IDP dMRI TBSS L2 Cingulum hippocampus R	Wald ratio	0.493062519	0.05	1
	IDP dMRI TBSS L2 Cingulum hippocampus L	Wald ratio	0.521356628	0.05	1
	IDP dMRI TBSS L2 Uncinate fasciculus L	Wald ratio	0.521356628	0.05	1
	IDP dMRI TBSS L3 Genu of corpus callosum	Wald ratio	0.521356628	0.05	1
	IDP dMRI TBSS L3 Body of corpus callosum	Wald ratio	0.521356628	0.05	1
	IDP dMRI TBSS L3 Splenium of corpus callosum	Wald ratio	0.819202033	0.05	1
	IDP dMRI TBSS L3 Inferior cerebellar peduncle R	Wald ratio	0.521356628	0.05	1
	IDP dMRI TBSS L3 Inferior cerebellar peduncle L	Wald ratio	0.521356628	0.05	1
	IDP dMRI TBSS L3 Superior cerebellar peduncle R	Inverse variance weighted	0.563202337	0.05	1
	IDP dMRI TBSS L3 Superior cerebellar peduncle L	Wald ratio	0.889224378	0.05	1
	IDP dMRI TBSS L3 Cerebral peduncle R	Wald ratio	0.262910998	0.05	1
	IDP dMRI TBSS L3 Anterior limb of internal capsule R	Wald ratio	0.357295967	0.05	1
	IDP dMRI TBSS L3 Anterior limb of internal capsule L	Wald ratio	0.473285954	0.05	1
	IDP dMRI TBSS L3 Retrolenticular part of internal capsule R	Wald ratio	0.493062519	0.05	1
	IDP dMRI TBSS L3 Anterior corona radiata R	Inverse variance weighted	0.355069534	0.05	1
	IDP dMRI TBSS L3 Anterior corona radiata L	Inverse variance weighted	0.359856261	0.05	1
	IDP dMRI TBSS L3 Posterior corona radiata R	Inverse variance weighted	0.951533925	0.05	1
	IDP dMRI TBSS L3 Posterior thalamic radiation R	Wald ratio	0.493062519	0.05	1

	IDP dMRI TBSS L3 Posterior thalamic radiation L	Wald ratio	0.493062519	0.05	1
	IDP dMRI TBSS L3 Sagittal stratum R	Wald ratio	0.493062519	0.05	1
	IDP dMRI TBSS L3 Sagittal stratum L	Wald ratio	0.493062519	0.05	1
	IDP dMRI TBSS L3 External capsule R	Wald ratio	0.521356628	0.05	1
	IDP dMRI TBSS L3 External capsule L	Wald ratio	0.521356628	0.05	1
	IDP dMRI TBSS L3 Cingulum cingulate gyrus R	Inverse variance weighted	0.358198018	0.05	1
	IDP dMRI TBSS L3 Cingulum cingulate gyrus L	Wald ratio	0.521356628	0.05	1
	IDP dMRI TBSS L3 Cingulum hippocampus R	Wald ratio	0.521356628	0.05	1
	IDP dMRI TBSS L3 Cingulum hippocampus L	Wald ratio	0.521356628	0.05	1
	IDP dMRI TBSS L3 Fornix cres+Stria terminalis R	Wald ratio	0.787247678	0.05	1
	IDP dMRI TBSS L3 Superior longitudinal fasciculus R	Wald ratio	0.493062519	0.05	1
	IDP dMRI TBSS L3 Superior longitudinal fasciculus L	Wald ratio	0.493062519	0.05	1
	IDP dMRI TBSS L3 Uncinate fasciculus R	Wald ratio	0.521356628	0.05	1
	IDP dMRI TBSS L3 Uncinate fasciculus L	Inverse variance weighted	0.866252253	0.05	1
	IDP dMRI TBSS ICVF Middle cerebellar peduncle	Wald ratio	0.493062519	0.05	1
	IDP dMRI TBSS ICVF Genu of corpus callosum	MR Egger	0.733303714	0.05	1
		Weighted median	0.471883782	0.05	1
		Inverse variance weighted	0.699140522	0.05	1
		Simple mode	0.507154865	0.05	1
		Weighted mode	0.541690268	0.05	1

	IDP dMRI TBSS ICVF Body of corpus callosum	MR Egger	0.973523508	0.05	1
		Weighted median	0.82942453	0.05	1
		Inverse variance weighted	0.556559161	0.05	1
		Simple mode	0.859761359	0.05	1
		Weighted mode	0.912811253	0.05	1
	IDP dMRI TBSS ICVF Splenium of corpus callosum	MR Egger	0.762313656	0.05	1
		Weighted median	0.322153923	0.05	1
		Inverse variance weighted	0.178430001	0.05	1
		Simple mode	0.310244358	0.05	1
		Weighted mode	0.913936155	0.05	1
	IDP dMRI TBSS ICVF Fornix	Wald ratio	0.428431715	0.05	1
	IDP dMRI TBSS ICVF Medial lemniscus R	Inverse variance weighted	0.278862192	0.05	1
	IDP dMRI TBSS ICVF Medial lemniscus L	Wald ratio	0.832012471	0.05	1
	IDP dMRI TBSS ICVF Inferior cerebellar peduncle R	Inverse variance weighted	0.738045289	0.05	1
	IDP dMRI TBSS ICVF Inferior cerebellar peduncle L	Inverse variance weighted	0.732587623	0.05	1
	IDP dMRI TBSS ICVF Superior cerebellar peduncle R	MR Egger	0.981981958	0.05	1
		Weighted median	0.917011882	0.05	1
		Inverse variance weighted	0.726056389	0.05	1
		Simple mode	0.975663803	0.05	1
		Weighted mode	0.946200446	0.05	1
	IDP dMRI TBSS ICVF Superior cerebellar peduncle L	Inverse variance weighted	0.764371098	0.05	1
	IDP dMRI TBSS ICVF Cerebral peduncle R	MR Egger	0.594913542	0.05	1

		Weighted median	0.99740167	0.05	1
		Inverse variance weighted	0.851161677	0.05	1
		Simple mode	0.927030647	0.05	1
		Weighted mode	0.838433332	0.05	1
	IDP dMRI TBSS ICVF Cerebral peduncle L	Wald ratio	0.831729927	0.05	1
	IDP dMRI TBSS ICVF Anterior limb of internal capsule R	Wald ratio	0.521356628	0.05	1
	IDP dMRI TBSS ICVF Anterior limb of internal capsule L	MR Egger	0.184329809	0.05	1
		Weighted median	0.918363374	0.05	1
		Inverse variance weighted	0.401775746	0.05	1
		Simple mode	0.597550372	0.05	1
		Weighted mode	0.717467418	0.05	1
	IDP dMRI TBSS ICVF Posterior limb of internal capsule R	MR Egger	0.387142968	0.05	1
		Weighted median	0.967697104	0.05	1
		Inverse variance weighted	0.648925811	0.05	1
		Simple mode	0.784286048	0.05	1
		Weighted mode	0.777177712	0.05	1
	IDP dMRI TBSS ICVF Posterior limb of internal capsule L	MR Egger	0.632629494	0.05	1
		Weighted median	0.467685883	0.05	1
		Inverse variance weighted	0.950879658	0.05	1
		Simple mode	0.536962848	0.05	1
		Weighted mode	0.508301147	0.05	1
		MR Egger	0.589884962	0.05	1

	IDP dMRI TBSS ICVF Retrolenticular part of internal capsule R	Weighted median	0.456103478	0.05	1
		Inverse variance weighted	0.981636835	0.05	1
		Simple mode	0.50303855	0.05	1
		Weighted mode	0.743377173	0.05	1
	IDP dMRI TBSS ICVF Retrolenticular part of internal capsule L	MR Egger	0.555448619	0.05	1
		Weighted median	0.44351135	0.05	1
		Inverse variance weighted	0.863401124	0.05	1
		Simple mode	0.488583697	0.05	1
		Weighted mode	0.530241042	0.05	1
	IDP dMRI TBSS ICVF Anterior corona radiata R	MR Egger	0.538830707	0.05	1
		Weighted median	0.425413261	0.05	1
		Inverse variance weighted	0.897901463	0.05	1
		Simple mode	0.527832324	0.05	1
		Weighted mode	0.507724089	0.05	1
	IDP dMRI TBSS ICVF Anterior corona radiata L	MR Egger	0.584981833	0.05	1
		Weighted median	0.464385668	0.05	1
		Inverse variance weighted	0.897341437	0.05	1
		Simple mode	0.502417651	0.05	1
		Weighted mode	0.529844695	0.05	1
	IDP dMRI TBSS ICVF Superior corona radiata R	MR Egger	0.48494434	0.05	1
		Weighted median	0.428916888	0.05	1
		Inverse variance weighted	0.890171941	0.05	1

		Simple mode	0.514672368	0.05	1
		Weighted mode	0.498371387	0.05	1
	IDP dMRI TBSS ICVF Superior corona radiata L	MR Egger	0.519228132	0.05	1
		Weighted median	0.432676478	0.05	1
		Inverse variance weighted	0.893560845	0.05	1
		Simple mode	0.516922548	0.05	1
		Weighted mode	0.51090843	0.05	1
	IDP dMRI TBSS ICVF Posterior corona radiata R	MR Egger	0.61392738	0.05	1
		Weighted median	0.436879084	0.05	1
		Inverse variance weighted	0.652671932	0.05	1
		Simple mode	0.496994537	0.05	1
		Weighted mode	0.537534406	0.05	1
	IDP dMRI TBSS ICVF Posterior corona radiata L	MR Egger	0.583692161	0.05	1
		Weighted median	0.434437082	0.05	1
		Inverse variance weighted	0.623990562	0.05	1
		Simple mode	0.501216467	0.05	1
		Weighted mode	0.528602447	0.05	1
	IDP dMRI TBSS ICVF Posterior thalamic radiation R	Wald ratio	0.493062519	0.05	1
	IDP dMRI TBSS ICVF Posterior thalamic radiation L	Inverse variance weighted	0.861313084	0.05	1
	IDP dMRI TBSS ICVF Sagittal stratum R	MR Egger	0.606405686	0.05	1
		Weighted median	0.539233246	0.05	1
		Inverse variance weighted	0.340175083	0.05	1

		Simple mode	0.854810966	0.05	1
		Weighted mode	0.80783302	0.05	1
	IDP dMRI TBSS ICVF Sagittal stratum L	Inverse variance weighted	0.780195793	0.05	1
	IDP dMRI TBSS ICVF External capsule R	MR Egger	0.815330105	0.05	1
		Weighted median	0.506268228	0.05	1
		Inverse variance weighted	0.665051611	0.05	1
		Simple mode	0.565180022	0.05	1
		Weighted mode	0.556761839	0.05	1
	IDP dMRI TBSS ICVF External capsule L	Inverse variance weighted	0.794374457	0.05	1
	IDP dMRI TBSS ICVF Cingulum cingulate gyrus R	Inverse variance weighted	0.375125998	0.05	1
	IDP dMRI TBSS ICVF Cingulum cingulate gyrus L	Wald ratio	0.653590016	0.05	1
	IDP dMRI TBSS ICVF Cingulum hippocampus R	MR Egger	0.631626812	0.05	1
		Weighted median	0.45444306	0.05	1
		Inverse variance weighted	0.564302156	0.05	1
		Simple mode	0.512599654	0.05	1
		Weighted mode	0.529107274	0.05	1
	IDP dMRI TBSS ICVF Cingulum hippocampus L	MR Egger	0.586726359	0.05	1
		Weighted median	0.441792901	0.05	1
		Inverse variance weighted	0.852343119	0.05	1
		Simple mode	0.518531959	0.05	1
		Weighted mode	0.512111114	0.05	1

	IDP dMRI TBSS ICVF Fornix cres+Stria terminalis R	Wald ratio	0.888104166	0.05	1
	IDP dMRI TBSS ICVF Superior longitudinal fasciculus R	Inverse variance weighted	0.813587509	0.05	1
	IDP dMRI TBSS ICVF Superior longitudinal fasciculus L	MR Egger	0.423956852	0.05	1
		Weighted median	0.392717239	0.05	1
		Inverse variance weighted	0.855528992	0.05	1
		Simple mode	0.502458805	0.05	1
		Weighted mode	0.472302124	0.05	1
	IDP dMRI TBSS ICVF Superior fronto-occipital fasciculus R	Inverse variance weighted	0.475235003	0.05	1
	IDP dMRI TBSS ICVF Uncinate fasciculus R	Wald ratio	0.521356628	0.05	1
	IDP dMRI TBSS ICVF Uncinate fasciculus L	Wald ratio	0.521356628	0.05	1
	IDP dMRI TBSS OD Pontine crossing tract	MR Egger	0.684879759	0.05	1
		Weighted median	0.19940097	0.05	1
		Inverse variance weighted	0.436904156	0.05	1
		Simple mode	0.419888472	0.05	1
		Weighted mode	0.432965797	0.05	1
	IDP dMRI TBSS OD Cerebral peduncle R	Wald ratio	0.679505007	0.05	1
	IDP dMRI TBSS OD Cerebral peduncle L	Wald ratio	0.679505007	0.05	1
	IDP dMRI TBSS OD Posterior limb of internal capsule L	Wald ratio	0.344942579	0.05	1
	IDP dMRI TBSS OD External capsule R	Inverse variance weighted	0.130114322	0.05	1
	IDP dMRI TBSS OD External capsule L	Wald ratio	0.383638343	0.05	1
	IDP dMRI TBSS OD Superior longitudinal fasciculus R	Wald ratio	0.18211806	0.05	1

	IDP dMRI TBSS ISOVF Fornix	Wald ratio	0.253830021	0.05	1
	IDP dMRI TBSS ISOVF External capsule R	Wald ratio	0.426966955	0.05	1
	IDP dMRI TBSS ISOVF Cingulum cingulate gyrus R	Wald ratio	0.718543472	0.05	1
	IDP dMRI ProbtrackX FA ar l	Wald ratio	0.521356628	0.05	1
	IDP dMRI ProbtrackX FA atr l	Wald ratio	0.521356628	0.05	1
	IDP dMRI ProbtrackX FA fmi	MR Egger	0.509069276	0.05	1
		Weighted median	0.545981454	0.05	1
		Inverse variance weighted	0.978976946	0.05	1
		Simple mode	0.599115813	0.05	1
		Weighted mode	0.501160719	0.05	1
	IDP dMRI ProbtrackX FA ifo l	Wald ratio	0.493062519	0.05	1
	IDP dMRI ProbtrackX FA ifo r	Wald ratio	0.458218193	0.05	1
	IDP dMRI ProbtrackX FA ml l	Wald ratio	0.044420499	0.05	1
	IDP dMRI ProbtrackX FA ptr r	Wald ratio	0.937268299	0.05	1
	IDP dMRI ProbtrackX FA slf l	Wald ratio	0.455378189	0.05	1
	IDP dMRI ProbtrackX FA str l	Wald ratio	0.699146077	0.05	1
	IDP dMRI ProbtrackX FA unc l	Wald ratio	0.521356628	0.05	1
	IDP dMRI ProbtrackX FA unc r	Wald ratio	0.521356628	0.05	1
	IDP dMRI ProbtrackX MD ar l	Wald ratio	0.521356628	0.05	1
	IDP dMRI ProbtrackX MD ar r	Wald ratio	0.521356628	0.05	1
	IDP dMRI ProbtrackX MD atr l	Wald ratio	0.521356628	0.05	1
	IDP dMRI ProbtrackX MD atr r	Wald ratio	0.521356628	0.05	1
	IDP dMRI ProbtrackX MD cgc l	MR Egger	0.960652684	0.05	1

		Weighted median	0.475370313	0.05	1
		Inverse variance weighted	0.639792011	0.05	1
		Simple mode	0.500724189	0.05	1
		Weighted mode	0.534260855	0.05	1
	IDP dMRI ProbtrackX MD cgc r	Inverse variance weighted	0.962772996	0.05	1
	IDP dMRI ProbtrackX MD fmi	Inverse variance weighted	0.35838964	0.05	1
	IDP dMRI ProbtrackX MD ifo l	Wald ratio	0.493062519	0.05	1
	IDP dMRI ProbtrackX MD ifo r	Wald ratio	0.493062519	0.05	1
	IDP dMRI ProbtrackX MD ilf l	Wald ratio	0.493062519	0.05	1
	IDP dMRI ProbtrackX MD ilf r	Wald ratio	0.493062519	0.05	1
	IDP dMRI ProbtrackX MD ptr l	Wald ratio	0.521356628	0.05	1
	IDP dMRI ProbtrackX MD ptr r	Wald ratio	0.521356628	0.05	1
	IDP dMRI ProbtrackX MD slf l	Inverse variance weighted	0.35575617	0.05	1
	IDP dMRI ProbtrackX MD slf r	Wald ratio	0.493062519	0.05	1
	IDP dMRI ProbtrackX MD str l	Wald ratio	0.202957464	0.05	1
	IDP dMRI ProbtrackX MD str r	Wald ratio	0.521356628	0.05	1
	IDP dMRI ProbtrackX MD unc l	Wald ratio	0.521356628	0.05	1
	IDP dMRI ProbtrackX MD unc r	Inverse variance weighted	0.418413644	0.05	1
	IDP dMRI ProbtrackX MO atr l	Wald ratio	0.498353083	0.05	1
	IDP dMRI ProbtrackX MO ml r	Wald ratio	0.448182051	0.05	1
	IDP T1 FAST ROIs L intracalc cortex	Wald ratio	0.670903433	0.05	1
	IDP dMRI ProbtrackX L1 ilf l	Wald ratio	0.521356628	0.05	1

	IDP T1 FAST ROIs R intracalc cortex	Wald ratio	0.670903433	0.05	1
	IDP dMRI ProbtrackX L1 slf l	Wald ratio	0.521356628	0.05	1
	IDP dMRI ProbtrackX L1 slf r	Wald ratio	0.27341234	0.05	1
	IDP dMRI ProbtrackX L1 str r	Wald ratio	0.510433227	0.05	1
	IDP dMRI ProbtrackX L1 unc l	Inverse variance weighted	0.921041555	0.05	1
	IDP dMRI ProbtrackX L1 unc r	Wald ratio	0.618863344	0.05	1
	IDP dMRI ProbtrackX L2 ar l	Wald ratio	0.521356628	0.05	1
	IDP dMRI ProbtrackX L2 ar r	Wald ratio	0.521356628	0.05	1
	IDP dMRI ProbtrackX L2 atr l	Wald ratio	0.521356628	0.05	1
	IDP dMRI ProbtrackX L2 atr r	Wald ratio	0.521356628	0.05	1
	IDP dMRI ProbtrackX L2 cgh l	Wald ratio	0.110659003	0.05	1
	IDP dMRI ProbtrackX L2 fma	Wald ratio	0.402950363	0.05	1
	IDP dMRI ProbtrackX L2 fmi	Inverse variance weighted	0.358139662	0.05	1
	IDP dMRI ProbtrackX L2 ifo l	Wald ratio	0.493062519	0.05	1
	IDP dMRI ProbtrackX L2 ifo r	Wald ratio	0.493062519	0.05	1
	IDP dMRI ProbtrackX L2 ilf l	Wald ratio	0.493062519	0.05	1
	IDP dMRI ProbtrackX L2 ilf r	Wald ratio	0.493062519	0.05	1
	IDP dMRI ProbtrackX L2 ptr l	Wald ratio	0.521356628	0.05	1
	IDP dMRI ProbtrackX L2 ptr r	Wald ratio	0.509771954	0.05	1
	IDP dMRI ProbtrackX L2 slf l	Inverse variance weighted	0.35514367	0.05	1
	IDP dMRI ProbtrackX L2 slf r	Wald ratio	0.493062519	0.05	1
	IDP dMRI ProbtrackX L2 str l	Wald ratio	0.202957464	0.05	1
	IDP dMRI ProbtrackX L2 str r	Wald ratio	0.202957464	0.05	1

	IDP dMRI ProbtrackX L2 unc l	Wald ratio	0.521356628	0.05	1
	IDP dMRI ProbtrackX L2 unc r	Inverse variance weighted	0.419259513	0.05	1
	IDP dMRI ProbtrackX L3 ar l	Wald ratio	0.521356628	0.05	1
	IDP dMRI ProbtrackX L3 ar r	Wald ratio	0.521356628	0.05	1
	IDP dMRI ProbtrackX L3 atr l	Wald ratio	0.521356628	0.05	1
	IDP dMRI ProbtrackX L3 atr r	Wald ratio	0.521356628	0.05	1
	IDP dMRI ProbtrackX L3 fmi	MR Egger	0.517463553	0.05	1
		Weighted median	0.420762689	0.05	1
		Inverse variance weighted	0.93401473	0.05	1
		Simple mode	0.529942877	0.05	1
		Weighted mode	0.502214524	0.05	1
	IDP dMRI ProbtrackX L3 ifo l	Inverse variance weighted	0.363285176	0.05	1
	IDP dMRI ProbtrackX L3 ifo r	Wald ratio	0.493062519	0.05	1
	IDP dMRI ProbtrackX L3 ilf l	Wald ratio	0.493062519	0.05	1
	IDP dMRI ProbtrackX L3 ilf r	Wald ratio	0.493062519	0.05	1
	IDP dMRI ProbtrackX L3 ptr l	Wald ratio	0.521356628	0.05	1
	IDP dMRI ProbtrackX L3 ptr r	Wald ratio	0.509771954	0.05	1
	IDP dMRI ProbtrackX L3 slf l	Inverse variance weighted	0.356984758	0.05	1
	IDP dMRI ProbtrackX L3 slf r	Wald ratio	0.493062519	0.05	1
	IDP dMRI ProbtrackX L3 str l	Wald ratio	0.509771954	0.05	1
	IDP dMRI ProbtrackX L3 unc l	Wald ratio	0.521356628	0.05	1
	IDP dMRI ProbtrackX L3 unc r	Inverse variance weighted	0.41744252	0.05	1

	IDP dMRI ProbtrackX ICVF ar l	MR Egger	0.635935719	0.05	1
		Weighted median	0.469007892	0.05	1
		Inverse variance weighted	0.943662172	0.05	1
		Simple mode	0.593470979	0.05	1
		Weighted mode	0.559935116	0.05	1
	IDP dMRI ProbtrackX ICVF ar r	MR Egger	0.607139213	0.05	1
		Weighted median	0.46820083	0.05	1
		Inverse variance weighted	0.937627897	0.05	1
		Simple mode	0.594664349	0.05	1
		Weighted mode	0.566286409	0.05	1
	IDP dMRI ProbtrackX ICVF atr l	MR Egger	0.640651573	0.05	1
		Weighted median	0.469067501	0.05	1
		Inverse variance weighted	0.894385572	0.05	1
		Simple mode	0.496132592	0.05	1
		Weighted mode	0.52539557	0.05	1
	IDP dMRI ProbtrackX ICVF atr r	MR Egger	0.651165227	0.05	1
		Weighted median	0.469936894	0.05	1
		Inverse variance weighted	0.913387921	0.05	1
		Simple mode	0.521701417	0.05	1
		Weighted mode	0.500981893	0.05	1
	IDP dMRI ProbtrackX ICVF cgc l	Wald ratio	0.653590016	0.05	1
	IDP dMRI ProbtrackX ICVF cgh l	MR Egger	0.653404953	0.05	1

		Weighted median	0.489194661	0.05	1
		Inverse variance weighted	0.900349945	0.05	1
		Simple mode	0.496762653	0.05	1
		Weighted mode	0.497214899	0.05	1
	IDP dMRI ProbtrackX ICVF cgh r	Wald ratio	0.521356628	0.05	1
	IDP dMRI ProbtrackX ICVF cst l	MR Egger	0.612226176	0.05	1
		Weighted median	0.460939984	0.05	1
		Inverse variance weighted	0.992940361	0.05	1
		Simple mode	0.620317999	0.05	1
		Weighted mode	0.533565047	0.05	1
	IDP dMRI ProbtrackX ICVF cst r	MR Egger	0.38714695	0.05	1
		Weighted median	0.632605359	0.05	1
		Inverse variance weighted	0.935068977	0.05	1
		Simple mode	0.688946211	0.05	1
		Weighted mode	0.546787159	0.05	1
	IDP dMRI ProbtrackX ICVF fma	Inverse variance weighted	0.526425117	0.05	1
	IDP dMRI ProbtrackX ICVF fmi	MR Egger	0.460279621	0.05	1
		Weighted median	0.487018125	0.05	1
		Inverse variance weighted	0.818774702	0.05	1
		Simple mode	0.599528097	0.05	1
		Weighted mode	0.518229681	0.05	1
	IDP dMRI ProbtrackX ICVF ifo l	Inverse variance weighted	0.371218793	0.05	1

	IDP dMRI ProbtrackX ICVF ifo r	Inverse variance weighted	0.370421751	0.05	1
	IDP dMRI ProbtrackX ICVF ilf l	Inverse variance weighted	0.36804063	0.05	1
	IDP dMRI ProbtrackX ICVF ilf r	Wald ratio	0.493062519	0.05	1
	IDP dMRI ProbtrackX ICVF mcp	Wald ratio	0.089130926	0.05	1
	IDP dMRI ProbtrackX ICVF ml l	Wald ratio	0.944635853	0.05	1
	IDP dMRI ProbtrackX ICVF ml r	Wald ratio	0.521356628	0.05	1
	IDP dMRI ProbtrackX ICVF ptr l	Wald ratio	0.493062519	0.05	1
	IDP dMRI ProbtrackX ICVF ptr r	Inverse variance weighted	0.42858964	0.05	1
	IDP dMRI ProbtrackX ICVF slf l	MR Egger	0.515437594	0.05	1
		Weighted median	0.417340252	0.05	1
		Inverse variance weighted	0.875959784	0.05	1
		Simple mode	0.514230799	0.05	1
		Weighted mode	0.493248167	0.05	1
	IDP dMRI ProbtrackX ICVF slf r	Inverse variance weighted	0.801631544	0.05	1
	IDP dMRI ProbtrackX ICVF str l	MR Egger	0.612517212	0.05	1
		Weighted median	0.447188675	0.05	1
		Inverse variance weighted	0.932903484	0.05	1
		Simple mode	0.520220422	0.05	1
		Weighted mode	0.502591725	0.05	1
	IDP dMRI ProbtrackX ICVF str r	MR Egger	0.552073597	0.05	1
		Weighted median	0.418770606	0.05	1
		Inverse variance weighted	0.904964336	0.05	1

		Simple mode	0.520787837	0.05	1
		Weighted mode	0.533413231	0.05	1
	IDP dMRI ProbtrackX ICVF unc l	Inverse variance weighted	0.957839724	0.05	1
	IDP dMRI ProbtrackX ICVF unc r	Inverse variance weighted	0.385891237	0.05	1
	IDP dMRI ProbtrackX OD atr r	Wald ratio	0.381922792	0.05	1
	IDP dMRI ProbtrackX OD ilf l	Wald ratio	0.976650615	0.05	1
	IDP dMRI ProbtrackX OD slf l	Wald ratio	0.224178133	0.05	1
	IDP dMRI ProbtrackX OD str l	Inverse variance weighted	0.937876767	0.05	1
	IDP dMRI ProbtrackX ISOVF atr r	Wald ratio	0.521356628	0.05	1
	IDP dMRI ProbtrackX ISOVF cgc l	Wald ratio	0.301595069	0.05	1
	IDP dMRI ProbtrackX ISOVF ifo r	Wald ratio	0.53006488	0.05	1
	IDP dMRI ProbtrackX ISOVF ilf l	Wald ratio	0.735896668	0.05	1
	IDP dMRI ProbtrackX ISOVF ilf r	MR Egger	0.470200782	0.05	0.005413142
		Weighted median	0.677626541	0.05	1
		Inverse variance weighted	0.605917037	0.05	1
		Simple mode	0.716480718	0.05	1
		Weighted mode	0.775127919	0.05	1
	IDP dMRI ProbtrackX ISOVF slf l	Inverse variance weighted	0.501741812	0.05	1
	IDP dMRI ProbtrackX ISOVF slf r	MR Egger	0.85795448	0.05	1
		Weighted median	0.753115013	0.05	1
		Inverse variance weighted	0.753715082	0.05	1
		Simple mode	0.710200574	0.05	1

		Weighted mode	0.731204563	0.05	1
	IDP dMRI ProbtrackX ISOVF unc l	Wald ratio	0.583538551	0.05	1
	IDP dMRI ProbtrackX ISOVF unc r	Wald ratio	0.618863344	0.05	1
	IDP T1 SIENAX brain-normalised volume	Wald ratio	0.125581563	0.05	1
Systemic lupus erythematosus	IDP T1 SIENAX peripheral grey normalised volume	Wald ratio	0.532114245	0.05	1
	IDP T1 FAST ROIs R temp occ fusif cortex	Wald ratio	0.677023812	0.05	1
	IDP T1 FAST ROIs R occ pole	Inverse variance weighted	0.852059242	0.05	1
	IDP T1 FAST ROIs L thalamus	Inverse variance weighted	0.532423045	0.05	1
	IDP T1 FAST ROIs R thalamus	Wald ratio	0.116791622	0.05	1
	IDP T1 FAST ROIs L putamen	Wald ratio	0.64470385	0.05	1
	IDP T1 FAST ROIs R putamen	Wald ratio	0.64470385	0.05	1
	IDP T1 FAST ROIs L pallidum	Wald ratio	0.904417545	0.05	1
	IDP T1 FIRST left caudate volume	Wald ratio	0.382001812	0.05	1
	IDP T1 FAST ROIs L hippocampus	Inverse variance weighted	0.914479045	0.05	1
	IDP T1 FAST ROIs R hippocampus	Wald ratio	0.435245553	0.05	1
	IDP T1 FAST ROIs L ventral striatum	Wald ratio	0.64470385	0.05	1
	IDP T1 FAST ROIs R ventral striatum	Inverse variance weighted	0.413252806	0.05	1
	IDP T1 FAST ROIs L cerebellum VI	Wald ratio	0.779669715	0.05	1
	IDP T1 FAST ROIs L cerebellum crus I	MR Egger	0.660457596	0.05	1
		Weighted median	0.242879661	0.05	1
		Inverse variance weighted	0.031595945	0.05	1
		Simple mode	0.628399244	0.05	1

		Weighted mode	0.54745209	0.05	1
	IDP T1 FAST ROIs R cerebellum crus I	Inverse variance weighted	0.490794602	0.05	1
	IDP T1 FAST ROIs L cerebellum crus II	MR Egger	0.683877376	0.05	1
		Weighted median	0.494802456	0.05	1
		Inverse variance weighted	0.798583111	0.05	1
		Simple mode	0.610789098	0.05	1
		Weighted mode	0.606376613	0.05	1
	IDP T1 FAST ROIs V cerebellum crus II	Inverse variance weighted	0.991825164	0.05	1
	IDP T1 FAST ROIs R cerebellum crus II	Inverse variance weighted	0.473350836	0.05	1
	IDP T1 FIRST left putamen volume	Wald ratio	0.960683587	0.05	1
	IDP T1 FAST ROIs R cerebellum VIIb	Wald ratio	0.268518175	0.05	1
	IDP T1 FAST ROIs V cerebellum VIIIa	MR Egger	0.672371649	0.05	1
		Weighted median	0.445059983	0.05	1
		Inverse variance weighted	0.273375055	0.05	1
		Simple mode	0.95816349	0.05	1
		Weighted mode	0.282853849	0.05	1
	IDP T1 FAST ROIs L cerebellum VIIIb	Wald ratio	0.99556381	0.05	1
	IDP T1 FAST ROIs V cerebellum VIIIb	Inverse variance weighted	0.256066441	0.05	1
	IDP T1 FAST ROIs R cerebellum VIIIb	Wald ratio	0.64470385	0.05	1
	IDP T1 FAST ROIs L cerebellum IX	Inverse variance weighted	0.968404008	0.05	1
	IDP T1 FAST ROIs V cerebellum IX	Inverse variance weighted	0.649932521	0.05	1

	IDP T1 FAST ROIs R cerebellum IX	Inverse variance weighted	0.91419709	0.05	1
	IDP T1 FAST ROIs V cerebellum X	Wald ratio	0.64470385	0.05	1
	IDP T2 FLAIR BIANCA WMH volume	Inverse variance weighted	0.082881808	0.05	1
	IDP SWI T2star left caudate	MR Egger	0.916192286	0.05	1
		Weighted median	0.145489889	0.05	1
		Inverse variance weighted	0.141657127	0.05	1
		Simple mode	0.359651327	0.05	1
		Weighted mode	0.249317023	0.05	1
	IDP SWI T2star right caudate	MR Egger	0.817896093	0.05	1
		Weighted median	0.523653053	0.05	1
		Inverse variance weighted	0.193261358	0.05	1
		Simple mode	0.970828424	0.05	1
		Weighted mode	0.803624087	0.05	1
	IDP T1 FIRST left pallidum volume	Wald ratio	0.497214336	0.05	1
	IDP SWI T2star left putamen	MR Egger	0.681939154	0.05	1
		Weighted median	0.107033561	0.05	1
		Inverse variance weighted	0.00930319	0.05	1
		Simple mode	0.368610975	0.05	1
		Weighted mode	0.608239861	0.05	1
	IDP SWI T2star right putamen	MR Egger	0.636411876	0.05	1
		Weighted median	0.595059301	0.05	1
		Inverse variance weighted	0.365014919	0.05	1

		Simple mode	0.924888104	0.05	1
		Weighted mode	0.942285155	0.05	1
	IDP SWI T2star left pallidum	MR Egger	0.694471589	0.05	1
		Weighted median	0.680994736	0.05	1
		Inverse variance weighted	0.773938995	0.05	1
		Simple mode	0.87985782	0.05	1
		Weighted mode	0.801365465	0.05	1
	IDP SWI T2star right pallidum	MR Egger	0.611102976	0.05	1
		Weighted median	0.905241999	0.05	1
		Inverse variance weighted	0.674241954	0.05	1
		Simple mode	0.962646684	0.05	1
		Weighted mode	0.653138913	0.05	1
	IDP T1 FIRST right pallidum volume	Wald ratio	0.497214336	0.05	1
	IDP dMRI TBSS FA Genu of corpus callosum	Wald ratio	0.002602759	0.05	0.993021958
	IDP dMRI TBSS FA Splenium of corpus callosum	MR Egger	0.535835702	0.05	0.976184187
		Weighted median	0.607684788	0.05	1
		Inverse variance weighted	0.974214435	0.05	1
		Simple mode	0.671717986	0.05	1
		Weighted mode	0.658030781	0.05	1
	IDP dMRI TBSS FA Corticospinal tract L	Wald ratio	0.870258755	0.05	1
	IDP dMRI TBSS FA Superior cerebellar peduncle R	Inverse variance weighted	0.962582769	0.05	1
	IDP dMRI TBSS FA Superior cerebellar peduncle L	Inverse variance weighted	0.969983969	0.05	1

	IDP dMRI TBSS FA Posterior limb of internal capsule R	Wald ratio	0.206391086	0.05	1
	IDP dMRI TBSS FA Retrolenticular part of internal capsule R	Inverse variance weighted	0.314023491	0.05	1
	IDP dMRI TBSS FA Retrolenticular part of internal capsule L	Wald ratio	0.215901514	0.05	0.999999965
	IDP dMRI TBSS FA Anterior corona radiata R	Wald ratio	0.002602759	0.05	0.987008224
	IDP dMRI TBSS FA Anterior corona radiata L	Wald ratio	0.002602759	0.05	0.982254006
	IDP dMRI TBSS FA Posterior corona radiata L	Wald ratio	0.215901514	0.05	0.999992548
	IDP dMRI TBSS FA Posterior thalamic radiation R	Inverse variance weighted	0.513199703	0.05	1
	IDP dMRI TBSS FA Posterior thalamic radiation L	Inverse variance weighted	0.542763759	0.05	1
	IDP dMRI TBSS FA Sagittal stratum R	Inverse variance weighted	0.752344904	0.05	1
	IDP dMRI TBSS FA Sagittal stratum L	Wald ratio	0.210732865	0.05	0.999999899
	IDP dMRI TBSS FA Cingulum cingulate gyrus R	Inverse variance weighted	0.045840348	0.05	0.999999469
	IDP dMRI TBSS FA Superior longitudinal fasciculus L	Inverse variance weighted	0.231594379	0.05	0.999999995
	IDP dMRI TBSS FA Uncinate fasciculus L	Wald ratio	0.05077602	0.05	0.999996003
	IDP dMRI TBSS MD Genu of corpus callosum	Wald ratio	0.002602759	0.05	1
	IDP dMRI TBSS MD Body of corpus callosum	Wald ratio	0.025090923	0.05	1
	IDP dMRI TBSS MD Splenium of corpus callosum	Inverse variance weighted	0.462382952	0.05	1
	IDP dMRI TBSS MD Corticospinal tract R	Wald ratio	0.121957276	0.05	1
	IDP dMRI TBSS MD Inferior cerebellar peduncle R	Inverse variance weighted	0.570633701	0.05	1
	IDP dMRI TBSS MD Inferior cerebellar peduncle L	Wald ratio	0.215901514	0.05	1

	IDP dMRI TBSS MD Superior cerebellar peduncle R	Wald ratio	0.95647923	0.05	1
	IDP dMRI TBSS MD Superior cerebellar peduncle L	Wald ratio	0.894082364	0.05	1
	IDP dMRI TBSS MD Anterior limb of internal capsule L	Wald ratio	0.162141369	0.05	1
	IDP dMRI TBSS MD Retrolenticular part of internal capsule R	Inverse variance weighted	0.583496767	0.05	1
	IDP T1 FIRST left caudate volume plus IDP T1 FIRST right caudate volume	Wald ratio	0.416431779	0.05	1
	IDP T1 FIRST left putamen volume plus IDP T1 FIRST right putamen volume	Wald ratio	0.780285216	0.05	1
	IDP T1 FIRST left pallidum volume plus IDP T1 FIRST right pallidum volume	Wald ratio	0.497214336	0.05	1
	IDP SWI T2star left thalamus plus IDP SWI T2star right thalamus	MR Egger	0.284568853	0.05	1
		Weighted median	0.51309511	0.05	1
		Inverse variance weighted	0.156431744	0.05	1
		Simple mode	0.632730882	0.05	1
		Weighted mode	0.633135811	0.05	1
	IDP dMRI TBSS MD Retrolenticular part of internal capsule L	Inverse variance weighted	0.544313356	0.05	1
	IDP SWI T2star left caudate plus IDP SWI T2star right caudate	MR Egger	0.829509595	0.05	1
		Weighted median	0.441008559	0.05	1
		Inverse variance weighted	0.108215356	0.05	1
		Simple mode	0.744303464	0.05	1
		Weighted mode	0.71996607	0.05	1
	IDP SWI T2star left putamen plus IDP SWI T2star right putamen	MR Egger	0.491094278	0.05	1
		Weighted median	0.28527089	0.05	1

		Inverse variance weighted	0.140892018	0.05	1
		Simple mode	0.062206289	0.05	1
		Weighted mode	0.965952759	0.05	1
	IDP SWI T2star left pallidum plus IDP SWI T2star right pallidum	MR Egger	0.615469002	0.05	1
		Weighted median	0.469039601	0.05	1
		Inverse variance weighted	0.341050497	0.05	1
		Simple mode	0.536070668	0.05	1
		Weighted mode	0.643604014	0.05	1
	volume Left-Lateral-Ventricle	MR Egger	0.645162371	0.05	0.933936616
		Weighted median	0.977522094	0.05	1
		Inverse variance weighted	0.868774406	0.05	1
		Simple mode	0.926522748	0.05	1
		Weighted mode	0.936856981	0.05	1
	volume Left-Inf-Lat-Vent	Wald ratio	0.945832957	0.05	1
	volume Left-Cerebellum-White-Matter	Inverse variance weighted	0.095318609	0.05	1
	volume Left-Cerebellum-Cortex	Wald ratio	0.545115517	0.05	1
	IDP dMRI TBSS MD Anterior corona radiata R	Inverse variance weighted	0.238245664	0.05	1
	volume Left-Putamen	Wald ratio	0.557015598	0.05	1
	volume 3rd-Ventricle	Wald ratio	0.195910179	0.05	0.999696537
	volume 4th-Ventricle	Inverse variance weighted	0.726261288	0.05	1
	volume Brain-Stem	Inverse variance weighted	0.576192715	0.05	1

	volume Left-Hippocampus	Inverse variance weighted	0.750376227	0.05	1
	volume CSF	MR Egger	0.92977063	0.05	0.809704296
		Weighted median	0.816246346	0.05	1
		Inverse variance weighted	0.854486893	0.05	1
		Simple mode	0.80986353	0.05	1
		Weighted mode	0.799329581	0.05	1
	IDP dMRI TBSS MD Anterior corona radiata L	Inverse variance weighted	0.195991092	0.05	1
	volume Left-Accumbens-area	Wald ratio	0.64470385	0.05	1
	volume Right-Lateral-Ventricle	Inverse variance weighted	0.856887268	0.05	1
	volume Right-Inf-Lat-Vent	Wald ratio	0.728221485	0.05	1
	volume Right-Cerebellum-White-Matter	Wald ratio	0.403899359	0.05	1
	volume Right-Cerebellum-Cortex	MR Egger	0.83123691	0.05	1
		Weighted median	0.526511206	0.05	1
		Inverse variance weighted	0.613644027	0.05	1
		Simple mode	0.484332721	0.05	1
		Weighted mode	0.529789391	0.05	1
	IDP dMRI TBSS MD Superior corona radiata R	Inverse variance weighted	0.237398993	0.05	1
	volume Right-Hippocampus	Wald ratio	0.435245553	0.05	1
	volume Right-Amygdala	Wald ratio	0.208000091	0.05	0.999913231
	volume Right-VentralDC	Wald ratio	0.620150544	0.05	1
	volume CC Posterior	Wald ratio	0.862007128	0.05	1
	IDP dMRI TBSS MD Superior corona radiata L	Inverse variance weighted	0.232857404	0.05	1

	IDP dMRI TBSS MD Posterior corona radiata R	Inverse variance weighted	0.524385602	0.05	1
	volume BrainSegVol-to-eTIV	Wald ratio	0.185099059	0.05	1
	IDP dMRI TBSS MD Posterior corona radiata L	Inverse variance weighted	0.523553817	0.05	1
	volume rhSurfaceHoles	Wald ratio	0.927704279	0.05	1
	DKTatlas lh cuneus area	Wald ratio	0.830734832	0.05	1
	IDP dMRI TBSS MD Posterior thalamic radiation R	Inverse variance weighted	0.572532841	0.05	1
	DKTatlas lh lateraloccipital area	Wald ratio	0.830734832	0.05	1
	DKTatlas lh lingual area	Wald ratio	0.79143115	0.05	1
	IDP dMRI TBSS MD Posterior thalamic radiation L	Inverse variance weighted	0.548212815	0.05	1
	DKTatlas lh parstriangularis area	Wald ratio	0.493468241	0.05	1
	DKTatlas lh pericalcarine area	Inverse variance weighted	0.498521301	0.05	1
	DKTatlas lh postcentral area	Inverse variance weighted	0.275876458	0.05	1
	DKTatlas lh posteriorcingulate area	Wald ratio	0.390268558	0.05	1
	DKTatlas lh precentral area	Wald ratio	0.303870331	0.05	1
	DKTatlas lh precuneus area	Wald ratio	0.876592618	0.05	1
	IDP dMRI TBSS MD Sagittal stratum R	Inverse variance weighted	0.567724774	0.05	1
	DKTatlas lh superiorparietal area	Wald ratio	0.579323243	0.05	1
	DKTatlas lh superiortemporal area	Wald ratio	0.022390728	0.05	0.774998393
	DKTatlas lh supramarginal area	Wald ratio	0.440328244	0.05	1
	DKTatlas lh WhiteSurfArea area	Wald ratio	0.325635773	0.05	0.99999992
	IDP dMRI TBSS MD Sagittal stratum L	Inverse variance weighted	0.460685676	0.05	1

	a2009s lh G&S subcentral area	Wald ratio	0.303870331	0.05	1
	a2009s lh G cuneus area	Wald ratio	0.771358568	0.05	1
	a2009s lh G front inf-Opercular area	Wald ratio	0.690300272	0.05	1
	IDP dMRI TBSS MD External capsule R	Wald ratio	0.002602759	0.05	1
	IDP dMRI TBSS MD External capsule L	Inverse variance weighted	0.002998632	0.05	1
	a2009s lh G pariet inf-Supramar area	Wald ratio	0.440328244	0.05	1
	a2009s lh G parietal sup area	Wald ratio	0.260824526	0.05	1
	a2009s lh G postcentral area	Wald ratio	0.302750819	0.05	1
	a2009s lh G precentral area	Wald ratio	0.303870331	0.05	1
	a2009s lh G precuneus area	Wald ratio	0.771358568	0.05	1
	IDP dMRI TBSS MD Cingulum cingulate gyrus R	MR Egger	0.266293745	0.05	1
		Weighted median	0.010696177	0.05	1
		Inverse variance weighted	0.030694254	0.05	1
		Simple mode	0.233783035	0.05	1
		Weighted mode	0.12438387	0.05	1
	IDP dMRI TBSS MD Cingulum cingulate gyrus L	MR Egger	0.264799128	0.05	1
		Weighted median	0.013836655	0.05	1
		Inverse variance weighted	0.029827597	0.05	1
		Simple mode	0.231048622	0.05	1
		Weighted mode	0.117341498	0.05	1
	a2009s lh S calcarine area	Wald ratio	0.79143115	0.05	1
	a2009s lh S central area	Wald ratio	0.301966455	0.05	1
	a2009s lh S collat transv ant area	Wald ratio	0.863310822	0.05	1

	a2009s lh S front middle area	Wald ratio	0.194046458	0.05	0.999616436
	IDP dMRI TBSS MD Cingulum hippocampus R	Inverse variance weighted	0.467321146	0.05	1
	a2009s lh S intrapariet&P trans area	Wald ratio	0.260824526	0.05	1
	IDP dMRI TBSS MD Cingulum hippocampus L	Inverse variance weighted	0.448957794	0.05	1
	a2009s lh S subparietal area	Wald ratio	0.79143115	0.05	1
	DKTatlas rh cuneus area	Wald ratio	0.79143115	0.05	1
	DKTatlas rh lateraloccipital area	Inverse variance weighted	0.280290992	0.05	1
	DKTatlas rh lingual area	Wald ratio	0.786952335	0.05	1
	DKTatlas rh parstriangularis area	Wald ratio	0.934909381	0.05	1
	DKTatlas rh pericalcarine area	Inverse variance weighted	0.495785651	0.05	1
	IDP dMRI TBSS MD Superior longitudinal fasciculus R	Inverse variance weighted	0.590018156	0.05	1
	DKTatlas rh postcentral area	Wald ratio	0.301966455	0.05	0.999999994
	DKTatlas rh precentral area	Wald ratio	0.303870331	0.05	1
	IDP dMRI TBSS MD Superior longitudinal fasciculus L	Inverse variance weighted	0.585614172	0.05	1
	a2009s rh G&S subcentral area	Wald ratio	0.301966455	0.05	1
	a2009s rh G&S cingul-Mid-Post area	Wald ratio	0.410816127	0.05	1
	a2009s rh G cuneus area	Wald ratio	0.79143115	0.05	1
	IDP dMRI TBSS MD Superior fronto-occipital fasciculus L	Wald ratio	0.984263652	0.05	1
	a2009s rh G oc-temp med-Lingual area	Wald ratio	0.786952335	0.05	1
	a2009s rh G parietal sup area	Wald ratio	0.919811521	0.05	1
	IDP dMRI TBSS MD Uncinate fasciculus R	Inverse variance weighted	0.06432855	0.05	1

	a2009s rh G precentral area	Wald ratio	0.303870331	0.05	1
	a2009s rh G precuneus area	Wald ratio	0.802587349	0.05	1
	IDP dMRI TBSS MD Uncinate fasciculus L	Inverse variance weighted	0.001188223	0.05	1
	a2009s rh Pole occipital area	Wald ratio	0.79143115	0.05	1
	a2009s rh S calcarine area	Wald ratio	0.786952335	0.05	1
	a2009s rh S central area	Wald ratio	0.303870331	0.05	1
	a2009s rh S collat transv ant area	Wald ratio	0.863310822	0.05	1
	a2009s rh S orbital med-olfact area	Wald ratio	0.465865558	0.05	1
	IDP dMRI TBSS MO Pontine crossing tract	MR Egger	0.278065154	0.05	1
		Weighted median	0.082740756	0.05	1
		Inverse variance weighted	0.117899556	0.05	1
		Simple mode	0.27099336	0.05	1
		Weighted mode	0.261460768	0.05	1
	DKTatlas lh postcentral thickness	Wald ratio	0.301966455	0.05	1
	a2009s lh G insular short thickness	Wald ratio	0.784865176	0.05	1
	a2009s lh G postcentral thickness	Wald ratio	0.301966455	0.05	1
	IDP T1 SIENAX CSF normalised volume	Inverse variance weighted	0.716204982	0.05	1
	IDP dMRI TBSS MO Medial lemniscus R	Wald ratio	0.231426947	0.05	1
	a2009s lh S postcentral thickness	Wald ratio	0.561449635	0.05	1
	DKTatlas rh lateraloccipital thickness	Wald ratio	0.64470385	0.05	1
	DKTatlas rh parstriangularis thickness	Wald ratio	0.884796426	0.05	1
	DKTatlas rh postcentral thickness	Wald ratio	0.301966455	0.05	1

	DKTatlas rh posteriorcingulate thickness	Inverse variance weighted	0.408578956	0.05	1
	a2009s rh G&S cingul-Mid-Ant thickness	Wald ratio	0.383383566	0.05	1
	a2009s rh G cuneus thickness	Wald ratio	0.64470385	0.05	1
	a2009s rh G postcentral thickness	Wald ratio	0.420383901	0.05	1
	a2009s rh Pole occipital thickness	Wald ratio	0.64470385	0.05	1
	a2009s rh S circular insula ant thickness	Wald ratio	0.107026774	0.05	0.963657431
	a2009s rh S oc sup&transversal thickness	Wald ratio	0.734095612	0.05	1
	a2009s rh S parieto occipital thickness	Wald ratio	0.346606793	0.05	0.999999998
	a2009s rh S temporal transverse thickness	Wald ratio	0.165948934	0.05	0.999865202
	IDP dMRI TBSS MO Anterior corona radiata R	Wald ratio	0.601333425	0.05	1
	IDP dMRI TBSS MO Superior corona radiata R	Wald ratio	0.074405134	0.05	1
	IDP dMRI TBSS MO Cingulum cingulate gyrus L	Inverse variance weighted	0.503556217	0.05	1
	IDP dMRI TBSS L1 Genu of corpus callosum	Wald ratio	0.210732865	0.05	1
	IDP dMRI TBSS L1 Anterior limb of internal capsule L	Wald ratio	0.162141369	0.05	1
	IDP dMRI TBSS L1 Posterior limb of internal capsule R	Wald ratio	0.178840776	0.05	1
	IDP dMRI TBSS L1 Posterior limb of internal capsule L	Wald ratio	0.708857786	0.05	1
	IDP dMRI TBSS L1 Anterior corona radiata R	Wald ratio	0.002602759	0.05	1
	IDP dMRI TBSS L1 Anterior corona radiata L	Wald ratio	0.002602759	0.05	1
	IDP dMRI TBSS L1 Posterior corona radiata R	Wald ratio	0.006144474	0.05	1
	IDP dMRI TBSS L1 Posterior corona radiata L	Wald ratio	0.002602759	0.05	1

	IDP dMRI TBSS L1 Sagittal stratum L	Wald ratio	0.002602759	0.05	1
	IDP dMRI TBSS L1 External capsule R	Wald ratio	0.002602759	0.05	1
	IDP dMRI TBSS L1 External capsule L	MR Egger	0.67967565	0.05	1
		Weighted median	0.052467978	0.05	1
		Inverse variance weighted	0.160178993	0.05	1
		Simple mode	0.212569409	0.05	1
		Weighted mode	0.197844098	0.05	1
	IDP dMRI TBSS L1 Cingulum hippocampus L	Wald ratio	0.210732865	0.05	1
	IDP dMRI TBSS L1 Fornix cres+Stria terminalis L	Wald ratio	0.72908351	0.05	1
	IDP T1 FAST ROIs L precentral gyrus	Wald ratio	0.301966455	0.05	1
	IDP dMRI TBSS L1 Superior longitudinal fasciculus R	Inverse variance weighted	0.81431929	0.05	1
	IDP dMRI TBSS L1 Uncinate fasciculus R	Wald ratio	0.215901514	0.05	1
	IDP dMRI TBSS L1 Uncinate fasciculus L	Wald ratio	0.010353034	0.05	1
	IDP dMRI TBSS L2 Pontine crossing tract	Wald ratio	0.231426947	0.05	1
	IDP dMRI TBSS L2 Genu of corpus callosum	MR Egger	0.291793926	0.05	1
		Weighted median	0.043487349	0.05	1
		Inverse variance weighted	0.092756611	0.05	1
		Simple mode	0.318552922	0.05	1
		Weighted mode	0.218347709	0.05	1
	IDP dMRI TBSS L2 Body of corpus callosum	Wald ratio	0.002602759	0.05	1
	IDP dMRI TBSS L2 Splenium of corpus callosum	MR Egger	0.414722405	0.05	1
		Weighted median	0.640760899	0.05	1

		Inverse variance weighted	0.97218981	0.05	1
		Simple mode	0.677895464	0.05	1
		Weighted mode	0.641371117	0.05	1
	IDP dMRI TBSS L2 Corticospinal tract R	Wald ratio	0.121957276	0.05	1
	IDP T1 SIENAX CSF unnormalised volume	MR Egger	0.64597162	0.05	0.924763803
		Weighted median	0.985744874	0.05	1
		Inverse variance weighted	0.859159034	0.05	1
		Simple mode	0.93495696	0.05	1
		Weighted mode	0.941963839	0.05	1
	IDP T1 FAST ROIs L temporal pole	Wald ratio	0.5309693	0.05	1
	IDP dMRI TBSS L2 Superior cerebellar peduncle R	Inverse variance weighted	0.987129609	0.05	1
	IDP dMRI TBSS L2 Superior cerebellar peduncle L	Wald ratio	0.894082364	0.05	1
	IDP dMRI TBSS L2 Posterior limb of internal capsule R	Inverse variance weighted	0.63563828	0.05	1
	IDP dMRI TBSS L2 Posterior limb of internal capsule L	Wald ratio	0.69358381	0.05	1
	IDP dMRI TBSS L2 Retrolenticular part of internal capsule R	Inverse variance weighted	0.341706831	0.05	1
	IDP dMRI TBSS L2 Retrolenticular part of internal capsule L	Wald ratio	0.215901514	0.05	1
	IDP dMRI TBSS L2 Anterior corona radiata R	Inverse variance weighted	0.231592668	0.05	1
	IDP dMRI TBSS L2 Anterior corona radiata L	Wald ratio	0.002602759	0.05	1
	IDP dMRI TBSS L2 Superior corona radiata R	MR Egger	0.784831718	0.05	1
		Weighted median	0.591442947	0.05	1

		Inverse variance weighted	0.400293941	0.05	1
		Simple mode	0.66905073	0.05	1
		Weighted mode	0.756082081	0.05	1
	IDP dMRI TBSS L2 Superior corona radiata L	Inverse variance weighted	0.606184139	0.05	1
	IDP dMRI TBSS L2 Posterior corona radiata R	Inverse variance weighted	0.612937438	0.05	1
	IDP dMRI TBSS L2 Posterior corona radiata L	Inverse variance weighted	0.596355404	0.05	1
	IDP dMRI TBSS L2 Posterior thalamic radiation R	Inverse variance weighted	0.563840634	0.05	1
	IDP dMRI TBSS L2 Posterior thalamic radiation L	Inverse variance weighted	0.573807438	0.05	1
	IDP dMRI TBSS L2 Sagittal stratum R	Inverse variance weighted	0.578925514	0.05	1
	IDP dMRI TBSS L2 Sagittal stratum L	Wald ratio	0.210732865	0.05	1
	IDP dMRI TBSS L2 Cingulum cingulate gyrus R	Wald ratio	0.002602759	0.05	1
	IDP dMRI TBSS L2 Cingulum cingulate gyrus L	Wald ratio	0.210732865	0.05	1
	IDP dMRI TBSS L2 Cingulum hippocampus R	Inverse variance weighted	0.509405745	0.05	1
	IDP dMRI TBSS L2 Cingulum hippocampus L	Wald ratio	0.002602759	0.05	1
	IDP dMRI TBSS L2 Uncinate fasciculus L	Wald ratio	0.002602759	0.05	1
	IDP dMRI TBSS L3 Middle cerebellar peduncle	Wald ratio	0.215901514	0.05	1
	IDP dMRI TBSS L3 Genu of corpus callosum	Wald ratio	0.002602759	0.05	1
	IDP dMRI TBSS L3 Body of corpus callosum	Wald ratio	0.002602759	0.05	1
	IDP dMRI TBSS L3 Splenium of corpus callosum	Inverse variance weighted	0.800141227	0.05	1

	IDP dMRI TBSS L3 Inferior cerebellar peduncle R	Wald ratio	0.002602759	0.05	1
	IDP dMRI TBSS L3 Inferior cerebellar peduncle L	Wald ratio	0.002602759	0.05	1
	IDP dMRI TBSS L3 Superior cerebellar peduncle R	Inverse variance weighted	0.982821339	0.05	1
	IDP dMRI TBSS L3 Superior cerebellar peduncle L	Wald ratio	0.894082364	0.05	1
	IDP dMRI TBSS L3 Cerebral peduncle R	Wald ratio	0.999251164	0.05	1
	IDP dMRI TBSS L3 Anterior limb of internal capsule R	Wald ratio	0.297032742	0.05	1
	IDP dMRI TBSS L3 Anterior limb of internal capsule L	Wald ratio	0.588967469	0.05	1
	IDP dMRI TBSS L3 Retrolenticular part of internal capsule R	Inverse variance weighted	0.567041902	0.05	1
	IDP dMRI TBSS L3 Retrolenticular part of internal capsule L	Wald ratio	0.215901514	0.05	1
	IDP dMRI TBSS L3 Anterior corona radiata R	Inverse variance weighted	0.233676659	0.05	1
	IDP dMRI TBSS L3 Anterior corona radiata L	Inverse variance weighted	0.186367895	0.05	1
	IDP dMRI TBSS L3 Superior corona radiata R	Wald ratio	0.210732865	0.05	1
	IDP dMRI TBSS L3 Superior corona radiata L	Wald ratio	0.210732865	0.05	1
	IDP dMRI TBSS L3 Posterior corona radiata R	Inverse variance weighted	0.308246325	0.05	1
	IDP dMRI TBSS L3 Posterior corona radiata L	Wald ratio	0.215901514	0.05	1
	IDP dMRI TBSS L3 Posterior thalamic radiation R	Inverse variance weighted	0.504933751	0.05	1
	IDP dMRI TBSS L3 Posterior thalamic radiation L	Inverse variance weighted	0.521730258	0.05	1
	IDP dMRI TBSS L3 Sagittal stratum R	Inverse variance weighted	0.525249622	0.05	1

	IDP dMRI TBSS L3 Sagittal stratum L	Inverse variance weighted	0.439799705	0.05	1
	IDP dMRI TBSS L3 External capsule R	Wald ratio	0.002602759	0.05	1
	IDP dMRI TBSS L3 External capsule L	Wald ratio	0.002602759	0.05	1
	IDP dMRI TBSS L3 Cingulum cingulate gyrus R	Inverse variance weighted	0.201257453	0.05	1
	IDP dMRI TBSS L3 Cingulum cingulate gyrus L	Wald ratio	0.002602759	0.05	1
	IDP dMRI TBSS L3 Cingulum hippocampus R	Wald ratio	0.002602759	0.05	1
	IDP dMRI TBSS L3 Cingulum hippocampus L	Wald ratio	0.002602759	0.05	1
	IDP dMRI TBSS L3 Fornix cres+Stria terminalis R	Wald ratio	0.315503639	0.05	1
	IDP dMRI TBSS L3 Superior longitudinal fasciculus R	Inverse variance weighted	0.577622496	0.05	1
	IDP dMRI TBSS L3 Superior longitudinal fasciculus L	Inverse variance weighted	0.570933295	0.05	1
	IDP dMRI TBSS L3 Uncinate fasciculus R	Wald ratio	0.002602759	0.05	1
	IDP dMRI TBSS L3 Uncinate fasciculus L	Inverse variance weighted	0.000317073	0.05	1
	IDP dMRI TBSS ICVF Middle cerebellar peduncle	Inverse variance weighted	0.462866383	0.05	1
	IDP dMRI TBSS ICVF Genu of corpus callosum	MR Egger	0.173829783	0.05	0.967178823
		Weighted median	0.384993633	0.05	1
		Inverse variance weighted	0.21386601	0.05	1
		Simple mode	0.774408748	0.05	1
		Weighted mode	0.840054367	0.05	1
	IDP dMRI TBSS ICVF Body of corpus callosum	MR Egger	0.542379906	0.05	1
		Weighted median	0.824305481	0.05	1

		Inverse variance weighted	0.952838537	0.05	1
		Simple mode	0.614716907	0.05	1
		Weighted mode	0.999647037	0.05	1
	IDP dMRI TBSS ICVF Splenium of corpus callosum	MR Egger	0.698060768	0.05	1
		Weighted median	0.471623315	0.05	1
		Inverse variance weighted	0.817568491	0.05	1
		Simple mode	0.331577504	0.05	1
		Weighted mode	0.450095215	0.05	1
	IDP dMRI TBSS ICVF Fornix	Wald ratio	0.984263652	0.05	1
	IDP dMRI TBSS ICVF Medial lemniscus R	Inverse variance weighted	0.191857268	0.05	1
	IDP dMRI TBSS ICVF Medial lemniscus L	Wald ratio	0.364315322	0.05	1
	IDP dMRI TBSS ICVF Inferior cerebellar peduncle R	MR Egger	0.776008122	0.05	1
		Weighted median	0.946272788	0.05	1
		Inverse variance weighted	0.690243546	0.05	1
		Simple mode	0.780820426	0.05	1
		Weighted mode	0.792257238	0.05	1
	IDP dMRI TBSS ICVF Inferior cerebellar peduncle L	MR Egger	0.835542824	0.05	1
		Weighted median	0.896646676	0.05	1
		Inverse variance weighted	0.722471961	0.05	1
		Simple mode	0.797277932	0.05	1
		Weighted mode	0.794457465	0.05	1
		MR Egger	0.76240666	0.05	0.999955836

	IDP dMRI TBSS ICVF Superior cerebellar peduncle R	Weighted median	0.499811739	0.05	1
		Inverse variance weighted	0.348325194	0.05	1
		Simple mode	0.782686388	0.05	1
		Weighted mode	0.852315879	0.05	1
	IDP dMRI TBSS ICVF Superior cerebellar peduncle L	Inverse variance weighted	0.436697127	0.05	1
	IDP dMRI TBSS ICVF Cerebral peduncle R	MR Egger	0.88414785	0.05	1
		Weighted median	0.712468967	0.05	1
		Inverse variance weighted	0.861234639	0.05	1
		Simple mode	0.732711518	0.05	1
		Weighted mode	0.682543531	0.05	1
	IDP dMRI TBSS ICVF Cerebral peduncle L	Inverse variance weighted	0.48788836	0.05	1
	IDP dMRI TBSS ICVF Anterior limb of internal capsule R	Wald ratio	0.002602759	0.05	0.99999888
	IDP dMRI TBSS ICVF Anterior limb of internal capsule L	MR Egger	0.155074762	0.05	0.022542971
		Weighted median	0.022483208	0.05	1
		Inverse variance weighted	0.003908531	0.05	1
		Simple mode	0.312391783	0.05	1
		Weighted mode	0.041032925	0.05	0.999999246
	IDP dMRI TBSS ICVF Posterior limb of internal capsule R	MR Egger	0.4096888	0.05	0.999183529
		Weighted median	0.376553136	0.05	1
		Inverse variance weighted	0.308763777	0.05	1
		Simple mode	0.308480802	0.05	1
		Weighted mode	0.932162636	0.05	1

	IDP dMRI TBSS ICVF Posterior limb of internal capsule L	MR Egger	0.294449999	0.05	0.831207077
		Weighted median	0.009714327	0.05	1
		Inverse variance weighted	0.022282651	0.05	1
		Simple mode	0.222176234	0.05	1
		Weighted mode	0.110912306	0.05	0.999999999
	IDP dMRI TBSS ICVF Retrolenticular part of internal capsule R	MR Egger	0.163986712	0.05	0.937514365
		Weighted median	0.012979705	0.05	1
		Inverse variance weighted	0.489328039	0.05	1
		Simple mode	0.469120871	0.05	1
		Weighted mode	0.104057565	0.05	1
	IDP dMRI TBSS ICVF Retrolenticular part of internal capsule L	MR Egger	0.374076551	0.05	0.999991339
		Weighted median	0.001577638	0.05	1
		Inverse variance weighted	0.014759735	0.05	1
		Simple mode	0.199955719	0.05	1
		Weighted mode	0.114806357	0.05	1
	IDP dMRI TBSS ICVF Anterior corona radiata R	MR Egger	0.391207974	0.05	0.996246014
		Weighted median	0.004120796	0.05	1
		Inverse variance weighted	0.026853835	0.05	1
		Simple mode	0.253808744	0.05	1
		Weighted mode	0.107164514	0.05	1
	IDP dMRI TBSS ICVF Anterior corona radiata L	MR Egger	0.343615779	0.05	0.997725802
		Weighted median	0.004223766	0.05	1

		Inverse variance weighted	0.016304699	0.05	1
		Simple mode	0.223974698	0.05	1
		Weighted mode	0.092440102	0.05	1
	IDP dMRI TBSS ICVF Superior corona radiata R	MR Egger	0.446697795	0.05	0.99543995
		Weighted median	0.004968914	0.05	1
		Inverse variance weighted	0.037966187	0.05	1
		Simple mode	0.227723077	0.05	1
		Weighted mode	0.113429048	0.05	1
	IDP dMRI TBSS ICVF Superior corona radiata L	MR Egger	0.411406019	0.05	0.993216867
		Weighted median	0.006011607	0.05	1
		Inverse variance weighted	0.030347093	0.05	1
		Simple mode	0.244635996	0.05	1
		Weighted mode	0.10375969	0.05	1
	IDP dMRI TBSS ICVF Posterior corona radiata R	MR Egger	0.26585181	0.05	0.502743837
		Weighted median	0.054503954	0.05	1
		Inverse variance weighted	0.056994904	0.05	1
		Simple mode	0.698524488	0.05	1
		Weighted mode	0.122756036	0.05	0.999999999
	IDP dMRI TBSS ICVF Posterior corona radiata L	MR Egger	0.273452059	0.05	0.84884786
		Weighted median	0.039945792	0.05	1
		Inverse variance weighted	0.048515778	0.05	1
		Simple mode	0.674655536	0.05	1

		Weighted mode	0.106500801	0.05	1
	IDP dMRI TBSS ICVF Posterior thalamic radiation R	Inverse variance weighted	0.462209783	0.05	1
	IDP dMRI TBSS ICVF Posterior thalamic radiation L	MR Egger	0.600789577	0.05	1
		Weighted median	0.538434337	0.05	1
		Inverse variance weighted	0.386586549	0.05	1
		Simple mode	0.665873748	0.05	1
		Weighted mode	0.84082217	0.05	1
	IDP dMRI TBSS ICVF Sagittal stratum R	MR Egger	0.434449153	0.05	0.999999095
		Weighted median	0.874266964	0.05	1
		Inverse variance weighted	0.591761571	0.05	1
		Simple mode	0.9378503	0.05	1
		Weighted mode	0.985391924	0.05	1
	IDP dMRI TBSS ICVF Sagittal stratum L	MR Egger	0.507556625	0.05	1
		Weighted median	0.751993718	0.05	1
		Inverse variance weighted	0.427070292	0.05	1
		Simple mode	0.928011103	0.05	1
		Weighted mode	0.965889503	0.05	1
	IDP dMRI TBSS ICVF External capsule R	MR Egger	0.270553411	0.05	0.999615892
		Weighted median	0.001484499	0.05	1
		Inverse variance weighted	0.002047548	0.05	1
		Simple mode	0.168898487	0.05	1
		Weighted mode	0.091330746	0.05	1

	IDP dMRI TBSS ICVF External capsule L	Inverse variance weighted	0.000968098	0.05	1
	IDP dMRI TBSS ICVF Cingulum cingulate gyrus R	Inverse variance weighted	0.090517947	0.05	1
	IDP dMRI TBSS ICVF Cingulum cingulate gyrus L	Inverse variance weighted	0.363920401	0.05	1
	IDP dMRI TBSS ICVF Cingulum hippocampus R	MR Egger	0.554932828	0.05	1
		Weighted median	0.003692464	0.05	1
		Inverse variance weighted	0.013616175	0.05	1
		Simple mode	0.136236811	0.05	1
		Weighted mode	0.109623972	0.05	1
	IDP dMRI TBSS ICVF Cingulum hippocampus L	MR Egger	0.341816004	0.05	0.999999607
		Weighted median	0.001577781	0.05	1
		Inverse variance weighted	0.007496049	0.05	1
		Simple mode	0.192523699	0.05	1
		Weighted mode	0.092890432	0.05	1
	IDP dMRI TBSS ICVF Fornix cres+Stria terminalis R	Inverse variance weighted	0.718739328	0.05	1
	IDP dMRI TBSS ICVF Fornix cres+Stria terminalis L	Wald ratio	0.210732865	0.05	1
	IDP dMRI TBSS ICVF Superior longitudinal fasciculus R	MR Egger	0.883690427	0.05	1
		Weighted median	0.170321986	0.05	1
		Inverse variance weighted	0.253720602	0.05	1
		Simple mode	0.301480706	0.05	1
		Weighted mode	0.935193518	0.05	1
		MR Egger	0.509340309	0.05	0.999990625

	IDP dMRI TBSS ICVF Superior longitudinal fasciculus L	Weighted median	0.005079965	0.05	1
		Inverse variance weighted	0.044847344	0.05	1
		Simple mode	0.220965587	0.05	1
		Weighted mode	0.102733603	0.05	1
	IDP dMRI TBSS ICVF Superior fronto-occipital fasciculus R	Inverse variance weighted	0.044275018	0.05	0.999999944
	IDP dMRI TBSS ICVF Superior fronto-occipital fasciculus L	Wald ratio	0.210732865	0.05	0.999999989
	IDP dMRI TBSS ICVF Uncinate fasciculus R	Wald ratio	0.002602759	0.05	1
	IDP dMRI TBSS ICVF Uncinate fasciculus L	Wald ratio	0.002602759	0.05	0.999999881
	IDP dMRI TBSS ICVF Tapetum R	Wald ratio	0.215901514	0.05	0.999999995
	IDP dMRI TBSS ICVF Tapetum L	Wald ratio	0.215901514	0.05	0.999999977
	IDP dMRI TBSS OD Pontine crossing tract	MR Egger	0.778336289	0.05	1
		Weighted median	0.198458426	0.05	1
		Inverse variance weighted	0.131669511	0.05	1
		Simple mode	0.478068073	0.05	1
		Weighted mode	0.343655052	0.05	1
	IDP dMRI TBSS OD Genu of corpus callosum	Wald ratio	0.629082339	0.05	1
	IDP dMRI TBSS OD Cerebral peduncle R	Wald ratio	0.946931183	0.05	1
	IDP dMRI TBSS OD Cerebral peduncle L	Wald ratio	0.946931183	0.05	1
	IDP dMRI TBSS OD Posterior limb of internal capsule L	Wald ratio	0.708857786	0.05	1
	IDP dMRI TBSS OD Posterior corona radiata R	Wald ratio	0.406938671	0.05	1
	IDP dMRI TBSS OD External capsule R	Inverse variance weighted	0.398118712	0.05	1

	IDP dMRI TBSS OD External capsule L	Wald ratio	0.955983267	0.05	1
	IDP dMRI TBSS OD Superior longitudinal fasciculus R	Wald ratio	0.150764629	0.05	1
	IDP dMRI TBSS ISOVF Fornix	Wald ratio	0.580942709	0.05	1
	IDP dMRI TBSS ISOVF External capsule R	Wald ratio	0.098575119	0.05	0.999997188
	IDP dMRI TBSS ISOVF Cingulum cingulate gyrus R	Inverse variance weighted	0.605197121	0.05	1
	IDP dMRI ProbtrackX FA ar l	Wald ratio	0.002602759	0.05	0.974979259
	IDP dMRI ProbtrackX FA atr l	Wald ratio	0.002602759	0.05	0.999880636
	IDP dMRI ProbtrackX FA atr r	Wald ratio	0.215901514	0.05	0.999999997
	IDP dMRI ProbtrackX FA fma	Wald ratio	0.215901514	0.05	0.999999879
	IDP dMRI ProbtrackX FA fmi	MR Egger	0.141072967	0.05	0.21124578
		Weighted median	0.072795203	0.05	1
		Inverse variance weighted	0.071684143	0.05	1
		Simple mode	0.979677692	0.05	1
		Weighted mode	0.131519747	0.05	0.999999997
	IDP dMRI ProbtrackX FA ifo l	Inverse variance weighted	0.457410199	0.05	1
	IDP dMRI ProbtrackX FA ifo r	Inverse variance weighted	0.502962571	0.05	1
	IDP dMRI ProbtrackX FA ilf l	Inverse variance weighted	0.30093088	0.05	1
	IDP dMRI ProbtrackX FA ilf r	Inverse variance weighted	0.30329922	0.05	1
	IDP dMRI ProbtrackX FA ml l	Wald ratio	0.276402952	0.05	1
	IDP dMRI ProbtrackX FA ptr l	Wald ratio	0.210732865	0.05	1
	IDP dMRI ProbtrackX FA ptr r	Wald ratio	0.015467135	0.05	1

	IDP dMRI ProbtrackX FA slf l	Inverse variance weighted	0.527108273	0.05	1
	IDP dMRI ProbtrackX FA slf r	Wald ratio	0.215901514	0.05	0.999999981
	IDP dMRI ProbtrackX FA str l	Wald ratio	0.824140896	0.05	1
	IDP dMRI ProbtrackX FA unc l	Wald ratio	0.002602759	0.05	0.99486791
	IDP dMRI ProbtrackX FA unc r	Wald ratio	0.002602759	0.05	0.9994153
	IDP dMRI ProbtrackX MD ar l	Wald ratio	0.002602759	0.05	1
	IDP dMRI ProbtrackX MD ar r	Wald ratio	0.002602759	0.05	1
	IDP dMRI ProbtrackX MD atr l	Wald ratio	0.002602759	0.05	1
	IDP dMRI ProbtrackX MD atr r	Wald ratio	0.002602759	0.05	1
	IDP dMRI ProbtrackX MD cgc l	MR Egger	0.26239732	0.05	1
		Weighted median	0.009966483	0.05	1
		Inverse variance weighted	0.017061374	0.05	1
		Simple mode	0.214359773	0.05	1
		Weighted mode	0.114790507	0.05	1
	IDP dMRI ProbtrackX MD cgc r	MR Egger	0.425831156	0.05	1
		Weighted median	0.136572082	0.05	1
		Inverse variance weighted	0.154850151	0.05	1
		Simple mode	0.272086825	0.05	1
		Weighted mode	0.293344694	0.05	1
	IDP dMRI ProbtrackX MD fmi	Inverse variance weighted	0.199467419	0.05	1
	IDP dMRI ProbtrackX MD ifo l	Inverse variance weighted	0.512109145	0.05	1
	IDP dMRI ProbtrackX MD ifo r	Inverse variance weighted	0.530405303	0.05	1

	IDP dMRI ProbtrackX MD ilf l	Inverse variance weighted	0.516412314	0.05	1
	IDP dMRI ProbtrackX MD ilf r	Inverse variance weighted	0.471265625	0.05	1
	IDP dMRI ProbtrackX MD ptr l	Wald ratio	0.002602759	0.05	1
	IDP dMRI ProbtrackX MD ptr r	Wald ratio	0.002602759	0.05	1
	IDP dMRI ProbtrackX MD slf l	Inverse variance weighted	0.225972846	0.05	1
	IDP dMRI ProbtrackX MD slf r	Inverse variance weighted	0.556315694	0.05	1
	IDP dMRI ProbtrackX MD str l	Wald ratio	0.142747549	0.05	1
	IDP dMRI ProbtrackX MD str r	Wald ratio	0.002602759	0.05	1
	IDP dMRI ProbtrackX MD unc l	Wald ratio	0.002602759	0.05	1
	IDP dMRI ProbtrackX MD unc r	Inverse variance weighted	0.507135319	0.05	1
	IDP dMRI ProbtrackX MO atr l	Wald ratio	0.139738269	0.05	0.999999826
	IDP dMRI ProbtrackX MO ml r	Wald ratio	0.50250112	0.05	1
	IDP T1 FAST ROIs L intracalc cortex	Wald ratio	0.79143115	0.05	1
	IDP dMRI ProbtrackX L1 fmi	Wald ratio	0.210732865	0.05	1
	IDP dMRI ProbtrackX L1 ifo l	Wald ratio	0.210732865	0.05	1
	IDP dMRI ProbtrackX L1 ifo r	Wald ratio	0.210732865	0.05	1
	IDP dMRI ProbtrackX L1 ilf l	Wald ratio	0.002602759	0.05	1
	IDP dMRI ProbtrackX L1 ilf r	Wald ratio	0.210732865	0.05	1
	IDP T1 FAST ROIs R intracalc cortex	Wald ratio	0.79143115	0.05	1
	IDP dMRI ProbtrackX L1 slf l	Wald ratio	0.002602759	0.05	1
	IDP dMRI ProbtrackX L1 slf r	Inverse variance weighted	0.863957411	0.05	1
	IDP dMRI ProbtrackX L1 str r	Wald ratio	0.93907452	0.05	1

	IDP dMRI ProbtrackX L1 unc l	Inverse variance weighted	0.001493147	0.05	1
	IDP dMRI ProbtrackX L1 unc r	Inverse variance weighted	0.931017843	0.05	1
	IDP dMRI ProbtrackX L2 ar l	Wald ratio	0.002602759	0.05	1
	IDP dMRI ProbtrackX L2 ar r	Wald ratio	0.002602759	0.05	1
	IDP dMRI ProbtrackX L2 atr l	Wald ratio	0.002602759	0.05	1
	IDP dMRI ProbtrackX L2 atr r	Wald ratio	0.002602759	0.05	1
	IDP dMRI ProbtrackX L2 cgc l	Wald ratio	0.210732865	0.05	1
	IDP dMRI ProbtrackX L2 cgc r	Wald ratio	0.210732865	0.05	1
	IDP dMRI ProbtrackX L2 cgh l	Wald ratio	0.615650434	0.05	1
	IDP dMRI ProbtrackX L2 fma	Wald ratio	0.576237553	0.05	1
	IDP dMRI ProbtrackX L2 fmi	Inverse variance weighted	0.20180645	0.05	1
	IDP dMRI ProbtrackX L2 ifo l	Inverse variance weighted	0.527802192	0.05	1
	IDP dMRI ProbtrackX L2 ifo r	Inverse variance weighted	0.555474936	0.05	1
	IDP dMRI ProbtrackX L2 ilf l	Inverse variance weighted	0.512222718	0.05	1
	IDP dMRI ProbtrackX L2 ilf r	Inverse variance weighted	0.484327725	0.05	1
	IDP dMRI ProbtrackX L2 ptr l	Wald ratio	0.002602759	0.05	1
	IDP dMRI ProbtrackX L2 ptr r	Wald ratio	0.002432625	0.05	1
	IDP dMRI ProbtrackX L2 slf l	Inverse variance weighted	0.232826054	0.05	1
	IDP dMRI ProbtrackX L2 slf r	Inverse variance weighted	0.583331222	0.05	1
	IDP dMRI ProbtrackX L2 str l	Wald ratio	0.142747549	0.05	1
	IDP dMRI ProbtrackX L2 str r	Wald ratio	0.142747549	0.05	1

	IDP dMRI ProbtrackX L2 unc l	Wald ratio	0.002602759	0.05	1
	IDP dMRI ProbtrackX L2 unc r	Inverse variance weighted	0.488559595	0.05	1
	IDP dMRI ProbtrackX L3 ar l	Wald ratio	0.002602759	0.05	1
	IDP dMRI ProbtrackX L3 ar r	Wald ratio	0.002602759	0.05	1
	IDP dMRI ProbtrackX L3 atr l	Wald ratio	0.002602759	0.05	1
	IDP dMRI ProbtrackX L3 atr r	Wald ratio	0.002602759	0.05	1
	IDP dMRI ProbtrackX L3 cgc l	Wald ratio	0.215901514	0.05	1
	IDP dMRI ProbtrackX L3 cgc r	Wald ratio	0.215901514	0.05	1
	IDP dMRI ProbtrackX L3 fmi	MR Egger	0.413223581	0.05	1
		Weighted median	0.012475093	0.05	1
		Inverse variance weighted	0.044434044	0.05	1
		Simple mode	0.264126597	0.05	1
		Weighted mode	0.120985965	0.05	1
	IDP dMRI ProbtrackX L3 ifo l	Inverse variance weighted	0.159219044	0.05	1
	IDP dMRI ProbtrackX L3 ifo r	Inverse variance weighted	0.498644285	0.05	1
	IDP dMRI ProbtrackX L3 ilf l	Inverse variance weighted	0.479440534	0.05	1
	IDP dMRI ProbtrackX L3 ilf r	Inverse variance weighted	0.444859427	0.05	1
	IDP dMRI ProbtrackX L3 ptr l	Wald ratio	0.002602759	0.05	1
	IDP dMRI ProbtrackX L3 ptr r	Wald ratio	0.002432625	0.05	1
	IDP dMRI ProbtrackX L3 slf l	Inverse variance weighted	0.213066112	0.05	1
	IDP dMRI ProbtrackX L3 slf r	Inverse variance weighted	0.536880714	0.05	1

	IDP dMRI ProbtrackX L3 str l	Wald ratio	0.002432625	0.05	1
	IDP dMRI ProbtrackX L3 str r	Wald ratio	0.210732865	0.05	1
	IDP dMRI ProbtrackX L3 unc l	Wald ratio	0.002602759	0.05	1
	IDP dMRI ProbtrackX L3 unc r	Inverse variance weighted	0.538223025	0.05	1
	IDP dMRI ProbtrackX ICVF ar l	MR Egger	0.281348583	0.05	0.999999512
		Weighted median	0.002270785	0.05	1
		Inverse variance weighted	0.003170112	0.05	1
		Simple mode	0.205276355	0.05	1
		Weighted mode	0.097809012	0.05	1
	IDP dMRI ProbtrackX ICVF ar r	MR Egger	0.286041547	0.05	0.999995575
		Weighted median	0.002933041	0.05	1
		Inverse variance weighted	0.003514043	0.05	1
		Simple mode	0.207195733	0.05	1
		Weighted mode	0.094335096	0.05	1
	IDP dMRI ProbtrackX ICVF atr l	MR Egger	0.286645235	0.05	0.999455195
		Weighted median	0.001431608	0.05	1
		Inverse variance weighted	0.005983629	0.05	1
		Simple mode	0.190780293	0.05	1
		Weighted mode	0.103315649	0.05	1
	IDP dMRI ProbtrackX ICVF atr r	MR Egger	0.284643789	0.05	0.997176288
		Weighted median	0.002936133	0.05	1
		Inverse variance weighted	0.007723708	0.05	1

		Simple mode	0.202141697	0.05	1
		Weighted mode	0.106556373	0.05	1
	IDP dMRI ProbtrackX ICVF cgc l	Inverse variance weighted	0.37796289	0.05	1
	IDP dMRI ProbtrackX ICVF cgc r	Wald ratio	0.210732865	0.05	1
	IDP dMRI ProbtrackX ICVF cgh l	MR Egger	0.284230069	0.05	0.999982992
		Weighted median	0.002614317	0.05	1
		Inverse variance weighted	0.005200365	0.05	1
		Simple mode	0.192142201	0.05	1
		Weighted mode	0.09725217	0.05	1
	IDP dMRI ProbtrackX ICVF cgh r	Wald ratio	0.002602759	0.05	1
	IDP dMRI ProbtrackX ICVF cst l	MR Egger	0.285164078	0.05	0.99177695
		Weighted median	0.004434408	0.05	1
		Inverse variance weighted	0.00874505	0.05	1
		Simple mode	0.21179943	0.05	1
		Weighted mode	0.094634075	0.05	1
	IDP dMRI ProbtrackX ICVF cst r	MR Egger	0.170754	0.05	0.987253861
		Weighted median	0.012538714	0.05	1
		Inverse variance weighted	0.008900028	0.05	1
		Simple mode	0.33540604	0.05	1
		Weighted mode	0.065443571	0.05	1
	IDP dMRI ProbtrackX ICVF fma	MR Egger	0.430489521	0.05	0.999444464
		Weighted median	0.948599958	0.05	1

		Inverse variance weighted	0.653386079	0.05	1
		Simple mode	0.819332607	0.05	1
		Weighted mode	0.818593706	0.05	1
	IDP dMRI ProbtrackX ICVF fmi	MR Egger	0.168503551	0.05	0.997891339
		Weighted median	0.008047361	0.05	1
		Inverse variance weighted	0.05961489	0.05	1
		Simple mode	0.934873357	0.05	1
		Weighted mode	0.076624906	0.05	1
	IDP dMRI ProbtrackX ICVF ifo l	Inverse variance weighted	0.109777935	0.05	1
	IDP dMRI ProbtrackX ICVF ifo r	Inverse variance weighted	0.114072717	0.05	1
	IDP dMRI ProbtrackX ICVF ilf l	Inverse variance weighted	0.127721811	0.05	1
	IDP dMRI ProbtrackX ICVF ilf r	Inverse variance weighted	0.429574649	0.05	1
	IDP dMRI ProbtrackX ICVF mcp	Inverse variance weighted	0.443358031	0.05	1
	IDP dMRI ProbtrackX ICVF ml l	Wald ratio	0.010353034	0.05	1
	IDP dMRI ProbtrackX ICVF ml r	Wald ratio	0.002602759	0.05	0.999998
	IDP dMRI ProbtrackX ICVF ptr l	Inverse variance weighted	0.419353086	0.05	1
	IDP dMRI ProbtrackX ICVF ptr r	MR Egger	0.763975254	0.05	0.795825734
		Weighted median	0.548673807	0.05	1
		Inverse variance weighted	0.734809068	0.05	1
		Simple mode	0.516705876	0.05	1
		Weighted mode	0.478687952	0.05	1

	IDP dMRI ProbtrackX ICVF slf l	MR Egger	0.415310235	0.05	0.999941056
		Weighted median	0.003122102	0.05	1
		Inverse variance weighted	0.026464718	0.05	1
		Simple mode	0.209846216	0.05	1
		Weighted mode	0.103210097	0.05	1
	IDP dMRI ProbtrackX ICVF slf r	MR Egger	0.772491959	0.05	1
		Weighted median	0.192252235	0.05	1
		Inverse variance weighted	0.231161589	0.05	1
		Simple mode	0.27935555	0.05	1
		Weighted mode	0.299247192	0.05	1
	IDP dMRI ProbtrackX ICVF str l	MR Egger	0.315204612	0.05	0.958650431
		Weighted median	0.006588508	0.05	1
		Inverse variance weighted	0.020564195	0.05	1
		Simple mode	0.23771065	0.05	1
		Weighted mode	0.110146729	0.05	1
	IDP dMRI ProbtrackX ICVF str r	MR Egger	0.377556434	0.05	0.983363388
		Weighted median	0.004999753	0.05	1
		Inverse variance weighted	0.025786989	0.05	1
		Simple mode	0.239561999	0.05	1
		Weighted mode	0.101524117	0.05	0.999999999
	IDP dMRI ProbtrackX ICVF unc l	Inverse variance weighted	0.001331102	0.05	1
	IDP dMRI ProbtrackX ICVF unc r	Inverse variance weighted	0.050347462	0.05	1

	IDP dMRI ProbtrackX OD atr r	Wald ratio	0.717234962	0.05	1
	IDP dMRI ProbtrackX OD ilf l	Wald ratio	0.687911205	0.05	1
	IDP dMRI ProbtrackX OD slf l	Wald ratio	0.098182111	0.05	1
	IDP dMRI ProbtrackX OD str l	Inverse variance weighted	0.763488063	0.05	1
	IDP dMRI ProbtrackX ISOVF atr r	Wald ratio	0.002602759	0.05	0.936532177
	IDP dMRI ProbtrackX ISOVF cgc l	Wald ratio	0.74521767	0.05	1
	IDP dMRI ProbtrackX ISOVF ifo r	Inverse variance weighted	0.143043083	0.05	1
	IDP dMRI ProbtrackX ISOVF ilf l	Wald ratio	0.582460511	0.05	1
	IDP dMRI ProbtrackX ISOVF ilf r	MR Egger	0.931821661	0.05	1
		Weighted median	0.814233379	0.05	1
		Inverse variance weighted	0.60179391	0.05	1
		Simple mode	0.976784105	0.05	1
		Weighted mode	0.985856075	0.05	1
	IDP dMRI ProbtrackX ISOVF slf l	Inverse variance weighted	0.710036455	0.05	1
	IDP dMRI ProbtrackX ISOVF slf r	MR Egger	0.910287723	0.05	1
		Weighted median	0.241125	0.05	1
		Inverse variance weighted	0.115993569	0.05	1
		Simple mode	0.48786361	0.05	1
		Weighted mode	0.48315795	0.05	1
	IDP dMRI ProbtrackX ISOVF unc l	Wald ratio	0.175670207	0.05	1
	IDP dMRI ProbtrackX ISOVF unc r	Wald ratio	0.161396376	0.05	1
	IDP T1 SIENAX brain-normalised volume	Wald ratio	0.667053941	0.05	1

Systemic sclerosis	IDP T1 SIENAX peripheral grey normalised volume	Wald ratio	0.78863381	0.05	1
	IDP T1 FAST ROIs R temp occ fusif cortex	Wald ratio	0.286097268	0.05	0.658099744
	IDP T1 FAST ROIs R occ pole	Inverse variance weighted	0.462222812	0.05	1
	IDP T1 FAST ROIs L thalamus	Inverse variance weighted	0.892910674	0.05	1
	IDP T1 FAST ROIs R thalamus	Wald ratio	0.886129163	0.05	1
	IDP T1 FAST ROIs L putamen	Wald ratio	0.317447311	0.05	1
	IDP T1 FAST ROIs R putamen	Wald ratio	0.317447311	0.05	1
	IDP T1 FAST ROIs L pallidum	Wald ratio	0.592906146	0.05	1
	IDP T1 FIRST left caudate volume	Wald ratio	0.76074189	0.05	1
	IDP T1 FAST ROIs L hippocampus	Inverse variance weighted	0.18357336	0.05	0.999992573
	IDP T1 FAST ROIs R hippocampus	Wald ratio	0.260890116	0.05	0.99990179
	IDP T1 FAST ROIs L ventral striatum	Wald ratio	0.317447311	0.05	1
	IDP T1 FAST ROIs R ventral striatum	Inverse variance weighted	0.918225533	0.05	1
	IDP T1 FAST ROIs L cerebellum VI	Wald ratio	0.486708795	0.05	0.999999999
	IDP T1 FAST ROIs L cerebellum crus I	MR Egger	0.476784644	0.05	0.999999991
		Weighted median	0.170043479	0.05	1
		Inverse variance weighted	0.149280168	0.05	1
		Simple mode	0.508101105	0.05	1
		Weighted mode	0.270836231	0.05	1
	IDP T1 FAST ROIs R cerebellum crus I	Inverse variance weighted	0.196256378	0.05	0.999999999
	IDP T1 FAST ROIs L cerebellum crus II	MR Egger	0.92476077	0.05	1
		Weighted median	0.393973492	0.05	1

		Inverse variance weighted	0.232040003	0.05	1
		Simple mode	0.547606093	0.05	1
		Weighted mode	0.563967632	0.05	1
	IDP T1 FAST ROIs V cerebellum crus II	Inverse variance weighted	0.698213203	0.05	1
	IDP T1 FAST ROIs R cerebellum crus II	Inverse variance weighted	0.432590717	0.05	1
	IDP T1 FIRST left putamen volume	Inverse variance weighted	0.692301279	0.05	1
	IDP T1 FAST ROIs R cerebellum VIIb	Wald ratio	0.631772719	0.05	1
	IDP T1 FAST ROIs V cerebellum VIIIa	MR Egger	0.813874768	0.05	0.897818938
		Weighted median	0.392028302	0.05	1
		Inverse variance weighted	0.355160929	0.05	1
		Simple mode	0.516637056	0.05	1
		Weighted mode	0.511379452	0.05	1
	IDP T1 FAST ROIs L cerebellum VIIIb	Wald ratio	0.773421397	0.05	1
	IDP T1 FAST ROIs V cerebellum VIIIb	Inverse variance weighted	0.395542668	0.05	1
	IDP T1 FAST ROIs R cerebellum VIIIb	Wald ratio	0.317447311	0.05	1
	IDP T1 FAST ROIs L cerebellum IX	Inverse variance weighted	0.637474484	0.05	1
	IDP T1 FIRST right putamen volume	Wald ratio	0.695684514	0.05	1
	IDP T1 FAST ROIs V cerebellum IX	Inverse variance weighted	0.921685387	0.05	1
	IDP T1 FAST ROIs R cerebellum IX	Inverse variance weighted	0.706198372	0.05	1
	IDP T1 FAST ROIs V cerebellum X	Wald ratio	0.317447311	0.05	1
	IDP T2 FLAIR BIANCA WMH volume	Inverse variance weighted	0.503989649	0.05	1

	IDP SWI T2star left caudate	MR Egger	0.187050432	0.05	0.010394689
		Weighted median	0.025970636	0.05	1
		Inverse variance weighted	0.024806089	0.05	1
		Simple mode	0.114553019	0.05	1
		Weighted mode	0.10860796	0.05	1
	IDP SWI T2star right caudate	MR Egger	0.847451028	0.05	1
		Weighted median	0.152961642	0.05	1
		Inverse variance weighted	0.02303285	0.05	1
		Simple mode	0.332005337	0.05	1
		Weighted mode	0.29091368	0.05	1
	IDP T1 FIRST left pallidum volume	Wald ratio	0.795673911	0.05	1
	IDP SWI T2star left putamen	MR Egger	0.414142196	0.05	0.999996998
		Weighted median	0.043523313	0.05	1
		Inverse variance weighted	0.006584347	0.05	1
		Simple mode	0.196860721	0.05	1
		Weighted mode	0.161233714	0.05	1
	IDP SWI T2star right putamen	MR Egger	0.93618761	0.05	1
		Weighted median	0.038686178	0.05	1
		Inverse variance weighted	0.00769033	0.05	1
		Simple mode	0.120534511	0.05	1
		Weighted mode	0.158597369	0.05	1
	IDP SWI T2star left pallidum	MR Egger	0.517073219	0.05	1

		Weighted median	0.500114423	0.05	1
		Inverse variance weighted	0.711596776	0.05	1
		Simple mode	0.658954092	0.05	1
		Weighted mode	0.569470716	0.05	1
	IDP SWI T2star right pallidum	MR Egger	0.384967241	0.05	0.999375298
		Weighted median	0.841466888	0.05	1
		Inverse variance weighted	0.686544633	0.05	1
		Simple mode	0.836112124	0.05	1
		Weighted mode	0.828343528	0.05	1
	IDP T1 FIRST right pallidum volume	Wald ratio	0.795673911	0.05	1
	IDP dMRI TBSS FA Genu of corpus callosum	Wald ratio	0.244071339	0.05	0.999999967
	IDP dMRI TBSS FA Splenium of corpus callosum	Inverse variance weighted	0.580080508	0.05	1
	IDP dMRI TBSS FA Corticospinal tract L	Wald ratio	0.97373934	0.05	1
	IDP dMRI TBSS FA Superior cerebellar peduncle R	Inverse variance weighted	0.778981118	0.05	1
	IDP dMRI TBSS FA Superior cerebellar peduncle L	Inverse variance weighted	0.782940306	0.05	1
	IDP dMRI TBSS FA Posterior limb of internal capsule R	Wald ratio	0.035547529	0.05	0.789480361
	IDP dMRI TBSS FA Retrolenticular part of internal capsule R	Inverse variance weighted	0.469869776	0.05	1
	IDP dMRI TBSS FA Anterior corona radiata R	Wald ratio	0.244071339	0.05	0.999999875
	IDP dMRI TBSS FA Anterior corona radiata L	Wald ratio	0.244071339	0.05	0.999999751
	IDP dMRI TBSS FA Posterior thalamic radiation R	Wald ratio	0.714679022	0.05	1

	IDP dMRI TBSS FA Posterior thalamic radiation L	Wald ratio	0.715518687	0.05	1
	IDP dMRI TBSS FA Sagittal stratum R	Wald ratio	0.657642799	0.05	1
	IDP dMRI TBSS FA Cingulum cingulate gyrus R	Inverse variance weighted	0.555785846	0.05	1
	IDP dMRI TBSS FA Superior longitudinal fasciculus L	Inverse variance weighted	0.925085617	0.05	1
	IDP dMRI TBSS FA Uncinate fasciculus L	Wald ratio	0.740315871	0.05	1
	IDP dMRI TBSS MD Genu of corpus callosum	Wald ratio	0.244071339	0.05	1
	IDP dMRI TBSS MD Body of corpus callosum	Wald ratio	0.117873094	0.05	0.999686909
	IDP dMRI TBSS MD Splenium of corpus callosum	Inverse variance weighted	0.521613181	0.05	1
	IDP dMRI TBSS MD Corticospinal tract R	Wald ratio	0.03156967	0.05	0.552406358
	IDP dMRI TBSS MD Superior cerebellar peduncle R	Wald ratio	0.853681973	0.05	1
	IDP dMRI TBSS MD Superior cerebellar peduncle L	Wald ratio	0.859491677	0.05	1
	IDP dMRI TBSS MD Anterior limb of internal capsule L	Wald ratio	0.975906478	0.05	1
	IDP dMRI TBSS MD Retrolenticular part of internal capsule R	Wald ratio	0.716358696	0.05	1
	IDP T1 FIRST left caudate volume plus IDP T1 FIRST right caudate volume	Wald ratio	0.898600227	0.05	1
	IDP T1 FIRST left putamen volume plus IDP T1 FIRST right putamen volume	Inverse variance weighted	0.855484637	0.05	1
	IDP T1 FIRST left pallidum volume plus IDP T1 FIRST right pallidum volume	Wald ratio	0.795673911	0.05	1
	IDP SWI T2star left thalamus plus IDP SWI T2star right thalamus	MR Egger	0.580740958	0.05	1
		Weighted median	0.074026723	0.05	1
		Inverse variance weighted	0.127079523	0.05	1
		Simple mode	0.214635088	0.05	1

		Weighted mode	0.217932222	0.05	1
	IDP dMRI TBSS MD Retrolenticular part of internal capsule L	Wald ratio	0.716358696	0.05	1
	IDP SWI T2star left caudate plus IDP SWI T2star right caudate	MR Egger	0.695997186	0.05	1
		Weighted median	0.129742528	0.05	1
		Inverse variance weighted	0.029971563	0.05	1
		Simple mode	0.28637823	0.05	1
		Weighted mode	0.211296059	0.05	1
	IDP SWI T2star left putamen plus IDP SWI T2star right putamen	MR Egger	0.576305153	0.05	1
		Weighted median	0.080116426	0.05	1
		Inverse variance weighted	0.020758715	0.05	1
		Simple mode	0.996250578	0.05	1
		Weighted mode	0.996289283	0.05	1
	IDP SWI T2star left pallidum plus IDP SWI T2star right pallidum	MR Egger	0.443093899	0.05	1
		Weighted median	0.876253293	0.05	1
		Inverse variance weighted	0.564237643	0.05	1
		Simple mode	0.823664232	0.05	1
		Weighted mode	0.818359376	0.05	1
	volume Left-Lateral-Ventricle	MR Egger	0.893988779	0.05	1
		Weighted median	0.589804848	0.05	1
		Inverse variance weighted	0.933313054	0.05	1
		Simple mode	0.648728737	0.05	1
		Weighted mode	0.63810138	0.05	1

	volume Left-Cerebellum-White-Matter	Inverse variance weighted	0.886470994	0.05	1
	volume Left-Cerebellum-Cortex	Inverse variance weighted	0.153182926	0.05	1
	IDP dMRI TBSS MD Anterior corona radiata R	Inverse variance weighted	0.479342042	0.05	1
	volume Left-Putamen	Inverse variance weighted	0.453148238	0.05	1
	volume 4th-Ventricle	Inverse variance weighted	0.253911746	0.05	1
	volume Brain-Stem	Wald ratio	0.618322614	0.05	1
	volume Left-Hippocampus	Inverse variance weighted	0.07861981	0.05	0.94439635
	volume CSF	MR Egger	0.531077293	0.05	0.00254261
		Weighted median	0.149923985	0.05	0.999949836
		Inverse variance weighted	0.305095462	0.05	1
		Simple mode	0.350339036	0.05	0.999816571
		Weighted mode	0.347600094	0.05	0.999816571
	IDP dMRI TBSS MD Anterior corona radiata L	Inverse variance weighted	0.452821824	0.05	1
	volume Left-Accumbens-area	Wald ratio	0.317447311	0.05	1
	volume Right-Lateral-Ventricle	Inverse variance weighted	0.547841411	0.05	1
	volume Right-Inf-Lat-Vent	Wald ratio	0.90948601	0.05	1
	volume Right-Cerebellum-White-Matter	Wald ratio	0.352236458	0.05	1
	volume Right-Cerebellum-Cortex	MR Egger	0.568120396	0.05	0.999997489
		Weighted median	0.139311466	0.05	0.999999989
		Inverse variance weighted	0.113873037	0.05	1
		Simple mode	0.27554332	0.05	0.999984236

		Weighted mode	0.298564393	0.05	0.999999698
	IDP dMRI TBSS MD Superior corona radiata R	Inverse variance weighted	0.478799648	0.05	1
	volume Right-Putamen	Wald ratio	0.695684514	0.05	1
	volume Right-Hippocampus	Wald ratio	0.260890116	0.05	0.998241856
	volume Right-Amygdala	Wald ratio	0.732918177	0.05	1
	volume Right-VentralDC	Wald ratio	0.279031653	0.05	1
	volume CC Posterior	Wald ratio	0.316729894	0.05	1
	IDP dMRI TBSS MD Superior corona radiata L	Inverse variance weighted	0.475898645	0.05	1
	IDP dMRI TBSS MD Posterior corona radiata R	Wald ratio	0.716358696	0.05	1
	volume BrainSegVol-to-eTIV	Wald ratio	0.202373974	0.05	1
	IDP dMRI TBSS MD Posterior corona radiata L	Wald ratio	0.715518687	0.05	1
	volume rhSurfaceHoles	Wald ratio	0.30880605	0.05	1
	DKTatlas lh cuneus area	Wald ratio	0.686461488	0.05	1
	IDP dMRI TBSS MD Posterior thalamic radiation R	Wald ratio	0.715518687	0.05	1
	DKTatlas lh lateraloccipital area	Wald ratio	0.686461488	0.05	1
	DKTatlas lh lingual area	Wald ratio	0.58746958	0.05	1
	IDP dMRI TBSS MD Posterior thalamic radiation L	Wald ratio	0.716358696	0.05	1
	DKTatlas lh parstriangularis area	Wald ratio	0.814707664	0.05	1
	DKTatlas lh pericalcarine area	Inverse variance weighted	0.341602288	0.05	1
	DKTatlas lh postcentral area	Inverse variance weighted	0.761463027	0.05	1
	DKTatlas lh posteriorcingulate area	Wald ratio	0.140364722	0.05	0.260141399

	DKTatlas lh precentral area	Wald ratio	0.927363755	0.05	1
	DKTatlas lh precuneus area	Wald ratio	0.773869132	0.05	1
	IDP dMRI TBSS MD Sagittal stratum R	Wald ratio	0.716358696	0.05	1
	DKTatlas lh superiorparietal area	Wald ratio	0.596461139	0.05	1
	DKTatlas lh superiortemporal area	Wald ratio	0.682277893	0.05	1
	DKTatlas lh supramarginal area	Wald ratio	0.956101919	0.05	1
	DKTatlas lh WhiteSurfArea area	Wald ratio	0.969695499	0.05	1
	IDP dMRI TBSS MD Sagittal stratum L	Wald ratio	0.716358696	0.05	1
	a2009s lh G&S subcentral area	Wald ratio	0.927363755	0.05	1
	a2009s lh G cuneus area	Wald ratio	0.748968331	0.05	1
	a2009s lh G front inf-Opercular area	Wald ratio	0.907364354	0.05	1
	IDP dMRI TBSS MD External capsule R	Wald ratio	0.244071339	0.05	1
	IDP dMRI TBSS MD External capsule L	Inverse variance weighted	0.344854587	0.05	1
	a2009s lh G pariet inf-Supramar area	Wald ratio	0.956101919	0.05	1
	a2009s lh G parietal sup area	Wald ratio	0.595962299	0.05	1
	a2009s lh G postcentral area	Wald ratio	0.595198784	0.05	1
	a2009s lh G precentral area	Wald ratio	0.927363755	0.05	1
	a2009s lh G precuneus area	Wald ratio	0.748968331	0.05	1
	IDP dMRI TBSS MD Cingulum cingulate gyrus R	MR Egger	0.466145321	0.05	1
		Weighted median	0.527478775	0.05	1
		Inverse variance weighted	0.482900275	0.05	1
		Simple mode	0.92908865	0.05	1
		Weighted mode	0.393981336	0.05	1

	IDP dMRI TBSS MD Cingulum cingulate gyrus L	MR Egger	0.466971998	0.05	1
		Weighted median	0.526796761	0.05	1
		Inverse variance weighted	0.482515187	0.05	1
		Simple mode	0.925114792	0.05	1
		Weighted mode	0.368114656	0.05	1
	a2009s lh S calcarine area	Wald ratio	0.58746958	0.05	1
	a2009s lh S central area	Wald ratio	0.931788382	0.05	1
	a2009s lh S collat transv ant area	Wald ratio	0.27096227	0.05	1
	a2009s lh S front middle area	Wald ratio	0.425246112	0.05	1
	IDP dMRI TBSS MD Cingulum hippocampus R	Wald ratio	0.716358696	0.05	1
	a2009s lh S intrapariet&P trans area	Wald ratio	0.595962299	0.05	1
	IDP dMRI TBSS MD Cingulum hippocampus L	Wald ratio	0.716358696	0.05	1
	a2009s lh S subparietal area	Wald ratio	0.58746958	0.05	1
	DKTatlas rh cuneus area	Wald ratio	0.58746958	0.05	1
	DKTatlas rh lingual area	Wald ratio	0.585660091	0.05	1
	DKTatlas rh parstriangularis area	Wald ratio	0.846333701	0.05	1
	DKTatlas rh pericalcarine area	Inverse variance weighted	0.554604672	0.05	1
	IDP dMRI TBSS MD Superior longitudinal fasciculus R	Wald ratio	0.716358696	0.05	1
	DKTatlas rh postcentral area	Wald ratio	0.931788382	0.05	1
	DKTatlas rh precentral area	Wald ratio	0.927363755	0.05	1
	IDP dMRI TBSS MD Superior longitudinal fasciculus L	Wald ratio	0.716358696	0.05	1
	a2009s rh G&S subcentral area	Wald ratio	0.931788382	0.05	1

	a2009s rh G&S cingul-Mid-Post area	Wald ratio	0.35671975	0.05	0.999999412
	a2009s rh G cuneus area	Wald ratio	0.58746958	0.05	1
	IDP dMRI TBSS MD Superior fronto-occipital fasciculus L	Wald ratio	0.693298002	0.05	1
	a2009s rh G oc-temp med-Lingual area	Wald ratio	0.585660091	0.05	1
	a2009s rh G parietal sup area	Wald ratio	0.156915904	0.05	0.928604017
	IDP dMRI TBSS MD Uncinate fasciculus R	Wald ratio	0.965872734	0.05	1
	a2009s rh G precentral area	Wald ratio	0.927363755	0.05	1
	a2009s rh G precuneus area	Wald ratio	0.598050007	0.05	1
	IDP dMRI TBSS MD Uncinate fasciculus L	Inverse variance weighted	0.31122279	0.05	1
	a2009s rh Pole occipital area	Wald ratio	0.58746958	0.05	1
	a2009s rh S calcarine area	Wald ratio	0.585660091	0.05	1
	a2009s rh S central area	Wald ratio	0.927363755	0.05	1
	a2009s rh S collat transv ant area	Wald ratio	0.27096227	0.05	1
	a2009s rh S orbital med-olfact area	Wald ratio	0.841052846	0.05	1
	IDP dMRI TBSS MO Pontine crossing tract	MR Egger	0.403468866	0.05	0.999994815
		Weighted median	0.571954403	0.05	1
		Inverse variance weighted	0.685670856	0.05	1
		Simple mode	0.602174657	0.05	1
		Weighted mode	0.546422369	0.05	1
	DKTatlas lh postcentral thickness	Wald ratio	0.931788382	0.05	1
	a2009s lh G insular short thickness	Wald ratio	0.509708256	0.05	1
	a2009s lh G postcentral thickness	Wald ratio	0.931788382	0.05	1

	IDP T1 SIENAX CSF normalised volume	Inverse variance weighted	0.754318178	0.05	1
	IDP dMRI TBSS MO Medial lemniscus R	Wald ratio	0.992740605	0.05	1
	a2009s lh S postcentral thickness	Wald ratio	0.968308782	0.05	1
	DKTatlas rh lateraloccipital thickness	Wald ratio	0.317447311	0.05	0.341371665
	DKTatlas rh parstriangularis thickness	Wald ratio	0.000191977	0.05	0.006075049
	DKTatlas rh postcentral thickness	Wald ratio	0.931788382	0.05	1
	DKTatlas rh posteriorcingulate thickness	Inverse variance weighted	0.29399892	0.05	1
	a2009s rh G&S cingul-Mid-Ant thickness	Wald ratio	0.22168861	0.05	0.998882684
	a2009s rh G cuneus thickness	Wald ratio	0.317447311	0.05	0.571970589
	a2009s rh G postcentral thickness	Wald ratio	0.931788382	0.05	1
	a2009s rh Pole occipital thickness	Wald ratio	0.317447311	0.05	0.608511435
	a2009s rh S oc sup&transversal thickness	Wald ratio	0.380302602	0.05	1
	a2009s rh S parieto occipital thickness	Wald ratio	0.32875212	0.05	1
	IDP dMRI TBSS MO Anterior corona radiata R	Wald ratio	0.460211403	0.05	1
	IDP dMRI TBSS MO Superior corona radiata R	Wald ratio	0.394341809	0.05	1
	IDP dMRI TBSS MO Cingulum cingulate gyrus L	Inverse variance weighted	0.267409062	0.05	1
	IDP dMRI TBSS L1 Anterior limb of internal capsule L	Wald ratio	0.975906478	0.05	1
	IDP dMRI TBSS L1 Posterior limb of internal capsule R	Wald ratio	0.431832326	0.05	1
	IDP dMRI TBSS L1 Posterior limb of internal capsule L	Wald ratio	0.458197503	0.05	1
	IDP dMRI TBSS L1 Anterior corona radiata R	Wald ratio	0.244071339	0.05	1
	IDP dMRI TBSS L1 Anterior corona radiata L	Wald ratio	0.244071339	0.05	1

	IDP dMRI TBSS L1 Posterior corona radiata R	Wald ratio	0.417760262	0.05	1
	IDP dMRI TBSS L1 Posterior corona radiata L	Wald ratio	0.244071339	0.05	1
	IDP dMRI TBSS L1 Sagittal stratum L	Wald ratio	0.244071339	0.05	1
	IDP dMRI TBSS L1 External capsule R	Wald ratio	0.244071339	0.05	1
	IDP dMRI TBSS L1 External capsule L	Inverse variance weighted	0.255142026	0.05	1
	IDP dMRI TBSS L1 Fornix cres+Stria terminalis L	Wald ratio	0.673170314	0.05	1
	IDP T1 FAST ROIs L precentral gyrus	Wald ratio	0.931788382	0.05	1
	IDP dMRI TBSS L1 Superior longitudinal fasciculus R	Inverse variance weighted	0.814697429	0.05	1
	IDP dMRI TBSS L1 Uncinate fasciculus L	Wald ratio	0.117974212	0.05	1
	IDP dMRI TBSS L2 Pontine crossing tract	Wald ratio	0.992740605	0.05	1
	IDP dMRI TBSS L2 Genu of corpus callosum	MR Egger	0.481727839	0.05	1
		Weighted median	0.268487019	0.05	1
		Inverse variance weighted	0.499393707	0.05	1
		Simple mode	0.471534476	0.05	1
		Weighted mode	0.372569579	0.05	1
	IDP dMRI TBSS L2 Body of corpus callosum	Wald ratio	0.244071339	0.05	1
	IDP dMRI TBSS L2 Splenium of corpus callosum	MR Egger	0.615276359	0.05	0.017514534
		Weighted median	0.519772049	0.05	1
		Inverse variance weighted	0.522970087	0.05	1
		Simple mode	0.63530473	0.05	1
		Weighted mode	0.566745912	0.05	1

	IDP dMRI TBSS L2 Corticospinal tract R	Wald ratio	0.03156967	0.05	0.523373407
	IDP T1 SIENAX CSF unnormalised volume	MR Egger	0.996906892	0.05	1
		Weighted median	0.674524056	0.05	1
		Inverse variance weighted	0.951941378	0.05	1
		Simple mode	0.636130862	0.05	1
		Weighted mode	0.61813349	0.05	1
	IDP T1 FAST ROIs L temporal pole	Wald ratio	0.7935511	0.05	1
	IDP dMRI TBSS L2 Superior cerebellar peduncle R	Inverse variance weighted	0.777578702	0.05	1
	IDP dMRI TBSS L2 Superior cerebellar peduncle L	Wald ratio	0.859491677	0.05	1
	IDP dMRI TBSS L2 Posterior limb of internal capsule R	Inverse variance weighted	0.669366491	0.05	1
	IDP dMRI TBSS L2 Posterior limb of internal capsule L	Wald ratio	0.411472081	0.05	1
	IDP dMRI TBSS L2 Retrolenticular part of internal capsule R	Inverse variance weighted	0.574311165	0.05	1
	IDP dMRI TBSS L2 Anterior corona radiata R	Inverse variance weighted	0.475093268	0.05	1
	IDP dMRI TBSS L2 Anterior corona radiata L	Wald ratio	0.244071339	0.05	1
	IDP dMRI TBSS L2 Superior corona radiata R	Inverse variance weighted	0.953167598	0.05	1
	IDP dMRI TBSS L2 Superior corona radiata L	Wald ratio	0.716358696	0.05	1
	IDP dMRI TBSS L2 Posterior corona radiata R	Wald ratio	0.715518687	0.05	1
	IDP dMRI TBSS L2 Posterior corona radiata L	Wald ratio	0.715518687	0.05	1
	IDP dMRI TBSS L2 Posterior thalamic radiation R	Wald ratio	0.714679022	0.05	1
	IDP dMRI TBSS L2 Posterior thalamic radiation L	Wald ratio	0.715518687	0.05	1

	IDP dMRI TBSS L2 Sagittal stratum R	Wald ratio	0.716358696	0.05	1
	IDP dMRI TBSS L2 Cingulum cingulate gyrus R	Wald ratio	0.244071339	0.05	1
	IDP dMRI TBSS L2 Cingulum hippocampus R	Wald ratio	0.716358696	0.05	1
	IDP dMRI TBSS L2 Cingulum hippocampus L	Wald ratio	0.244071339	0.05	1
	IDP dMRI TBSS L2 Uncinate fasciculus L	Wald ratio	0.244071339	0.05	1
	IDP dMRI TBSS L3 Genu of corpus callosum	Wald ratio	0.244071339	0.05	1
	IDP dMRI TBSS L3 Body of corpus callosum	Wald ratio	0.244071339	0.05	1
	IDP dMRI TBSS L3 Splenium of corpus callosum	Wald ratio	0.41474127	0.05	1
	IDP dMRI TBSS L3 Inferior cerebellar peduncle R	Wald ratio	0.244071339	0.05	1
	IDP dMRI TBSS L3 Inferior cerebellar peduncle L	Wald ratio	0.244071339	0.05	1
	IDP dMRI TBSS L3 Superior cerebellar peduncle R	Inverse variance weighted	0.779237005	0.05	1
	IDP dMRI TBSS L3 Superior cerebellar peduncle L	Wald ratio	0.859491677	0.05	1
	IDP dMRI TBSS L3 Cerebral peduncle R	Wald ratio	0.662748203	0.05	1
	IDP dMRI TBSS L3 Anterior limb of internal capsule R	Wald ratio	0.840741091	0.05	1
	IDP dMRI TBSS L3 Anterior limb of internal capsule L	Wald ratio	0.493550594	0.05	1
	IDP dMRI TBSS L3 Retrolenticular part of internal capsule R	Wald ratio	0.716358696	0.05	1
	IDP dMRI TBSS L3 Anterior corona radiata R	Inverse variance weighted	0.476420916	0.05	1
	IDP dMRI TBSS L3 Anterior corona radiata L	Inverse variance weighted	0.446915317	0.05	1
	IDP dMRI TBSS L3 Posterior corona radiata R	Inverse variance weighted	0.197525145	0.05	1

	IDP dMRI TBSS L3 Posterior thalamic radiation R	Wald ratio	0.714679022	0.05	1
	IDP dMRI TBSS L3 Posterior thalamic radiation L	Wald ratio	0.716358696	0.05	1
	IDP dMRI TBSS L3 Sagittal stratum R	Wald ratio	0.716358696	0.05	1
	IDP dMRI TBSS L3 Sagittal stratum L	Wald ratio	0.716358696	0.05	1
	IDP dMRI TBSS L3 External capsule R	Wald ratio	0.244071339	0.05	1
	IDP dMRI TBSS L3 External capsule L	Wald ratio	0.244071339	0.05	1
	IDP dMRI TBSS L3 Cingulum cingulate gyrus R	Inverse variance weighted	0.456072272	0.05	1
	IDP dMRI TBSS L3 Cingulum cingulate gyrus L	Wald ratio	0.244071339	0.05	1
	IDP dMRI TBSS L3 Cingulum hippocampus R	Wald ratio	0.244071339	0.05	1
	IDP dMRI TBSS L3 Cingulum hippocampus L	Wald ratio	0.244071339	0.05	1
	IDP dMRI TBSS L3 Fornix cres+Stria terminalis R	Wald ratio	0.966127412	0.05	1
	IDP dMRI TBSS L3 Superior longitudinal fasciculus R	Wald ratio	0.716358696	0.05	1
	IDP dMRI TBSS L3 Superior longitudinal fasciculus L	Wald ratio	0.716358696	0.05	1
	IDP dMRI TBSS L3 Uncinate fasciculus R	Wald ratio	0.244071339	0.05	1
	IDP dMRI TBSS L3 Uncinate fasciculus L	Inverse variance weighted	0.265950133	0.05	1
	IDP dMRI TBSS ICVF Middle cerebellar peduncle	Wald ratio	0.716358696	0.05	1
	IDP dMRI TBSS ICVF Genu of corpus callosum	MR Egger	0.339544607	0.05	0.994377396
		Weighted median	0.72326179	0.05	1
		Inverse variance weighted	0.981117314	0.05	1
		Simple mode	0.953468748	0.05	1

		Weighted mode	0.572612896	0.05	1
	IDP dMRI TBSS ICVF Body of corpus callosum	MR Egger	0.478079692	0.05	0.889558006
		Weighted median	0.841691561	0.05	1
		Inverse variance weighted	0.648942606	0.05	1
		Simple mode	0.775038894	0.05	1
		Weighted mode	0.629659104	0.05	1
	IDP dMRI TBSS ICVF Splenium of corpus callosum	MR Egger	0.986125978	0.05	1
		Weighted median	0.834425432	0.05	1
		Inverse variance weighted	0.672529627	0.05	1
		Simple mode	0.750890724	0.05	1
		Weighted mode	0.725320768	0.05	1
	IDP dMRI TBSS ICVF Fornix	Wald ratio	0.693298002	0.05	1
	IDP dMRI TBSS ICVF Medial lemniscus R	Inverse variance weighted	0.851871497	0.05	1
	IDP dMRI TBSS ICVF Medial lemniscus L	Wald ratio	0.293967754	0.05	1
	IDP dMRI TBSS ICVF Inferior cerebellar peduncle R	Inverse variance weighted	0.449860195	0.05	1
	IDP dMRI TBSS ICVF Inferior cerebellar peduncle L	Inverse variance weighted	0.461091528	0.05	1
	IDP dMRI TBSS ICVF Superior cerebellar peduncle R	MR Egger	0.747269826	0.05	0.999999971
		Weighted median	0.84887072	0.05	1
		Inverse variance weighted	0.669081325	0.05	1
		Simple mode	0.72824937	0.05	1
		Weighted mode	0.785289195	0.05	1
	IDP dMRI TBSS ICVF Superior cerebellar peduncle L	Inverse variance weighted	0.561540838	0.05	1

	IDP dMRI TBSS ICVF Cerebral peduncle R	MR Egger	0.807684994	0.05	0.998907442
		Weighted median	0.119422936	0.05	1
		Inverse variance weighted	0.33913722	0.05	1
		Simple mode	0.308655602	0.05	1
		Weighted mode	0.162102041	0.05	1
	IDP dMRI TBSS ICVF Cerebral peduncle L	Wald ratio	0.773421397	0.05	1
	IDP dMRI TBSS ICVF Anterior limb of internal capsule R	Wald ratio	0.244071339	0.05	1
	IDP dMRI TBSS ICVF Anterior limb of internal capsule L	MR Egger	0.487252871	0.05	0.093122605
		Weighted median	0.251922955	0.05	1
		Inverse variance weighted	0.509274181	0.05	1
		Simple mode	0.65077914	0.05	1
		Weighted mode	0.378383137	0.05	1
	IDP dMRI TBSS ICVF Posterior limb of internal capsule R	MR Egger	0.76311177	0.05	1
		Weighted median	0.617168665	0.05	1
		Inverse variance weighted	0.737768706	0.05	1
		Simple mode	0.568862387	0.05	1
		Weighted mode	0.592386923	0.05	1
	IDP dMRI TBSS ICVF Posterior limb of internal capsule L	MR Egger	0.560885574	0.05	0.999647823
		Weighted median	0.189848312	0.05	1
		Inverse variance weighted	0.325916619	0.05	1
		Simple mode	0.447593099	0.05	1
		Weighted mode	0.357295391	0.05	1

	IDP dMRI TBSS ICVF Retrolenticular part of internal capsule R	MR Egger	0.411104212	0.05	0.999925593
		Weighted median	0.328926923	0.05	1
		Inverse variance weighted	0.797944836	0.05	1
		Simple mode	0.540307663	0.05	1
		Weighted mode	0.352878315	0.05	1
	IDP dMRI TBSS ICVF Retrolenticular part of internal capsule L	MR Egger	0.598416685	0.05	1
		Weighted median	0.25355726	0.05	1
		Inverse variance weighted	0.321807039	0.05	1
		Simple mode	0.424363179	0.05	1
		Weighted mode	0.345323042	0.05	1
	IDP dMRI TBSS ICVF Anterior corona radiata R	MR Egger	0.607567644	0.05	0.999999994
		Weighted median	0.180648916	0.05	1
		Inverse variance weighted	0.337248653	0.05	1
		Simple mode	0.405755319	0.05	1
		Weighted mode	0.338501583	0.05	1
	IDP dMRI TBSS ICVF Anterior corona radiata L	MR Egger	0.583110663	0.05	0.999999991
		Weighted median	0.232526619	0.05	1
		Inverse variance weighted	0.321641786	0.05	1
		Simple mode	0.42243631	0.05	1
		Weighted mode	0.35874246	0.05	1
	IDP dMRI TBSS ICVF Superior corona radiata R	MR Egger	0.64002605	0.05	0.999999998
		Weighted median	0.152669649	0.05	1

		Inverse variance weighted	0.351608624	0.05	1
		Simple mode	0.445161373	0.05	1
		Weighted mode	0.340226671	0.05	1
	IDP dMRI TBSS ICVF Superior corona radiata L	MR Egger	0.618875434	0.05	0.999999988
		Weighted median	0.170119655	0.05	1
		Inverse variance weighted	0.342159131	0.05	1
		Simple mode	0.425724959	0.05	1
		Weighted mode	0.346928301	0.05	1
	IDP dMRI TBSS ICVF Posterior corona radiata R	MR Egger	0.506522272	0.05	0.95834856
		Weighted median	0.426461178	0.05	1
		Inverse variance weighted	0.429450324	0.05	1
		Simple mode	0.780691474	0.05	1
		Weighted mode	0.38583446	0.05	1
	IDP dMRI TBSS ICVF Posterior corona radiata L	MR Egger	0.521014699	0.05	0.998794996
		Weighted median	0.399503964	0.05	1
		Inverse variance weighted	0.422248486	0.05	1
		Simple mode	0.732948541	0.05	1
		Weighted mode	0.392276698	0.05	1
	IDP dMRI TBSS ICVF Posterior thalamic radiation R	Wald ratio	0.716358696	0.05	1
	IDP dMRI TBSS ICVF Posterior thalamic radiation L	Inverse variance weighted	0.842098624	0.05	1
	IDP dMRI TBSS ICVF Sagittal stratum R	MR Egger	0.815165655	0.05	1
		Weighted median	0.887134254	0.05	1

		Inverse variance weighted	0.914987958	0.05	1
		Simple mode	0.723264609	0.05	1
		Weighted mode	0.822335766	0.05	1
	IDP dMRI TBSS ICVF Sagittal stratum L	Inverse variance weighted	0.883215258	0.05	1
	IDP dMRI TBSS ICVF External capsule R	MR Egger	0.594555105	0.05	1
		Weighted median	0.292347051	0.05	1
		Inverse variance weighted	0.273721076	0.05	1
		Simple mode	0.734983227	0.05	1
		Weighted mode	0.35678993	0.05	1
	IDP dMRI TBSS ICVF External capsule L	Inverse variance weighted	0.291538838	0.05	1
	IDP dMRI TBSS ICVF Cingulum cingulate gyrus R	Inverse variance weighted	0.388656965	0.05	1
	IDP dMRI TBSS ICVF Cingulum cingulate gyrus L	Wald ratio	0.806203168	0.05	1
	IDP dMRI TBSS ICVF Cingulum hippocampus R	MR Egger	0.571247716	0.05	1
		Weighted median	0.220406497	0.05	1
		Inverse variance weighted	0.300317001	0.05	1
		Simple mode	0.424952547	0.05	1
		Weighted mode	0.352490959	0.05	1
	IDP dMRI TBSS ICVF Cingulum hippocampus L	MR Egger	0.582243999	0.05	1
		Weighted median	0.251146166	0.05	1
		Inverse variance weighted	0.307166129	0.05	1
		Simple mode	0.400208517	0.05	1

		Weighted mode	0.384496221	0.05	1
	IDP dMRI TBSS ICVF Fornix cres+Stria terminalis R	Wald ratio	0.566658579	0.05	1
	IDP dMRI TBSS ICVF Superior longitudinal fasciculus R	Inverse variance weighted	0.964144364	0.05	1
	IDP dMRI TBSS ICVF Superior longitudinal fasciculus L	MR Egger	0.682137489	0.05	1
		Weighted median	0.298985234	0.05	1
		Inverse variance weighted	0.362382393	0.05	1
		Simple mode	0.419996753	0.05	1
		Weighted mode	0.343330342	0.05	1
	IDP dMRI TBSS ICVF Superior fronto-occipital fasciculus R	Inverse variance weighted	0.188175937	0.05	1
	IDP dMRI TBSS ICVF Uncinate fasciculus R	Wald ratio	0.244071339	0.05	1
	IDP dMRI TBSS ICVF Uncinate fasciculus L	Wald ratio	0.244071339	0.05	1
	IDP dMRI TBSS OD Pontine crossing tract	MR Egger	0.708397564	0.05	1
		Weighted median	0.425663214	0.05	1
		Inverse variance weighted	0.634007351	0.05	1
		Simple mode	0.529027664	0.05	1
		Weighted mode	0.506954785	0.05	1
	IDP dMRI TBSS OD Cerebral peduncle R	Wald ratio	0.318995536	0.05	1
	IDP dMRI TBSS OD Cerebral peduncle L	Wald ratio	0.318995536	0.05	1
	IDP dMRI TBSS OD Posterior limb of internal capsule L	Wald ratio	0.458197503	0.05	1
	IDP dMRI TBSS OD External capsule R	Inverse variance weighted	0.125480937	0.05	0.999667598
	IDP dMRI TBSS OD External capsule L	Wald ratio	0.746834948	0.05	1

	IDP dMRI TBSS OD Superior longitudinal fasciculus R	Wald ratio	0.960646665	0.05	1
	IDP dMRI TBSS ISOVF Fornix	Wald ratio	0.851873188	0.05	1
	IDP dMRI TBSS ISOVF External capsule R	Wald ratio	0.213253478	0.05	0.99977613
	IDP dMRI TBSS ISOVF Cingulum cingulate gyrus R	Wald ratio	0.769106023	0.05	1
	IDP dMRI ProbtrackX FA ar l	Wald ratio	0.244071339	0.05	0.999999453
	IDP dMRI ProbtrackX FA atr l	Wald ratio	0.244071339	0.05	1
	IDP dMRI ProbtrackX FA fmi	MR Egger	0.363512436	0.05	0.514592827
		Weighted median	0.248384934	0.05	1
		Inverse variance weighted	0.60226839	0.05	1
		Simple mode	0.525818154	0.05	1
		Weighted mode	0.344410209	0.05	1
	IDP dMRI ProbtrackX FA ifo l	Wald ratio	0.716358696	0.05	1
	IDP dMRI ProbtrackX FA ifo r	Wald ratio	0.742285071	0.05	1
	IDP dMRI ProbtrackX FA ml l	Wald ratio	0.318857879	0.05	1
	IDP dMRI ProbtrackX FA ptr r	Wald ratio	0.118235647	0.05	0.999999999
	IDP dMRI ProbtrackX FA slf l	Wald ratio	0.742285071	0.05	1
	IDP dMRI ProbtrackX FA str l	Wald ratio	0.888108782	0.05	1
	IDP dMRI ProbtrackX FA unc l	Wald ratio	0.244071339	0.05	0.999999982
	IDP dMRI ProbtrackX FA unc r	Wald ratio	0.244071339	0.05	1
	IDP dMRI ProbtrackX MD ar l	Wald ratio	0.244071339	0.05	1
	IDP dMRI ProbtrackX MD ar r	Wald ratio	0.244071339	0.05	1
	IDP dMRI ProbtrackX MD atr l	Wald ratio	0.244071339	0.05	1
	IDP dMRI ProbtrackX MD atr r	Wald ratio	0.244071339	0.05	1

	IDP dMRI ProbtrackX MD cgc l	MR Egger	0.475746425	0.05	1
		Weighted median	0.510188853	0.05	1
		Inverse variance weighted	0.466425041	0.05	1
		Simple mode	0.902912769	0.05	1
		Weighted mode	0.397360236	0.05	1
	IDP dMRI ProbtrackX MD cgc r	Inverse variance weighted	0.806782125	0.05	1
	IDP dMRI ProbtrackX MD fmi	Inverse variance weighted	0.454965923	0.05	1
	IDP dMRI ProbtrackX MD ifo l	Wald ratio	0.716358696	0.05	1
	IDP dMRI ProbtrackX MD ifo r	Wald ratio	0.716358696	0.05	1
	IDP dMRI ProbtrackX MD ilf l	Wald ratio	0.716358696	0.05	1
	IDP dMRI ProbtrackX MD ilf r	Wald ratio	0.716358696	0.05	1
	IDP dMRI ProbtrackX MD ptr l	Wald ratio	0.244071339	0.05	1
	IDP dMRI ProbtrackX MD ptr r	Wald ratio	0.244071339	0.05	1
	IDP dMRI ProbtrackX MD slf l	Inverse variance weighted	0.471527368	0.05	1
	IDP dMRI ProbtrackX MD slf r	Wald ratio	0.716358696	0.05	1
	IDP dMRI ProbtrackX MD str l	Wald ratio	0.875951608	0.05	1
	IDP dMRI ProbtrackX MD str r	Wald ratio	0.244071339	0.05	1
	IDP dMRI ProbtrackX MD unc l	Wald ratio	0.244071339	0.05	1
	IDP dMRI ProbtrackX MD unc r	Inverse variance weighted	0.953242493	0.05	1
	IDP dMRI ProbtrackX MO atr l	Wald ratio	0.836057278	0.05	1
	IDP dMRI ProbtrackX MO ml r	Wald ratio	0.696720005	0.05	1
	IDP T1 FAST ROIs L intracalc cortex	Wald ratio	0.58746958	0.05	1

	IDP dMRI ProbtrackX L1 ilf l	Wald ratio	0.244071339	0.05	1
	IDP T1 FAST ROIs R intracalc cortex	Wald ratio	0.58746958	0.05	1
	IDP dMRI ProbtrackX L1 slf l	Wald ratio	0.244071339	0.05	1
	IDP dMRI ProbtrackX L1 slf r	Wald ratio	0.626814355	0.05	1
	IDP dMRI ProbtrackX L1 str r	Wald ratio	0.337277301	0.05	0.99987255
	IDP dMRI ProbtrackX L1 unc l	Inverse variance weighted	0.333873557	0.05	1
	IDP dMRI ProbtrackX L1 unc r	Wald ratio	0.116974398	0.05	0.857154737
	IDP dMRI ProbtrackX L2 ar l	Wald ratio	0.244071339	0.05	1
	IDP dMRI ProbtrackX L2 ar r	Wald ratio	0.244071339	0.05	1
	IDP dMRI ProbtrackX L2 atr l	Wald ratio	0.244071339	0.05	1
	IDP dMRI ProbtrackX L2 atr r	Wald ratio	0.244071339	0.05	1
	IDP dMRI ProbtrackX L2 cgh l	Wald ratio	0.60105547	0.05	1
	IDP dMRI ProbtrackX L2 fma	Wald ratio	0.259962365	0.05	1
	IDP dMRI ProbtrackX L2 fmi	Inverse variance weighted	0.456411907	0.05	1
	IDP dMRI ProbtrackX L2 ifo l	Wald ratio	0.716358696	0.05	1
	IDP dMRI ProbtrackX L2 ifo r	Wald ratio	0.716358696	0.05	1
	IDP dMRI ProbtrackX L2 ilf l	Wald ratio	0.716358696	0.05	1
	IDP dMRI ProbtrackX L2 ilf r	Wald ratio	0.716358696	0.05	1
	IDP dMRI ProbtrackX L2 ptr l	Wald ratio	0.244071339	0.05	1
	IDP dMRI ProbtrackX L2 ptr r	Wald ratio	0.241105448	0.05	1
	IDP dMRI ProbtrackX L2 slf l	Inverse variance weighted	0.475878668	0.05	1
	IDP dMRI ProbtrackX L2 slf r	Wald ratio	0.716358696	0.05	1
	IDP dMRI ProbtrackX L2 str l	Wald ratio	0.875951608	0.05	1

	IDP dMRI ProbtrackX L2 str r	Wald ratio	0.875951608	0.05	1
	IDP dMRI ProbtrackX L2 unc l	Wald ratio	0.244071339	0.05	1
	IDP dMRI ProbtrackX L2 unc r	Inverse variance weighted	0.936674913	0.05	1
	IDP dMRI ProbtrackX L3 ar l	Wald ratio	0.244071339	0.05	1
	IDP dMRI ProbtrackX L3 ar r	Wald ratio	0.244071339	0.05	1
	IDP dMRI ProbtrackX L3 atr l	Wald ratio	0.244071339	0.05	1
	IDP dMRI ProbtrackX L3 atr r	Wald ratio	0.244071339	0.05	1
	IDP dMRI ProbtrackX L3 fmi	MR Egger	0.619921026	0.05	1
		Weighted median	0.210799772	0.05	1
		Inverse variance weighted	0.354531773	0.05	1
		Simple mode	0.40775177	0.05	1
		Weighted mode	0.389242998	0.05	1
	IDP dMRI ProbtrackX L3 ifo l	Inverse variance weighted	0.430431649	0.05	1
	IDP dMRI ProbtrackX L3 ifo r	Wald ratio	0.716358696	0.05	1
	IDP dMRI ProbtrackX L3 ilf l	Wald ratio	0.716358696	0.05	1
	IDP dMRI ProbtrackX L3 ilf r	Wald ratio	0.716358696	0.05	1
	IDP dMRI ProbtrackX L3 ptr l	Wald ratio	0.244071339	0.05	1
	IDP dMRI ProbtrackX L3 ptr r	Wald ratio	0.241105448	0.05	1
	IDP dMRI ProbtrackX L3 slf l	Inverse variance weighted	0.463412771	0.05	1
	IDP dMRI ProbtrackX L3 slf r	Wald ratio	0.716358696	0.05	1
	IDP dMRI ProbtrackX L3 str l	Wald ratio	0.241105448	0.05	1
	IDP dMRI ProbtrackX L3 unc l	Wald ratio	0.244071339	0.05	1

	IDP dMRI ProbtrackX L3 unc r	Inverse variance weighted	0.981109242	0.05	1
	IDP dMRI ProbtrackX ICVF ar l	MR Egger	0.633586919	0.05	1
		Weighted median	0.188814056	0.05	1
		Inverse variance weighted	0.194668037	0.05	1
		Simple mode	0.404697608	0.05	1
		Weighted mode	0.340973137	0.05	1
	IDP dMRI ProbtrackX ICVF ar r	MR Egger	0.638919426	0.05	1
		Weighted median	0.197791775	0.05	1
		Inverse variance weighted	0.197072373	0.05	1
		Simple mode	0.41917471	0.05	1
		Weighted mode	0.361691489	0.05	1
	IDP dMRI ProbtrackX ICVF atr l	MR Egger	0.557430946	0.05	0.999999997
		Weighted median	0.214291479	0.05	1
		Inverse variance weighted	0.299647042	0.05	1
		Simple mode	0.40785424	0.05	1
		Weighted mode	0.33940052	0.05	1
	IDP dMRI ProbtrackX ICVF atr r	MR Egger	0.553024454	0.05	0.999999932
		Weighted median	0.191890338	0.05	1
		Inverse variance weighted	0.302825736	0.05	1
		Simple mode	0.427107842	0.05	1
		Weighted mode	0.365424704	0.05	1
	IDP dMRI ProbtrackX ICVF cgc l	Wald ratio	0.806203168	0.05	1

	IDP dMRI ProbtrackX ICVF cgh l	MR Egger	0.552103297	0.05	1
		Weighted median	0.203660812	0.05	1
		Inverse variance weighted	0.296722222	0.05	1
		Simple mode	0.391197322	0.05	1
		Weighted mode	0.361482537	0.05	1
	IDP dMRI ProbtrackX ICVF cgh r	Wald ratio	0.244071339	0.05	1
	IDP dMRI ProbtrackX ICVF cst l	MR Egger	0.637930281	0.05	1
		Weighted median	0.167626117	0.05	1
		Inverse variance weighted	0.202070161	0.05	1
		Simple mode	0.401479508	0.05	1
		Weighted mode	0.359653864	0.05	1
	IDP dMRI ProbtrackX ICVF cst r	MR Egger	0.497998896	0.05	0.999999734
		Weighted median	0.183009744	0.05	1
		Inverse variance weighted	0.342522757	0.05	1
		Simple mode	0.454164715	0.05	1
		Weighted mode	0.329859483	0.05	1
	IDP dMRI ProbtrackX ICVF fma	Inverse variance weighted	0.397659409	0.05	1
	IDP dMRI ProbtrackX ICVF fmi	MR Egger	0.409336186	0.05	0.999996791
		Weighted median	0.46683903	0.05	1
		Inverse variance weighted	0.576554012	0.05	1
		Simple mode	0.514125337	0.05	1
		Weighted mode	0.339793049	0.05	1

	IDP dMRI ProbtrackX ICVF ifo l	Inverse variance weighted	0.400561439	0.05	1
	IDP dMRI ProbtrackX ICVF ifo r	Inverse variance weighted	0.40317974	0.05	1
	IDP dMRI ProbtrackX ICVF ilf l	Inverse variance weighted	0.411449406	0.05	1
	IDP dMRI ProbtrackX ICVF ilf r	Wald ratio	0.716358696	0.05	1
	IDP dMRI ProbtrackX ICVF mcp	Wald ratio	0.882471773	0.05	1
	IDP dMRI ProbtrackX ICVF ml l	Wald ratio	0.117974212	0.05	0.999999998
	IDP dMRI ProbtrackX ICVF ml r	Wald ratio	0.244071339	0.05	1
	IDP dMRI ProbtrackX ICVF ptr l	Wald ratio	0.716358696	0.05	1
	IDP dMRI ProbtrackX ICVF ptr r	Inverse variance weighted	0.615687317	0.05	1
	IDP dMRI ProbtrackX ICVF slf l	MR Egger	0.621127191	0.05	1
		Weighted median	0.2790865	0.05	1
		Inverse variance weighted	0.338681149	0.05	1
		Simple mode	0.435454425	0.05	1
		Weighted mode	0.373655762	0.05	1
	IDP dMRI ProbtrackX ICVF slf r	Inverse variance weighted	0.956162079	0.05	1
	IDP dMRI ProbtrackX ICVF str l	MR Egger	0.569905529	0.05	0.99999306
		Weighted median	0.175359955	0.05	1
		Inverse variance weighted	0.325146567	0.05	1
		Simple mode	0.432158894	0.05	1
		Weighted mode	0.370291378	0.05	1
	IDP dMRI ProbtrackX ICVF str r	MR Egger	0.600243349	0.05	0.999999785
		Weighted median	0.182798017	0.05	1

		Inverse variance weighted	0.335173281	0.05	1
		Simple mode	0.433402071	0.05	1
		Weighted mode	0.375012528	0.05	1
	IDP dMRI ProbtrackX ICVF unc l	Inverse variance weighted	0.192810462	0.05	1
	IDP dMRI ProbtrackX ICVF unc r	Inverse variance weighted	0.361903174	0.05	1
	IDP dMRI ProbtrackX OD atr r	Wald ratio	0.804492702	0.05	1
	IDP dMRI ProbtrackX OD ilf l	Wald ratio	0.948709469	0.05	1
	IDP dMRI ProbtrackX OD slf l	Wald ratio	0.475346549	0.05	1
	IDP dMRI ProbtrackX OD str l	Inverse variance weighted	0.665520525	0.05	1
	IDP dMRI ProbtrackX ISOVF atr r	Wald ratio	0.244071339	0.05	0.999994619
	IDP dMRI ProbtrackX ISOVF cgc l	Wald ratio	0.200130752	0.05	1
	IDP dMRI ProbtrackX ISOVF ifo r	Wald ratio	0.218634351	0.05	0.999788321
	IDP dMRI ProbtrackX ISOVF ilf l	Wald ratio	0.365577152	0.05	0.999999997
	IDP dMRI ProbtrackX ISOVF ilf r	MR Egger	0.51411324	0.05	1
		Weighted median	0.755879679	0.05	1
		Inverse variance weighted	0.823302262	0.05	1
		Simple mode	0.776193093	0.05	1
		Weighted mode	0.794503635	0.05	1
	IDP dMRI ProbtrackX ISOVF slf l	Inverse variance weighted	0.068173282	0.05	0.99911884
	IDP dMRI ProbtrackX ISOVF slf r	MR Egger	0.789217274	0.05	1
		Weighted median	0.805206224	0.05	1
		Inverse variance weighted	0.439298277	0.05	1

		Simple mode	0.953975615	0.05	1
		Weighted mode	0.980899534	0.05	1
	IDP dMRI ProbtrackX ISOVF unc l	Wald ratio	0.1179551	0.05	0.999250486
	IDP dMRI ProbtrackX ISOVF unc r	Wald ratio	0.118116496	0.05	0.999992627
	IDP T1 SIENAX brain-normalised volume	Wald ratio	0.725730978	0.05	1
TMD muscular pain lnked with fibromyalgia	IDP T1 SIENAX peripheral grey normalised volume	Wald ratio	0.825447659	0.05	1
	IDP T1 FAST ROIs R temp occ fusif cortex	Wald ratio	0.16549383	0.05	1
	IDP T1 FAST ROIs R occ pole	Inverse variance weighted	0.43473963	0.05	1
	IDP T1 FAST ROIs L thalamus	Inverse variance weighted	0.196458213	0.05	1
	IDP T1 FAST ROIs R thalamus	Wald ratio	0.069611091	0.05	1
	IDP T1 FAST ROIs L putamen	Wald ratio	0.659119587	0.05	1
	IDP T1 FAST ROIs R putamen	Wald ratio	0.659119587	0.05	1
	IDP T1 FAST ROIs L pallidum	Wald ratio	0.625569314	0.05	1
	IDP T1 FIRST left caudate volume	Wald ratio	0.934043472	0.05	1
	IDP T1 FAST ROIs L hippocampus	Inverse variance weighted	0.227209075	0.05	1
	IDP T1 FAST ROIs R hippocampus	Wald ratio	0.331106494	0.05	1
	IDP T1 FAST ROIs L ventral striatum	Wald ratio	0.659119587	0.05	1
	IDP T1 FAST ROIs R ventral striatum	Inverse variance weighted	0.445493361	0.05	1
	IDP T1 FAST ROIs L cerebellum VI	Wald ratio	0.309543093	0.05	1
	IDP T1 FAST ROIs L cerebellum crus I	MR Egger	0.553357231	0.05	1
		Weighted median	0.221315204	0.05	1
		Inverse variance weighted	0.569794435	0.05	1

		Simple mode	0.319176818	0.05	1
		Weighted mode	0.176295011	0.05	1
	IDP T1 FAST ROIs R cerebellum crus I	Inverse variance weighted	0.653935384	0.05	1
	IDP T1 FAST ROIs L cerebellum crus II	MR Egger	0.93010152	0.05	1
		Weighted median	0.657413351	0.05	1
		Inverse variance weighted	0.289357965	0.05	1
		Simple mode	0.794810846	0.05	1
		Weighted mode	0.786331789	0.05	1
	IDP T1 FAST ROIs V cerebellum crus II	Inverse variance weighted	0.288207126	0.05	1
	IDP T1 FAST ROIs R cerebellum crus II	Inverse variance weighted	0.708471905	0.05	1
	IDP T1 FIRST left putamen volume	Inverse variance weighted	0.331275135	0.05	1
	IDP T1 FAST ROIs R cerebellum VIIb	Wald ratio	0.974674585	0.05	1
	IDP T1 FAST ROIs V cerebellum VIIIa	MR Egger	0.526542784	0.05	1
		Weighted median	0.4682689	0.05	1
		Inverse variance weighted	0.461987246	0.05	1
		Simple mode	0.305960362	0.05	1
		Weighted mode	0.865027224	0.05	1
	IDP T1 FAST ROIs L cerebellum VIIIb	Wald ratio	0.691663905	0.05	1
	IDP T1 FAST ROIs V cerebellum VIIIb	Inverse variance weighted	0.741791717	0.05	1
	IDP T1 FAST ROIs R cerebellum VIIIb	Wald ratio	0.659119587	0.05	1
	IDP T1 FAST ROIs L cerebellum IX	Inverse variance weighted	0.677946196	0.05	1

	IDP T1 FIRST right putamen volume	Wald ratio	0.83014044	0.05	1
	IDP T1 FAST ROIs V cerebellum IX	Inverse variance weighted	0.730469669	0.05	1
	IDP T1 FAST ROIs R cerebellum IX	Inverse variance weighted	0.437683245	0.05	1
	IDP T1 FAST ROIs V cerebellum X	Wald ratio	0.659119587	0.05	1
	IDP T2 FLAIR BIANCA WMH volume	Inverse variance weighted	0.640425109	0.05	1
	IDP SWI T2star left caudate	MR Egger	0.471293653	0.05	1
		Weighted median	0.617647795	0.05	1
		Inverse variance weighted	0.420479992	0.05	1
		Simple mode	0.873257557	0.05	1
		Weighted mode	0.605532404	0.05	1
	IDP SWI T2star right caudate	MR Egger	0.804622948	0.05	1
		Weighted median	0.591030593	0.05	1
		Inverse variance weighted	0.699232873	0.05	1
		Simple mode	0.64702152	0.05	1
		Weighted mode	0.550098636	0.05	1
	IDP T1 FIRST left pallidum volume	Wald ratio	0.155922656	0.05	1
	IDP SWI T2star left putamen	MR Egger	0.411082467	0.05	1
		Weighted median	0.541822834	0.05	1
		Inverse variance weighted	0.219598234	0.05	1
		Simple mode	0.705455424	0.05	1
		Weighted mode	0.717748598	0.05	1
	IDP SWI T2star right putamen	MR Egger	0.311892268	0.05	1

		Weighted median	0.600997605	0.05	1
		Inverse variance weighted	0.311427582	0.05	1
		Simple mode	0.841258388	0.05	1
		Weighted mode	0.850449304	0.05	1
	IDP SWI T2star left pallidum	MR Egger	0.866415298	0.05	1
		Weighted median	0.381480945	0.05	1
		Inverse variance weighted	0.858409081	0.05	1
		Simple mode	0.556271701	0.05	1
		Weighted mode	0.503768022	0.05	1
	IDP SWI T2star right pallidum	MR Egger	0.783275959	0.05	1
		Weighted median	0.815162806	0.05	1
		Inverse variance weighted	0.819829196	0.05	1
		Simple mode	0.793214276	0.05	1
		Weighted mode	0.757532704	0.05	1
	IDP T1 FIRST right pallidum volume	Wald ratio	0.155922656	0.05	1
	IDP dMRI TBSS FA Genu of corpus callosum	Wald ratio	0.394986168	0.05	1
	IDP dMRI TBSS FA Splenium of corpus callosum	Inverse variance weighted	0.143293158	0.05	1
	IDP dMRI TBSS FA Corticospinal tract L	Wald ratio	0.133349743	0.05	1
	IDP dMRI TBSS FA Superior cerebellar peduncle R	Inverse variance weighted	0.193251074	0.05	1
	IDP dMRI TBSS FA Superior cerebellar peduncle L	Inverse variance weighted	0.18401861	0.05	1
	IDP dMRI TBSS FA Posterior limb of internal capsule R	Wald ratio	0.972492191	0.05	1

	IDP dMRI TBSS FA Retrolenticular part of internal capsule R	Inverse variance weighted	0.189641102	0.05	1
	IDP dMRI TBSS FA Anterior corona radiata R	Wald ratio	0.394986168	0.05	1
	IDP dMRI TBSS FA Anterior corona radiata L	Wald ratio	0.394986168	0.05	1
	IDP dMRI TBSS FA Posterior thalamic radiation R	Wald ratio	0.155496452	0.05	1
	IDP dMRI TBSS FA Posterior thalamic radiation L	Wald ratio	0.144675584	0.05	1
	IDP dMRI TBSS FA Sagittal stratum R	Wald ratio	0.400775693	0.05	1
	IDP dMRI TBSS FA Cingulum cingulate gyrus R	Inverse variance weighted	0.232463177	0.05	1
	IDP dMRI TBSS FA Superior longitudinal fasciculus L	Inverse variance weighted	0.693395056	0.05	1
	IDP dMRI TBSS FA Uncinate fasciculus L	Wald ratio	0.246837709	0.05	1
	IDP dMRI TBSS MD Genu of corpus callosum	Wald ratio	0.394986168	0.05	1
	IDP dMRI TBSS MD Body of corpus callosum	Wald ratio	0.996492826	0.05	1
	IDP dMRI TBSS MD Splenium of corpus callosum	Inverse variance weighted	0.363454388	0.05	1
	IDP dMRI TBSS MD Corticospinal tract R	Wald ratio	0.4137905	0.05	1
	IDP dMRI TBSS MD Superior cerebellar peduncle R	Wald ratio	0.592281348	0.05	1
	IDP dMRI TBSS MD Superior cerebellar peduncle L	Wald ratio	0.197100982	0.05	1
	IDP dMRI TBSS MD Anterior limb of internal capsule L	Wald ratio	0.469587514	0.05	1
	IDP dMRI TBSS MD Retrolenticular part of internal capsule R	Wald ratio	0.156425206	0.05	1
	IDP T1 FIRST left caudate volume plus IDP T1 FIRST right caudate volume	Wald ratio	0.90234515	0.05	1
	IDP T1 FIRST left putamen volume plus IDP T1 FIRST right putamen volume	Inverse variance weighted	0.258267791	0.05	1

	IDP T1 FIRST left pallidum volume plus IDP T1 FIRST right pallidum volume	Wald ratio	0.155922656	0.05	1
	IDP SWI T2star left thalamus plus IDP SWI T2star right thalamus	MR Egger	0.78696552	0.05	1
		Weighted median	0.671892115	0.05	1
		Inverse variance weighted	0.924919592	0.05	1
		Simple mode	0.71150899	0.05	1
		Weighted mode	0.682060878	0.05	1
	IDP dMRI TBSS MD Retrolenticular part of internal capsule L	Wald ratio	0.156425206	0.05	1
	IDP SWI T2star left caudate plus IDP SWI T2star right caudate	MR Egger	0.778373624	0.05	1
		Weighted median	0.908069726	0.05	1
		Inverse variance weighted	0.41948417	0.05	1
		Simple mode	0.810688632	0.05	1
		Weighted mode	0.847747035	0.05	1
	IDP SWI T2star left putamen plus IDP SWI T2star right putamen	MR Egger	0.401635338	0.05	1
		Weighted median	0.708049915	0.05	1
		Inverse variance weighted	0.496431281	0.05	1
		Simple mode	0.918909634	0.05	1
		Weighted mode	0.888307894	0.05	1
	IDP SWI T2star left pallidum plus IDP SWI T2star right pallidum	MR Egger	0.838221979	0.05	1
		Weighted median	0.859819876	0.05	1
		Inverse variance weighted	0.896149966	0.05	1
		Simple mode	0.858748092	0.05	1
		Weighted mode	0.824638361	0.05	1

	volume Left-Lateral-Ventricle	MR Egger	0.64568737	0.05	1
		Weighted median	0.085611448	0.05	1
		Inverse variance weighted	0.6621558	0.05	1
		Simple mode	0.350341553	0.05	1
		Weighted mode	0.348145412	0.05	1
	volume Left-Cerebellum-White-Matter	Inverse variance weighted	0.320159555	0.05	1
	volume Left-Cerebellum-Cortex	Inverse variance weighted	0.73796099	0.05	1
	IDP dMRI TBSS MD Anterior corona radiata R	Inverse variance weighted	0.124763119	0.05	1
	volume Left-Putamen	Inverse variance weighted	0.540821202	0.05	1
	volume 4th-Ventricle	Inverse variance weighted	0.783905828	0.05	1
	volume Brain-Stem	Wald ratio	0.758316474	0.05	1
	volume Left-Hippocampus	Inverse variance weighted	0.811732703	0.05	1
	volume CSF	MR Egger	0.592301571	0.05	1
		Weighted median	0.511258887	0.05	1
		Inverse variance weighted	0.73863279	0.05	1
		Simple mode	0.531217812	0.05	1
		Weighted mode	0.537054985	0.05	1
	IDP dMRI TBSS MD Anterior corona radiata L	Inverse variance weighted	0.131959292	0.05	1
	volume Left-Accumbens-area	Wald ratio	0.659119587	0.05	1
	volume Right-Lateral-Ventricle	Inverse variance weighted	0.693974731	0.05	1
	volume Right-Inf-Lat-Vent	Wald ratio	0.130500952	0.05	1

	volume Right-Cerebellum-White-Matter	Wald ratio	0.869825928	0.05	1
	volume Right-Cerebellum-Cortex	MR Egger	0.328570628	0.05	1
		Weighted median	0.364948896	0.05	1
		Inverse variance weighted	0.773348283	0.05	1
		Simple mode	0.695295426	0.05	1
		Weighted mode	0.298017244	0.05	1
	IDP dMRI TBSS MD Superior corona radiata R	Inverse variance weighted	0.124895575	0.05	1
	volume Right-Putamen	Wald ratio	0.83014044	0.05	1
	volume Right-Hippocampus	Wald ratio	0.331106494	0.05	1
	volume Right-Amygdala	Wald ratio	0.202209836	0.05	1
	volume Right-VentralDC	Wald ratio	0.78859248	0.05	1
	volume CC Posterior	Wald ratio	0.199732896	0.05	1
	IDP dMRI TBSS MD Superior corona radiata L	Inverse variance weighted	0.125613716	0.05	1
	IDP dMRI TBSS MD Posterior corona radiata R	Wald ratio	0.156425206	0.05	1
	volume BrainSegVol-to-eTIV	Wald ratio	0.995264808	0.05	1
	IDP dMRI TBSS MD Posterior corona radiata L	Wald ratio	0.144675584	0.05	1
	volume rhSurfaceHoles	Wald ratio	0.607655587	0.05	1
	DKTatlas lh cuneus area	Wald ratio	0.52791753	0.05	1
	IDP dMRI TBSS MD Posterior thalamic radiation R	Wald ratio	0.144675584	0.05	1
	DKTatlas lh lateraloccipital area	Wald ratio	0.52791753	0.05	1
	DKTatlas lh lingual area	Wald ratio	0.562183051	0.05	1
	IDP dMRI TBSS MD Posterior thalamic radiation L	Wald ratio	0.156425206	0.05	1

	DKTatlas lh parstriangularis area	Wald ratio	0.139563261	0.05	1
	DKTatlas lh pericalcarine area	Inverse variance weighted	0.200487975	0.05	1
	DKTatlas lh postcentral area	Inverse variance weighted	0.398617809	0.05	1
	DKTatlas lh posteriorcingulate area	Wald ratio	0.692752889	0.05	1
	DKTatlas lh precentral area	Wald ratio	0.093235599	0.05	1
	DKTatlas lh precuneus area	Wald ratio	0.342515673	0.05	1
	IDP dMRI TBSS MD Sagittal stratum R	Wald ratio	0.156425206	0.05	1
	DKTatlas lh superiorparietal area	Wald ratio	0.024710309	0.05	1
	DKTatlas lh superiortemporal area	Wald ratio	0.770012303	0.05	1
	DKTatlas lh supramarginal area	Wald ratio	0.411211453	0.05	1
	DKTatlas lh WhiteSurfArea area	Wald ratio	0.493957625	0.05	1
	IDP dMRI TBSS MD Sagittal stratum L	Wald ratio	0.156425206	0.05	1
	a2009s lh G&S subcentral area	Wald ratio	0.093235599	0.05	1
	a2009s lh G cuneus area	Wald ratio	0.61273532	0.05	1
	a2009s lh G front inf-Opercular area	Wald ratio	0.417314967	0.05	1
	IDP dMRI TBSS MD External capsule R	Wald ratio	0.394986168	0.05	1
	IDP dMRI TBSS MD External capsule L	Inverse variance weighted	0.826594135	0.05	1
	a2009s lh G pariet inf-Supramar area	Wald ratio	0.411211453	0.05	1
	a2009s lh G parietal sup area	Wald ratio	0.024286481	0.05	1
	a2009s lh G postcentral area	Wald ratio	0.024157183	0.05	0.999999999
	a2009s lh G precentral area	Wald ratio	0.093235599	0.05	1
	a2009s lh G precuneus area	Wald ratio	0.61273532	0.05	1
		MR Egger	0.924105243	0.05	1

	IDP dMRI TBSS MD Cingulum cingulate gyrus R	Weighted median	0.291675783	0.05	1
		Inverse variance weighted	0.321123634	0.05	1
		Simple mode	0.396601653	0.05	1
		Weighted mode	0.436735804	0.05	1
	IDP dMRI TBSS MD Cingulum cingulate gyrus L	MR Egger	0.902455677	0.05	1
		Weighted median	0.296970601	0.05	1
		Inverse variance weighted	0.327899897	0.05	1
		Simple mode	0.418864222	0.05	1
		Weighted mode	0.432103834	0.05	1
	a2009s lh S calcarine area	Wald ratio	0.562183051	0.05	1
	a2009s lh S central area	Wald ratio	0.095580705	0.05	1
	a2009s lh S collat transv ant area	Wald ratio	0.094984738	0.05	1
	a2009s lh S front middle area	Wald ratio	0.831409068	0.05	1
	IDP dMRI TBSS MD Cingulum hippocampus R	Wald ratio	0.156425206	0.05	1
	a2009s lh S intrapariet&P trans area	Wald ratio	0.024286481	0.05	1
	IDP dMRI TBSS MD Cingulum hippocampus L	Wald ratio	0.156425206	0.05	1
	a2009s lh S subparietal area	Wald ratio	0.562183051	0.05	1
	DKTatlas rh cuneus area	Wald ratio	0.562183051	0.05	1
	DKTatlas rh lingual area	Wald ratio	0.5588321	0.05	1
	DKTatlas rh parstriangularis area	Wald ratio	0.286406584	0.05	1
	DKTatlas rh pericalcarine area	Inverse variance weighted	0.815327582	0.05	1
	IDP dMRI TBSS MD Superior longitudinal fasciculus R	Wald ratio	0.156425206	0.05	1

	DKTatlas rh postcentral area	Wald ratio	0.095580705	0.05	1
	DKTatlas rh precentral area	Wald ratio	0.093235599	0.05	1
	IDP dMRI TBSS MD Superior longitudinal fasciculus L	Wald ratio	0.156425206	0.05	1
	a2009s rh G&S subcentral area	Wald ratio	0.095580705	0.05	1
	a2009s rh G&S cingul-Mid-Post area	Wald ratio	0.79052803	0.05	1
	a2009s rh G cuneus area	Wald ratio	0.562183051	0.05	1
	IDP dMRI TBSS MD Superior fronto-occipital fasciculus L	Wald ratio	0.133166826	0.05	1
	a2009s rh G oc-temp med-Lingual area	Wald ratio	0.5588321	0.05	1
	a2009s rh G parietal sup area	Wald ratio	0.56420857	0.05	1
	IDP dMRI TBSS MD Uncinate fasciculus R	Wald ratio	0.482884584	0.05	1
	a2009s rh G precentral area	Wald ratio	0.093235599	0.05	1
	a2009s rh G precuneus area	Wald ratio	0.737160126	0.05	1
	IDP dMRI TBSS MD Uncinate fasciculus L	Inverse variance weighted	0.737209853	0.05	1
	a2009s rh Pole occipital area	Wald ratio	0.562183051	0.05	1
	a2009s rh S calcarine area	Wald ratio	0.5588321	0.05	1
	a2009s rh S central area	Wald ratio	0.093235599	0.05	1
	a2009s rh S collat transv ant area	Wald ratio	0.094984738	0.05	1
	a2009s rh S orbital med-olfact area	Wald ratio	0.148613996	0.05	1
	IDP dMRI TBSS MO Pontine crossing tract	MR Egger	0.791639493	0.05	1
		Weighted median	0.26391552	0.05	1
		Inverse variance weighted	0.122558545	0.05	1
		Simple mode	0.515363242	0.05	1

		Weighted mode	0.413748127	0.05	1
	DKTatlas lh postcentral thickness	Wald ratio	0.095580705	0.05	1
	a2009s lh G insular short thickness	Wald ratio	0.861547959	0.05	1
	a2009s lh G postcentral thickness	Wald ratio	0.095580705	0.05	1
	IDP T1 SIENAX CSF normalised volume	Inverse variance weighted	0.05963106	0.05	1
	IDP dMRI TBSS MO Medial lemniscus R	Wald ratio	0.075014801	0.05	1
	a2009s lh S postcentral thickness	Wald ratio	0.476322229	0.05	1
	DKTatlas rh lateraloccipital thickness	Wald ratio	0.659119587	0.05	1
	DKTatlas rh parstriangularis thickness	Wald ratio	0.81424997	0.05	1
	DKTatlas rh postcentral thickness	Wald ratio	0.095580705	0.05	1
	DKTatlas rh posteriorcingulate thickness	Inverse variance weighted	0.071972075	0.05	1
	a2009s rh G&S cingul-Mid-Ant thickness	Wald ratio	0.577600689	0.05	1
	a2009s rh G cuneus thickness	Wald ratio	0.659119587	0.05	1
	a2009s rh G postcentral thickness	Wald ratio	0.095580705	0.05	1
	a2009s rh Pole occipital thickness	Wald ratio	0.659119587	0.05	1
	a2009s rh S oc sup&transversal thickness	Wald ratio	0.890054462	0.05	1
	a2009s rh S parieto occipital thickness	Wald ratio	0.716241578	0.05	1
	IDP dMRI TBSS MO Anterior corona radiata R	Wald ratio	0.108535405	0.05	1
	IDP dMRI TBSS MO Superior corona radiata R	Wald ratio	0.260423023	0.05	1
	IDP dMRI TBSS MO Cingulum cingulate gyrus L	Inverse variance weighted	0.212612215	0.05	1
	IDP dMRI TBSS L1 Anterior limb of internal capsule L	Wald ratio	0.469587514	0.05	1
	IDP dMRI TBSS L1 Posterior limb of internal capsule R	Wald ratio	0.856507359	0.05	1

	IDP dMRI TBSS L1 Posterior limb of internal capsule L	Wald ratio	0.061088793	0.05	1
	IDP dMRI TBSS L1 Anterior corona radiata R	Wald ratio	0.394986168	0.05	1
	IDP dMRI TBSS L1 Anterior corona radiata L	Wald ratio	0.394986168	0.05	1
	IDP dMRI TBSS L1 Posterior corona radiata R	Wald ratio	0.997916753	0.05	1
	IDP dMRI TBSS L1 Posterior corona radiata L	Wald ratio	0.394986168	0.05	1
	IDP dMRI TBSS L1 Sagittal stratum L	Wald ratio	0.394986168	0.05	1
	IDP dMRI TBSS L1 External capsule R	Wald ratio	0.394986168	0.05	1
	IDP dMRI TBSS L1 External capsule L	Inverse variance weighted	0.727649536	0.05	1
	IDP dMRI TBSS L1 Fornix cres+Stria terminalis L	Wald ratio	0.38447484	0.05	1
	IDP T1 FAST ROIs L precentral gyrus	Wald ratio	0.095580705	0.05	1
	IDP dMRI TBSS L1 Superior longitudinal fasciculus R	Inverse variance weighted	0.679311324	0.05	1
	IDP dMRI TBSS L1 Uncinate fasciculus L	Wald ratio	0.548087164	0.05	1
	IDP dMRI TBSS L2 Pontine crossing tract	Wald ratio	0.075014801	0.05	1
	IDP dMRI TBSS L2 Genu of corpus callosum	MR Egger	0.98485476	0.05	1
		Weighted median	0.29184764	0.05	1
		Inverse variance weighted	0.12416323	0.05	1
		Simple mode	0.498884626	0.05	1
		Weighted mode	0.473703093	0.05	1
	IDP dMRI TBSS L2 Body of corpus callosum	Wald ratio	0.394986168	0.05	1
	IDP dMRI TBSS L2 Splenium of corpus callosum	MR Egger	0.673495302	0.05	0.999998979
		Weighted median	0.060190794	0.05	1

		Inverse variance weighted	0.045692345	0.05	1
		Simple mode	0.247169373	0.05	1
		Weighted mode	0.226702049	0.05	1
	IDP dMRI TBSS L2 Corticospinal tract R	Wald ratio	0.4137905	0.05	1
	IDP T1 SIENAX CSF unnormalised volume	MR Egger	0.516941761	0.05	1
		Weighted median	0.075600911	0.05	1
		Inverse variance weighted	0.627105056	0.05	1
		Simple mode	0.356680052	0.05	1
		Weighted mode	0.331409435	0.05	1
	IDP T1 FAST ROIs L temporal pole	Wald ratio	0.834870425	0.05	1
	IDP dMRI TBSS L2 Superior cerebellar peduncle R	Inverse variance weighted	0.191992913	0.05	1
	IDP dMRI TBSS L2 Superior cerebellar peduncle L	Wald ratio	0.197100982	0.05	1
	IDP dMRI TBSS L2 Posterior limb of internal capsule R	Inverse variance weighted	0.310022636	0.05	1
	IDP dMRI TBSS L2 Posterior limb of internal capsule L	Wald ratio	0.164047873	0.05	1
	IDP dMRI TBSS L2 Retrolenticular part of internal capsule R	Inverse variance weighted	0.111483352	0.05	1
	IDP dMRI TBSS L2 Anterior corona radiata R	Inverse variance weighted	0.125816022	0.05	1
	IDP dMRI TBSS L2 Anterior corona radiata L	Wald ratio	0.394986168	0.05	1
	IDP dMRI TBSS L2 Superior corona radiata R	Inverse variance weighted	0.075759148	0.05	1
	IDP dMRI TBSS L2 Superior corona radiata L	Wald ratio	0.156425206	0.05	1
	IDP dMRI TBSS L2 Posterior corona radiata R	Wald ratio	0.144675584	0.05	1

	IDP dMRI TBSS L2 Posterior corona radiata L	Wald ratio	0.144675584	0.05	1
	IDP dMRI TBSS L2 Posterior thalamic radiation R	Wald ratio	0.155496452	0.05	1
	IDP dMRI TBSS L2 Posterior thalamic radiation L	Wald ratio	0.144675584	0.05	1
	IDP dMRI TBSS L2 Sagittal stratum R	Wald ratio	0.156425206	0.05	1
	IDP dMRI TBSS L2 Cingulum cingulate gyrus R	Wald ratio	0.394986168	0.05	1
	IDP dMRI TBSS L2 Cingulum hippocampus R	Wald ratio	0.156425206	0.05	1
	IDP dMRI TBSS L2 Cingulum hippocampus L	Wald ratio	0.394986168	0.05	1
	IDP dMRI TBSS L2 Uncinate fasciculus L	Wald ratio	0.394986168	0.05	1
	IDP dMRI TBSS L3 Genu of corpus callosum	Wald ratio	0.394986168	0.05	1
	IDP dMRI TBSS L3 Body of corpus callosum	Wald ratio	0.394986168	0.05	1
	IDP dMRI TBSS L3 Splenium of corpus callosum	Wald ratio	0.182422439	0.05	1
	IDP dMRI TBSS L3 Inferior cerebellar peduncle R	Wald ratio	0.394986168	0.05	1
	IDP dMRI TBSS L3 Inferior cerebellar peduncle L	Wald ratio	0.394986168	0.05	1
	IDP dMRI TBSS L3 Superior cerebellar peduncle R	Inverse variance weighted	0.188070795	0.05	1
	IDP dMRI TBSS L3 Superior cerebellar peduncle L	Wald ratio	0.197100982	0.05	1
	IDP dMRI TBSS L3 Cerebral peduncle R	Wald ratio	0.841480581	0.05	1
	IDP dMRI TBSS L3 Anterior limb of internal capsule R	Wald ratio	0.541043383	0.05	1
	IDP dMRI TBSS L3 Anterior limb of internal capsule L	Wald ratio	0.651592918	0.05	1
	IDP dMRI TBSS L3 Retrolenticular part of internal capsule R	Wald ratio	0.156425206	0.05	1

	IDP dMRI TBSS L3 Anterior corona radiata R	Inverse variance weighted	0.125483211	0.05	1
	IDP dMRI TBSS L3 Anterior corona radiata L	Inverse variance weighted	0.13378369	0.05	1
	IDP dMRI TBSS L3 Posterior corona radiata R	Inverse variance weighted	0.459628554	0.05	1
	IDP dMRI TBSS L3 Posterior thalamic radiation R	Wald ratio	0.155496452	0.05	1
	IDP dMRI TBSS L3 Posterior thalamic radiation L	Wald ratio	0.156425206	0.05	1
	IDP dMRI TBSS L3 Sagittal stratum R	Wald ratio	0.156425206	0.05	1
	IDP dMRI TBSS L3 Sagittal stratum L	Wald ratio	0.156425206	0.05	1
	IDP dMRI TBSS L3 External capsule R	Wald ratio	0.394986168	0.05	1
	IDP dMRI TBSS L3 External capsule L	Wald ratio	0.394986168	0.05	1
	IDP dMRI TBSS L3 Cingulum cingulate gyrus R	Inverse variance weighted	0.130992386	0.05	1
	IDP dMRI TBSS L3 Cingulum cingulate gyrus L	Wald ratio	0.394986168	0.05	1
	IDP dMRI TBSS L3 Cingulum hippocampus R	Wald ratio	0.394986168	0.05	1
	IDP dMRI TBSS L3 Cingulum hippocampus L	Wald ratio	0.394986168	0.05	1
	IDP dMRI TBSS L3 Fornix cres+Stria terminalis R	Wald ratio	0.49635722	0.05	1
	IDP dMRI TBSS L3 Superior longitudinal fasciculus R	Wald ratio	0.156425206	0.05	1
	IDP dMRI TBSS L3 Superior longitudinal fasciculus L	Wald ratio	0.156425206	0.05	1
	IDP dMRI TBSS L3 Uncinate fasciculus R	Wald ratio	0.394986168	0.05	1
	IDP dMRI TBSS L3 Uncinate fasciculus L	Inverse variance weighted	0.163861942	0.05	1
	IDP dMRI TBSS ICVF Middle cerebellar peduncle	Wald ratio	0.156425206	0.05	1
		MR Egger	0.626251832	0.05	1

	IDP dMRI TBSS ICVF Genu of corpus callosum	Weighted median	0.245532843	0.05	1
		Inverse variance weighted	0.068085494	0.05	1
		Simple mode	0.311228859	0.05	1
		Weighted mode	0.265582059	0.05	1
	IDP dMRI TBSS ICVF Body of corpus callosum	MR Egger	0.626176399	0.05	1
		Weighted median	0.050198478	0.05	1
		Inverse variance weighted	0.024752602	0.05	1
		Simple mode	0.20905129	0.05	1
		Weighted mode	0.189977465	0.05	1
	IDP dMRI TBSS ICVF Splenium of corpus callosum	MR Egger	0.467732457	0.05	1
		Weighted median	0.098065231	0.05	1
		Inverse variance weighted	0.047552231	0.05	1
		Simple mode	0.38022739	0.05	1
		Weighted mode	0.298390903	0.05	1
	IDP dMRI TBSS ICVF Fornix	Wald ratio	0.133166826	0.05	1
	IDP dMRI TBSS ICVF Medial lemniscus R	Inverse variance weighted	0.571775928	0.05	1
	IDP dMRI TBSS ICVF Medial lemniscus L	Wald ratio	0.32230805	0.05	1
	IDP dMRI TBSS ICVF Inferior cerebellar peduncle R	Inverse variance weighted	0.434318299	0.05	1
	IDP dMRI TBSS ICVF Inferior cerebellar peduncle L	Inverse variance weighted	0.422991742	0.05	1
	IDP dMRI TBSS ICVF Superior cerebellar peduncle R	MR Egger	0.817681926	0.05	1
		Weighted median	0.6094559	0.05	1
		Inverse variance weighted	0.703570444	0.05	1

		Simple mode	0.598595871	0.05	1
		Weighted mode	0.537642099	0.05	1
	IDP dMRI TBSS ICVF Superior cerebellar peduncle L	Inverse variance weighted	0.741471964	0.05	1
	IDP dMRI TBSS ICVF Cerebral peduncle R	MR Egger	0.311196581	0.05	1
		Weighted median	0.34064066	0.05	1
		Inverse variance weighted	0.41200194	0.05	1
		Simple mode	0.511909966	0.05	1
		Weighted mode	0.442648575	0.05	1
	IDP dMRI TBSS ICVF Cerebral peduncle L	Wald ratio	0.691663905	0.05	1
	IDP dMRI TBSS ICVF Anterior limb of internal capsule R	Wald ratio	0.394986168	0.05	1
	IDP dMRI TBSS ICVF Anterior limb of internal capsule L	MR Egger	0.144759354	0.05	1
		Weighted median	0.603286572	0.05	1
		Inverse variance weighted	0.832694203	0.05	1
		Simple mode	0.670900801	0.05	1
		Weighted mode	0.597546734	0.05	1
	IDP dMRI TBSS ICVF Posterior limb of internal capsule R	MR Egger	0.707953221	0.05	1
		Weighted median	0.054383517	0.05	1
		Inverse variance weighted	0.055118205	0.05	1
		Simple mode	0.243549318	0.05	1
		Weighted mode	0.245671529	0.05	1
	IDP dMRI TBSS ICVF Posterior limb of internal capsule L	MR Egger	0.989627186	0.05	1
		Weighted median	0.307495553	0.05	1

		Inverse variance weighted	0.123548156	0.05	1
		Simple mode	0.516205625	0.05	1
		Weighted mode	0.475665565	0.05	1
	IDP dMRI TBSS ICVF Retrolenticular part of internal capsule R	MR Egger	0.848188972	0.05	1
		Weighted median	0.199294859	0.05	1
		Inverse variance weighted	0.073820013	0.05	1
		Simple mode	0.366115436	0.05	1
		Weighted mode	0.398676497	0.05	1
	IDP dMRI TBSS ICVF Retrolenticular part of internal capsule L	MR Egger	0.961193336	0.05	1
		Weighted median	0.25602012	0.05	1
		Inverse variance weighted	0.126939659	0.05	1
		Simple mode	0.520397874	0.05	1
		Weighted mode	0.477336318	0.05	1
	IDP dMRI TBSS ICVF Anterior corona radiata R	MR Egger	0.950695148	0.05	1
		Weighted median	0.283280316	0.05	1
		Inverse variance weighted	0.119284238	0.05	1
		Simple mode	0.506094909	0.05	1
		Weighted mode	0.476783853	0.05	1
	IDP dMRI TBSS ICVF Anterior corona radiata L	MR Egger	0.979960306	0.05	1
		Weighted median	0.272163197	0.05	1
		Inverse variance weighted	0.125881166	0.05	1
		Simple mode	0.500761413	0.05	1

		Weighted mode	0.484059348	0.05	1
	IDP dMRI TBSS ICVF Superior corona radiata R	MR Egger	0.917129802	0.05	1
		Weighted median	0.259772059	0.05	1
		Inverse variance weighted	0.114287212	0.05	1
		Simple mode	0.486533567	0.05	1
		Weighted mode	0.506419644	0.05	1
	IDP dMRI TBSS ICVF Superior corona radiata L	MR Egger	0.938389835	0.05	1
		Weighted median	0.269266391	0.05	1
		Inverse variance weighted	0.117514031	0.05	1
		Simple mode	0.481653453	0.05	1
		Weighted mode	0.476975877	0.05	1
	IDP dMRI TBSS ICVF Posterior corona radiata R	MR Egger	0.843991069	0.05	1
		Weighted median	0.094545272	0.05	1
		Inverse variance weighted	0.064642214	0.05	1
		Simple mode	0.211277603	0.05	1
		Weighted mode	0.44578704	0.05	1
	IDP dMRI TBSS ICVF Posterior corona radiata L	MR Egger	0.857598493	0.05	1
		Weighted median	0.075262015	0.05	1
		Inverse variance weighted	0.067388561	0.05	1
		Simple mode	0.203711816	0.05	1
		Weighted mode	0.452265161	0.05	1
	IDP dMRI TBSS ICVF Posterior thalamic radiation R	Wald ratio	0.156425206	0.05	1

	IDP dMRI TBSS ICVF Posterior thalamic radiation L	Inverse variance weighted	0.122598219	0.05	1
	IDP dMRI TBSS ICVF Sagittal stratum R	MR Egger	0.97678796	0.05	1
		Weighted median	0.10913195	0.05	1
		Inverse variance weighted	0.213898309	0.05	1
		Simple mode	0.317590614	0.05	1
		Weighted mode	0.300763253	0.05	1
	IDP dMRI TBSS ICVF Sagittal stratum L	Inverse variance weighted	0.085084142	0.05	1
	IDP dMRI TBSS ICVF External capsule R	MR Egger	0.997798765	0.05	1
		Weighted median	0.413281675	0.05	1
		Inverse variance weighted	0.378701165	0.05	1
		Simple mode	0.445894332	0.05	1
		Weighted mode	0.486822365	0.05	1
	IDP dMRI TBSS ICVF External capsule L	Inverse variance weighted	0.673263307	0.05	1
	IDP dMRI TBSS ICVF Cingulum cingulate gyrus R	Inverse variance weighted	0.157784955	0.05	1
	IDP dMRI TBSS ICVF Cingulum cingulate gyrus L	Wald ratio	0.233240472	0.05	1
	IDP dMRI TBSS ICVF Cingulum hippocampus R	MR Egger	0.73688641	0.05	1
		Weighted median	0.323200043	0.05	1
		Inverse variance weighted	0.352988989	0.05	1
		Simple mode	0.40055315	0.05	1
		Weighted mode	0.442694398	0.05	1
		MR Egger	0.981072048	0.05	1

	IDP dMRI TBSS ICVF Cingulum hippocampus L	Weighted median	0.24737788	0.05	1
		Inverse variance weighted	0.13539609	0.05	1
		Simple mode	0.487095104	0.05	1
		Weighted mode	0.458639216	0.05	1
	IDP dMRI TBSS ICVF Fornix cres+Stria terminalis R	Wald ratio	0.994553731	0.05	1
	IDP dMRI TBSS ICVF Superior longitudinal fasciculus R	Inverse variance weighted	0.146563708	0.05	1
	IDP dMRI TBSS ICVF Superior longitudinal fasciculus L	MR Egger	0.88040671	0.05	1
		Weighted median	0.236081728	0.05	1
		Inverse variance weighted	0.11153192	0.05	1
		Simple mode	0.485211302	0.05	1
		Weighted mode	0.479703519	0.05	1
	IDP dMRI TBSS ICVF Superior fronto-occipital fasciculus R	Inverse variance weighted	0.954556793	0.05	1
	IDP dMRI TBSS ICVF Uncinate fasciculus R	Wald ratio	0.394986168	0.05	1
	IDP dMRI TBSS ICVF Uncinate fasciculus L	Wald ratio	0.394986168	0.05	1
	IDP dMRI TBSS OD Pontine crossing tract	MR Egger	0.619534958	0.05	0.999999999
		Weighted median	0.04942218	0.05	1
		Inverse variance weighted	0.018508711	0.05	1
		Simple mode	0.250264605	0.05	1
		Weighted mode	0.250506896	0.05	1
	IDP dMRI TBSS OD Cerebral peduncle R	Wald ratio	0.42520086	0.05	1
	IDP dMRI TBSS OD Cerebral peduncle L	Wald ratio	0.42520086	0.05	1
	IDP dMRI TBSS OD Posterior limb of internal capsule L	Wald ratio	0.061088793	0.05	1

	IDP dMRI TBSS OD External capsule R	Inverse variance weighted	0.790536945	0.05	1
	IDP dMRI TBSS OD External capsule L	Wald ratio	0.509409009	0.05	1
	IDP dMRI TBSS OD Superior longitudinal fasciculus R	Wald ratio	0.6866473	0.05	1
	IDP dMRI TBSS ISOVF Fornix	Wald ratio	0.385887017	0.05	1
	IDP dMRI TBSS ISOVF External capsule R	Wald ratio	0.035282992	0.05	1
	IDP dMRI TBSS ISOVF Cingulum cingulate gyrus R	Wald ratio	0.805934027	0.05	1
	IDP dMRI ProbtrackX FA ar l	Wald ratio	0.394986168	0.05	1
	IDP dMRI ProbtrackX FA atr l	Wald ratio	0.394986168	0.05	1
	IDP dMRI ProbtrackX FA fmi	MR Egger	0.897983198	0.05	1
		Weighted median	0.120873916	0.05	1
		Inverse variance weighted	0.044969255	0.05	1
		Simple mode	0.451136958	0.05	1
		Weighted mode	0.426153607	0.05	1
	IDP dMRI ProbtrackX FA ifo l	Wald ratio	0.156425206	0.05	1
	IDP dMRI ProbtrackX FA ifo r	Wald ratio	0.158295329	0.05	1
	IDP dMRI ProbtrackX FA ml l	Wald ratio	0.140212543	0.05	1
	IDP dMRI ProbtrackX FA ptr r	Wald ratio	0.552284982	0.05	1
	IDP dMRI ProbtrackX FA slf l	Wald ratio	0.159236715	0.05	1
	IDP dMRI ProbtrackX FA str l	Wald ratio	0.902810151	0.05	1
	IDP dMRI ProbtrackX FA unc l	Wald ratio	0.394986168	0.05	1
	IDP dMRI ProbtrackX FA unc r	Wald ratio	0.394986168	0.05	1
	IDP dMRI ProbtrackX MD ar l	Wald ratio	0.394986168	0.05	1

	IDP dMRI ProbtrackX MD ar r	Wald ratio	0.394986168	0.05	1
	IDP dMRI ProbtrackX MD atr l	Wald ratio	0.394986168	0.05	1
	IDP dMRI ProbtrackX MD atr r	Wald ratio	0.394986168	0.05	1
	IDP dMRI ProbtrackX MD cgc l	MR Egger	0.804819325	0.05	1
		Weighted median	0.358668666	0.05	1
		Inverse variance weighted	0.385448327	0.05	1
		Simple mode	0.398343546	0.05	1
		Weighted mode	0.466189648	0.05	1
	IDP dMRI ProbtrackX MD cgc r	Inverse variance weighted	0.683303751	0.05	1
	IDP dMRI ProbtrackX MD fmi	Inverse variance weighted	0.131318613	0.05	1
	IDP dMRI ProbtrackX MD ifo l	Wald ratio	0.156425206	0.05	1
	IDP dMRI ProbtrackX MD ifo r	Wald ratio	0.156425206	0.05	1
	IDP dMRI ProbtrackX MD ilf l	Wald ratio	0.156425206	0.05	1
	IDP dMRI ProbtrackX MD ilf r	Wald ratio	0.156425206	0.05	1
	IDP dMRI ProbtrackX MD ptr l	Wald ratio	0.394986168	0.05	1
	IDP dMRI ProbtrackX MD ptr r	Wald ratio	0.394986168	0.05	1
	IDP dMRI ProbtrackX MD slf l	Inverse variance weighted	0.126727429	0.05	1
	IDP dMRI ProbtrackX MD slf r	Wald ratio	0.156425206	0.05	1
	IDP dMRI ProbtrackX MD str l	Wald ratio	0.87025599	0.05	1
	IDP dMRI ProbtrackX MD str r	Wald ratio	0.394986168	0.05	1
	IDP dMRI ProbtrackX MD unc l	Wald ratio	0.394986168	0.05	1
	IDP dMRI ProbtrackX MD unc r	Inverse variance weighted	0.448541596	0.05	1

	IDP dMRI ProbtrackX MO atr l	Wald ratio	0.714192952	0.05	1
	IDP dMRI ProbtrackX MO ml r	Wald ratio	0.959265543	0.05	1
	IDP T1 FAST ROIs L intracalc cortex	Wald ratio	0.562183051	0.05	1
	IDP dMRI ProbtrackX L1 ilf l	Wald ratio	0.394986168	0.05	1
	IDP T1 FAST ROIs R intracalc cortex	Wald ratio	0.562183051	0.05	1
	IDP dMRI ProbtrackX L1 slf l	Wald ratio	0.394986168	0.05	1
	IDP dMRI ProbtrackX L1 slf r	Wald ratio	0.596888096	0.05	1
	IDP dMRI ProbtrackX L1 str r	Wald ratio	0.216417265	0.05	1
	IDP dMRI ProbtrackX L1 unc l	Inverse variance weighted	0.799890079	0.05	1
	IDP dMRI ProbtrackX L1 unc r	Wald ratio	0.934043472	0.05	1
	IDP dMRI ProbtrackX L2 ar l	Wald ratio	0.394986168	0.05	1
	IDP dMRI ProbtrackX L2 ar r	Wald ratio	0.394986168	0.05	1
	IDP dMRI ProbtrackX L2 atr l	Wald ratio	0.394986168	0.05	1
	IDP dMRI ProbtrackX L2 atr r	Wald ratio	0.394986168	0.05	1
	IDP dMRI ProbtrackX L2 cgh l	Wald ratio	0.31574182	0.05	1
	IDP dMRI ProbtrackX L2 fma	Wald ratio	0.914632259	0.05	1
	IDP dMRI ProbtrackX L2 fmi	Inverse variance weighted	0.130892827	0.05	1
	IDP dMRI ProbtrackX L2 ifo l	Wald ratio	0.156425206	0.05	1
	IDP dMRI ProbtrackX L2 ifo r	Wald ratio	0.156425206	0.05	1
	IDP dMRI ProbtrackX L2 ilf l	Wald ratio	0.156425206	0.05	1
	IDP dMRI ProbtrackX L2 ilf r	Wald ratio	0.156425206	0.05	1
	IDP dMRI ProbtrackX L2 ptr l	Wald ratio	0.394986168	0.05	1
	IDP dMRI ProbtrackX L2 ptr r	Wald ratio	0.336810512	0.05	1

	IDP dMRI ProbtrackX L2 slf l	Inverse variance weighted	0.125618718	0.05	1
	IDP dMRI ProbtrackX L2 slf r	Wald ratio	0.156425206	0.05	1
	IDP dMRI ProbtrackX L2 str l	Wald ratio	0.87025599	0.05	1
	IDP dMRI ProbtrackX L2 str r	Wald ratio	0.87025599	0.05	1
	IDP dMRI ProbtrackX L2 unc l	Wald ratio	0.394986168	0.05	1
	IDP dMRI ProbtrackX L2 unc r	Inverse variance weighted	0.443744247	0.05	1
	IDP dMRI ProbtrackX L3 ar l	Wald ratio	0.394986168	0.05	1
	IDP dMRI ProbtrackX L3 ar r	Wald ratio	0.394986168	0.05	1
	IDP dMRI ProbtrackX L3 atr l	Wald ratio	0.394986168	0.05	1
	IDP dMRI ProbtrackX L3 atr r	Wald ratio	0.394986168	0.05	1
	IDP dMRI ProbtrackX L3 fmi	MR Egger	0.937286941	0.05	1
		Weighted median	0.298669794	0.05	1
		Inverse variance weighted	0.113244169	0.05	1
		Simple mode	0.508796752	0.05	1
		Weighted mode	0.460891254	0.05	1
	IDP dMRI ProbtrackX L3 ifo l	Inverse variance weighted	0.139377751	0.05	1
	IDP dMRI ProbtrackX L3 ifo r	Wald ratio	0.156425206	0.05	1
	IDP dMRI ProbtrackX L3 ilf l	Wald ratio	0.156425206	0.05	1
	IDP dMRI ProbtrackX L3 ilf r	Wald ratio	0.156425206	0.05	1
	IDP dMRI ProbtrackX L3 ptr l	Wald ratio	0.394986168	0.05	1
	IDP dMRI ProbtrackX L3 ptr r	Wald ratio	0.336810512	0.05	1
	IDP dMRI ProbtrackX L3 slf l	Inverse variance weighted	0.128900548	0.05	1

	IDP dMRI ProbtrackX L3 slf r	Wald ratio	0.156425206	0.05	1
	IDP dMRI ProbtrackX L3 str l	Wald ratio	0.336810512	0.05	1
	IDP dMRI ProbtrackX L3 unc l	Wald ratio	0.394986168	0.05	1
	IDP dMRI ProbtrackX L3 unc r	Inverse variance weighted	0.457132688	0.05	1
	IDP dMRI ProbtrackX ICVF ar l	MR Egger	0.980021822	0.05	1
		Weighted median	0.296961625	0.05	1
		Inverse variance weighted	0.171661181	0.05	1
		Simple mode	0.509830538	0.05	1
		Weighted mode	0.453413766	0.05	1
	IDP dMRI ProbtrackX ICVF ar r	MR Egger	0.967006387	0.05	1
		Weighted median	0.290477961	0.05	1
		Inverse variance weighted	0.166290376	0.05	1
		Simple mode	0.491685115	0.05	1
		Weighted mode	0.472940105	0.05	1
	IDP dMRI ProbtrackX ICVF atr l	MR Egger	0.984508513	0.05	1
		Weighted median	0.302079866	0.05	1
		Inverse variance weighted	0.137777172	0.05	1
		Simple mode	0.521947835	0.05	1
		Weighted mode	0.462530628	0.05	1
	IDP dMRI ProbtrackX ICVF atr r	MR Egger	0.977806867	0.05	1
		Weighted median	0.29557787	0.05	1
		Inverse variance weighted	0.135224498	0.05	1

		Simple mode	0.505133627	0.05	1
		Weighted mode	0.472675328	0.05	1
	IDP dMRI ProbtrackX ICVF cgc l	Wald ratio	0.233240472	0.05	1
	IDP dMRI ProbtrackX ICVF cgh l	MR Egger	0.976380496	0.05	1
		Weighted median	0.277021082	0.05	1
		Inverse variance weighted	0.139377631	0.05	1
		Simple mode	0.508296165	0.05	1
		Weighted mode	0.468598277	0.05	1
	IDP dMRI ProbtrackX ICVF cgh r	Wald ratio	0.394986168	0.05	1
	IDP dMRI ProbtrackX ICVF cst l	MR Egger	0.969299721	0.05	1
		Weighted median	0.299131757	0.05	1
		Inverse variance weighted	0.1553245	0.05	1
		Simple mode	0.489499064	0.05	1
		Weighted mode	0.477308047	0.05	1
	IDP dMRI ProbtrackX ICVF cst r	MR Egger	0.876070677	0.05	1
		Weighted median	0.272615853	0.05	1
		Inverse variance weighted	0.09287267	0.05	1
		Simple mode	0.454325455	0.05	1
		Weighted mode	0.434504504	0.05	1
	IDP dMRI ProbtrackX ICVF fma	Inverse variance weighted	0.05224614	0.05	1
	IDP dMRI ProbtrackX ICVF fmi	MR Egger	0.962949098	0.05	1
		Weighted median	0.240310769	0.05	1

		Inverse variance weighted	0.051225658	0.05	1
		Simple mode	0.146743317	0.05	1
		Weighted mode	0.422068382	0.05	1
	IDP dMRI ProbtrackX ICVF ifo l	Inverse variance weighted	0.151803359	0.05	1
	IDP dMRI ProbtrackX ICVF ifo r	Inverse variance weighted	0.150575251	0.05	1
	IDP dMRI ProbtrackX ICVF ilf l	Inverse variance weighted	0.146884843	0.05	1
	IDP dMRI ProbtrackX ICVF ilf r	Wald ratio	0.156425206	0.05	1
	IDP dMRI ProbtrackX ICVF mcp	Wald ratio	0.168341952	0.05	1
	IDP dMRI ProbtrackX ICVF ml l	Wald ratio	0.548087164	0.05	1
	IDP dMRI ProbtrackX ICVF ml r	Wald ratio	0.394986168	0.05	1
	IDP dMRI ProbtrackX ICVF ptr l	Wald ratio	0.156425206	0.05	1
	IDP dMRI ProbtrackX ICVF ptr r	Inverse variance weighted	0.084370583	0.05	1
	IDP dMRI ProbtrackX ICVF slf l	MR Egger	0.936021736	0.05	1
		Weighted median	0.256103169	0.05	1
		Inverse variance weighted	0.119187547	0.05	1
		Simple mode	0.488218814	0.05	1
		Weighted mode	0.443859708	0.05	1
	IDP dMRI ProbtrackX ICVF slf r	Inverse variance weighted	0.148262518	0.05	1
	IDP dMRI ProbtrackX ICVF str l	MR Egger	0.997530218	0.05	1
		Weighted median	0.297854723	0.05	1
		Inverse variance weighted	0.123845562	0.05	1

		Simple mode	0.501067927	0.05	1
		Weighted mode	0.428157359	0.05	1
	IDP dMRI ProbtrackX ICVF str r	MR Egger	0.959056929	0.05	1
		Weighted median	0.288097413	0.05	1
		Inverse variance weighted	0.119986445	0.05	1
		Simple mode	0.499362537	0.05	1
		Weighted mode	0.452522663	0.05	1
	IDP dMRI ProbtrackX ICVF unc l	Inverse variance weighted	0.32402885	0.05	1
	IDP dMRI ProbtrackX ICVF unc r	Inverse variance weighted	0.17409573	0.05	1
	IDP dMRI ProbtrackX OD atr r	Wald ratio	0.351430497	0.05	1
	IDP dMRI ProbtrackX OD ilf l	Wald ratio	0.130217429	0.05	1
	IDP dMRI ProbtrackX OD slf l	Wald ratio	0.796712108	0.05	1
	IDP dMRI ProbtrackX OD str l	Inverse variance weighted	0.085360439	0.05	1
	IDP dMRI ProbtrackX ISOVF atr r	Wald ratio	0.394986168	0.05	1
	IDP dMRI ProbtrackX ISOVF cgc l	Wald ratio	0.273011454	0.05	1
	IDP dMRI ProbtrackX ISOVF ifo r	Wald ratio	0.052805521	0.05	1
	IDP dMRI ProbtrackX ISOVF ilf l	Wald ratio	0.387304424	0.05	1
	IDP dMRI ProbtrackX ISOVF ilf r	MR Egger	0.563692866	0.05	0.005458732
		Weighted median	0.7350802	0.05	1
		Inverse variance weighted	0.874075768	0.05	1
		Simple mode	0.7892123	0.05	1
		Weighted mode	0.789612167	0.05	1

	IDP dMRI ProbtrackX ISOVF slf l	Inverse variance weighted	0.68542978	0.05	1
	IDP dMRI ProbtrackX ISOVF slf r	MR Egger	0.929472795	0.05	1
		Weighted median	0.830925663	0.05	1
		Inverse variance weighted	0.808248575	0.05	1
		Simple mode	0.865451576	0.05	1
		Weighted mode	0.851332636	0.05	1
	IDP dMRI ProbtrackX ISOVF unc l	Wald ratio	0.950336879	0.05	1
	IDP dMRI ProbtrackX ISOVF unc r	Wald ratio	0.931301948	0.05	1
	IDP T1 SIENAX brain-normalised volume	Wald ratio	0.986427892	0.05	1
Vasculitis limited to skin, not elsewhere classified	IDP T1 SIENAX peripheral grey normalised volume	Wald ratio	0.290880748	0.05	1
	IDP T1 FAST ROIs R temp occ fusif cortex	Wald ratio	0.50278879	0.05	1
	IDP T1 FAST ROIs R occ pole	Inverse variance weighted	0.057863616	0.05	1
	IDP T1 FAST ROIs L thalamus	Inverse variance weighted	0.22320773	0.05	1
	IDP T1 FAST ROIs R thalamus	Wald ratio	0.997153807	0.05	1
	IDP T1 FAST ROIs L putamen	Wald ratio	0.711733745	0.05	1
	IDP T1 FAST ROIs R putamen	Wald ratio	0.711733745	0.05	1
	IDP T1 FAST ROIs L pallidum	Wald ratio	0.313870209	0.05	0.999998235
	IDP T1 FIRST left caudate volume	Wald ratio	0.873106415	0.05	1
	IDP T1 FAST ROIs L hippocampus	Inverse variance weighted	0.459835108	0.05	1
	IDP T1 FAST ROIs R hippocampus	Wald ratio	0.774182705	0.05	1
	IDP T1 FAST ROIs L ventral striatum	Wald ratio	0.711733745	0.05	1
	IDP T1 FAST ROIs R ventral striatum	Inverse variance weighted	0.343523639	0.05	1

	IDP T1 FAST ROIs L cerebellum VI	Wald ratio	0.970587457	0.05	1
	IDP T1 FAST ROIs L cerebellum crus I	MR Egger	0.666884426	0.05	1
		Weighted median	0.385056147	0.05	1
		Inverse variance weighted	0.438748373	0.05	1
		Simple mode	0.571365978	0.05	1
		Weighted mode	0.522012783	0.05	1
	IDP T1 FAST ROIs R cerebellum crus I	Inverse variance weighted	0.469600898	0.05	1
	IDP T1 FAST ROIs L cerebellum crus II	MR Egger	0.543919633	0.05	0.994475084
		Weighted median	0.089557282	0.05	1
		Inverse variance weighted	0.096272329	0.05	1
		Simple mode	0.301523103	0.05	1
		Weighted mode	0.434967735	0.05	1
	IDP T1 FAST ROIs V cerebellum crus II	Inverse variance weighted	0.691022312	0.05	1
	IDP T1 FAST ROIs R cerebellum crus II	Inverse variance weighted	0.233794556	0.05	1
	IDP T1 FIRST left putamen volume	Inverse variance weighted	0.378982917	0.05	1
	IDP T1 FAST ROIs R cerebellum VIIb	Wald ratio	0.549762422	0.05	1
	IDP T1 FAST ROIs V cerebellum VIIIa	MR Egger	0.511007961	0.05	1
		Weighted median	0.791375192	0.05	1
		Inverse variance weighted	0.567646324	0.05	1
		Simple mode	0.90221227	0.05	1
		Weighted mode	0.869819738	0.05	1
	IDP T1 FAST ROIs L cerebellum VIIIb	Wald ratio	0.794983368	0.05	1

	IDP T1 FAST ROIs V cerebellum VIIIb	Inverse variance weighted	0.882717732	0.05	1
	IDP T1 FAST ROIs R cerebellum VIIIb	Wald ratio	0.711733745	0.05	1
	IDP T1 FAST ROIs L cerebellum IX	Inverse variance weighted	0.365316845	0.05	1
	IDP T1 FIRST right putamen volume	Wald ratio	0.400805986	0.05	1
	IDP T1 FAST ROIs V cerebellum IX	Inverse variance weighted	0.619759446	0.05	1
	IDP T1 FAST ROIs R cerebellum IX	Inverse variance weighted	0.351584956	0.05	1
	IDP T1 FAST ROIs V cerebellum X	Wald ratio	0.711733745	0.05	1
	IDP T2 FLAIR BIANCA WMH volume	Inverse variance weighted	0.316151267	0.05	1
	IDP SWI T2star left caudate	MR Egger	0.981132785	0.05	1
		Weighted median	0.450178193	0.05	1
		Inverse variance weighted	0.316132849	0.05	1
		Simple mode	0.323792424	0.05	1
		Weighted mode	0.627437076	0.05	1
	IDP SWI T2star right caudate	MR Egger	0.879442211	0.05	1
		Weighted median	0.207257113	0.05	1
		Inverse variance weighted	0.254722303	0.05	1
		Simple mode	0.399408376	0.05	1
		Weighted mode	0.346306148	0.05	1
	IDP T1 FIRST left pallidum volume	Wald ratio	0.61913063	0.05	1
	IDP SWI T2star left putamen	MR Egger	0.70118432	0.05	1
		Weighted median	0.85777388	0.05	1

		Inverse variance weighted	0.610991216	0.05	1
		Simple mode	0.334258973	0.05	1
		Weighted mode	0.656192216	0.05	1
	IDP SWI T2star right putamen	MR Egger	0.533164661	0.05	0.999963857
		Weighted median	0.603509486	0.05	1
		Inverse variance weighted	0.334452055	0.05	1
		Simple mode	0.875363165	0.05	1
		Weighted mode	0.868567725	0.05	1
	IDP SWI T2star left pallidum	MR Egger	0.271974966	0.05	1
		Weighted median	0.510898587	0.05	1
		Inverse variance weighted	0.089815552	0.05	1
		Simple mode	0.918333126	0.05	1
		Weighted mode	0.911111254	0.05	1
	IDP SWI T2star right pallidum	MR Egger	0.758799917	0.05	1
		Weighted median	0.808750685	0.05	1
		Inverse variance weighted	0.664944214	0.05	1
		Simple mode	0.922456542	0.05	1
		Weighted mode	0.300593179	0.05	0.999997949
	IDP T1 FIRST right pallidum volume	Wald ratio	0.61913063	0.05	1
	IDP dMRI TBSS FA Genu of corpus callosum	Wald ratio	0.528680752	0.05	1
	IDP dMRI TBSS FA Splenium of corpus callosum	Inverse variance weighted	0.82175469	0.05	1
	IDP dMRI TBSS FA Corticospinal tract L	Wald ratio	0.127421999	0.05	0.999970447

	IDP dMRI TBSS FA Superior cerebellar peduncle R	Inverse variance weighted	3.05E-05	0.05	0.377592826
	IDP dMRI TBSS FA Superior cerebellar peduncle L	Inverse variance weighted	2.83E-05	0.05	0.247716395
	IDP dMRI TBSS FA Posterior limb of internal capsule R	Wald ratio	0.270454452	0.05	1
	IDP dMRI TBSS FA Retrolenticular part of internal capsule R	Inverse variance weighted	0.406177204	0.05	1
	IDP dMRI TBSS FA Anterior corona radiata R	Wald ratio	0.528680752	0.05	1
	IDP dMRI TBSS FA Anterior corona radiata L	Wald ratio	0.528680752	0.05	1
	IDP dMRI TBSS FA Posterior thalamic radiation R	Wald ratio	0.186057544	0.05	1
	IDP dMRI TBSS FA Posterior thalamic radiation L	Wald ratio	0.218820582	0.05	1
	IDP dMRI TBSS FA Sagittal stratum R	Wald ratio	0.068086287	0.05	1
	IDP dMRI TBSS FA Cingulum cingulate gyrus R	Inverse variance weighted	0.997945172	0.05	1
	IDP dMRI TBSS FA Superior longitudinal fasciculus L	Inverse variance weighted	0.260241036	0.05	1
	IDP dMRI TBSS FA Uncinate fasciculus L	Wald ratio	0.603032529	0.05	1
	IDP dMRI TBSS MD Genu of corpus callosum	Wald ratio	0.528680752	0.05	1
	IDP dMRI TBSS MD Body of corpus callosum	Wald ratio	0.948598689	0.05	1
	IDP dMRI TBSS MD Splenium of corpus callosum	Inverse variance weighted	0.937704936	0.05	1
	IDP dMRI TBSS MD Corticospinal tract R	Wald ratio	0.88250456	0.05	1
	IDP dMRI TBSS MD Superior cerebellar peduncle R	Wald ratio	0.00180465	0.05	1
	IDP dMRI TBSS MD Superior cerebellar peduncle L	Wald ratio	0.000529016	0.05	1
	IDP dMRI TBSS MD Anterior limb of internal capsule L	Wald ratio	0.773477678	0.05	1

	IDP dMRI TBSS MD Retrolenticular part of internal capsule R	Wald ratio	0.184961435	0.05	0.999999685
	IDP T1 FIRST left caudate volume plus IDP T1 FIRST right caudate volume	Wald ratio	0.699191646	0.05	1
	IDP T1 FIRST left putamen volume plus IDP T1 FIRST right putamen volume	Inverse variance weighted	0.455857092	0.05	1
	IDP T1 FIRST left pallidum volume plus IDP T1 FIRST right pallidum volume	Wald ratio	0.61913063	0.05	1
	IDP SWI T2star left thalamus plus IDP SWI T2star right thalamus	MR Egger	0.990792744	0.05	1
		Weighted median	0.088712887	0.05	1
		Inverse variance weighted	0.068978531	0.05	1
		Simple mode	0.270390897	0.05	1
		Weighted mode	0.275920462	0.05	1
	IDP dMRI TBSS MD Retrolenticular part of internal capsule L	Wald ratio	0.184961435	0.05	0.999824698
	IDP SWI T2star left caudate plus IDP SWI T2star right caudate	MR Egger	0.628682293	0.05	1
		Weighted median	0.4633476	0.05	1
		Inverse variance weighted	0.34036075	0.05	1
		Simple mode	0.517577026	0.05	1
		Weighted mode	0.40233858	0.05	1
	IDP SWI T2star left putamen plus IDP SWI T2star right putamen	MR Egger	0.561752272	0.05	1
		Weighted median	0.8366236	0.05	1
		Inverse variance weighted	0.664057063	0.05	1
		Simple mode	0.905358781	0.05	1
		Weighted mode	0.974398452	0.05	1
		MR Egger	0.219881708	0.05	1

	IDP SWI T2star left pallidum plus IDP SWI T2star right pallidum	Weighted median	0.89385581	0.05	1
		Inverse variance weighted	0.294635486	0.05	1
		Simple mode	0.838861769	0.05	1
		Weighted mode	0.790947621	0.05	1
	volume Left-Lateral-Ventricle	MR Egger	0.929855701	0.05	1
		Weighted median	0.247308639	0.05	1
		Inverse variance weighted	0.034141659	0.05	1
		Simple mode	0.575902597	0.05	1
		Weighted mode	0.631385028	0.05	1
	volume Left-Cerebellum-White-Matter	Inverse variance weighted	0.769449666	0.05	1
	volume Left-Cerebellum-Cortex	Inverse variance weighted	0.746248504	0.05	1
	IDP dMRI TBSS MD Anterior corona radiata R	Inverse variance weighted	0.835316729	0.05	1
	volume Left-Putamen	Inverse variance weighted	0.237286552	0.05	1
	volume 4th-Ventricle	Inverse variance weighted	0.719657075	0.05	1
	volume Brain-Stem	Wald ratio	0.297075596	0.05	1
	volume Left-Hippocampus	Inverse variance weighted	0.251110434	0.05	1
	volume CSF	MR Egger	0.989089222	0.05	1
		Weighted median	0.037358148	0.05	0.909715971
		Inverse variance weighted	0.030565779	0.05	0.991344179
		Simple mode	0.226378391	0.05	0.778496888
		Weighted mode	0.20357302	0.05	0.778496888

	IDP dMRI TBSS MD Anterior corona radiata L	Inverse variance weighted	0.871237555	0.05	1
	volume Left-Accumbens-area	Wald ratio	0.711733745	0.05	1
	volume Right-Lateral-Ventricle	Inverse variance weighted	0.390713136	0.05	1
	volume Right-Inf-Lat-Vent	Wald ratio	0.8030099	0.05	1
	volume Right-Cerebellum-White-Matter	Wald ratio	0.48935851	0.05	1
	volume Right-Cerebellum-Cortex	MR Egger	0.719348815	0.05	1
		Weighted median	0.414493507	0.05	1
		Inverse variance weighted	0.448553713	0.05	1
		Simple mode	0.494819841	0.05	1
		Weighted mode	0.505236666	0.05	1
	IDP dMRI TBSS MD Superior corona radiata R	Inverse variance weighted	0.836031767	0.05	1
	volume Right-Putamen	Wald ratio	0.400805986	0.05	1
	volume Right-Hippocampus	Wald ratio	0.774182705	0.05	1
	volume Right-Amygdala	Wald ratio	0.567521558	0.05	0.999999999
	volume Right-VentralDC	Wald ratio	0.302264033	0.05	1
	volume CC Posterior	Wald ratio	0.131494317	0.05	1
	IDP dMRI TBSS MD Superior corona radiata L	Inverse variance weighted	0.839869194	0.05	1
	IDP dMRI TBSS MD Posterior corona radiata R	Wald ratio	0.184961435	0.05	0.99680274
	volume BrainSegVol-to-eTIV	Wald ratio	0.187738805	0.05	0.999999275
	IDP dMRI TBSS MD Posterior corona radiata L	Wald ratio	0.218820582	0.05	0.999837013
	volume rhSurfaceHoles	Wald ratio	0.757637471	0.05	1
	DKTatlas lh cuneus area	Wald ratio	0.914772678	0.05	1

	IDP dMRI TBSS MD Posterior thalamic radiation R	Wald ratio	0.218820582	0.05	0.999998151
	DKTatlas lh lateraloccipital area	Wald ratio	0.914772678	0.05	1
	DKTatlas lh lingual area	Wald ratio	0.998627886	0.05	1
	IDP dMRI TBSS MD Posterior thalamic radiation L	Wald ratio	0.184961435	0.05	0.999854138
	DKTatlas lh parstriangularis area	Wald ratio	0.928933951	0.05	1
	DKTatlas lh pericalcarine area	Inverse variance weighted	0.498199486	0.05	1
	DKTatlas lh postcentral area	Inverse variance weighted	0.965003859	0.05	1
	DKTatlas lh posteriorcingulate area	Wald ratio	0.564168491	0.05	1
	DKTatlas lh precentral area	Wald ratio	0.605516749	0.05	1
	DKTatlas lh precuneus area	Wald ratio	0.514908272	0.05	1
	IDP dMRI TBSS MD Sagittal stratum R	Wald ratio	0.184961435	0.05	0.999997287
	DKTatlas lh superiorparietal area	Wald ratio	0.53430433	0.05	1
	DKTatlas lh superiortemporal area	Wald ratio	0.997707231	0.05	1
	DKTatlas lh supramarginal area	Wald ratio	0.811974348	0.05	1
	DKTatlas lh WhiteSurfArea area	Wald ratio	0.666864232	0.05	1
	IDP dMRI TBSS MD Sagittal stratum L	Wald ratio	0.184961435	0.05	0.999967708
	a2009s lh G&S subcentral area	Wald ratio	0.605516749	0.05	1
	a2009s lh G cuneus area	Wald ratio	0.870433043	0.05	1
	a2009s lh G front inf-Opercular area	Wald ratio	0.531044459	0.05	1
	IDP dMRI TBSS MD External capsule R	Wald ratio	0.528680752	0.05	1
	IDP dMRI TBSS MD External capsule L	Inverse variance weighted	0.746066724	0.05	1
	a2009s lh G pariet inf-Supramar area	Wald ratio	0.811974348	0.05	1

	a2009s lh G parietal sup area	Wald ratio	0.534771604	0.05	1
	a2009s lh G postcentral area	Wald ratio	0.537290513	0.05	1
	a2009s lh G precentral area	Wald ratio	0.605516749	0.05	1
	a2009s lh G precuneus area	Wald ratio	0.870433043	0.05	1
	IDP dMRI TBSS MD Cingulum cingulate gyrus R	MR Egger	0.403751862	0.05	1
		Weighted median	0.984221565	0.05	1
		Inverse variance weighted	0.755947815	0.05	1
		Simple mode	0.890619457	0.05	1
		Weighted mode	0.725810046	0.05	1
	IDP dMRI TBSS MD Cingulum cingulate gyrus L	MR Egger	0.40248578	0.05	1
		Weighted median	0.989329336	0.05	1
		Inverse variance weighted	0.758090973	0.05	1
		Simple mode	0.899845597	0.05	1
		Weighted mode	0.739563214	0.05	1
	a2009s lh S calcarine area	Wald ratio	0.998627886	0.05	1
	a2009s lh S central area	Wald ratio	0.602163924	0.05	1
	a2009s lh S collat transv ant area	Wald ratio	0.401838671	0.05	1
	a2009s lh S front middle area	Wald ratio	0.338081622	0.05	1
	IDP dMRI TBSS MD Cingulum hippocampus R	Wald ratio	0.184961435	0.05	0.999965886
	a2009s lh S intrapariet&P trans area	Wald ratio	0.534771604	0.05	1
	IDP dMRI TBSS MD Cingulum hippocampus L	Wald ratio	0.184961435	0.05	0.999963963
	a2009s lh S subparietal area	Wald ratio	0.998627886	0.05	1

	DKTatlas rh cuneus area	Wald ratio	0.998627886	0.05	1
	DKTatlas rh lingual area	Wald ratio	1	0.05	1
	DKTatlas rh parstriangularis area	Wald ratio	0.935960922	0.05	1
	DKTatlas rh pericalcarine area	Inverse variance weighted	0.944382408	0.05	1
	IDP dMRI TBSS MD Superior longitudinal fasciculus R	Wald ratio	0.184961435	0.05	0.999989672
	DKTatlas rh postcentral area	Wald ratio	0.602163924	0.05	1
	DKTatlas rh precentral area	Wald ratio	0.605516749	0.05	1
	IDP dMRI TBSS MD Superior longitudinal fasciculus L	Wald ratio	0.184961435	0.05	0.999997208
	a2009s rh G&S subcentral area	Wald ratio	0.602163924	0.05	1
	a2009s rh G&S cingul-Mid-Post area	Wald ratio	0.227887301	0.05	0.996892481
	a2009s rh G cuneus area	Wald ratio	0.998627886	0.05	1
	IDP dMRI TBSS MD Superior fronto-occipital fasciculus L	Wald ratio	0.001442867	0.05	1
	a2009s rh G oc-temp med-Lingual area	Wald ratio	1	0.05	1
	a2009s rh G parietal sup area	Wald ratio	0.487302872	0.05	1
	IDP dMRI TBSS MD Uncinate fasciculus R	Wald ratio	0.778518657	0.05	1
	a2009s rh G precentral area	Wald ratio	0.605516749	0.05	1
	a2009s rh G precuneus area	Wald ratio	0.929191139	0.05	1
	IDP dMRI TBSS MD Uncinate fasciculus L	Inverse variance weighted	0.698366534	0.05	1
	a2009s rh Pole occipital area	Wald ratio	0.998627886	0.05	1
	a2009s rh S calcarine area	Wald ratio	1	0.05	1
	a2009s rh S central area	Wald ratio	0.605516749	0.05	1
	a2009s rh S collat transv ant area	Wald ratio	0.401838671	0.05	1

	a2009s rh S orbital med-olfact area	Wald ratio	0.122118788	0.05	0.789906365
	IDP dMRI TBSS MO Pontine crossing tract	MR Egger	0.414125847	0.05	1
		Weighted median	0.206009443	0.05	1
		Inverse variance weighted	0.731741618	0.05	1
		Simple mode	0.451051748	0.05	1
		Weighted mode	0.282064186	0.05	1
	DKTatlas lh postcentral thickness	Wald ratio	0.602163924	0.05	1
	a2009s lh G insular short thickness	Wald ratio	0.162614097	0.05	0.922864931
	a2009s lh G postcentral thickness	Wald ratio	0.602163924	0.05	1
	IDP T1 SIENAX CSF normalised volume	Inverse variance weighted	0.004387794	0.05	1
	IDP dMRI TBSS MO Medial lemniscus R	Wald ratio	0.500165007	0.05	1
	a2009s lh S postcentral thickness	Wald ratio	0.736368194	0.05	1
	DKTatlas rh lateraloccipital thickness	Wald ratio	0.711733745	0.05	1
	DKTatlas rh parstriangularis thickness	Wald ratio	0.999676839	0.05	1
	DKTatlas rh postcentral thickness	Wald ratio	0.602163924	0.05	1
	DKTatlas rh posteriorcingulate thickness	Inverse variance weighted	0.977842599	0.05	1
	a2009s rh G&S cingul-Mid-Ant thickness	Wald ratio	0.136487768	0.05	1
	a2009s rh G cuneus thickness	Wald ratio	0.711733745	0.05	1
	a2009s rh G postcentral thickness	Wald ratio	0.602163924	0.05	1
	a2009s rh Pole occipital thickness	Wald ratio	0.711733745	0.05	1
	a2009s rh S oc sup&transversal thickness	Wald ratio	0.776649535	0.05	1
	a2009s rh S parieto occipital thickness	Wald ratio	0.869884148	0.05	1

	IDP dMRI TBSS MO Anterior corona radiata R	Wald ratio	0.570350384	0.05	1
	IDP dMRI TBSS MO Superior corona radiata R	Wald ratio	0.323622521	0.05	0.999999856
	IDP dMRI TBSS MO Cingulum cingulate gyrus L	Inverse variance weighted	0.635637372	0.05	1
	IDP dMRI TBSS L1 Anterior limb of internal capsule L	Wald ratio	0.773477678	0.05	1
	IDP dMRI TBSS L1 Posterior limb of internal capsule R	Wald ratio	0.044284785	0.05	0.581409494
	IDP dMRI TBSS L1 Posterior limb of internal capsule L	Wald ratio	0.047855687	0.05	0.797222592
	IDP dMRI TBSS L1 Anterior corona radiata R	Wald ratio	0.528680752	0.05	1
	IDP dMRI TBSS L1 Anterior corona radiata L	Wald ratio	0.528680752	0.05	1
	IDP dMRI TBSS L1 Posterior corona radiata R	Wald ratio	0.710356894	0.05	1
	IDP dMRI TBSS L1 Posterior corona radiata L	Wald ratio	0.528680752	0.05	1
	IDP dMRI TBSS L1 Sagittal stratum L	Wald ratio	0.528680752	0.05	1
	IDP dMRI TBSS L1 External capsule R	Wald ratio	0.528680752	0.05	1
	IDP dMRI TBSS L1 External capsule L	Inverse variance weighted	0.984765486	0.05	1
	IDP dMRI TBSS L1 Fornix cres+Stria terminalis L	Wald ratio	0.339021808	0.05	0.999999544
	IDP T1 FAST ROIs L precentral gyrus	Wald ratio	0.602163924	0.05	1
	IDP dMRI TBSS L1 Superior longitudinal fasciculus R	Inverse variance weighted	0.156826944	0.05	0.99986196
	IDP dMRI TBSS L1 Uncinate fasciculus L	Wald ratio	0.985209094	0.05	1
	IDP dMRI TBSS L2 Pontine crossing tract	Wald ratio	0.500165007	0.05	1
	IDP dMRI TBSS L2 Genu of corpus callosum	MR Egger	0.996514479	0.05	1
		Weighted median	0.378924567	0.05	1

		Inverse variance weighted	0.269723928	0.05	1
		Simple mode	0.543665892	0.05	1
		Weighted mode	0.535531825	0.05	1
	IDP dMRI TBSS L2 Body of corpus callosum	Wald ratio	0.528680752	0.05	1
	IDP dMRI TBSS L2 Splenium of corpus callosum	MR Egger	0.486015058	0.05	1
		Weighted median	0.126555276	0.05	1
		Inverse variance weighted	0.342515916	0.05	1
		Simple mode	0.286889865	0.05	0.999998321
		Weighted mode	0.351165791	0.05	0.999998321
	IDP dMRI TBSS L2 Corticospinal tract R	Wald ratio	0.88250456	0.05	1
	IDP T1 SIENAX CSF unnormalised volume	MR Egger	0.800934906	0.05	1
		Weighted median	0.191981489	0.05	1
		Inverse variance weighted	0.023660087	0.05	1
		Simple mode	0.586331692	0.05	1
		Weighted mode	0.652666711	0.05	1
	IDP T1 FAST ROIs L temporal pole	Wald ratio	0.287094952	0.05	1
	IDP dMRI TBSS L2 Superior cerebellar peduncle R	Inverse variance weighted	2.97E-05	0.05	1
	IDP dMRI TBSS L2 Superior cerebellar peduncle L	Wald ratio	0.000529016	0.05	1
	IDP dMRI TBSS L2 Posterior limb of internal capsule R	Inverse variance weighted	0.74262248	0.05	1
	IDP dMRI TBSS L2 Posterior limb of internal capsule L	Wald ratio	0.587404621	0.05	1
	IDP dMRI TBSS L2 Retrolenticular part of internal capsule R	Inverse variance weighted	0.747479268	0.05	1

	IDP dMRI TBSS L2 Anterior corona radiata R	Inverse variance weighted	0.840938481	0.05	1
	IDP dMRI TBSS L2 Anterior corona radiata L	Wald ratio	0.528680752	0.05	1
	IDP dMRI TBSS L2 Superior corona radiata R	Inverse variance weighted	0.225203381	0.05	1
	IDP dMRI TBSS L2 Superior corona radiata L	Wald ratio	0.184961435	0.05	0.999760383
	IDP dMRI TBSS L2 Posterior corona radiata R	Wald ratio	0.218820582	0.05	0.999999419
	IDP dMRI TBSS L2 Posterior corona radiata L	Wald ratio	0.218820582	0.05	0.999999205
	IDP dMRI TBSS L2 Posterior thalamic radiation R	Wald ratio	0.186057544	0.05	0.999924439
	IDP dMRI TBSS L2 Posterior thalamic radiation L	Wald ratio	0.218820582	0.05	0.999990852
	IDP dMRI TBSS L2 Sagittal stratum R	Wald ratio	0.184961435	0.05	0.999911925
	IDP dMRI TBSS L2 Cingulum cingulate gyrus R	Wald ratio	0.528680752	0.05	1
	IDP dMRI TBSS L2 Cingulum hippocampus R	Wald ratio	0.184961435	0.05	0.999805377
	IDP dMRI TBSS L2 Cingulum hippocampus L	Wald ratio	0.528680752	0.05	1
	IDP dMRI TBSS L2 Uncinate fasciculus L	Wald ratio	0.528680752	0.05	1
	IDP dMRI TBSS L3 Genu of corpus callosum	Wald ratio	0.528680752	0.05	1
	IDP dMRI TBSS L3 Body of corpus callosum	Wald ratio	0.528680752	0.05	1
	IDP dMRI TBSS L3 Splenium of corpus callosum	Wald ratio	0.578174069	0.05	1
	IDP dMRI TBSS L3 Inferior cerebellar peduncle R	Wald ratio	0.528680752	0.05	1
	IDP dMRI TBSS L3 Inferior cerebellar peduncle L	Wald ratio	0.528680752	0.05	1
	IDP dMRI TBSS L3 Superior cerebellar peduncle R	Inverse variance weighted	2.88E-05	0.05	1

	IDP dMRI TBSS L3 Superior cerebellar peduncle L	Wald ratio	0.000529016	0.05	1
	IDP dMRI TBSS L3 Cerebral peduncle R	Wald ratio	0.782805955	0.05	1
	IDP dMRI TBSS L3 Anterior limb of internal capsule R	Wald ratio	0.937448563	0.05	1
	IDP dMRI TBSS L3 Anterior limb of internal capsule L	Wald ratio	0.872838694	0.05	1
	IDP dMRI TBSS L3 Retrolenticular part of internal capsule R	Wald ratio	0.184961435	0.05	0.999998079
	IDP dMRI TBSS L3 Anterior corona radiata R	Inverse variance weighted	0.839176704	0.05	1
	IDP dMRI TBSS L3 Anterior corona radiata L	Inverse variance weighted	0.879529495	0.05	1
	IDP dMRI TBSS L3 Posterior corona radiata R	Inverse variance weighted	0.391672565	0.05	1
	IDP dMRI TBSS L3 Posterior thalamic radiation R	Wald ratio	0.186057544	0.05	0.999946853
	IDP dMRI TBSS L3 Posterior thalamic radiation L	Wald ratio	0.184961435	0.05	0.999850246
	IDP dMRI TBSS L3 Sagittal stratum R	Wald ratio	0.184961435	0.05	0.999988115
	IDP dMRI TBSS L3 Sagittal stratum L	Wald ratio	0.184961435	0.05	0.999865235
	IDP dMRI TBSS L3 External capsule R	Wald ratio	0.528680752	0.05	1
	IDP dMRI TBSS L3 External capsule L	Wald ratio	0.528680752	0.05	1
	IDP dMRI TBSS L3 Cingulum cingulate gyrus R	Inverse variance weighted	0.86672273	0.05	1
	IDP dMRI TBSS L3 Cingulum cingulate gyrus L	Wald ratio	0.528680752	0.05	1
	IDP dMRI TBSS L3 Cingulum hippocampus R	Wald ratio	0.528680752	0.05	1
	IDP dMRI TBSS L3 Cingulum hippocampus L	Wald ratio	0.528680752	0.05	1
	IDP dMRI TBSS L3 Fornix cres+Stria terminalis R	Wald ratio	0.960627429	0.05	1

	IDP dMRI TBSS L3 Superior longitudinal fasciculus R	Wald ratio	0.184961435	0.05	0.999991986
	IDP dMRI TBSS L3 Superior longitudinal fasciculus L	Wald ratio	0.184961435	0.05	0.999998892
	IDP dMRI TBSS L3 Uncinate fasciculus R	Wald ratio	0.528680752	0.05	1
	IDP dMRI TBSS L3 Uncinate fasciculus L	Inverse variance weighted	0.886043171	0.05	1
	IDP dMRI TBSS ICVF Middle cerebellar peduncle	Wald ratio	0.184961435	0.05	1
	IDP dMRI TBSS ICVF Genu of corpus callosum	MR Egger	0.410815445	0.05	0.999863181
		Weighted median	0.379688004	0.05	1
		Inverse variance weighted	0.700343628	0.05	1
		Simple mode	0.439172707	0.05	1
		Weighted mode	0.454873053	0.05	1
	IDP dMRI TBSS ICVF Body of corpus callosum	MR Egger	0.864419012	0.05	1
		Weighted median	0.636403418	0.05	1
		Inverse variance weighted	0.94588481	0.05	1
		Simple mode	0.618863858	0.05	1
		Weighted mode	0.638951483	0.05	1
	IDP dMRI TBSS ICVF Splenium of corpus callosum	MR Egger	0.446951406	0.05	0.18375811
		Weighted median	0.745851372	0.05	1
		Inverse variance weighted	0.735866013	0.05	1
		Simple mode	0.658068124	0.05	1
		Weighted mode	0.574624018	0.05	1
	IDP dMRI TBSS ICVF Fornix	Wald ratio	0.001442867	0.05	0.056663614

	IDP dMRI TBSS ICVF Medial lemniscus R	Inverse variance weighted	0.713590329	0.05	1
	IDP dMRI TBSS ICVF Medial lemniscus L	Wald ratio	0.512829743	0.05	1
	IDP dMRI TBSS ICVF Inferior cerebellar peduncle R	Inverse variance weighted	0.923200696	0.05	1
	IDP dMRI TBSS ICVF Inferior cerebellar peduncle L	Inverse variance weighted	0.91362229	0.05	1
	IDP dMRI TBSS ICVF Superior cerebellar peduncle R	MR Egger	0.284664463	0.05	0.003319239
		Weighted median	0.220334168	0.05	1
		Inverse variance weighted	0.235115851	0.05	0.999995747
		Simple mode	0.94531648	0.05	1
		Weighted mode	0.764201692	0.05	1
	IDP dMRI TBSS ICVF Superior cerebellar peduncle L	Inverse variance weighted	0.065388192	0.05	0.971494689
	IDP dMRI TBSS ICVF Cerebral peduncle R	MR Egger	0.357881764	0.05	0.046069329
		Weighted median	0.61997481	0.05	1
		Inverse variance weighted	0.281735908	0.05	1
		Simple mode	0.923511682	0.05	1
		Weighted mode	0.804566992	0.05	1
	IDP dMRI TBSS ICVF Cerebral peduncle L	Wald ratio	0.794983368	0.05	1
	IDP dMRI TBSS ICVF Anterior limb of internal capsule R	Wald ratio	0.528680752	0.05	1
	IDP dMRI TBSS ICVF Anterior limb of internal capsule L	MR Egger	0.619420876	0.05	0.626789985
		Weighted median	0.741477038	0.05	1
		Inverse variance weighted	0.681285711	0.05	1
		Simple mode	0.936337811	0.05	1

		Weighted mode	0.651903875	0.05	1
	IDP dMRI TBSS ICVF Posterior limb of internal capsule R	MR Egger	0.351096366	0.05	1
		Weighted median	0.428878788	0.05	1
		Inverse variance weighted	0.991377679	0.05	1
		Simple mode	0.532352534	0.05	1
		Weighted mode	0.534958411	0.05	1
	IDP dMRI TBSS ICVF Posterior limb of internal capsule L	MR Egger	0.74615672	0.05	0.999999822
		Weighted median	0.533147476	0.05	1
		Inverse variance weighted	0.865327332	0.05	1
		Simple mode	0.49764303	0.05	1
		Weighted mode	0.501754276	0.05	1
	IDP dMRI TBSS ICVF Retrolenticular part of internal capsule R	MR Egger	0.769897131	0.05	1
		Weighted median	0.920917439	0.05	1
		Inverse variance weighted	0.969799426	0.05	1
		Simple mode	0.538080279	0.05	1
		Weighted mode	0.696357194	0.05	1
	IDP dMRI TBSS ICVF Retrolenticular part of internal capsule L	MR Egger	0.823069135	0.05	1
		Weighted median	0.678340917	0.05	1
		Inverse variance weighted	0.878642191	0.05	1
		Simple mode	0.489218716	0.05	1
		Weighted mode	0.516345413	0.05	1
		MR Egger	0.839628543	0.05	1

	IDP dMRI TBSS ICVF Anterior corona radiata R	Weighted median	0.673750397	0.05	1
		Inverse variance weighted	0.903590365	0.05	1
		Simple mode	0.466033055	0.05	1
		Weighted mode	0.539205332	0.05	1
	IDP dMRI TBSS ICVF Anterior corona radiata L	MR Egger	0.793639653	0.05	1
		Weighted median	0.614907285	0.05	1
		Inverse variance weighted	0.870452578	0.05	1
		Simple mode	0.498993582	0.05	1
		Weighted mode	0.525600482	0.05	1
	IDP dMRI TBSS ICVF Superior corona radiata R	MR Egger	0.893326714	0.05	1
		Weighted median	0.734553907	0.05	1
		Inverse variance weighted	0.934372932	0.05	1
		Simple mode	0.483295457	0.05	1
		Weighted mode	0.544907278	0.05	1
	IDP dMRI TBSS ICVF Superior corona radiata L	MR Egger	0.859162249	0.05	1
		Weighted median	0.699610473	0.05	1
		Inverse variance weighted	0.914728432	0.05	1
		Simple mode	0.49385364	0.05	1
		Weighted mode	0.554878758	0.05	1
	IDP dMRI TBSS ICVF Posterior corona radiata R	MR Egger	0.471102706	0.05	0.81060595
		Weighted median	0.931384452	0.05	1
		Inverse variance weighted	0.744745246	0.05	1

		Simple mode	0.772182864	0.05	1
		Weighted mode	0.76117941	0.05	1
	IDP dMRI TBSS ICVF Posterior corona radiata L	MR Egger	0.491664626	0.05	0.987486285
		Weighted median	0.937006236	0.05	1
		Inverse variance weighted	0.759386429	0.05	1
		Simple mode	0.698086698	0.05	1
		Weighted mode	0.723801163	0.05	1
	IDP dMRI TBSS ICVF Posterior thalamic radiation R	Wald ratio	0.184961435	0.05	1
	IDP dMRI TBSS ICVF Posterior thalamic radiation L	Inverse variance weighted	0.023477006	0.05	1
	IDP dMRI TBSS ICVF Sagittal stratum R	MR Egger	0.693225498	0.05	0.004238644
		Weighted median	0.103011416	0.05	1
		Inverse variance weighted	0.414383222	0.05	1
		Simple mode	0.250415975	0.05	1
		Weighted mode	0.219683797	0.05	1
	IDP dMRI TBSS ICVF Sagittal stratum L	Inverse variance weighted	0.015425095	0.05	1
	IDP dMRI TBSS ICVF External capsule R	MR Egger	0.437078733	0.05	0.999795422
		Weighted median	0.794964125	0.05	1
		Inverse variance weighted	0.98124266	0.05	1
		Simple mode	0.885545924	0.05	1
		Weighted mode	0.681157691	0.05	1
	IDP dMRI TBSS ICVF External capsule L	Inverse variance weighted	0.66411549	0.05	1

	IDP dMRI TBSS ICVF Cingulum cingulate gyrus R	Inverse variance weighted	0.968846667	0.05	1
	IDP dMRI TBSS ICVF Cingulum cingulate gyrus L	Wald ratio	0.262596629	0.05	1
	IDP dMRI TBSS ICVF Cingulum hippocampus R	MR Egger	0.548252196	0.05	1
		Weighted median	0.652362615	0.05	1
		Inverse variance weighted	0.995357435	0.05	1
		Simple mode	0.851943087	0.05	1
		Weighted mode	0.624875745	0.05	1
	IDP dMRI TBSS ICVF Cingulum hippocampus L	MR Egger	0.79190123	0.05	1
		Weighted median	0.651403175	0.05	1
		Inverse variance weighted	0.84694538	0.05	1
		Simple mode	0.470193972	0.05	1
		Weighted mode	0.527899893	0.05	1
	IDP dMRI TBSS ICVF Fornix cres+Stria terminalis R	Wald ratio	0.097633864	0.05	1
	IDP dMRI TBSS ICVF Superior longitudinal fasciculus R	Inverse variance weighted	0.64425654	0.05	1
	IDP dMRI TBSS ICVF Superior longitudinal fasciculus L	MR Egger	0.95410923	0.05	1
		Weighted median	0.807614698	0.05	1
		Inverse variance weighted	0.963674746	0.05	1
		Simple mode	0.513109985	0.05	1
		Weighted mode	0.550826507	0.05	1
	IDP dMRI TBSS ICVF Superior fronto-occipital fasciculus R	Inverse variance weighted	0.318641821	0.05	1
	IDP dMRI TBSS ICVF Uncinate fasciculus R	Wald ratio	0.528680752	0.05	1

	IDP dMRI TBSS ICVF Uncinate fasciculus L	Wald ratio	0.528680752	0.05	1
	IDP dMRI TBSS OD Pontine crossing tract	MR Egger	0.518122612	0.05	0.021514719
		Weighted median	0.967115011	0.05	1
		Inverse variance weighted	0.964400973	0.05	1
		Simple mode	0.637148903	0.05	1
		Weighted mode	0.847221045	0.05	1
	IDP dMRI TBSS OD Cerebral peduncle R	Wald ratio	0.776969403	0.05	1
	IDP dMRI TBSS OD Cerebral peduncle L	Wald ratio	0.776969403	0.05	1
	IDP dMRI TBSS OD Posterior limb of internal capsule L	Wald ratio	0.047855687	0.05	1
	IDP dMRI TBSS OD External capsule R	Inverse variance weighted	0.445853508	0.05	1
	IDP dMRI TBSS OD External capsule L	Wald ratio	0.585889106	0.05	1
	IDP dMRI TBSS OD Superior longitudinal fasciculus R	Wald ratio	0.463033859	0.05	1
	IDP dMRI TBSS ISOVF Fornix	Wald ratio	0.870577525	0.05	1
	IDP dMRI TBSS ISOVF External capsule R	Wald ratio	0.14507695	0.05	1
	IDP dMRI TBSS ISOVF Cingulum cingulate gyrus R	Wald ratio	0.46593013	0.05	1
	IDP dMRI ProbtrackX FA ar l	Wald ratio	0.528680752	0.05	1
	IDP dMRI ProbtrackX FA atr l	Wald ratio	0.528680752	0.05	1
	IDP dMRI ProbtrackX FA fmi	MR Egger	0.78429781	0.05	1
		Weighted median	0.356775571	0.05	1
		Inverse variance weighted	0.692452906	0.05	1
		Simple mode	0.44283762	0.05	1
		Weighted mode	0.480662405	0.05	1

	IDP dMRI ProbtrackX FA ifo l	Wald ratio	0.184961435	0.05	1
	IDP dMRI ProbtrackX FA ifo r	Wald ratio	0.172524636	0.05	1
	IDP dMRI ProbtrackX FA ml l	Wald ratio	0.734521804	0.05	1
	IDP dMRI ProbtrackX FA ptr r	Wald ratio	0.986094219	0.05	1
	IDP dMRI ProbtrackX FA slf l	Wald ratio	0.172178722	0.05	1
	IDP dMRI ProbtrackX FA str l	Wald ratio	0.060461644	0.05	0.92490001
	IDP dMRI ProbtrackX FA unc l	Wald ratio	0.528680752	0.05	1
	IDP dMRI ProbtrackX FA unc r	Wald ratio	0.528680752	0.05	1
	IDP dMRI ProbtrackX MD ar l	Wald ratio	0.528680752	0.05	1
	IDP dMRI ProbtrackX MD ar r	Wald ratio	0.528680752	0.05	1
	IDP dMRI ProbtrackX MD atr l	Wald ratio	0.528680752	0.05	1
	IDP dMRI ProbtrackX MD atr r	Wald ratio	0.528680752	0.05	1
	IDP dMRI ProbtrackX MD cgc l	MR Egger	0.401247729	0.05	1
		Weighted median	0.950800927	0.05	1
		Inverse variance weighted	0.801309276	0.05	1
		Simple mode	0.911970721	0.05	1
		Weighted mode	0.741522306	0.05	1
	IDP dMRI ProbtrackX MD cgc r	Inverse variance weighted	0.237831637	0.05	1
	IDP dMRI ProbtrackX MD fmi	Inverse variance weighted	0.868255666	0.05	1
	IDP dMRI ProbtrackX MD ifo l	Wald ratio	0.184961435	0.05	0.999998643
	IDP dMRI ProbtrackX MD ifo r	Wald ratio	0.184961435	0.05	0.999984279
	IDP dMRI ProbtrackX MD ilf l	Wald ratio	0.184961435	0.05	0.999980896
	IDP dMRI ProbtrackX MD ilf r	Wald ratio	0.184961435	0.05	0.999875513

	IDP dMRI ProbtrackX MD ptr l	Wald ratio	0.528680752	0.05	1
	IDP dMRI ProbtrackX MD ptr r	Wald ratio	0.528680752	0.05	1
	IDP dMRI ProbtrackX MD slf l	Inverse variance weighted	0.845693861	0.05	1
	IDP dMRI ProbtrackX MD slf r	Wald ratio	0.184961435	0.05	0.99943028
	IDP dMRI ProbtrackX MD str l	Wald ratio	0.479213069	0.05	1
	IDP dMRI ProbtrackX MD str r	Wald ratio	0.528680752	0.05	1
	IDP dMRI ProbtrackX MD unc l	Wald ratio	0.528680752	0.05	1
	IDP dMRI ProbtrackX MD unc r	Inverse variance weighted	0.680026507	0.05	1
	IDP dMRI ProbtrackX MO atr l	Wald ratio	0.288499993	0.05	1
	IDP dMRI ProbtrackX MO ml r	Wald ratio	0.926227229	0.05	1
	IDP T1 FAST ROIs L intracalc cortex	Wald ratio	0.998627886	0.05	1
	IDP dMRI ProbtrackX L1 ilf l	Wald ratio	0.528680752	0.05	1
	IDP T1 FAST ROIs R intracalc cortex	Wald ratio	0.998627886	0.05	1
	IDP dMRI ProbtrackX L1 slf l	Wald ratio	0.528680752	0.05	1
	IDP dMRI ProbtrackX L1 slf r	Wald ratio	0.176935604	0.05	0.412329532
	IDP dMRI ProbtrackX L1 str r	Wald ratio	0.362040805	0.05	1
	IDP dMRI ProbtrackX L1 unc l	Inverse variance weighted	0.731237707	0.05	1
	IDP dMRI ProbtrackX L1 unc r	Wald ratio	0.831450316	0.05	1
	IDP dMRI ProbtrackX L2 ar l	Wald ratio	0.528680752	0.05	1
	IDP dMRI ProbtrackX L2 ar r	Wald ratio	0.528680752	0.05	1
	IDP dMRI ProbtrackX L2 atr l	Wald ratio	0.528680752	0.05	1
	IDP dMRI ProbtrackX L2 atr r	Wald ratio	0.528680752	0.05	1
	IDP dMRI ProbtrackX L2 cgh l	Wald ratio	0.083262385	0.05	1

	IDP dMRI ProbtrackX L2 fma	Wald ratio	0.859131458	0.05	1
	IDP dMRI ProbtrackX L2 fmi	Inverse variance weighted	0.866252909	0.05	1
	IDP dMRI ProbtrackX L2 ifo l	Wald ratio	0.184961435	0.05	0.99999945
	IDP dMRI ProbtrackX L2 ifo r	Wald ratio	0.184961435	0.05	0.999996488
	IDP dMRI ProbtrackX L2 ilf l	Wald ratio	0.184961435	0.05	0.999982907
	IDP dMRI ProbtrackX L2 ilf r	Wald ratio	0.184961435	0.05	0.999988115
	IDP dMRI ProbtrackX L2 ptr l	Wald ratio	0.528680752	0.05	1
	IDP dMRI ProbtrackX L2 ptr r	Wald ratio	0.541903823	0.05	1
	IDP dMRI ProbtrackX L2 slf l	Inverse variance weighted	0.839895696	0.05	1
	IDP dMRI ProbtrackX L2 slf r	Wald ratio	0.184961435	0.05	0.99986163
	IDP dMRI ProbtrackX L2 str l	Wald ratio	0.479213069	0.05	1
	IDP dMRI ProbtrackX L2 str r	Wald ratio	0.479213069	0.05	1
	IDP dMRI ProbtrackX L2 unc l	Wald ratio	0.528680752	0.05	1
	IDP dMRI ProbtrackX L2 unc r	Inverse variance weighted	0.672227773	0.05	1
	IDP dMRI ProbtrackX L3 ar l	Wald ratio	0.528680752	0.05	1
	IDP dMRI ProbtrackX L3 ar r	Wald ratio	0.528680752	0.05	1
	IDP dMRI ProbtrackX L3 atr l	Wald ratio	0.528680752	0.05	1
	IDP dMRI ProbtrackX L3 atr r	Wald ratio	0.528680752	0.05	1
	IDP dMRI ProbtrackX L3 fmi	MR Egger	0.860920645	0.05	1
		Weighted median	0.644858006	0.05	1
		Inverse variance weighted	0.927911681	0.05	1
		Simple mode	0.501931176	0.05	1

		Weighted mode	0.544470315	0.05	1
	IDP dMRI ProbtrackX L3 ifo l	Inverse variance weighted	0.903317579	0.05	1
	IDP dMRI ProbtrackX L3 ifo r	Wald ratio	0.184961435	0.05	0.999990508
	IDP dMRI ProbtrackX L3 ilf l	Wald ratio	0.184961435	0.05	0.999987072
	IDP dMRI ProbtrackX L3 ilf r	Wald ratio	0.184961435	0.05	0.999930829
	IDP dMRI ProbtrackX L3 ptr l	Wald ratio	0.528680752	0.05	1
	IDP dMRI ProbtrackX L3 ptr r	Wald ratio	0.541903823	0.05	1
	IDP dMRI ProbtrackX L3 slf l	Inverse variance weighted	0.856647363	0.05	1
	IDP dMRI ProbtrackX L3 slf r	Wald ratio	0.184961435	0.05	0.999370431
	IDP dMRI ProbtrackX L3 str l	Wald ratio	0.541903823	0.05	1
	IDP dMRI ProbtrackX L3 unc l	Wald ratio	0.528680752	0.05	1
	IDP dMRI ProbtrackX L3 unc r	Inverse variance weighted	0.693536822	0.05	1
	IDP dMRI ProbtrackX ICVF ar l	MR Egger	0.69022378	0.05	1
		Weighted median	0.514341671	0.05	1
		Inverse variance weighted	0.705663883	0.05	1
		Simple mode	0.473047229	0.05	1
		Weighted mode	0.528544061	0.05	1
	IDP dMRI ProbtrackX ICVF ar r	MR Egger	0.718947176	0.05	1
		Weighted median	0.552470738	0.05	1
		Inverse variance weighted	0.731631368	0.05	1
		Simple mode	0.457638309	0.05	1
		Weighted mode	0.535445421	0.05	1

	IDP dMRI ProbtrackX ICVF atr l	MR Egger	0.73816184	0.05	1
		Weighted median	0.543834858	0.05	1
		Inverse variance weighted	0.819873646	0.05	1
		Simple mode	0.498775387	0.05	1
		Weighted mode	0.520557243	0.05	1
	IDP dMRI ProbtrackX ICVF atr r	MR Egger	0.727683527	0.05	1
		Weighted median	0.503720269	0.05	1
		Inverse variance weighted	0.82316465	0.05	1
		Simple mode	0.475401551	0.05	1
		Weighted mode	0.519374334	0.05	1
	IDP dMRI ProbtrackX ICVF cgc l	Wald ratio	0.262596629	0.05	1
	IDP dMRI ProbtrackX ICVF cgh l	MR Egger	0.725451281	0.05	1
		Weighted median	0.539836002	0.05	1
		Inverse variance weighted	0.811262158	0.05	1
		Simple mode	0.489576056	0.05	1
		Weighted mode	0.517847899	0.05	1
	IDP dMRI ProbtrackX ICVF cgh r	Wald ratio	0.528680752	0.05	1
	IDP dMRI ProbtrackX ICVF cst l	MR Egger	0.713873147	0.05	1
		Weighted median	0.538925076	0.05	1
		Inverse variance weighted	0.762695872	0.05	1
		Simple mode	0.486789544	0.05	1
		Weighted mode	0.54293577	0.05	1

	IDP dMRI ProbtrackX ICVF cst r	MR Egger	0.45174496	0.05	0.821859803
		Weighted median	0.966422875	0.05	1
		Inverse variance weighted	0.638457085	0.05	1
		Simple mode	0.797854989	0.05	1
		Weighted mode	0.621027228	0.05	1
	IDP dMRI ProbtrackX ICVF fma	Inverse variance weighted	0.717109489	0.05	1
	IDP dMRI ProbtrackX ICVF fmi	MR Egger	0.790209772	0.05	1
		Weighted median	0.424347059	0.05	1
		Inverse variance weighted	0.69204317	0.05	1
		Simple mode	0.489997934	0.05	1
		Weighted mode	0.518728231	0.05	1
	IDP dMRI ProbtrackX ICVF ifo l	Inverse variance weighted	0.949285044	0.05	1
	IDP dMRI ProbtrackX ICVF ifo r	Inverse variance weighted	0.94508641	0.05	1
	IDP dMRI ProbtrackX ICVF ilf l	Inverse variance weighted	0.932052498	0.05	1
	IDP dMRI ProbtrackX ICVF ilf r	Wald ratio	0.184961435	0.05	1
	IDP dMRI ProbtrackX ICVF mcp	Wald ratio	0.427812095	0.05	1
	IDP dMRI ProbtrackX ICVF ml l	Wald ratio	0.985209094	0.05	1
	IDP dMRI ProbtrackX ICVF ml r	Wald ratio	0.528680752	0.05	1
	IDP dMRI ProbtrackX ICVF ptr l	Wald ratio	0.184961435	0.05	1
	IDP dMRI ProbtrackX ICVF ptr r	Inverse variance weighted	0.687926855	0.05	1
	IDP dMRI ProbtrackX ICVF slf l	MR Egger	0.862939508	0.05	1
		Weighted median	0.718610965	0.05	1

		Inverse variance weighted	0.91197722	0.05	1
		Simple mode	0.465597342	0.05	1
		Weighted mode	0.53714599	0.05	1
	IDP dMRI ProbtrackX ICVF slf r	Inverse variance weighted	0.656911765	0.05	1
	IDP dMRI ProbtrackX ICVF str l	MR Egger	0.766200045	0.05	1
		Weighted median	0.575848155	0.05	1
		Inverse variance weighted	0.868792878	0.05	1
		Simple mode	0.477796813	0.05	1
		Weighted mode	0.519936039	0.05	1
	IDP dMRI ProbtrackX ICVF str r	MR Egger	0.826432274	0.05	1
		Weighted median	0.652896328	0.05	1
		Inverse variance weighted	0.897473767	0.05	1
		Simple mode	0.501900598	0.05	1
		Weighted mode	0.51841838	0.05	1
	IDP dMRI ProbtrackX ICVF unc l	Inverse variance weighted	0.378174672	0.05	1
	IDP dMRI ProbtrackX ICVF unc r	Inverse variance weighted	0.983895242	0.05	1
	IDP dMRI ProbtrackX OD atr r	Wald ratio	0.399782532	0.05	0.999979406
	IDP dMRI ProbtrackX OD ilf l	Wald ratio	0.961716243	0.05	1
	IDP dMRI ProbtrackX OD slf l	Wald ratio	0.372184759	0.05	1
	IDP dMRI ProbtrackX OD str l	Inverse variance weighted	0.01343919	0.05	0.642598984
	IDP dMRI ProbtrackX ISOVF atr r	Wald ratio	0.528680752	0.05	1
	IDP dMRI ProbtrackX ISOVF cgc l	Wald ratio	0.133757892	0.05	0.986669647

	IDP dMRI ProbtrackX ISOVF ifo r	Wald ratio	0.556115245	0.05	1
	IDP dMRI ProbtrackX ISOVF ilf l	Wald ratio	0.82202259	0.05	1
	IDP dMRI ProbtrackX ISOVF ilf r	MR Egger	0.739323143	0.05	0.002747051
		Weighted median	0.211876477	0.05	0.999999995
		Inverse variance weighted	0.0958284	0.05	0.999999898
		Simple mode	0.409418708	0.05	1
		Weighted mode	0.427852281	0.05	1
	IDP dMRI ProbtrackX ISOVF slf l	Inverse variance weighted	0.449475356	0.05	1
	IDP dMRI ProbtrackX ISOVF slf r	MR Egger	0.536360543	0.05	1
		Weighted median	0.907407478	0.05	1
		Inverse variance weighted	0.753725364	0.05	1
		Simple mode	0.97600152	0.05	1
		Weighted mode	0.905560062	0.05	1
	IDP dMRI ProbtrackX ISOVF unc l	Wald ratio	0.889093198	0.05	1
	IDP dMRI ProbtrackX ISOVF unc r	Wald ratio	0.834233822	0.05	1
	IDP T1 SIENAX brain-normalised volume	Wald ratio	0.803408758	0.05	1

Table S9. Statistical power calculation of reverse MR

Supplementary Table 9: Statistical power calculation of reverse MR					
Outcome	Exposure	Method	Pval	Variance_explained	Power_80_5
IDP dMRI ProbtrackX L1 slf r	Behcet's disease	MR Egger	0.580966152	0.05	1
		Weighted median	0.458005573	0.05	1
		Inverse variance weighted	0.419253197	0.05	1
		Simple mode	0.807960287	0.05	1
		Weighted mode	0.648734359	0.05	1
IDP SWI T2star left thalamus plus IDP SWI T2star right thalamus	Connective tissue disorder	Inverse variance weighted	0.109033762	0.05	0.002542628
IDP dMRI TBSS ICVF Anterior limb of internal capsule L		Inverse variance weighted	0.940103636	0.05	1
IDP dMRI TBSS ICVF Cingulum hippocampus R		Inverse variance weighted	0.251997257	0.05	1
IDP dMRI TBSS ISOVF Cingulum cingulate gyrus R		Inverse variance weighted	0.20219476	0.05	1
IDP dMRI ProbtrackX ISOVF ifo r		Inverse variance weighted	0.291614549	0.05	1
IDP T1 FAST ROIs L hippocampus	Dermatopolymyositis	Wald ratio	0.435938618	0.05	1
IDP dMRI TBSS FA Splenium of corpus callosum		Wald ratio	0.013626544	0.05	1
IDP dMRI TBSS L2 Splenium of corpus callosum		Wald ratio	0.009394747	0.05	1
IDP dMRI TBSS ICVF Body of corpus callosum		Wald ratio	0.075843349	0.05	1
IDP dMRI TBSS ICVF Splenium of corpus callosum		Wald ratio	0.037016178	0.05	1

IDP dMRI TBSS ICVF Posterior limb of internal capsule R		Wald ratio	0.044041184	0.05	1
IDP SWI T2star left pallidum	Diseases of the musculoskeletal system and connective tissue	Inverse variance weighted	0.316796725	0.05	1
volume Left-Lateral-Ventricle		Inverse variance weighted	0.642132782	0.05	1
volume Right-Lateral-Ventricle		Inverse variance weighted	0.972842578	0.05	1
DKTatlas lh pericalcarine area		Inverse variance weighted	0.941015863	0.05	1
IDP T1 SIENAX CSF normalised volume		Inverse variance weighted	0.706643504	0.05	1
IDP dMRI TBSS MO Cingulum cingulate gyrus L		Inverse variance weighted	0.683818303	0.05	1
IDP T1 SIENAX CSF unnormalised volume		Inverse variance weighted	0.702659488	0.05	1
IDP dMRI TBSS ICVF Cerebral peduncle R		Inverse variance weighted	0.163471603	0.05	1
IDP dMRI TBSS FA Superior longitudinal fasciculus L	Drug-induced systemic lupus erythematosus	MR Egger	0.801814291	0.05	1
		Weighted median	0.596163528	0.05	1
		Inverse variance weighted	0.341802996	0.05	1
		Simple mode	0.788604376	0.05	1
		Weighted mode	0.603757375	0.05	1
DKTatlas lh pericalcarine area		MR Egger	0.371734635	0.05	1
		Weighted median	0.508678863	0.05	1
		Inverse variance weighted	0.365446343	0.05	1
		Simple mode	0.134487076	0.05	1
		Weighted mode	0.426523801	0.05	1
		MR Egger	0.877202151	0.05	1

DKTatlas rh posteriorcingulate thickness		Weighted median	0.898131913	0.05	1
		Inverse variance weighted	0.695977534	0.05	1
		Simple mode	0.462736062	0.05	1
		Weighted mode	0.414715764	0.05	1
IDP dMRI TBSS L3 Posterior corona radiata R		MR Egger	0.744781221	0.05	1
		Weighted median	0.66334372	0.05	1
		Inverse variance weighted	0.9896649	0.05	1
		Simple mode	0.337950046	0.05	1
		Weighted mode	0.565017945	0.05	1
IDP dMRI TBSS L3 Uncinate fasciculus L		MR Egger	0.601028159	0.05	1
		Weighted median	0.804827792	0.05	1
		Inverse variance weighted	0.660833161	0.05	1
		Simple mode	0.924197108	0.05	1
		Weighted mode	0.910611463	0.05	1
IDP dMRI TBSS ICVF Retrolenticular part of internal capsule R		MR Egger	0.689482978	0.05	1
		Weighted median	0.744677593	0.05	1
		Inverse variance weighted	0.652935751	0.05	1
		Simple mode	0.874231749	0.05	1
		Weighted mode	0.718377491	0.05	1
IDP dMRI ProbtrackX ICVF ar l		MR Egger	0.913150041	0.05	1
		Weighted median	0.360121611	0.05	1
		Inverse variance weighted	0.941487375	0.05	1

		Simple mode	0.609489951	0.05	1
		Weighted mode	0.361402557	0.05	1
IDP dMRI ProbtrackX ICVF ar r		MR Egger	0.948214264	0.05	1
		Weighted median	0.430280358	0.05	1
		Inverse variance weighted	0.762362404	0.05	1
		Simple mode	0.430464604	0.05	1
		Weighted mode	0.46899947	0.05	1
IDP dMRI ProbtrackX ICVF cst l		MR Egger	0.897631965	0.05	1
		Weighted median	0.818222053	0.05	1
		Inverse variance weighted	0.698147556	0.05	1
		Simple mode	0.513368745	0.05	1
		Weighted mode	0.898925918	0.05	1
IDP dMRI ProbtrackX ICVF unc l		MR Egger	0.705030401	0.05	1
		Weighted median	0.643943396	0.05	1
		Inverse variance weighted	0.771023008	0.05	1
		Simple mode	0.983623804	0.05	1
		Weighted mode	0.752161174	0.05	1
IDP T1 FAST ROIs V cerebellum VIIIa	Fibromyalgia	MR Egger	0.108086484	0.05	1
		Weighted median	0.201695038	0.05	1
		Inverse variance weighted	0.013340416	0.05	1
		Simple mode	0.195575792	0.05	1
		Weighted mode	0.140881839	0.05	1

IDP SWI T2star left pallidum		MR Egger	0.729808389	0.05	1
		Weighted median	0.617628416	0.05	1
		Inverse variance weighted	0.804712156	0.05	1
		Simple mode	0.638686378	0.05	1
		Weighted mode	0.701773723	0.05	1
IDP SWI T2star left pallidum plus IDP SWI T2star right pallidum		MR Egger	0.772350616	0.05	1
		Weighted median	0.414900809	0.05	1
		Inverse variance weighted	0.740996267	0.05	1
		Simple mode	0.394553797	0.05	1
		Weighted mode	0.311952888	0.05	1
IDP T1 FAST ROIs L cerebellum crus I	Fibromyalgia related co-morbidities	MR Egger	0.620346673	0.05	1
		Weighted median	0.161112206	0.05	0.006813343
		Inverse variance weighted	0.064905568	0.05	0.006965452
		Simple mode	0.290981832	0.05	0.002768258
		Weighted mode	0.274672244	0.05	0.002722918
IDP dMRI TBSS FA Superior cerebellar peduncle R		MR Egger	0.04625737	0.05	1
		Weighted median	0.483171897	0.05	1
		Inverse variance weighted	0.369432278	0.05	1
		Simple mode	0.574136185	0.05	1
		Weighted mode	0.533378592	0.05	1
IDP dMRI TBSS FA Superior cerebellar peduncle L		MR Egger	0.25218142	0.05	1
		Weighted median	0.960855008	0.05	1

		Inverse variance weighted	0.870040844	0.05	1
		Simple mode	0.696448166	0.05	0.77055365
		Weighted mode	0.788598548	0.05	1
IDP dMRI TBSS MD Splenium of corpus callosum		MR Egger	0.0910662	0.05	0.002548727
		Weighted median	0.651032969	0.05	1
		Inverse variance weighted	0.631807952	0.05	1
		Simple mode	0.617685128	0.05	0.18370717
		Weighted mode	0.644809675	0.05	0.770810331
volume Left-Cerebellum-Cortex		MR Egger	0.648723657	0.05	1
		Weighted median	0.062778814	0.05	0.004043085
		Inverse variance weighted	0.014076164	0.05	0.004678893
		Simple mode	0.191875445	0.05	0.002605698
		Weighted mode	0.197507236	0.05	0.002628342
DKTatlas rh posteriorcingulate thickness	Giant cell arteritis	Wald ratio	0.43559043	0.05	1
volume 4th-Ventricle	Giant cell arteritis with polymyalgia rheumatica	Wald ratio	0.365293001	0.05	1
DKTatlas rh posteriorcingulate thickness		Wald ratio	0.43559043	0.05	1
IDP dMRI TBSS OD Pontine crossing tract		Wald ratio	0.727411081	0.05	1
IDP T1 FAST ROIs R ventral striatum	Multiple Sclerosis	MR Egger	0.198390762	0.05	1
		Weighted median	0.129591916	0.05	1
		Inverse variance weighted	0.678551558	0.05	1
		Simple mode	0.748415927	0.05	1
		Weighted mode	0.140335579	0.05	1

IDP SWI T2star left caudate		MR Egger	0.244295867	0.05	1
		Weighted median	0.112659029	0.05	1
		Inverse variance weighted	0.185290249	0.05	1
		Simple mode	0.074663139	0.05	1
		Weighted mode	0.162740665	0.05	1
IDP SWI T2star right caudate		MR Egger	0.202244322	0.05	1
		Weighted median	0.099528711	0.05	1
		Inverse variance weighted	0.067972768	0.05	1
		Simple mode	0.275666178	0.05	1
		Weighted mode	0.037679275	0.05	1
IDP SWI T2star left caudate plus IDP SWI T2star right caudate		MR Egger	0.178592247	0.05	1
		Weighted median	0.06723551	0.05	1
		Inverse variance weighted	0.081842037	0.05	1
		Simple mode	0.385548257	0.05	1
		Weighted mode	0.056584079	0.05	1
volume Left-Lateral-Ventricle		MR Egger	0.616716191	0.05	1
		Weighted median	0.159517065	0.05	1
		Inverse variance weighted	0.320442415	0.05	1
		Simple mode	0.848056816	0.05	1
		Weighted mode	0.488289933	0.05	1
IDP T1 SIENAX CSF normalised volume		MR Egger	0.476451538	0.05	1
		Weighted median	0.25958759	0.05	1

		Inverse variance weighted	0.226175541	0.05	1
		Simple mode	0.152257953	0.05	1
		Weighted mode	0.378003432	0.05	1
IDP T1 SIENAX CSF unnormalised volume		MR Egger	0.481411798	0.05	1
		Weighted median	0.224173371	0.05	1
		Inverse variance weighted	0.226995749	0.05	1
		Simple mode	0.1737805	0.05	1
		Weighted mode	0.344789913	0.05	1
IDP dMRI ProbtrackX OD str l		MR Egger	0.528478816	0.05	1
		Weighted median	0.87482674	0.05	1
		Inverse variance weighted	0.10577965	0.05	1
		Simple mode	0.5179394	0.05	1
		Weighted mode	0.858172358	0.05	1
IDP T1 FAST ROIs L thalamus	Polyarteritis nodosa	MR Egger	0.443387413	0.05	1
		Weighted median	0.612482353	0.05	1
		Inverse variance weighted	0.8866667	0.05	1
		Simple mode	0.481016249	0.05	1
		Weighted mode	0.58906694	0.05	1
volume Right-Cerebellum-Cortex		MR Egger	0.551541691	0.05	1
		Weighted median	0.710540805	0.05	1
		Inverse variance weighted	0.930772282	0.05	1
		Simple mode	0.635579183	0.05	1

		Weighted mode	0.449731944	0.05	1
DKTatlas lh pericalcarine area		MR Egger	0.371161994	0.05	1
		Weighted median	0.287525344	0.05	1
		Inverse variance weighted	0.270653286	0.05	1
		Simple mode	0.20735498	0.05	1
		Weighted mode	0.413988192	0.05	1
IDP T1 FAST ROIs R occ pole	Polymyositis	MR Egger	0.456609422	0.05	1
		Weighted median	0.616973065	0.05	1
		Inverse variance weighted	0.201505263	0.05	1
		Simple mode	0.938971206	0.05	1
		Weighted mode	0.907075867	0.05	1
IDP dMRI TBSS ICVF Medial lemniscus R		MR Egger	0.38372487	0.05	1
		Weighted median	0.640676515	0.05	1
		Inverse variance weighted	0.564853015	0.05	1
		Simple mode	0.965207943	0.05	1
		Weighted mode	0.58518953	0.05	1
IDP dMRI TBSS ICVF Cingulum cingulate gyrus L		MR Egger	0.656322221	0.05	1
		Weighted median	0.841035952	0.05	1
		Inverse variance weighted	0.555273689	0.05	1
		Simple mode	0.527228555	0.05	1
		Weighted mode	0.509652862	0.05	1
		MR Egger	0.729202111	0.05	1

IDP dMRI ProbtrackX ICVF cgc l		Weighted median	0.938820894	0.05	1
		Inverse variance weighted	0.460344891	0.05	1
		Simple mode	0.909643892	0.05	1
		Weighted mode	0.887153302	0.05	1
DKTatlas rh lateraloccipital area	Sjogren's syndrome (Firth correction)	Wald ratio	0.001700133	0.05	1
IDP dMRI TBSS L1 Superior longitudinal fasciculus R		Wald ratio	0.371718796	0.05	1
IDP SWI T2star left putamen	Systemic connective tissue disorders	Inverse variance weighted	0.846096218	0.05	1
IDP SWI T2star left thalamus plus IDP SWI T2star right thalamus		Inverse variance weighted	0.878823852	0.05	1
IDP SWI T2star left caudate plus IDP SWI T2star right caudate		Inverse variance weighted	0.932252789	0.05	1
IDP SWI T2star left putamen plus IDP SWI T2star right putamen		Inverse variance weighted	0.928007834	0.05	1
IDP T1 FAST ROIs L cerebellum crus I	Systemic lupus erythematosus	MR Egger	0.589928494	0.05	1
		Weighted median	0.632794079	0.05	1
		Inverse variance weighted	0.746437997	0.05	1
		Simple mode	0.519228155	0.05	1
		Weighted mode	0.680176648	0.05	1
IDP SWI T2star left putamen		MR Egger	0.633140531	0.05	1
		Weighted median	0.291242131	0.05	1
		Inverse variance weighted	0.536982647	0.05	1
		Simple mode	0.412360311	0.05	1

		Weighted mode	0.416123232	0.05	1
IDP dMRI TBSS FA Cingulum cingulate gyrus R		MR Egger	0.74402079	0.05	1
		Weighted median	0.244024787	0.05	1
		Inverse variance weighted	0.088306138	0.05	1
		Simple mode	0.494669668	0.05	1
		Weighted mode	0.469949094	0.05	1
IDP dMRI TBSS MD External capsule L		MR Egger	0.83477841	0.05	1
		Weighted median	0.796132398	0.05	1
		Inverse variance weighted	0.899168426	0.05	1
		Simple mode	0.776166729	0.05	1
		Weighted mode	0.806966745	0.05	1
IDP dMRI TBSS MD Cingulum cingulate gyrus R		MR Egger	0.556493762	0.05	1
		Weighted median	0.282633701	0.05	1
		Inverse variance weighted	0.494421518	0.05	1
		Simple mode	0.46563266	0.05	1
		Weighted mode	0.360308919	0.05	1
IDP dMRI TBSS MD Cingulum cingulate gyrus L		MR Egger	0.829041799	0.05	1
		Weighted median	0.138987501	0.05	1
		Inverse variance weighted	0.104069349	0.05	1
		Simple mode	0.353584676	0.05	1
		Weighted mode	0.368604715	0.05	1
		MR Egger	0.555237808	0.05	1

IDP dMRI TBSS MD Uncinate fasciculus L		Weighted median	0.022419757	0.05	1
		Inverse variance weighted	0.067965004	0.05	1
		Simple mode	0.244351422	0.05	1
		Weighted mode	0.217225501	0.05	1
IDP dMRI TBSS L3 Uncinate fasciculus L		MR Egger	0.671955998	0.05	1
		Weighted median	0.107440995	0.05	1
		Inverse variance weighted	0.090986077	0.05	1
		Simple mode	0.231066146	0.05	1
		Weighted mode	0.249896818	0.05	1
IDP dMRI TBSS ICVF Anterior limb of internal capsule L		MR Egger	0.968511762	0.05	1
		Weighted median	0.516477025	0.05	1
		Inverse variance weighted	0.387541392	0.05	1
		Simple mode	0.995863788	0.05	1
		Weighted mode	0.790230608	0.05	1
IDP dMRI TBSS ICVF Posterior limb of internal capsule L		MR Egger	0.549667046	0.05	1
		Weighted median	0.087527439	0.05	1
		Inverse variance weighted	0.438375389	0.05	1
		Simple mode	0.355466956	0.05	1
		Weighted mode	0.221809879	0.05	1
IDP dMRI TBSS ICVF Retrolenticular part of internal capsule L		MR Egger	0.352047689	0.05	1
		Weighted median	0.26637524	0.05	1
		Inverse variance weighted	0.09660388	0.05	1

		Simple mode	0.844814213	0.05	1
		Weighted mode	0.835760953	0.05	1
IDP dMRI TBSS ICVF Anterior corona radiata R		MR Egger	0.509916755	0.05	1
		Weighted median	0.737813111	0.05	1
		Inverse variance weighted	0.972426269	0.05	1
		Simple mode	0.815773251	0.05	1
		Weighted mode	0.605152532	0.05	1
IDP dMRI TBSS ICVF Anterior corona radiata L		MR Egger	0.521961008	0.05	1
		Weighted median	0.347039575	0.05	1
		Inverse variance weighted	0.612177055	0.05	1
		Simple mode	0.512397139	0.05	1
		Weighted mode	0.374713653	0.05	1
IDP dMRI TBSS ICVF Superior corona radiata R		MR Egger	0.269287462	0.05	1
		Weighted median	0.477690612	0.05	1
		Inverse variance weighted	0.414527738	0.05	1
		Simple mode	0.735814579	0.05	1
		Weighted mode	0.808522671	0.05	1
IDP dMRI TBSS ICVF Superior corona radiata L		MR Egger	0.263213941	0.05	1
		Weighted median	0.640166067	0.05	1
		Inverse variance weighted	0.591755809	0.05	1
		Simple mode	0.627397834	0.05	1
		Weighted mode	0.305639467	0.05	1

IDP dMRI TBSS ICVF Posterior corona radiata L		MR Egger	0.3779419	0.05	1
		Weighted median	0.047179295	0.05	1
		Inverse variance weighted	0.018617376	0.05	1
		Simple mode	0.400745728	0.05	1
		Weighted mode	0.206088917	0.05	1
IDP dMRI TBSS ICVF External capsule R		MR Egger	0.680707941	0.05	1
		Weighted median	0.56783907	0.05	1
		Inverse variance weighted	0.761184553	0.05	1
		Simple mode	0.638371747	0.05	1
		Weighted mode	0.611204049	0.05	1
IDP dMRI TBSS ICVF External capsule L		MR Egger	0.875058862	0.05	1
		Weighted median	0.659617259	0.05	1
		Inverse variance weighted	0.645320319	0.05	1
		Simple mode	0.754420466	0.05	1
		Weighted mode	0.748075535	0.05	1
IDP dMRI TBSS ICVF Cingulum hippocampus R		MR Egger	0.681177634	0.05	1
		Weighted median	0.606522818	0.05	1
		Inverse variance weighted	0.251685255	0.05	1
		Simple mode	0.83226238	0.05	1
		Weighted mode	0.880956249	0.05	1
IDP dMRI TBSS ICVF Cingulum hippocampus L		MR Egger	0.602662412	0.05	1
		Weighted median	0.41222854	0.05	1

		Inverse variance weighted	0.319757245	0.05	1
		Simple mode	0.417105236	0.05	1
		Weighted mode	0.878409219	0.05	1
IDP dMRI TBSS ICVF Superior longitudinal fasciculus L		MR Egger	0.403721632	0.05	1
		Weighted median	0.688345104	0.05	1
		Inverse variance weighted	0.310942449	0.05	1
		Simple mode	0.936323779	0.05	1
		Weighted mode	0.929239118	0.05	1
IDP dMRI TBSS ICVF Superior fronto-occipital fasciculus R		MR Egger	0.576327277	0.05	1
		Weighted median	0.998996278	0.05	1
		Inverse variance weighted	0.734786008	0.05	1
		Simple mode	0.982997272	0.05	1
		Weighted mode	0.79108713	0.05	1
IDP dMRI ProbtrackX MD cgc l		MR Egger	0.795885112	0.05	1
		Weighted median	0.140751381	0.05	1
		Inverse variance weighted	0.093110954	0.05	1
		Simple mode	0.346670308	0.05	1
		Weighted mode	0.337713284	0.05	1
IDP dMRI ProbtrackX L1 unc l		MR Egger	0.63498642	0.05	1
		Weighted median	0.061895213	0.05	1
		Inverse variance weighted	0.039808189	0.05	1
		Simple mode	0.245189503	0.05	1

		Weighted mode	0.23615655	0.05	1
IDP dMRI ProbtrackX L3 fmi		MR Egger	0.992630106	0.05	1
		Weighted median	0.795912666	0.05	1
		Inverse variance weighted	0.782707807	0.05	1
		Simple mode	0.641081213	0.05	1
		Weighted mode	0.801502046	0.05	1
IDP dMRI ProbtrackX ICVF ar l		MR Egger	0.360356133	0.05	1
		Weighted median	0.161517496	0.05	1
		Inverse variance weighted	0.120286921	0.05	1
		Simple mode	0.586464789	0.05	1
		Weighted mode	0.292782485	0.05	1
IDP dMRI ProbtrackX ICVF ar r		MR Egger	0.352266345	0.05	1
		Weighted median	0.390620908	0.05	1
		Inverse variance weighted	0.119943932	0.05	1
		Simple mode	0.897407587	0.05	1
		Weighted mode	0.90764873	0.05	1
IDP dMRI ProbtrackX ICVF atr l		MR Egger	0.788963929	0.05	1
		Weighted median	0.727782569	0.05	1
		Inverse variance weighted	0.80279737	0.05	1
		Simple mode	0.648679099	0.05	1
		Weighted mode	0.631317313	0.05	1
		MR Egger	0.756310941	0.05	1

IDP dMRI ProbtrackX ICVF atr r		Weighted median	0.856449313	0.05	1
		Inverse variance weighted	0.490623034	0.05	1
		Simple mode	0.898170341	0.05	1
		Weighted mode	0.852767653	0.05	1
IDP dMRI ProbtrackX ICVF cgh l		MR Egger	0.412161977	0.05	1
		Weighted median	0.073131346	0.05	1
		Inverse variance weighted	0.125175952	0.05	1
		Simple mode	0.195013918	0.05	1
		Weighted mode	0.929761167	0.05	1
IDP dMRI ProbtrackX ICVF cst l		MR Egger	0.328118377	0.05	1
		Weighted median	0.42944965	0.05	1
		Inverse variance weighted	0.298046362	0.05	1
		Simple mode	0.948992535	0.05	1
		Weighted mode	0.258100431	0.05	1
IDP dMRI ProbtrackX ICVF cst r		MR Egger	0.331628219	0.05	1
		Weighted median	0.44340918	0.05	1
		Inverse variance weighted	0.389686882	0.05	1
		Simple mode	0.95838345	0.05	1
		Weighted mode	0.34851972	0.05	1
IDP dMRI ProbtrackX ICVF slf l		MR Egger	0.362634363	0.05	1
		Weighted median	0.658555014	0.05	1
		Inverse variance weighted	0.467236877	0.05	1

		Simple mode	0.791114095	0.05	1
		Weighted mode	0.283657061	0.05	1
IDP dMRI ProbtrackX ICVF str l		MR Egger	0.250382606	0.05	1
		Weighted median	0.635329962	0.05	1
		Inverse variance weighted	0.781378718	0.05	1
		Simple mode	0.476763959	0.05	1
		Weighted mode	0.171662418	0.05	1
IDP dMRI ProbtrackX ICVF str r		MR Egger	0.27441278	0.05	1
		Weighted median	0.972366253	0.05	1
		Inverse variance weighted	0.594979401	0.05	1
		Simple mode	0.618970469	0.05	1
		Weighted mode	0.655474295	0.05	1
IDP dMRI ProbtrackX ICVF unc l		MR Egger	0.367100207	0.05	1
		Weighted median	0.457679322	0.05	1
		Inverse variance weighted	0.220609042	0.05	1
		Simple mode	0.973376538	0.05	1
		Weighted mode	0.982266341	0.05	1
IDP SWI T2star left caudate	Systemic sclerosis	Wald ratio	0.950626935	0.05	1
IDP SWI T2star right caudate		Wald ratio	0.225951858	0.05	1
IDP SWI T2star left putamen		Wald ratio	0.439349633	0.05	1
IDP SWI T2star right putamen		Wald ratio	0.499060332	0.05	1

IDP SWI T2star left caudate plus IDP SWI T2star right caudate		Wald ratio	0.489516876	0.05	1
IDP SWI T2star left putamen plus IDP SWI T2star right putamen		Wald ratio	0.95899912	0.05	1
IDP dMRI TBSS L2 Splenium of corpus callosum	TMD muscular pain lnked with fibromyalgia	MR Egger	0.389780845	0.05	1
		Weighted median	0.91455952	0.05	1
		Inverse variance weighted	0.925828143	0.05	1
		Simple mode	0.646600366	0.05	1
		Weighted mode	0.617609163	0.05	1
IDP dMRI TBSS ICVF Body of corpus callosum		MR Egger	0.325564247	0.05	1
		Weighted median	0.965574239	0.05	1
		Inverse variance weighted	0.696304315	0.05	1
		Simple mode	0.999948176	0.05	1
		Weighted mode	0.918442862	0.05	1
IDP dMRI TBSS ICVF Splenium of corpus callosum		MR Egger	0.735864427	0.05	1
		Weighted median	0.043113526	0.05	1
		Inverse variance weighted	0.471904834	0.05	1
		Simple mode	0.169901931	0.05	1
		Weighted mode	0.149062944	0.05	1
IDP dMRI TBSS OD Pontine crossing tract		MR Egger	0.207603747	0.05	1
		Weighted median	0.382960541	0.05	1
		Inverse variance weighted	0.310811171	0.05	1
		Simple mode	0.844581283	0.05	1

		Weighted mode	0.509099432	0.05	1
IDP dMRI ProbtrackX FA fmi		MR Egger	0.637373188	0.05	1
		Weighted median	0.979437719	0.05	1
		Inverse variance weighted	0.845013523	0.05	1
		Simple mode	0.511684257	0.05	1
		Weighted mode	0.869974367	0.05	1
IDP dMRI TBSS FA Superior cerebellar peduncle R	Vasculitis limited to skin, not elsewhere classified	MR Egger	0.415200896	0.05	1
		Weighted median	0.740141165	0.05	1
		Inverse variance weighted	0.759690793	0.05	1
		Simple mode	0.753179545	0.05	1
		Weighted mode	0.705091035	0.05	1
IDP dMRI TBSS FA Superior cerebellar peduncle L		MR Egger	0.285975091	0.05	1
		Weighted median	0.871366076	0.05	1
		Inverse variance weighted	0.70845378	0.05	1
		Simple mode	0.772859891	0.05	1
		Weighted mode	0.867314037	0.05	1
volume Left-Lateral-Ventricle		MR Egger	0.267074897	0.05	1
		Weighted median	0.915436498	0.05	1
		Inverse variance weighted	0.592434104	0.05	1
		Simple mode	0.876121341	0.05	1
		Weighted mode	0.8896188	0.05	1
volume CSF		MR Egger	0.079094191	0.05	1

		Weighted median	0.404528682	0.05	1
		Inverse variance weighted	0.17794615	0.05	1
		Simple mode	0.296043593	0.05	1
		Weighted mode	0.264933645	0.05	1
IDP T1 SIENAX CSF normalised volume		MR Egger	0.076563864	0.05	1
		Weighted median	0.946501731	0.05	1
		Inverse variance weighted	0.584768254	0.05	1
		Simple mode	0.982781103	0.05	1
		Weighted mode	0.952962788	0.05	1
IDP T1 SIENAX CSF unnormalised volume		MR Egger	0.074209632	0.05	1
		Weighted median	0.912643388	0.05	1
		Inverse variance weighted	0.558844695	0.05	1
		Simple mode	0.999489695	0.05	1
		Weighted mode	0.924331521	0.05	1
IDP dMRI TBSS L2 Superior cerebellar peduncle R		MR Egger	0.625982349	0.05	1
		Weighted median	0.531205789	0.05	1
		Inverse variance weighted	0.950023782	0.05	1
		Simple mode	0.334158053	0.05	1
		Weighted mode	0.700282386	0.05	1
IDP dMRI TBSS L3 Superior cerebellar peduncle R		MR Egger	0.595054394	0.05	1
		Weighted median	0.935642316	0.05	1
		Inverse variance weighted	0.985247153	0.05	1

		Simple mode	0.634876122	0.05	1
		Weighted mode	0.739685947	0.05	1
IDP dMRI TBSS ICVF Posterior thalamic radiation L		MR Egger	0.076745969	0.05	1
		Weighted median	0.314192594	0.05	1
		Inverse variance weighted	0.459759995	0.05	1
		Simple mode	0.317170117	0.05	1
		Weighted mode	0.189150542	0.05	1
IDP dMRI TBSS ICVF Sagittal stratum L		MR Egger	0.017976257	0.05	1
		Weighted median	0.04319976	0.05	1
		Inverse variance weighted	0.197000986	0.05	1
		Simple mode	0.204048454	0.05	1
		Weighted mode	0.066941481	0.05	1
IDP dMRI ProbtrackX OD str l		MR Egger	0.519207665	0.05	1
		Weighted median	0.590421195	0.05	1
		Inverse variance weighted	0.150512468	0.05	1
		Simple mode	0.85049661	0.05	1
		Weighted mode	0.623573211	0.05	1

Table S10: Summary of Statistical Power and Outcome Classification Across All 18 AIDs

Supplementary Table 10: Summary of Statistical Power and Outcome Classification Across All 18 AIDs				
AID	Cases (n)	Bonferroni-Significant?	Power (%)	Classification
Multiple sclerosis	47,429	Yes	>99%	Hypothesis-confirming
Systemic lupus erythematosus	647	Yes	>99%	Hypothesis-confirming
Fibromyalgia	737	Yes (reverse MR)	>99%	Hypothesis-confirming
Vasculitis limited to skin	288	Yes	24.8-100%	Exploratory only
Behçet's disease	27	No	0.25-100%	True negative
Polymyositis	44	No	0.25-100%	True negative
Dermatopolymyositis	208	No	0.25-100%	True negative
Drug-induced Systemic lupus erythematosus	101	No	0.25-100%	True negative
Polyarteritis nodosa	82	No	0.25-100%	True negative
Systemic sclerosis	302	No	0.25-100%	True negative
Giant cell arteritis	459	No	0.25-100%	True negative
Giant cell arteritis with polymyalgia rheumatica	421	No	0.27-100%	True negative
Sjögren's syndrome	N/A (407,746 individuals)	No	0.28-100%	True negative
Connective tissue disorder	3,206	No	>99%	True negative
Diseases of the musculoskeletal system and connective tissue	115,768	No	>99%	True negative
Fibromyalgia related co-morbidities	2,305	No	>99%	True negative
Systemic connective tissue disorders	5,647	No	0.54-100%	True negative
TMD muscular pain Inked with fibromyalgia	2,530	No	0.55-100%	True negative
